# Harnessing CO_2_ Radical Anion-Mediated Electron Transfer for Scalable Copper-Catalyzed Cross-Coupling

**DOI:** 10.1021/jacs.5c18868

**Published:** 2025-12-29

**Authors:** Shuo Wu, Chia-Jung Yang, Mu-Jeng Cheng, Wei Liu

**Affiliations:** † Department of Chemistry, 1757 Virginia Tech, Blacksburg, Virginia 24061, United States; ‡ Department of Chemistry, 34912 National Cheng Kung University, Tainan 701, Taiwan

## Abstract

The inherently sluggish single-electron transfer from copper­(I) complexes to alkyl halides remains a central bottleneck in copper-catalyzed cross-coupling chemistry. Here, we introduce a conceptually distinct strategy that overcomes this limitation by harnessing the unique reactivity of the carbon dioxide radical anion (CO_2_^·–^) to undergo efficient single-electron transfer to alkyl bromides. The strategy relies on the generation of CO_2_^·–^ via Cu-catalyzed C–H bond activation of the formate anion. CO_2_^·–^ then undergoes an efficient single-electron transfer to alkyl bromides to generate alkyl radicals for subsequent Cu-catalyzed transformations. A broad range of unactivated alkyl bromides and structurally diverse nucleophiles—including heteroaryl amines, sulfonamides, anilines, sulfinates, and nitriles—are efficiently coupled to afford C­(sp^3^)–N, C­(sp^3^)–S, and C­(sp^3^)–C bonds in good to excellent yields. The cost-effectiveness and simplicity of this protocol enable decagram-scale synthesis while facilitating rapid reaction optimization and library synthesis for late-stage diversification of drug molecules through high-throughput experimentation.

## Graphical abstract

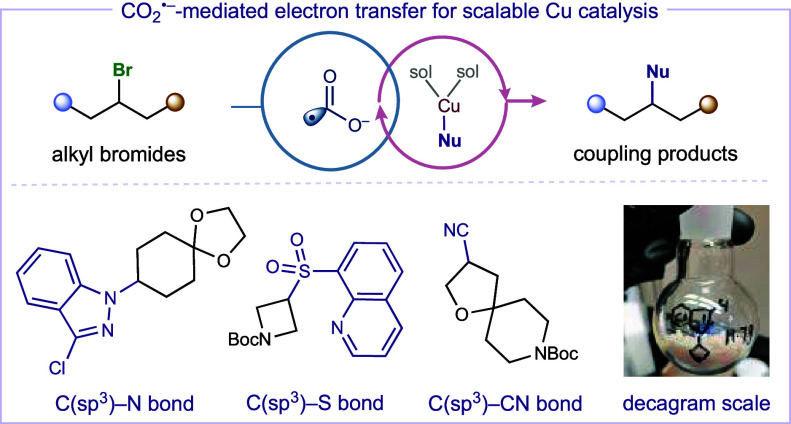

## Introduction

Building on the seminal work by Lovering,^1^ which shows that a higher fraction of sp^3^-hybridized carbons in drug candidates correlates strongly with clinical success, there is a growing interest in the rapid synthesis and evaluation of such sp^3^-rich medicines for disease treatment and prevention.^2^ Consequently, the development of efficient and scalable methods for the construction of C­(sp^3^) centers continues to be a cornerstone in synthetic organic chemistry. In this context, transition-metal-catalyzed cross-coupling reactions are indispensable tools for forging C­(sp^3^)–C and C­(sp^3^)–heteroatom bonds in modern synthetic chemistry.^3^ Copper catalysis, in particular, has emerged as an attractive platform due to its low cost, earth abundance, and unique reactivity with alkyl radicals.^4^ In particular, copper­(II) species are known to rapidly transfer ligands to alkyl radical intermediates to form C–C or C–heteroatom bonds via either Cu^III^ intermediates^5^ or a group transfer pathway.^6^ However, a persistent challenge in Cu-catalyzed cross-coupling of alkyl halides is the sluggish single-electron transfer from Cu­(I) catalysts to these substrates, which hampers the efficient generation of alkyl radicals for subsequent transformations (Figure 1a).

**Figure 1:**
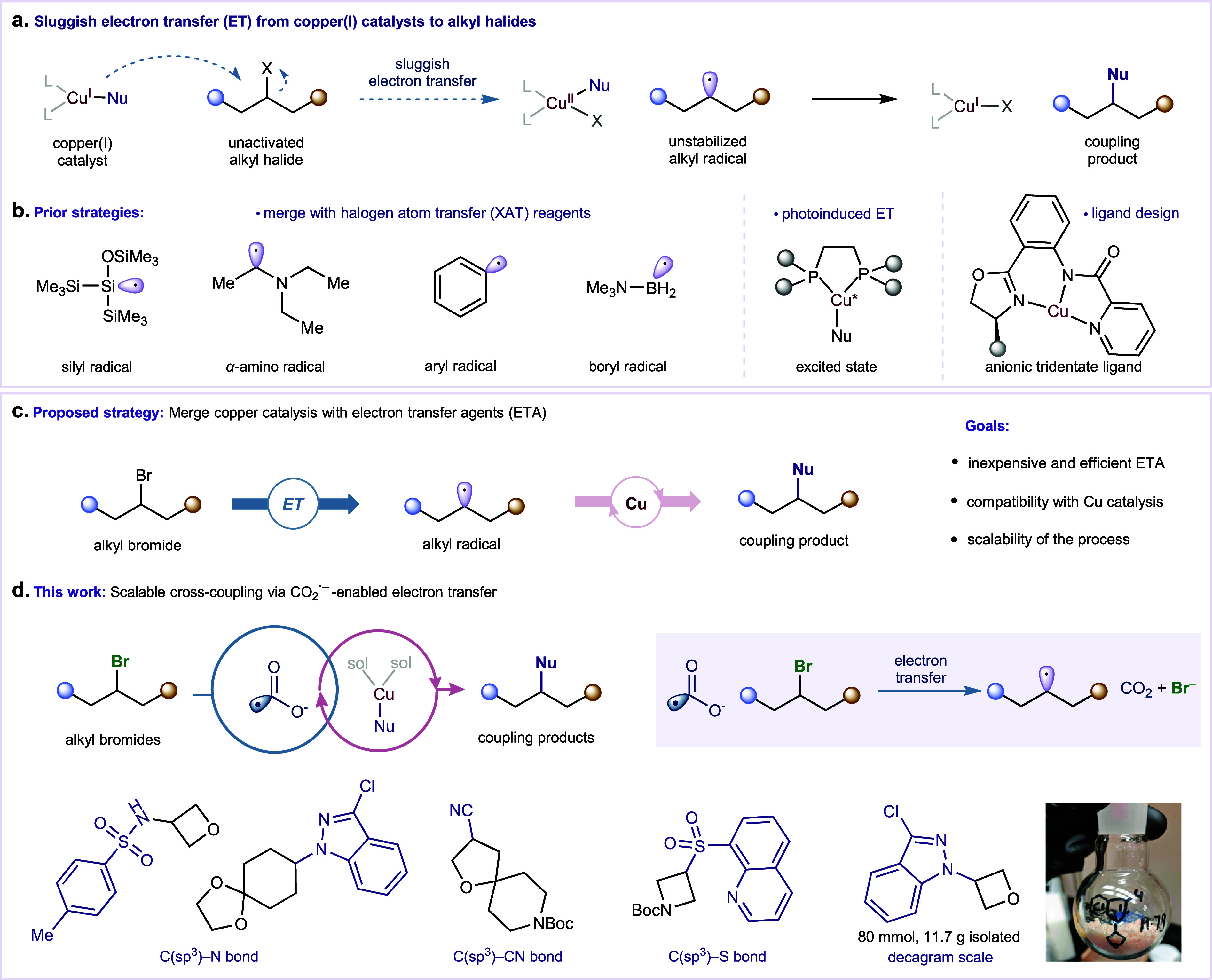
Development of a scalable Cu-catalyzed alkyl bromide cross-coupling by harnessing the electron transfer from the CO_2_ radical anion. (a) Intrinsic limitation of the Cu-catalyzed cross-coupling of alkyl electrophiles. (b) Previous strategies for the Cu-catalyzed cross-coupling of unactivated alkyl halides. (c) Our proposed strategy is merging copper catalysis with single-electron transfer. (d) This work: Cu-catalyzed scalable cross-coupling of alkyl bromides is achieved by harnessing CO_2_^·–^-mediated electron transfer.

To address this SET limitation of copper catalysis, several strategies have been developed (Figure 1b). Seminal work by MacMillan has shown that silyl radicals, generated via photoredox catalysis, are capable of abstracting halogens from alkyl halides to form alkyl radicals for copper catalysis.^7^ Yet, the high cost of these silyl radical precursors (e.g., supersilanol) limits the scalability of these transformations. Subsequent studies by Leonori,^8^ our group,^9^ and others^10^ expanded the toolbox to include α-amino-alkyl, aryl, and amine-ligated boryl radicals that promote halogen atom transfer (XAT), enabling cross-coupling of a diverse range of nucleophiles under copper catalysis. Nonetheless, most of these XAT reagents remain limited to less abundant and more labile alkyl iodides. The Fu and Peters groups have pioneered the use of photoexcited ligated-Cu catalysts to reduce unactivated alkyl halides.^11^ While highly enabling asymmetric catalysis, this approach often necessitates the use of costly phosphine ligands. More recently, Liu and co-workers developed a tridentate anionic ligand that enhances the reducing capability of Cu­(I) catalyst, enabling unactivated alkyl halides to couple with different nucleophiles.^12^ However, the reliance on specially designed ligands limits the practicality and scalability of this approach. Therefore, developing a general and efficient method via a mechanistically distinct pathway for Cu-catalyzed cross-coupling of unactivated alkyl halides remains highly desirable.

We recently questioned whether copper catalysis could instead be merged with a cost-effective single-electron-transfer (SET) agent (Figure 1c). In this scenario, an unactivated alkyl halide would be reduced by an in situ-generated SET reagent, thus circumventing the sluggish SET step between copper­(I) species and alkyl halides. Ideally, such a reagent should be compatible with copper catalysis, engage different nucleophiles, and be cost-effective to enable the process to be readily scalable. In this context, we were intrigued by the recent advancement in the use of carbon dioxide radical anion (CO_2_^·–^), which has emerged as a highly reactive nucleophilic radical species for organic synthesis.^13^ While many of these advancements focus on carboxylation reactions, with either gaseous carbon dioxide^14^ or formate salts^15^ as the precursors, a number of reports have demonstrated that this intermediate could serve as a strong single-electron donor.^16^ For example, pioneering work by Jui disclosed that CO_2_^·–^ is effective in reducing aryl halides, ammonium salts, trifluoroarenes, and aldehydes.^16d^ However, such intriguing reactivity has not yet been employed for transition metal catalysis.

In this work, we harnessed CO_2_^·–^ as a strong electron transfer agent to enable Cu-catalyzed cross-coupling of unactivated alkyl bromides (Figure 1d). We demonstrate that under optimized conditions, a broad scope of unactivated alkyl bromides and diverse nucleophiles can be coupled to construct C­(sp^3^)–N, C­(sp^3^)–S, and C­(sp^3^)–CN bonds using copper catalysis, enabled by the CO_2_^·–^ SET mechanism. We further show that, given the low cost of the reagents, the protocol can be carried out on a decagram scale, offering a potentially practical route to sp^3^-rich molecules of medicinal relevance.

## Results and Discussion

### Hypothesis

A detailed mechanism for the proposed CO_2_^·–^-enabled, Cu-catalyzed coupling reaction is depicted in Figure 2a. We hypothesize that the initial transmetalation of the copper­(I) catalyst **1** with a nucleophile would form a copper-nucleophile (Cu–Nu) complex **2**, which could be oxidized by an *O*-centered radical precursor **3** to afford alkoxy radical **4** and Cu^II^ species **5**. We reasoned that this *O*-centered radical **4** (BDE of O–H ≈ 105 kcal·mol^–1^)^17^ could abstract the hydrogen atom from formate (BDE of H–COO^–^ ≈ 86 kcal·mol^–1^),^18^ thereby generating the highly reducing CO_2_^·–^. A subsequent SET from CO_2_^·–^ to unactivated alkyl bromide **6** would then furnish the corresponding alkyl radical **7** with concurrent release of CO_2_ and bromide. At this stage, we anticipate that the Cu^II^ intermediate **5** would rapidly trap the alkyl radical to generate a high-valent Cu^III^ species **8**, which would undergo C­(sp^3^)–C or C­(sp^3^)–heteroatom bond-forming reductive elimination to afford the desired coupling product **9** while regenerating the Cu^I^ catalyst. A key challenge of the proposed catalytic cycle lies in the generation of a strongly reducing CO_2_^·–^ species under overall oxidizing conditions, which necessitates the identification of a suitable precursor for the *O*-centered radical compatible with the catalytic system.

**Figure 2:**
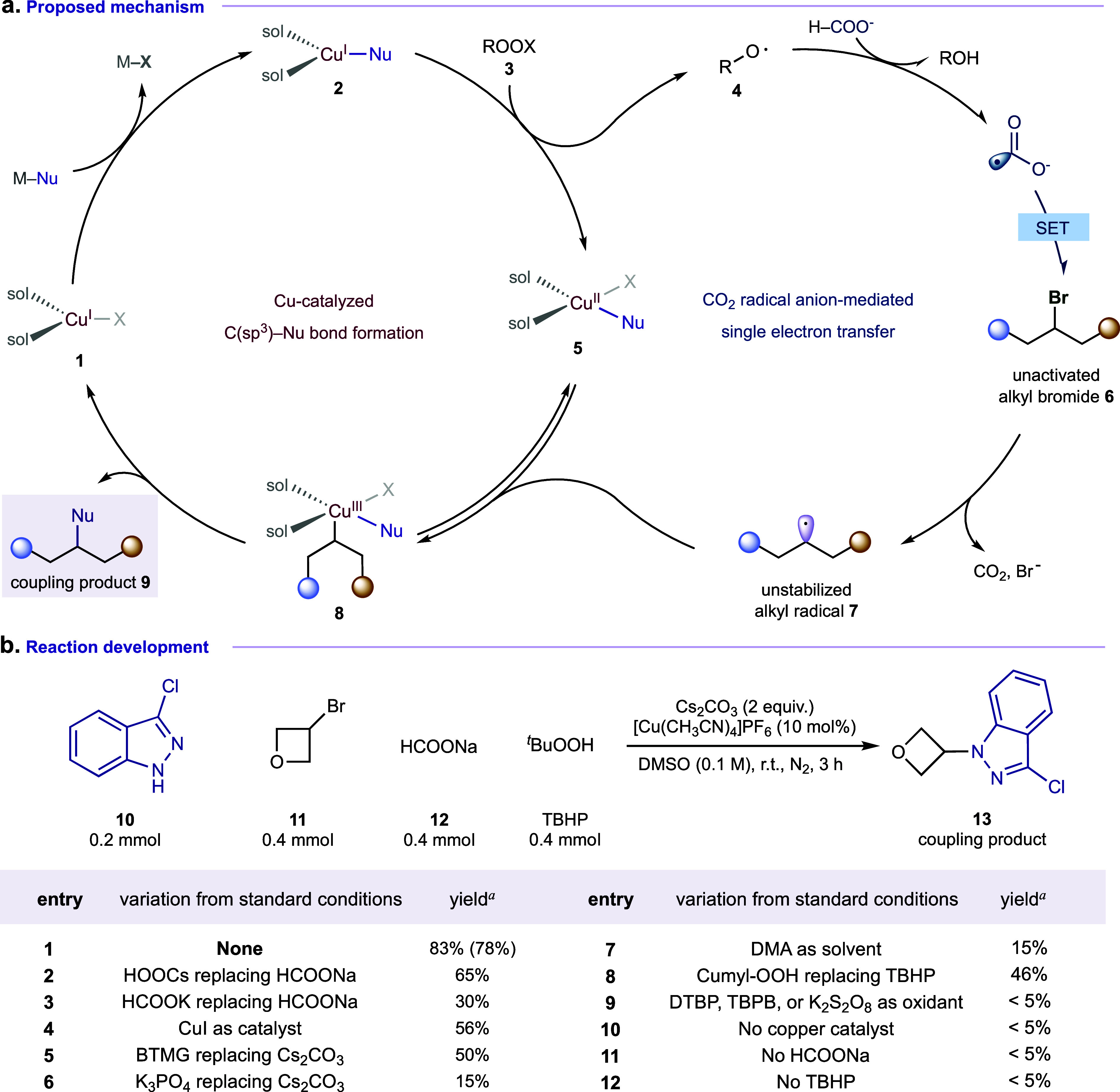
Development of Cu-catalyzed cross-coupling of alkyl bromides enabled by a CO_2_^·–^-mediated electron transfer. (a) Proposed catalytic cycle for the CO_2_^·–^-mediated, Cu-catalyzed cross-coupling of alkyl bromides. (b) Optimization of the reaction conditions. *^a^* Yield was determined by ^1^H NMR using 1,3,5-trimethoxybenzene as the internal standard. Isolated yield shown in parentheses. BTMG, 2-*tert*-butyl-1,1,3,3-tetramethylguanidine; DTBP, di-*tert*-butyl peroxide; TBPB, *tert*-butyl peroxybenzoate.

### Reaction Development

We commenced our studies by developing a Cu-catalyzed, CO_2_^·–^-mediated C­(sp^3^)–N bond-forming reaction using 3-chloro-1*H*-indazole (**10**) and 3-bromooxetane (**11**) as model substrates (Figure 2b). In the presence of [Cu­(CH_3_CN)_4_]­PF_6_ (10 mol %) as the catalyst, sodium formate as the precursor of CO_2_^·–^, *tert*-butyl hydroperoxide (TBHP, 70 wt % in H_2_O) as the precursor of *tert*-butoxy radical, and Cs_2_CO_3_ as the base, **10** reacted smoothly with **11** in DMSO at room temperature to afford the coupling product **13** in 78% isolated yield (**entry 1**). Notably, no exogenous ligands are required to promote this reaction. The requirement of excess TBHP and sodium formate could be due to the quenching of the CO_2_^·–^ by the oxidant. The use of other formate salts, such as cesium formate or potassium formate, was less efficient (**entries 2** and **3**). Alternative copper salts such as copper iodide could also be used as catalysts, albeit with diminished efficiency (**entry 4**). An organic base BTMG could also be used in this transformation (**entry 5**) and was found to be superior for a few other *N*-nucleophiles. Other inorganic bases, such as K_3_PO_4_, are also compatible with this transformation, albeit in diminished yield (**entry 6**). The use of DMSO as the solvent is essential for this transformation, presumably due to its excellent ability to dissolve all reaction components (**entry 7**). While cumene hydroperoxide could replace TBHP with lower efficiency, other commonly employed *O*-centered radical precursors, including di-*tert*-butyl peroxide, *tert*-butyl peroxybenzoate, or potassium persulfate, were ineffective (**entries 8** and **9**). Control experiments confirmed that the presence of sodium formate, TBHP, and copper catalysts is indispensable for this C­(sp^3^)–N coupling reaction (**entries 10**–**12**).

### Mechanistic Studies

Mechanistic studies were then conducted to probe the involvement of CO_2_^·–^ and alkyl radical intermediates in this reaction (Figure 3a). When phenyl vinyl sulfone (**14**) was used in place of the alkyl bromide substrate, the carboxylic acid product (**15**) was isolated in 23% yield. This result is consistent with the formation of CO_2_^·–^, which reacts with **14** via the carboxylation pathway.

**Figure 3:**
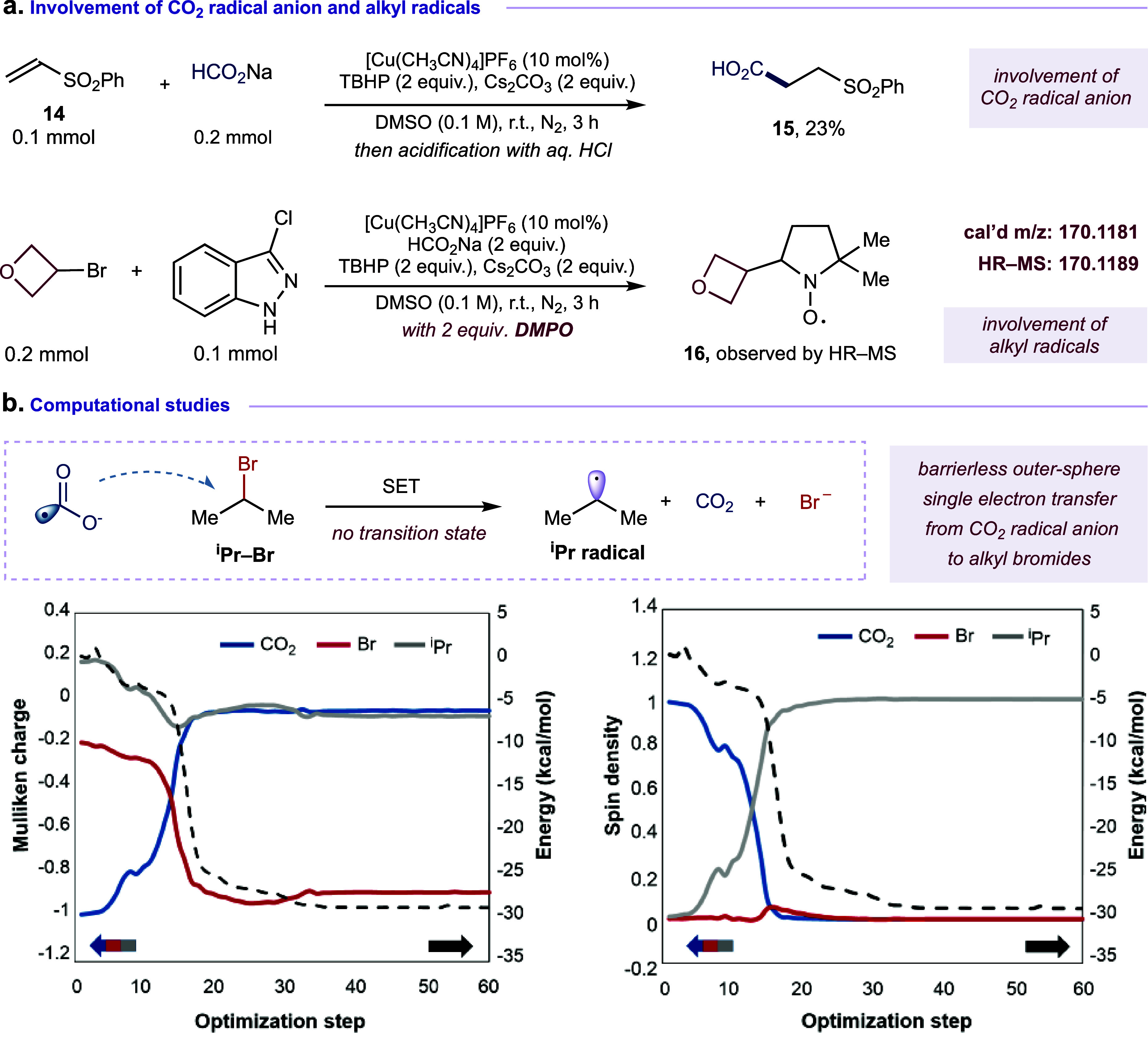
Mechanism investigation of Cu-catalyzed cross-coupling of alkyl bromides enabled by CO_2_^·–^-mediated electron transfer. (a) Mechanistic studies supporting the formation of CO_2_^·–^ and alkyl radical intermediates. (b) DFT calculations at the B3LYP-D3BJ/def2-SVP/SMD­(DMSO) level of theory are on the electron transfer from CO_2_^·–^ to isopropyl bromide.

Additionally, when the standard reaction was conducted in the presence of a radical trap, 3,4-dihydro-2,3-dimethyl-2*H*-pyrrole 1-oxide (DMPO), the formation of the radical addition product **16** was detected by high-resolution mass spectrometry (HR-MS), in agreement with the generation of an alkyl radical.

Moreover, density functional theory (DFT) calculations were performed to probe the reaction between CO_2_^·–^ and an alkyl bromide, using isopropyl bromide (^i^Pr–Br) as a model substrate (Figure 3b). Notably, when the optimized CO_2_^·–^ and ^i^Pr–Br structures were placed in the same simulation cell and subjected to geometry optimization, the system spontaneously yielded CO_2_, Br^–^, and an isopropyl radical (^i^Pr·) (see the Supporting Information and Supporting Movie for details). This observation indicates the absence of a transition state in this reaction, suggesting a barrierless outer-sphere electron transfer process. This is consistent with the strong reducing power of CO_2_^·–^, which can efficiently donate an electron to ^i^Pr–Br. Mulliken charge population and spin density analyses further support this conclusion. As geometry optimization proceeds, the negative charge on CO_2_^·–^ rapidly diminishes, while the charge on Br approaches −1.0 |*e*|. Concurrently, the spin density on CO_2_^·–^ vanishes, whereas that on the ^i^Pr fragment increases to ∼1.0. Collectively, these computational studies highlight the kinetically facile nature of SET from CO_2_^·–^ to an alkyl bromide, the key step enabling the efficient initiation of radical pathways in this coupling reaction.

### Scope of the Reaction

With the optimized CO_2_^·–^-mediated Cu-catalyzed conditions in hand, we next evaluated the generality of this transformation with respect to both the *N*-nucleophiles and the alkyl bromides (Table 1). Indazoles bearing a range of electronic substituents reacted smoothly under standard conditions to deliver the desired products in high yields (**17**–**19**). Azaindole frameworks are also well tolerated, affording the desired products (**20** and **21**) in good yields. In addition, the protocol could be applied to the alkylation of indole scaffolds bearing either electron-withdrawing or electron-donating substituents (**22**–**26**). Pyrazoles, often sensitive to oxidative or basic conditions, underwent coupling efficiently without observable *N*-oxidation or decomposition (**27** and **28**).

**Table 1:**
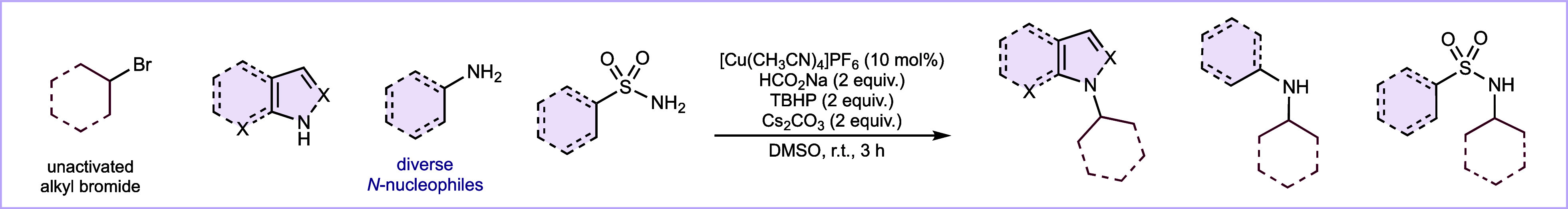

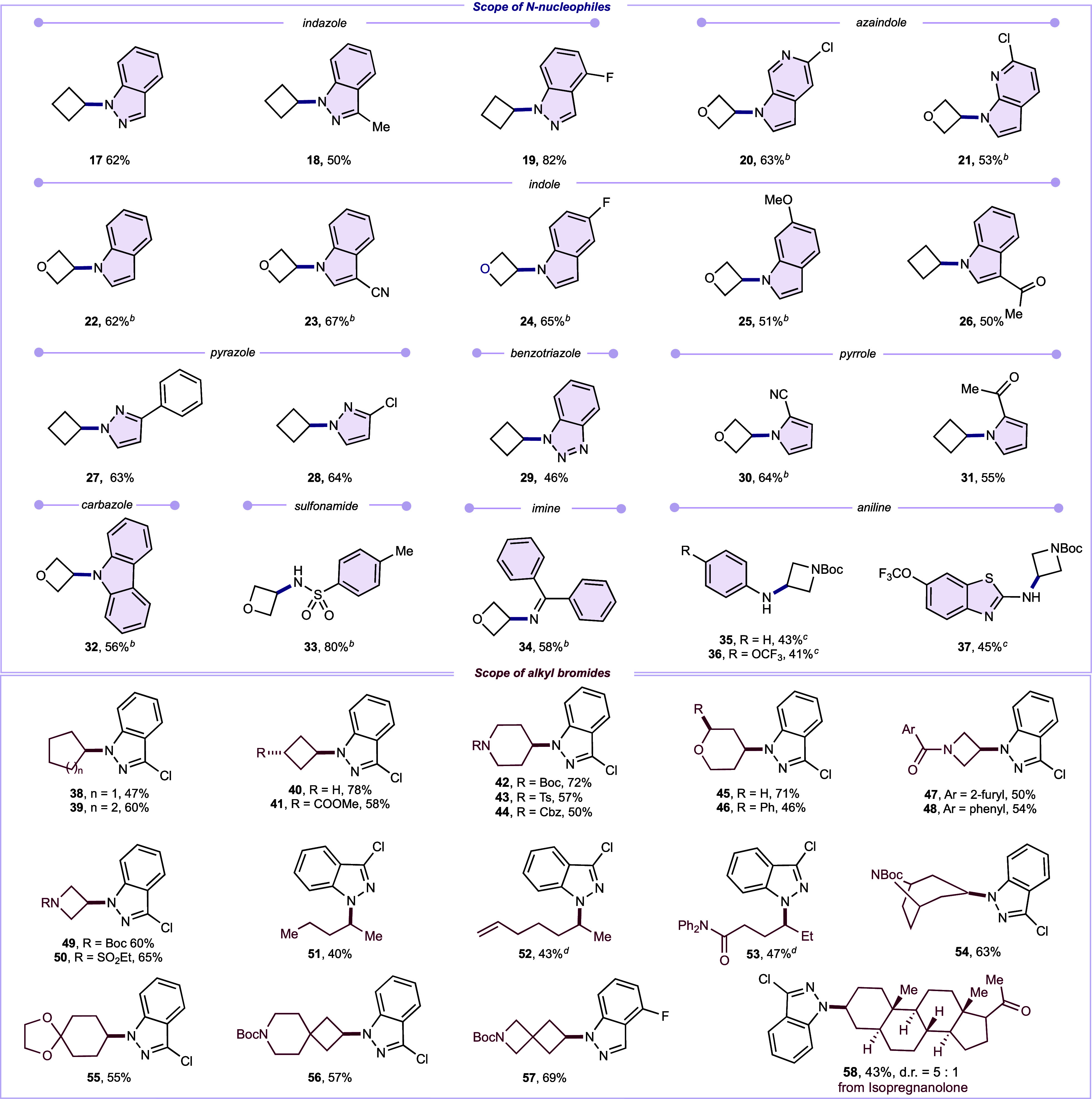
Scope of the CO_2_^·–^-Enabled, Cu-Catalyzed C­(sp^3^)–N Bond Formation^a^

a Reactions were performed on a 0.2 mmol scale of *N*-nucleophiles, with 0.02 mmol [Cu­(CH_3_CN)_4_]­PF_6_, 0.4 mmol of alkyl bromides, base, HCO_2_Na, and TBHP.

b BTMG was used as a base.

c K_3_PO_4_ was used as a base.

d HCO_2_Cs was used as a reductant. Isolated yields were reported. Boc = *tert*-butyloxycarbonyl, Ts = *p*-toluenesulfonyl, Cbz = benzyloxycarbonyl.

Other nitrogen-based nucleophiles such as benzotriazoles (**29**), pyrroles (**30** and **31**), and carbazoles (**32**) were all compatible with this reaction system, affording products in good yields while tolerating reactive motifs such as aryl halides, esters, and carbonyls. Notably, both sulfonamides (**33**) and imine (**34**) could also be engaged in this protocol, highlighting the ability of this system to accommodate weakly nucleophilic nitrogen centers. Anilines (**35**–**37**) were also competent coupling reagents when K_3_PO_4_ was used as the base. Nonetheless, alkyl amines and amides were found to be less effective under the standard reaction conditions (Figure S2).

The coupling reaction also exhibits a broad tolerance toward alkyl bromides. A range of secondary alkyl bromides underwent efficient transformation to furnish the desired products in moderate to excellent yields. Cyclic bromides such as cyclopentyl (**38**), cyclohexyl (**39**), and cyclobutyl bromides (**40** and **41**) were smoothly converted. A variety of brominated saturated heterocyclic substrates, including piperidinyl (**42**–**44**), tetrahydropyranyl-2-yl (**45** and **46**), and azetidinyl bromides (**47**–**50**), were ideal precursors for this coupling reaction. Acyclic alkyl bromides (**51**–**53**) could also undergo smooth conversion to produce the coupling product in a moderate yield. Bicyclic systems, including norbornyl and a group of spiro substrates, were also reactive (**54**–**58**). Functional groups such as alkenes, amides, ethers, carbamates, acetals, esters, and sulfonyl groups were well tolerated in this transformation. Tertiary alkyl bromides, however, could not participate in this transformation, presumably due to the steric hindrance of the tertiary radicals (Figure S2).

### Formation of C–S and C–CN Bonds

Having shown that CO_2_^·–^ could mediate the formation of C­(sp^3^)–N bond formation, we next evaluated whether other nucleophiles could be engaged in this system (Table 2). Alkyl sulfones are ubiquitous and significant motifs in pharmaceuticals and agrochemicals due to their unique biological activities.^19^ They also serve as versatile building blocks for many chemical transformations in organic synthesis.^20^ Conventional substitution of organosulfinates and alkyl halides provides a reliable method for the construction of C­(sp^3^)-sulfonyl bonds.^21^ Despite its efficiency, this method is limited to the use of primary alkyl halides. Thus, an efficient and practical approach for the preparation of alkyl sulfones from readily available secondary alkyl bromides is highly desirable.

**Table 2:**
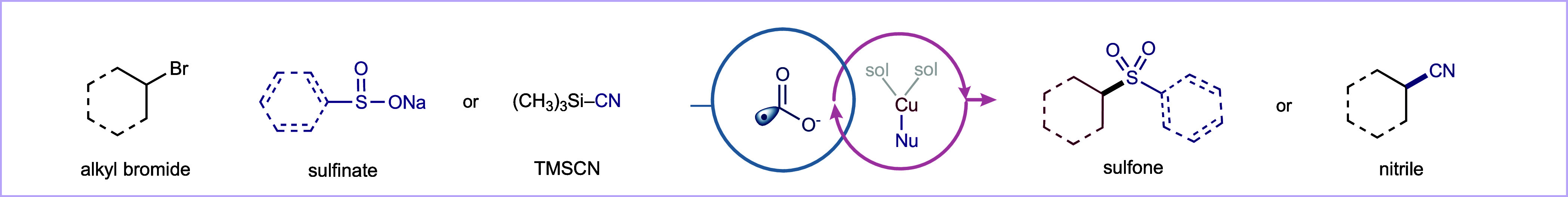

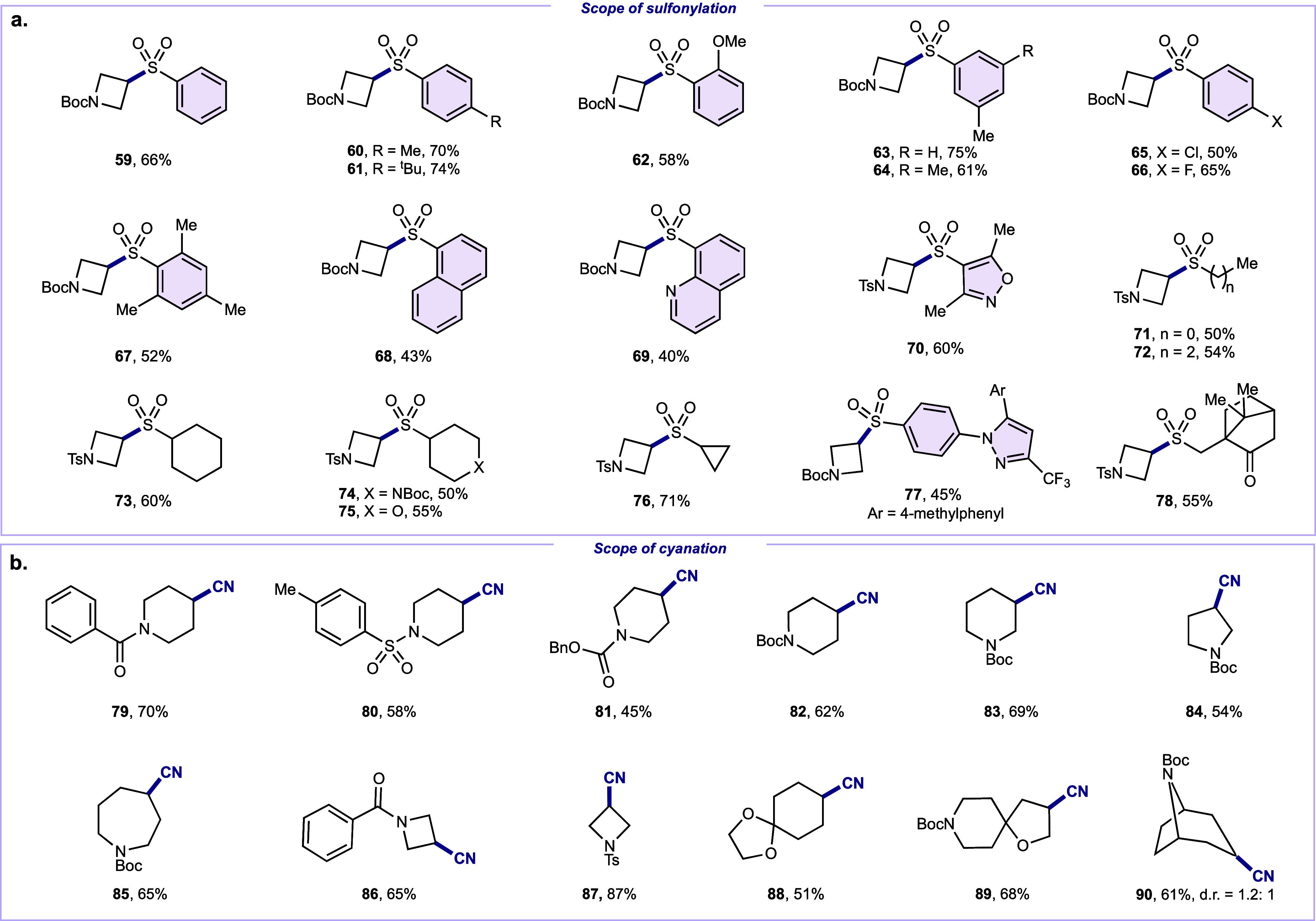
Scope of CO_2_^·–^-Enabled, Cu-Catalyzed C­(sp^3^)–S^a^ and C­(sp^3^)–CN^b^ Bond Formation

a Conditions for sulfonylation: reactions were performed on the 0.2 mmol scale of sulfinates, with 0.02 mmol [Cu­(CH_3_CN)_4_]­PF_6_, 0.4 mmol alkyl bromides, HCO_2_H, HCO_2_Cs, and TBHP at 50 °C for 3 h.

b Conditions for cyanation: reactions were performed on the 0.2 mmol scale of alkyl bromides, with 0.02 mmol CuCN, 0.4 mmol TMSCN, Cs_2_CO_3_, HCO_2_Na, and TBHP at 50 °C for 20 h. Isolated yields were reported. Boc = *tert*-butyloxycarbonyl, Ts = *p*-toluenesulfonyl.

We sought to apply our system to the construction of C­(sp^3^)-sulfonyl bonds by employing organosulfinates as nucleophiles. Under slightly modified conditions, employing cesium formate as a reductant and formic acid as an additive, a diverse array of alkyl sulfones was prepared efficiently at 50 °C. The addition of formic acid was found to increase the yield of this transformation, presumably by buffering the reaction system. As depicted in Table 2a, a group of aromatic sulfinates (**59**–**64**) bearing electron-donating substituents at various positions (*ortho-, meta-*, and *para-*) or halogen atoms (**65** and **66**) were successfully converted into the desired sulfones in satisfactory yields.

The reaction also showed good tolerance toward trisubstituted aryl sulfinates (**67**) as well as valuable heterocyclic moieties, including quinoline and isoxazole (**68**–**70**). Moreover, both primary (**71** and **72**) and secondary alkyl sulfinates, including cyclohexyl (**73**), piperidinyl (**74**), tetrahydropyranyl (**75**), and cyclopropyl (**76**), which are susceptible to oxidation conditions that can lead to SO_2_ extrusion,^22^ were also compatible with this coupling reaction, affording the corresponding sulfone products in good yields. Drug-like molecules, such as camphor- and celecoxib-based sulfinates, which can be readily prepared in a single step, are also compatible coupling partners, delivering structurally complex alkyl sulfones (**77** and **78**).

Building on this established CO_2_^·–^-mediated Cu-catalyzed platform, we also achieved the cyanation of diverse alkyl bromides using TMSCN as the cyanide source (Table 2b). Under the optimal conditions, a variety of cyclic secondary alkyl bromides showcased good reactivity, affording cyclic alkyl nitrile products derived from piperidines (**79**–**83**), pyrrolidines (**84**), azepane (**85**), and azetidines (**86** and **87**). Spiro and bridged alkyl bromides also proved to be effective for the cyanation reaction (**88**–**90**).

### Synthetic Utility

Finally, we aimed to demonstrate the synthetic potential of these protocols in the pharmaceutical industry, encompassing both medicinal and process chemistry applications. The operational simplicity and mild conditions render the reaction rapidly amenable to high-throughput experimentation (HTE) for rapid optimization and library synthesis. In a 96-well plate, four representative alkyl bromides (**B1**–**B4**) and two bases (Cs_2_CO_3_ and BTMG) were screened against 12 structurally diverse drug molecules, which contain sulfonamides (**N1**–**N4**), azaindoles (**N5** and **N6**), indole (**N7**), sulfimide (**N8**), imidazole (**N9**), heteroaryl amines (**N10**–**N11**), and carbazole (**N12**) (Figure 4a). To our delight, over 80% of the reactions generated detectable coupling products by HPLC, and approximately 50% exhibited product formation with >20% liquid chromatography area percentage (LCAP) yields (Figure 4b).

**Figure 4:**
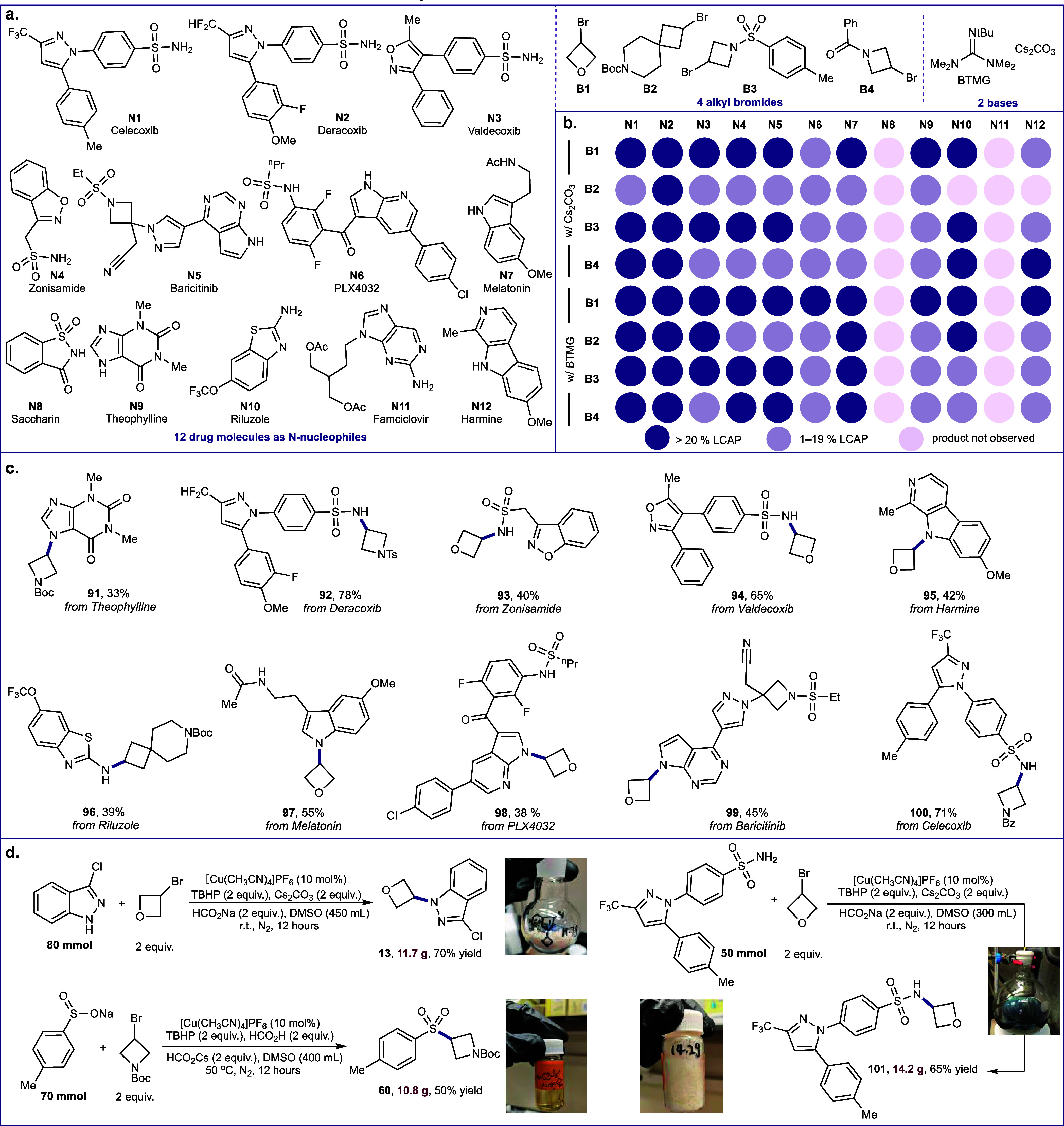
Synthetic utility in the pharmaceutical industry and decagram-scale synthesis. (a) Structure of molecules used for high-throughput experimentations. (b) Results of high-throughput experiments in a 96-well plate. LCAP: liquid chromatography area percentage. (c) Cross-coupling of complex molecules at the 0.2 mmol scale. (d) Decagram-scale synthesis.

The success of the HTEs was confirmed by isolation on a preparative scale (0.2 mmol), affording a broad range of densely functionalized C­(sp^3^)–N coupling products (**91**–**100**) in synthetically useful yields (Figure 4c).

Finally, the low cost and commercial availability of the SET reagents (aq. TBHP, ∼$50/mol and HCOONa, ∼$10/mol) further enable ready scalability of the protocol (Figure 4d). Two representative C­(sp^3^)–N and C­(sp^3^)–S bond-forming reactions were readily scaled up to the decagram scale in a good yield. The decagram-scale synthesis has also been applied to the late-stage diversification of a drug molecule, celecoxib, affording alkylation product **101** (14.2 g) in 65% yield without any specialized equipment.

## Conclusions

In summary, we have developed a scalable and operationally simple Cu-catalyzed cross-coupling platform that harnesses CO_2_^·–^ as a cost-effective and robust SET reagent to enable the functionalization of unactivated alkyl bromides. This approach exhibits a broad substrate scope, including diverse *N*-nucleophiles and structurally complex alkyl bromides. Moreover, the protocol can be readily extended to C­(sp^3^)–S and C­(sp^3^)–CN bond formation. Importantly, the simplicity and low cost of the reagents enable miniaturization for HTE and scale-up to the decagram scale, making this method particularly attractive for applications in high-throughput reaction development, library synthesis, and late-stage diversification of drug molecules. We anticipate that this CO_2_^·–^-enabled platform will provide new opportunities for sustainable and scalable synthesis of sp^3^-rich molecules with broad applications for the pharmaceutical industry.

## Supplementary Material





## Notes

SET movie (MP4)
General information, reaction optimization details, supplementary figures, experimental details, characterizations of new compounds, copies of NMR spectra (PDF)
