# Supplementary material for: Harnessing CO2 Radical Anion-Mediated Electron Transfer for Scalable Copper-Catalyzed Cross-Coupling
Source: J Am Chem Soc. 2025 Dec 29;148(1):1717–27. doi: 10.1021/jacs.5c18868 (PMC12814328; doi:10.1021/jacs.5c18868)
Supplement: Supplementary file 2 [file ja5c18868_si_002.pdf]

Supporting Information

Harnessing CO_2 Radical Anion-Mediated Electron Transfer for Scalable Copper-Catalyzed Cross-Coupling

Shuo Wu[a], Chia-Jung Yang[b], Mu-Jeng Cheng[b], Wei Liu[a]*

[a]Department of Chemistry, Virginia Tech, Blacksburg, VA 24061, United States
[b]Department of Chemistry, National Cheng Kung University, Tainan 701, Taiwan

* liuwei@vt.edu

Table of Contents

A. Experimental Section S3
B. Synthesis of Substrates S4
B1. Synthesis of Alkyl Bromides S4
B2. Synthesis of Sodium Arylsulfinate S4
B3. Characterization of Unreported Products S5
C. Experimental Procedures S7
C1. The Set-up and Procedure for Decagram-scale Reaction S7
C2. Copper-Catalyzed Amination of Alkyl Bromides S8
C2.1. Optimization Studies S8
C2.2. General Procedure I: Amination of Alkyl Bromides S8
C2.3. Characterization of Products S9
C3. Copper-Catalyzed Sulfonylation of Alkyl Bromides S31
C3.1. Optimization Studies S31
C3.2. General Procedure II: Sulfonylation of Alkyl Bromides S31
C3.3. Characterization of Products S32
C4. Copper-Catalyzed Cyanation of Alkyl Bromides S40
C4.1. Optimization Studies S40
C4.2. General Procedure III: Cyanation of Alkyl Bromides S40
C4.3. Characterization of Products S41
C5. Other Alkyl Bromides and Nucleophiles Evaluated. S45
D. High-throughput Experimentation (HTE) for Reaction Optimization S45
E. Mechanistic Studies S47
E1. Proof of Alkyl Radical Generation S47
E2. Capture of $CO_2^{\bullet-}$ Intermediate using Radical Acceptor S48
E3. Density Functional Theory (DFT) Computational Studies S49
F. References S64
G. NMR Spectra of Substrates S65

A. Experimental Section

General Information: Commercial reagents were purchased from Fisher Scientific, Millipore-Sigma, Ambeed, ChemScene or AKSci, and were used as received. TBHP (70% aqueous solution) was purchased from Millipore-Sigma. DMSO was purchased from Millipore-Sigma and was stored in glovebox. All reactions were carried out in oven-dried glassware under an atmosphere of nitrogen unless otherwise noted. Flash column chromatography was performed using F60 silica gel (40-63 μm, 230-400 mesh, 60 Å) purchased from Silicycle. Thin-layer chromatography (TLC) was performed on Silicycle 250 mm silica gel F-254 plates, and visualization was affected by observation of fluorescence-quenching with ultraviolet light and staining with $KMnO_4$. GC/MS analysis was performed on a Thermo-Fischer Scientific ISQ QD single quadrupole mass spectrometer. ^{1}H, ^{13}C and ^{19}F NMR spectra were recorded on Bruker 400 (400, 101, and 376 MHz). Data is reported in ppm using $CDCl_3$ as the solvent unless otherwise specified. Data for ^{1}H NMR, ^{19}F NMR and ^{13}C NMR are reported as follows: chemical shift (δ ppm), multiplicity (s = singlet, d = doublet, t = triplet, m = multiplet, br = broad), integration, coupling constant (Hz). High-resolution mass spectrometry (HRMS) analyses were performed at the Mass Spectrometry Research Incubator at Virginia Tech.

B. Synthesis of Substrates

B1. Synthesis of Alkyl Bromides

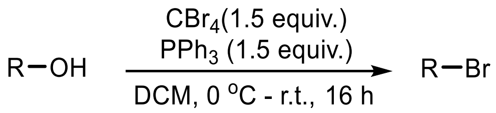

General procedure A: The reaction was performed according to the reported procedure[1] To a dry solution of alcohol (5 mmol, 1.0 equiv.) and PPh_3 (7.5 mmol, 1.5 equiv.) in DCM (12 mL) at 0 °C was added dropwise a solution of CBr_4 (7.5 mmol, 1.5 equiv.) in DCM (8 mL). The reaction was stirred for 16 h under nitrogen and the precipitate was removed via filtration. The filtrate was then concentrated in vacuo and the product isolated by flash column chromatography to give the alkyl bromide.

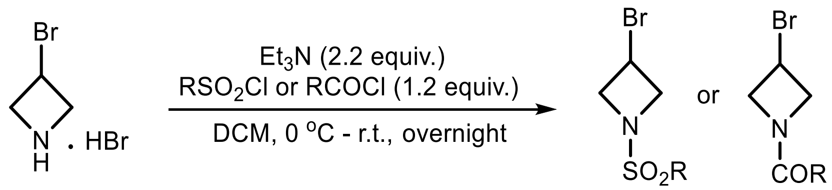

General procedure B: The reaction was performed according to the reported procedure.[2] Et_3N (11.0 mmol, 2.2 equiv.) was slowly added into to a mixture of 4-bromopiperidine hydrobromide (5 mmol, 1.0 equiv.) and sulfonyl chlorides or acyl chlorides (4.194 g, 22 mmol, 1.1 equiv.) in DCM (25 mL, 0.2 M) at 0 °C while stirring. The reaction mixture was warmed to room temperature overnight, and diluted with aqueous HCl (2 M, 40 mL). The layers were separated, and the aqueous layer was extracted with DCM (3 x 10 mL). The combined organic layers were washed with brine (10 mL), dried (Na_2SO_4), filtered, and evaporated in vacuo. The impure crude was purified by flash chromatography.

B2. Synthesis of Sodium Arylsulfinate

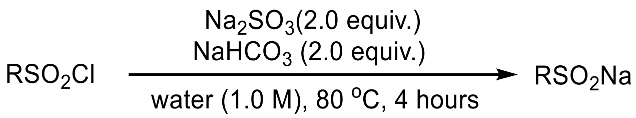

The reaction was performed according to the reported procedure.[3] Sodium sulfite (6 mmol, 2 eq.), sodium bicarbonate (6 mmol, 2 eq.) and the corresponding aryl sulfonyl chloride (3 mmol, 1 eq.) were dissolved in distilled water (3 mL). The reaction mixture was stirred for 4 h at 80 °C. After cooling down to room temperature, water was removed in vacuo. 10 mL of ethanol was then added to this white residue and the resulting heterogeneous solution was filtered. The filtrate was concentrated under reduced pressure and the desired sodium aryl sulfinates were obtained as crystalline powders.

B3. Characterization of Unreported Products

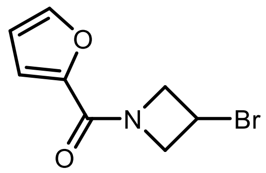

(3-bromoazetidin-1-yl)(furan-2-yl)methanone

The title compound was prepared according to the ***General Procedure B*** and isolated by flash chromatography (Hexane: EtOAc = 10:1) as a yellow solid (62% yield).

^{1}H NMR (400 MHz, CDCl$_3$) δ 7.49 (dd, J = 1.7, 0.8 Hz, 1H), 7.10 (dd, J = 3.5, 0.8 Hz, 1H), 6.49 (dd, J = 3.5, 1.8 Hz, 1H), 5.20 – 4.30 (m, 5H).

^{13}C NMR spectrum was complicated due to the presence of rotamers.

^{13}C NMR (101 MHz, CDCl$_3$) δ 158.5, 147.6, 144.8, 115.9, 115.9, 111.7, 63.0, 59.3, 45.8, 33.5.

HRMS (ESI): $C_8H_9BrNO_2^+$ (M+H)$^+$: 229.9817, found: 229.9809.

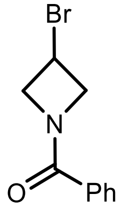

(3-bromoazetidin-1-yl)(phenyl)methanone

The title compound was prepared according to the ***General Procedure B*** and isolated by flash chromatography (Hexane: EtOAc = 10:1) as a white solid (73% yield).

^{1}H NMR (400 MHz, CDCl$_3$) δ 7.64 – 7.58 (m, 2H), 7.51 – 7.44 (m, 1H), 7.44 – 7.38 (m, 2H), 4.86 – 4.78 (m, 2H), 4.65 – 4.58 (m, 1H), 4.56 – 4.37 (m, 2H).

^{13}C NMR spectrum was complicated due to the presence of rotamers.

^{13}C NMR (101 MHz, CDCl$_3$) δ 170.4, 132.5, 131.4, 128.5, 127.9, 63.8, 59.7, 45.4, 33.0.

HRMS (ESI): $C_{10}H_{11}BrNO^+$ (M+H)$^+$: 240.0024, found: 240.0016.

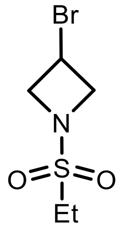

3-bromo-1-(ethylsulfonyl)azetidine

The title compound was prepared according to the ***General Procedure B*** and isolated by flash chromatography (Hexane: EtOAc = 10:1) as a colorless liquid (66% yield).

^{1}**H NMR (400 MHz, CDCl$_3$)** δ 4.58 – 4.50 (m, 1H), 4.48 –4.40 (m, 2H), 4.21 –4.14 (m, 2H), 2.96 (q, *J* = 7.4 Hz, 2H), 1.32 (t, *J* = 7.4 Hz, 3H).

^{13}**C NMR (101 MHz, CDCl$_3$)** δ 60.2, 46.2, 31.9, 7.9.

HRMS (ESI): $C_5H_{11}BrNO_2S^+$ $(M+H)^+$: 227.9694, found: 227.9690.

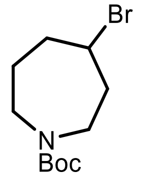

***tert*-butyl 4-bromoazepane-1-carboxylate**

The title compound was prepared according to the ***General Procedure A*** and isolated by flash chromatography (Hexane: EtOAc = 80:1) as a colorless liquid (with rotamer, 65% yield).

^{1}**H NMR (400 MHz, CDCl$_3$)** δ 4.42 – 4.30 (m, 1H), 3.55 – 3.25 (m, 4H), 2.32 – 2.20 (m, 1H), 2.20 – 2.05 (m, 3H), 2.02 – 1.88 (m, 1H), 1.76 – 1.65 (m, 1H), 1.45 (s, 9H).

^{13}C NMR spectrum was complicated due to the presence of rotamers.

^{13}**C NMR (101 MHz, CDCl$_3$)** δ 155.4&155.4, 79.5&79.5, 54.0&53.9, 46.1&45.2, 43.3&42.9, 39.8&39.4, 36.8&36.6, 28.5&28.5, 25.1&24.9.

HRMS (ESI): $C_{11}H_{21}BrNO_2^+$ $(M+H)^+$: 278.0756, found: 278.0754.

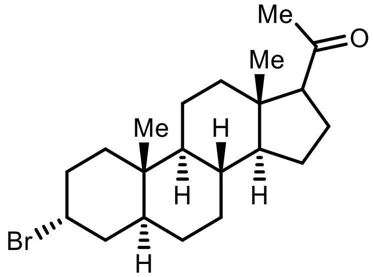

1-((3*R*,5*S*,8*R*,9*S*,10*S*,13*S*,14*S*)-3-bromo-10,13-dimethylhexadecahydro-1*H*-cyclopenta[*a*]phenanthren-17-yl)ethan-1-one

The title compound was prepared according to the ***General Procedure A*** and isolated by flash chromatography (Hexane: EtOAc = 80:1) as a colorless liquid (56% yield, d.r.= 10:1).

^{1}**H NMR (400 MHz, CDCl$_3$)** δ 4.80 – 4.60 (m, 1.1H), 2.78 (dd, *J* = 8.4, 2.6 Hz, 0.1H), 2.53 (t, *J* = 8.8 Hz, 1H), 2.19 – 2.07 (m, 4.4H), 2.04 – 1.88 (m, 3.3H), 1.77 – 1.60 (m, 6.6H), 1.55 – 1.10 (m, 11H), 1.05 – 0.95 (m, 1.1H), 0.92 – 0.85 (m, 1.1H), 0.78 (s, 3H), 0.77 (s, 0.6H), 0.60 (s, 3H).

^{13}**C NMR (101 MHz, CDCl$_3$)** δ 212.6&209.6, 63.8&61.3, 56.7&55.6, 55.9&53.2, 53.8&50.3, 45.8&44.2, 40.1&40.0, 39.0&39.0, 37.3&36.2, 36.3&35.7, 35.4&35.3, 32.9&32.9, 32.8&31.7, 32.0&31.5, 31.0&31.0, 27.8&27.8, 25.8&24.4, 24.3&22.8, 20.9&20.8, 20.7&13.5, 12.3&12.3.

HRMS (ESI): $C_{21}H_{34}BrO^+$ $(M+H)^+$: 381.1793, found: 381.1788.

C. Experimental Procedures

C1. The Set-up and Procedure for Decagram-scale Reaction

The decagram-scale reaction was performed at room temperature for 12 hours.

Procedure: In a glove box, to a 1L round-bottomed flask, $Cu(CH_3CN)_4PF_6$, sodium formate, Cs_2CO_3 and N-containing nucleophiles (1 equiv.) were sequentially added, the flask was then sealed with a rubber septum. Outside the glove box, DMSO (around 400 mL) and alkyl halides were added *via* syringe. TBHP was then added slowly via syringe while the flask was cooled in an ice bath. The flask was sealed with parafilm and then stirred at room temperature for 12 hours (*Note: A large amount of CO_2 gas is released during the reaction, so it is recommended to connect a balloon filled with a small amount of N_2.*). After the reaction was complete, 100 mL of saturated Na_2SO_3 solution was added to flask to quench the remaining TBHP. Then, the reaction mixture was transferred to an extraction funnel, 300 mL of H_2O and 100 mL of brine were added and the organic layer was extracted with EtOAc. The organic layer was washed with brine twice. The combined organic layers were dried over anhydrous Na_2SO_4, filtered, and concentrated to dryness. The crude residue was purified by column chromatography to afford the desired products.

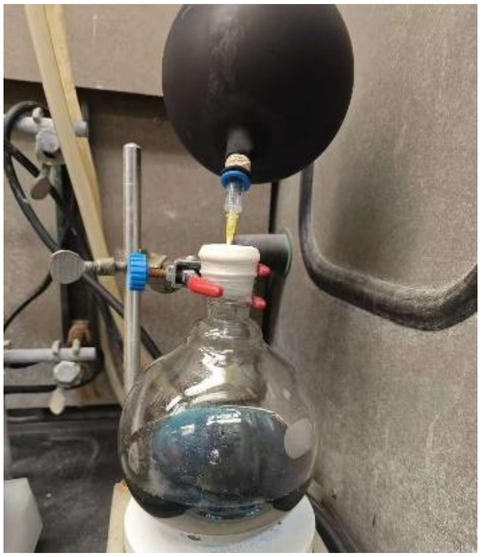

Figure S1: Setup for the decagram-scale reaction at room temperature.

C2. Copper-Catalyzed Amination of Alkyl Bromides

C2.1. Optimization Studies

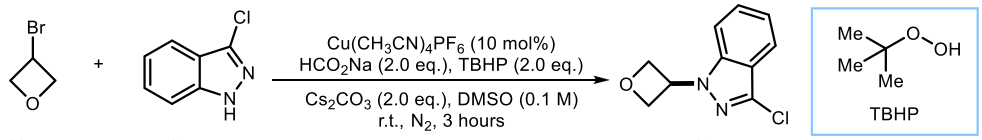

11, 2 equiv. **10**, 0.2 mmol **13**

Table S1. Optimization of the model reaction

Entry	Deviations	NMRy **13** (%)
1	none	83 (78)[a]
2	No **Cu**	< 5%
3	No TBHP, HCO_2Na or Base	< 5%
4	Under air	50%
5	HCO_2K instead of HCO_2Na	30%
6	HCO_2Cs instead of HCO_2Na	65%
7	$Cu(CH_3CN)_4BF_4$ (10 mol%)	56%
8	CuI (10 mol%)	56%
9	CuBr (10 mol%)	60%
10	$Cu(OAc)_2$ (10 mol%)	36%
11	$Cu(OTf)_2$ (10 mol%)	53%
12	CuOAc (10 mol%)	57%
13	CH_3CN as solvent	0%
14	DMA as solvent	21%
15	DMF as solvent	15%
16	K_3PO_4 as Base	15%
17	K_2CO_3 as Base	12%
18	TMG as Base	0
19	BTMG as Base	50%
20	CHP as oxidant	46%
21	DTBP, TBPB or $K_2S_2O_8$ as oxidant	0
22	TBHP in decane	73%

All reactions were performed on a 0.1 mmol scale; yield of **13** determined by ^{1}H NMR analysis of the crude reaction mixture by comparison with 1,3,5-trimethoxybenzene as internal standard. [a] Yield of isolated **13**. TMG: 1,1,3,3-Tetramethylguanidine; BTMG: 2-*tert*-Butyl-1,1,3,3-tetramethylguanidine; DTBP: di-*tert*-butyl peroxide; TBPB: *tert*-butyl peroxybenzoate; CHP: cumene hydroperoxide.

C2.2. General Procedure I: Amination of Alkyl Bromides

In a glove box, to a 4 mL glass vial, $Cu(CH_3CN)_4PF_6$ (7.5 mg, 0.02 mmol, 10 mol%), sodium formate (27.2 mg, 0.4 mmol, 2.0 equiv.), Cs_2CO_3 (130 mg, 0.4 mmol, 2.0 equiv.), *N*-containing nucleophiles (if solid, 0.2 mmol, 1 equiv.) and alkyl bromides (if solid, 0.6 mmol, 2 equiv.) were sequentially added, then the vial was sealed with a screw-top cap with septum. Outside the glove box, DMSO (0.1 M, 2.0 mL), alkyl bromides (if liquid, 0.6 mmol, 2.0 equiv.) or *N*-containing nucleophiles (if liquid, 0.2 mmol, 1 equiv.) followed by TBHP (60 μL, 0.4 mmol, 2.0 equiv.) were added *via* syringe. The vial was sealed with parafilm and then stirred at room temperature for 3 hours. After the reaction was complete, the reaction mixture was

transferred to an extraction funnel, 10 mL of H_2O and 2 mL of brine were added and the organic layer was extracted with EtOAc. The organic layer was washed with brine twice. The combined organic layers were dried over anhydrous Na_2SO_4, filtered, and concentrated to dryness. The crude residue was purified by column chromatography to afford the corresponding product in the stated yield with >95% purity according to ^{1}H NMR analysis.

C2.3. Characterization of Products

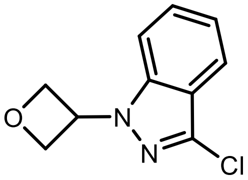

3-chloro-1-(oxetan-3-yl)-1*H*-indazole (13)

The title compound was prepared according to the ***General Procedure I*** *(Cs₂CO₃ as base)* and isolated by flash chromatography (Hexane: EtOAc = 20:1) as a yellow solid (33 mg, 78% yield).

^{1}H NMR (400 MHz, $CDCl_3$) δ 7.72 – 7.66 (m, 1H), 7.50 – 7.42 (m, 2H), 7.26 – 7.21 (m, 1H), 5.78 – 5.68 (m, 1H), 5.28 (dd, *J* = 7.1, 6.3 Hz, 2H), 5.09 (dd, *J* = 7.9, 6.7 Hz, 2H).

^{13}C NMR (101 MHz, $CDCl_3$) δ 140.4, 134.1, 127.9, 121.9, 121.7, 120.2, 109.2, 77.2, 52.9.

Matching reported literature data.[4]

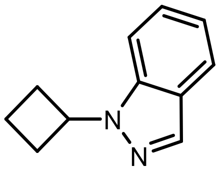

1- cyclobutyl-1*H*-indazole (17)

The title compound was prepared according to the ***General Procedure I*** *(Cs_2CO_3 as base)* and isolated by flash chromatography (Hexane: EtOAc = 100:1) as a yellow liquid (21 mg, 62% yield).

^{1}H NMR (400 MHz, $CDCl_3$) δ 8.04 (s, 1H), 7.74 – 7.70 (m, 1H), 7.47 – 7.42 (m, 1H), 7.38 – 7.33 (m, 1H), 7.16 – 7.10 (m, 1H), 5.14 – 5.03 (m, 1H), 2.88 – 2.76 (m, 2H), 2.58 – 2.49 (m, 2H), 2.02 – 2.90 (m, 2H).

^{13}C NMR (101 MHz, $CDCl_3$) δ 138.9, 132.9, 125.9, 124.2, 121.1, 120.5, 109.2, 52.5, 29.9, 15.1.

Matching reported literature data.[5]

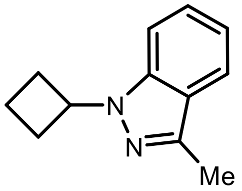

1- cyclobutyl-3-methyl-1*H*-indazole (18)

The title compound was prepared according to the ***General Procedure I*** *(Cs$_2$CO$_3$ as base)* and isolated by flash chromatography (Hexane: EtOAc = 100:1) as a yellow liquid (18 mg, 50% yield).

^{1}H NMR (400 MHz, CDCl$_3$) δ 7.66 – 7.61 (m, 1H), 7.41 – 7.31 (m, 2H), 7.12 – 7.07 (m, 1H), 5.06 – 4.96 (m, 1H), 2.86 – 2.74 (m, 2H), 2.60 (s, 3H), 2.54 – 2.45 (m, 2H), 2.00 – 1.85 (m, 2H).

^{13}C NMR (101 MHz, CDCl$_3$) δ 141.4, 139.8, 125.9, 123.5, 120.4, 119.6, 109.1, 52.2, 30.0, 15.0, 12.0.

HRMS (ESI): $C_{12}H_{15}N_2^+$ (M+H)$^+$: 187.1235, found: 187.1236.

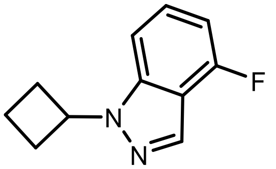

1-cyclobutyl-4-fluoro-1*H*-indazole (19)

The title compound was prepared according to the ***General Procedure I*** *(Cs$_2$CO$_3$ as base)* and isolated by flash chromatography (Hexane: EtOAc = 100:1) as a yellow liquid (31 mg, 82% yield).

^{1}H NMR (400 MHz, CDCl$_3$) δ 8.06 (s, 1H), 7.25 – 7.20 (m, 1H), 7.20 – 7.14 (m, 1H), 6.76 – 6.67 (m, 1H), 5.06 – 4.95 (m, 1H), 2.83 – 2.71 (m, 2H), 2.55 – 2.45 (m, 2H), 2.01 – 1.85 (m, 2H).

^{13}C NMR (101 MHz, CDCl$_3$) δ 155.8 (d, J = 252.2 Hz), 141.6 (d, J = 9.2 Hz), 129.4 (d, J = 2.0 Hz), 126.9 (d, J = 7.7 Hz), 114.5 (d, J = 23.2 Hz), 105.2 (d, J = 4.2 Hz), 104.7 (d, J = 18.5 Hz), 52.8, 29.9, 15.1.

^{19}F NMR (376 MHz, CDCl$_3$) δ -118.05 (dd, J = 9.8, 4.8 Hz, 1F)

HRMS (ESI): $C_{11}H_{12}FN_2^+$ (M+H)$^+$: 191.0985, found: 191.0985.

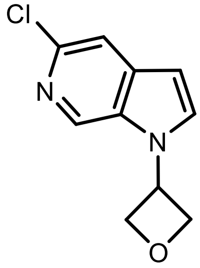

5-chloro-1-(oxetan-3-yl)-1*H*-pyrrolo[2,3-*c*]pyridine (20)

The title compound was prepared according to the ***General Procedure I*** *(BTMG as base)* and isolated by flash chromatography (Hexane: EtOAc = 1:1) as a colorless liquid (26 mg, 63% yield).

^{1}H NMR (400 MHz, CDCl$_3$) δ 8.66 – 8.62 (m, 1H), 7.62 (d, J = 3.3 Hz, 1H), 7.52 (d, J = 1.0 Hz, 1H), 6.57 – 6.54 (m, 1H), 5.63 – 5.54 (m, 1H), 5.21 (dd, J = 7.4, 7.4 Hz, 2H), 5.01 (dd, J = 7.4, 5.9 Hz, 2H).

^{13}C NMR (101 MHz, CDCl$_3$) δ 141.0, 136.9, 132.1, 131.7, 130.7, 115.1, 102.0, 77.7, 51.4.

HRMS (ESI): $C_{10}H_{10}ClN_2O^+$ (M+H)$^+$: 209.0482, found: 209.0480.

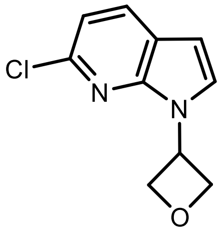

6-chloro-1-(oxetan-3-yl)-1*H*-pyrrolo[2,3-*b*]pyridine (21)

The title compound was prepared according to the ***General Procedure I*** *(BTMG as base)* and isolated by flash chromatography (Hexane: EtOAc = 15:1) as a yellow solid (22 mg, 53% yield).

^{1}H NMR (400 MHz, CDCl$_3$) δ 7.85 (d, *J* = 8.2 Hz, 1H), 7.70 (d, *J* = 3.7 Hz, 1H), 7.09 (d, *J* = 8.1 Hz, 1H), 6.60 (d, *J* = 3.6 Hz, 1H), 6.14 – 6.05 (m, 1H), 5.18 (dd, *J* = 7.4, 7.4 Hz, 2H), 4.95 (dd, *J* = 6.6, 6.6 Hz, 2H).

^{13}C NMR (101 MHz, CDCl$_3$) δ 146.5, 144.8, 131.4, 124.5, 119.1, 116.5, 101.8, 78.5, 47.9.

HRMS (ESI): $C_{10}H_{10}ClN_2O^+$ (M+H)$^+$: 209.0482, found: 209.0477.

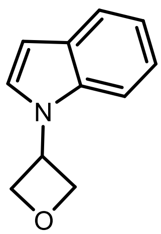

1-(oxetan-3-yl)-1*H*-indole (22)

The title compound was prepared according to the ***General Procedure I*** *(BTMG as base)* and isolated by flash chromatography (Hexane: EtOAc = 30:1) as a yellow liquid (22 mg, 62% yield).

^{1}H NMR (400 MHz, CDCl$_3$) δ 7.70 – 7.65 (m, 1H), 7.50 – 7.46 (m, 1H), 7.46 – 7.42 (m, 1H), 7.29 – 7.22 (m, 1H), 7.20 – 7.13 (m, 1H), 6.64 – 6.61 (m, 1H), 5.63 – 5.55 (m, 1H), 5.18 (dd, *J* = 7.4, 7.4 Hz, 2H), 5.11 (dd, *J* = 6.7, 6.7 Hz, 2H).

^{13}C NMR (101 MHz, CDCl$_3$) δ 135.6, 129.0, 124.9, 122.1, 121.4, 120.1, 109.4, 102.9, 78.1, 50.6.

Matching reported literature data.[6]

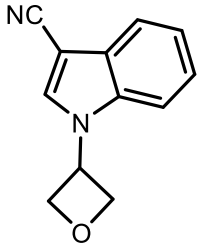

1-(oxetan-3-yl)-1*H*-indole-3-carbonitrile (23)

The title compound was prepared according to the ***General Procedure I*** *(BTMG as base)* and isolated by flash chromatography (Hexane: EtOAc = 5:1) as a yellow liquid (27 mg, 67% yield).

^{1}H NMR (400 MHz, $CDCl_3$) δ 7.89 (s, 1H), 7.80 – 7.76 (m, 1H), 7.61 – 7.55 (m, 1H), 7.41 – 7.31 (m, 2H), 5.62 – 5.54 (m, 1H), 5.24 – 5.39 (m, 2H), 5.06 – 5.01 (m, 2H).

^{13}C NMR (101 MHz, $CDCl_3$) δ 134.8, 132.3, 128.1, 124.4, 122.8, 120.4, 115.4, 110.6, 87.4, 77.0, 51.7.

HRMS (ESI): $C_{12}H_{11}N_2O^+$ $(M+H)^+$: 199.0871, found: 199.0865.

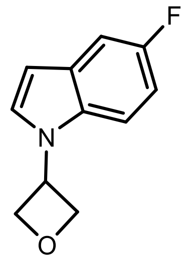

5-fluoro-1-(oxetan-3-yl)-1*H*-indole (24)

The title compound was prepared according to the ***General Procedure I*** *(BTMG as base)* and isolated by flash chromatography (Hexane: EtOAc = 25:1) as a yellow liquid (25 mg, 65% yield).

^{1}H NMR (400 MHz, $CDCl_3$) δ 7.46 (d, *J* = 3.3 Hz, 1H), 7.40 (dd, *J* = 9.0, 4.2 Hz, 1H), 7.30 (dd, *J* = 9.4, 2.5 Hz, 1H), 7.03 – 6.95 (m, 1H), 6.57 (d, *J* = 4.0 Hz, 1H), 5.58 – 5.48 (m, 1H), 5.17 (dd, *J* = 7.4, 7.4 Hz, 2H), 5.07 (dd, *J* = 6.7, 6.7 Hz, 2H).

^{13}C NMR (101 MHz, $CDCl_3$) δ 158.1 (d, *J* = 235.1 Hz), 132.2, 126.6, 129.3 (d, *J* = 10.2 Hz), 110.5 (d, *J* = 26.4 Hz), 110.1 (d, *J* = 9.6 Hz), 106.1 (d, *J* = 23.4 Hz), 102.8 (d, *J* = 4.6 Hz), 77.9, 50.9.

^{19}F NMR (376 MHz, $CDCl_3$) δ -124.4 – -124.5 (m, 1F)

HRMS (ESI): $C_{11}H_{11}FNO^+$ $(M+H)^+$: 192.0825, found: 192.0834.

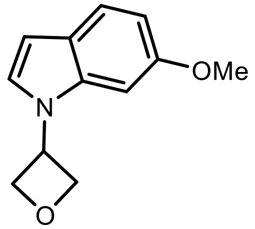

6-methoxy-1-(oxetan-3-yl)-1*H*-indole (25)

The title compound was prepared according to the ***General Procedure I*** *(BTMG as base)* and isolated by flash chromatography (Hexane: EtOAc = 6:1) as a yellow liquid (21 mg, 51% yield).

^{1}H NMR (400 MHz, $CDCl_3$) δ 7.52 (d, *J* = 8.6 Hz, 1H), 7.33 (d, *J* = 3.3 Hz, 1H), 6.94 (d, *J* = 2.2 Hz, 1H), 6.83 (dd, *J* = 8.6, 2.2 Hz, 1H), 6.54 (d, *J* = 2.5 Hz, 1H), 5.56 – 5.48 (m, 1H), 5.16 (dd, *J* = 7.3, 7.3 Hz, 2H), 5.09 (dd, *J* = 6.7, 6.7 Hz, 2H), 3.88 (s, 3H).

^{13}C NMR (101 MHz, $CDCl_3$) δ 156.5, 136.3, 123.7, 123.1, 121.9, 109.9, 102.8, 93.3, 78.0, 55.8, 50.5.

HRMS (ESI): $C_{12}H_{14}NO_2^+$ $(M+H)^+$: 204.1025, found: 204.1016.

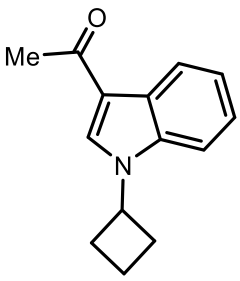

1-(1-cyclobutyl-1*H*-indol-3-yl)ethan-1-one (26)

The title compound was prepared according to the ***General Procedure I*** *(Cs_2CO_3 as base)* and isolated by flash chromatography (Hexane: EtOAc = 50:1) as a yellow solid (21 mg, 50% yield).

^{1}H NMR (400 MHz, $CDCl_3$) δ 8.39 – 8.33 (m, 1H), 7.87 (s, 1H), 7.38 – 7.32 (m, 1H), 7.31 – 7.26 (m, 2H), 4.92 – 4.81 (m, 1H), 2.70 – 2.60 (m, 2H), 2.55 (s, 3H), 2.53 – 2.42 (m, 2H), 2.05 – 1.96 (m, 2H).

^{13}C NMR (101 MHz, $CDCl_3$) δ 193.0, 136.5, 131.7, 126.5, 123.1, 122.6, 122.6, 117.1, 110.2, 50.7, 30.1, 27.7, 15.3.

HRMS (ESI): $C_{14}H_{16}NO^+$ $(M+H)^+$: 214.1232, found: 214.1236.

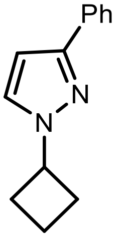

1- cyclobutyl-3-phenyl-1*H*-pyrazole (27)

The title compound was prepared according to the ***General Procedure I*** *(Cs_2CO_3 as base)* and isolated by flash chromatography (Hexane: EtOAc = 75:1) as a yellow liquid (25 mg, 63% yield).

^{1}H NMR (400 MHz, $CDCl_3$) δ 7.84 – 7.80 (m, 2H), 7.49 – 7.46 (m, 1H), 7.42 – 7.36 (m, 2H), 7.31 – 7.26 (m, 1H), 6.55 (d, *J* = 2.3 Hz, 1H), 4.86 – 4.76 (m, 1H), 2.63 – 2.47 (m, 4H), 1.94 – 1.80 (m, 2H).

^{13}C NMR (101 MHz, $CDCl_3$) δ 151.2, 133.8, 128.6, 128.5, 127.4, 125.7, 102.5, 55.8, 30.7, 14.7.

HRMS (ESI): $C_{13}H_{15}N_2^+$ $(M+H)^+$: 199.1235, found: 199.1235.

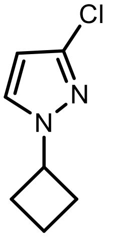

2- chloro-1-cyclobutyl-1*H*-pyrazole (28)

The title compound was prepared according to the ***General Procedure I*** *(Cs_2CO_3 as base)* and isolated by flash chromatography (Hexane: EtOAc = 100:1) as a yellow liquid (20 mg, 64% yield).

^{1}H NMR (400 MHz, CDCl$_3$) δ 7.35 – 7.32 (m, 1H), 6.14 – 6.11 (m, 1H), 4.69 – 4.61 (m, 1H), 2.57 – 2.48 (m, 2H), 2.46 – 2.39 (m, 2H), 1.90 – 1.76 (m, 2H).

^{13}C NMR (101 MHz, CDCl$_3$) δ 138.9, 129.4, 104.5, 56.2, 30.3, 14.5.

HRMS (ESI): $C_7H_{10}ClN_2^+$ $(M+H)^+$: 157.0533, found: 157.0527.

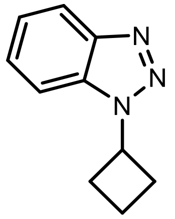

1- cyclobutyl-1*H*-benzo[*d*][1,2,3]triazole (29)

The title compound was prepared according to the ***General Procedure I*** *(Cs_2CO_3 as base)* and isolated by flash chromatography (Hexane: EtOAc = 15:1) as a yellow liquid (16 mg, 46% yield).

^{1}H NMR (400 MHz, CDCl$_3$) δ 8.09 – 8.02 (m, 1H), 7.58 – 7.51 (m, 1H), 7.49 – 7.41 (m, 1H), 7.39 – 7.32 (m, 1H), 5.30 – 5.20 (m, 1H), 3.00 – 2.88 (m, 2H), 2.72 – 2.62 (m, 2H), 2.12 – 2.00 (m, 2H).

^{13}C NMR (101 MHz, CDCl$_3$) δ 146.2, 132.4, 127.0, 123.8, 120.1, 109.6, 52.6, 29.8, 15.5.

Matching reported literature data.[7]

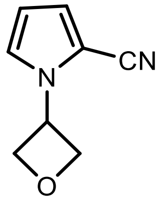

1-(oxetan-3-yl)-1*H*-pyrrole-2-carbonitrile (30)

The title compound was prepared according to the ***General Procedure I*** *(BTMG as base)* and isolated by flash chromatography (Hexane: EtOAc = 10:1) as a white solid (19 mg, 64% yield).

^{1}H NMR (400 MHz, CDCl$_3$) δ 7.35 (dd, J = 2.9, 1.6 Hz, 1H), 6.82 (dd, J = 4.0, 1.6 Hz, 1H), 6.33 (dd, J = 4.0, 2.8 Hz, 1H), 5.52 – 5.44 (m, 1H), 5.13 (dd, J = 7.6, 7.6 Hz, 2H), 4.87 (dd, J = 6.6, 6.6 Hz, 2H).

^{13}C NMR (101 MHz, CDCl$_3$) δ 123.2, 120.5, 113.2, 111.1, 104.0, 78.2, 52.2.

HRMS (ESI): $C_8H_9N_2O^+$ $(M+H)^+$: 149.0715, found: 149.0710.

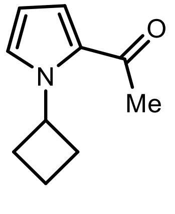

1-(1-cyclobutyl-1*H*-pyrrol-2-yl)ethan-1-one (31)

The title compound was prepared according to the ***General Procedure I*** *(Cs_2CO_3 as base)* and isolated by flash chromatography (Hexane: EtOAc = 100:1) as a yellow liquid (17 mg, 55% yield).

^{1}H NMR (400 MHz, $CDCl_3$) δ 7.17 – 7.13 (m, 1H), 6.96 (dd, *J* = 4.0, 1.7 Hz, 1H), 6.16 (dd, *J* = 4.0, 2.6 Hz, 1H), 5.51 – 5.41 (m, 1H), 2.54 – 2.46 (m, 2H), 2.41 (s, 3H), 2.25 – 2.15 (m, 2H), 1.84 – 1.76 (m, 2H).

^{13}C NMR (101 MHz, $CDCl_3$) δ 188.1, 130.5, 125.9, 120.4, 108.1, 52.4, 31.0, 27.5, 14.5.

HRMS (ESI): $C_{10}H_{14}NO^+$ $(M+H)^+$: 164.1075, found: 164.1074.

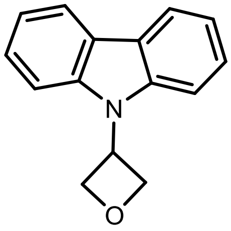

9-(oxetan-3-yl)-9*H*-carbazole (32)

The title compound was prepared according to the ***General Procedure I*** *(BTMG as base)* and isolated by flash chromatography (Hexane: EtOAc = 15:1) as a white solid (25 mg, 56% yield).

^{1}H NMR (400 MHz, $CDCl_3$) δ 8.16 – 8.11 (m, 2H), 7.75 (d, *J* = 8.3 Hz, 2H), 7.54 – 7.47 (m, 2H), 7.33 – 7.27 (m, 2H), 5.92 – 5.83 (m, 1H), 5.50 (dd, *J* = 7.7, 7.7 Hz, 2H), 5.27 (dd, *J* = 6.6, 6.6 Hz, 2H).

^{13}C NMR (101 MHz, $CDCl_3$) δ 139.4, 126.0, 123.5, 120.6, 119.7, 109.6, 76.2, 49.2.

Matching reported literature data.[8]

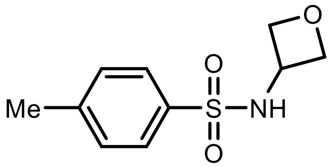

4-methyl-*N*-(oxetan-3-yl)benzenesulfonamide (33)

The title compound was prepared according to the ***General Procedure I*** *(BTMG as base)* and isolated by flash chromatography (Hexane: EtOAc = 1:1) as a white solid (36.5 mg, 80% yield).

NMR (400 MHz, $CDCl_3$) δ 7.74 – 7.69 (m, 2H), 7.33 – 7.27 (m, 2H), 5.81 (d, *J* = 9.1 Hz, 1H), 4.67 (dd, *J* = 7.1, 7.1 Hz, 2H), 4.54 – 4.43 (m, 1H), 4.37 (dd, *J* = 6.7, 6.7 Hz, 2H), 2.42 (s, 3H).

^{13}C NMR (101 MHz, $CDCl_3$) δ 144.1, 137.1, 123.0, 126.9, 78.4, 47.8, 21.6.

HRMS (ESI): $C_{10}H_{14}NO_3S^+$ $(M+H)^+$: 228.0694, found: 228.0688.

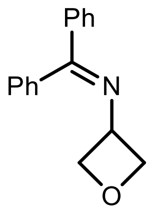

N-(oxetan-3-yl)-1,1-diphenylmethanimine (34)

The title compound was prepared according to the ***General Procedure I*** *(BTMG as base, 12 hours for the reaction)* and isolated by flash chromatography (Hexane: EtOAc = 1:1) as a colorless liquid (28 mg, 58% yield).

^{1}H NMR (400 MHz, CDCl$_3$) δ 7.70 – 7.62 (m, 2H), 7.49 – 7.37 (m, 4H), 7.38 – 7.32 (m, 2H), 7.09 – 7.00 (m, 2H), 4.89 – 4.80 (m, 2H), 4.75 – 4.64 (m, 3H).

^{13}C NMR (101 MHz, CDCl$_3$) δ 169.5, 139.2, 136.8, 130.5, 128.9, 128.7, 128.6, 128.2, 127.5, 79.2, 56.3.

Partially matching reported literature data.[8]

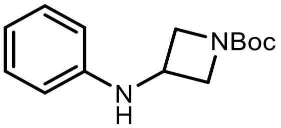

***tert*-butyl 3-(phenylamino)azetidine-1-carboxylate (35)**

The title compound was prepared according to the ***General Procedure I*** *(K_3PO_4 as base)* and isolated by flash chromatography (Hexane: EtOAc = 5:1) as a yellow solid (21 mg, 43% yield).

^{1}H NMR (400 MHz, CDCl$_3$) δ 7.23 – 7.15 (m, 2H), 6.81 – 6.74 (m, 1H), 6.55 – 6.48 (m, 2H), 4.32 – 4.25 (m, 2H), 4.24 – 4.16 (m, 1H), 3.73 (dd, J = 9.1, 4.4 Hz, 2H), 1.45 (s, 9H).

^{13}C NMR (101 MHz, CDCl$_3$) δ 156.2, 146.1, 129.5, 118.6, 113.2, 80.0, 56.4, 43.2, 28.4.

HRMS (ESI): $C_9H_{13}N_2^+$ (M+H - Boc)$^+$: 149.1079, found: 149.1068.

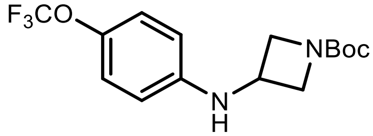

***tert*-butyl 3-((4-(trifluoromethoxy)phenyl)amino)azetidine-1-carboxylate (36)**

The title compound was prepared according to the ***General Procedure I*** *(K_3PO_4 as base)* and isolated by flash chromatography (DCM) as a yellow solid (27 mg, 41% yield).

^{1}H NMR (400 MHz, CDCl$_3$) δ 7.08 – 6.98 (m, 2H), 6.51 – 6.41 (m, 2H), 4.29 (dd, J = 8.9, 7.0 Hz, 2H), 4.21 – 4.13 (m, 1H), 4.09 (br, 1H), 3.72 (dd, J = 9.1, 4.6 Hz, 2H), 1.44 (s, 9H).

^{13}C NMR (101 MHz, CDCl$_3$) δ 156.2, 144.9, 141.4, 122.6, 121.9 (q, J = 255.5 Hz), 113.4, 79.8, 56.6, 43.3, 28.4.

^{19}F NMR (376 MHz, CDCl$_3$) δ -58.5 (s, 3F).

HRMS (ESI): $C_{10}H_{12}F_3N_2O^+$ (M+H - Boc)$^+$: 233.0902, found: 233.0898.

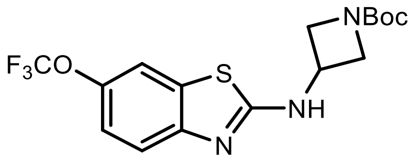

***tert*-butyl 3-((6-(trifluoromethoxy)benzo[*d*]thiazol-2-yl)amino)azetidine-1-carboxylate (37)**

The title compound was prepared according to the ***General Procedure I*** *(K_3PO_4 as base)* and isolated by flash chromatography (Hexane: EtOAc = 2:1) as a white solid (35 mg, 45% yield).

^{1}H NMR (400 MHz, CDCl$_3$) δ 7.51 (d, *J* = 8.8 Hz, 1H), 7.48 – 7.43 (m, 1H), 7.19 – 7.14 (m, 1H), 6.19 (br, 1H), 4.64 – 4.55 (m, 1H), 4.37 (dd, *J* = 9.4, 7.4 Hz, 2H), 3.89 (dd, *J* = 9.4, 4.9 Hz, 2H), 1.45 (s, 9H).

^{13}C NMR (101 MHz, CDCl$_3$) δ 165.9, 156.2, 150.9, 144.0, 131.4, 120.6 (q, *J* = 258.6 Hz), 119.9, 119.7, 114.1, 80.1, 56.6 (br), 43.3, 28.4.

^{19}F NMR (376 MHz, CDCl$_3$) δ -58.2 (s, 3F)

HRMS (ESI): $C_{11}H_{11}F_3N_3OS^+$ (M+H - Boc)$^+$: 290.0575, found: 290.0573.

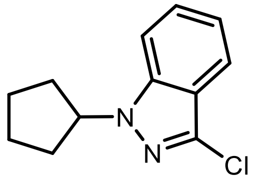

3-chloro-1-cyclopentyl-1*H*-indazole (38)

The title compound was prepared according to the ***General Procedure I*** *(Cs_2CO_3 as base)* and isolated by flash chromatography (Hexane: EtOAc = 150:1) as a yellow liquid (21 mg, 47% yield).

^{1}H NMR (400 MHz, CDCl$_3$) δ 7.68 – 7.62 (m, 1H), 7.44 – 7.36 (m, 2H), 7.21 – 7.15 (m, 1H), 4.98 – 4.88 (m, 1H), 2.19 – 2.12 (m, 4H), 2.03 – 1.92 (m, 2H), 1.78 – 1.68 (m, 2H).

^{13}C NMR (101 MHz, CDCl$_3$) δ 140.6, 132.1, 127.0, 121.2, 121.0, 119.8, 109.6, 59.9, 32.2, 24.5.

Matching reported literature data.[4]

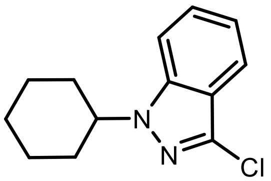

3-chloro-1-cyclohexyl-1*H*-indazole (39)

The title compound was prepared according to the ***General Procedure I*** *(Cs_2CO_3 as base)* and isolated by

flash chromatography (Hexane: EtOAc = 100:1) as a yellow liquid (31 mg, 60% yield).

^{1}H NMR (400 MHz, $CDCl_3$) δ 7.70 – 7.62 (m, 1H), 7.46 – 7.35 (m, 2H), 7.22 – 7.13 (m, 1H), 4.40 – 4.28 (m, 1H), 2.07 – 2.00 (m, 4H), 1.98 – 1.92 (m, 2H), 1.79 – 1.72 (m, 1H), 1.50 – 1.40 (m, 2H), 1.37 – 1.30 (m, 1H).

^{13}C NMR (101 MHz, $CDCl_3$) δ 140.0, 132.2, 127.0, 121.0, 121.0, 119.8, 109.4, 58.5, 32.5, 25.8, 25.3.

Matching reported literature data.[4]

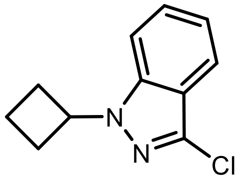

3-chloro-1-cyclobutyl-1*H*-indazole (40)

The title compound was prepared according to the ***General Procedure I*** *(Cs_2CO_3 as base)* and isolated by flash chromatography (Hexane: EtOAc = 100:1) as a yellow solid (32 mg, 78% yield).

^{1}H NMR (400 MHz, $CDCl_3$) δ 7.68 – 7.63 (m, 1H), 7.43 – 7.36 (m, 2H), 7.22 – 7.14 (m, 1H), 5.06 – 4.95 (m, 1H), 2.84 – 2.74 (m, 2H), 2.54 – 2.46 (m, 2H), 2.00 – 1.85 (m, 2H).

^{13}C NMR (101 MHz, $CDCl_3$) δ 140.2, 132.7, 127.2, 121.2, 121.2, 119.8, 109.5, 52.8, 30.0, 14.9.

Matching reported literature data.[8]

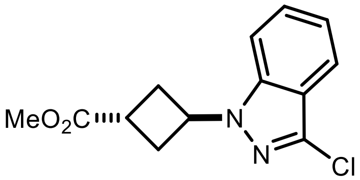

methyl 3-(3-chloro-1*H*-indazol-1-yl)cyclobutane-1-carboxylate (41)

The title compound was prepared according to the ***General Procedure I*** *(Cs_2CO_3 as base)* and isolated by flash chromatography (Hexane: EtOAc = 50:1) as a colorless liquid (32 mg, 58% yield).

^{1}H NMR (400 MHz, $CDCl_3$) δ 7.67 – 7.61 (m, 1H), 7.44 – 7.34 (m, 2H), 7.21 – 7.15 (m, 1H), 5.37 – 5.29 (m, 1H), 3.78 (s, 3H), 3.30 – 3.20 (m, 1H), 3.10 – 3.00 (m, 2H), 2.80 – 2.74 (m, 2H).

^{13}C NMR (101 MHz, $CDCl_3$) δ 176.4, 140.5, 133.4, 127.5, 121.5, 121.2, 119.8, 109.3, 52.1, 50.6, 33.1, 32.4.

Matching reported literature data.[9]

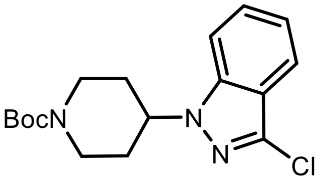

tert-butyl 4-(3-chloro-1_H_-indazol-1-yl)piperidine-1-carboxylate (42)

The title compound was prepared according to the ***General Procedure I*** *(Cs$_2$CO$_3$ as base)* and isolated by flash chromatography (Hexane: EtOAc = 30:1) as a yellow liquid (48 mg, 72% yield).

^{1}H NMR (400 MHz, CDCl$_3$) δ 7.70 – 7.65 (m, 1H), 7.46 – 7.39 (m, 2H), 7.24 – 7.16 (m, 1H), 4.55 – 4.44 (m, 1H), 4.31 (br, 2H), 2.93 (br, 2H), 2.28 – 2.17 (m, 2H), 2.03 – 1.94 (m, 2H), 1.48 (s, 9H).

^{13}C NMR (101 MHz, CDCl$_3$) δ 153.5, 139.0, 131.8, 126.3, 120.3, 120.2, 119.0, 108.1, 78.8, 55.5, 42.2, 30.4, 27.4.

Matching reported literature data.[4]

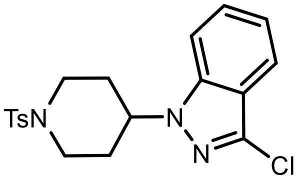

3-chloro-1-(1-tosylpiperidin-4-yl)-1_H_-indazole (43)

The title compound was prepared according to the ***General Procedure I*** *(Cs$_2$CO$_3$ as base)* and isolated by flash chromatography (Hexane: EtOAc = 10:1) as a white solid (44 mg, 57% yield).

^{1}H NMR (400 MHz, CDCl$_3$) δ 7.73 – 7.60 (m, 3H), 7.41 – 7.32 (m, 3H), 7.32 – 7.28 (m, 1H), 7.21 – 7.14 (m, 1H), 4.35 – 4.25 (m, 1H), 3.98 – 3.88 (m, 2H), 2.60 – 2.52 (m, 2H), 2.46 (s, 3H), 2.42 – 2.32 (m, 2H), 2.10 – 2.00 (m, 2H).

^{13}C NMR (101 MHz, CDCl$_3$) δ 143.8, 140.0, 133.2, 133.0, 129.8, 127.8, 127.5, 121.4, 121.3, 120.1, 109.1, 55.6, 45.5, 30.7, 21.6.

Matching reported literature data.[4]

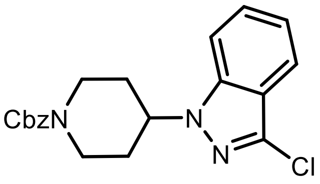

benzyl 4-(3-chloro-1_H_-indazol-1-yl)piperidine-1-carboxylate (44)

The title compound was prepared according to the ***General Procedure I*** *(Cs$_2$CO$_3$ as base)* and isolated by flash chromatography (Hexane: EtOAc = 10:1) as a yellow liquid (37 mg, 50% yield).

^{1}H NMR (400 MHz, CDCl$_3$) δ 7.70 – 7.65 (m, 1H), 7.45 – 7.30 (m, 7H), 7.24 – 7.18 (m, 1H), 5.17 (s, 2H), 4.59 – 4.49 (m, 1H), 4.39 (br, 2H), 3.03 (br, 2H), 2.32 – 2.20 (m, 2H), 2.07 – 1.95 (m, 2H).

^{13}C NMR (101 MHz, CDCl$_3$) δ 155.2, 140.1, 136.7, 133.0, 128.6, 128.1, 128.0, 127.4, 121.4, 121.2, 120.0, 109.1, 67.4, 56.3, 43.3, 31.4.

Matching reported literature data.[10]

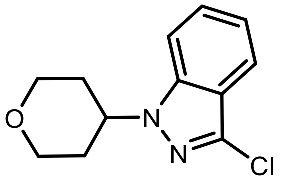

3-chloro-1-(tetrahydro-2*H*-pyran-4-yl)-1*H*-indazole (45)

The title compound was prepared according to the ***General Procedure I*** *(Cs_2CO_3 as base)* and isolated by flash chromatography (Hexane: EtOAc = 30:1) as a white solid (33 mg, 71% yield).

^{1}H NMR (400 MHz, $CDCl_3$) δ 7.70 – 7.65 (m, 1H), 7.46 – 7.39 (m, 2H), 7.23 – 7.17 (m, 1H), 4.64 – 4.54 (m, 1H), 4.20 – 4.12 (m, 2H), 3.64 – 3.55 (m, 2H), 2.46 – 2.34 (m, 2H), 2.00 – 1.93 (m, 2H).

^{13}C NMR (101 MHz, $CDCl_3$) δ 140.0, 132.8, 127.3, 121.3, 121.3, 120.0, 109.2, 67.2, 55.7, 32.4.

Matching reported literature data.[11]

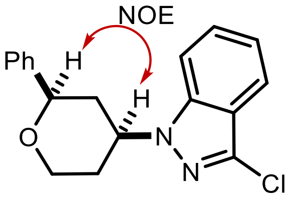

3-chloro-1-((2*R*,4*S*)-2-phenyltetrahydro-2*H*-pyran-4-yl)-1*H*-indazole (46)

The title compound was prepared according to the ***General Procedure I*** *(Cs_2CO_3 as base)* and isolated by flash chromatography (Hexane: EtOAc = 30:1) as a yellow liquid (29 mg, 46% yield).

^{1}H NMR (400 MHz, $CDCl_3$) δ 7.71 – 7.66 (m, 1H), 7.51 – 7.43 (m, 2H), 7.42 – 7.38 (m, 2H), 7.37 – 7.31 (m, 2H), 7.30 – 7.26 (m, 1H), 7.24 – 7.18 (m, 1H), 4.84 – 4.73 (m, 1H), 4.56 (dd, *J* = 11.4, 2.2 Hz, 1H), 4.36 (ddd, *J* = 11.9, 4.8, 1.7 Hz, 1H), 3.87 – 3.77 (m, 1H), 2.56 – 2.36 (m, 2H), 2.27 – 2.20 (m, 1H), 2.08 – 2.01 (m, 1H).

^{13}C NMR (101 MHz, $CDCl_3$) δ 141.5, 140.1, 133.0, 128.5, 127.9, 127.4, 126.0, 121.4, 121.3, 120.1, 109.2, 79.3, 67.2, 56.2, 39.6, 31.9.

HRMS (ESI): $C_{18}H_{18}ClN_2O^+$ $(M+H)^+$: 313.1108, found: 313.1103.

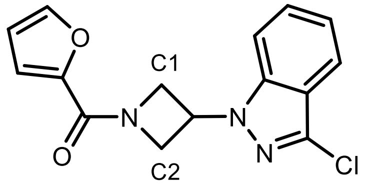

(3-(3-chloro-1*H*-indazol-1-yl)azetidin-1-yl)(furan-2-yl)methanone (47)

The title compound was prepared according to the ***General Procedure I*** *(Cs_2CO_3 as base)* and isolated by flash chromatography (Hexane: EtOAc = 1:1) as a yellow solid (30 mg, 50% yield).

^{1}H NMR (400 MHz, $CDCl_3$) δ 7.73 – 7.68 (m, 1H), 7.52 – 7.40 (m, 3H), 7.29 – 7.22 (m, 1H), 7.13 (d, *J* = 3.5 Hz, 1H), 6.50 (dd, *J* = 3.5, 1.8 Hz, 1H), 5.54 – 5.44 (m, 1H), 5.18 – 4.98 (m, 2H), 4.78 – 4.60 (m, 2H).

^{13}C NMR spectrum was complicated due to the presence of rotamers.

^{13}C NMR (101 MHz, $CDCl_3$) δ 158.8, 147.9, 144.7, 140.8, 134.6, 128.1, 122.0, 121.8, 120.3, 115.9, 111.7, 109.0, 58.9, 55.5, 48.2. *C1 and C2 were observed simultaneously.*

HRMS (ESI): $C_{15}H_{13}ClN_3O_2^+$ $(M+H)^+$: 302.0696, found: 302.0687.

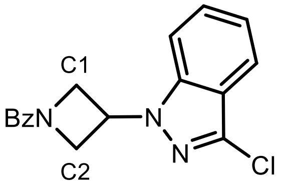

(3-(3-chloro-1*H*-indazol-1-yl)azetidin-1-yl)(phenyl)methanone (48)

The title compound was prepared according to the ***General Procedure I*** *(Cs_2CO_3 as base)* and isolated by flash chromatography (Hexane: EtOAc = 5:1) as a colorless liquid (32 mg, 54% yield).

^{1}H NMR (400 MHz, $CDCl_3$) δ 7.73 – 7.68 (m, 3H), 7.52 – 7.38 (m, 5H), 7.29 – 7.23 (m, 1H), 5.50 – 5.42 (m, 1H), 5.05 – 4.87 (m, 1H), 4,84 – 4.62 (m, 3H).

^{13}C NMR spectrum was complicated due to the presence of rotamers.

^{13}C NMR (101 MHz, $CDCl_3$) δ 169.4, 139.8, 133.5 131.8, 130.3, 127.5, 127.1, 126.9, 121.0, 120.7, 119.2, 107.9, 58.6, 54.7, 46.7. (*C1 and C2 were observed simultaneously*.)

HRMS (ESI): $C_{17}H_{15}ClN_3O^+$ $(M+H)^+$: 312.0904, found: 312.0894.

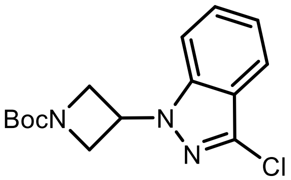

***tert*-butyl 3-(3-chloro-1*H*-indazol-1-yl)azetidine-1-carboxylate (49)**

The title compound was prepared according to the ***General Procedure I*** *(Cs_2CO_3 as base)* and isolated by flash chromatography (Hexane: EtOAc = 30:1) as a yellow liquid (37 mg, 60% yield).

^{1}H NMR (400 MHz, $CDCl_3$) δ 7.72 – 7.65 (m, 1H), 7.49 – 7.39 (m, 2H), 7.26 – 7.20 (m, 1H), 5.37 – 5.27 (m, 1H), 4.56 – 4.46 (m, 2H), 4.42 (dd, *J* = 8.6, 8.6 Hz, 2H), 1.48 (s, 9H).

^{13}C NMR (101 MHz, $CDCl_3$) δ 156.1, 140.7, 134.2, 128.0, 121.9, 121.7, 120.2, 109.1, 80.1, 56.0, 47.5, 28.4.

Matching reported literature data.[4]

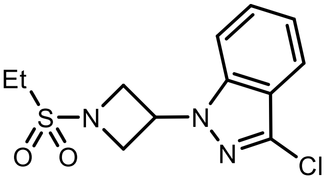

3-chloro-1-(1-(ethylsulfonyl)azetidin-3-yl)-1*H*-indazole (50)

The title compound was prepared according to the ***General Procedure I*** *(Cs_2CO_3 as base)* and isolated by flash chromatography (Hexane: EtOAc = 5:1) as a colorless liquid (39 mg, 65% yield).

^{1}H NMR (400 MHz, $CDCl_3$) δ 7.72 – 7.67 (m, 1H), 7.51 – 7.43 (m, 2H), 7.28 – 7.23 (m, 1H), 5.46 – 5.37 (m, 1H), 4.66 – 4.60 (m, 2H), 4.46 – 4.40 (m, 2H), 3.14 (q, J = 7.4 Hz, 2H), 1.46 (t, J = 7.4 Hz, 3H).

^{13}C NMR (101 MHz, $CDCl_3$) δ 140.7, 134.7, 128.2, 122.1, 121.9, 120.3, 109.1, 56.7, 46.8, 45.5, 7.9.

HRMS (ESI): $C_{12}H_{15}ClN_3O_2S$+ $(M+H)^+$: 300.0574, found: 300.0572.

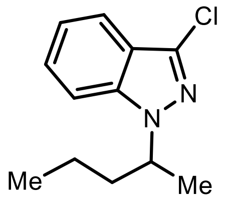

3-chloro-1-(pentan-2-yl)-1*H*-indazole (51)

The title compound was prepared according to the ***General Procedure I*** *(Cs_2CO_3 as base)* and isolated by flash chromatography (Hexane: EtOAc = 150:1) as a yellow liquid (18 mg, 40% yield).

^{1}H NMR (400 MHz, $CDCl_3$) δ 7.68 – 7.63 (m, 1H), 7.43 – 7.36 (m, 2H), 7.21 – 7.14 (m, 1H), 4.64 – 4.55 (m, 1H), 2.13 – 2.03 (m, 1H), 1.84 – 1.74 (m, 1H), 1.55 (d, J = 6.7 Hz, 3H), 1.25 – 1.25 –1.17 (m, 1H), 1.15 –1.07 (m, 1H), 0.87 (t, J = 7.3 Hz, 3H).

^{13}C NMR (101 MHz, $CDCl_3$) δ 140.6, 132.4, 127.1, 121.0, 120.8, 119.8, 109.3, 55.0, 38.6, 20.8, 19.7, 13.7.

HRMS (ESI): $C_{12}H_{16}ClN_2^+$ $(M+H)^+$: 223.1002, found: 223.1001.

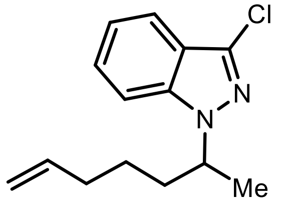

3-chloro-1-(hept-6-en-2-yl)-1*H*-indazole (52)

The title compound was prepared according to the ***General Procedure I*** *(Cs_2CO_3 as base, HCO_2Cs as reductant)* and isolated by flash chromatography (Hexane: EtOAc = 150:1) as a colorless liquid (21.4 mg, 43% yield).

^{1}H NMR (400 MHz, $CDCl_3$) δ 7.70 – 7.64 (m, 1H), 7.43 – 7.36 (m, 2H), 7.22 – 7.14 (m, 1H), 5.77 – 5.64 (m, 1H), 4.99 – 4.85 (m, 2H), 4.64 – 4.53 (m, 1H), 2.16 – 2.06 (m, 1H), 2.04 – 1.98 (m, 2H), 1.89 – 1.79 1.17 (m, 1H), 1.55 (d, J = 6.7 Hz, 3H), 1.37 –1.25 (m, 1H), 1.24 –1.13 (m, 1H).

^{13}C NMR (101 MHz, $CDCl_3$) δ 140.6, 138.3, 132.5, 127.1, 121.0, 120.9, 119.8, 114.9, 109.3, 55.2, 35.8, 33.3, 25.7, 20.8.

HRMS (ESI): $C_{14}H_{18}ClN_2^+$ $(M+H)^+$: 249.1153, found: 249.1152.

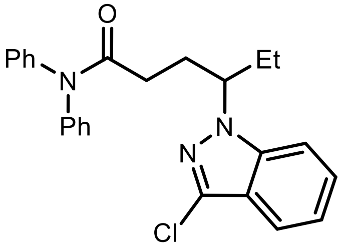

4-(3-chloro-1*H*-indazol-1-yl)-*N,N*-diphenylhexanamide (53)

The title compound was prepared according to the ***General Procedure I*** *(Cs_2CO_3 as base, HCO_2Cs as reductant)* and isolated by flash chromatography (Hexane: EtOAc = 20:1) as a colorless liquid (39 mg, 47% yield).

^{1}H NMR (400 MHz, $CDCl_3$) δ 7.72 – 7.66 (m, 1H), 7.48 – 7.40 (m, 2H), 7.35 – 7.00 (m, 9H), 6.80 – 6.35 (m, 2H), 4.71 – 4.61 (m, 1H), 2.39 – 2.31 (m, 1H), 2.22 – 2.13 (m, 1H), 2.10 – 2.00 (m, 2H), 1.95 – 1.85 (m, 1H), 1.71 – 1.62 (m, 1H), 0.75 (t, J = 7.4 Hz, 3H).

^{13}C NMR (101 MHz, $CDCl_3$) δ 172.1, 142.3, 142.2, 133.1, 129.4, 128.5, 127.4, 126.3, 121.2, 120.5, 119.7, 109.7, 59.73, 31.0, 30.2, 28.4, 11.0.

HRMS (ESI): $C_{25}H_{25}ClN_3O+^+$ $(M+H)^+$: 418.1681, found: 418.1680.

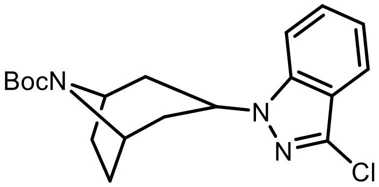

***tert*-butyl 3-(3-chloro-1*H*-indazol-1-yl)-8-azabicyclo[3.2.1]octane-8-carboxylate (54)**

The title compound was prepared according to the ***General Procedure I*** *(Cs_2CO_3 as base)* and isolated by flash chromatography (DCM as eluent) as a white solid (45 mg, 63% yield).

^{1}H NMR (400 MHz, $CDCl_3$) δ 7.69 – 7.63 (m, 1H), 7.41 – 7.35 (m, 2H), 7.21 – 7.15 (m, 1H), 5.00 – 4.88 (m, 1H), 4.52 – 4.30 (m, 2H), 2.53 – 2.38 (m, 2H), 2.15 – 2.06 (m, 2H), 1.95 – 1.89 (m, 2H), 1.86 – 1.80 (m, 2H), 1.53 (s, 9H).

^{13}C NMR spectrum was complicated due to the presence of rotamers.

^{13}C NMR (101 MHz, $CDCl_3$) δ 153.4, 139.7, 132.5, 127.2, 121.6, 121.3, 120.1, 109.6, 79.7, 53.3, 52.4, 36.6, 36.1, 28.5, 27.7.

Matching reported literature data.[11]

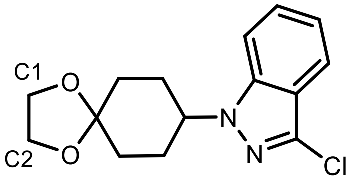

3-chloro-1-(1,4-dioxaspiro[4.5]decan-8-yl)-1*H*-indazole (55)

The title compound was prepared according to the ***General Procedure I*** *(Cs_2CO_3 as base)* and isolated by

flash chromatography (Hexane: EtOAc = 30:1) as a yellow solid (32 mg, 55% yield).

^{1}H NMR (400 MHz, $CDCl_3$) δ 7.68 – 7.63 (m, 1H), 7.47 – 7.37 (m, 2H), 7.21 – 7.14 (m, 1H), 4.51 – 4.38 (m, 1H), 3.99 (s, 4H), 2.47 – 2.34 (m, 2H), 2.05 – 1.92 (m, 4H), 1.82 – 1.72 (m, 2H).

^{13}C NMR (101 MHz, $CDCl_3$) δ 140.1, 132.3, 127.1, 121.2, 121.1, 119.9, 109.5, 107.6, 64.5, 64.4, 57.3, 33.7, 29.3. *C1 and C2 were observed simultaneously in the carbon spectrum.*

Matching reported literature data.[4]

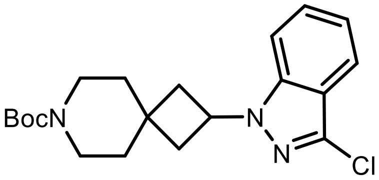

***tert*-butyl 2-(3-chloro-1*H*-indazol-1-yl)-7-azaspiro[3.5]nonane-7-carboxylate (56)**

The title compound was prepared according to the ***General Procedure I*** *(Cs_2CO_3 as base)* and isolated by flash chromatography (DCM as eluent) as a colorless liquid (43 mg, 57% yield).

^{1}H NMR (400 MHz, $CDCl_3$) δ 7.67 – 7.62 (m, 1H), 7.42 – 7.33 (m, 2H), 7.21 – 7.14 (m, 1H), 5.08 – 4.94 (m, 1H), 3.46 – 3.39 (m, 2H), 3.37 – 3.30 (m, 2H), 2.57 – 2.49 (m, 2H), 2.48 – 2.40 (m, 2H), 1.75 – 1.65 (m, 4H), 1.46 (s, 9H).

^{13}C NMR (101 MHz, $CDCl_3$) δ 155.0, 140.4, 132.8, 127.3, 121.3, 121.3, 119.8, 109.4, 79.4, 48.2, 40.8, 38.9, 36.0, 32.3, 28.5.

Matching reported literature data.[8]

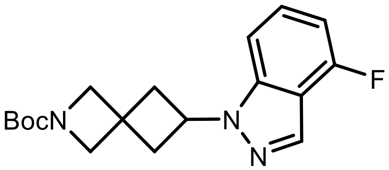

***tert*-butyl 6-(4-fluoro-1*H*-indazol-1-yl)-2-azaspiro[3.3]heptane-2-carboxylate (57)**

The title compound was prepared according to the ***General Procedure I*** *(Cs_2CO_3 as base)* and isolated by flash chromatography (Hexane: EtOAc = 30:1) as a white solid (46 mg, 69% yield).

^{1}H NMR (400 MHz, $CDCl_3$) δ 8.07 (s, 1H), 7.31 – 7.24 (m, 1H), 7.14 (d, J = 8.4 Hz, 1H), 6.76 (dd, J = 9.9, 7.6 Hz, 1H), 4.98 – 4.87 (m, 1H), 4.09 (s, 2H), 4.00 (s, 2H), 2.98 – 2.80 (m, 2H), 2.82 – 2.73 (m, 2H), 1.45 (s, 9H).

^{13}C NMR (101 MHz, $CDCl_3$) δ 156.2, 155.8 (d, J = 253.5 Hz), 141.7 (d, J = 9.1 Hz), 129.8 (d, J = 1.9 Hz), 127.2 (d, J = 7.6 Hz), 114.54 (d, J = 23.2 Hz), 105.04 (d, J = 14.2 Hz), 104.9, 79.5, 60.1, 47.7, 40.4, 31.9, 28.4.

^{19}F NMR (376 MHz, $CDCl_3$) δ -117.76 (dd, J = 10.1, 5.1 Hz, 1F)

HRMS (ESI): $C_{18}H_{23}FN_3O_2^+$ $(M+H)^+$: 332.1774, found: 332.1768.

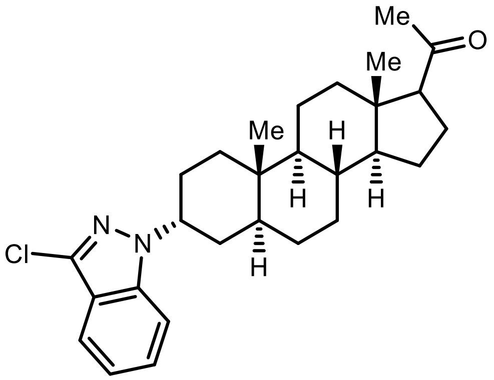

1-((3*R*,5*S*,8*R*,9*S*,10*S*,13*S*,14*S*)-3-(3-chloro-1*H*-indazol-1-yl)-10,13-dimethylhexadecahydro-1*H*-cyclopenta[*a*]phenanthren-17-yl)ethan-1-one (58)

The title compound was prepared according to the ***General Procedure I*** *(Cs_2CO_3 as base)* and isolated by flash chromatography (Hexane: EtOAc = 25:1) as a white solid (39 mg, 43% yield, d.r. = 5:1).

^{1}H NMR (400 MHz, CDCl$_3$) δ 7.70 – 7.60 (m, 1.2H), 7.43 – 7.35 (m, 2.4H), 7.20 – 7.13 (m, 1.2H), 4.45 – 4.30 (m, 1.2H), 2.83 – 2.78 (m, 0.2H), 2.53 (t, *J* = 8.8 Hz, 1H), 2.28 – 1.98 (m, 8.4H), 1.92 – 1.84 (m, 2.4H), 1.74 – 1.60 (m, 6H), 1.45 – 1.15 (m, 12H), 0.96 (s, 3H), 0.95 (s, 0.6H), 0.92 (s, 0.6H), 0.84 – 0.74 (m, 1.2H), 0.62 (s, 3H).

^{13}C NMR (101 MHz, CDCl$_3$) δ 212.9&209.6, 140.1&140.0, 132.2&132.1, 127.0&127.0, 121.0&120.9 (two carbon peaks overlapped), 119.8&119.7, 109.4&109.4, 63.9&61.3, 58.8&58.6, 56.7&53.6, 54.3&50.3, 46.1&46.0, 45.8&44.3, 39.1&39.1, 37.8&35.7, 35.8&35.70, 35.5&35.3, 34.5&34.4, 32.9&32.0, 32.2&31.5, 28.5&28.5, 28.0&25.9, 24.4&24.4, 22.8&21.1, 21.2&20.9, 13.5&13.5, 12.6&12.5.

HRMS (ESI): $C_{28}H_{38}ClN_2O^+$ (M+H)$^+$: 453.2673, found: 453.2679.

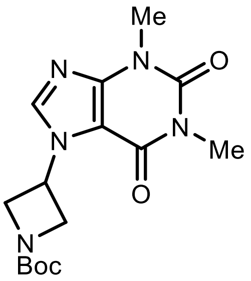

***tert*-butyl 3-(1,3-dimethyl-2,6-dioxo-1,2,3,6-tetrahydro-7*H*-purin-7-yl)azetidine-1-carboxylate (91)**

The title compound was prepared according to the ***General Procedure I*** *(BTMG as base)* and isolated by flash chromatography (EtOAc as eluent) as a white solid (22 mg, 33% yield).

^{1}H NMR (400 MHz, CDCl$_3$) δ 7.80 (s, 1H), 5.50 – 5.42 (m, 1H), 4.51 – 4.42 (m, 2H), 4.22 (dd, *J* = 9.7, 5.4 Hz, 2H), 3.59 (s, 3H), 3.39 (s, 3H), 1.47 (s, 9H).

^{13}C NMR (101 MHz, CDCl$_3$) δ 156.4, 155.1, 151.5, 149.4, 139.0, 107.2, 80.6, 56.2 (br), 46.1, 29.9, 28.3, 28.1.

HRMS (ESI): $C_{10}H_{14}N_5O_2^+$ (M+H - Boc)$^+$: 236.1147, found: 236.1146.

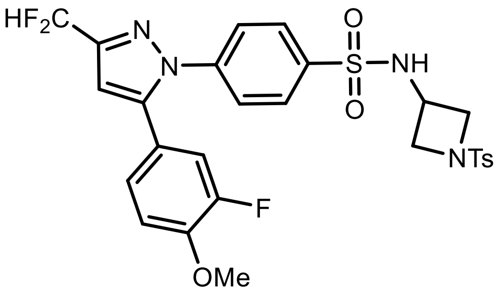

4-(3-(difluoromethyl)-5-(3-fluoro-4-methoxyphenyl)-1*H*-pyrazol-1-yl)-*N*-(1-tosylazetidin-3-yl)benzenesulfonamide (92)

The title compound was prepared according to the ***General Procedure I*** *(BTMG as base)* and isolated by flash chromatography (Hexane: EtOAc = 1:1) as a colorless liquid (81 mg, 67% yield).

^{1}H NMR (400 MHz, CDCl$_3$) δ 7.79 – 7.74 (m, 2H), 7.66 – 7.62 (m, 2H), 7.46 – 7.41 (m, 2H), 7.36 – 7.31 (m, 2H), 7.01 (dd, *J* = 11.5, 2.1 Hz, 1H), 6.96 (dd, *J* = 8.6, 8.6 Hz, 1H), 6.87 – 6.83 (m, 1H), 6.76 (t, *J* = 54.9 Hz, 1H), 6.70 (s, 1H), 5.63 (d, *J* = 9.7 Hz, 1H), 4.04 – 3.94 (m, 1H), 3.90 (s, 3H), 3.83 – 3.77 (m, 2H), 3.54 – 3.48 (m, 2H), 2.43 (s, 3H).

^{13}C NMR (101 MHz, CDCl$_3$) δ 152.1 (d, *J* = 248.3 Hz), 148.7 (d, *J* = 10.5 Hz), 148.5 (t, *J* = 29.8 Hz), 144.8, 143.8, 142.9, 139.2, 131.3, 130.0, 128.2, 128.0, 125.6, 125.2 (d, *J* = 3.7 Hz), 121.5 (d, *J* = 7.1 Hz), 116.6 (d, *J* = 19.7 Hz), 113.7 (d, *J* = 2.4 Hz), 110.9 (t, *J* = 234.8 Hz), 106.0, 57.6, 56.3, 42.1, 21.6.

^{19}F NMR (376 MHz, CDCl$_3$) δ -112.47 (d, *J* = 54.8 Hz, 2F), -133.09 (dd, *J* = 11.5, 8.6 Hz, 1F).

HRMS (ESI): $C_{27}H_{26}F_3N_4O_5S_2$+ (M+H+): 607.1291, found: 607.1305.

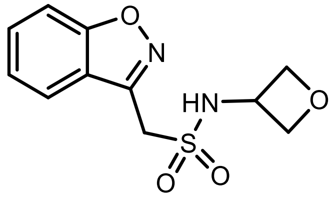

1-(benzo[*d*]isoxazol-3-yl)-*N*-(oxetan-3-yl)methanesulfonamide (93)

The title compound was prepared according to the ***General Procedure I*** *(BTMG as base)* and isolated by flash chromatography (Hexane: EtOAc = 2:1) as a colorless liquid (21 mg, 40% yield).

^{1}H NMR (400 MHz, CDCl$_3$) δ 7.89 – 7.85 (m, 1H), 7.66 – 7.60 (m, 2H), 7.45 – 7.37 (m, 1H), 5.40 (d, *J* = 9.0 Hz, 1H), 4.70 (dd, *J* = 7.2, 7.2 Hz, 2H), 4.65 (s, 2H), 4.50 (dd, *J* = 6.6, 6.6 Hz, 2H). *The* ***NH*** *was not observed.*

^{13}C NMR (101 MHz, CDCl$_3$) δ 163.8, 149.6, 130.8, 124.6, 122.3, 120.6, 110.1, 78.8, 49.9, 48.4.

HRMS (ESI): $C_{11}H_{13}N_2O_4S^+$ (M+H)$^+$: 269.0596, found: 269.0589.

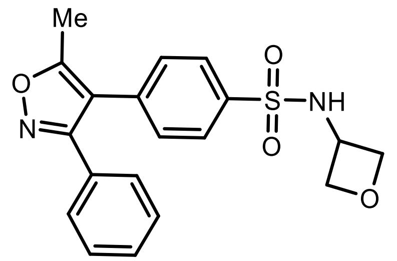

4-(5-methyl-3-phenylisoxazol-4-yl)-*N*-(oxetan-3-yl)benzenesulfonamide (94)

The title compound was prepared according to the ***General Procedure I*** *(BTMG as base)* and isolated by flash chromatography (Hexane: EtOAc = 2:1) as a colorless liquid (48 mg, 65% yield).

^{1}H NMR (400 MHz, CDCl$_3$) δ 7.84 – 7.80 (m, 2H), 7.40 – 7.27 (m, 7H), 6.0 (d, *J* = 9.2 Hz, 1H), 4.69 (dd, J = 7.0, 7.0 Hz, 2H), 4.59 – 4.48 (m, 1H), 4.39 (dd, *J* = 6.5, 6.5 Hz, 2H), 2.47 (s, 3H).

^{13}C NMR (101 MHz, CDCl$_3$) δ 167.4, 161.1, 139.3, 135.7, 130.6, 129.9, 128.8, 128.5, 128.4, 127.3, 114.4, 78.3, 47.9, 11.8.

HRMS (ESI): $C_{19}H_{19}N_2O_4S^+$ $(M+H)^+$: 371.1066, found: 371.1063.

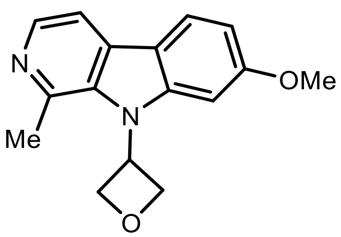

7-methoxy-1-methyl-9-(oxetan-3-yl)-9*H*-pyrido[3,4-*b*]indole (95)

The title compound was prepared according to the ***General Procedure I*** *(BTMG as base)* and isolated by flash chromatography (Hexane: EtOAc = 2:1) as a yellow solid (22 mg, 42% yield).

^{1}H NMR (400 MHz, CDCl$_3$) δ 8.29 (d, *J* = 5.2 Hz, 1H), 8.02 (d, *J* = 8.7 Hz, 1H), 7.84 (d, *J* = 2.1 Hz, 1H), 7.72 (d, *J* = 5.2 Hz, 1H), 6.97 (dd, *J* = 8.7, 2.2 Hz, 1H), 6.43 – 6.34 (m, 1H), 5.48 (dd, *J* = 7.6, 5.8 Hz, 2H), 5.25 (dd, *J* = 7.9, 7.9 Hz, 2H), 3.98 (s, 3H), 2.97 (s, 3H).

^{13}C NMR (101 MHz, CDCl$_3$) δ 161.0, 141.1, 140.0, 138.9, 135.8, 129.6, 122.7, 116.4, 112.3, 109.8, 95.7, 76.3, 55.8, 50.8, 24.7.

HRMS (ESI): $C_{16}H_{17}N_2O_2^+$ $(M+H)^+$: 269.1290, found: 269.1289.

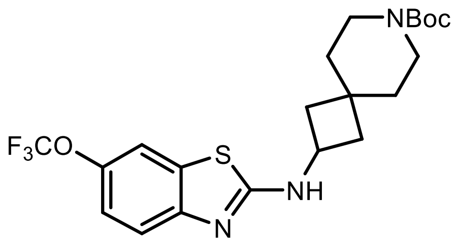

***tert*-butyl 2-((6-(trifluoromethoxy)benzo[*d*]thiazol-2-yl)amino)-7-azaspiro[3.5]nonane-7-carboxylate (96)**

The title compound was prepared according to the ***General Procedure I*** *(K_3PO_4 as base)* and isolated by flash chromatography (Hexane: EtOAc = 2:1) as a white solid (36 mg, 39% yield).

^{1}H NMR (400 MHz, $CDCl_3$) δ 7.49 (d, *J* = 8.8 Hz, 1H), 7.47 – 7.43 (m, 1H), 7.19 – 7.13 (m, 1H), 5.90 (br, 1H), 4.25 – 4.14 (m, 1H), 3.42 – 3.34 (m, 2H), 3.32 – 3.25 (m, 2H), 2.51 – 2.44 (m, 2H), 1.79 – 1.72 (m, 2H), 1.65 – 1.60 (m, 2H), 1.55 – 1.50 (m, 2H), 1.45 (s, 9H).

^{13}C NMR (101 MHz, $CDCl_3$) δ 166.7, 154.9, 150.8, 143.7, 131.0, 120.6 (q, *J* = 256.9 Hz) 119.8, 119.1, 114.1, 79.5, 45.5, 40.3, 39.4 (br), 35.9 (br), 32.5, 28.5.

^{19}F NMR (376 MHz, $CDCl_3$) δ -58.2 (s, 3F)

HRMS (ESI): $C_{21}H_{27}F_3N_3O_3S^+$ (M+H - Boc)$^+$: 458.1720, found: 458.1725.

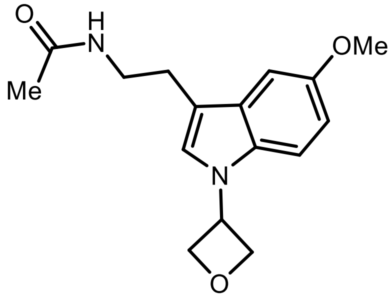

N-(2-(5-methoxy-1-(oxetan-3-yl)-1*H*-indol-3-yl)ethyl)acetamide (97)

The title compound was prepared according to the ***General Procedure I*** *(BTMG as base)* and isolated by flash chromatography (EtOAc as eluent) as a white solid (32 mg, 55% yield).

^{1}H NMR (400 MHz, $CDCl_3$) δ 7.35 (d, *J* = 8.9 Hz, 1H), 7.22 (s, 1H), 7.05 (d, *J* = 2.5 Hz, 1H), 6.90 (dd, *J* = 8.9, 2.5 Hz, 1H), 5.65 (br, 1H), 5.51 – 5.42 (m, 1H), 5.13 (dd, *J* = 7.3, 7.3 Hz, 2H), 5.04 (dd, *J* = 6.6, 6.6 Hz, 2H), 3.86 (s, 3H), 3.62 – 3.55 (m, 2H), 2.95 (t, *J* = 6.8 Hz, 2H), 1.95 (s, 3H).

^{13}C NMR (101 MHz, $CDCl_3$) δ 170.1, 154.3, 131.2, 128.8, 123.4, 112.9, 112.5, 110.4, 101.0, 78.0, 56.0, 50.8, 39.9, 25.4, 23.4.

HRMS (ESI): $C_{16}H_{21}N_2O_3^+$ (M+H)$^+$: 289.1552, found: 289.1559.

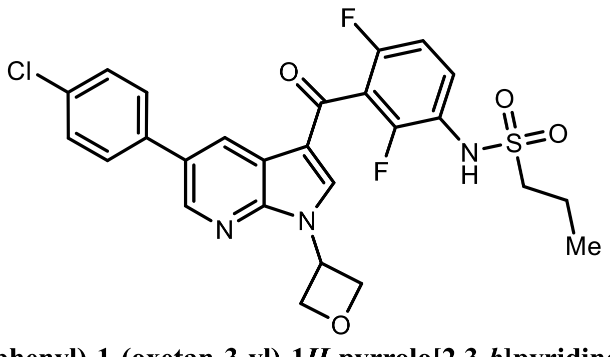

N-(3-(5-(4-chlorophenyl)-1-(oxetan-3-yl)-1*H*-pyrrolo[2,3-*b*]pyridine-3-carbonyl)-2,4-difluorophenyl)propane-1-sulfonamide (98)

The title compound was prepared according to the ***General Procedure I*** *(BTMG as base)* and isolated by

flash chromatography (Hexane: EtOAc = 1:1) as a white solid (40 mg, 38% yield).

^{1}H NMR (400 MHz, CDCl$_3$) δ 8.84 (d, *J* = 2.2 Hz, 1H), 8.62 (d, *J* = 2.3 Hz, 1H), 8.02 (s, 1H), 7.74 – 7.66 (m, 1H), 7.62 – 7.57 (m, 2H), 7.51 – 7.44 (m, 2H), 7.11 – 7.05 (m, 1H), 6.12 – 6.05 (m, 1H), 5.23 (t, *J* = 7.4 Hz, 2H), 5.02 (d, *J* = 6.5 Hz, 2H), 3.16 – 3.10 (m, 2H), 1.96 – 1.85 (m, 2H), 1.06 (t, *J* = 7.4 Hz, 3H).

Note: Not all ^{13}C resonances could be unambiguously assigned due to the extremely low solubility of this compound in CDCl$_3$.

^{13}C NMR (101 MHz, CDCl$_3$) δ 180.5, 147.9, 144.4, 136.8, 134.9, 134.0, 132.6, 129.4, 129.3, 128.8, 125.7 (d, *J* = 9.5 Hz), 121.9 (dd, *J* = 4.0, 14.1 Hz), 122.0, 118.8, 116.9, 112.7 (d, *J* =3.6, 18.6 Hz), 77.60, 54.5, 49.3, 17.3, 12. 9.

^{19}F NMR (376 MHz, CDCl$_3$) δ -115.06 – -115.14 (m, 1F), -126.15 (d, *J* = 8.8 Hz, 1F).

HRMS (ESI): $C_{26}H_{23}ClF_2N_3O_4S^+$ (M+H)$^+$: 546.1066, found: 546.1063.

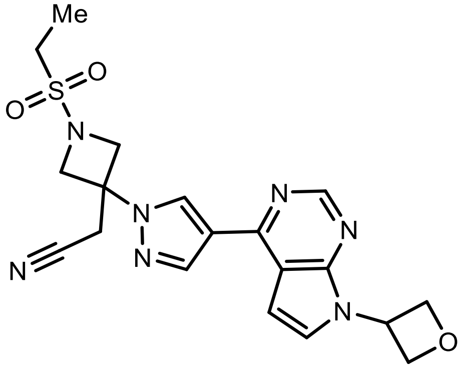

2-(1-(ethylsulfonyl)-3-(4-(7-(oxetan-3-yl)-7*H*-pyrrolo[2,3-*d*]pyrimidin-4-yl)-1*H*-pyrazol-1-yl)azetidin-3-yl)acetonitrile (99)

The title compound was prepared according to the ***General Procedure I*** *(BTMG as base)* and isolated by flash chromatography (EtOAc as eluent) as a white solid (38 mg, 45% yield).

^{1}H NMR (400 MHz, CDCl$_3$) δ 8.81 (s, 1H), 8.45 (s, 1H), 8.34 (s, 1H), 7.75 (d, *J* = 3.8 Hz, 1H), 6.87 (d, *J* = 3.8 Hz, 1H), 6.12 – 6.03 (m, 1H), 5.21 (dd, *J* = 7.4, 7.4 Hz, 2H), 5.02 (dd, *J* = 6.6, 6.6 Hz, 2H), 4.65 – 4.61 (m, 2H), 4.27 – 4.23(m, 2H), 3.41 (s, 2H), 3.08 (q, *J* = 7.4 Hz, 2H), 1.41 (t, *J* = 7.4 Hz, 3H).

^{13}C NMR (101 MHz, CDCl$_3$) δ 150.6 (two carbon peaks overlapped), 149.3, 139.8, 127.1, 124.5, 122.2, 114.0, 113.5, 100.0, 77.1, 57.9, 55.1, 47.3, 45.8, 26.7, 6.9.

HRMS (ESI): $C_{19}H_{22}N_7O_3S^+$ (M+H)$^+$: 428.1505, found: 428.1506.

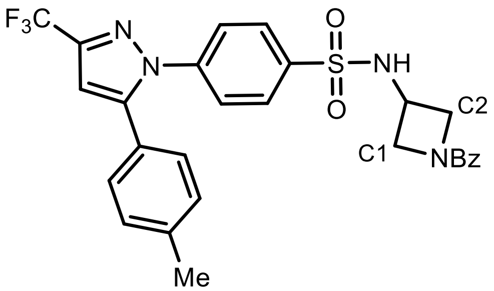

N-(1-benzoylazetidin-3-yl)-4-(5-(p-tolyl)-3-(trifluoromethyl)-1H-pyrazol-1-yl)benzenesulfonamide (100)

The title compound was prepared according to the ***General Procedure I*** *(BTMG as base)* and isolated by flash chromatography (Hexane: EtOAc = 1:1) as a colorless liquid (77 mg, 71% yield).

^{1}H NMR (400 MHz, CDCl$_3$) δ 7.79 – 7.73 (m, 2H), 7.49 – 7.45 (m, 2H), 7.44 – 7.38 (m, 3H), 7.36 – 7.30 (m, 2H), 7.16 – 7.11 (m, 2H), 7.09 – 7.05 (m, 2H), 7.00 (d, J = 8.4 Hz, 1H), 6.73 (s, 1H), 4.31 (br, 2H), 4.21 – 4.11 (m, 1H), 3.98 (d, J = 49.8 Hz, 2H), 2.35 (s, 3H).

^{13}C NMR (101 MHz, CDCl$_3$) δ 170.3, 145.3, 144.1 (q, J = 38.5 Hz), 142.7, 139.9, 139.6, 132.3, 131.6, 129.8 (two carbons overlapped), 128.7, 128.6, 127.9, 127.8, 125.6, 121.1 (q, J = 269.2 Hz), 106.4 (q, J = 2.2 Hz), 60.8, 56.0, 43.2, 21.3. *C1 and C2 were observed simultaneously in the carbon spectrum.*

^{19}F NMR (376 MHz, CDCl$_3$) δ -62.4 (s, 3F).

HRMS (ESI): $C_{27}H_{24}F_3N_4O_3S^+$ $(M+H)^+$: 541.1516, found: 541.1519.

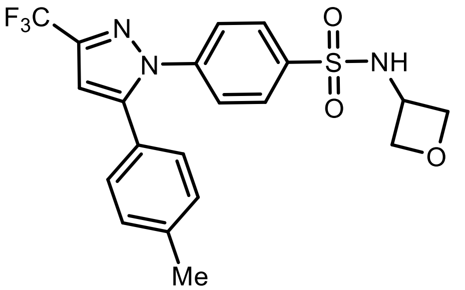

N-(oxetan-3-yl)-4-(5-(p-tolyl)-3-(trifluoromethyl)-1H-pyrazol-1-yl)benzenesulfonamide (101)

The title compound was prepared according to the ***General Procedure I*** *(BTMG as base)* and isolated by flash chromatography (Hexane: EtOAc = 2:1) as a colorless liquid (14.2 g, 65% yield).

^{1}H NMR (400 MHz, CDCl$_3$) δ 7.85 – 7.79 (m, 2H), 7.51 – 7.45 (m, 2H), 7.20 – 7.13 (m, 2H), 7.12 – 7.05 (m, 2H), 6.74 (s, 1H), 5.89 (d, J = 8.8 Hz, 1H), 4.70 (dd, J = 7.0, 7.0 Hz, 2H), 4.55 – 4.45 (m, 1H), 4.37 (dd, J = 6.5, 6.5 Hz, 2H), 2.37 (s, 3H).

^{13}C NMR (101 MHz, CDCl$_3$) δ 145.3, 144.2 (q, J = 38.6 Hz), 142.9, 140.0, 139.5, 129.8, 128.7, 127.9, 125.8, 125.6, 121.0 (q, J = 269.2 Hz), 106.4 (q, J = 2.1 Hz), 78.3, 47.8, 21.3.

^{19}F NMR (376 MHz, CDCl$_3$) δ -62.4 (s, 3F).

HRMS (ESI): $C_{20}H_{19}F_3N_3O_3S^+$ $(M+H)^+$: 438.1099, found: 438.1093.

C3. Copper-Catalyzed Sulfonylation of Alkyl Bromides

C3.1. Optimization Studies

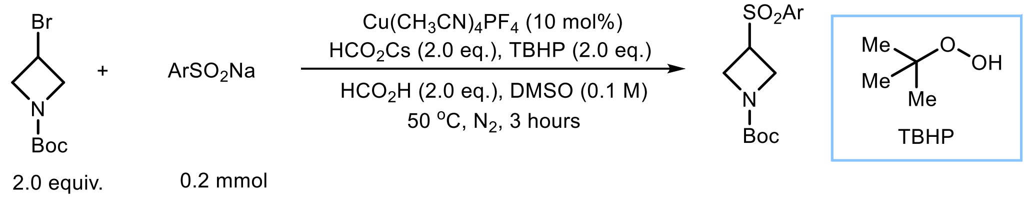

Table S2. Optimization of the model reaction

Entry	Deviations	NMRy (%)
1	none	64(66)[a]
2	No **Cu**	< 5
3	No TBHP, HCO_2Cs	< 5
4	No HCO_2H	14
5	HCO_2Na	28
6	HCO_2K	50
7	$Cu(CH_3CN)_4BF_4$ (10 mol%)	33
8	Cu(OAc)2 (10 mol%)	42
9	CuI (10 mol%)	18
10	CuOAc (10 mol%)	31
11	CuTc (10 mol%)	38
12	$Cu(OTf)_2$ (10 mol%)	30
13	$Cu(acac)_2$ (10 mol%)	8%
14	CH_3CN as solvent	0
15	DMA as solvent	10
16	DMF as solvent	15
17	TFA instead of HCO_2H	30
18	HOAc instead of HCO_2H	25
19	HFIP instead of HCO_2H	< 5
20	$(^tBuO)_2POOH$ instead of HCO_2H	27
21	DTBP, TBPB or $K_2S_2O_8$ as oxidant	< 5

All reactions were performed on a 0.1 mmol scale; yield of product determined by ^1H NMR analysis of the crude reaction mixture by comparison with 1,3,5-trimethoxybenzene as internal standard. [a]Yield of isolated product. TFA: trifluoroacetic acid; HFIP: hexafluoro-2-propanol; DTBP: di-*tert*-butyl peroxide; TBPB: *tert*-butyl peroxybenzoate.

C3.2. General Procedure *II*: Sulfonylation of Alkyl Bromides

In a glove box, to a 4 mL glass vial, $Cu(CH_3CN)_4PF_6$ (7.5 mg, 0.02 mmol, 10 mol%), cesium formate (88 mg, 0.4 mmol, 2.0 equiv.), sodium arylsulfinate (0.2 mmol, 1.0 equiv.) and alkyl bromides (if solid, 0.4 mmol, 2.0 equiv.) were sequentially added, then the vial was sealed with a screw-top cap with septum. Outside the glove box, DMSO (0.1 M, 2.0 mL), alkyl bromides (if liquid, 0.4 mmol, 2.0 equiv.) and formic acid (16 µL, 0.4 mmol, 2.0 equiv.) followed by TBHP (60 µL, 0.4 mmol, 2.0 equiv.) were added *via* syringe. The vial was sealed with Parafilm and then stirred at 50 °C for 3 hours. After the reaction was complete, the reaction mixture was transferred to an extraction funnel, 10 mL of H_2O and 2 mL of brine were added

and the organic layer was extracted with EtOAc. The organic layer was washed with brine twice. The combined organic layers were dried over anhydrous Na_2SO_4, filtered, and concentrated to dryness. The crude residue was purified by column chromatography to afford the corresponding product in the stated yield with >95% purity according to ^{1}H NMR analysis.

C3.3. Characterization of Products

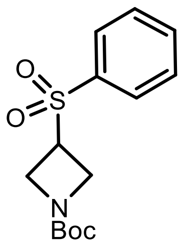

tert-butyl 3-(phenylsulfonyl)azetidine-1-carboxylate (59)

The title compound was prepared according to the ***General Procedure II*** and isolated by flash chromatography (Hexane: EtOAc = 5:1) as a colorless liquid (39 mg, 66% yield).

^{1}H NMR (400 MHz, $CDCl_3$) δ 7.95 – 7.85 (m, 2H), 7.72 – 7.65 (m, 1H), 7.52 – 7.55 (m, 2H), 4.31 – 4.19 (m, 2H), 4.04 (dd, *J* = 8.9, 8.9 Hz, 2H), 4.00 – 3.90 (m, 1H), 1.40 (s, 9H).

^{13}C NMR (101 MHz, $CDCl_3$) δ 155.7, 137.0, 134.4, 129.7, 128.4, 80.5, 51.2, 49.6, 28.3.

HRMS (ESI): $C_9H_{12}NO_2S^+$ $(M+H - Boc)^+$: 198.0589, found: 198.0592.

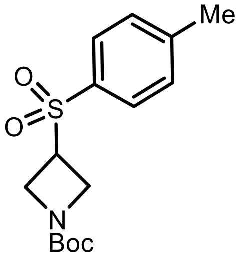

tert-butyl 3-tosylazetidine-1-carboxylate (60)

The title compound was prepared according to the ***General Procedure II*** and isolated by flash chromatography (Hexane: EtOAc = 5:1) as a colorless liquid (44 mg, 70% yield).

^{1}H NMR (400 MHz, $CDCl_3$) δ 7.77 (d, *J* = 8.2 Hz, 2H), 7.37 (d, *J* = 8.0 Hz, 2H), 4.30 – 4.16 (m, 2H), 4.08 – 3.98 (m, 2H), 3.97 – 3.88 (m, 1H), 2.44 (s, 3H), 1.40 (s, 9H).

^{13}C NMR (101 MHz, $CDCl_3$) δ 155.7, 145.6, 134.0, 130.3, 128.5, 80.5, 51.3, 49.7, 28.3, 21.7.

Matching reported literature data.[12]

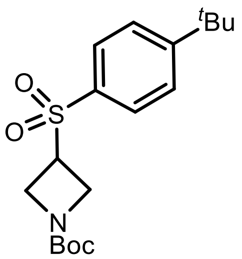

tert-butyl 3-((4-(tert-butyl)phenyl)sulfonyl)azetidine-1-carboxylate (61)

The title compound was prepared according to the ***General Procedure II*** and isolated by flash chromatography (Hexane: EtOAc = 5:1) as a colorless liquid (52 mg, 74% yield).

^{1}H NMR (400 MHz, $CDCl_3$) δ 7.83 – 7.78 (m, 2H), 7.61 – 7.55 (m, 2H), 4.32 – 4.20 (m, 2H), 4.05 (dd, *J* = 8.9, 8.9 Hz, 2H), 3.98 – 3.90 (m, 1H), 1.41 (s, 9H), 1.33 (s, 9H).

^{13}C NMR (101 MHz, $CDCl_3$) δ 158.4, 155.7, 134.0, 128.3, 126.7, 80.4, 51.2, 49.3, 35.4, 31.0, 28.3.

HRMS (ESI): $C_{13}H_{20}NO_2S^+$ (M+H - Boc)$^+$: 254.1215, found: 254.1216.

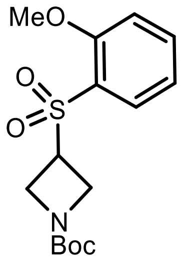

tert-butyl 3-((2-methoxyphenyl)sulfonyl)azetidine-1-carboxylate (62)

The title compound was prepared according to the ***General Procedure II*** and isolated by flash chromatography (Hexane: EtOAc = 2:1) as a colorless liquid (38 mg, 58% yield).

^{1}H NMR (400 MHz, $CDCl_3$) δ 7.98 (dd, *J* = 7.9, 1.8 Hz, 1H), 7.63 – 7.57 (m, 1H), 7.14 – 7.08 (m, 1H), 7.05 – 7.01 (m, 1H), 4.40 – 4.33 (m, 1H), 4.33 – 4.20 (m, 2H), 4.06 (t, *J* = 8.9 Hz, 2H), 3.95 (s, 3H), 1.42 (s, 9H).

^{13}C NMR (101 MHz, $CDCl_3$) δ 157.4, 155.8, 136.1, 130.9, 125.5, 120.9, 112.5, 80.3, 56.4, 50.0, 49.5, 28.3.

HRMS (ESI): $C_{10}H_{14}NO_3S^+$ (M+H - Boc)$^+$: 228.0694, found: 228.0703.

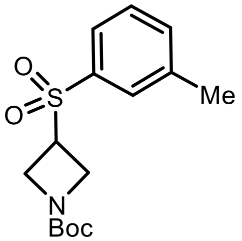

tert-butyl 3-(m-tolylsulfonyl)azetidine-1-carboxylate (63)

The title compound was prepared according to the ***General Procedure II*** and isolated by flash chromatography (Hexane: EtOAc = 5:1) as a colorless liquid (47 mg, 75% yield).

^{1}H NMR (400 MHz, CDCl$_3$) δ 7.73 – 7.64 (m, 2H), 7.50 – 7.42 (m, 2H), 4.30 – 4.20 (m, 2H), 4.04 (dd, *J* = 8.8, 8.8 Hz, 2H), 3.98 – 3.90 (m, 1H), 2.43 (s, 3H), 1.41 (s, 9H).

^{13}C NMR (101 MHz, CDCl$_3$) δ 155.7, 140.0, 136.9, 135.2, 129.5, 128.7, 125.5, 80.5, 51.2, 49.6, 28.3, 21.3.

HRMS (ESI): $C_{10}H_{14}NO_2S^+$ (M+H - Boc)$^+$: 212.0745, found: 212.0750.

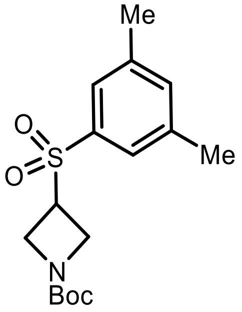

***tert*-butyl 3-(mesitylsulfonyl)azetidine-1-carboxylate (64)**

The title compound was prepared according to the ***General Procedure II*** and isolated by flash chromatography (Hexane: EtOAc = 5:1) as a white solid (40 mg, 61% yield).

^{1}H NMR (400 MHz, CDCl$_3$) δ 7.50 – 7.47 (m, 2H), 7.29 – 7.27 (m, 1H), 4.34 – 4.20 (m, 2H), 4.04 (dd, *J* = 8.9, 8.9 Hz, 2H), 3.97 – 3.86 (m, 1H), 2.39 (s, 6H) (two methyl group overlapped), 1.42 (s, 9H).

^{13}C NMR (101 MHz, CDCl$_3$) δ 155.8, 139.8, 136.8, 136.1, 125.8, 80.5, 51.1, 49.6, 28.3, 21.2.

HRMS (ESI): $C_{11}H_{16}NO_2S^+$ (M+H - Boc)$^+$: 226.0902, found: 226.0904.

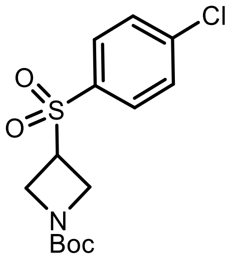

***tert*-butyl 3-((4-chlorophenyl)sulfonyl)azetidine-1-carboxylate (65)**

The title compound was prepared according to the ***General Procedure II*** *(reaction was conducted at room temperature)* and isolated by flash chromatography (Hexane: EtOAc = 5:1) as a colorless liquid (33 mg, 50% yield).

^{1}H NMR (400 MHz, CDCl$_3$) δ 7.88 – 7.81 (m, 2H), 7.59 – 7.53 (m, 2H), 4.29 – 4.18 (m, 2H), 4.06 (dd, *J* = 8.9, 8.9 Hz, 2H), 3.99 – 3.90 (m, 1H), 1.41 (s, 9H).

^{13}C NMR (101 MHz, CDCl$_3$) δ 155.7, 141.4, 135.5, 130.0, 129.9, 80.7, 51.4, 49.6, 28.3.

HRMS (ESI): $C_9H_{11}ClNO_2S^+$ (M+H - Boc)$^+$: 232.0199, found: 232.0191.

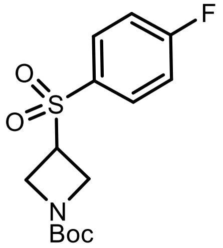

***tert*-butyl 3-((4-fluorophenyl)sulfonyl)azetidine-1-carboxylate (66)**

The title compound was prepared according to the ***General Procedure II*** and isolated by flash chromatography (Hexane: EtOAc = 4:1) as a colorless liquid (42mg, 65% yield).

^{1}H NMR (400 MHz, CDCl$_3$) δ 7.96 – 7.88 (m, 2H), 7.30 – 7.22 (m, 2H), 4.23 (dd, *J* = 9.6, 5.4 Hz, 2H), 4.06 (dd, *J* = 8.9, 8.9 Hz, 2H), 3.99 – 3.90 (m, 1H), 1.41 (s, 9H).

^{13}C NMR (101 MHz, CDCl$_3$) δ 166.3 (d, *J* = 257.8 Hz), 155.7, 133.1 (d, *J* = 3.3 Hz), 131.4 (d, *J* = 9.8 Hz), 117.1 (d, *J* = 22.6 Hz), 80.6, 51.4, 49.6, 28.3.

^{19}F NMR (376 MHz, CDCl$_3$) δ -101.95 – -102.03 (m, 1F).

HRMS (ESI): $C_9H_{11}FNO_2S^+$ (M+H - Boc)$^+$: 216.0495, found: 216.0490.

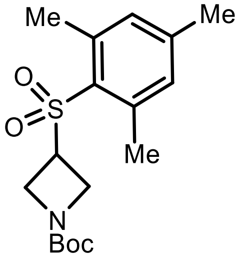

***tert*-butyl 3-(mesitylsulfonyl)azetidine-1-carboxylate (67)**

The title compound was prepared according to the ***General Procedure II*** and isolated by flash chromatography (Hexane: EtOAc = 5:1) as a colorless liquid (35 mg, 52% yield).

^{1}H NMR (400 MHz, CDCl$_3$) δ 7.00 – 6.94 (m, 2H), 4.35 – 4.20 (m, 2H), 4.10 – 3.95 (m, 3H), 2.66 (s, 6H) (two methyl group overlapped), 2.30 (s, 3H), 1.43 (s, 9H).

^{13}C NMR (101 MHz, CDCl$_3$) δ 155.9, 143.9, 140.2, 132.6, 131.4, 80.4, 51.0, 49.3, 28.3, 23.0, 21.0.

HRMS (ESI): $C_{12}H_{18}NO_2S^+$ (M+H - Boc)$^+$: 240.1058, found: 240.1061.

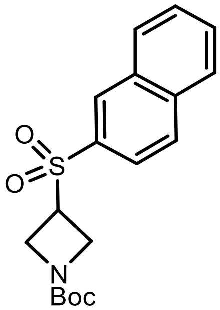

***tert*-butyl 3-(naphthalen-2-ylsulfonyl)azetidine-1-carboxylate (68)**

The title compound was prepared according to the ***General Procedure II*** and isolated by flash chromatography (Hexane: EtOAc = 5:1) as a colorless liquid (30 mg, 43% yield).

^{1}H NMR (400 MHz, CDCl$_3$) δ 8.5 (s, 1H), 8.05 – 7.98 (m, 2H), 7.96 – 7.92 (m, 1H), 7.87 – 7.82 (m, 1H), 7.73 – 7.63 (m, 2H), 4.40 – 4.26 (m, 2H), 4.13 – 4.01 (m, 3H), 1.40 (s, 9H).

^{13}C NMR (101 MHz, CDCl$_3$) δ 155.7, 135.6, 133.8, 132.2, 130.6, 130.0, 129.7, 129.5, 128.1, 128.0, 122.7, 80.6, 51.3, 49.4, 28.3.

HRMS (ESI): $C_{13}H_{14}NO_2S^+$ (M+H - Boc)$^+$: 248.0745, found: 248.0728.

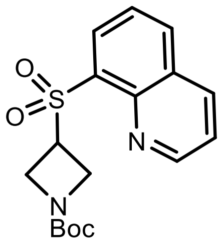

***tert*-butyl 3-(quinolin-8-ylsulfonyl)azetidine-1-carboxylate (69)**

The title compound was prepared according to the ***General Procedure II*** and isolated by flash chromatography (Hexane: EtOAc = 2:1) as a colorless liquid (28 mg, 40% yield).

^{1}H NMR (400 MHz, CDCl$_3$) δ 9.05 (dd, J = 4.3, 1.8 Hz, 1H), 8.57 (dd, J = 7.3, 1.5 Hz, 1H), 8.28 (dd, J = 8.4, 1.8 Hz, 1H), 8.13 (dd, J = 8.2, 1.5 Hz, 1H), 7.70 (dd, J = 8.2, 7.3 Hz, 1H), 7.57 (dd, J = 8.3, 4.3 Hz, 1H), 5.17 – 5.08 (m, 1H), 4.41 (br, 2H), 4.16 – 4.02 (m, 2H), 1.43 (s, 9H).

^{13}C NMR (101 MHz, CDCl$_3$) δ 155.9, 151.6, 144.1, 136.8, 134.9, 132.6, 131.2, 129.0, 125.7, 122.5, 80.3, 51.2, 49.6, 28.3.

HRMS (ESI): $C_{17}H_{21}N_2O_4S^+$ (M+H)$^+$: 349.1222, found: 349.1223.

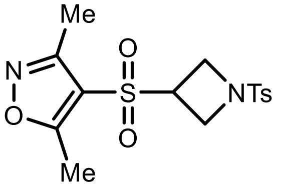

3,5-dimethyl-4-((1-tosylazetidin-3-yl)sulfonyl)isoxazole (70)

The title compound was prepared according to the ***General Procedure II*** and isolated by flash chromatography (Hexane: EtOAc = 2:1) as a yellow oil (44.4 mg, 60% yield).

^{1}H NMR (400 MHz, CDCl$_3$) δ 7.72 – 7.67 (m, 2H), 7.41 – 7.36 (m, 2H), 4.07 – 4.01 (m, 2H), 3.98 – 3.86 (m, 3H), 2.56 (s, 3H), 2.47 (s, 3H), 2.31 (s, 3H).

^{13}C NMR (101 MHz, CDCl$_3$) δ 175.9, 157.6, 145.1, 130.9, 130.1, 128.3, 113.3, 51.3, 50.4, 21.7, 12.7, 10.9.

HRMS (ESI): $C_{15}H_{19}N_2O_5S_2^+$ (M+H)$^+$: 371.0730, found: 371.0728.

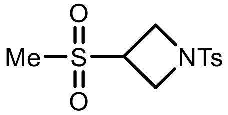

3-(methylsulfonyl)-1-tosylazetidine (71)

The title compound was prepared according to the ***General Procedure II*** and isolated by flash chromatography (Hexane: EtOAc = 1:3) as a white solid (28.5mg, 50% yield).

^{1}H NMR (400 MHz, CDCl$_3$) δ 7.76 – 7.70 (m, 2H), 7.43 – 7.37 (m, 2H), 4.14 – 4.02 (m, 4H), 3.87 – 3.77 (m, 1H), 2.82 (s, 3H), 2.47 (s, 3H).

^{13}C NMR (101 MHz, CDCl$_3$) δ 145.0, 130.9, 130.1, 128.5, 50.6, 49.2, 38.4, 21.7.

HRMS (ESI): $C_{11}H_{16}NO_4S_2^+$ (M+H)$^+$: 290.0515, found: 290.0522.

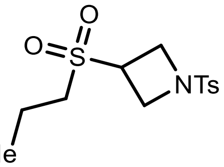

3-(propylsulfonyl)-1-tosylazetidine (72)

The title compound was prepared according to the ***General Procedure II*** and isolated by flash chromatography (Hexane: EtOAc = 2:1) as a white solid (34.2 mg, 54% yield).

^{1}H NMR (400 MHz, CDCl$_3$) δ 7.75 – 7.70 (m, 2H), 7.42 – 7.36 (m, 2H), 4.08 (d, J = 3.6 Hz, 2H), 4.06 (d, J = 1.9 Hz, 2H), 3.89 – 3.80 (m, 1H), 2.84 – 2.78 (m, 2H), 2.45 (s, 3H), 1.80 – 1.70 (m, 2H),1.03 (t, J = 7.4 Hz, 3H).

^{13}C NMR (101 MHz, CDCl$_3$) δ 144.9, 131.0, 130.1, 128.4, 53.2, 50.5, 47.6, 21.7, 15.3, 13.1.

HRMS (ESI): $C_{13}H_{20}NO_4S_2^+$ (M+H)$^+$: 318.0828, found: 318.0837.

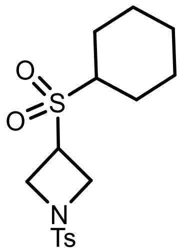

3-(cyclohexylsulfonyl)-1-tosylazetidine (73)

The title compound was prepared according to the ***General Procedure II*** and isolated by flash chromatography (Hexane: EtOAc = 2:1) as a yellow solid (43 mg, 60% yield).

^{1}H NMR (400 MHz, CDCl$_3$) δ 7.74 – 7.70 (m, 2H), 7.42 – 7.36 (m, 2H), 4.07 (d, J = 7.7 Hz, 4H), 3.96 – 3.86 (m, 1H), 2.76 – 2.66 (m, 1H), 2.45 (s, 3H), 2.00 – 1.93 (m, 2H), 1.91 – 1.84 (m, 2H), 1.71 – 1.64 (m, 1H), 1.43 – 1.32 (m, 2H), 1.28 – 1.18 (m, 3H).

^{13}C NMR (101 MHz, CDCl$_3$) δ 144.8, 131.0, 130.1, 128.4, 61.3, 50.7 (two carbon peaks overlapped), 44.9, 24.9 (two carbon peaks overlapped), 21.7.

HRMS (ESI): $C_{16}H_{24}NO_4S_2$+ $(M+H)^+$: 358.1147, found: 358.1154.

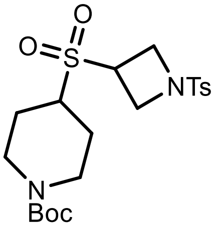

tert-butyl 4-((1-tosylazetidin-3-yl)sulfonyl)piperidine-1-carboxylate (74)

The title compound was prepared according to the ***General Procedure II*** and isolated by flash chromatography (Hexane: EtOAc = 1:1) as a white solid (45.8 mg, 50% yield).

^{1}H NMR (400 MHz, $CDCl_3$) δ 7.74 – 7.68 (m, 2H), 7.42 – 7.36 (m, 2H), 4.21 (br, 2H), 4.10 – 4.04 (m, 4H), 3.97 – 3.88 (m, 1H), 2.92 – 2.82 (m, 1H), 2.75 – 2.60 (m, 2H), 2.45 (s, 3H), 1.93 – 1.83 (m, 2H), 1.62 – 1.50 (m, 2H), 1.42 (s, 9H).

^{13}C NMR (101 MHz, $CDCl_3$) δ 154.2, 144.9, 130.8, 130.1, 128.4, 80.4, 59.3, 50.6, 45.1, 42.3 (br), 28.4, 24.3, 21.7.

HRMS (ESI): $C_{15}H_{23}N_2O_4S_2^+$ $(M+H - Boc)^+$: 359.1094, found: 359.1091.

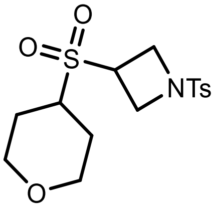

3-((tetrahydro-2*H*-pyran-4-yl)sulfonyl)-1-tosylazetidine (75)

The title compound was prepared according to the ***General Procedure II*** and isolated by flash chromatography (Hexane: EtOAc = 1:3) as a white solid (39.5 mg, 55% yield).

^{1}H NMR (400 MHz, $CDCl_3$) δ 7.75 – 7.69 (m, 2H), 7.43 – 7.35 (m, 2H), 4.11– 4.02 (m, 6H), 3.97– 3.87 (m, 1H), 3.37– 3.28 (m, 2H), 3.03– 2.94 (m, 1H), 2.46 (s, 3H), 1.85– 1.71 (m, 4H).

^{13}C NMR (101 MHz, $CDCl_3$) δ 144.9, 130.8, 130.1, 128.4, 66.2, 58.2, 50.5, 44.9, 24.9, 21.7.

HRMS (ESI): $C_{15}H_{22}NO_5S_2^+$ $(M+H)^+$: 360.0934, found: 360.0947.

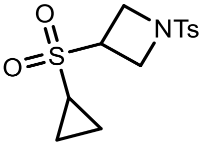

3-(cyclopropylsulfonyl)-1-tosylazetidine (76)

The title compound was prepared according to the ***General Procedure II*** and isolated by flash chromatography (Hexane: EtOAc = 1:1) as a white solid (44.8 mg, 71% yield).

^{1}H NMR (400 MHz, $CDCl_3$) δ 7.75 – 7.70 (m, 2H), 7.41 – 7.36 (m, 2H), 4.09 (d, J = 7.6 Hz, 4H), 3.96 – 3.87 (m, 1H), 2.45 (s, 3H), 2.27 – 2.20 (m, 1H), 1.18 – 1.13 (m, 2H), 1.04 – 0.98 (m, 2H).

^{13}C NMR (101 MHz, CDCl$_3$) δ 144.8, 131.0, 130.1, 128.4, 50.8, 48.60, 27.6, 21.7, 4.5.

HRMS (ESI): $C_{13}H_{18}NO_4S_2^+$ (M+H)$^+$: 316.0672, found: 316.0675.

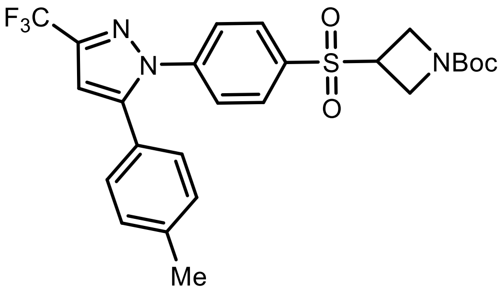

***tert*-butyl 3-((4-(5-(*p*-tolyl)-3-(trifluoromethyl)-1*H*-pyrazol-1-yl)phenyl)sulfonyl)azetidine-1-carboxylate (77)**

The title compound was prepared according to the ***General Procedure II*** and isolated by flash chromatography (Hexane: EtOAc = 1:3) as a yellow oil (47 mg, 45% yield).

^{1}H NMR (400 MHz, CDCl$_3$) δ 7.92 – 7.86 (m, 2H), 7.58 – 7.53 (m, 2H), 7.21 – 7.16 (m, 2H), 7.12 – 7.07 (m, 2H), 6.75 (s, 1H), 4.30 – 4.18 (m, 2H), 4.10 – 4.02 (m, 2H), 3.98 – 3.91 (m, 1H), 2.39 (s, 3H), 1.43 (s, 9H).

^{13}C NMR (101 MHz, CDCl$_3$) δ 155.7, 145.4, 144.0, 144.5 (q, J = 38.8 Hz), 140.1, 136.3, 129.9, 129.5, 128.7, 125.8, 125.5, 121.0 (q, J = 269.1 Hz), 106.6, 80.7, 51.3, 49.6, 28.3, 21.3.

^{19}F NMR (376 MHz, CDCl$_3$) δ -62.6.

HRMS (ESI): $C_{20}H_{19}F_3N_3O_2S^+$ (M+H - Boc)$^+$: 422.1145, found: 422.1150.

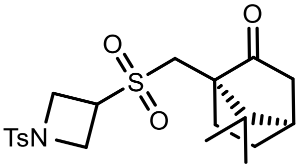

(1*S*,4*R*)-7,7-dimethyl-1-(((1-tosylazetidin-3-yl)sulfonyl)methyl)bicyclo[2.2.1]heptan-2-one (78)

The title compound was prepared according to the ***General Procedure II*** and isolated by flash chromatography (Hexane: EtOAc = 2:1) as a yellow oil (46.8mg, 55% yield).

^{1}H NMR (400 MHz, CDCl$_3$) δ 7.75 – 7.70 (m, 2H), 7.39 – 7.34 (m, 2H), 4.25 – 4.17 (m, 1H), 4.14 – 4.04 (m, 4H), 3.13 (d, J = 15.1 Hz, 1H), 2.66 (d, J = 15.2 Hz, 1H), 2.44 (s, 3H), 2.36 – 2.20 (m, 2H), 2.13 – 2.09 (m, 1H), 2.06 – 1.98 (m, 1H), 1.89 (d, J = 18.7 Hz, 1H), 1.61 (ddd, J = 14.0, 9.3, 4.6 Hz, 1H), 1.43 (ddd, J = 13.0, 9.3, 3.9 Hz, 1H), 0.97 (s, 3H), 0.81 (s, 3H).

^{13}C NMR (101 MHz, CDCl$_3$) δ 216.0, 144.5, 131.4, 130.0, 128.4, 58.6, 51.5, 50.2, 50.1, 49.1, 42.5, 27.1, 25.3, 21.7, 19.7, 19.3.

HRMS (ESI): $C_{20}H_{28}NO_5S_2^+$ (M+H)$^+$: 426.1403, found: 426.1409.

C4. Copper-Catalyzed Cyanation of Alkyl Bromides

C4.1. Optimization Studies

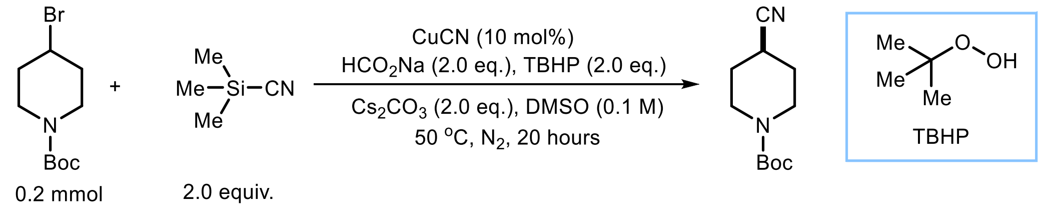

Table S3. Optimization of the model reaction

Entry	Deviations	NMRy (%)
1	none	65 (64)[a]
2	No **Cu**	< 5
3	No TBHP, HCO_2Na or Base	< 5
4	$Cu(CH_3CN)_4BF_4$ (10 mol%)	20
5	$Cu(CH_3CN)_4PF_6$ (10 mol%)	12
6	CuI (10 mol%)	33
7	CuBr (10 mol%)	36
8	CuTc (10 mol%)	10
9	CuOTf (10 mol%)	34
10	$Cu(acac)_2$ (10 mol%)	8
11	CH_3CN as solvent	0
12	DMA as solvent	< 5
13	DMF as solvent	10
14	K_3PO_4 as Base	36
15	K_2CO_3 as Base	16
16	CsF as Base	41
17	KF as Base	48
18	DTBP, TBPB or $K_2S_2O_8$ as oxidant	0
19	Room temperature	48

All reactions were performed on a 0.1 mmol scale; yield of prodduct determined by ^1H NMR analysis of the crude reaction mixture by comparison with 1,3,5-trimethoxybenzene as internal standard. [a] Yield of isolated product. DTBP: di-*tert*-butyl peroxide; TBPB: *tert*-butyl peroxybenzoate.

C4.2. General Procedure *III*: Cyantion of Alkyl Bromides

In a glove box, to a 4 mL glass vial, CuCN (1.8 mg, 0.02 mmol, 10 mol%), sodium formate (27.2 mg, 0.4 mmol, 2.0 equiv.), Cs_2CO_3 (130 mg, 0.4 mmol, 2.0 equiv.), alkyl bromides (if solid, 0.2 mmol, 1 equiv.) were sequentially added, then the vial was sealed with a screw-top cap with septum. Outside the glove box, DMSO (0.1 M, 2.0 mL), alkyl bromides (if liquid, 0.2 mmol, 1.0 equiv.) and TMSCN (50 µL, 0.4 mmol, 2 equiv.) followed by TBHP (60 µL, 0.4 mmol, 2.0 equiv.) were added *via* syringe. The vial was sealed with Parafilm and then stirred at 50 °C for 20 hours. After the reaction was complete, the reaction mixture was transferred to an extraction funnel, 10 mL of H_2O and 2 mL of brine were added and the organic layer was extracted with EtOAc. The organic layer was washed with brine twice. The combined organic layers were dried over anhydrous Na_2SO_4, filtered, and concentrated to dryness. The crude residue was purified by

column chromatography to afford the corresponding product in the stated yield with >95% purity according to ^{1}H NMR analysis.

C4.3. Characterization of Products

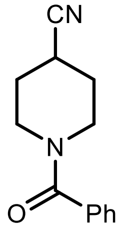

1-benzoylpiperidine-4-carbonitrile (79)

The title compound was prepared according to the ***General Procedure III*** and isolated by flash chromatography (Hexane: EtOAc = 3:1) as a yellow liquid (30 mg, 70% yield).

^{1}H NMR (400 MHz, $CDCl_3$) δ 7.49 – 7.32 (m, 5H), 4.16 – 3.20 (m, 4H), 2.96 – 2.87 (m, 1H), 2.10 – 1.65 (m, 4H).

^{13}C NMR spectrum was complicated due to the presence of rotamers.

^{13}C NMR (101 MHz, $CDCl_3$) δ 169.5, 134.3, 129.0, 127.6, 125.8, 119.7, 44.8 (br), 39.1 (br), 27.4 (br), 25.4.

Matching reported literature data.[13]

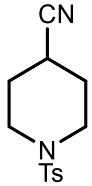

1-tosylpiperidine-4-carbonitrile (80)

The title compound was prepared according to the ***General Procedure III*** and isolated by flash chromatography (Hexane: EtOAc = 3:1) as a white solid (30 mg, 58% yield).

^{1}H NMR (400 MHz, $CDCl_3$) δ 7.66 – 7.59 (m, 2H), 7.36 – 7.30 (m, 2H), 3.16 – 3.06 (m, 4H), 2.76 – 2.68 (m, 1H), 2.44 (s, 3H), 2.04 – 1.90 (m, 4H).

^{13}C NMR (101 MHz, $CDCl_3$) δ 144.1, 132.8, 129.9, 127.6, 120.4, 43.8, 28.0, 25.4, 21.6.

Matching reported literature data.[14]

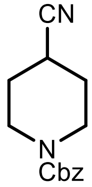

benzyl 4-cyanopiperidine-1-carboxylate (81)

The title compound was prepared according to the ***General Procedure III*** and isolated by flash

chromatography (Hexane: EtOAc = 3:1) as a colorless liquid (22 mg, 45% yield).

NMR (400 MHz, $CDCl_3$) δ 7.40 – 7.30 (m, 5H), 5.13 (s, 2H), 3.76 – 3.67 (m, 2H), 3.49 – 3.40 (m, 2H), 2.86 – 2.78 (m, 1H), 1.92 – 1.76 (m, 4H).

^{13}C NMR (101 MHz, $CDCl_3$) δ 155.0, 136.5, 128.6, 128.2, 128.0, 120.9, 67.4, 41.9, 28.3, 26.2.

Matching reported literature data.[15]

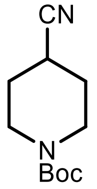

***tert*-butyl 4-cyanopiperidine-1-carboxylate (82)**

The title compound was prepared according to the ***General Procedure III*** and isolated by flash chromatography (Hexane: EtOAc = 5:1) as a white liquid (26 mg, 62% yield).

^{1}H NMR (400 MHz, $CDCl_3$) δ 3.68 – 3.61(m, 2H), 3.37 – 3.29 (m, 2H), 2.83 – 2.76 (m, 1H), 1.91 – 1.83 (m, 2H), 1.82 – 1.74 (m, 2H), 1.45 (s, 9H).

^{13}C NMR (101 MHz, $CDCl_3$) δ 153.4, 120.1, 79.1, 41.0, 27.4, 27.3, 25.3.

Matching reported literature data.[15]

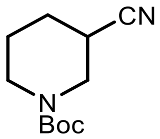

***tert*-butyl 3-cyanopiperidine-1-carboxylate (83)**

The title compound was prepared according to the ***General Procedure III*** and isolated by flash chromatography (Hexane: EtOAc = 4:1) as a colorless liquid (29 mg, 69% yield).

NMR (400 MHz, $CDCl_3$) δ 3.8 – 3.1 (m, 4H), 2.70 – 2.61 (m, 1H), 2.04 – 1.93 (m, 1H), 1.87 – 1.73 (m, 2H), 1.56 – 1.48 (m, 1H), 1.45 (s, 9H).

^{13}C NMR (101 MHz, $CDCl_3$) δ 154.2, 120.1, 80.5, 45.2, 43.5, 28.3, 27.9, 27.4, 23.1.

Matching reported literature data.[16]

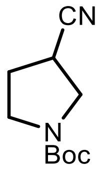

***tert*-butyl 3-cyanopyrrolidine-1-carboxylate (84)**

The title compound was prepared according to the ***General Procedure III*** and isolated by flash chromatography (Hexane: EtOAc = 6:1) as a yellow liquid (21 mg, 54% yield).

^{1}**H NMR (400 MHz, CDCl$_3$)** δ 3.70 – 3.35 (m, 4H), 3.15 – 3.01 (m, 1H), 2.30 – 2.11 (m, 2H), 1.44 (s, 9H).

^{13}**C NMR (101 MHz, CDCl$_3$)** δ 153.8, 120.0, 80.2, 48.9, 44.5, 30.2, 29.4, 28.4.

Matching reported literature data.[17]

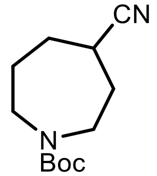

***tert*-butyl 4-cyanoazepane-1-carboxylate (85)**

The title compound was prepared according to the ***General Procedure III*** and isolated by flash chromatography (Hexane: EtOAc = 4:1) as a colorless liquid (29 mg, 65% yield).

NMR (400 MHz, CDCl$_3$) δ 3.60 – 3.30 (m, 4H), 2.85 – 2.77 (m, 1H), 2.03 – 1.83 (m, 5H), 1.80 – 1.73 (m, 1H), 1.45 (s, 9H).

^{13}C NMR spectrum was complicated due to the presence of rotamers.

^{13}**C NMR (101 MHz, CDCl$_3$)** δ 155.3&155.2, 121.8&121.8, 79.8&79.7, 46.2&45.3, 43.9&43.7, 32.0&31.8, 29.7&29.4, 29.4&29.1, 28.4, 25.6&25.47.

Matching reported literature data.[15]

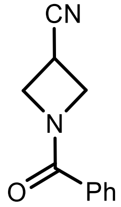

1-benzoylazetidine-3-carbonitrile (86)

The title compound was prepared according to the ***General Procedure III*** and isolated by flash chromatography (Hexane: EtOAc = 1:1) as a white solid (24 mg, 65% yield).

NMR (400 MHz, CDCl$_3$) δ 7.63 – 7.56 (m, 2H), 7.54 – 7.47 (m, 1H), 7.47 – 7.40 (m, 2H), 4.56 – 4.38 (m, 4H), 3.60 – 3.49 (m, 1H).

^{13}**C NMR (101 MHz, CDCl$_3$)** δ 170.3, 131.5, 131.5, 128.4, 127.5, 118.8, 17.6 (one C peak cannot be assigned ambiguously due to the broadness of the peak).

Matching reported literature data.[18]

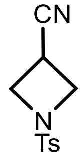

1-tosylazetidine-3-carbonitrile (87)

The title compound was prepared according to the ***General Procedure III*** and isolated by flash chromatography (Hexane: EtOAc = 3:1) as a white solid (41 mg, 87% yield).

NMR (400 MHz, $CDCl_3$) δ 7.74 – 7.68 (m, 2H), 7.42 – 7.37 (m, 2H), 4.05 (dd, *J* = 8.5, 8.5 Hz, 1H), 3.89 (dd, *J* = 8.1, 6.8 Hz, 2H), 3.37 – 3.25 (m, 2H), 2.46 (s, 3H).

^{13}C NMR (101 MHz, $CDCl_3$) δ 145.1, 130.6, 130.2, 128.4, 118.3, 53.3, 21.7, 16.9.

Matching reported literature data.[15]

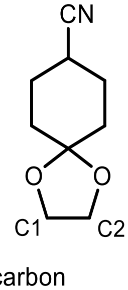

1,4-dioxaspiro[4.5]decane-8-carbonitrile (88)

The title compound was prepared according to the ***General Procedure III*** and isolated by flash chromatography (Hexane: EtOAc = 10:1) as a yellow liquid (17 mg, 51% yield).

^{1}H NMR (400 MHz, $CDCl_3$) δ 3.97 – 3.91 (m, 4H), 2.69 – 2.61 (m, 1H), 2.00 – 1.88 (m, 4H), 1.87 – 1.80 (m, 2H), 1.65 – 1.58 (m, 2H).

^{13}C NMR (101 MHz, $CDCl_3$) δ 121.8, 107.1, 64.5, 64.4, 32.7, 27.0, 26.7. C1 and C2 were observed simultaneously.

Matching reported literature data.[15]

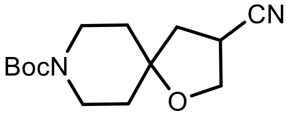

***tert*-butyl 3-cyano-1-oxa-8-azaspiro[4.5]decane-8-carboxylate (89)**

The title compound was prepared according to the ***General Procedure III*** and isolated by flash chromatography (Hexane: EtOAc = 5:1) as a yellow liquid (36 mg, 68% yield).

^{1}H NMR (400 MHz, $CDCl_3$) δ 4.08 (dd, *J* = 9.1, 7.2 Hz, 1H), 3.99 (dd, *J* = 9.1, 6.3 Hz, 1H), 3.67 (br, 2H), 3.30 – 3.16 (m, 2H), 3.16 – 3.08 (m, 1H), 2.15 – 2.08 (m, 1H), 2.06 – 1.99 (m, 1H), 1.80 – 1.73 (m, 1H), 1.66 – 1.50 (m, 3H), 1.43 (s, 9H).

^{13}C NMR (101 MHz, $CDCl_3$) δ 154.6, 120.6, 81.2, 79.6, 68.6, 41.5, 41.1, 35.9, 28.4, 28.4.

HRMS (ESI): $C_9H_{16}N_2O^+$ $(M+H - Boc)^+$: 167.1184, found: 167.1186.

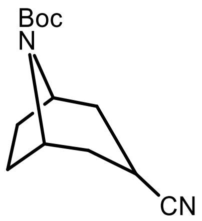

tert-butyl -3-cyano-8-azabicyclo[3.2.1]octane-8-carboxylate (90)

The title compound was prepared according to the ***General Procedure III*** and isolated by flash chromatography (Hexane: EtOAc = 6:1) as a yellow liquid (29 mg, 61% yield, d.r. = 1.2: 1).

^{1}H NMR (400 MHz, $CDCl_3$) δ 4.40 – 4.07 (d, 4.4H), 3.00 – 2.90 (m, 2.2H), 2.23 – 2.18 (m, 2.2H), 2.16 – 2.14 (m, 1.2H), 2.11 – 1.95 (m, 7.6H), 1.88 – 1.80 (m, 4.4H), 1.64 – 1.56 (m, 2.2H), 1.46 (s, 9H), 1.44 (s, 10.8H).

^{13}C NMR (101 MHz, $CDCl_3$) δ 153.0&153.0, 124.4&121.7, 79.97&79.76, 52.8&52.2, 34.2&33.5, 32.1&31.4, 31.8&30.9, 29.7&29.3, 28.5&28.5, 27.6&27.0, 20.67&19.37.

Matching reported literature data.[15]

C4. Other Alkyl Bromides and Nucleophiles Evaluated

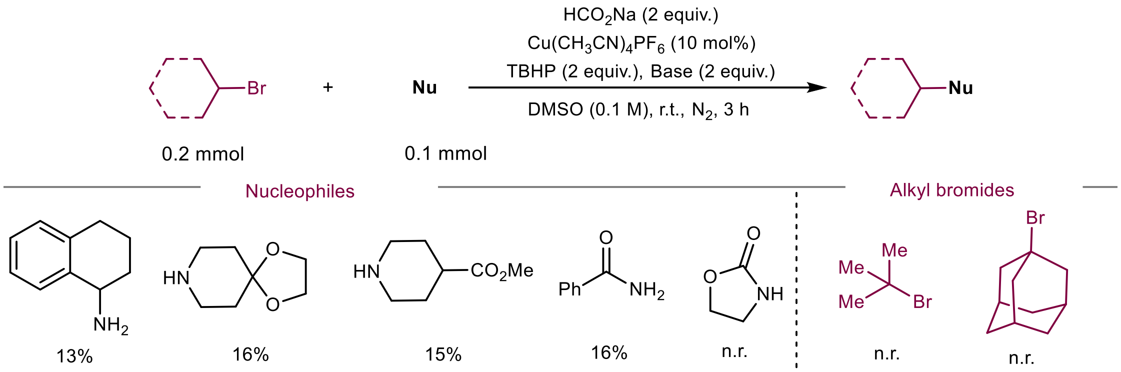

Figure S2. Other alkyl bromides and nucleophiles.

D. High-throughput Experimentation (HTE) for Reaction Optimization

In a nitrogen-filled glovebox, a 96 well plate of 1 mL vials was charged with insoluble solids (e.g., Cs_2CO_3 (2.0 equiv, 20 μmol) and HCO_2Na (2.0 equiv, 20 μmol)). A solution of $Cu(CH_3CN)_4PF_6$ (0.1 equiv. 1 μmol) in DMSO (20 μL), alkyl bromides (2 equiv, 20 μmol) in DMSO (20 μL), BTMG (2 equiv. 20 μmol) in DMSO (20 μL) (For the reactions using BTMG as base) and nucleophiles (1 equiv, 10 μmol) in DMSO (20 μL) was added followed by a solution of TBHP (2.0 equiv, 20 μmol) in DMSO (20 μL). And then, the plate was stirred for 3 hours at room temperature. After the reactions complicated, aliquots of 25 μL were taken from the supernatants and diluted with 0.75 mL MeCN. Analysis was performed by Liquid Chromatography-Mass Spectrometry (LC-MS) and the ratios of the absolute areas of the product: whole reaction components was used for analyzing reaction outcomes.

12 drug molecules as N-nuclephiles:

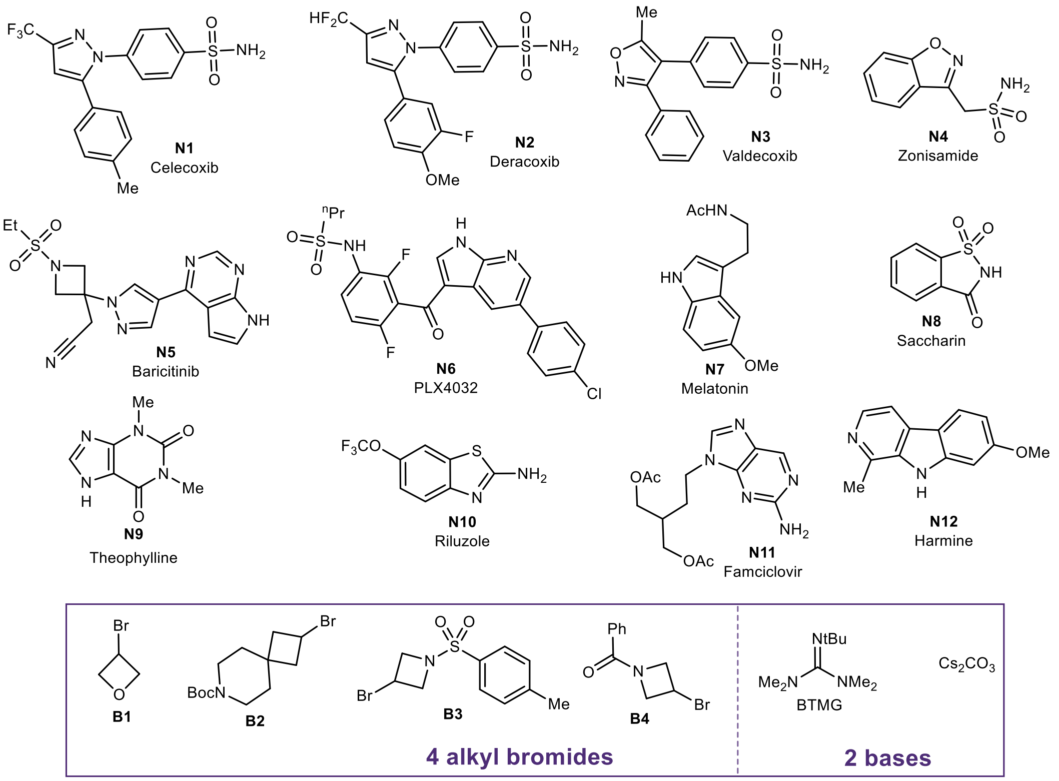

Figure S3. The drug molecules and alkyl bromides for HTE optimization.

Table S4. Results of HTE optimization

	N1	N2	N3	N4	N5	N6	N7	N8	N9	N10	N11	N12
B1	40%	26%	32%	35%	33%	10%	22%	nd	21%	30%	nd	5%
B2	6%	31%	4%	6%	10%	5%	5%	nd	12%	nd	nd	nd
B3	20%	23%	24%	21%	27%	7%	8%	nd	9%	23%	nd	7%
B4	22%	22%	5%	10%	10%	5%	7%	nd	6%	38%	nd	20%
B1	52%	40%	34%	54%	58%	24%	23%	nd	20%	24%	nd	25%
B2	20%	21%	22%	<5%	13%	6%	25%	nd	5%	30%	nd	6%
B3	36%	38%	30%	21%	23%	4%	23%	nd	8%	6%	nd	4%
B4	50%	26%	16%	20%	22%	8%	26%	nd	6%	3%	nd	5%

[a]Analysis was performed by LC-MS and the ratios of the absolute areas of the product: whole reaction components were use for analyzing reaction outcomes. The black numbers refer to the reaction using Cs_2CO_3 as the base, while the blue numbers refer to the reaction using BTMG as the base.

E. Mechanistic Studies

E1. Proof of Alkyl Radical Generation

To probe the generation of alkyl radical in our reaction system, we carried out the standard reaction in the presence of DMPO as radical trapping agent. After 3 hours, the radical addition product can be detected by high-resolution mass spectrometry (HR-MS), confirming the generation of alkyl radicals under the reaction conditions.

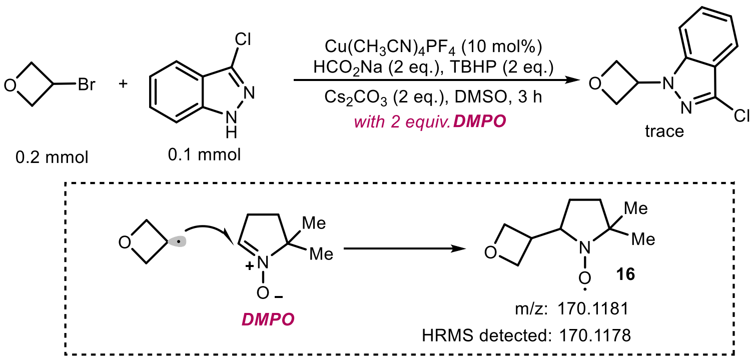

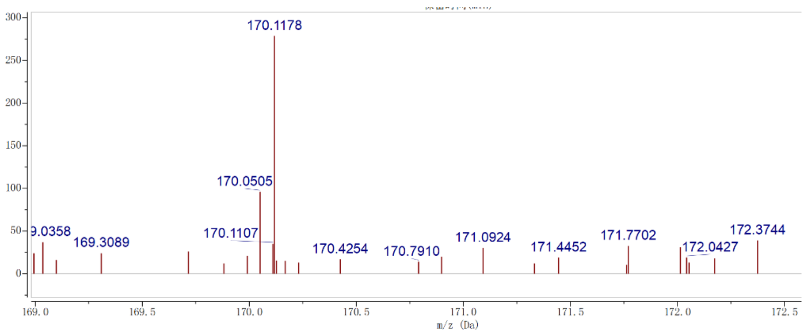

Figure S4. Characterization of alkyl radical using DMPO under standard condition.

A parallel reaction conducted in the absence of the *N*-containing nucleophiles also lead to the detection of addition product, with an even stronger signal intensity.

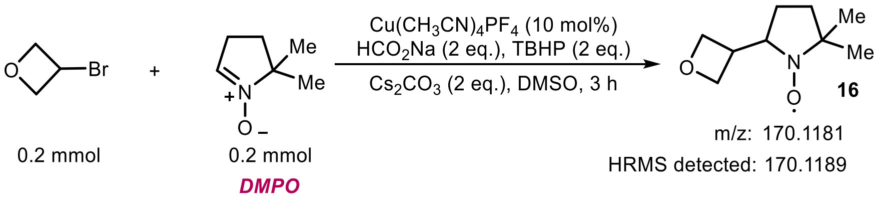

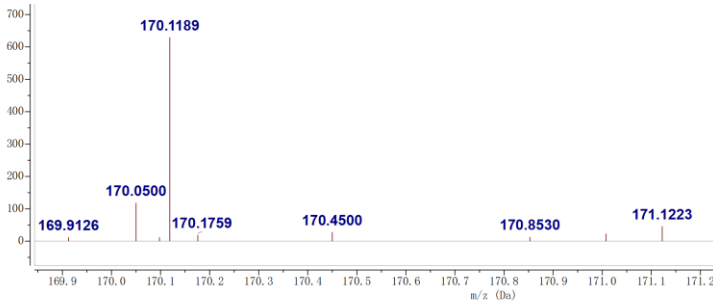

Figure S5. Characterization of alkyl radical using DMPO in the absence of nucleophiles.

The generation of alkyl radical in our reaction was further confirmed by a trapping experiment. Using *tert*-butyl acrylate as a general radical acceptor, the alkyl radical was captured successfully in about 18% yield, while generating both hydrogenation and elimination products. Based on this finding, we conclude that the alkyl bromides can be reduced to the corresponding alkyl radicals in our system.

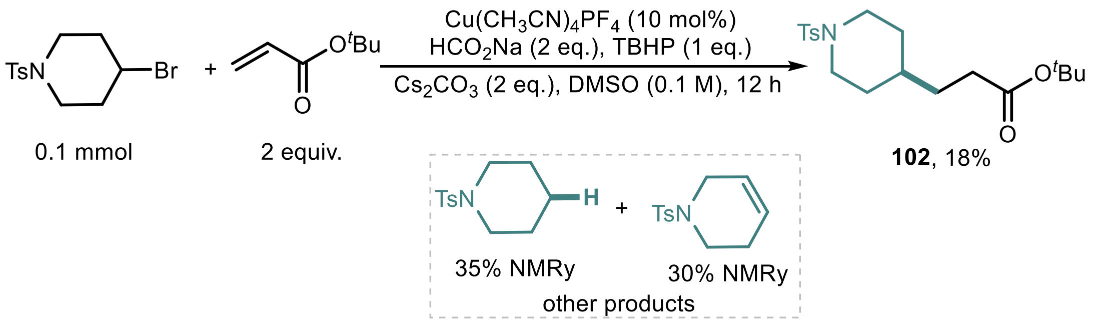

Figure S6. The capture of alkyl radical by tert-butyl acrylate.

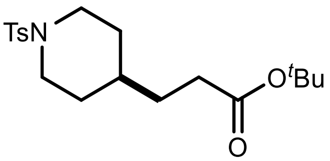

tert-butyl 3-(1-tosylpiperidin-4-yl)propanoate (102)

^{1}H NMR (400 MHz, CDCl$_3$) δ 7.67 – 7.58 (m, 2H), 7.34 – 7.27 (m, 2H), 3.81 – 3.69 (m, 2H), 2.42 (s, 3H), 2.26 – 2.13 (m, 4H), 1.75 – 1.62 (m, 2H), 1.55 – 1.45 (m, 2H), 1.40 (s, 9H), 1.35 – 1.21 (m, 3H).

^{13}C NMR (101 MHz, CDCl$_3$) δ 171.8, 142.4, 132.2, 128.6, 126.7, 79.2, 45.3, 33.6, 31.7, 30.2, 30.0, 27.1, 20.5.

Matching reported literature data.[19]

E2. Capture of $CO_2^{\bullet-}$ Intermediate using Radical Acceptor

To confirm the generation of $CO_2^{\bullet-}$ in our reaction system, we used phenyl vinyl sulfone **5** as radical

acceptor to capture the $CO_2^{\bullet-}$. After 3 hours, the expected addition product, 3-(phenylsulfonyl)propanoic acid **15**, was isolated in 23% yield following acidification. This result strongly supports the generation of $CO_2^{\bullet-}$ under standard conditions.

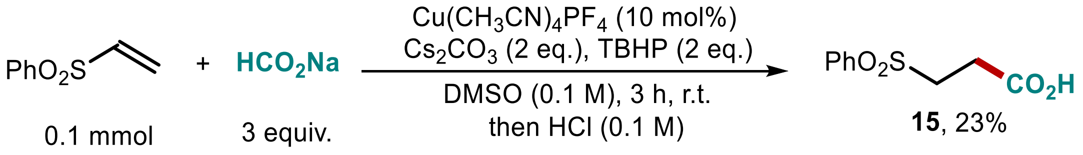

Figure S7. The capture of $CO_2^{\bullet-}$ species using phenyl vinyl sulfone as acceptor.

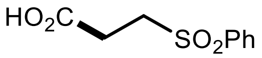

3-(phenylsulfonyl)propanoic acid (15)

^{1}H NMR (400 MHz, $CDCl_3$) δ 7.89 – 7.82 (m, 2H), 7.66 – 7.59 (m, 1H), 7.56 – 7.49 (m, 2H), 3.35 (t, *J* = 7.6 Hz, 2H), 2.74 (t, *J* = 7.6 Hz, 2H).

^{13}C NMR (101 MHz, $CDCl_3$) δ 175.2, 138.4, 134.2, 129.5, 128.2, 51.2, 27.5.

Matching reported literature data.[1] (*NMR spectra are recorded in* ***page S263***)

E3. Density Functional Theory (DFT) Computational Studies

Density Functional Theory (DFT) calculations were performed using Gaussian 16[20] at the B3LYP[21-23] level of theory with the D3 dispersion correction and Becke–Johnson damping[24-25] (B3LYP-D3BJ), employing the double-ζ quality def2-SVP[26-27] basis set. All optimizations were conducted within the SMD[28] solvation model using DMSO as the solvent. Each reactant was individually optimized to locate its most stable structure. Subsequently, the two optimized reactants were placed together in a single box with an initial intermolecular distance of 4 Å, and a geometry optimization was carried out under the same conditions. Throughout this process, the variation of total energy, Mulliken charge distribution, and spin density was monitored at each optimization step.

Table S5. Variation of relative electronic energy, Mulliken charge, and spin density during geometry optimization

Optimization step	ΔE (kcal/mol)	Mulliken charge			Spin density		
		CO_2	Br	C_3H_7	CO_2	Br	C_3H_7
**1**	0.0	-0.98	-0.19	0.18	0.98	0.00	0.01
**2**	-0.1	-0.98	-0.20	0.18	0.98	0.00	0.02
**3**	1.0	-0.97	-0.21	0.18	0.97	0.00	0.02

**4**	-0.6	-0.97	-0.21	0.18	0.97	0.01	0.03
**5**	-1.2	-0.94	-0.22	0.16	0.94	0.01	0.05
**6**	-2.2	-0.89	-0.24	0.13	0.89	0.01	0.11
**7**	-3.1	-0.82	-0.26	0.08	0.81	0.01	0.18
**8**	-3.4	-0.79	-0.26	0.05	0.77	0.01	0.23
**9**	-3.1	-0.80	-0.26	0.06	0.79	0.02	0.20
**10**	-3.6	-0.76	-0.27	0.04	0.74	0.00	0.26
**11**	-3.8	-0.74	-0.28	0.02	0.71	0.00	0.29
**12**	-4.1	-0.67	-0.31	-0.02	0.62	0.00	0.39
**13**	-4.5	-0.57	-0.37	-0.07	0.48	0.00	0.53
**14**	-5.4	-0.44	-0.45	-0.11	0.30	0.01	0.69
**15**	-8.2	-0.24	-0.64	-0.12	0.09	0.05	0.86
**16**	-13.7	-0.15	-0.74	-0.11	0.04	0.06	0.90
**17**	-19.7	-0.09	-0.83	-0.08	0.01	0.05	0.94
**18**	-23.5	-0.08	-0.85	-0.07	0.01	0.04	0.95
**19**	-24.6	-0.06	-0.88	-0.06	0.00	0.03	0.96
**20**	-25.0	-0.05	-0.89	-0.06	0.00	0.03	0.97
**21**	-25.3	-0.05	-0.90	-0.05	0.00	0.02	0.98
**22**	-26.0	-0.05	-0.91	-0.04	0.00	0.02	0.98
**23**	-26.3	-0.05	-0.92	-0.04	0.00	0.01	0.99
**24**	-26.5	-0.05	-0.92	-0.03	0.00	0.01	0.99
**25**	-26.6	-0.05	-0.93	-0.02	0.00	0.01	0.99
**26**	-26.7	-0.05	-0.93	-0.02	0.00	0.01	0.99
**27**	-27.0	-0.05	-0.93	-0.02	0.00	0.00	1.00
**28**	-27.1	-0.05	-0.93	-0.02	0.00	0.00	1.00
**29**	-27.1	-0.05	-0.93	-0.02	0.00	0.00	1.00
**30**	-27.5	-0.05	-0.92	-0.03	0.00	0.00	1.00
**31**	-27.8	-0.05	-0.91	-0.04	0.00	0.00	1.00
**32**	-28.2	-0.05	-0.90	-0.05	0.00	0.00	1.00
**33**	-28.4	-0.04	-0.89	-0.07	0.00	0.00	1.00
**34**	-28.5	-0.05	-0.87	-0.07	0.00	0.00	1.00
**35**	-28.5	-0.05	-0.89	-0.06	0.00	0.00	1.00
**36**	-28.7	-0.05	-0.88	-0.07	0.00	0.00	1.00
**37**	-28.7	-0.05	-0.88	-0.07	0.00	0.00	1.00
**38**	-28.7	-0.05	-0.88	-0.07	0.00	0.00	1.00
**39**	-28.8	-0.05	-0.88	-0.07	0.00	0.00	1.00
**40**	-28.8	-0.05	-0.88	-0.07	0.00	0.00	1.00
**41**	-28.8	-0.05	-0.88	-0.07	0.00	0.00	1.00
**42**	-28.8	-0.05	-0.88	-0.07	0.00	0.00	1.00

**43**	-28.7	-0.05	-0.88	-0.07	0.00	0.00	1.00
**44**	-28.8	-0.05	-0.88	-0.07	0.00	0.00	1.00
**45**	-28.8	-0.05	-0.88	-0.07	0.00	0.00	1.00
**46**	-28.8	-0.05	-0.88	-0.07	0.00	0.00	1.00
**47**	-28.8	-0.05	-0.88	-0.07	0.00	0.00	1.00
**48**	-28.8	-0.05	-0.88	-0.07	0.00	0.00	1.00
**49**	-28.7	-0.05	-0.88	-0.07	0.00	0.00	1.00
**50**	-28.8	-0.05	-0.88	-0.07	0.00	0.00	1.00
**51**	-28.8	-0.05	-0.88	-0.07	0.00	0.00	1.00
**52**	-28.8	-0.05	-0.88	-0.07	0.00	0.00	1.00
**53**	-28.8	-0.05	-0.88	-0.07	0.00	0.00	1.00
**54**	-28.5	-0.04	-0.88	-0.07	0.00	0.00	1.00
**55**	-28.8	-0.05	-0.88	-0.07	0.00	0.00	1.00
**56**	-28.8	-0.05	-0.88	-0.07	0.00	0.00	1.00
**57**	-28.8	-0.05	-0.88	-0.07	0.00	0.00	1.00
**58**	-28.8	-0.05	-0.88	-0.07	0.00	0.00	1.00
**59**	-28.8	-0.05	-0.88	-0.07	0.00	0.00	1.00
**60**	-28.8	-0.05	-0.88	-0.07	0.00	0.00	1.00

Optimization step 1

C	-2.417338	-0.034131	-0.174438
H	-2.774284	-0.178620	-1.203281
C	-2.868286	-1.180893	0.712733
H	-2.555126	-2.153486	0.307806
H	-2.456119	-1.078570	1.728396
H	-3.969596	-1.174232	0.786066
C	-2.817772	1.336559	0.341658
H	-2.404068	1.515422	1.346040
H	-2.469015	2.137954	-0.324757
H	-3.918051	1.394576	0.407420
Br	-0.432757	-0.104263	-0.380076
C	3.537641	-0.028384	0.099696
O	4.465071	-0.850841	0.180769
O	3.420840	1.179249	0.366365

Optimization step 2

C	-2.412790	-0.021832	-0.169534
H	-2.775071	-0.124931	-1.201005
C	-2.862174	-1.200552	0.674858
H	-2.558004	-2.158720	0.230063
H	-2.447166	-1.139027	1.692935
H	-3.962477	-1.189517	0.751411
C	-2.803538	1.329204	0.401636
H	-2.387138	1.467339	1.411602
H	-2.457556	2.155341	-0.235811
H	-3.902818	1.385338	0.472977
Br	-0.416372	-0.092438	-0.390846
C	3.476104	-0.044585	0.184882
O	4.388913	-0.881705	0.204920
O	3.445794	1.189967	0.295879

Optimization step 3

C	-2.392776	-0.043687	-0.262870
H	-2.629762	-0.280357	-1.307362
C	-2.937269	-1.110548	0.666692

H	-2.596107	-2.116772	0.378596
H	-2.648944	-0.917179	1.712281
H	-4.036825	-1.093215	0.607069
C	-2.829240	1.367515	0.079647
H	-2.540347	1.638351	1.107733
H	-2.409929	2.107261	-0.618052
H	-3.927220	1.416858	0.009741
Br	-0.349795	-0.129929	-0.239538
C	3.412890	0.042355	-0.056847
O	4.241707	-0.865139	-0.016989
O	3.447083	1.147482	0.508749

Optimization step 4

C	-2.391603	-0.025061	-0.228303
H	-2.675539	-0.186673	-1.275898
C	-2.903928	-1.150924	0.649196
H	-2.581866	-2.135444	0.280305
H	-2.564916	-1.031443	1.690266
H	-4.005900	-1.127105	0.643490
	-2.803009	1.359175	0.234887
C	-2.463292	1.553036	1.264471
H	-2.407356	2.145109	-0.424561
H	-3.903368	1.421865	0.220517
Br	-0.363731	-0.119440	-0.303644
C	3.398842	-0.014823	0.121573
O	4.273611	-0.884516	0.092534
O	3.417765	1.200872	0.353072

Optimization step 5

C	-2.335846	-0.042429	-0.292305
H	-2.521977	-0.263702	-1.350085
C	-2.898321	-1.127297	0.601573
H	-2.540111	-2.125407	0.309816
H	-2.648476	-0.946891	1.658874
H	-3.996435	-1.119359	0.505110
C	-2.782715	1.360397	0.060496
H	-2.532660	1.611627	1.103232
H	-2.340219	2.112375	-0.608735
H	-3.878454	1.411421	-0.048455
Br	-0.282172	-0.117637	-0.199496
C	3.242750	0.030824	0.017411
O	4.016872	-0.919775	-0.061524
O	3.355522	1.183308	0.447718

Optimization step 6

C	-2.269262	-0.052831	-0.313405
H	-2.411207	-0.282668	-1.375768
C	-2.822922	-1.145260	0.571973
H	-2.437513	-2.135966	0.289790
H	-2.601007	-0.955176	1.633581
H	-3.919822	-1.163969	0.453368
C	-2.739954	1.343725	0.021485
H	-2.520207	1.602558	1.068953
H	-2.292295	2.098055	-0.641313
H	-3.834283	1.381720	-0.114230
Br	-0.194848	-0.085985	-0.164764
C	3.025631	0.049211	0.013676
O	3.641843	-1.000029	-0.123758
O	3.317540	1.162011	0.460005

Optimization step 7

C	-2.203715	-0.037245	-0.287538
H	-2.377091	-0.212504	-1.356764
C	-2.705169	-1.180054	0.560745
H	-2.314084	-2.145410	0.211764
H	-2.425082	-1.043219	1.615926
H	-3.810977	-1.224348	0.508800
C	-2.664964	1.334455	0.141563
H	-2.387157	1.536470	1.186860
H	-2.241608	2.123201	-0.495139
H	-3.768630	1.395512	0.068361
Br	-0.119143	-0.047049	-0.225038
C	2.819027	0.044118	0.122536
O	3.198332	-1.112464	-0.035764
O	3.304614	1.143637	0.399850

Optimization step 8

C	-2.178904	-0.052057	-0.319507
H	-2.321619	-0.265975	-1.386629

C	-2.721025	-1.166479	0.546999
H	-2.319595	-2.145926	0.249288
H	-2.481832	-0.995563	1.608803
H	-3.821993	-1.210873	0.454656
C	-2.677528	1.329439	0.041191
H	-2.440667	1.575399	1.088718
H	-2.240910	2.102222	-0.608152
H	-3.776402	1.372916	-0.073056
Br	-0.077754	-0.049317	-0.165432
C	2.762517	0.054533	0.066688
O	3.149972	-1.089375	-0.130833
O	3.226785	1.127034	0.436365

Optimization step 9

C	-2.215420	-0.065514	-0.335395
H	-2.373008	-0.290349	-1.400321
C	-2.793112	-1.166851	0.536936
H	-2.392714	-2.155957	0.265382
H	-2.559051	-0.988810	1.600865
H	-3.891533	-1.204785	0.438095
C	-2.742135	1.317222	0.006521
H	-2.508245	1.579454	1.052888
H	-2.302178	2.090936	-0.642356
H	-3.838586	1.355560	-0.111519
Br	-0.116782	-0.066293	-0.145944
C	2.898306	0.038046	0.069920
O	3.473177	-1.020781	-0.154629
O	3.160179	1.170380	0.434270

Optimization step 10

C	-2.179476	-0.063933	-0.339440
H	-2.313325	-0.272554	-1.410116
C	-2.727557	-1.189722	0.514301
H	-2.308325	-2.166007	0.223679
H	-2.504256	-1.023371	1.582029
H	-3.824886	-1.246735	0.407631
C	-2.709590	1.307908	0.025287
H	-2.489560	1.551014	1.078861
H	-2.272946	2.096343	-0.607722
H	-3.805553	1.337552	-0.103054
Br	-0.061330	-0.036238	-0.141500
C	2.741643	0.021329	0.123371
O	3.206655	-1.067913	-0.171589
O	3.157757	1.135239	0.401600

Optimization step 11

C	-2.173913	-0.069625	-0.338612
H	-2.304240	-0.280826	-1.409508
C	-2.711243	-1.195548	0.519526
H	-2.283216	-2.168417	0.232060
H	-2.483287	-1.024229	1.585043
H	-3.809998	-1.264536	0.419707
C	-2.703804	1.301055	0.026216
H	-2.477690	1.545167	1.077822
H	-2.267484	2.088824	-0.607317
H	-3.801748	1.334639	-0.096718
Br	-0.048625	-0.032503	-0.146240
C	2.724260	0.024845	0.104763
O	3.186515	-1.073912	-0.161153
O	3.103200	1.141740	0.416899

Optimization step 12

C	-2.165764	-0.083179	-0.351172
H	-2.267085	-0.304976	-1.423401
C	-2.689760	-1.207986	0.511491
H	-2.236051	-2.173798	0.240489
H	-2.471584	-1.021437	1.576214
H	-3.788412	-1.307320	0.409142
C	-2.704905	1.285514	-0.003400
H	-2.485139	1.539011	1.047063
H	-2.262280	2.069899	-0.636507
H	-3.804447	1.324516	-0.132946
Br	-0.000377	-0.019650	-0.128533
C	2.651641	0.017817	0.097183
O	3.103596	-1.075987	-0.193253
O	2.994017	1.137095	0.430002

Optimization step 13

C	-2.190602	-0.096580	-0.357204
H	-2.254403	-0.318479	-1.432456

C	-2.674844	-1.230876	0.504215
H	-2.184801	-2.178133	0.234304
H	-2.452355	-1.036228	1.565461
H	-3.774621	-1.380365	0.418786
C	-2.724396	1.267251	-0.008422
H	-2.493532	1.518621	1.039083
H	-2.276346	2.049836	-0.638349
H	-3.829649	1.328920	-0.127592
Br	0.051497	-0.004661	-0.125701
C	2.608200	0.017760	0.069547
O	3.046411	-1.087023	-0.186274
O	2.872735	1.141229	0.447708

Optimization step 14

C	-2.247957	-0.107914	-0.375884
H	-2.240907	-0.356286	-1.447003
C	-2.710164	-1.220749	0.507258
H	-2.183856	-2.155973	0.273848
H	-2.496212	-0.988185	1.560915
H	-3.810296	-1.424039	0.434811
C	-2.749852	1.268265	-0.071270
H	-2.517457	1.538867	0.969508
H	-2.266225	2.020674	-0.708960
H	-3.858183	1.384661	-0.198331
Br	0.109116	-0.008937	-0.095104
C	2.597662	0.023303	0.055247
O	2.989569	-1.082791	-0.247696
O	2.787423	1.147245	0.466667

Optimization step 15

C	-2.454490	-0.092601	-0.387978
H	-2.337539	-0.369567	-1.443640
C	-2.874445	-1.167816	0.518494
H	-2.313970	-2.082112	0.311399
H	-2.640844	-0.893442	1.552925
H	-3.980048	-1.447180	0.509189
C	-2.838654	1.305962	-0.134275
H	-2.579066	1.581685	0.894158
H	-2.276043	1.987488	-0.778871
H	-3.940499	1.577217	-0.261319
Br	0.169039	-0.056134	-0.070546
C	2.756153	0.061169	0.040527
O	3.010678	-1.068942	-0.296454
O	2.816853	1.190232	0.479536

Optimization step 16

C	-2.601462	-0.064053	-0.394473
H	-2.464910	-0.327243	-1.449655
C	-3.020197	-1.147403	0.494948
H	-2.476327	-2.066496	0.266210
H	-2.781863	-0.897623	1.536201
H	-4.130048	-1.421146	0.494109
C	-2.929535	1.340463	-0.126708
H	-2.672222	1.601104	0.908173
H	-2.342874	2.009700	-0.763927
H	-4.020653	1.661793	-0.255963
Br	0.183641	-0.098968	-0.062777
C	2.915129	0.080828	0.083034
O	3.161308	-1.023076	-0.294378
O	2.873423	1.228675	0.434535

Optimization step 17

C	-2.827576	-0.034062	-0.378605
H	-2.733997	-0.272115	-1.443631
C	-3.228371	-1.155562	0.530266
H	-2.747625	-2.116244	0.263724
H	-2.980498	-0.954214	1.596577
H	-4.326387	-1.347429	0.519661
C	-3.110103	1.393104	-0.046542
H	-2.848489	1.653928	1.004126
H	-2.562747	2.095576	-0.704790
H	-4.189150	1.652269	-0.150788
Br	0.208108	-0.139491	-0.131402
C	3.136895	0.134218	0.099884
O	3.413892	-0.936067	-0.173478
O	2.996111	1.204095	0.458997

Optimization step 18

C	-2.905898	-0.021108	-0.371697
H	-2.807841	-0.261257	-1.435111

C	-3.290265	-1.134924	0.535725
H	-2.806346	-2.084359	0.249623
H	-3.022821	-0.924784	1.589036
H	-4.389678	-1.332079	0.540542
C	-3.169518	1.401593	-0.029472
H	-2.893679	1.638498	1.016345
H	-2.617151	2.093183	-0.686925
H	-4.248031	1.678803	-0.122613
Br	0.168266	-0.150713	-0.138296
C	3.274598	0.132881	0.106079
O	3.510494	-0.983004	-0.140818
O	3.169848	1.257539	0.421526

Optimization step 19

C	-3.016351	-0.001571	-0.420147
H	-2.843176	-0.235444	-1.476119
C	-3.392213	-1.119942	0.480610
H	-2.879718	-2.054469	0.198007
H	-3.127988	-0.901587	1.530657
H	-4.486504	-1.343013	0.481983
C	-3.245768	1.418808	-0.051162
H	-2.974288	1.614425	1.001568
H	-2.651772	2.103091	-0.677129
H	-4.310891	1.740000	-0.153573
Br	0.192485	-0.172255	-0.080507
C	3.370126	0.146403	0.094504
O	3.548109	-0.966487	-0.201781
O	3.232217	1.271954	0.362973

Optimization step 20

C	-3.060557	0.003439	-0.422537
H	-2.881567	-0.233644	-1.476839
C	-3.427628	-1.110274	0.487769
H	-2.913751	-2.044548	0.208656
H	-3.153772	-0.883267	1.533164
H	-4.520858	-1.335688	0.499501
C	-3.275997	1.425317	-0.050740
H	-2.995728	1.614626	1.000472
H	-2.677352	2.104684	-0.677614
H	-4.338112	1.756164	-0.146921
Br	0.195617	-0.183619	-0.084403
C	3.408950	0.159715	0.088760
O	3.588445	-0.954798	-0.193526
O	3.257298	1.277194	0.367798

Optimization step 21

C	-3.173093	0.001636	-0.472949
H	-2.968310	-0.223485	-1.525503
C	-3.524446	-1.121179	0.458595
H	-3.005182	-2.056058	0.185560
H	-3.241935	-0.875088	1.497852
H	-4.606090	-1.327873	0.456609
C	-3.399230	1.422584	-0.049898
H	-3.117486	1.565813	1.008258
H	-2.803673	2.129349	-0.653125
H	-4.457351	1.708987	-0.145636
Br	0.213756	-0.169296	-0.046415
C	3.488563	0.140964	0.073377
O	3.630253	-0.970772	-0.238078
O	3.415720	1.263232	0.331298

Optimization step 22

C	-3.234843	-0.002665	-0.454474
H	-3.032414	-0.228652	-1.505158
C	-3.556318	-1.126466	0.479598
H	-3.047481	-2.061567	0.191240
H	-3.250693	-0.883609	1.513399
H	-4.642949	-1.342097	0.511093
C	-3.437517	1.419810	-0.041723
H	-3.132868	1.573123	1.009384
H	-2.852977	2.117260	-0.665260
H	-4.499743	1.726015	-0.114601
Br	0.218389	-0.167866	-0.070126
C	3.528392	0.144648	0.068646
O	3.694613	-0.972233	-0.205716
O	3.432541	1.267594	0.355968

Optimization step 23

C	-3.349034	0.006304	-0.466405
H	-3.124481	-0.196986	-1.516561

C	-3.638516	-1.137403	0.446028
H	-3.138841	-2.062595	0.116845
H	-3.302665	-0.919500	1.475868
H	-4.725774	-1.361993	0.509938
C	-3.523082	1.419126	-0.022392
H	-3.196245	1.549526	1.025137
H	-2.944926	2.120809	-0.645600
H	-4.584177	1.748427	-0.064715
Br	0.238294	-0.164424	-0.056482
C	3.601138	0.139750	0.061371
O	3.749525	-0.984661	-0.195700
O	3.517198	1.273472	0.316244

Optimization step 24

C	-3.442335	-0.000184	-0.471012
H	-3.212328	-0.190982	-1.523544
C	-3.695048	-1.155589	0.434809
H	-3.214364	-2.077492	0.069772
H	-3.316033	-0.957002	1.454012
H	-4.778720	-1.381332	0.546892
C	-3.600749	1.407826	-0.010896
H	-3.238217	1.535317	1.025557
H	-3.047389	2.113925	-0.651039
H	-4.662178	1.741132	-0.009795
Br	0.261516	-0.146244	-0.053859
C	3.640431	0.127400	0.049301
O	3.778696	-1.004645	-0.187570
O	3.584101	1.261927	0.307570

Optimization step 25

C	-3.582831	0.004280	-0.483880
H	-3.347293	-0.161936	-1.539768
C	-3.794805	-1.173546	0.401456
H	-3.361016	-2.092261	-0.025062
H	-3.344288	-1.017909	1.399786
H	-4.871264	-1.385250	0.586203
C	-3.714072	1.402534	0.010723
H	-3.289335	1.516319	1.025700
H	-3.206037	2.122511	-0.650863
H	-4.773173	1.734756	0.087743
Br	0.301426	-0.135118	-0.041104
C	3.702474	0.117609	0.039852
O	3.823495	-1.020298	-0.175320
O	3.673743	1.258751	0.268569

Optimization step 26

C	-3.597155	0.006831	-0.483431
H	-3.372635	-0.153197	-1.542554
C	-3.805921	-1.175881	0.396322
H	-3.396654	-2.096925	-0.048367
H	-3.328726	-1.036142	1.384660
H	-4.880586	-1.371066	0.605619
C	-3.723754	1.401500	0.022439
H	-3.272545	1.512046	1.026333
H	-3.238195	2.128474	-0.648050
H	-4.782533	1.724667	0.130155
Br	0.311106	-0.134885	-0.045021
C	3.701607	0.117531	0.045025
O	3.822052	-1.021856	-0.160019
O	3.669760	1.261009	0.258246

Optimization step 27

C	-3.641642	0.012703	-0.476177
H	-3.498663	-0.113249	-1.553281
C	-3.833890	-1.193015	0.378059
H	-3.574739	-2.121479	-0.152899
H	-3.211417	-1.141792	1.291930
H	-4.881480	-1.293474	0.730710
C	-3.753679	1.385549	0.092315
H	-3.155168	1.488637	1.017117
H	-3.414307	2.156348	-0.616617
H	-4.797124	1.635835	0.376008
Br	0.358529	-0.121965	-0.077887
C	3.668956	0.108669	0.073346
O	3.788326	-1.039700	-0.063349
O	3.629913	1.261516	0.216827

Optimization step 28

C	-3.710385	0.019161	-0.475769
H	-3.634440	-0.075515	-1.562775

C	-3.876276	-1.205685	0.357670
H	-3.765724	-2.127777	-0.231543
H	-3.129005	-1.237873	1.175045
H	-4.868214	-1.237272	0.853315
C	-3.793897	1.371825	0.144724
H	-3.067426	1.477387	0.973728
H	-3.595535	2.175743	-0.579724
H	-4.792470	1.560161	0.589911
Br	0.399049	-0.110577	-0.097435
C	3.658659	0.099517	0.088107
O	3.776742	-1.054274	0.003675
O	3.625444	1.257577	0.184308

Optimization step 29

C	-3.678149	0.012843	-0.485036
H	-3.560338	-0.112500	-1.565397
C	-3.844666	-1.190246	0.377985
H	-3.645009	-2.125090	-0.167948
H	-3.159229	-1.157368	1.247278
H	-4.868406	-1.264269	0.801724
C	-3.757922	1.385594	0.088803
H	-3.089279	1.494087	0.964703
H	-3.477519	2.159019	-0.642771
H	-4.778233	1.629341	0.453700
Br	0.363992	-0.124766	-0.072094
C	3.672549	0.110217	0.075804
O	3.806757	-1.036281	-0.075474
O	3.629170	1.265425	0.211306

Optimization step 30

C	-3.820181	0.019553	-0.490637
H	-3.821248	-0.073971	-1.580605
C	-3.887946	-1.198456	0.363599
H	-3.940436	-2.126869	-0.225779
H	-2.995457	-1.274789	1.018710
H	-4.764487	-1.182323	1.046009
C	-3.789264	1.370972	0.134406
H	-2.897643	1.488218	0.785331
H	-3.756182	2.179652	-0.611305
H	-4.667761	1.551775	0.791127
Br	0.399251	-0.116451	-0.085648
C	3.679786	0.102855	0.088225
O	3.837163	-1.047264	-0.028003
O	3.634721	1.265330	0.178081

Optimization step 31

C	-3.993115	0.028989	-0.488564
H	-4.096303	-0.039679	-1.575470
C	-3.948876	-1.203550	0.345629
H	-4.235872	-2.109631	-0.210724
H	-2.927094	-1.379004	0.745338
H	-4.609958	-1.127130	1.234629
C	-3.830602	1.360890	0.157753
H	-2.793310	1.497236	0.535279
H	-4.030453	2.195604	-0.531141
H	-4.488761	1.481964	1.044970
Br	0.432981	-0.111153	-0.084481
C	3.711917	0.097386	0.084295
O	3.880906	-1.054637	-0.012373
O	3.668026	1.263225	0.152283

Optimization step 32

C	-4.060523	0.047433	-0.455521
H	-4.235450	-0.010067	-1.533599
C	-3.921905	-1.199364	0.345099
H	-4.322216	-2.087799	-0.169833
H	-2.857535	-1.415973	0.581659
H	-4.434796	-1.121947	1.326384
C	-3.771255	1.368348	0.168182
H	-2.683784	1.489912	0.372521
H	-4.068441	2.215619	-0.470302
H	-4.271742	1.490247	1.151698
Br	0.413054	-0.120281	-0.096373
C	3.728201	0.102500	0.082742
O	3.890277	-1.050942	0.006695
O	3.680968	1.267982	0.152244

Optimization step 33

C	-4.132641	0.107944	-0.343910
H	-4.472412	0.043458	-1.382638

C	-3.791612	-1.153299	0.369085
H	-4.386628	-2.012429	0.016159
H	-2.720990	-1.425210	0.244131
H	-3.949509	-1.062824	1.460207
C	-3.510779	1.378994	0.119708
H	-2.418359	1.394999	-0.098334
H	-3.947191	2.266391	-0.369609
H	-3.603287	1.512111	1.212731
Br	0.334699	-0.155817	-0.107869
C	3.729752	0.124121	0.066355
O	3.852509	-1.028134	-0.025056
O	3.649441	1.276953	0.203223

Optimization step 34

C	-4.055272	0.071212	-0.389364
H	-4.350855	-0.005844	-1.441369
C	-3.735245	-1.197403	0.337203
H	-4.235567	-2.076893	-0.102591
H	-2.643087	-1.403918	0.340727
H	-4.027628	-1.131966	1.397008
C	-3.503154	1.373025	0.096966
H	-2.394852	1.401383	0.008882
H	-3.903883	2.237223	-0.460684
H	-3.727147	1.521542	1.163054
Br	0.338090	-0.108112	-0.059090
C	3.643616	0.075593	0.051395
O	3.752714	-1.070806	-0.103405
O	3.666063	1.234284	0.176644

Optimization step 35

C	-4.131448	0.066997	-0.377348
H	-4.492697	0.021704	-1.411406
C	-3.802045	-1.211014	0.323923
H	-4.415739	-2.060115	-0.017680
H	-2.737546	-1.487443	0.154952
H	-3.918820	-1.112475	1.418296
C	-3.600451	1.365933	0.134926
H	-2.517900	1.469119	-0.099543
H	-4.112907	2.238275	-0.301950
H	-3.677887	1.426627	1.235198
Br	0.385120	-0.106736	-0.078424
C	3.663150	0.084128	0.057512
O	3.800579	-1.069767	-0.042023
O	3.651804	1.245245	0.158637

Optimization step 36

C	-4.025428	0.061048	-0.401959
H	-4.307276	-0.003043	-1.460166
C	-3.774324	-1.203862	0.345691
H	-4.281375	-2.069496	-0.106770
H	-2.686528	-1.428155	0.376794
H	-4.095970	-1.126977	1.404018
C	-3.576821	1.376959	0.134183
H	-2.469483	1.452423	0.119481
H	-3.980396	2.225172	-0.439856
H	-3.871493	1.506388	1.195524
Br	0.367194	-0.114207	-0.086494
C	3.653637	0.090782	0.066569
O	3.770325	-1.063068	-0.038610
O	3.626967	1.249487	0.172529

Optimization step 37

C	-4.061361	0.066689	-0.390811
H	-4.368167	0.004429	-1.441226
C	-3.774703	-1.198361	0.343931
H	-4.320616	-2.059265	-0.072645
H	-2.690872	-1.442084	0.308386
H	-4.029191	-1.114133	1.417638
C	-3.564891	1.372359	0.129429
H	-2.459348	1.441555	0.038086
H	-3.996486	2.230408	-0.409696
H	-3.786755	1.493516	1.206819
Br	0.364747	-0.115756	-0.084303
C	3.663384	0.091319	0.063546
O	3.782806	-1.061396	-0.045533
O	3.631033	1.249520	0.173866

Optimization step 38

C	-4.059930	0.067581	-0.384816
H	-4.379896	0.005767	-1.431150

C	-3.758426	-1.199030	0.342526
H	-4.315174	-2.058732	-0.062775
H	-2.676042	-1.445070	0.284099
H	-3.990241	-1.114162	1.420400
C	-3.544769	1.370532	0.125582
H	-2.440618	1.432252	0.012195
H	-3.981168	2.231776	-0.405091
H	-3.745466	1.491867	1.206014
Br	0.357908	-0.113082	-0.082288
C	3.658407	0.088046	0.061057
O	3.774135	-1.064306	-0.051701
O	3.629633	1.245731	0.175490

Optimization step 39

C	-4.058883	0.066429	-0.383012
H	-4.385371	0.005850	-1.427476
C	-3.752014	-1.201040	0.340370
H	-4.311428	-2.060492	-0.061796
H	-2.669915	-1.445731	0.273551
H	-3.975655	-1.117458	1.420144
C	-3.540720	1.369046	0.124973
H	-2.436930	1.428614	0.007834
H	-3.977983	2.230679	-0.404311
H	-3.737328	1.490873	1.206224
Br	0.355198	-0.110476	-0.080990
C	3.658378	0.085793	0.059586
O	3.772219	-1.066759	-0.053355
O	3.630548	1.243381	0.174478

Optimization step 40

C	-4.058736	0.060938	-0.381043
H	-4.401246	0.004475	-1.420776
C	-3.738740	-1.208734	0.332202
H	-4.299303	-2.068883	-0.066685
H	-2.656212	-1.446556	0.250956
H	-3.948603	-1.131186	1.415610
C	-3.540681	1.363830	0.125918
H	-2.437295	1.423300	0.006510
H	-3.980477	2.225456	-0.400965
H	-3.733748	1.484907	1.208485
Br	0.350246	-0.101507	-0.076864
C	3.661223	0.078572	0.055172
O	3.770951	-1.074790	-0.055334
O	3.636534	1.236488	0.168284

Optimization step 41

C	-4.060251	0.053932	-0.379952
H	-4.419842	0.001176	-1.414163
C	-3.724694	-1.217459	0.322690
H	-4.285651	-2.078912	-0.072696
H	-2.641794	-1.447531	0.226434
H	-3.920272	-1.146113	1.409422
C	-3.543110	1.357375	0.126326
H	-2.440436	1.418887	0.002621
H	-3.987656	2.218766	-0.396718
H	-3.731066	1.476333	1.210393
Br	0.346137	-0.090877	-0.071246
C	3.663885	0.070156	0.049011
O	3.768721	-1.084077	-0.058390
O	3.643396	1.228333	0.160876

Optimization step 42

C	-4.065541	0.043743	-0.382070
H	-4.441806	-0.006520	-1.410372
C	-3.711952	-1.227875	0.311025
H	-4.267179	-2.093112	-0.084076
H	-2.627399	-1.445896	0.205937
H	-3.899527	-1.164194	1.399898
C	-3.555379	1.349330	0.125601
H	-2.453341	1.418039	0.001400
H	-4.006275	2.209001	-0.394691
H	-3.742274	1.465354	1.210506
Br	0.343429	-0.077851	-0.062632
C	3.670181	0.060262	0.039689
O	3.768643	-1.094996	-0.063618
O	3.655597	1.218663	0.150874

Optimization step 43

C	-4.078755	0.018447	-0.387315
H	-4.494721	-0.032328	-1.400014

C	-3.681070	-1.248950	0.288809
H	-4.223826	-2.125139	-0.099230
H	-2.593756	-1.439217	0.161023
H	-3.846218	-1.196838	1.382504
C	-3.582164	1.331270	0.114819
H	-2.482203	1.415371	-0.015305
H	-4.049141	2.184423	-0.401744
H	-3.762088	1.446435	1.201733
Br	0.336418	-0.049087	-0.040275
C	3.685983	0.039080	0.011596
O	3.768920	-1.118436	-0.081790
O	3.682748	1.196719	0.133440

Optimization step 44

C	-4.082633	0.036916	-0.384130
H	-4.471397	-0.014702	-1.407452
C	-3.709967	-1.231831	0.303885
H	-4.256300	-2.103640	-0.089505
H	-2.623330	-1.438075	0.195186
H	-3.893693	-1.173787	1.394084
C	-3.574879	1.344333	0.120971
H	-2.474154	1.421191	-0.010374
H	-4.033934	2.202871	-0.394449
H	-3.754056	1.459269	1.207471
Br	0.341351	-0.071834	-0.054678
C	3.686599	0.056355	0.032647
O	3.782080	-1.099028	-0.072050
O	3.673528	1.214833	0.144367

Optimization step 45

C	-4.073429	0.055013	-0.385361
H	-4.425053	-0.003967	-1.421518
C	-3.740992	-1.211350	0.327872
H	-4.290962	-2.077597	-0.072606
H	-2.655868	-1.439590	0.251898
H	-3.956033	-1.137392	1.410681
C	-3.564030	1.361043	0.122081
H	-2.461180	1.431537	0.002901
H	-4.010762	2.220114	-0.403406
H	-3.757225	1.480532	1.204787
Br	0.350904	-0.098134	-0.070483
C	3.676466	0.076838	0.052190
O	3.784153	-1.076036	-0.064123
O	3.651768	1.235010	0.163307

Optimization step 46

C	-4.089299	0.016216	-0.391029
H	-4.506113	-0.044831	-1.402358
C	-3.684828	-1.243703	0.295309
H	-4.217045	-2.126053	-0.093629
H	-2.595023	-1.428378	0.180571
H	-3.862963	-1.187757	1.386178
C	-3.596820	1.333590	0.103379
H	-2.497332	1.423682	-0.029127
H	-4.068014	2.182265	-0.417251
H	-3.776225	1.453651	1.188887
Br	0.337762	-0.053602	-0.036092
C	3.696021	0.043575	0.011552
O	3.779089	-1.111998	-0.097763
O	3.689736	1.200175	0.139596

Optimization step 47

C	-4.077919	0.037170	-0.390026
H	-4.456224	-0.021604	-1.416673
C	-3.717757	-1.227130	0.313335
H	-4.256606	-2.100096	-0.087405
H	-2.629484	-1.435064	0.227539
H	-3.924861	-1.162357	1.398346
C	-3.583784	1.349299	0.116800
H	-2.481746	1.431360	-0.000213
H	-4.039881	2.202723	-0.409462
H	-3.779563	1.467032	1.199432
Br	0.347869	-0.077975	-0.056210
C	3.682378	0.061794	0.037409
O	3.779723	-1.092161	-0.077917
O	3.667206	1.219703	0.151752

Optimization step 48

C	-4.071030	0.049599	-0.386580
H	-4.431021	-0.009247	-1.419909

C	-3.732269	-1.216278	0.324701
H	-4.279327	-2.083965	-0.076471
H	-2.646263	-1.438798	0.246608
H	-3.946028	-1.144273	1.407851
C	-3.567321	1.357911	0.120828
H	-2.464341	1.430543	0.004873
H	-4.015201	2.214785	-0.406986
H	-3.764099	1.477612	1.202914
Br	0.350546	-0.092062	-0.067194
C	3.674110	0.072214	0.048930
O	3.779016	-1.080931	-0.067895
O	3.653014	1.230285	0.161101

Optimization step 49

C	-4.051539	0.073662	-0.379552
H	-4.380654	0.005044	-1.422837
C	-3.763348	-1.187878	0.362028
H	-4.327796	-2.046139	-0.035189
H	-2.683228	-1.441974	0.305762
H	-3.995159	-1.088676	1.438917
C	-3.534015	1.379898	0.119963
H	-2.428118	1.428816	0.015379
H	-3.958819	2.237323	-0.425346
H	-3.743292	1.514703	1.197580
Br	0.361401	-0.126884	-0.093664
C	3.647483	0.099993	0.069075
O	3.771273	-1.051354	-0.047668
O	3.613296	1.256077	0.194530

Optimization step 50

C	-4.067942	0.046631	-0.382584
H	-4.444121	-0.007743	-1.410572
C	-3.720618	-1.221976	0.319546
H	-4.273519	-2.088234	-0.076488
H	-2.635660	-1.442037	0.224406
H	-3.917842	-1.153046	1.406301
C	-3.561085	1.354201	0.123635
H	-2.458213	1.422504	0.004818
H	-4.008817	2.211924	-0.402459
H	-3.754098	1.473687	1.206928
Br	0.348003	-0.085484	-0.066900
C	3.669278	0.066748	0.046851
O	3.771775	-1.087374	-0.064562
O	3.652522	1.225033	0.157546

Optimization step 51

C	-4.082905	0.027972	-0.389869
H	-4.479872	-0.030900	-1.409384
C	-3.702066	-1.235080	0.304502
H	-4.238800	-2.111895	-0.090659
H	-2.613132	-1.432574	0.203821
H	-3.894412	-1.175026	1.392624
C	-3.588595	1.342017	0.111677
H	-2.487700	1.427086	-0.013008
H	-4.051476	2.193791	-0.411308
H	-3.776161	1.460306	1.195870
Br	0.342926	-0.066637	-0.047414
C	3.688096	0.053169	0.026512
O	3.779174	-1.101518	-0.086337
O	3.677320	1.210647	0.145661

Optimization step 52

C	-4.088705	0.015744	-0.392375
H	-4.514989	-0.048668	-1.399197
C	-3.686379	-1.240817	0.301446
H	-4.213827	-2.124473	-0.090549
H	-2.595480	-1.423987	0.197258
H	-3.875225	-1.182252	1.390215
C	-3.602622	1.335577	0.102061
H	-2.501110	1.424340	-0.016552
H	-4.066841	2.180736	-0.429851
H	-3.796576	1.461555	1.184327
Br	0.342721	-0.056742	-0.038912
C	3.690650	0.046190	0.018897
O	3.775611	-1.108141	-0.100526
O	3.685780	1.202962	0.143787

Optimization step 53

C	-4.080383	0.035491	-0.386920
H	-4.478408	-0.025868	-1.405799

C	-3.711998	-1.226457	0.316557
H	-4.250572	-2.101683	-0.079525
H	-2.623613	-1.430732	0.225388
H	-3.913345	-1.160386	1.402662
C	-3.583361	1.349473	0.112415
H	-2.480543	1.426083	-0.001743
H	-4.035122	2.200785	-0.420686
H	-3.781597	1.475224	1.193845
Br	0.348508	-0.078042	-0.056837
C	3.679955	0.061789	0.040656
O	3.775967	-1.091375	-0.083242
O	3.666552	1.219658	0.155604

Optimization step 54

C	-4.061030	-0.054067	-0.436710
H	-4.543040	-0.190333	-1.410508
C	-3.605881	-1.259363	0.314257
H	-4.078801	-2.180393	-0.055849
H	-2.503194	-1.387172	0.243618
H	-3.824071	-1.166063	1.393902
C	-3.651618	1.319150	-0.024029
H	-2.555426	1.460062	-0.101519
H	-4.143029	2.099168	-0.625260
H	-3.894540	1.504455	1.038359
Br	0.346868	-0.013847	0.045454
C	3.680969	0.017030	-0.073163
O	3.704090	-1.129624	-0.262421
O	3.699293	1.155677	0.167950

Optimization step 55

C	-4.061566	0.011180	-0.397070
H	-4.482771	-0.072114	-1.405380
C	-3.662555	-1.237274	0.316969
H	-4.199041	-2.122152	-0.059117
H	-2.573154	-1.426938	0.207516
H	-3.845587	-1.154199	1.402768
C	-3.576241	1.343944	0.066332
H	-2.477419	1.432726	-0.068334
H	-4.052863	2.176735	-0.474145
H	-3.758417	1.481841	1.146540
Br	0.344118	-0.059923	-0.024528
C	3.659036	0.047913	0.008549
O	3.738888	-1.102059	-0.148878
O	3.660247	1.200414	0.166373

Optimization step 56

C	-4.085394	0.015157	-0.392965
H	-4.511055	-0.051590	-1.399988
C	-3.683456	-1.240409	0.303364
H	-4.211997	-2.124251	-0.086664
H	-2.592733	-1.424349	0.198531
H	-3.871599	-1.178816	1.391799
C	-3.599423	1.336628	0.097663
H	-2.498230	1.425442	-0.022907
H	-4.065176	2.180259	-0.435344
H	-3.791949	1.464085	1.179687
Br	0.342890	-0.057131	-0.037146
C	3.686793	0.046413	0.017616
O	3.771168	-1.107454	-0.106426
O	3.682639	1.202714	0.146541

Optimization step 57

C	-4.087909	0.011160	-0.399147
H	-4.510675	-0.063562	-1.406994
C	-3.689381	-1.239796	0.307429
H	-4.203739	-2.129211	-0.088691
H	-2.595468	-1.416230	0.222040
H	-3.896443	-1.177607	1.393119
C	-3.614919	1.338869	0.086669
H	-2.513189	1.433212	-0.015556
H	-4.075917	2.176160	-0.460458
H	-3.825773	1.475851	1.164730
Br	0.347220	-0.057962	-0.031408
C	3.691357	0.047387	0.016071
O	3.772126	-1.105182	-0.122407
O	3.687076	1.203224	0.150527

Optimization step 58

C	-4.100680	0.002923	-0.398289
H	-4.538174	-0.075974	-1.399715

C	-3.682623	-1.243850	0.306154
H	-4.189570	-2.140383	-0.085267
H	-2.588011	-1.406496	0.211012
H	-3.882300	-1.183481	1.393384
C	-3.627840	1.333948	0.079981
H	-2.526310	1.425210	-0.029972
H	-4.088676	2.170019	-0.469712
H	-3.833877	1.475254	1.158319
Br	0.346507	-0.050465	-0.026286
C	3.696185	0.042442	0.007438
O	3.775949	-1.110702	-0.130536
O	3.700163	1.196869	0.151818

Optimization step 59

C	-4.085876	0.008315	-0.397957
H	-4.513918	-0.069654	-1.403518
C	-3.679729	-1.240747	0.309132
H	-4.197755	-2.131446	-0.079859
H	-2.586777	-1.415769	0.212899
H	-3.876940	-1.173956	1.395650
C	-3.609113	1.338232	0.079674
H	-2.508154	1.429776	-0.034674
H	-4.074329	2.174262	-0.465988
H	-3.809898	1.476523	1.158669
Br	0.345922	-0.055630	-0.028085
C	3.684716	0.045634	0.011090
O	3.765148	-1.106594	-0.131230
O	3.684914	1.200181	0.154749

Optimization step 60

C	-4.087767	0.010429	-0.396860
H	-4.514874	-0.062101	-1.403033
C	-3.683288	-1.241541	0.304878
H	-4.201875	-2.130362	-0.087540
H	-2.590422	-1.417957	0.209188
H	-3.880591	-1.180081	1.392167
C	-3.607603	1.336151	0.087976
H	-2.505931	1.426112	-0.023174
H	-4.069617	2.175716	-0.454870
H	-3.810025	1.471803	1.167601
Br	0.344992	-0.054517	-0.031031
C	3.687339	0.044295	0.013299
O	3.768408	-1.108874	-0.120297
O	3.687409	1.199995	0.149048

F. References

(1) Zhang, W.; Lin, S. Electroreductive Carbofunctionalization of Alkenes with Alkyl Bromides via a Radical-Polar Crossover Mechanism. *J. Am. Chem. Soc.* **2020**, *142* (49), 20661–20670.

(2) Zhao, H.; McMillan, A. J.; Constantin, T.; Mykura, R. C.; Juliá, F.; Leonori, D. Merging Halogen-Atom Transfer (XAT) and Cobalt Catalysis to Override E2-Selectivity in the Elimination of Alkyl Halides: A Mild Route toward contra-Thermodynamic Olefins. *J. Am. Chem. Soc.* **2021**, *143* (36), 14806–14813.

(3) Bogonda, G.; Patil, D. V.; Kim, H. Y.; Oh, K. Visible-Light-Promoted Thiyl Radical Generation from Sodium Sulfinates: A Radical–Radical Coupling to Thioesters. *Org. Lett.* **2019**, *21* (10), 3774–3779.

(4) Sang, R.; Gestwicki, J. E. Radical Strategy to the Boron-to-Copper Transmetalation Problem: N-Alkylation with Alkylboronic Esters. *J. Am. Chem. Soc.* **2025**, *147* (26), 23259–23269.

(5) Tang, Z.-L.; Ouyang, X.-H.; Song, R.-J.; Li, J.-H. Decarboxylative C(sp^3)–N Cross-Coupling of Diacyl Peroxides with Nitrogen Nucleophiles. *Org. Lett.* **2021**, *23* (3), 1000–1004.

(6) Wang, Z.; Chen, Z.; Sun, J. Catalytic Enantioselective Intermolecular Desymmetrization of 3-Substituted Oxetanes. *Angew. Chem. Int. Ed.* **2013**, *52* (26), 6685–6688.

(7) Chen, R.; Yuan, H.; Wang, Y.; Chen, H.; Zhang, Y. Aerobic Electrochemical Csp3 – N Coupling between Aliphatic Carboxylic Acids and N-heterocycles. *Organometallics* **2023**, *42* (15), 1–5.

(8) Dow, N.; Cabré, A.; MacMillan, D. A general N-alkylation platform via copper metallaphotoredox and silyl radical activation of alkyl halides. *Chem* **2021**, *7* (7), 1827–1842.

(9) Lv, X.-Y.; Martin, R. Cu-Catalyzed C(sp^3) Amination of Unactivated Secondary Alkyl Iodides Promoted by Diaryliodonium Salts. *Org. Lett.* **2023**, *25* (20), 3750–3754.

(10) Carson, W. P., II; Tsymbal, A. V.; Pipal, R. W.; Edwards, G. A.; Martinelli, J. R.; Cabré, A.; MacMillan, D. W. C. Free-Radical Deoxygenative Amination of Alcohols via Copper Metallaphotoredox Catalysis. *J. Am. Chem. Soc.* **2024**, *146* (23), 15681–15687.

(11) Górski, B.; Barthelemy, A.; Douglas, J.; Juliá, F.; Leonori, D. Copper-catalysed amination of alkyl iodides enabled by halogen-atom transfer. *nat.catal* **2021**, *4* (7), 623–630.

(12) He, J.; Chen, G.; Zhang, B.; Li, Y.; Chen, J.; Xiao, W.; Liu, F.; Li, C. Catalytic Decarboxylative Radical Sulfonylation. *Chem* **2020**, *6* (5), 1149–1159.

(13) Melnykov, K.; Liashuk, O.; Smyrnov, O.; Lesyk, D.; Holota, Y.; Borysko, P.; Yakubovskyi, V.; Grygorenko, O. Lipophilicity effects of monofluorination at the tertiary aliphatic carbon as a function of α-substituent. *J. Fluor. Chem.* **2025**, *281*.

(14) Zheng, K.; Liang, C.; Chen, H.; Zhao, Y.; Wang, Z.; Cheng, J. I2 Catalyzed and TBHP/Ammonium-Promoted Conversion of Arylethanone to Nitriles via β-Scission of Iminyl Radicals. *Org. Lett.* **2024**, *26* (18), 3935–3939.

(15) Zhao, H.; Cuomo, V.; Rossi-Ashton, J.; Procter, D. Aryl sulfonium salt electron donor-acceptor complexes for halogen atom transfer: Isocyanides as tunable coupling partners. *Chem* **2024**, *10* (4).

(16) Liu, R. Y.; Bae, M.; Buchwald, S. L. Mechanistic Insight Facilitates Discovery of a Mild and Efficient Copper-Catalyzed Dehydration of Primary Amides to Nitriles Using Hydrosilanes. *J. Am. Chem. Soc.* **2018**, *140* (5), 1627–1631.

(17) Singh, M.; Dhote, P.; Johnson, D.; Figueroa-Lazú, S.; Elles, C.; Boskovic, Z. Photochemical Decarbonylation of Oxetanone and Azetidinone: Spectroscopy, Computational Models, and Synthetic Applications. *Angew. Chem. Int. Ed.* **2023**, *62* (3).
(18) Craven, P.; Aimon, A.; Dow, M.; Fleury-Bregeot, N.; Guilleux, R.; Morgentin, R.; Roche, D.; Kalliokoski, T.; Foster, R.; Marsden, S.; et al. Design, synthesis and decoration of molecular scaffolds for exploitation in the production of alkaloid-like libraries. *Bioorg. Med. Chem.* **2015**, *23* (11), 2629–2635.
(19) Escobar, R. A.; Johannes, J. W. Reductive Radical Conjugate Addition of Alkyl Electrophiles Catalyzed by a Cobalt/Iridium Photoredox System. *Org. Lett.* **2021**, *23* (15), 6046–6051.
(20) Gaussian 16, Revision C.01, M. J. Frisch, G. W. Trucks, H. B. Schlegel, G. E. Scuseria, M. A. Robb, J. R. Cheeseman, G. Scalmani, V. Barone, G. A. Petersson, H. Nakatsuji, X. Li, M. Caricato, A. V. Marenich, J. Bloino, B. G. Janesko, R. Gomperts, B. Mennucci, H. P. Hratchian, J. V. Ortiz, A. F. Izmaylov, J. L. Sonnenberg, D. Williams-Young, F. Ding, F. Lipparini, F. Egidi, J. Goings, B. Peng, A. Petrone, T. Henderson, D. Ranasinghe, V. G. Zakrzewski, J. Gao, N. Rega, G. Zheng, W. Liang, M. Hada, M. Ehara, K. Toyota, R. Fukuda, J. Hasegawa, M. Ishida, T. Nakajima, Y. Honda, O. Kitao, H. Nakai, T. Vreven, K. Throssell, J. A. Montgomery, Jr., J. E. Peralta, F. Ogliaro, M. J. Bearpark, J. J. Heyd, E. N. Brothers, K. N. Kudin, V. N. Staroverov, T. A. Keith, R. Kobayashi, J. Normand, K. Raghavachari, A. P. Rendell, J. C. Burant, S. S. Iyengar, J. Tomasi, M. Cossi, J. M. Millam, M. Klene, C. Adamo, R. Cammi, J. W. Ochterski, R. L. Martin, K. Morokuma, O. Farkas, J. B. Foresman, and D. J. Fox, Gaussian, Inc., Wallingford CT, **2016**.
(21) Becke, A. -D., Density-functional exchange-energy approximation with correct asymptotic behavior. *Phys. Rev. A.* **1988**, 38, 3098-3100.
(22) Becke, A. -D., Density-functional thermochemistry. III. The role of exact exchange. *J. Chem. Phys.* **1993**, 98, 5648-5652.
(23) Lee, C.; Yang, W.; Parr, R. -G., Development of the Colle-Salvetti correlation-energy formula into a functional of the electron density. *Phys. Rev. B*, **1988**, 37, 785-789.
(24) Grimme, S.; Antony, Jens.; Ehrlich, Stephan.; Krieg, Helge., A consistent and accurate ab initio parametrization of density functional dispersion correction (DFT-D) for the 94 elements H-Pu. *J. Chem. Phys.* **2010**, 132, 154104-154019.
(25) Grimme, S.; Ehrlich, S.; Goerigk, L., Effect of the damping function in dispersion corrected density functional theory. *J. Comput. Chem.* **2011**, 32, 1456-1465.
(26) Weigend, F., Accurate Coulomb-fitting basis sets for H to Rn. *Phys. Chem. Chem. Phys.* **2006**, 8, 1057-1065.
(27) Weigend, F.; Ahlrichs, R., Balanced basis sets of split valence, triple zeta valence and quadruple zeta valence quality for H to Rn: Design and assessment of accuracy. *Phys. Chem. Chem. Phys.* **2005**, 7, 3297-3305.
(28) Marenich, A. V.; Cramer, C. J.; Truhlar, D. G., Universal solvation model based on solute electron density and on a continuum model of the solvent defined by the bulk dielectric constant and atomic surface tensions. *J. Phys. Chem. B*, **2009,** *113* (18), 6378-6396.

G. NMR Spectra of Substrates

^{1}H NMR ($CDCl_3$, 400 MHz)

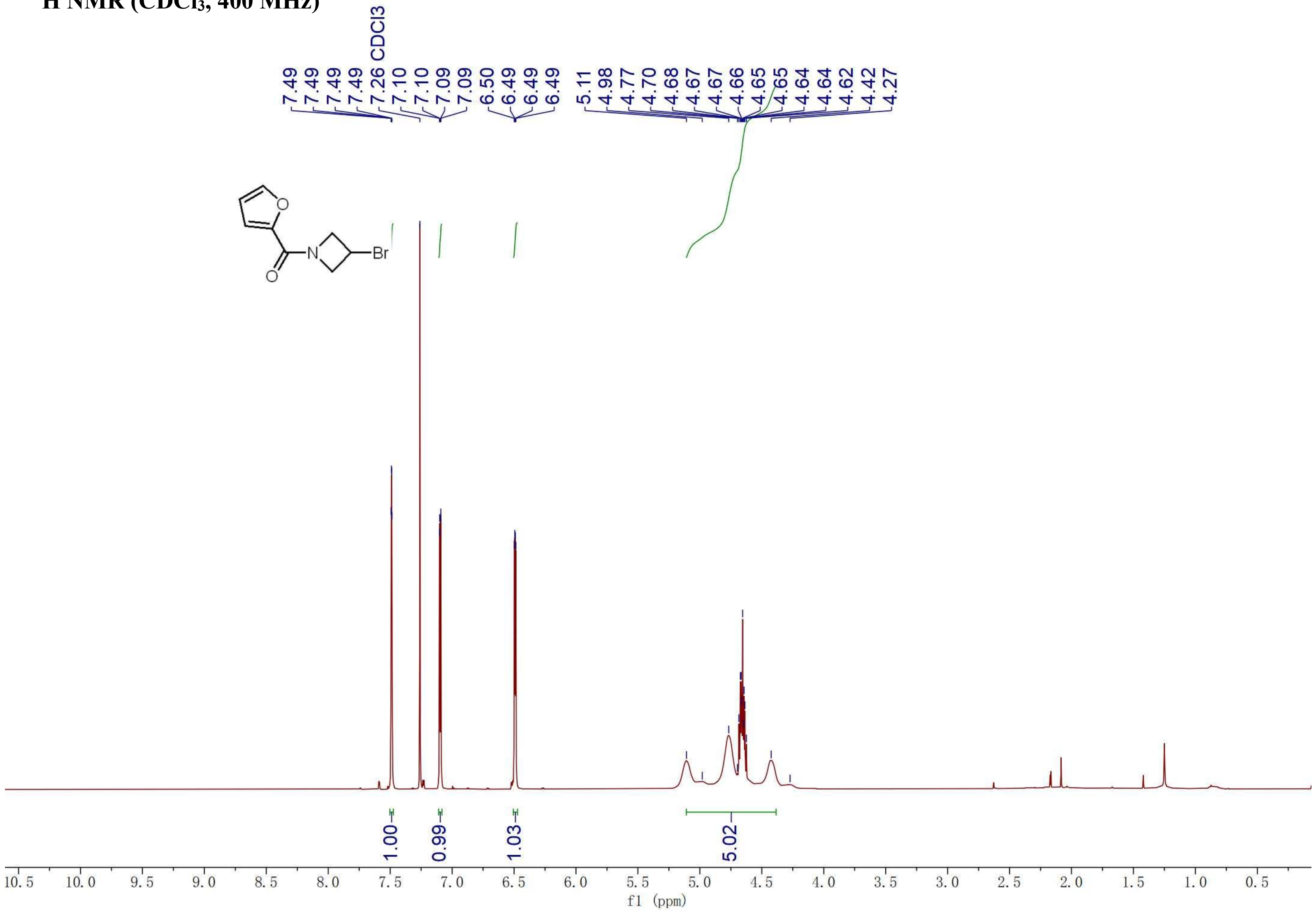

^{13}C NMR ($CDCl_3$, 101 MHz)

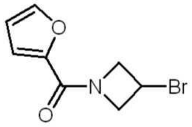

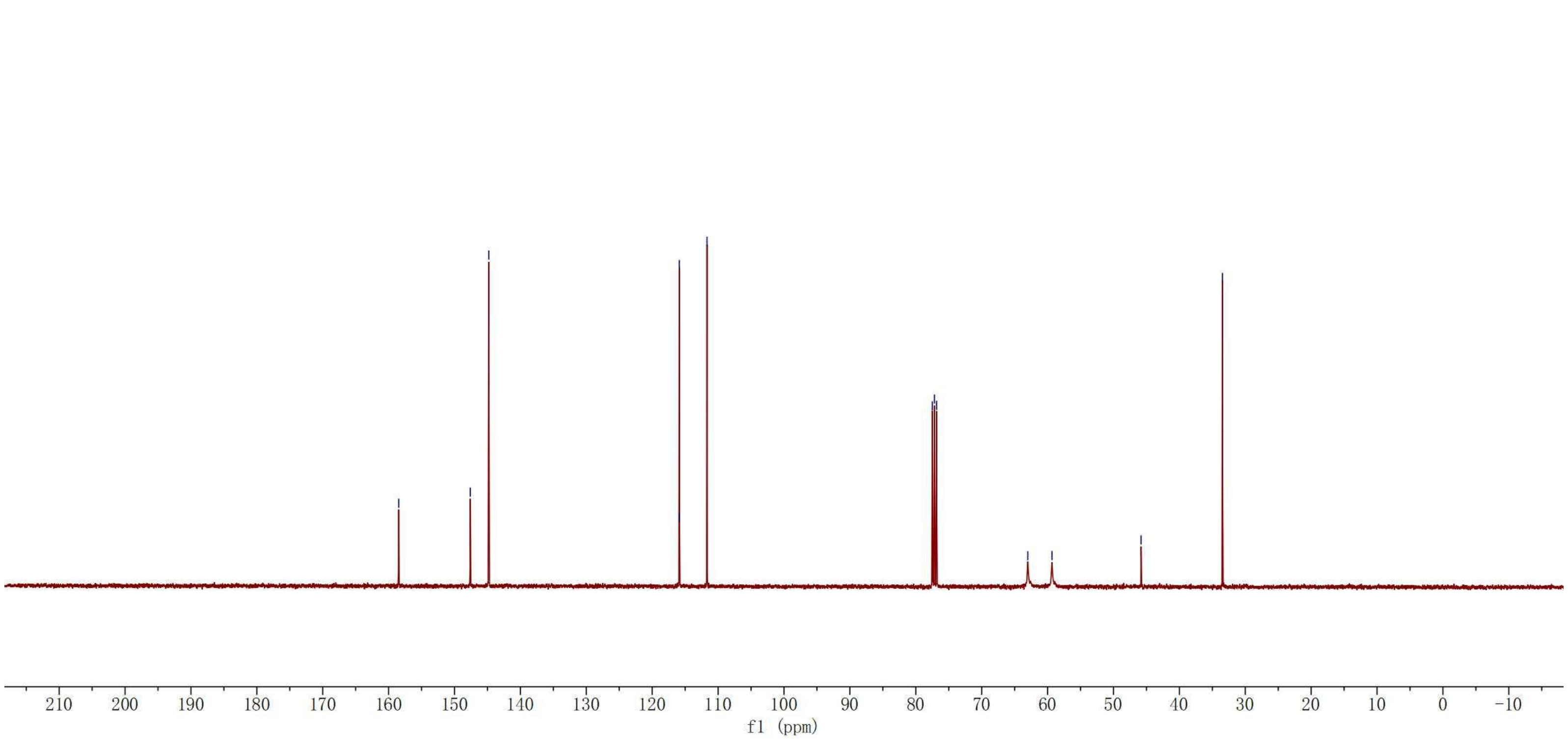

^{1}H NMR ($CDCl_3$, 400 MHz)

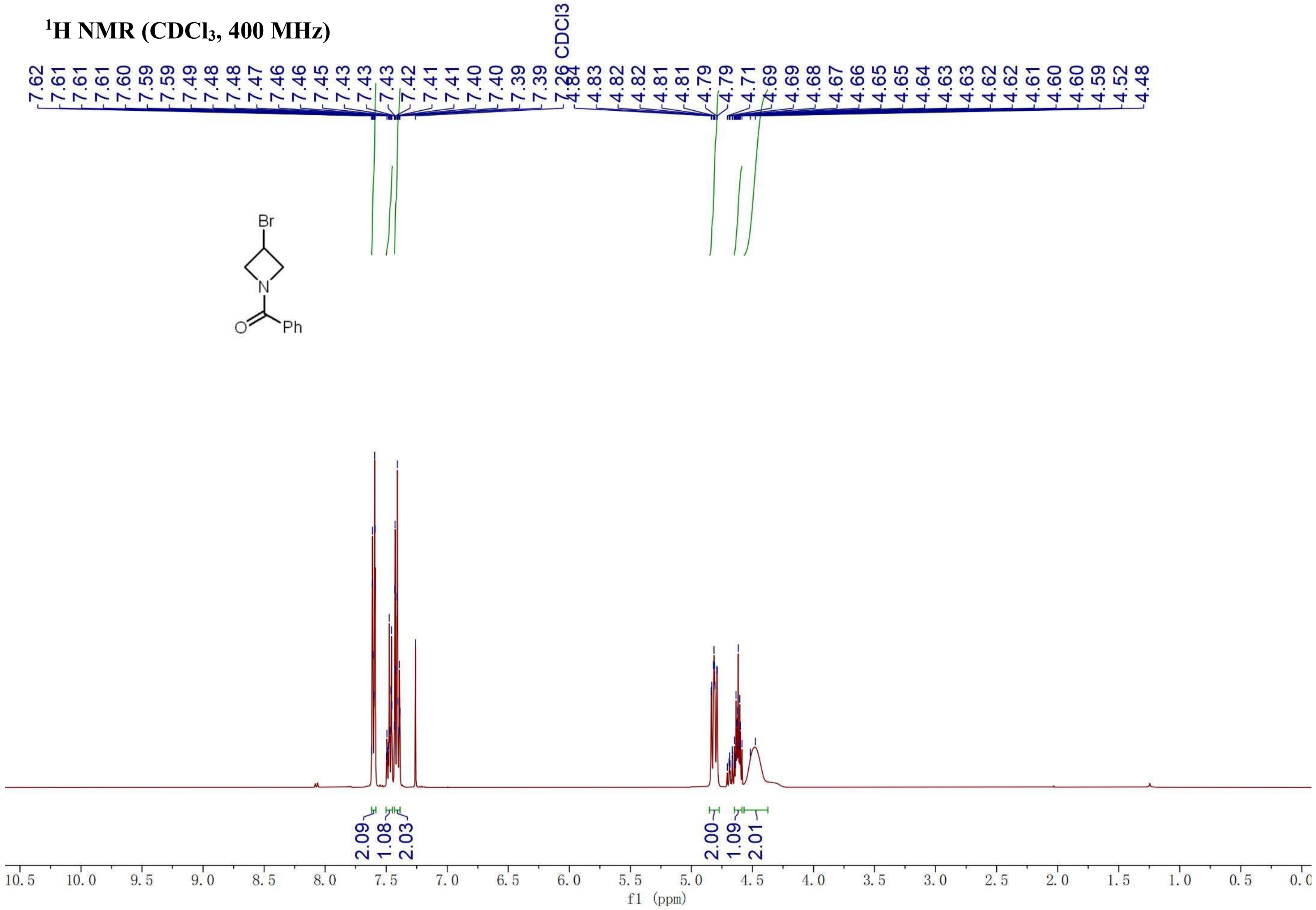

^{13}C NMR (CDCl$_3$, 101 MHz)

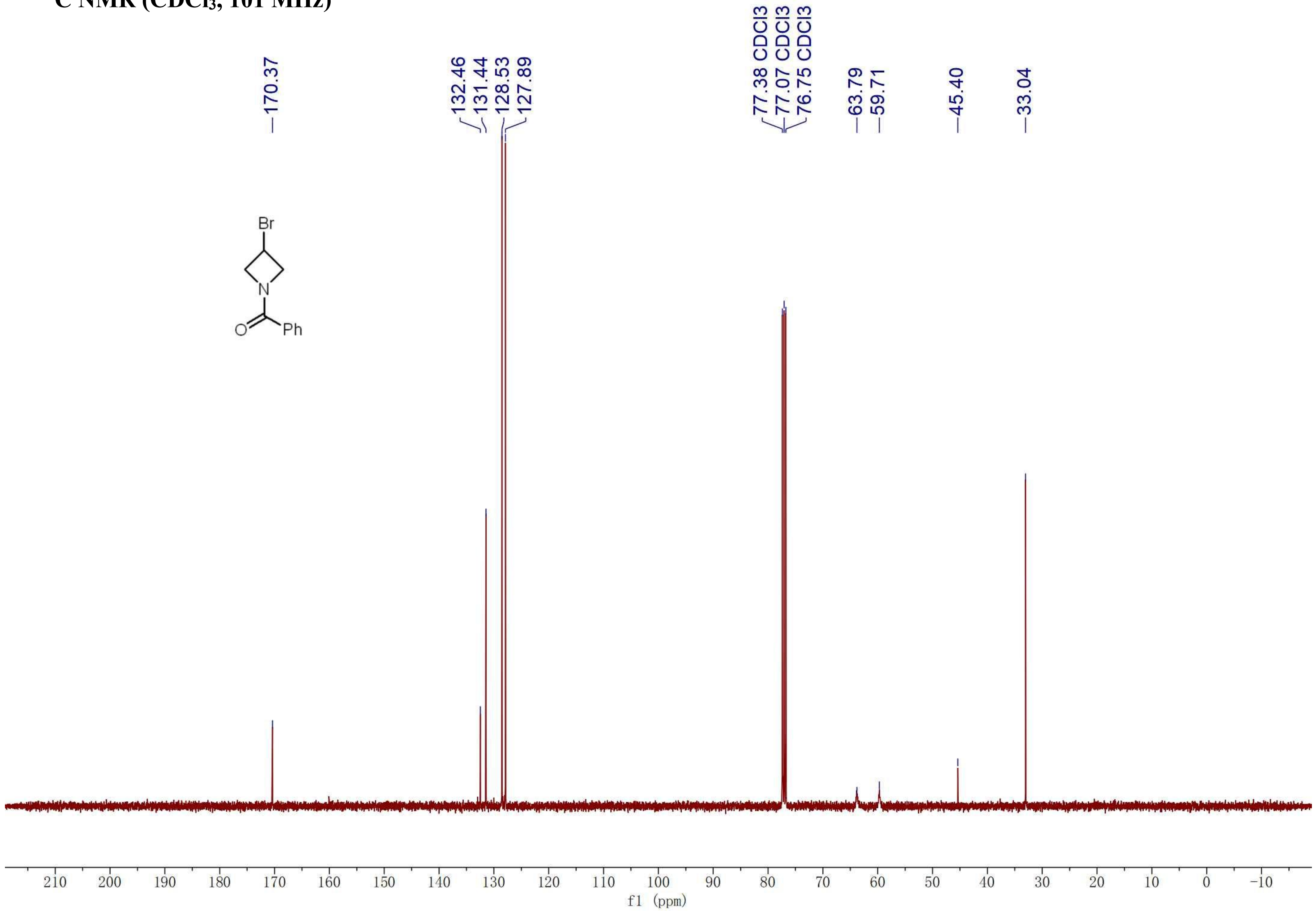

^{1}H NMR ($CDCl_3$, 400 MHz)

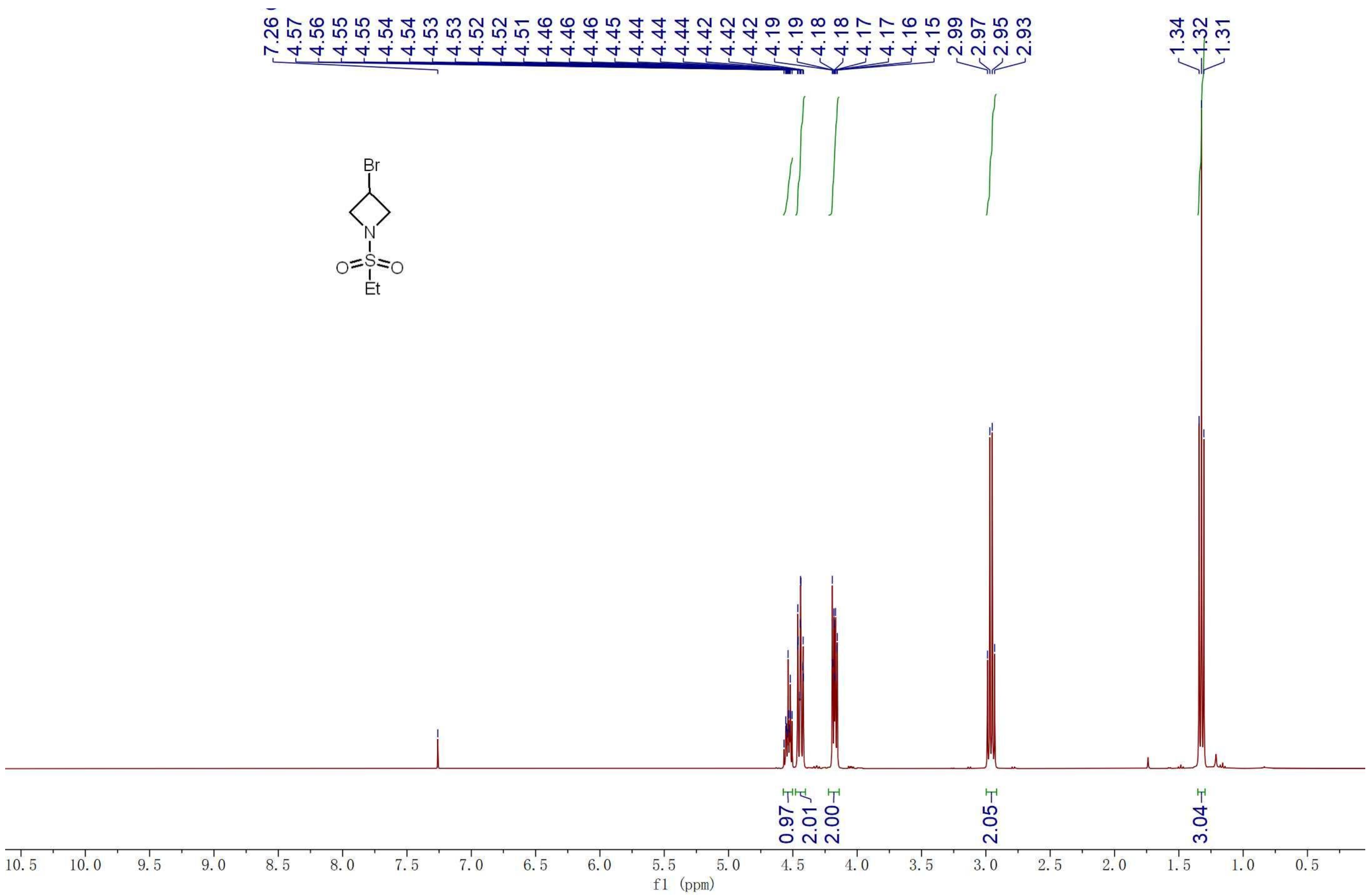

^{13}C NMR ($CDCl_3$, 101 MHz)

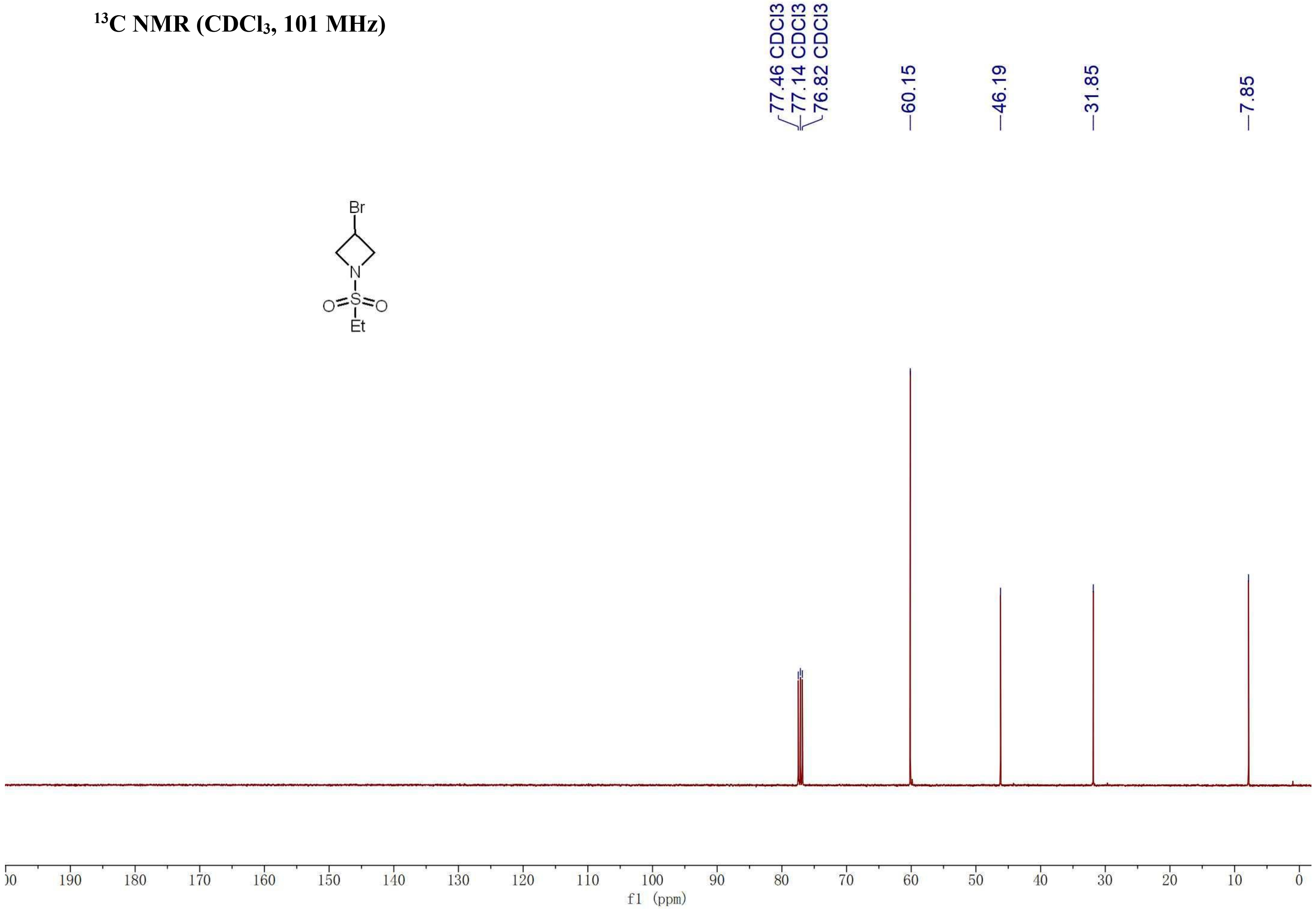

^{1}H NMR (CDCl₃, 400 MHz)

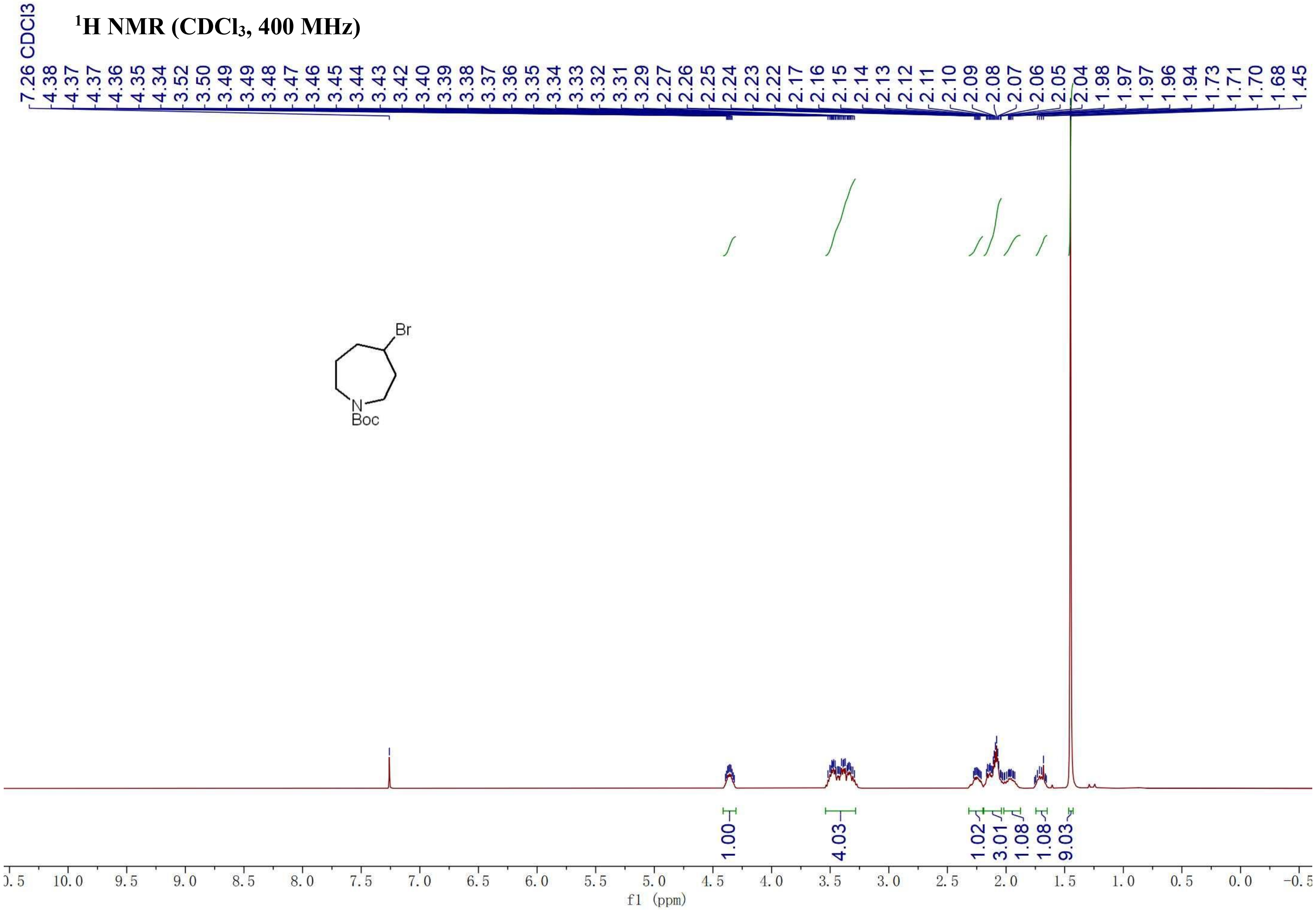

^{13}C NMR ($CDCl_3$, 101 MHz)

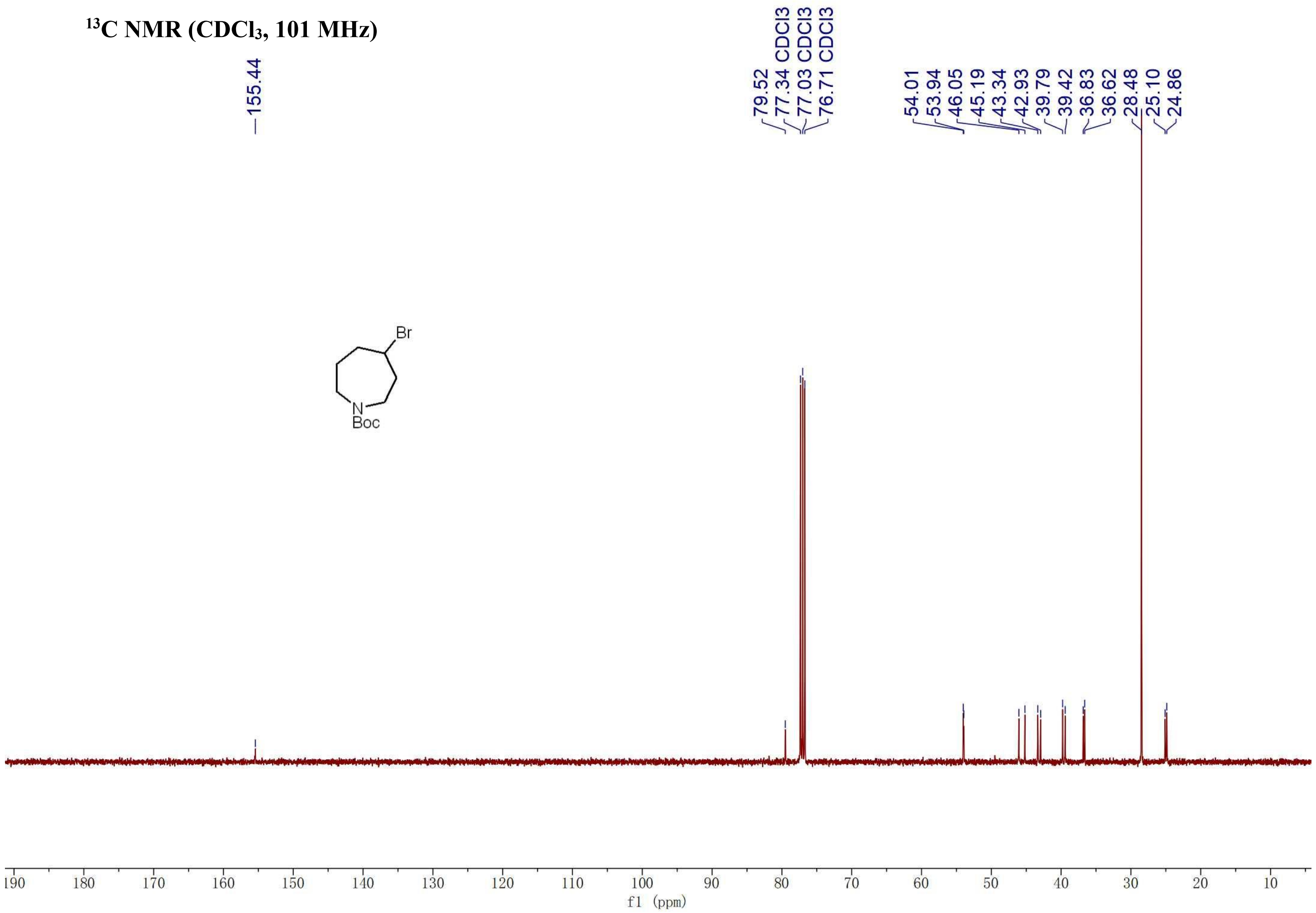

^{1}H NMR ($CDCl_3$, 400 MHz)

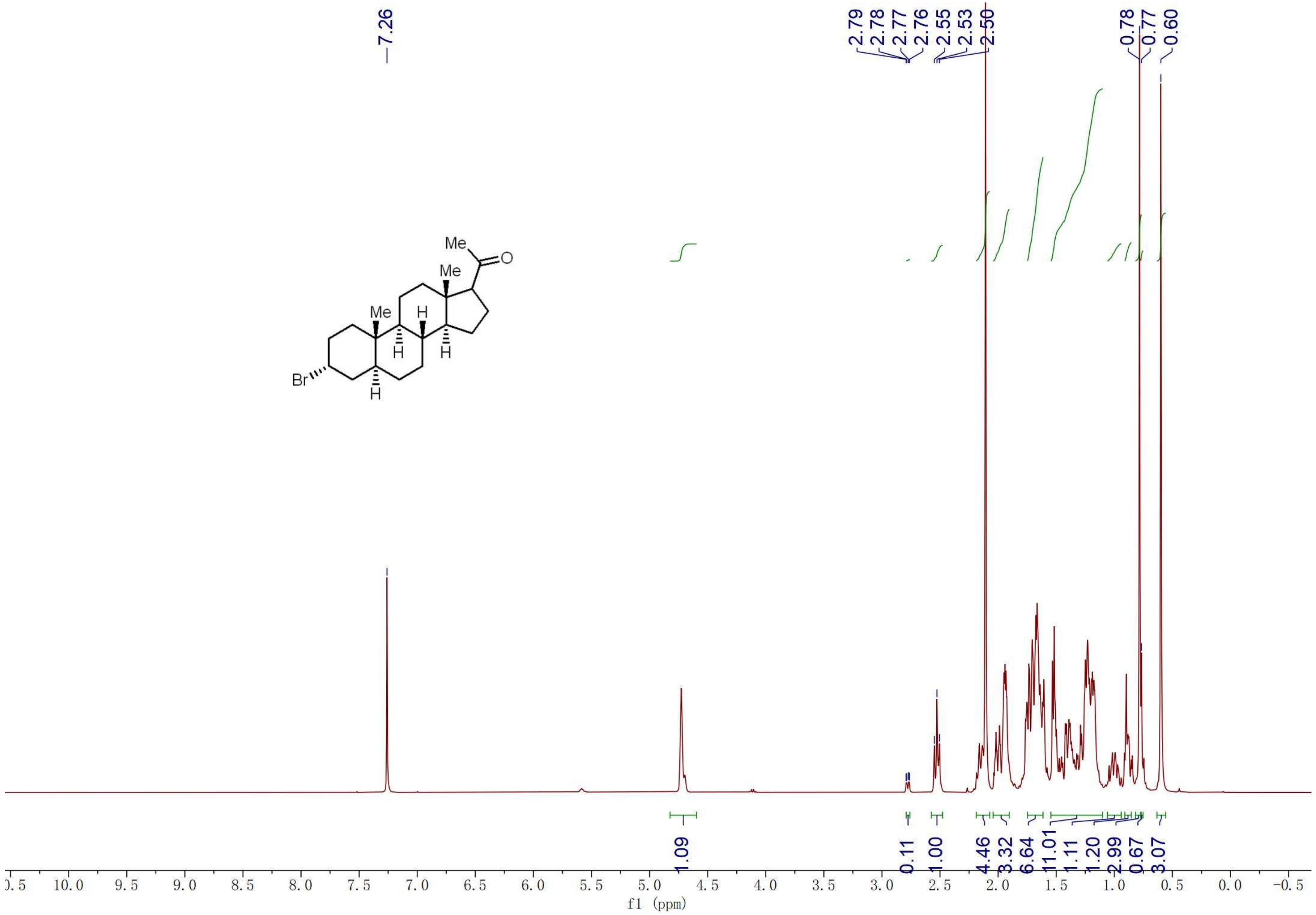

^{13}C NMR (CDCl$_3$, 101 MHz)

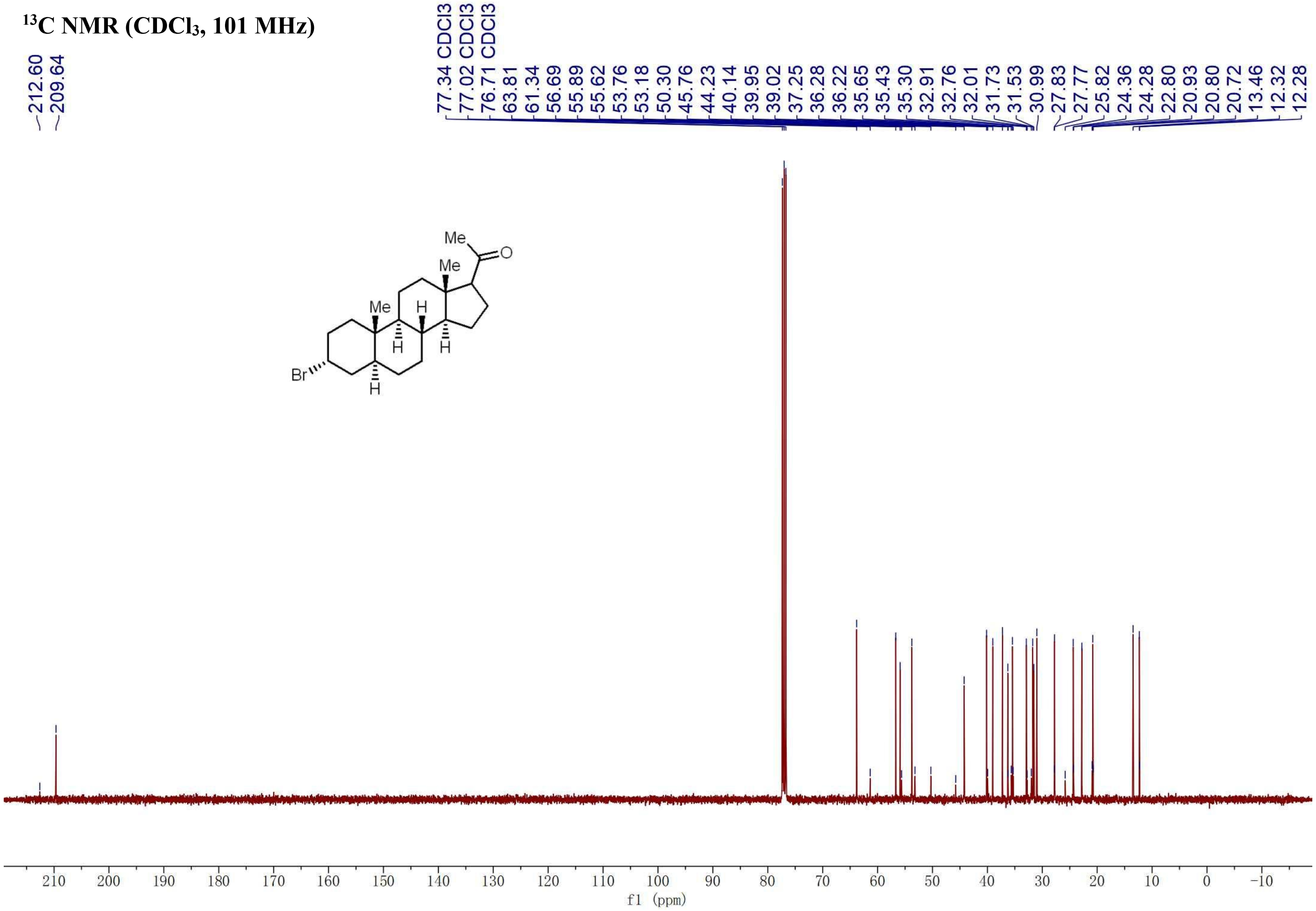

^{1}H NMR ($CDCl_3$, 400 MHz)

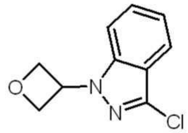

13

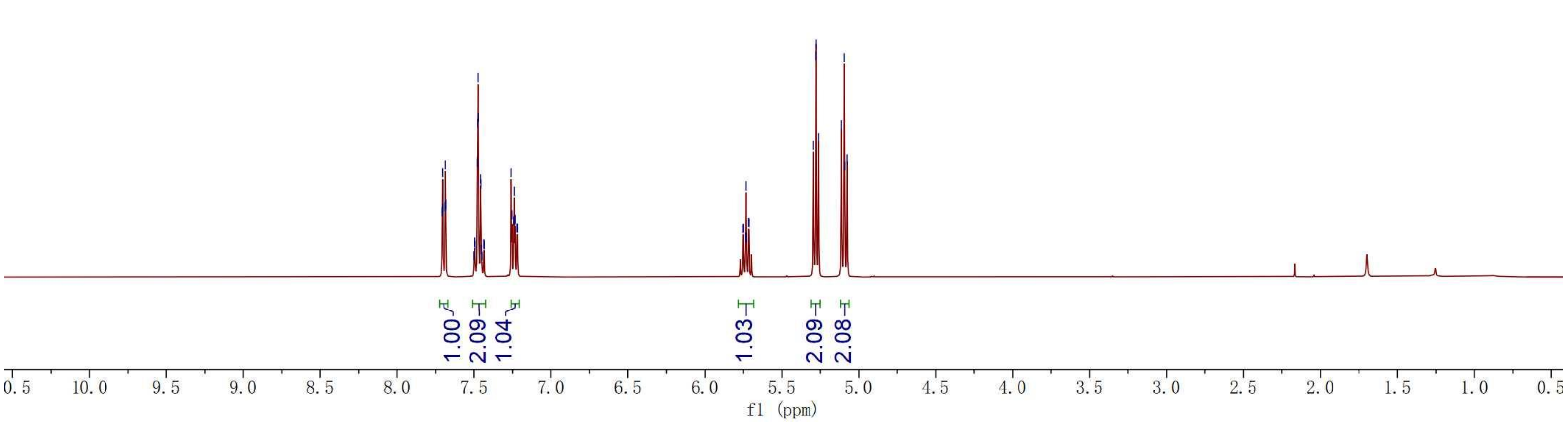

^{13}C NMR ($CDCl_3$, 101 MHz)

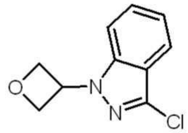

13

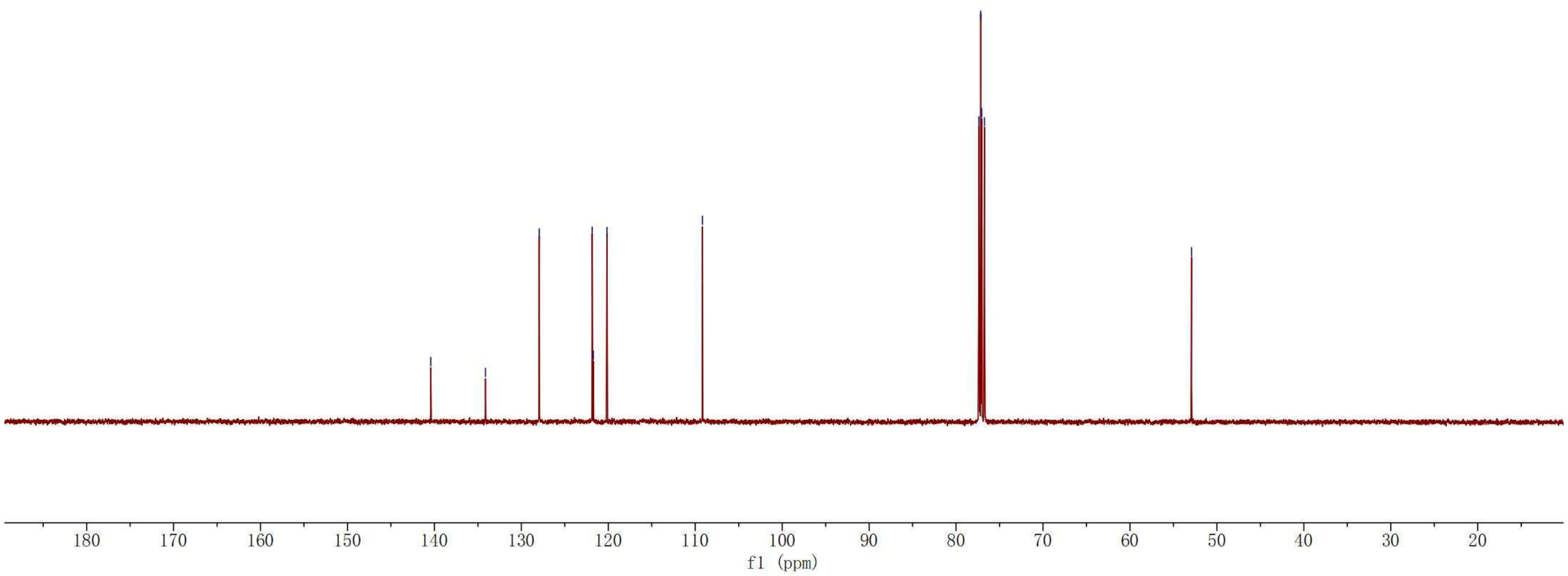

^{1}H NMR (CDCl3, 400 MHz)

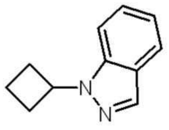

17

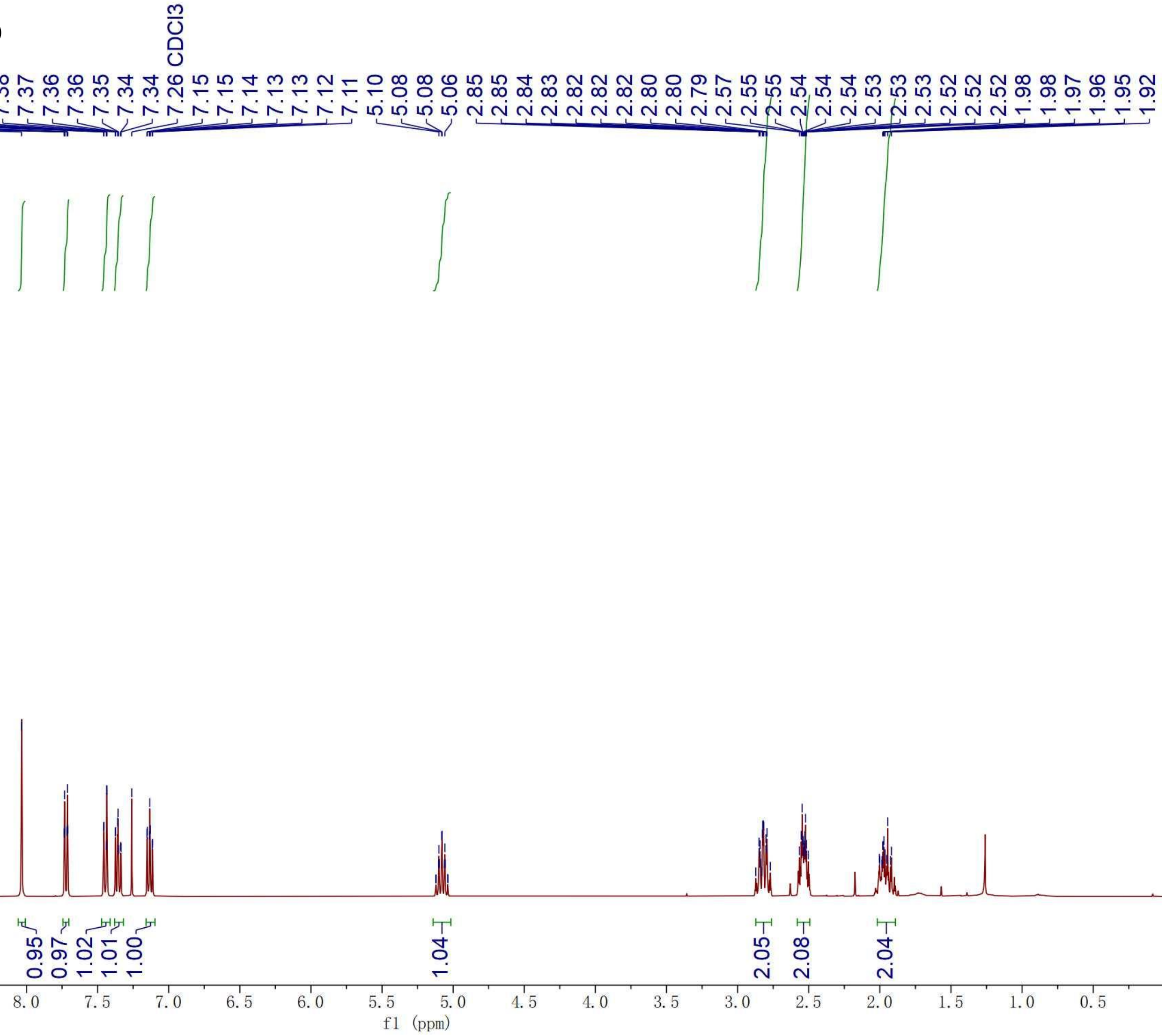

^{13}C NMR ($CDCl_3$, 101 MHz)

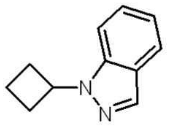

17

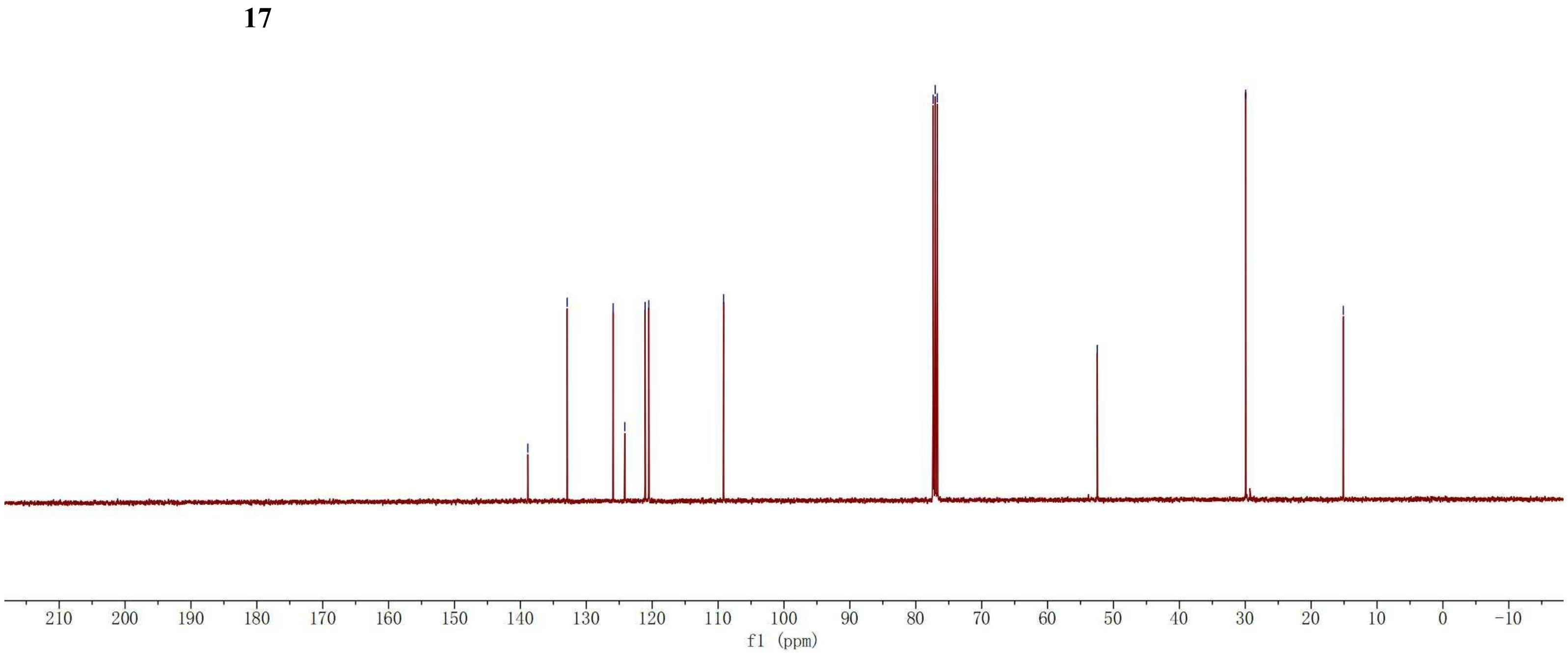

^{1}H NMR ($CDCl_3$, 400 MHz)

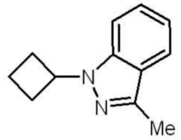

18

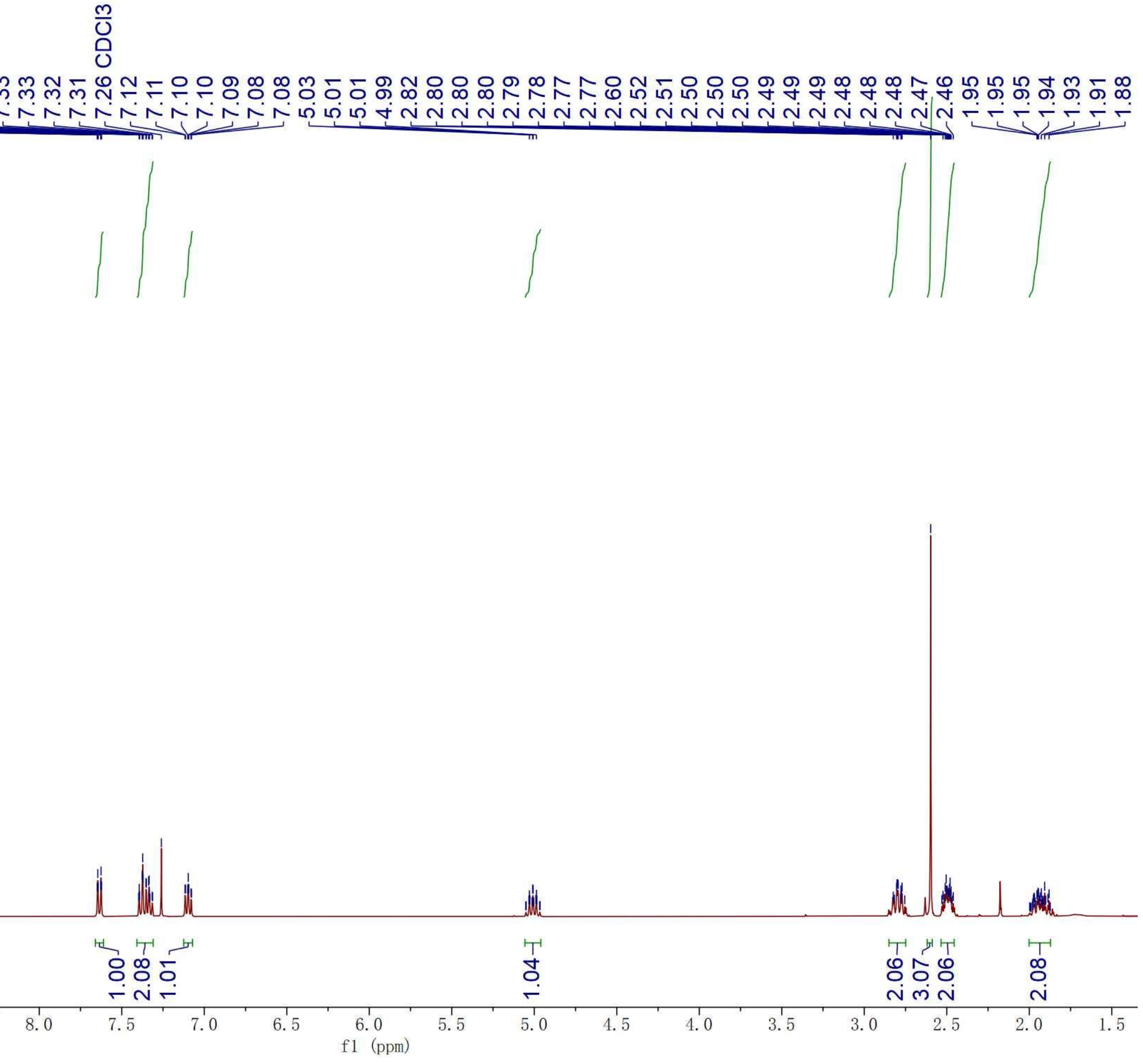

^{13}C NMR ($CDCl_3$, 101 MHz)

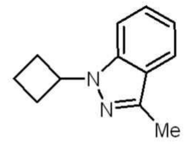

18

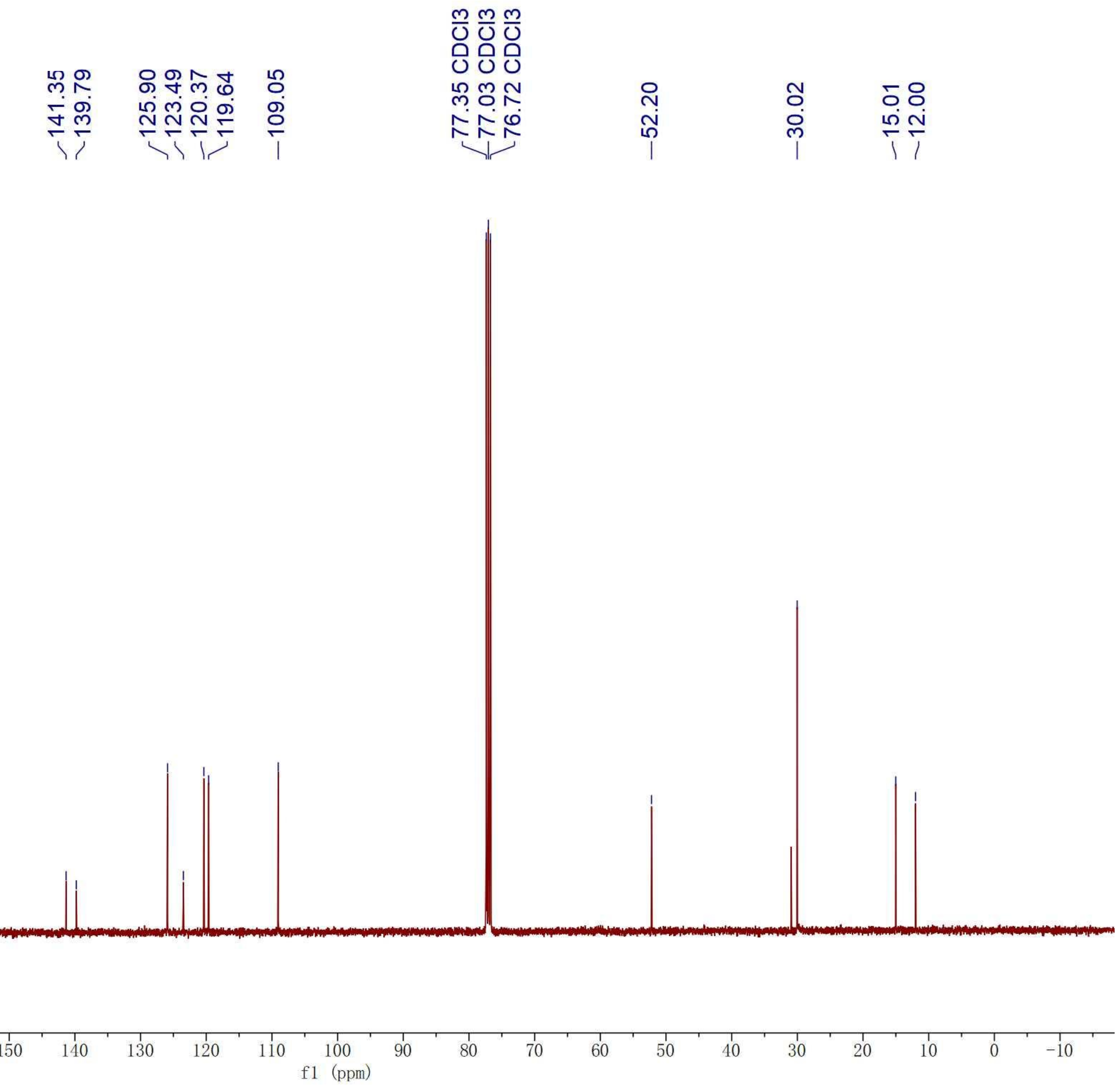

^{1}H NMR ($CDCl_3$, 400 MHz)

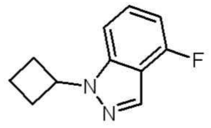

19

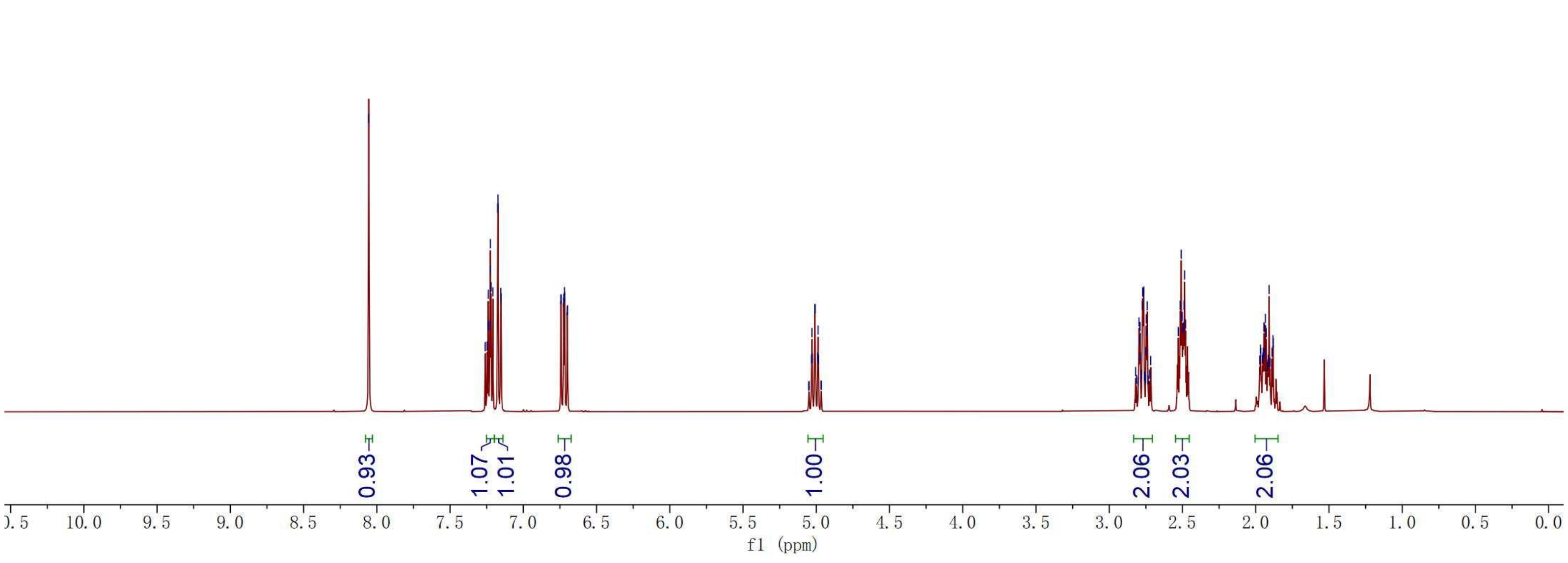

^{19}F NMR ($CDCl_3$, 376 MHz)

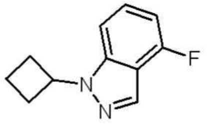

19

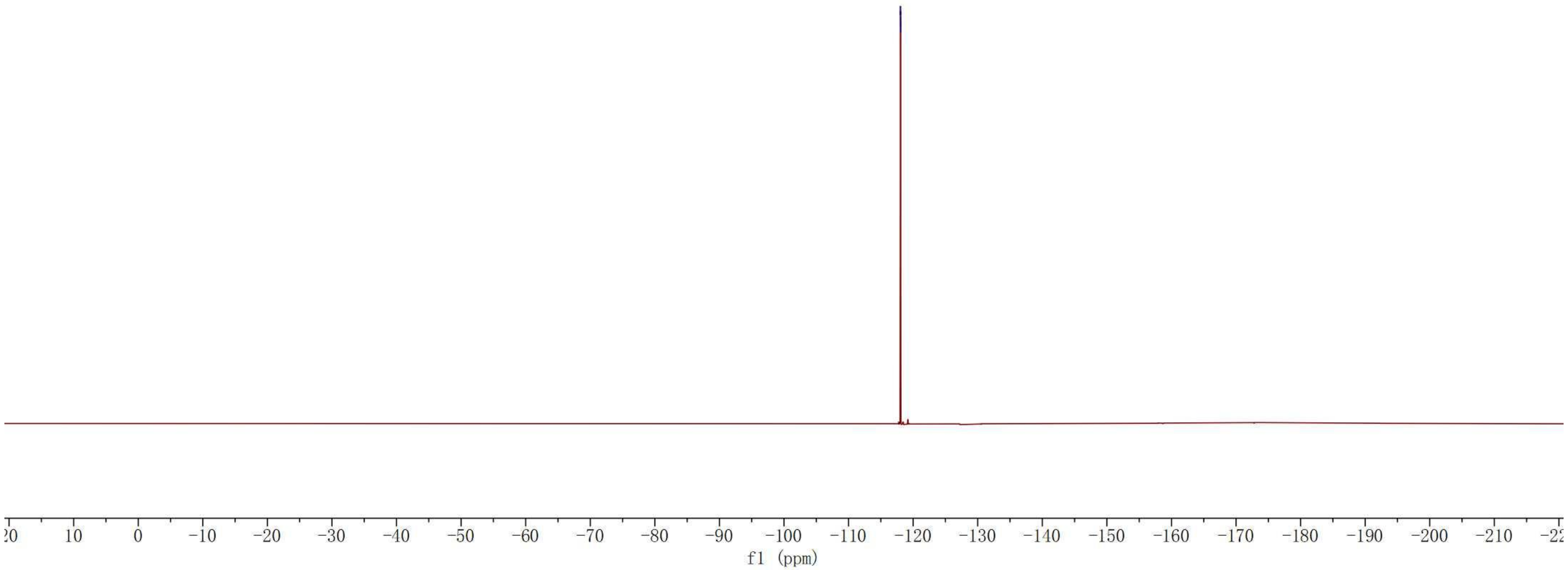

^{13}C NMR ($CDCl_3$, 101 MHz)

19

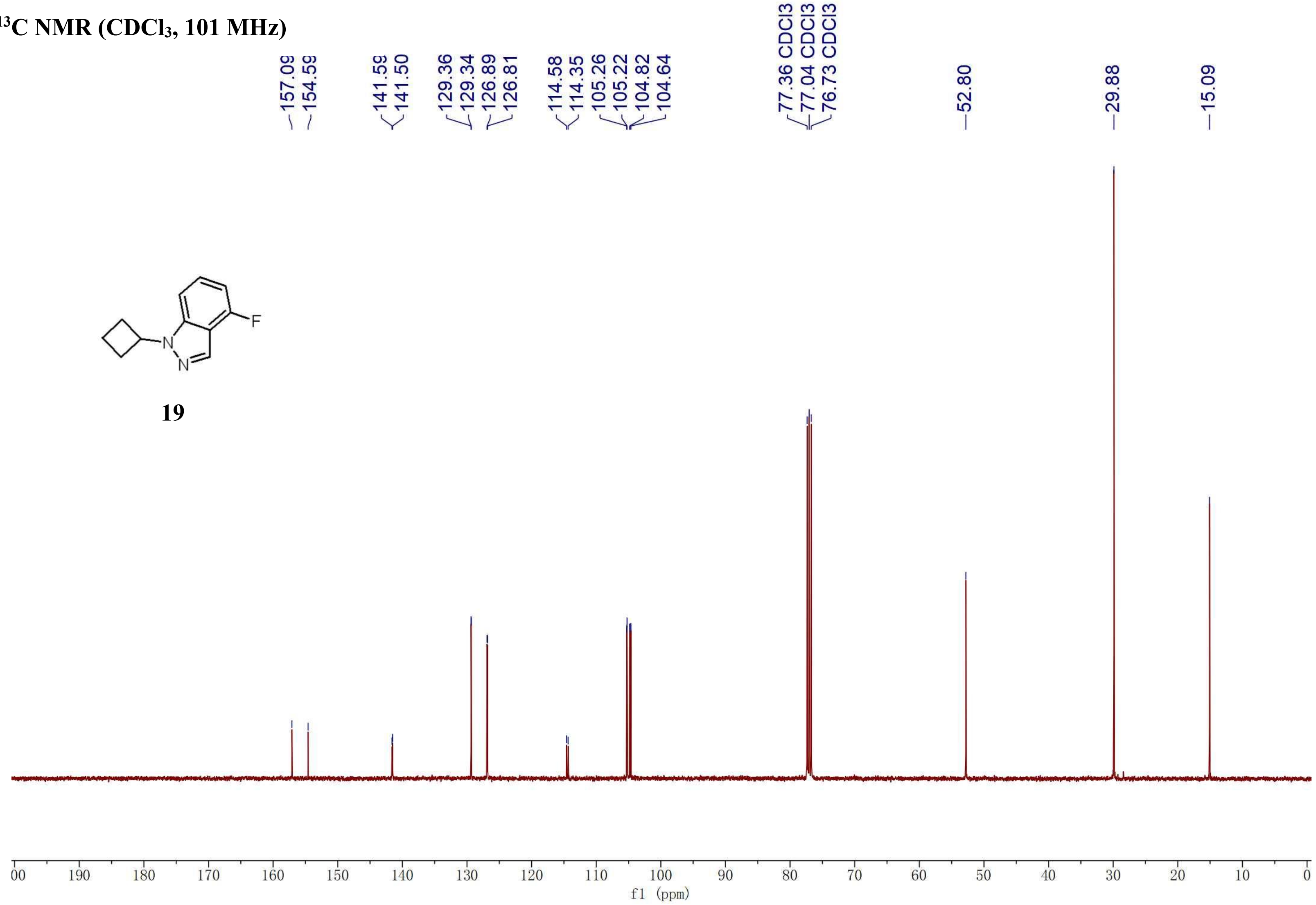

^{1}H NMR (CDCl$_3$, 400 MHz)

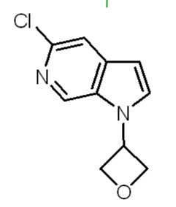

20

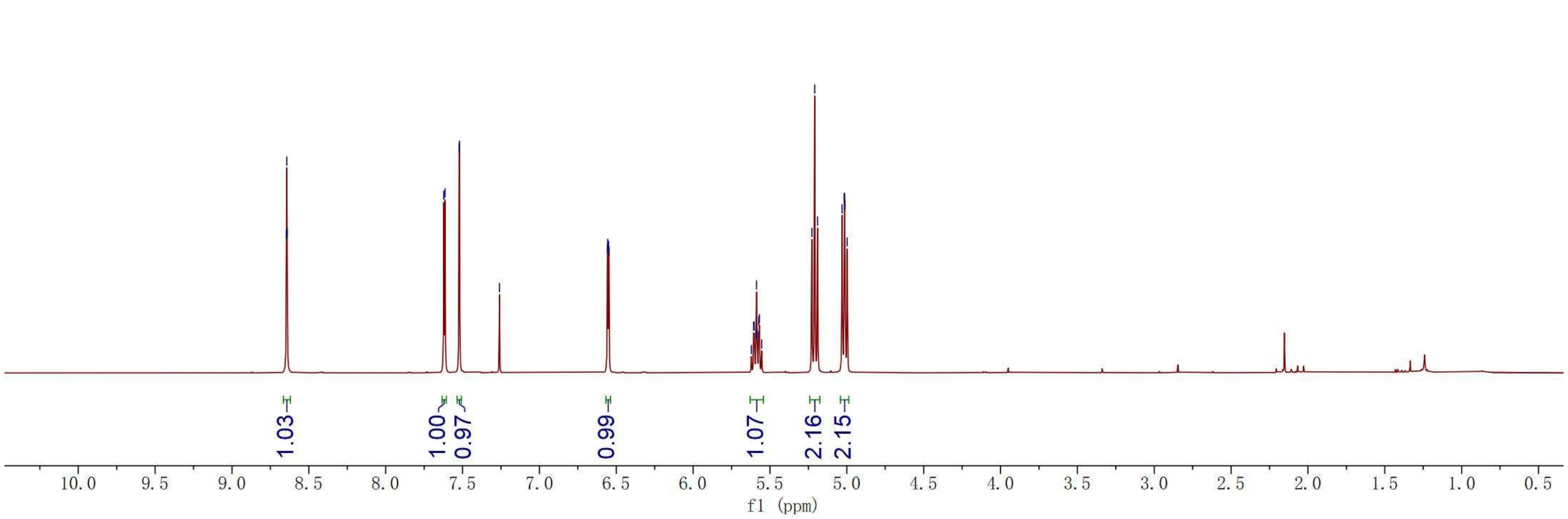

^{13}C NMR ($CDCl_3$, 101 MHz)

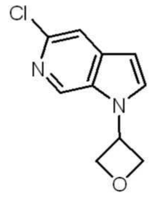

20

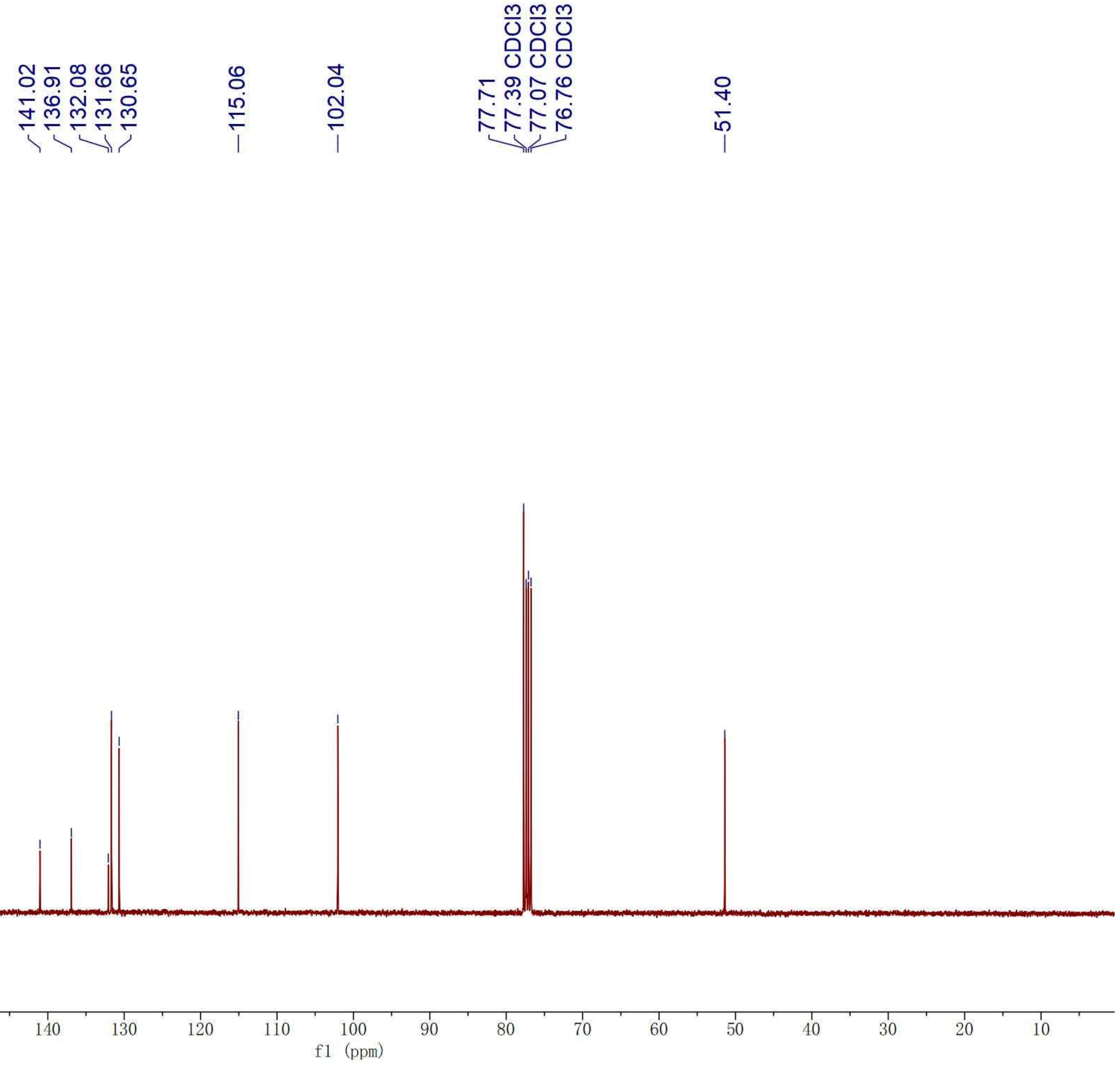

^{1}H NMR ($CDCl_3$, 400 MHz)

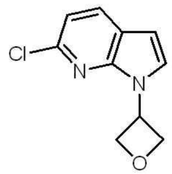

21

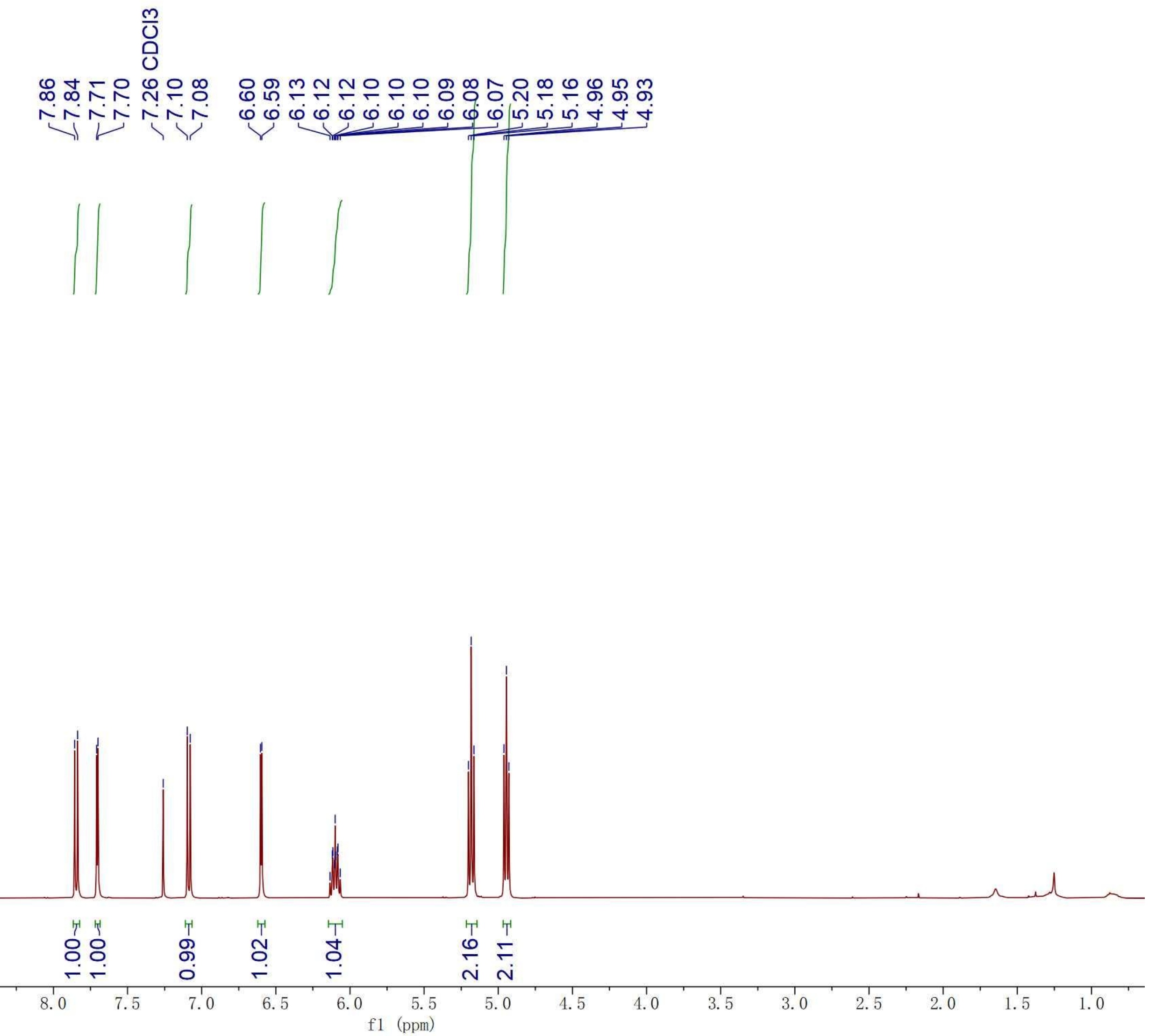

^{13}C NMR ($CDCl_3$, 101 MHz)

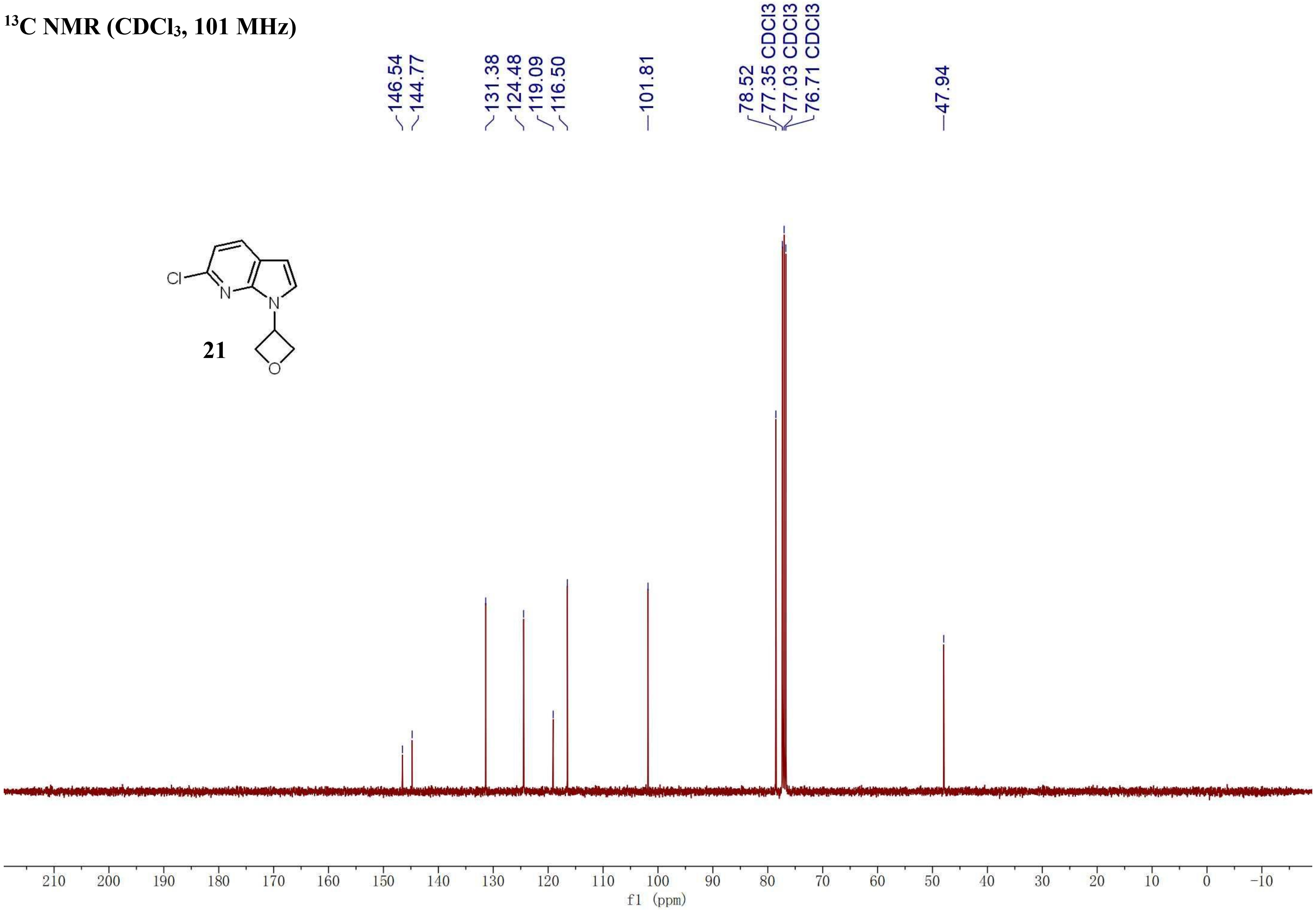

21

^{1}H NMR ($CDCl_3$, 400 MHz)

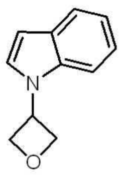

22

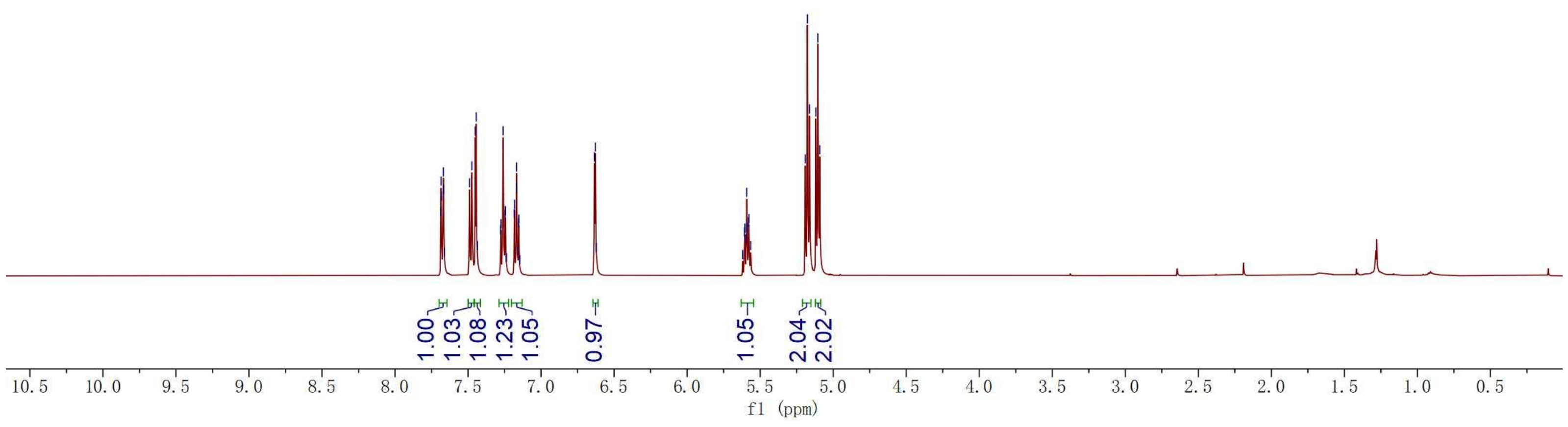

^{13}C NMR ($CDCl_3$, 101 MHz)

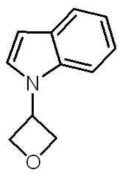

22

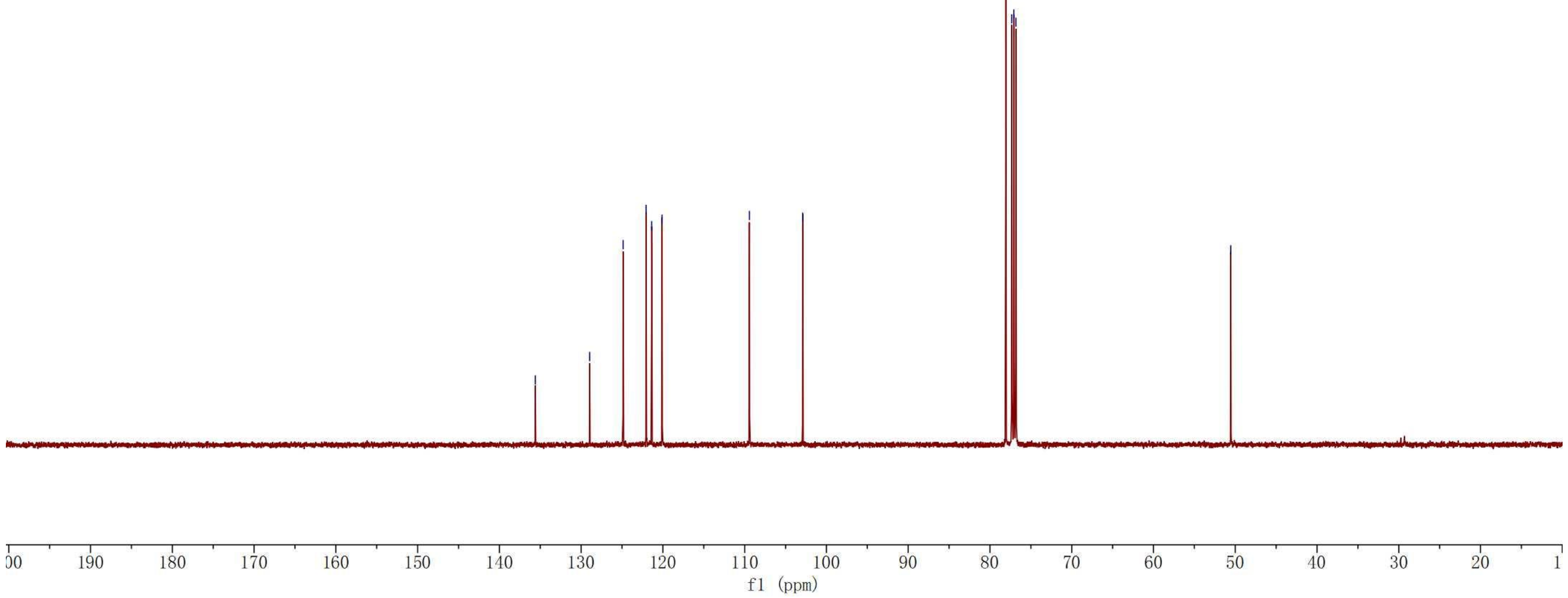

^{1}H NMR ($CDCl_3$, 400 MHz)

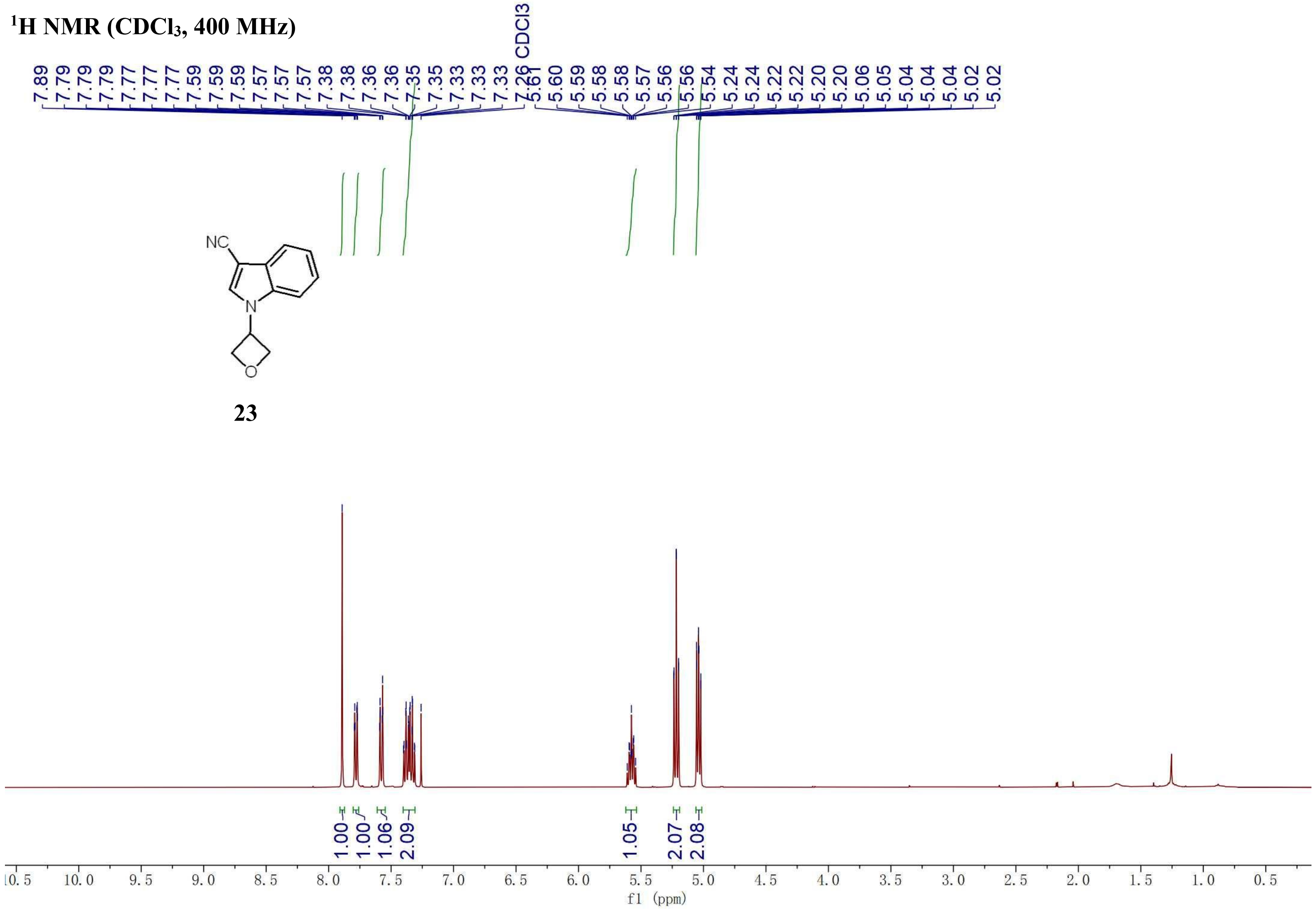

23

^{13}C NMR ($CDCl_3$, 101 MHz)

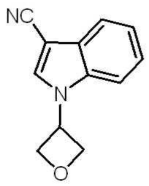

23

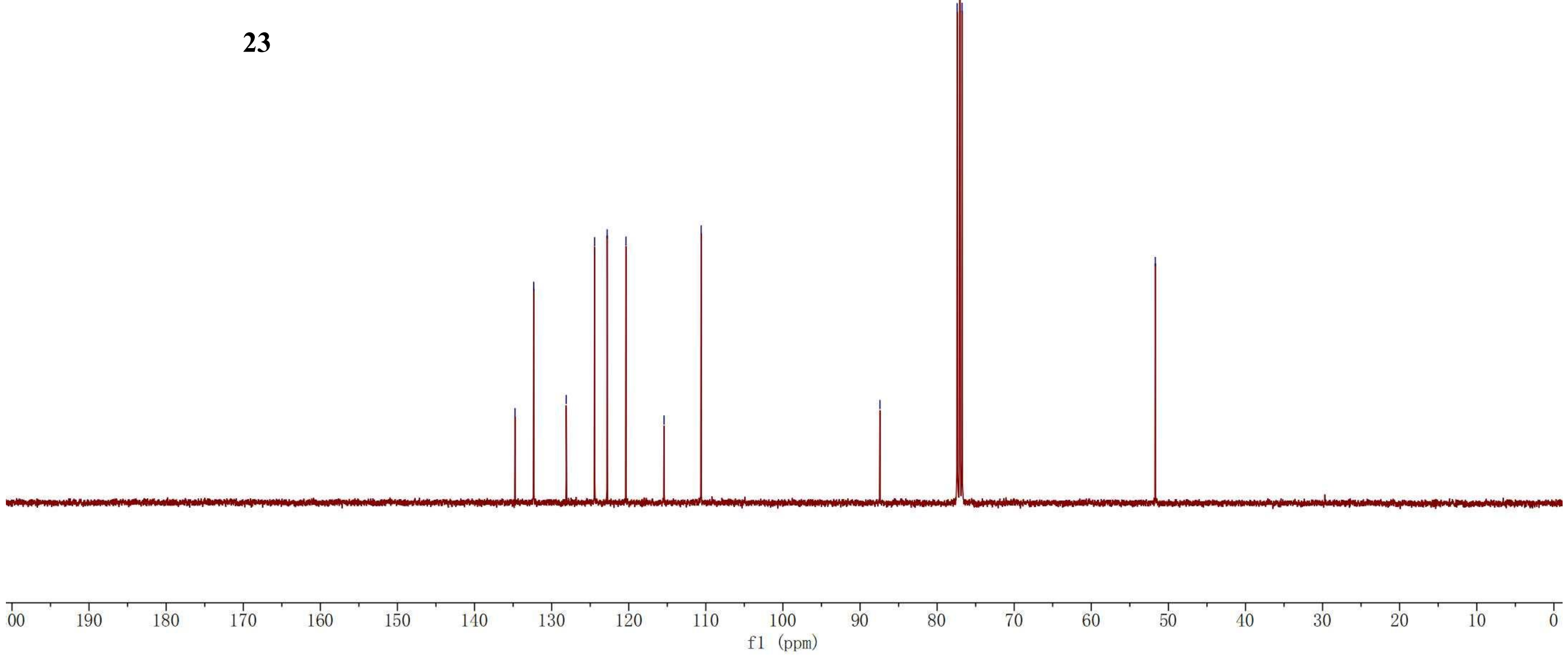

^1H NMR ($CDCl_3$, 400 MHz)

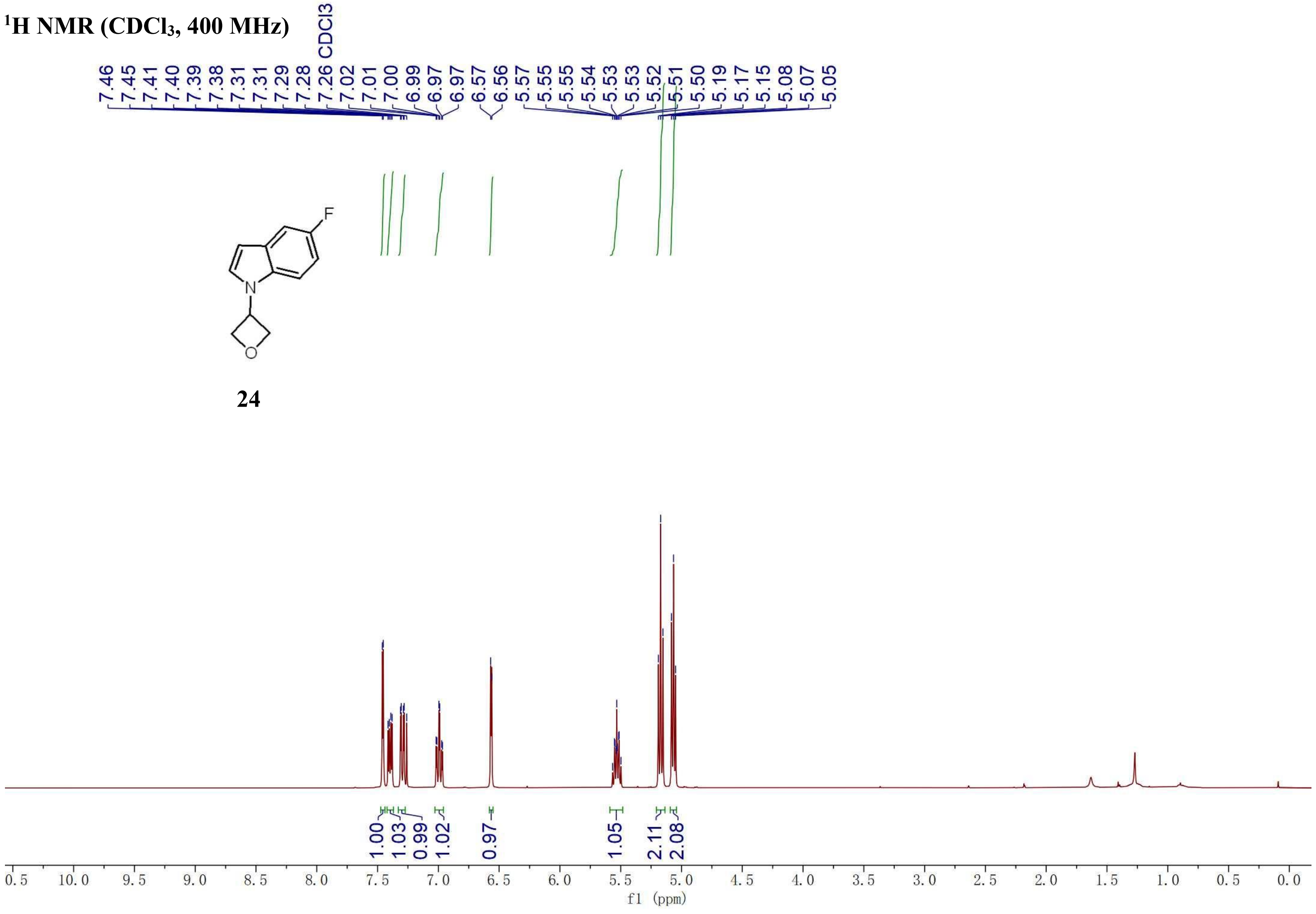

24

^{19}F NMR ($CDCl_3$, 376 MHz)

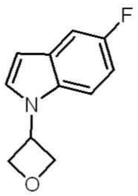

24

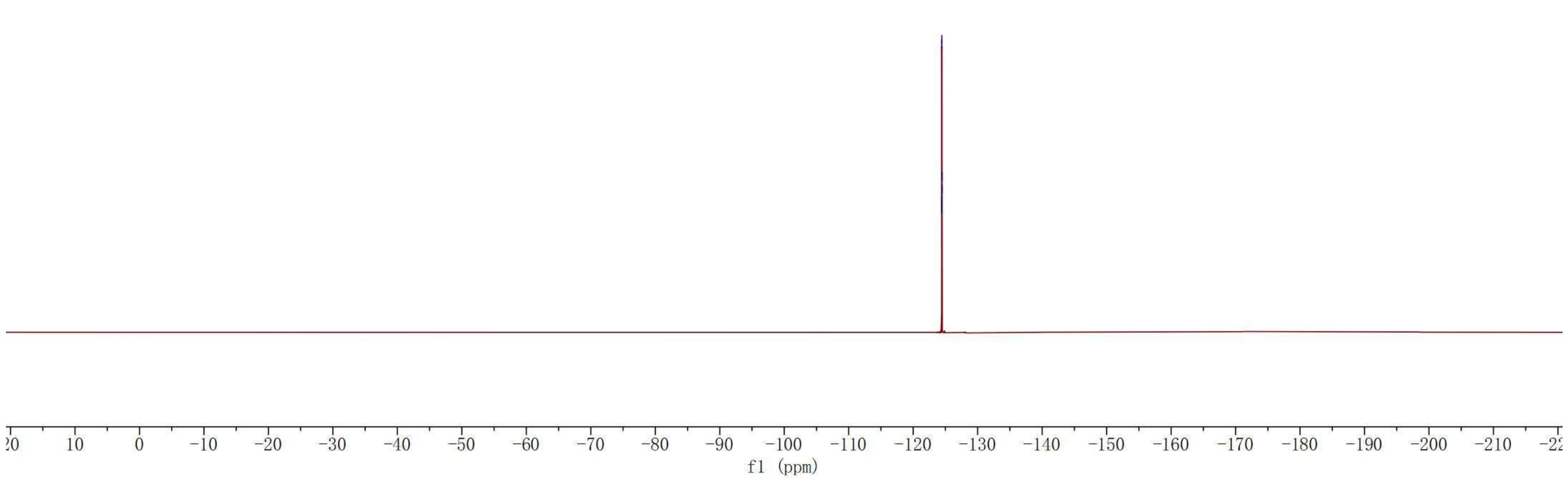

^{13}C NMR ($CDCl_3$, 101 MHz)

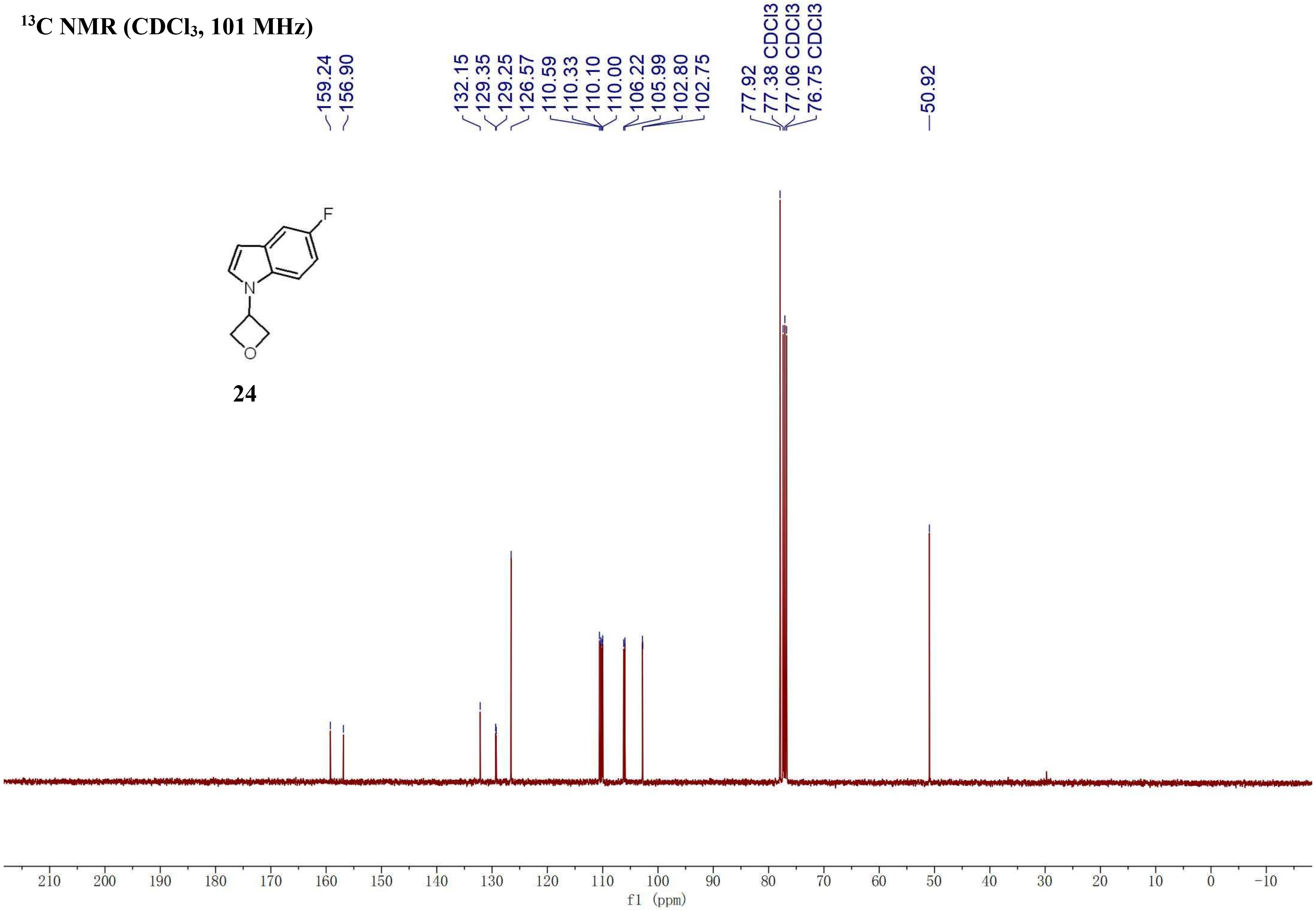

^{1}H NMR ($CDCl_3$, 400 MHz)

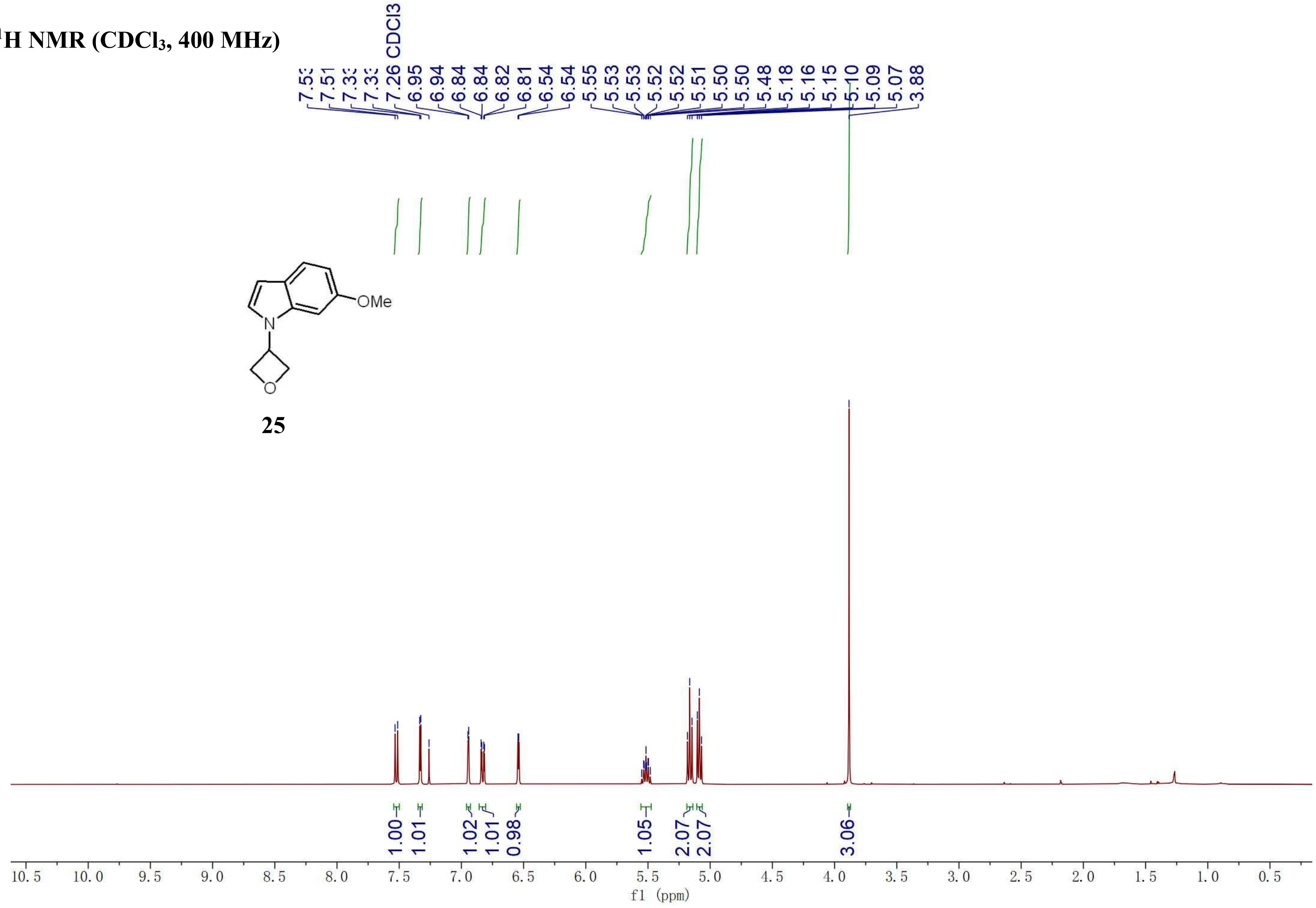

25

^{13}C NMR ($CDCl_3$, 101 MHz)

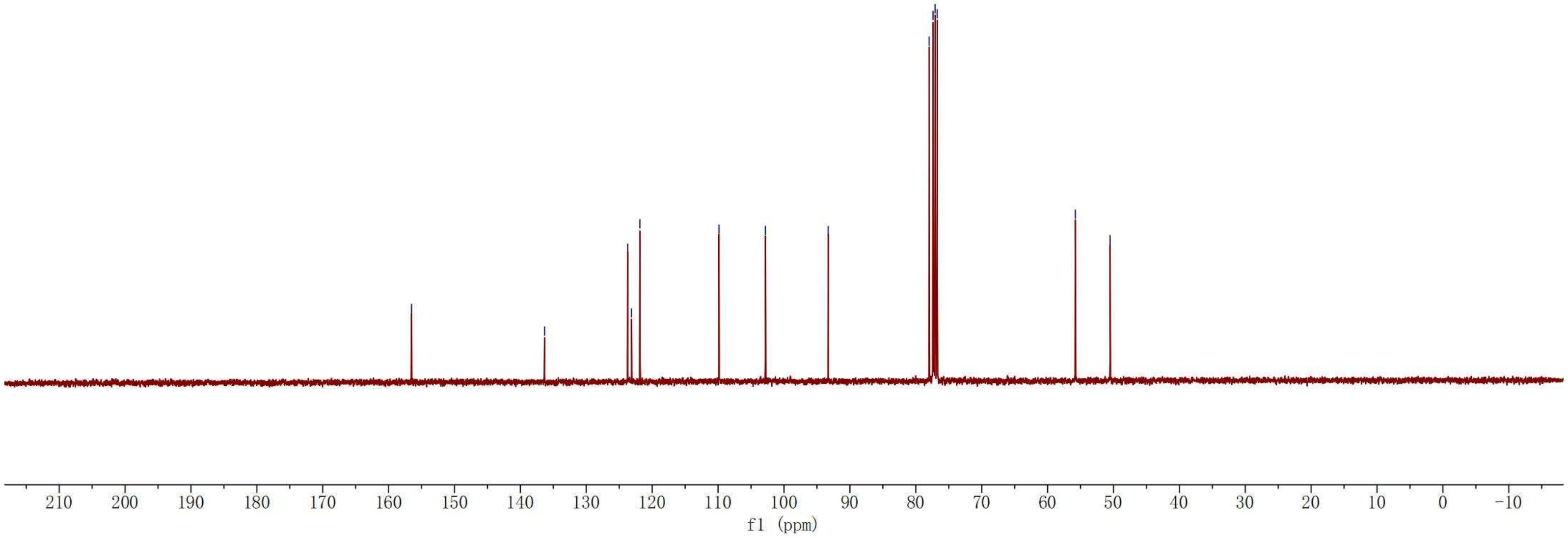

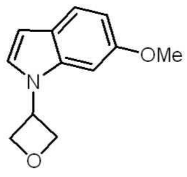

25

^{1}H NMR ($CDCl_3$, 400 MHz)

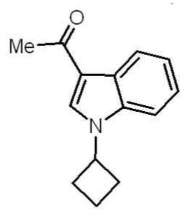

26

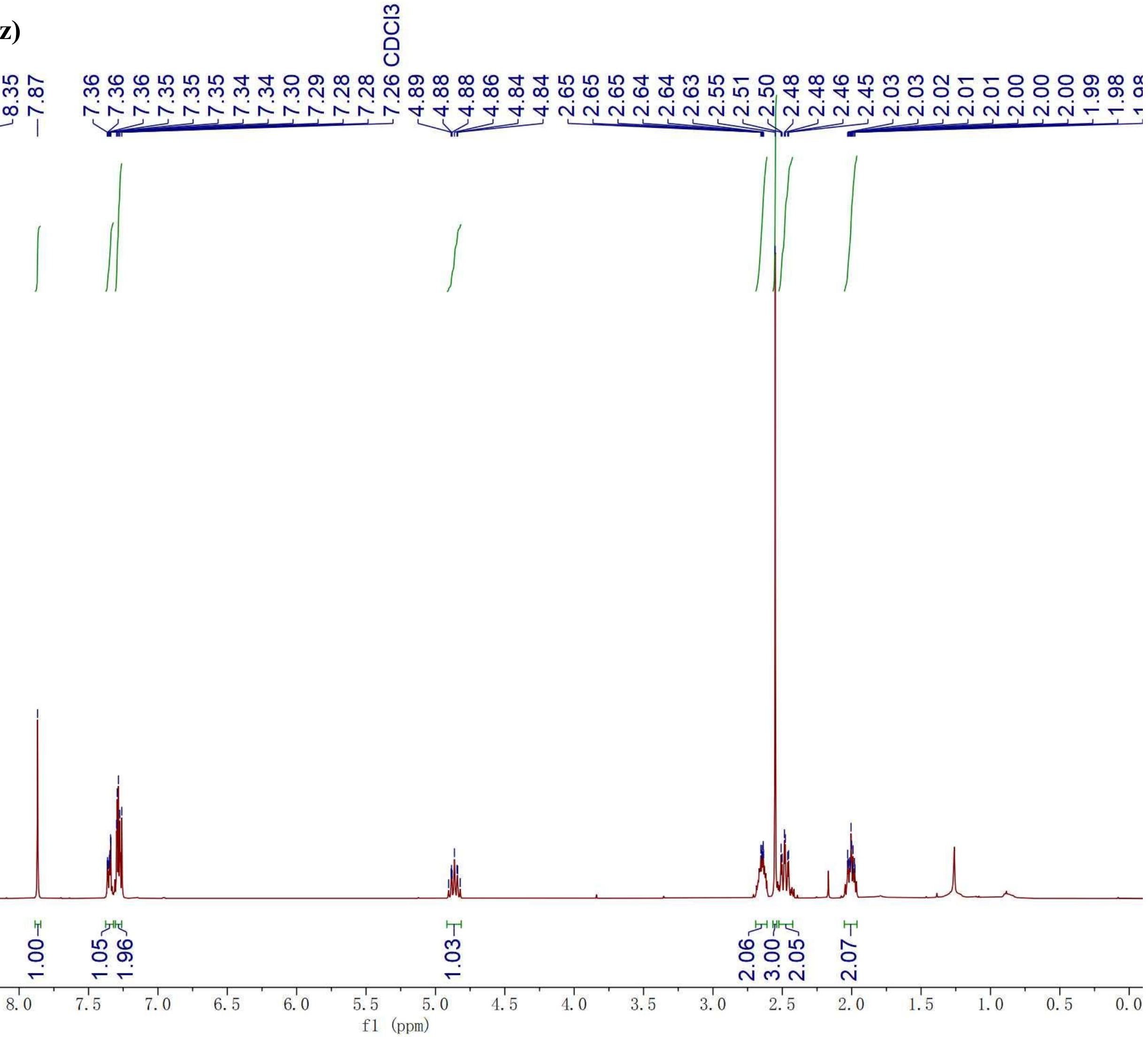

^{13}C NMR (CDCl$_3$, 101 MHz)

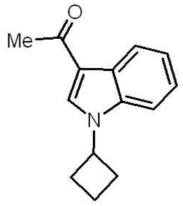

26

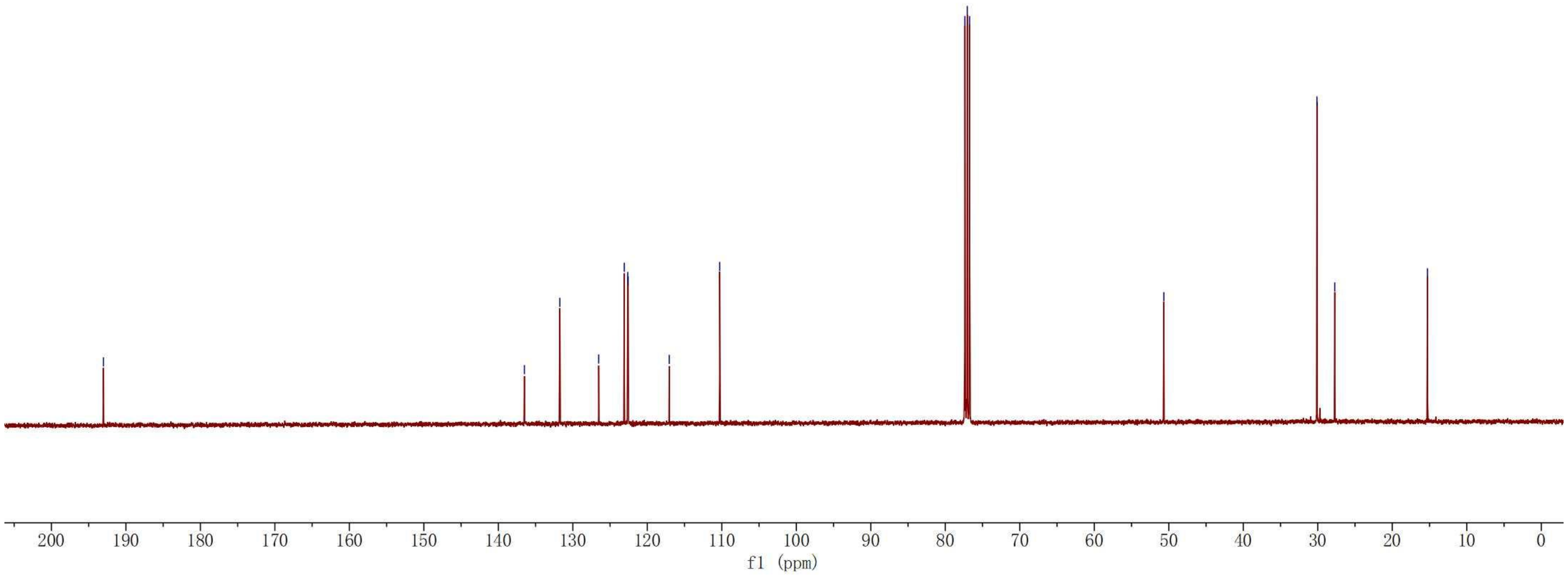

^{1}H NMR ($CDCl_3$, 400 MHz)

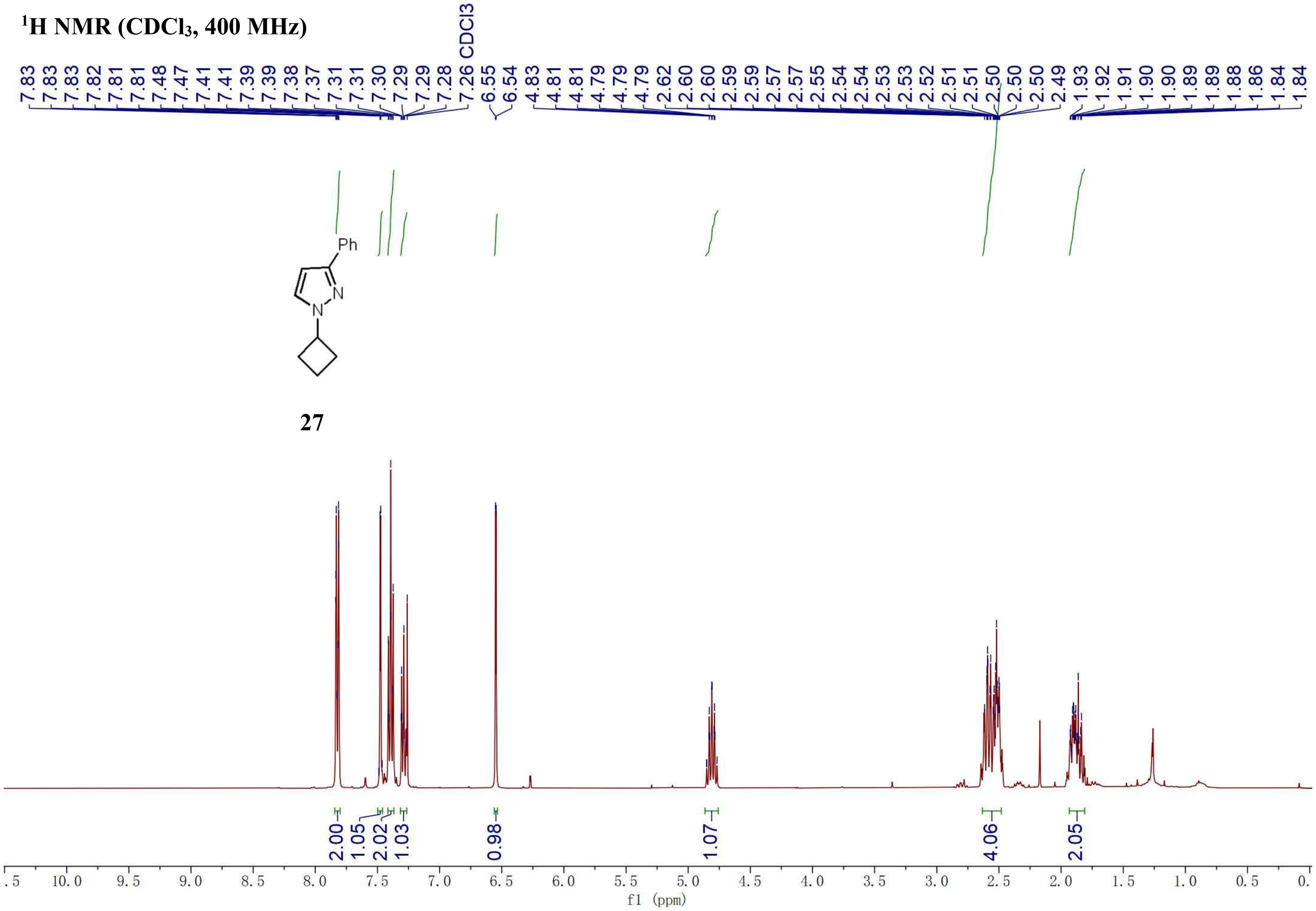

^{13}C NMR ($CDCl_3$, 101 MHz)

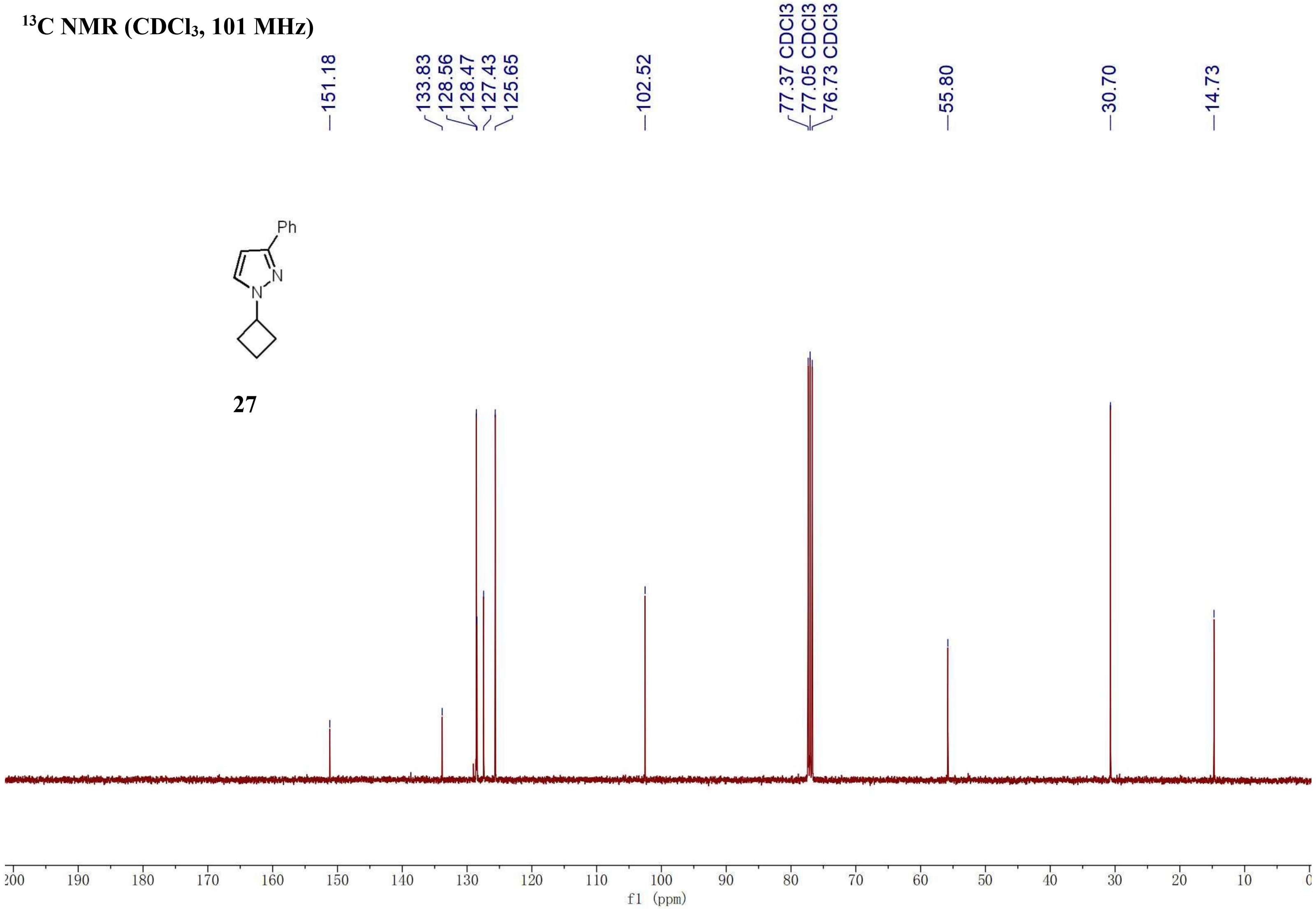

27

^{1}H NMR ($CDCl_3$, 400 MHz)

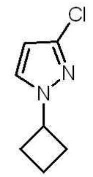

28

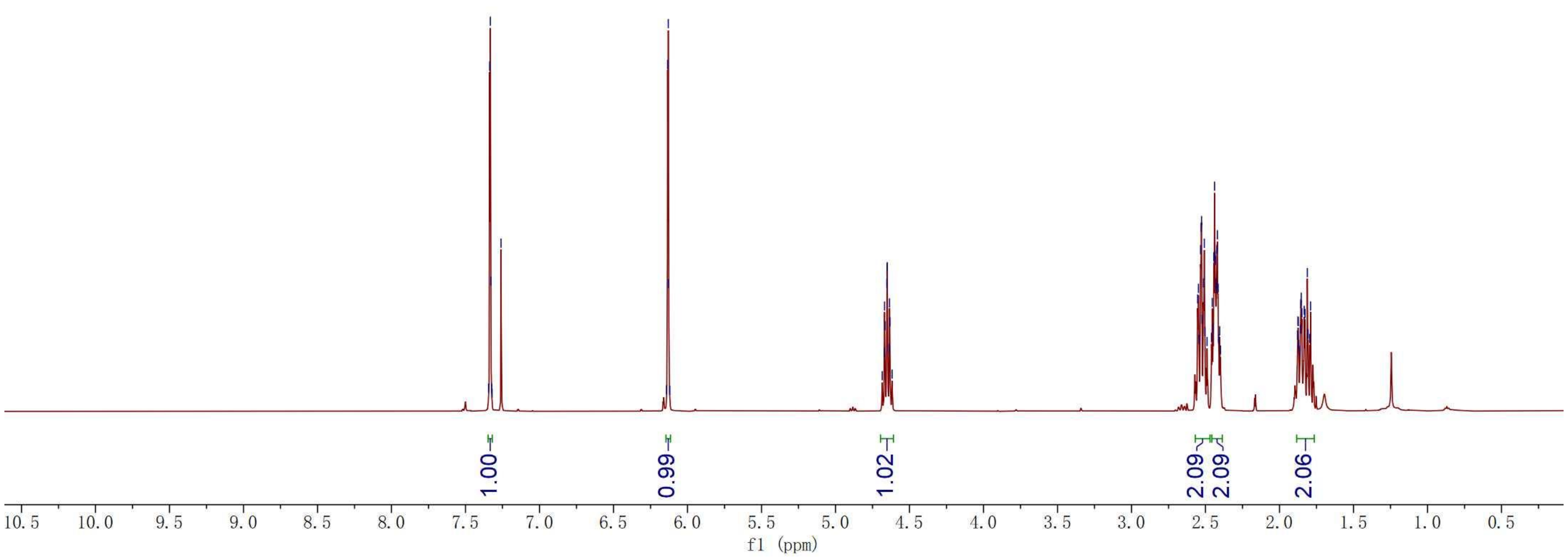

^{13}C NMR ($CDCl_3$, 101 MHz)

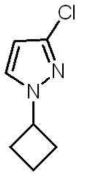

28

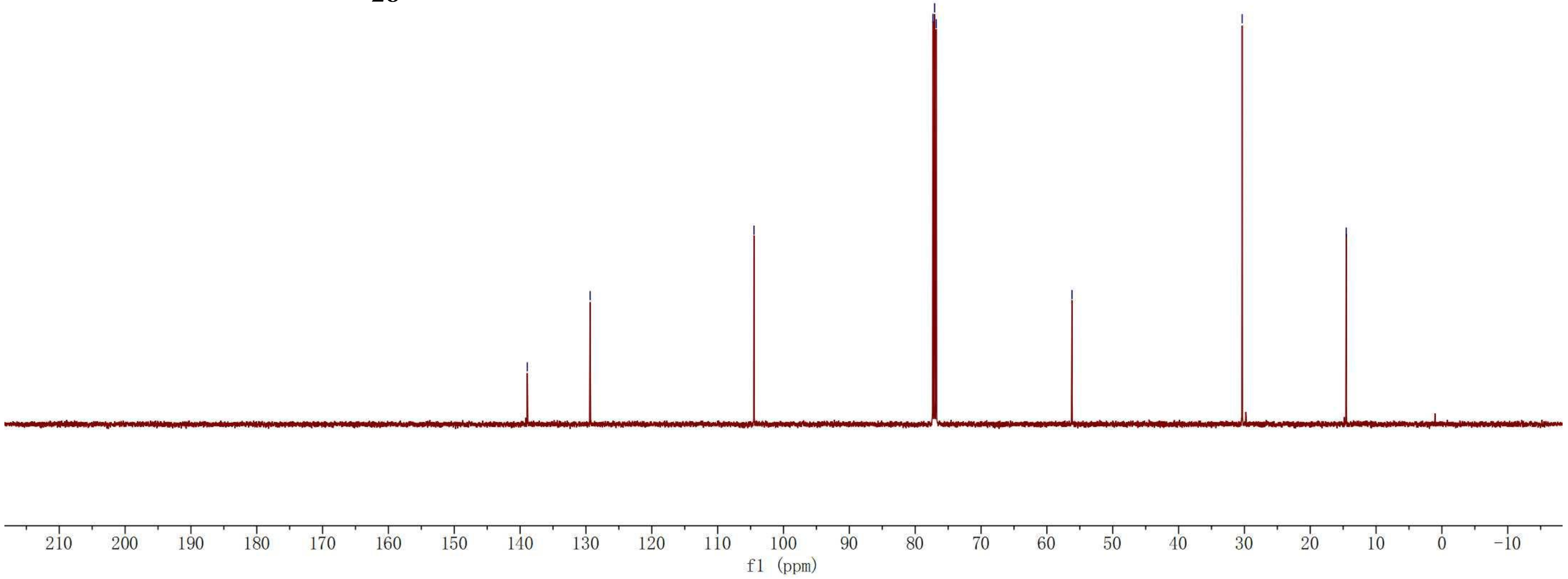

^{1}H NMR ($CDCl_3$, 400 MHz)

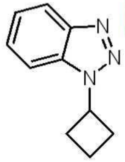

29

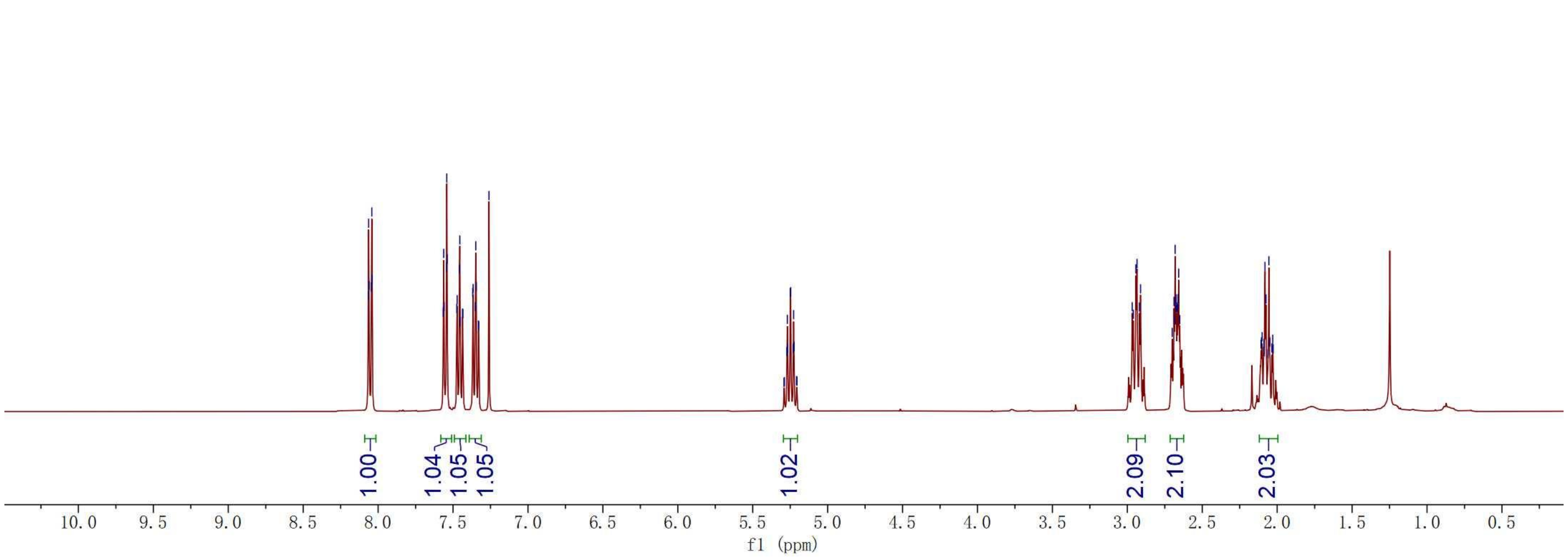

^{13}C NMR ($CDCl_3$, 101 MHz)

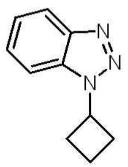

29

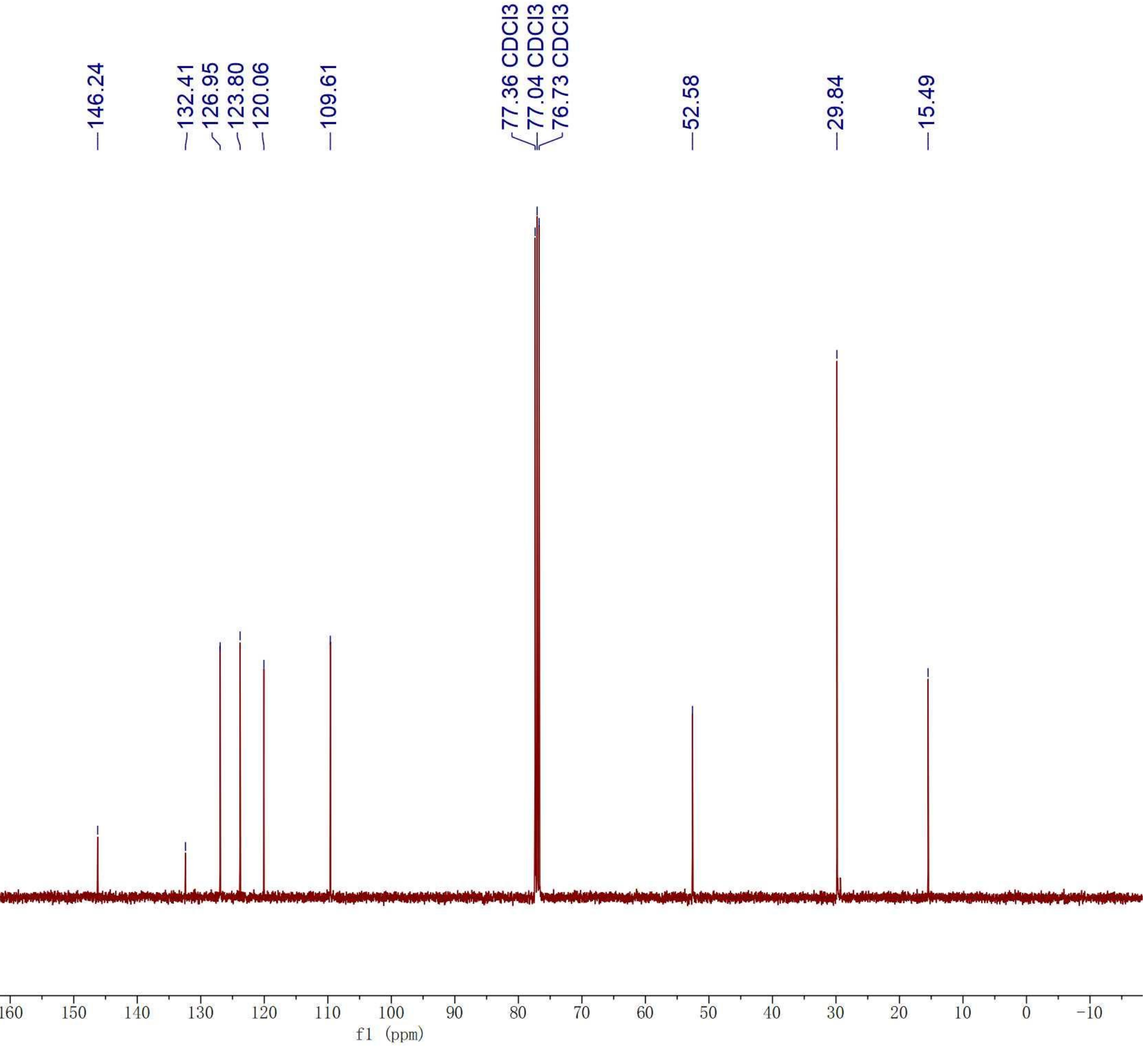

^{1}H NMR ($CDCl_3$, 400 MHz)

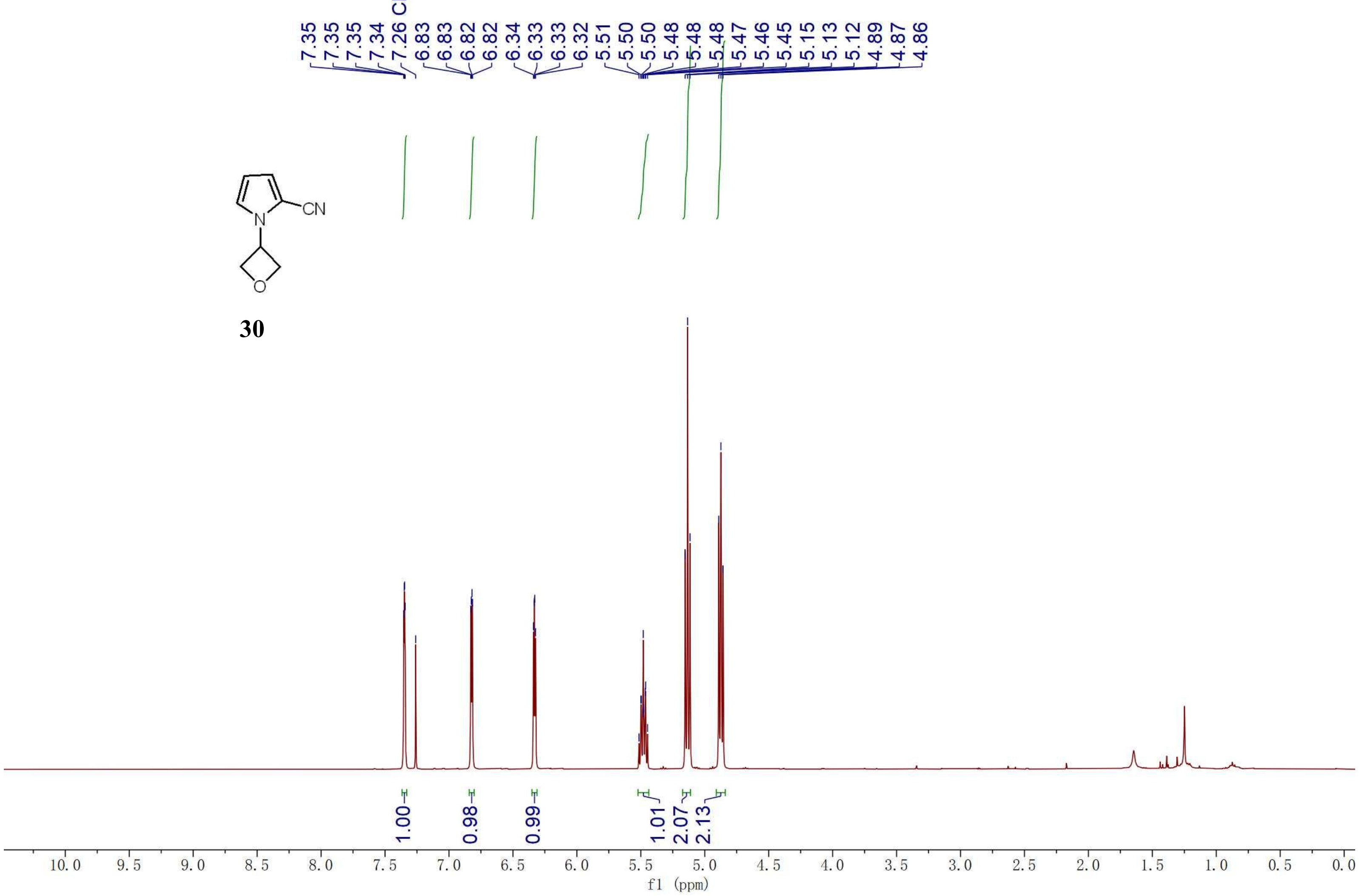

30

^{13}C NMR (CDCl$_3$, 101 MHz)

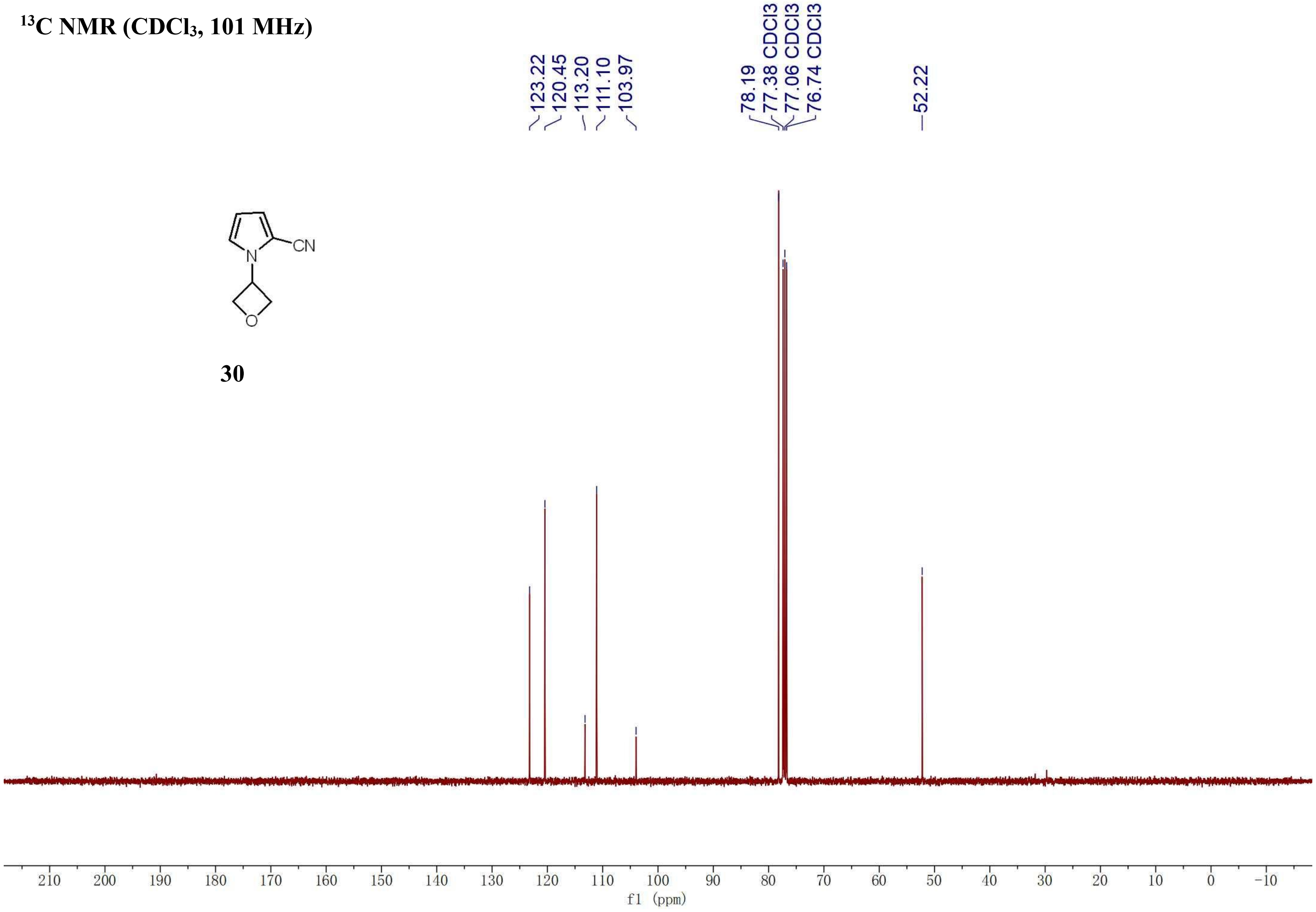

^{1}H NMR ($CDCl_3$, 400 MHz)

31

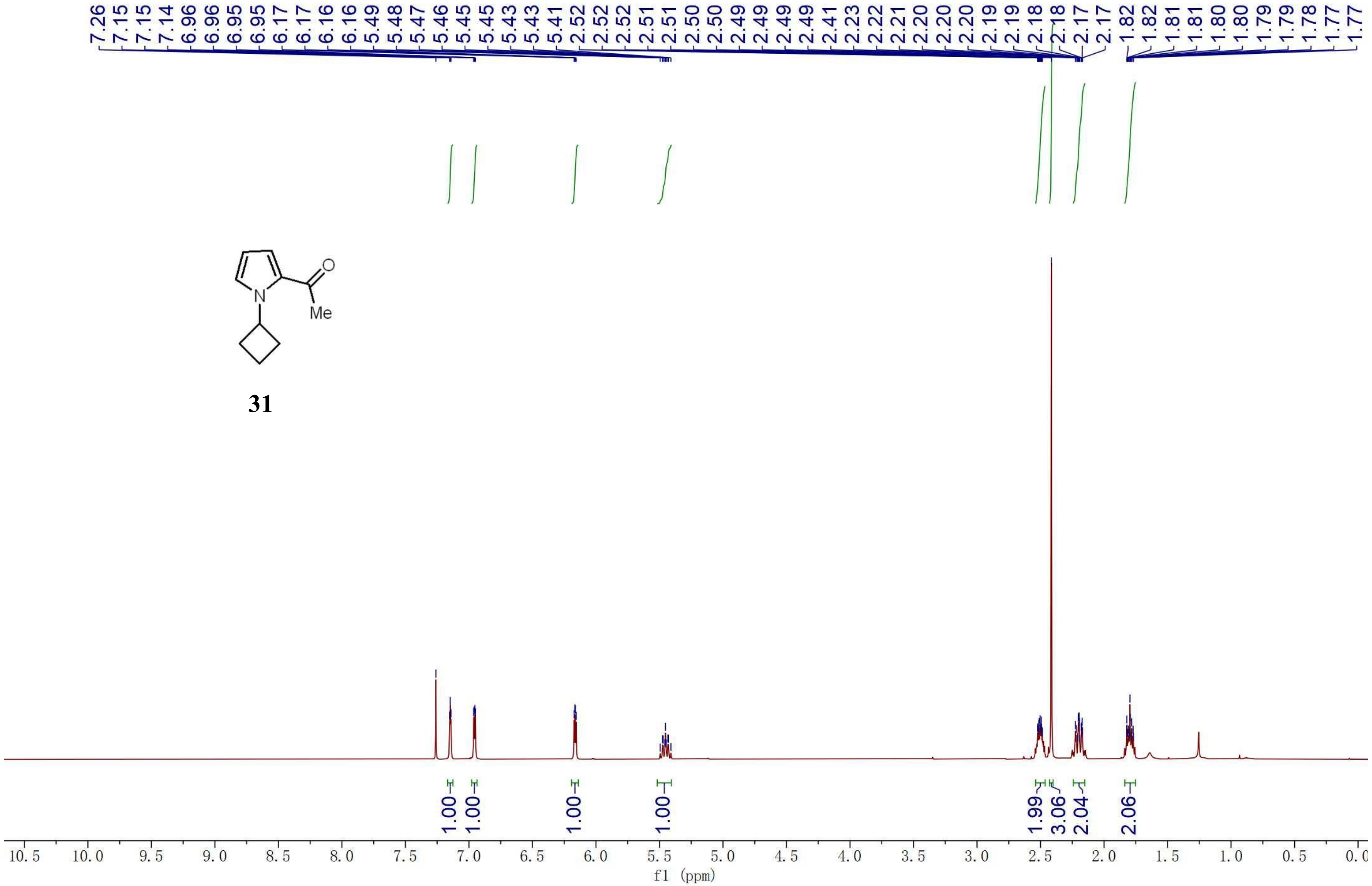

^{13}C NMR ($CDCl_3$, 101 MHz)

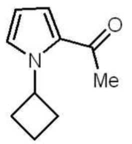

31

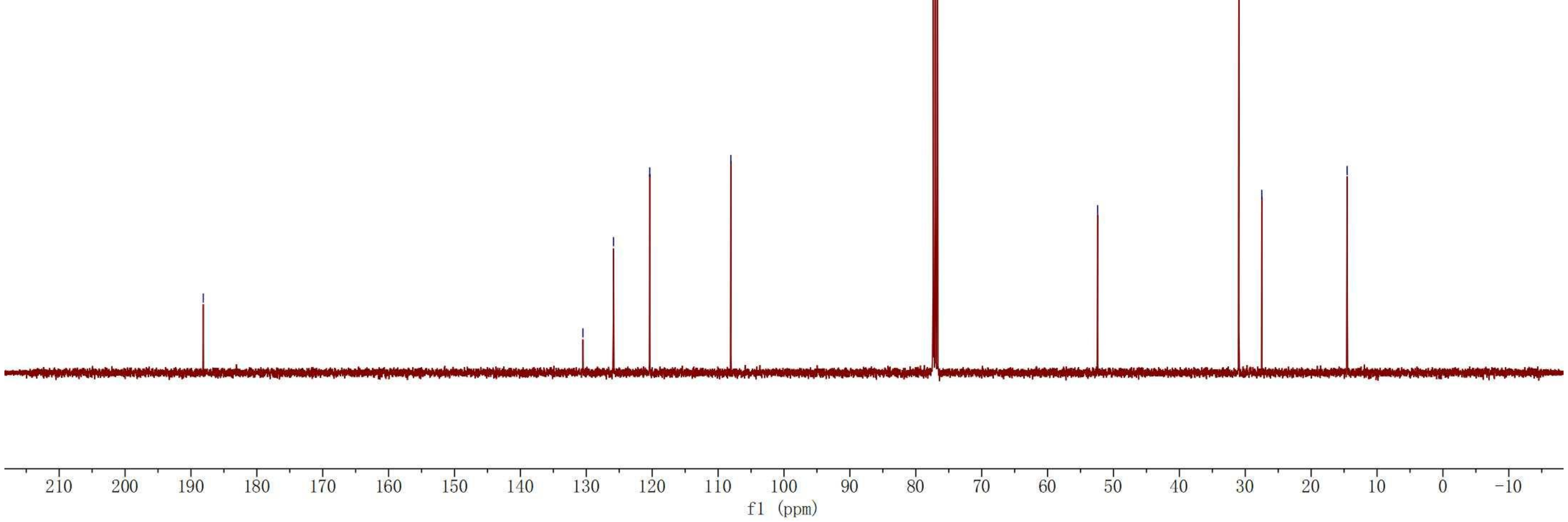

^{1}H NMR of 32 (CDCl$_3$, 400 MHz)

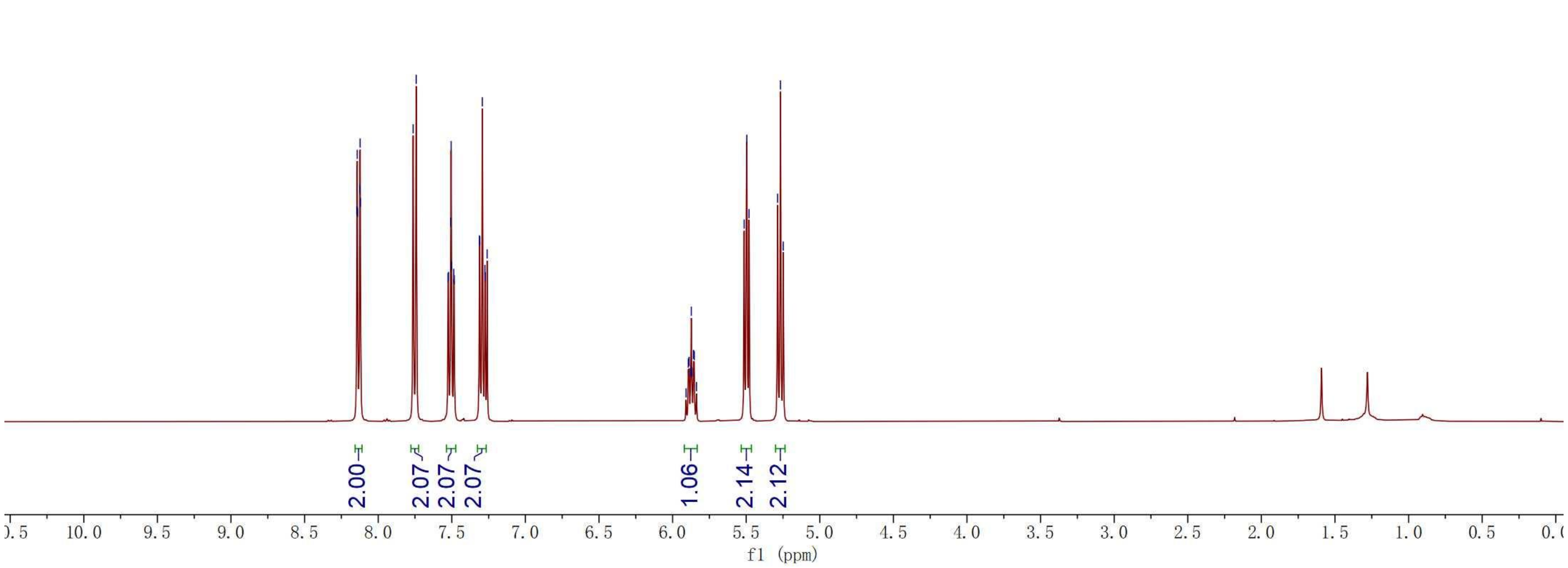

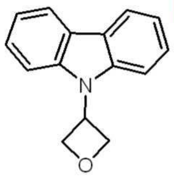

32

^{13}C NMR ($CDCl_3$, 101 MHz)

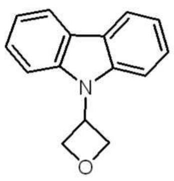

32

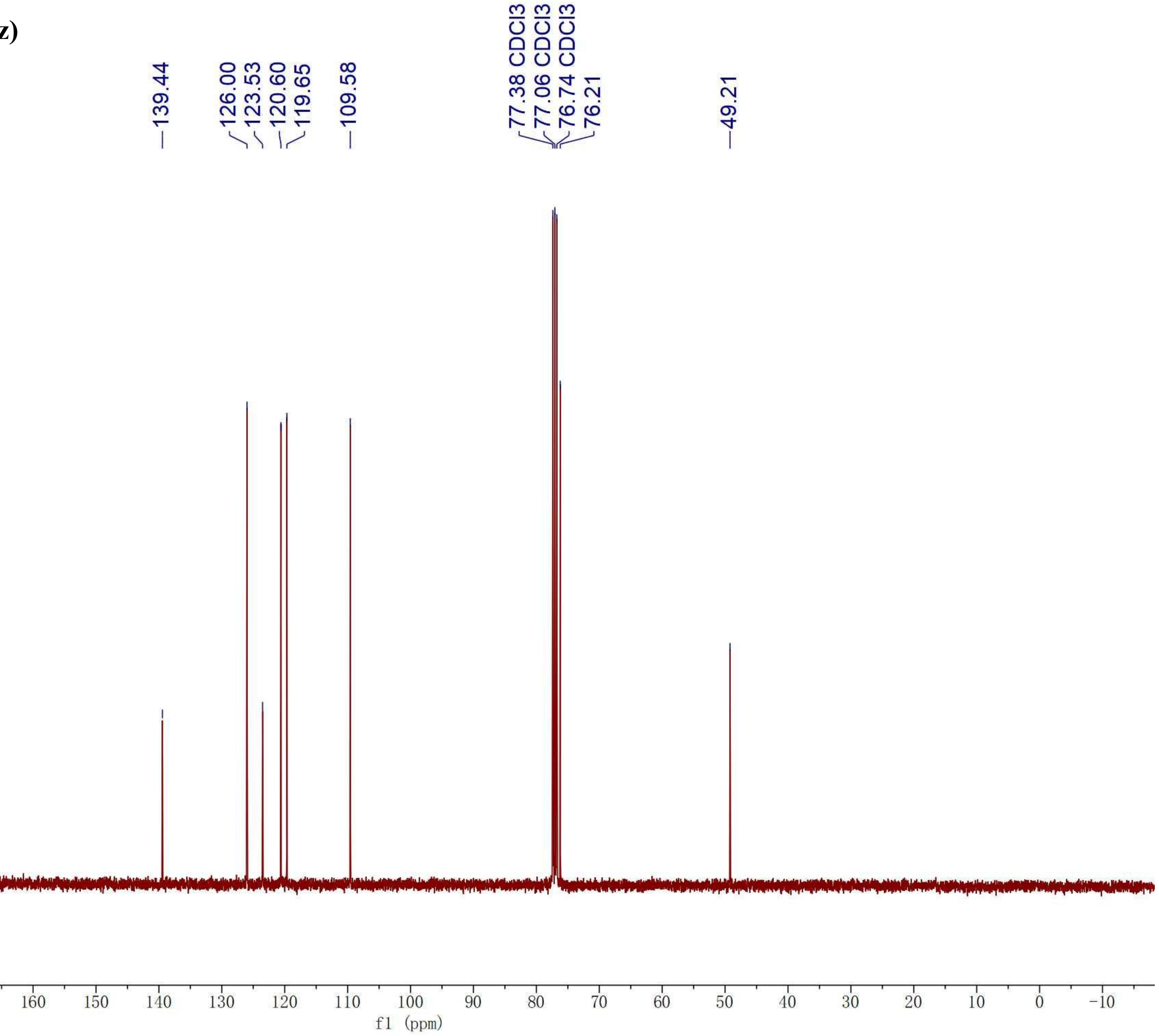

^{1}H NMR ($CDCl_3$, 400 MHz)

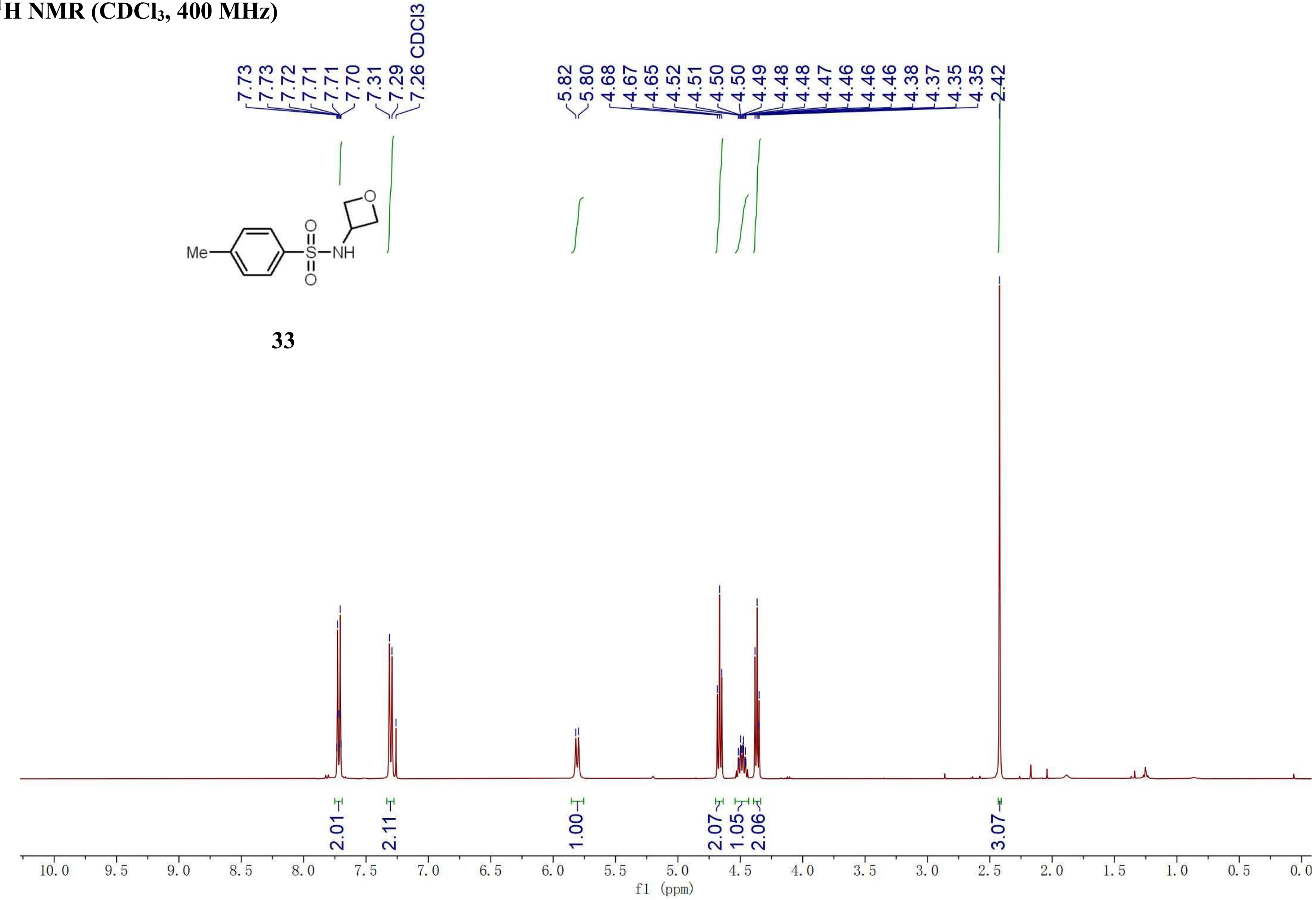

^{13}C NMR ($CDCl_3$, 101 MHz)

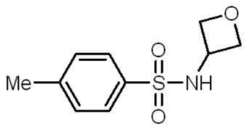

33

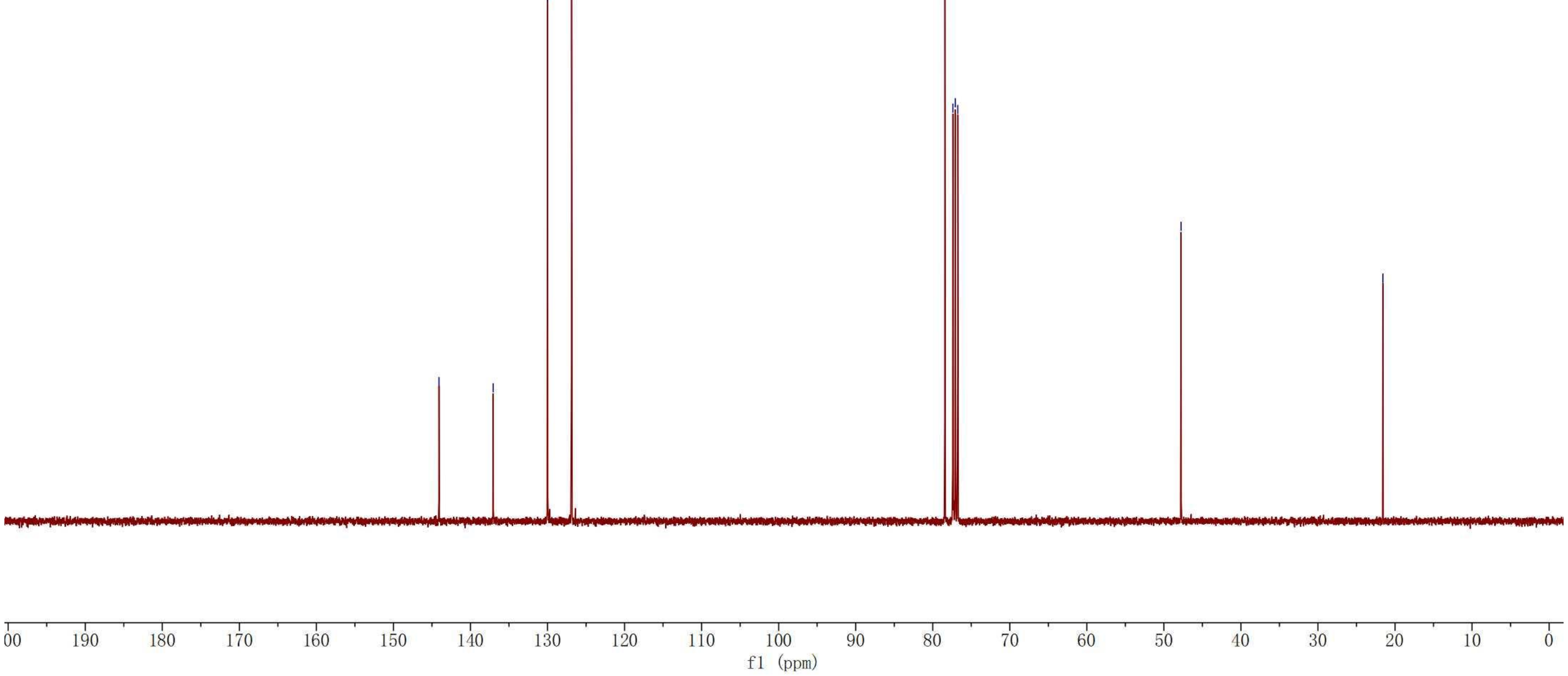

^{1}H NMR ($CDCl_3$, 400 MHz)

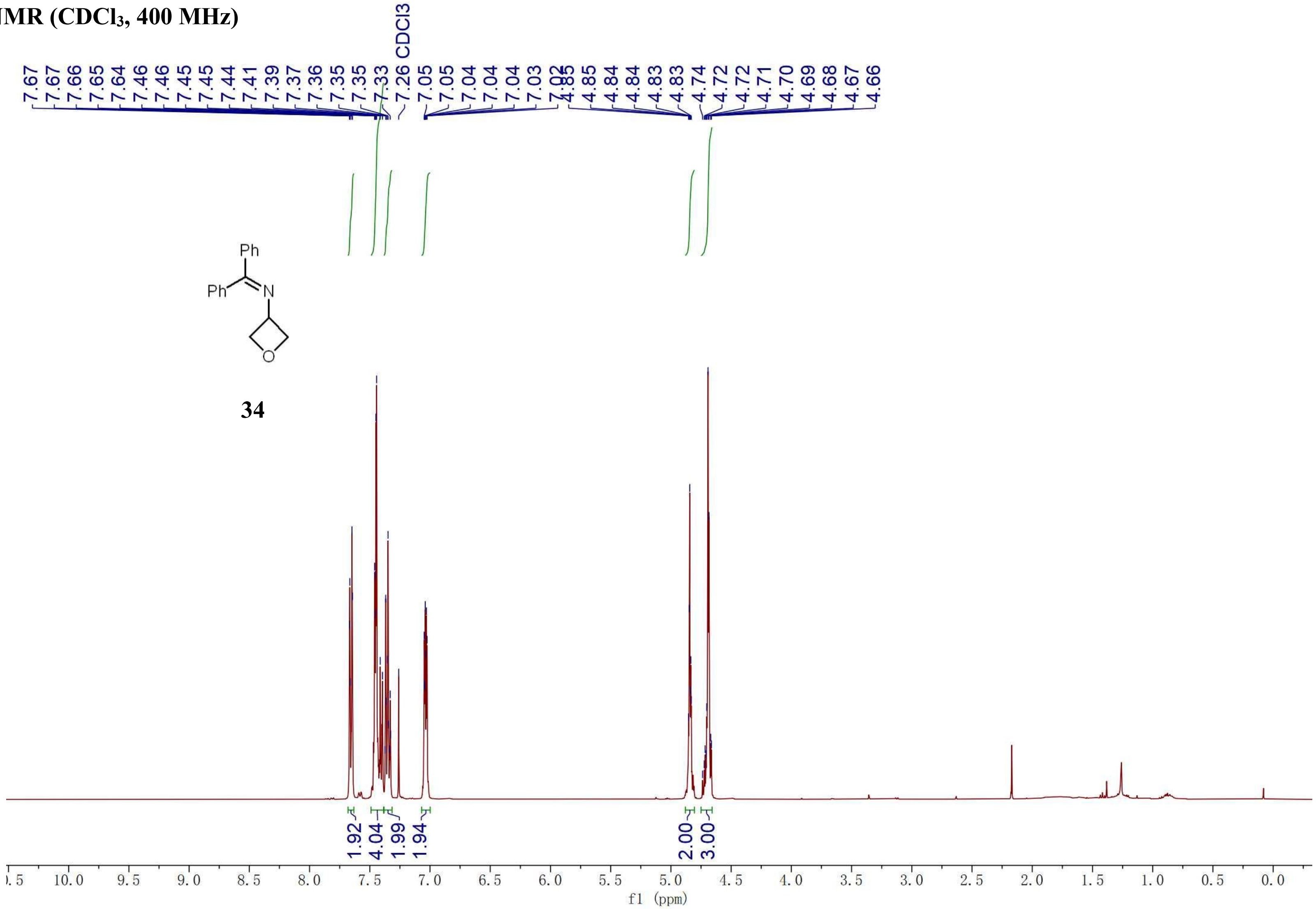

34

^{13}C NMR ($CDCl_3$, 101 MHz)

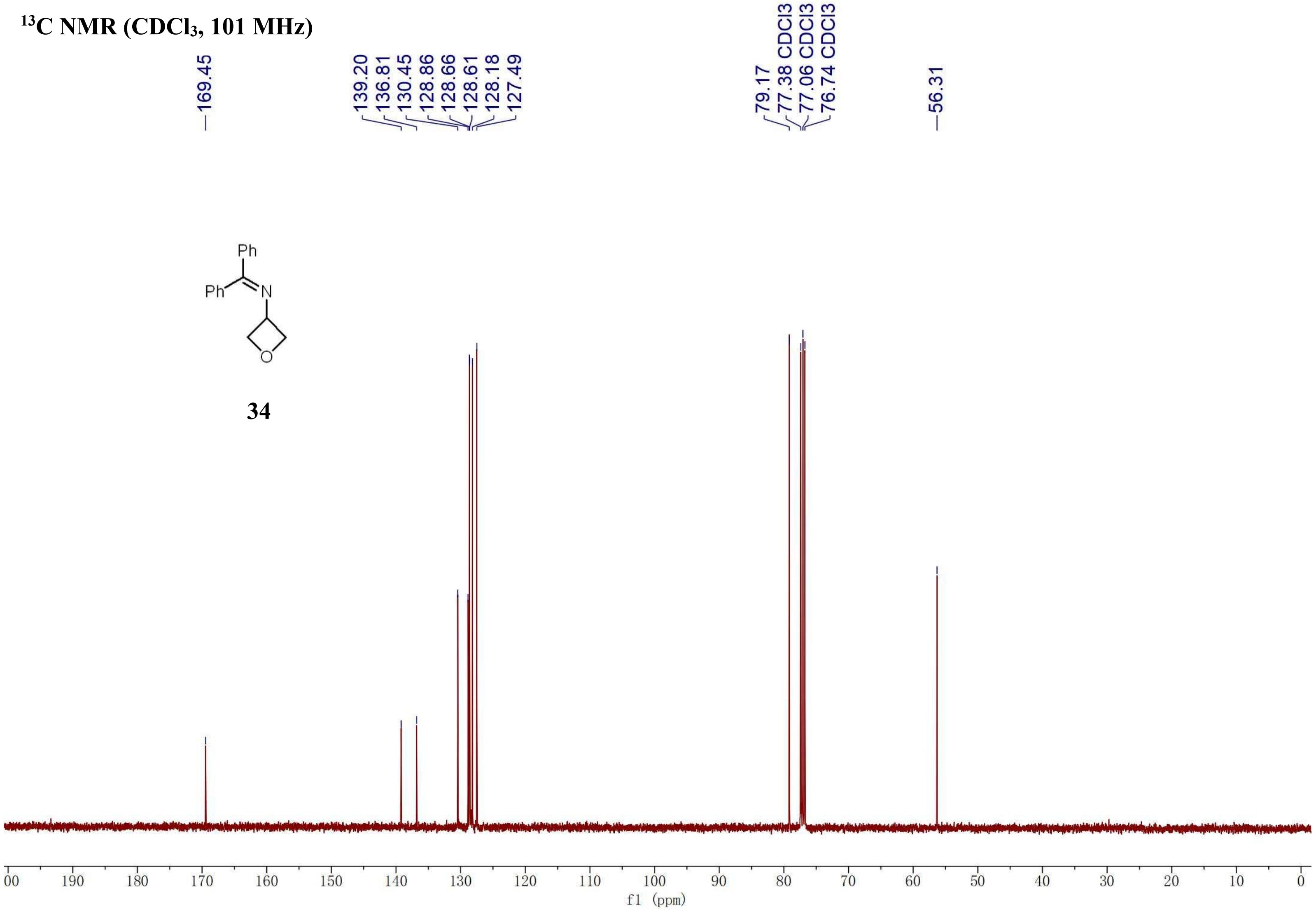

34

^{1}H NMR (CDCl$_3$, 400 MHz)

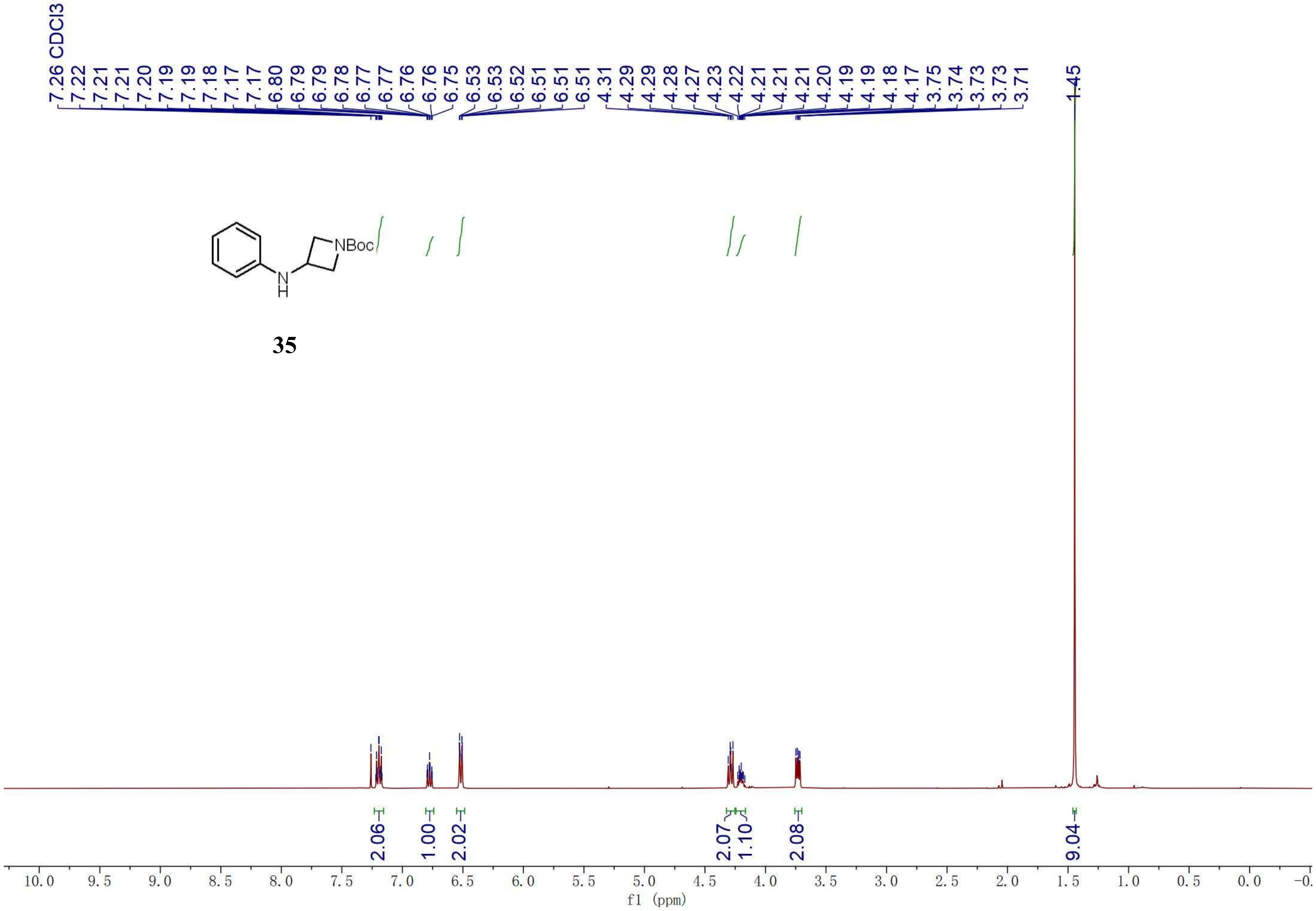

35

^{13}C NMR ($CDCl_3$, 101 MHz)

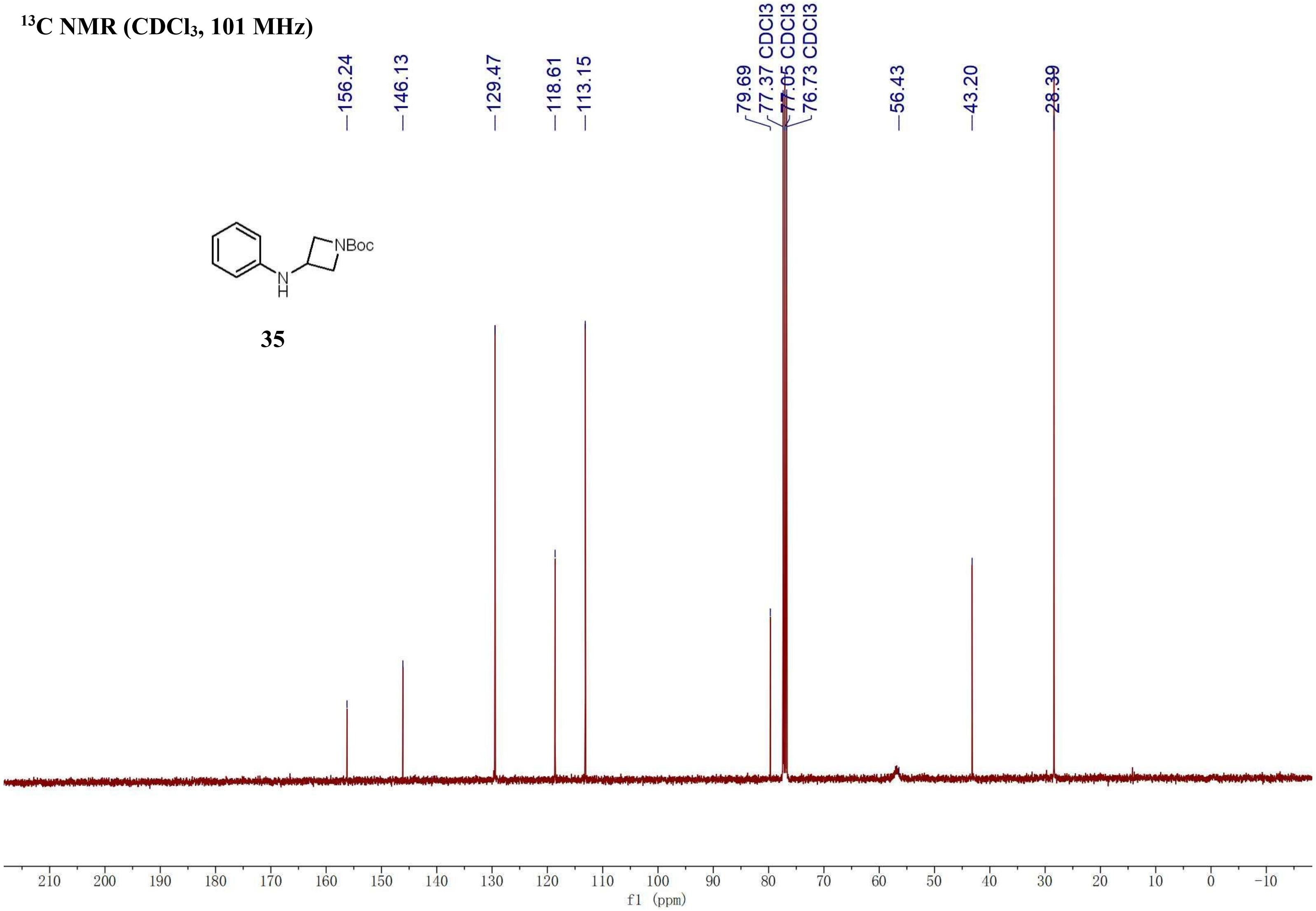

^{1}H NMR ($CDCl_3$, 400 MHz)

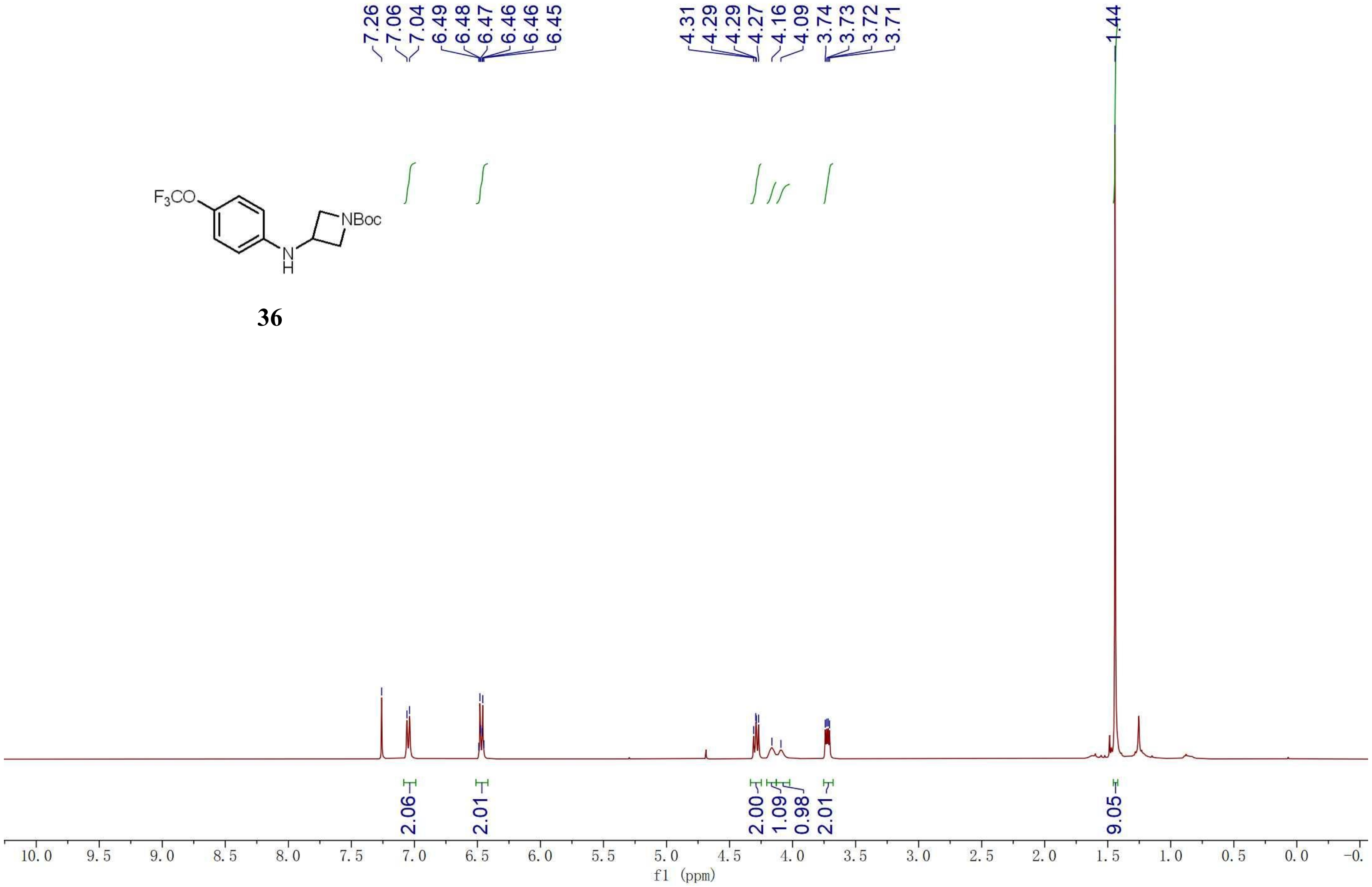

^{19}F NMR ($CDCl_3$, 376 MHz)

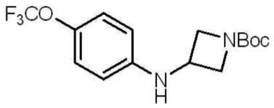

36

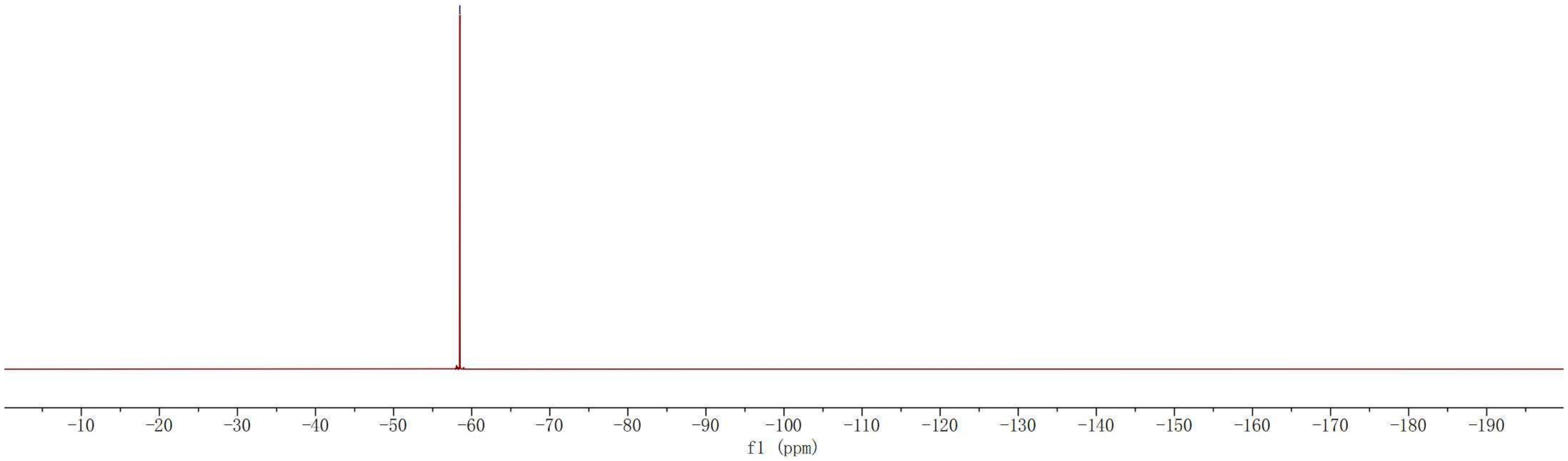

^{13}C NMR ($CDCl_3$, 101 MHz)

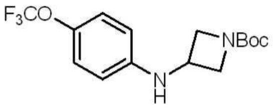

36

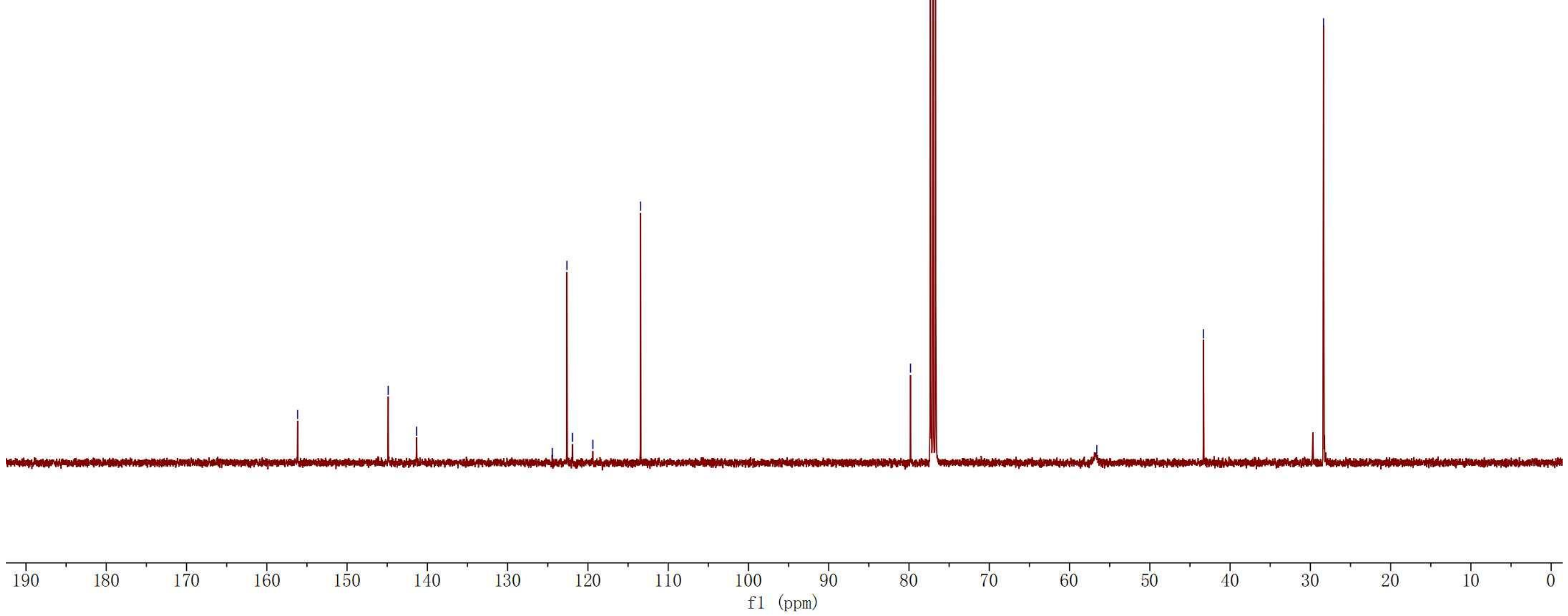

^{1}H NMR ($CDCl_3$, 400 MHz)

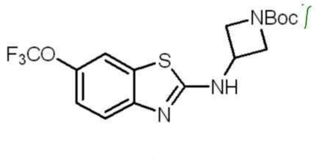

37

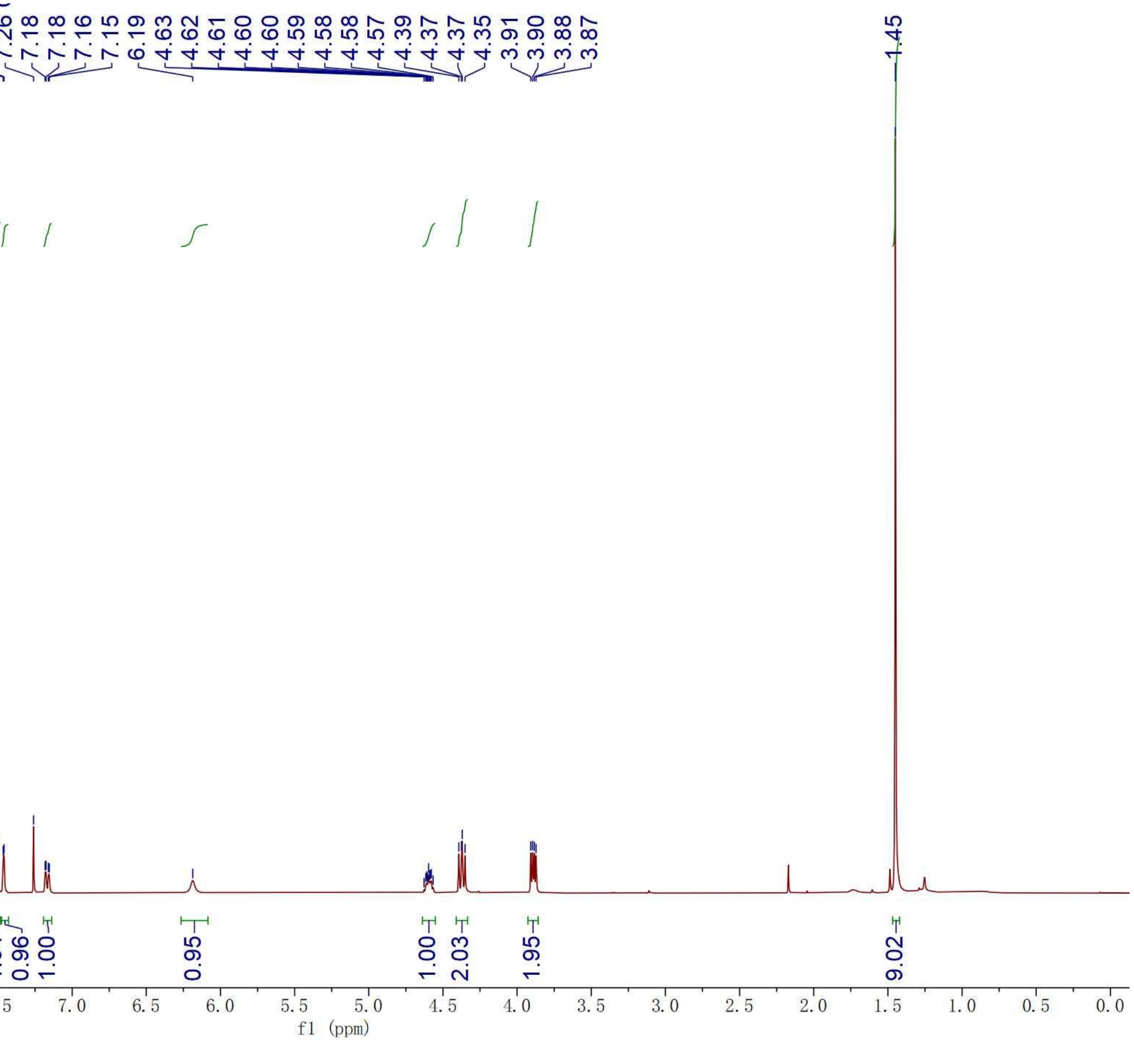

^{19}F NMR ($CDCl_3$, 376 MHz)

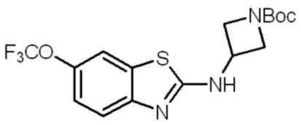

37

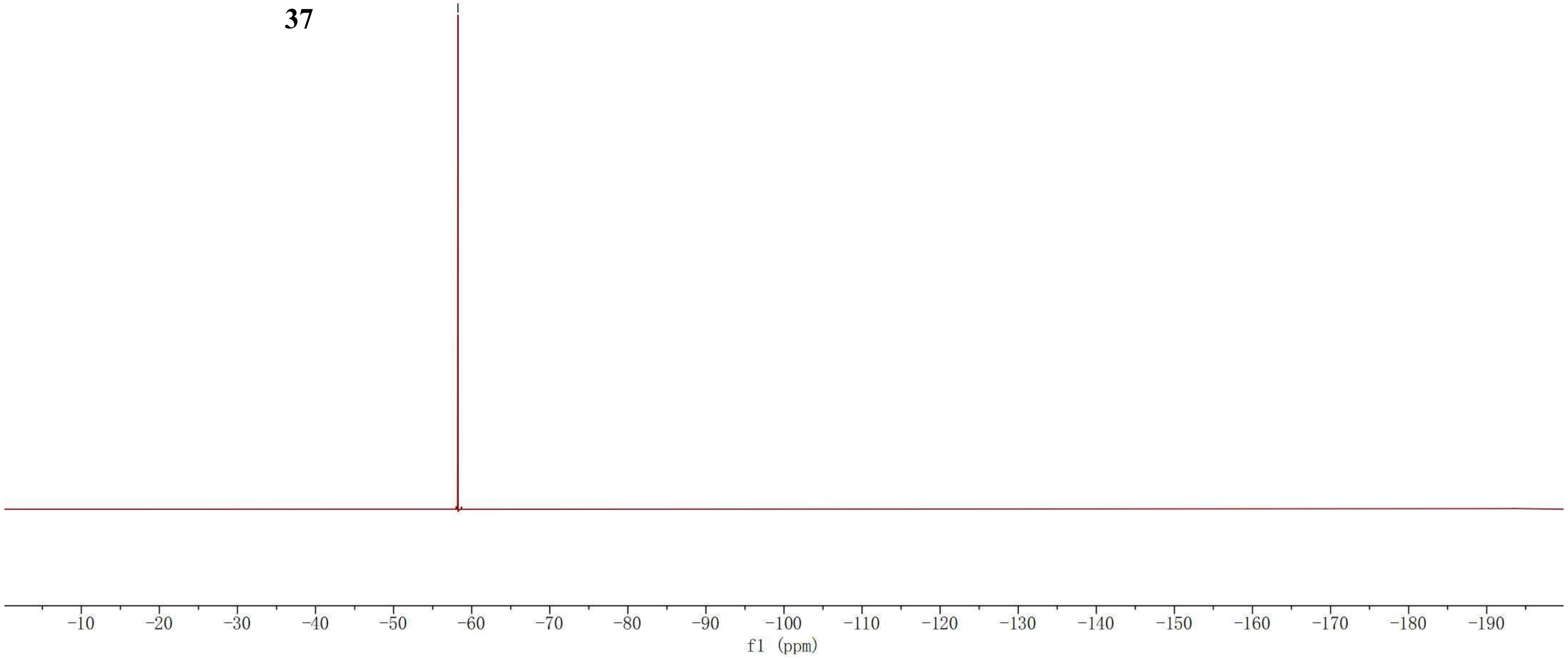

^{13}C NMR ($CDCl_3$, 101 MHz)

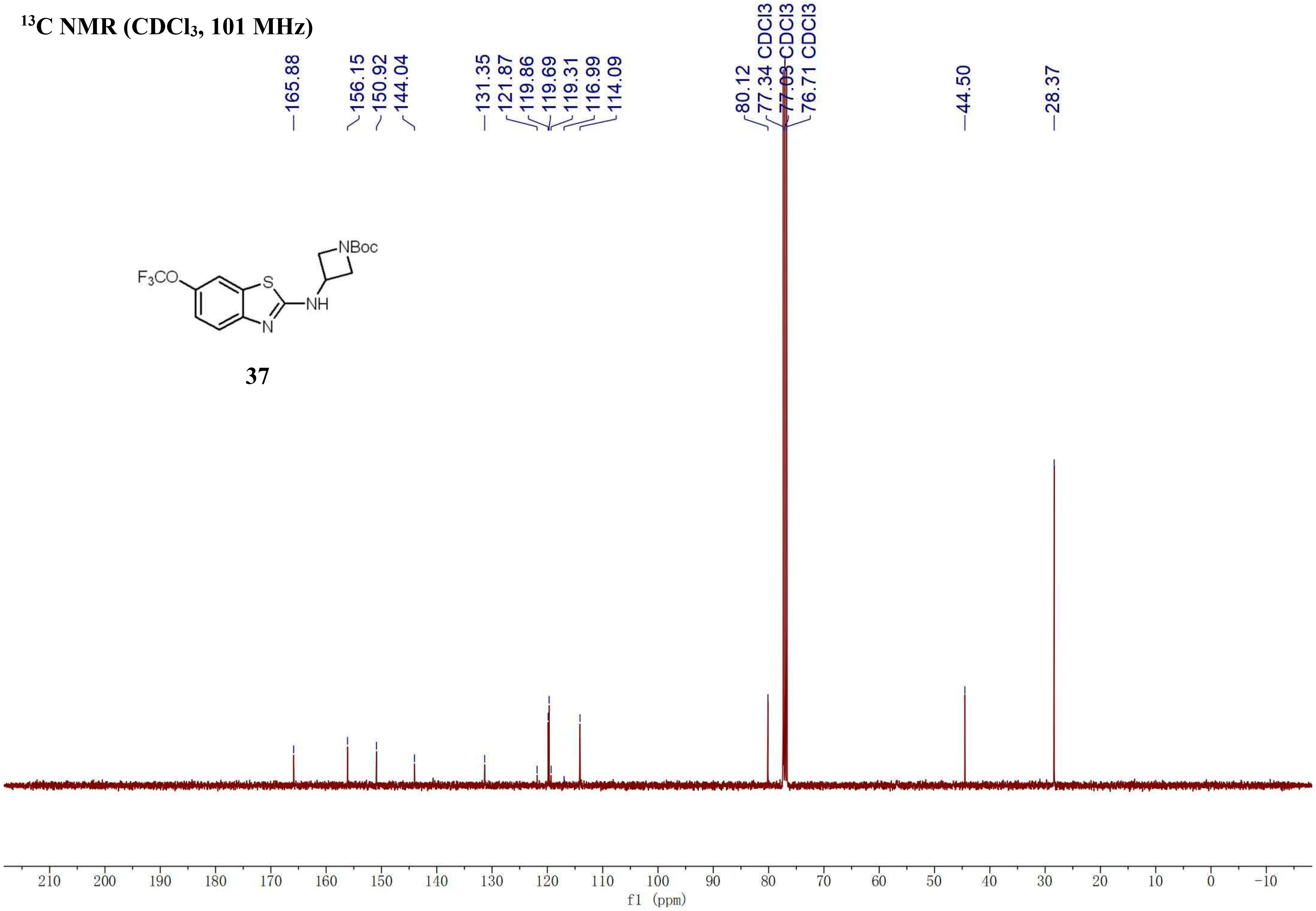

37

^{1}H NMR ($CDCl_3$, 400 MHz)

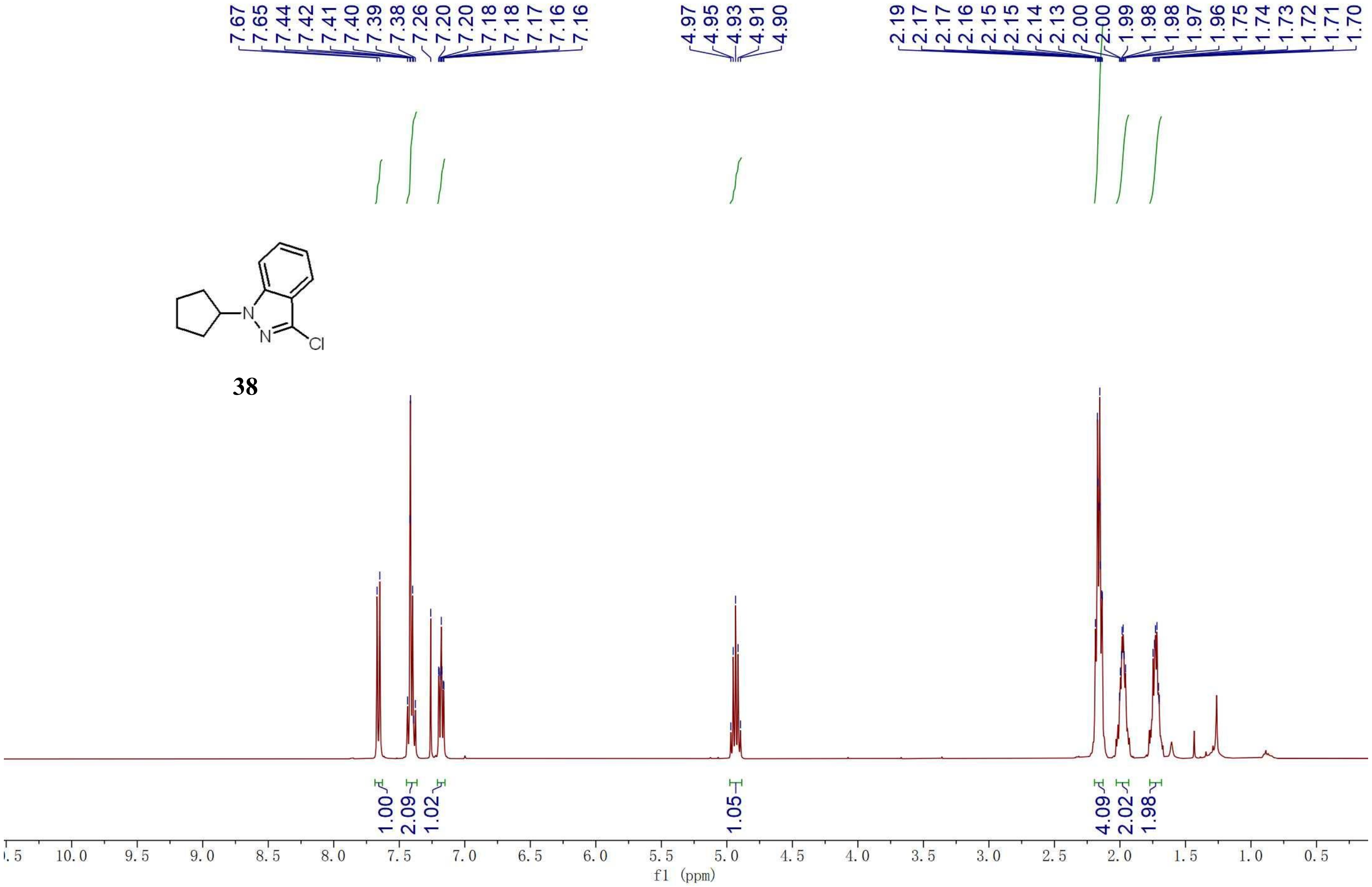

38

^{13}C NMR ($CDCl_3$, 101 MHz)

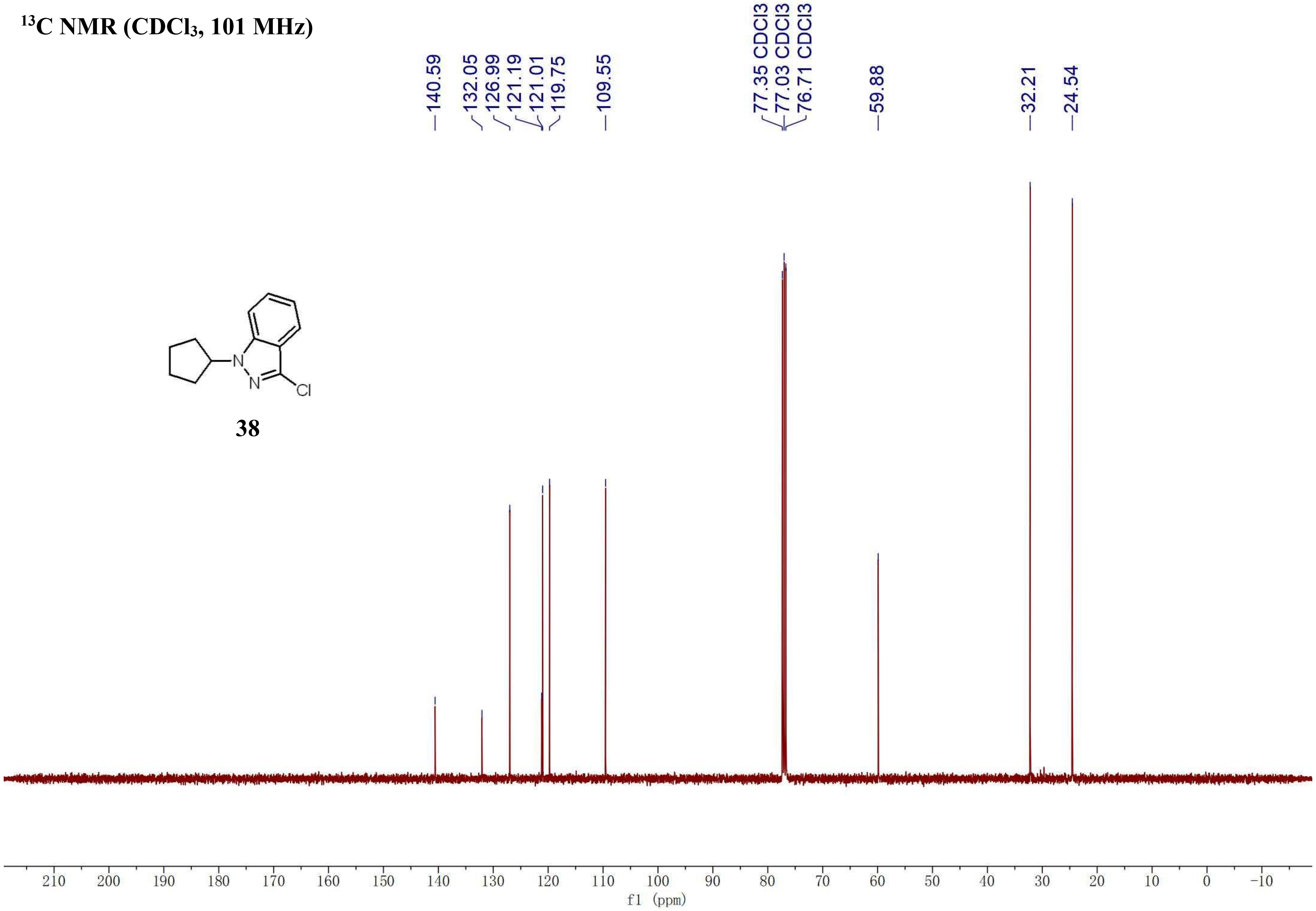

38

^{1}H NMR (CDCl$_3$, 400 MHz)

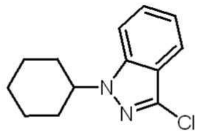

39

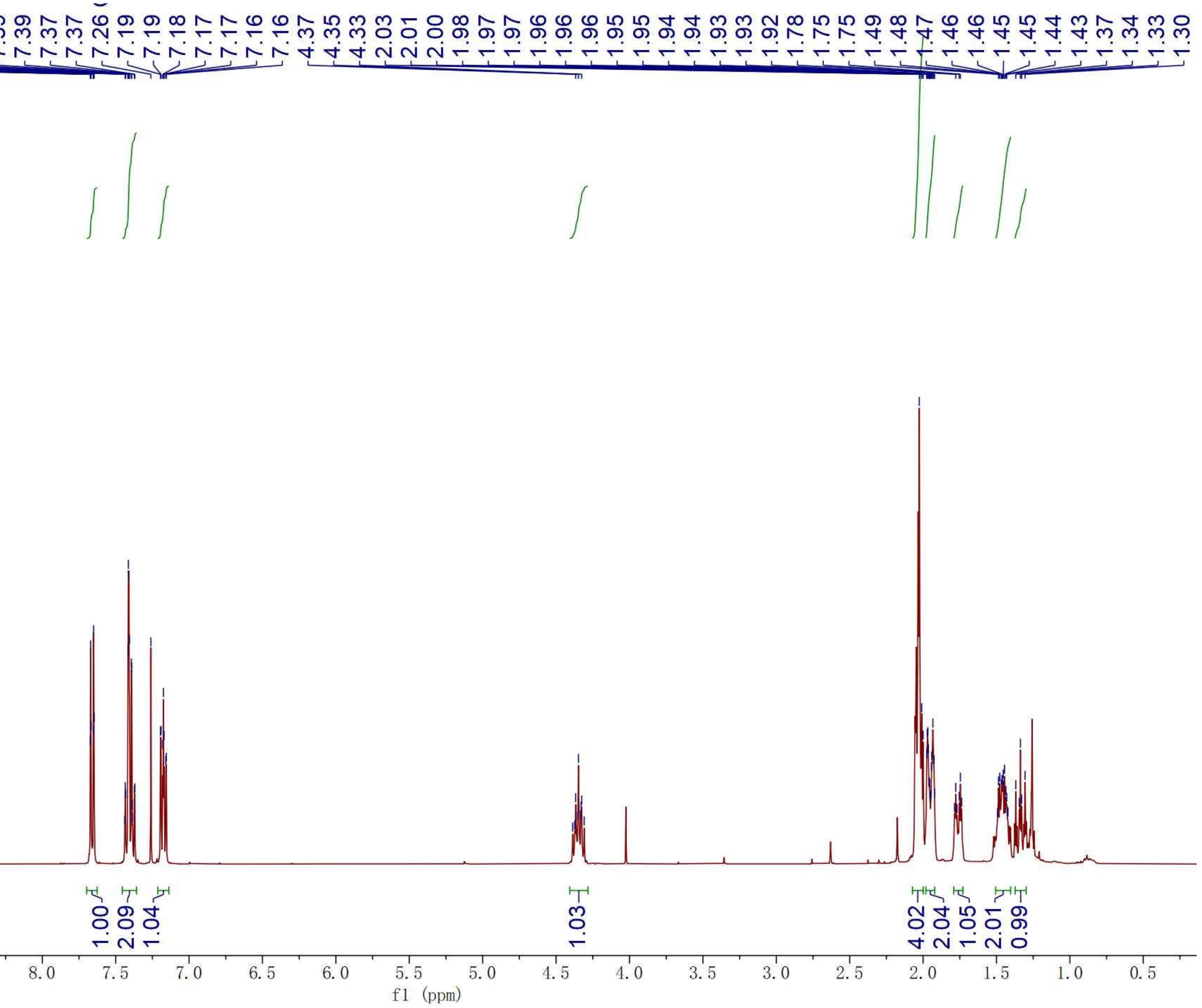

^{13}C NMR (CDCl$_3$, 101 MHz)

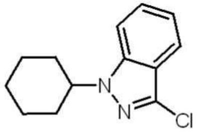

39

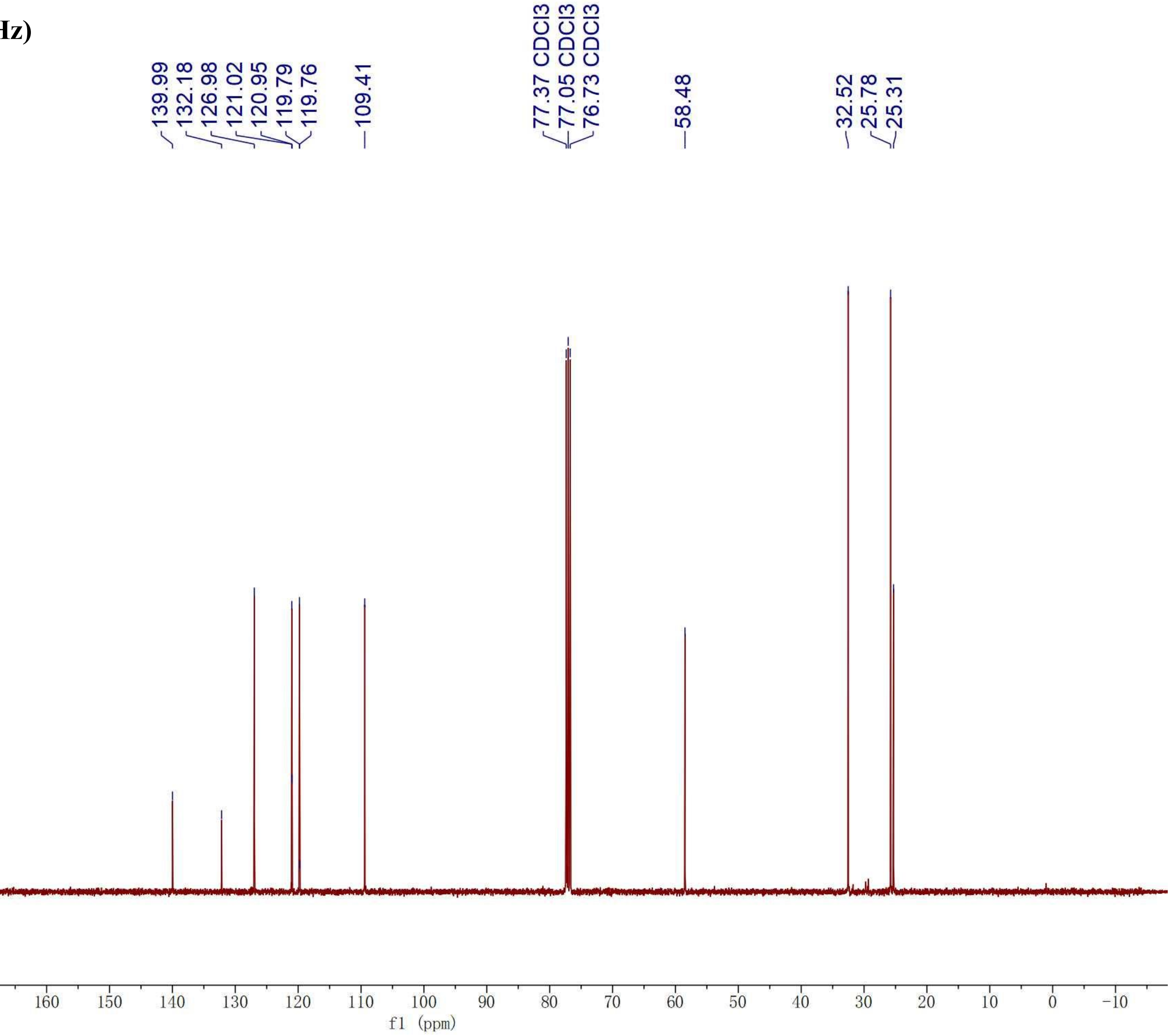

^{1}H NMR ($CDCl_3$, 400 MHz)

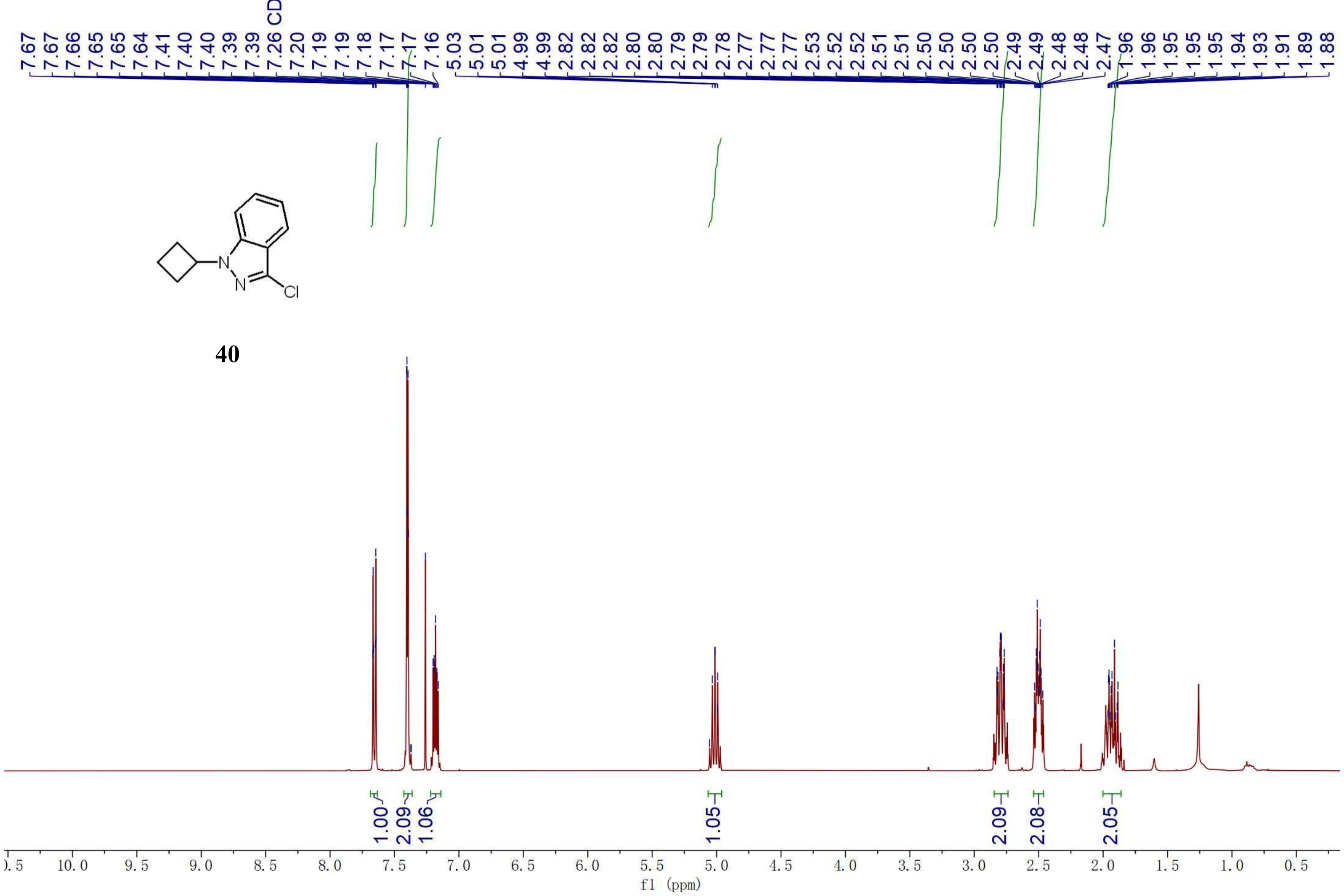

40

^{13}C NMR (CDCl3, 101 MHz)

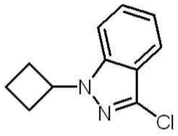

40

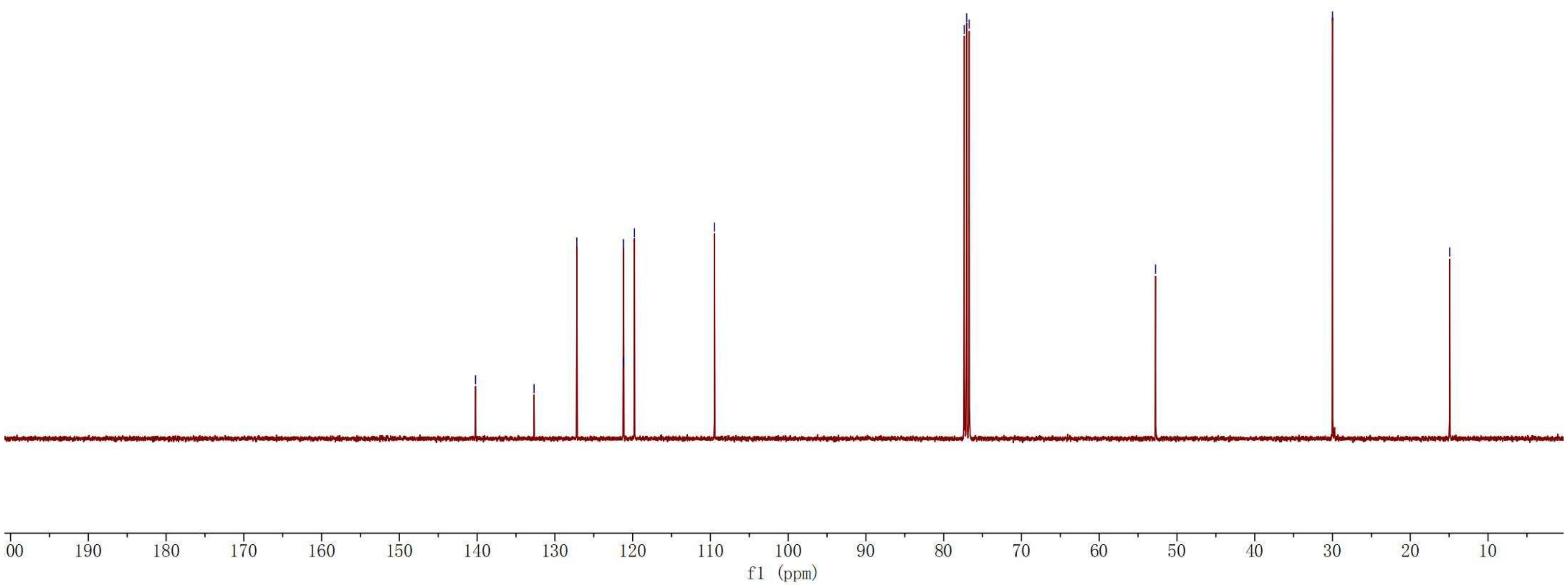

^{1}H NMR ($CDCl_3$, 400 MHz)

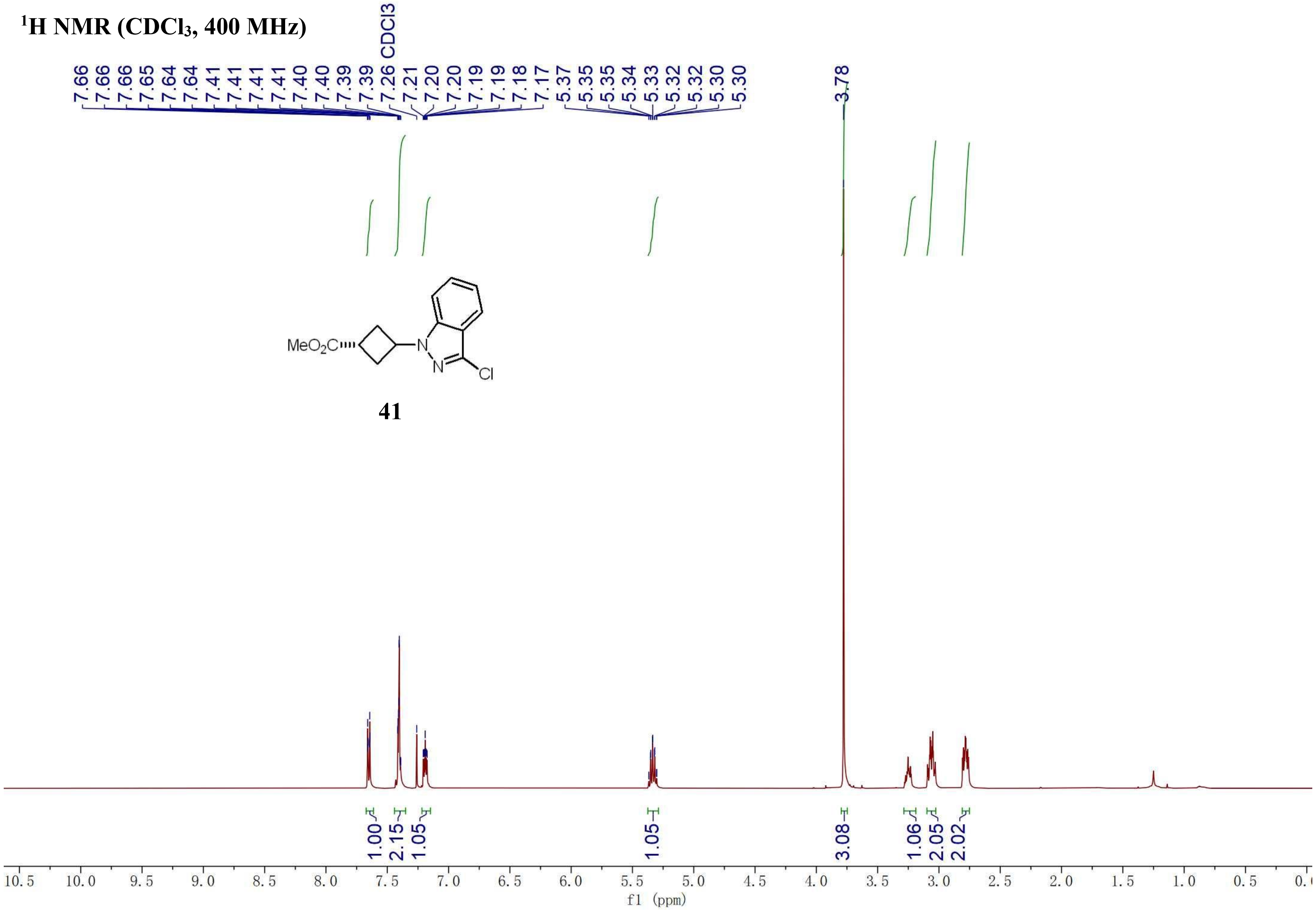

41

^{13}C NMR ($CDCl_3$, 101 MHz)

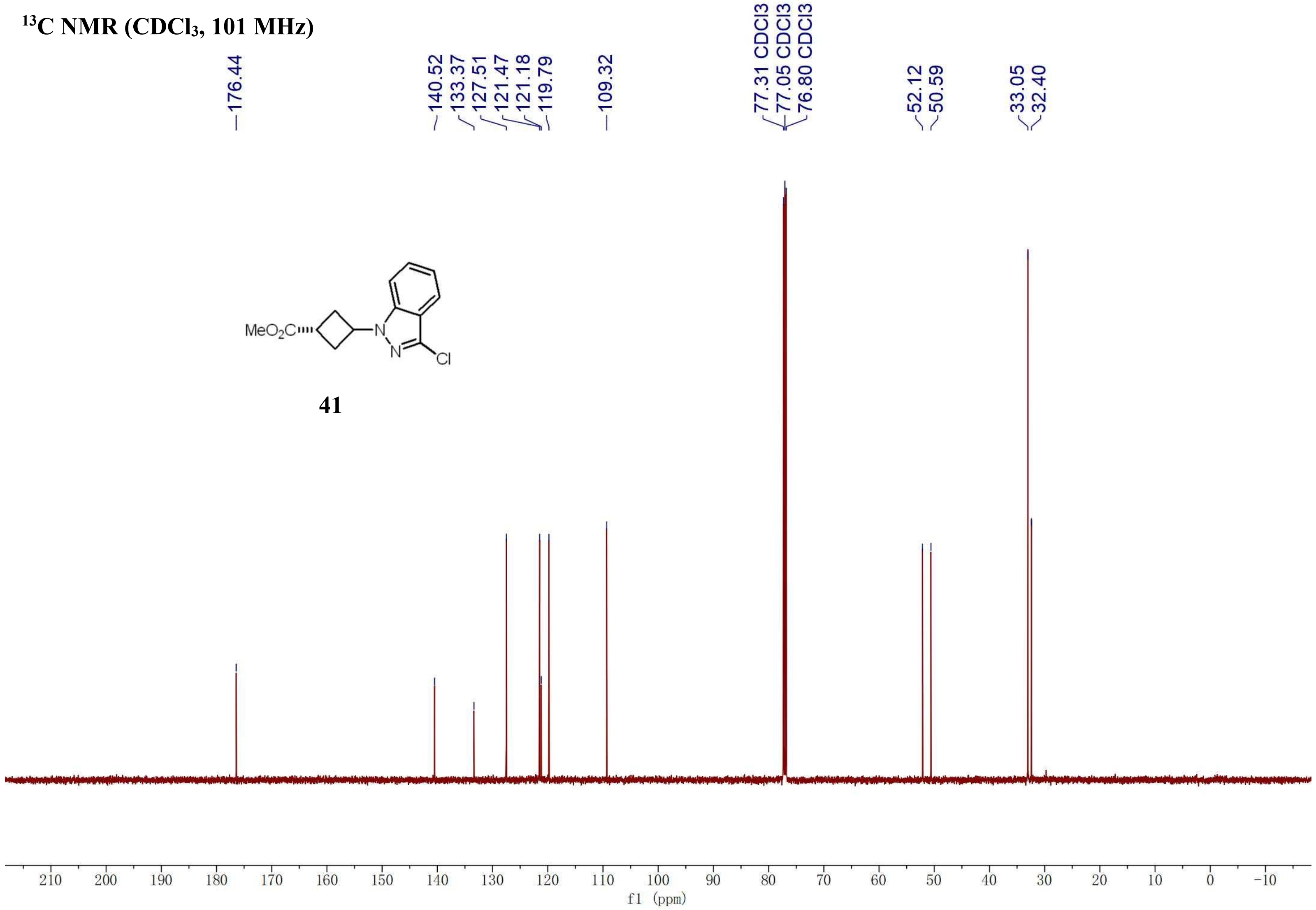

^{1}H NMR ($CDCl_3$, 400 MHz)

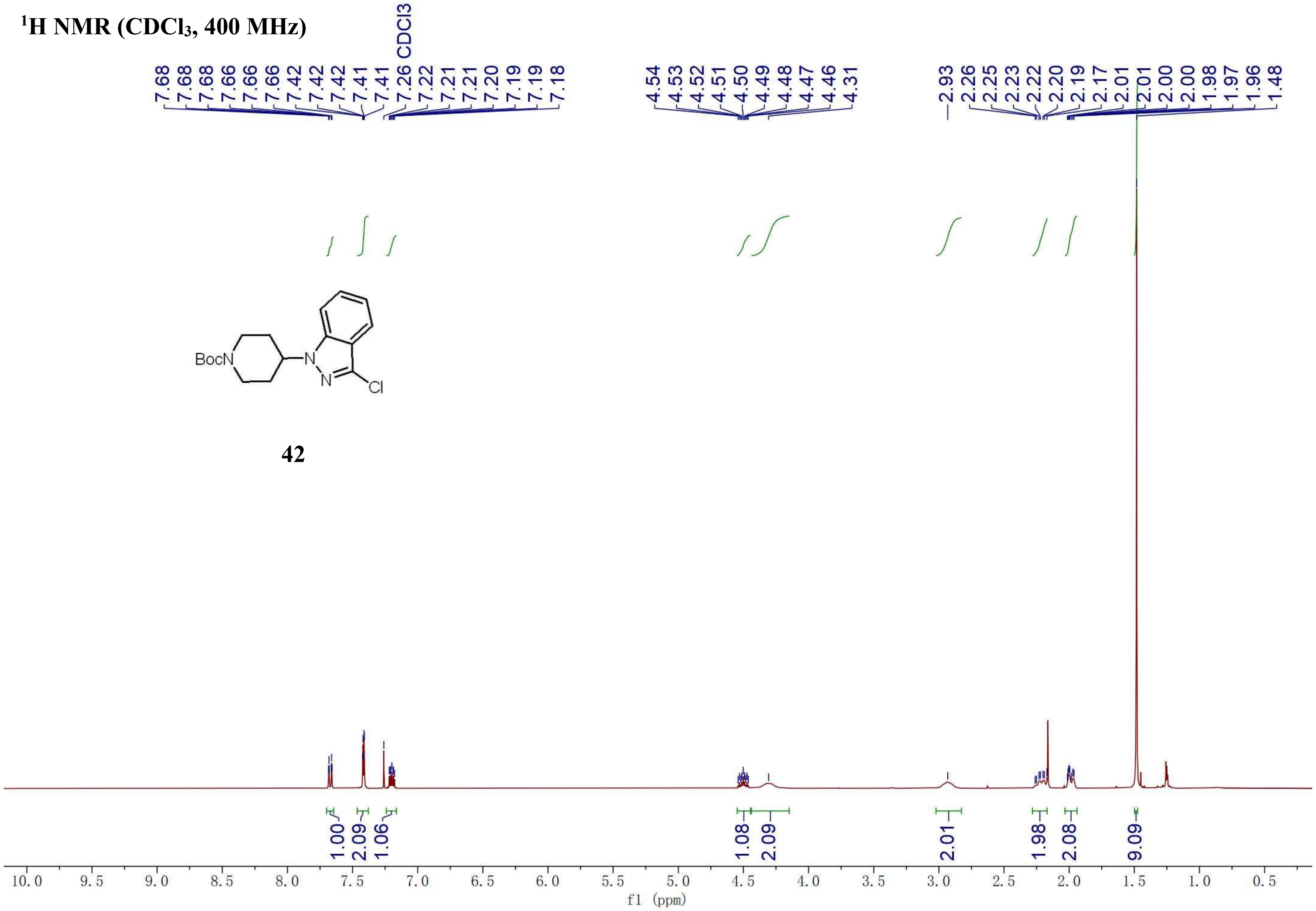

42

^{13}C NMR (CDCl$_3$, 101 MHz)

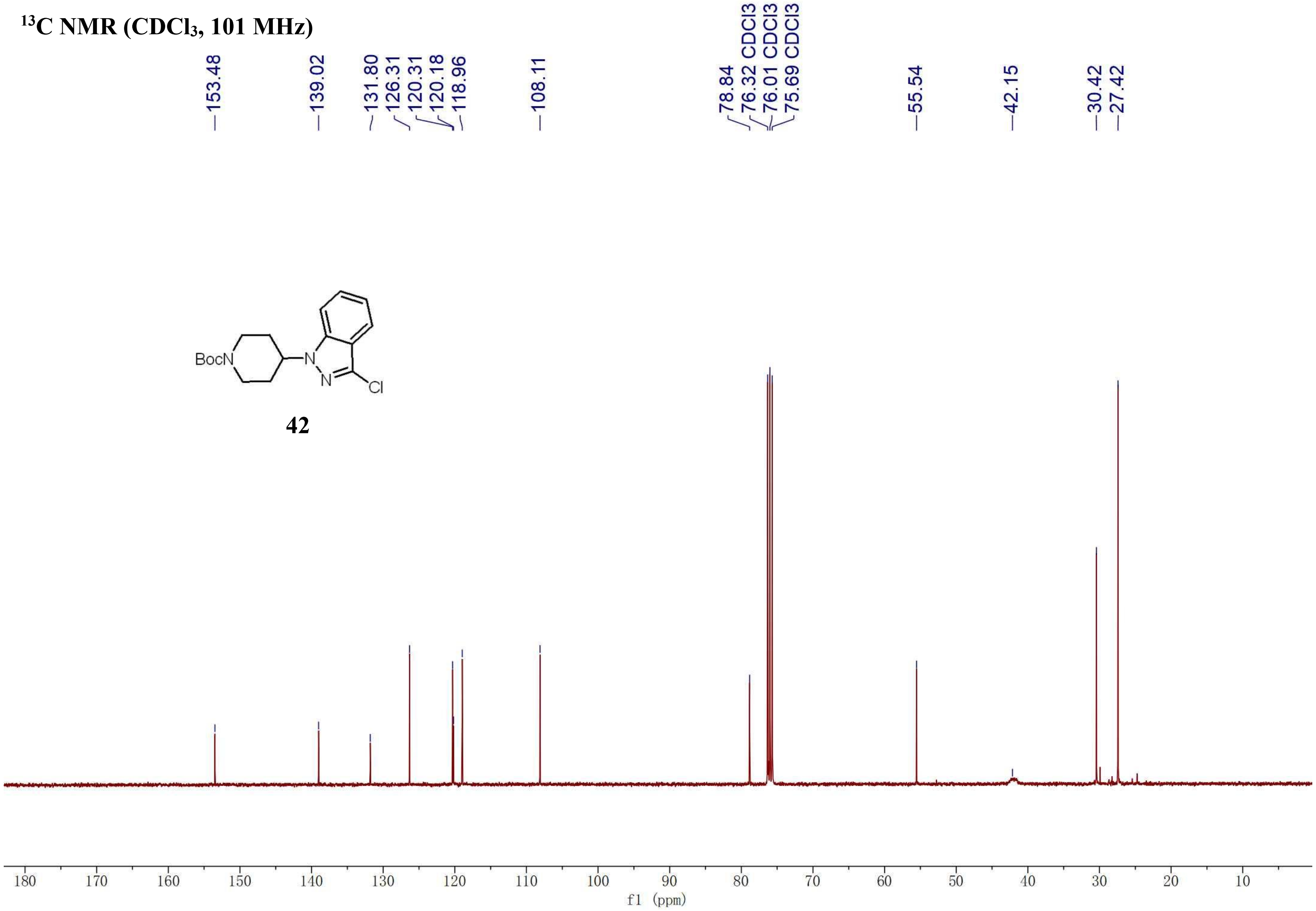

42

^{1}H NMR ($CDCl_3$, 400 MHz)

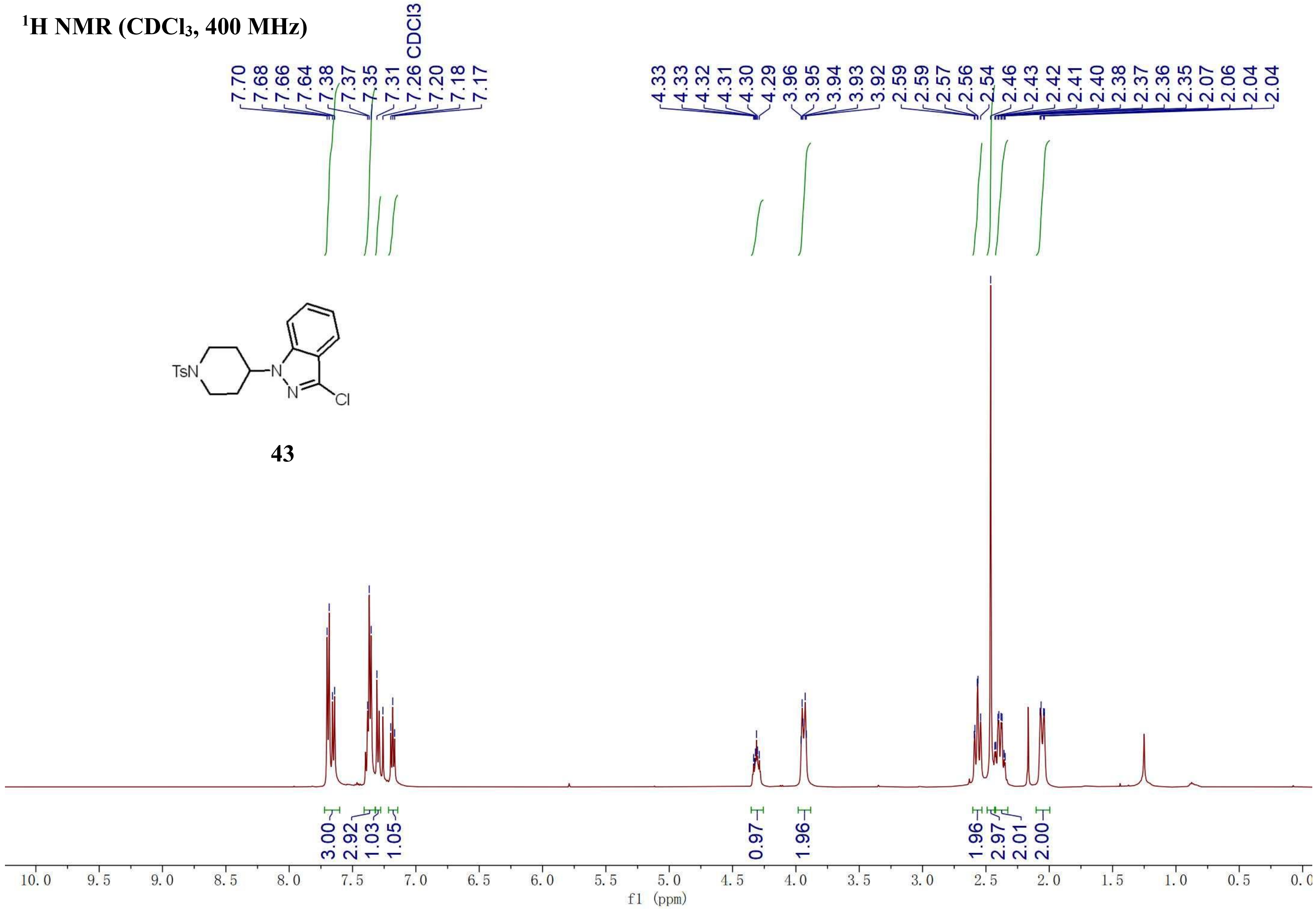

43

^{13}C NMR (CDCl$_3$, 101 MHz)

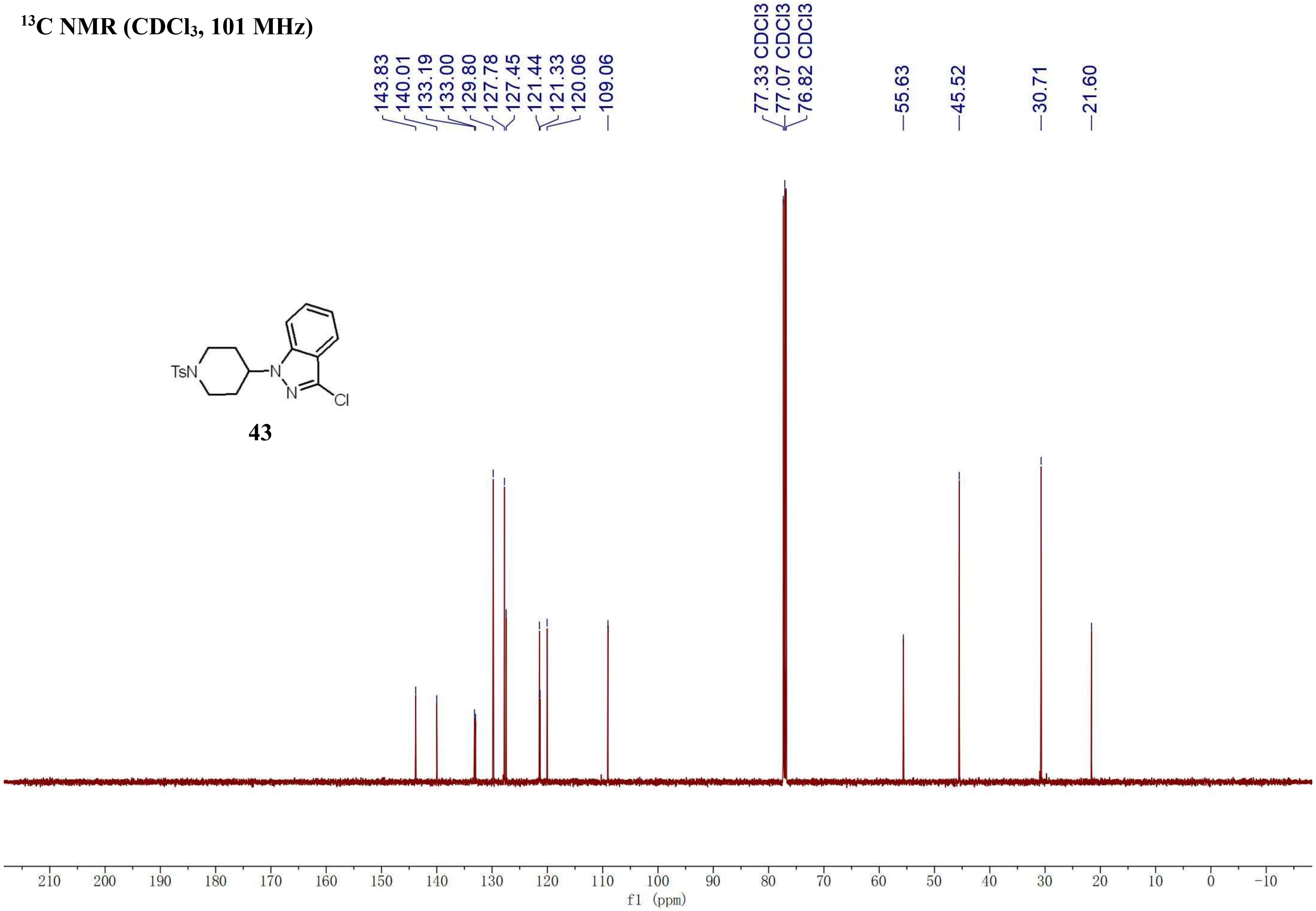

43

^{1}H NMR (CDCl$_3$, 400 MHz)

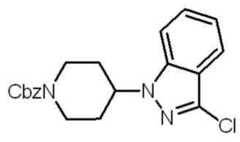

44

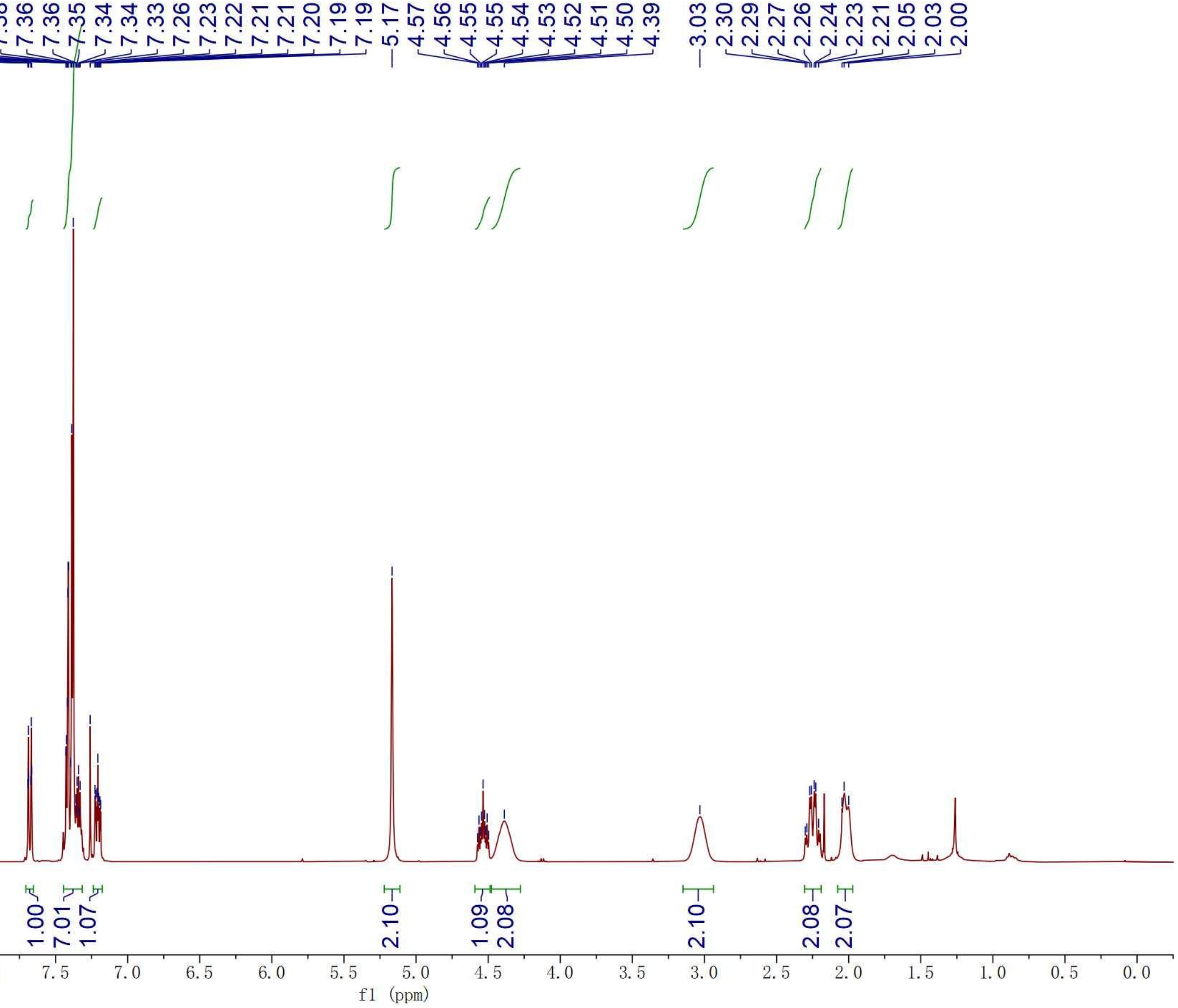

^{13}C NMR ($CDCl_3$, 101 MHz)

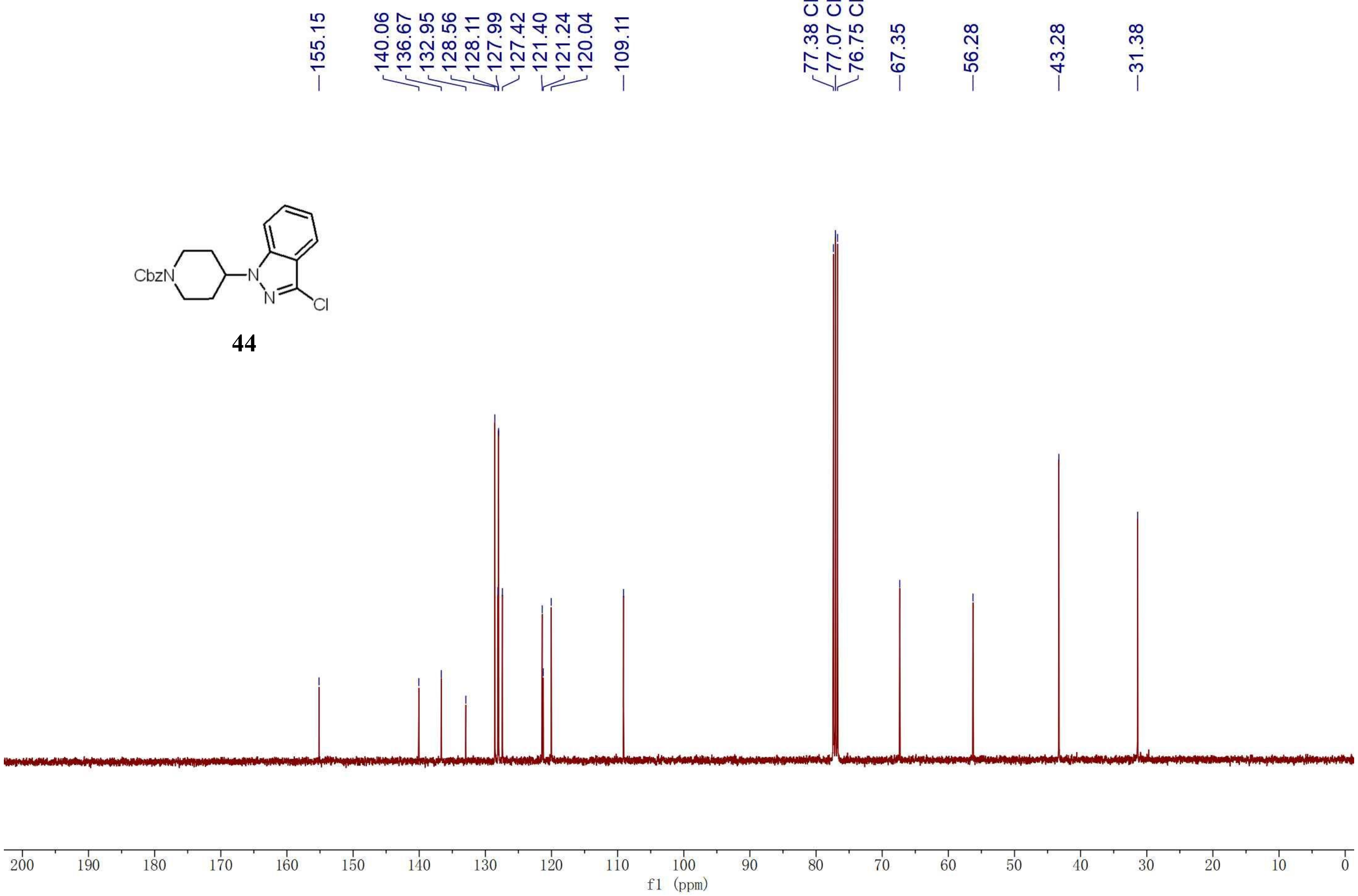

44

^{1}H NMR ($CDCl_3$, 400 MHz)

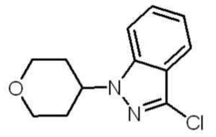

45

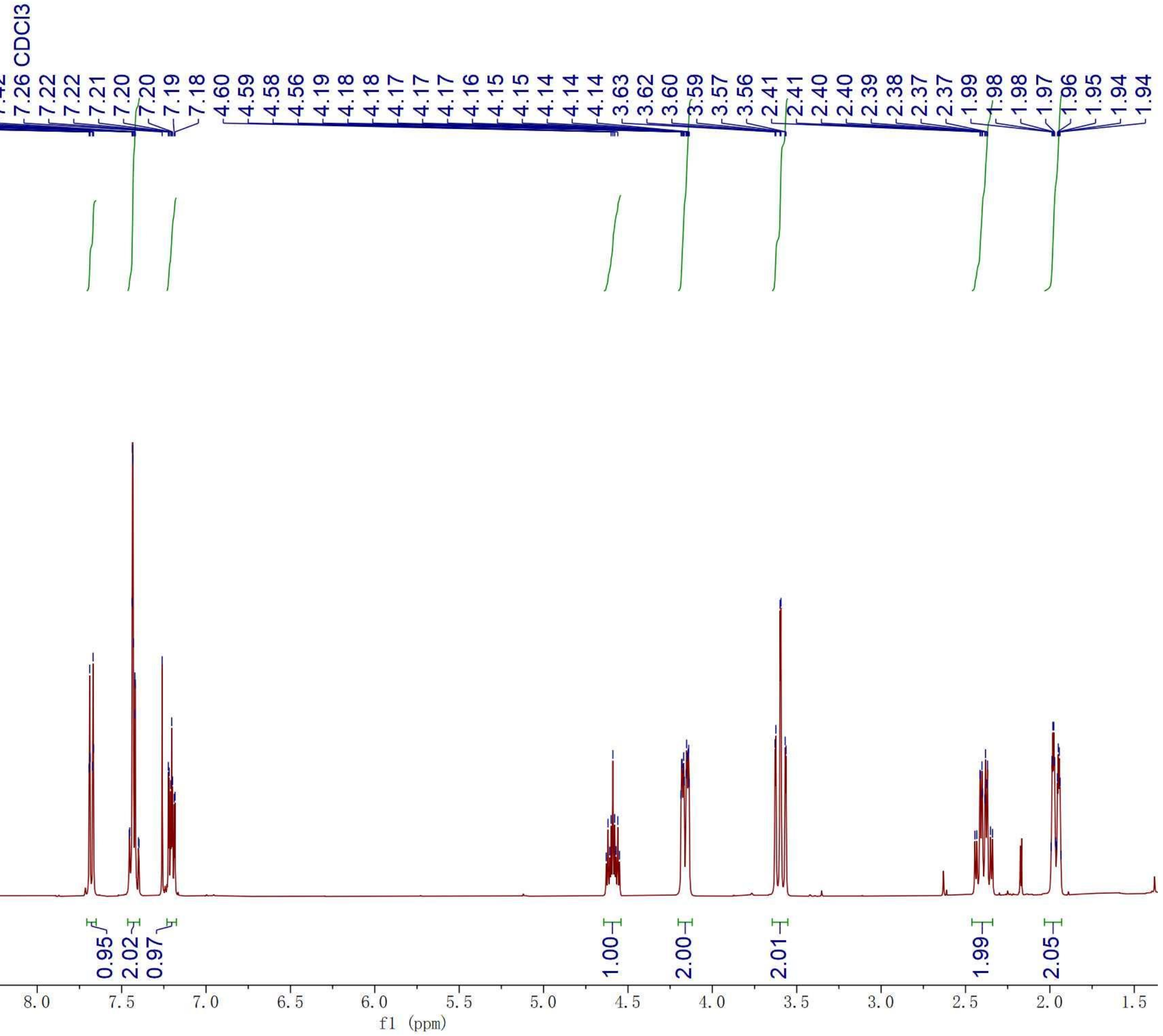

^{13}C NMR ($CDCl_3$, 101 MHz)

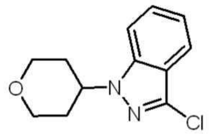

45

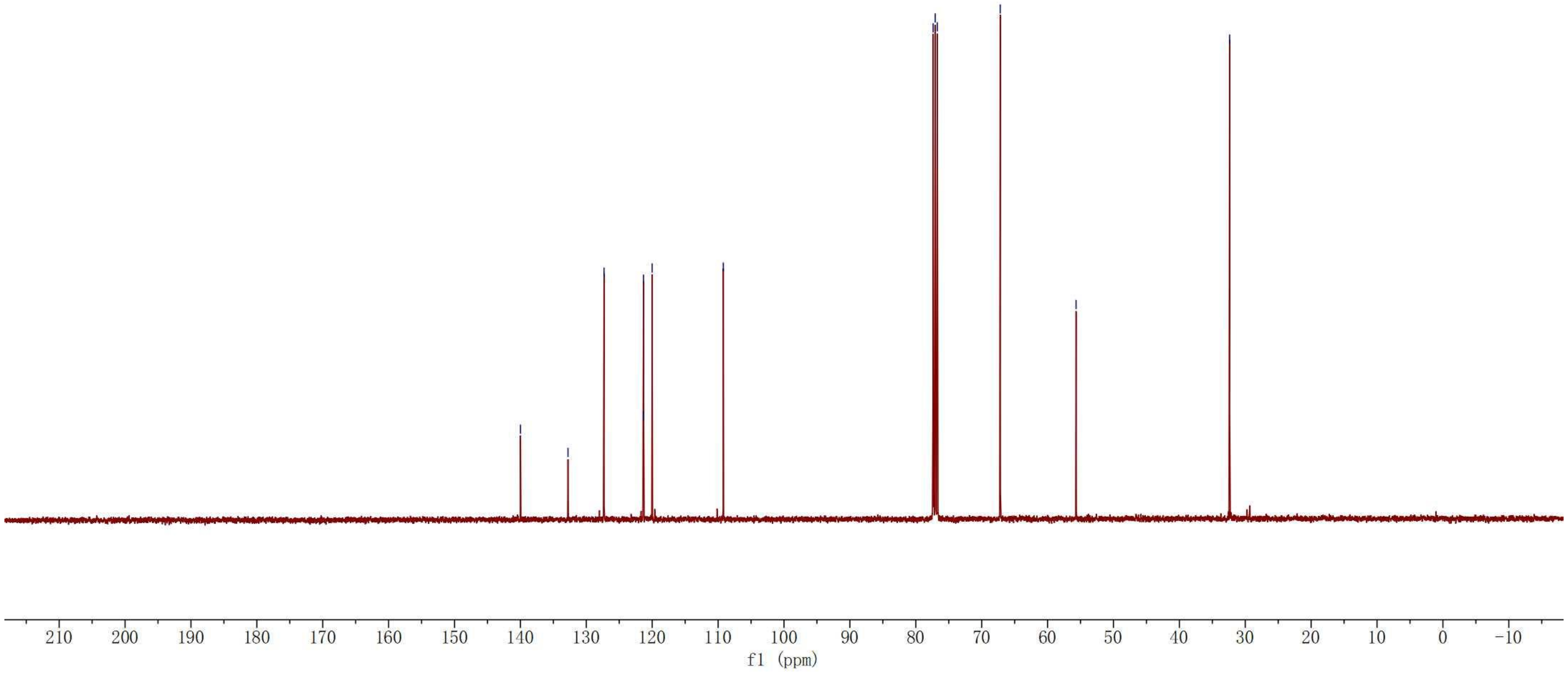

^{1}H NMR ($CDCl_3$, 400 MHz)

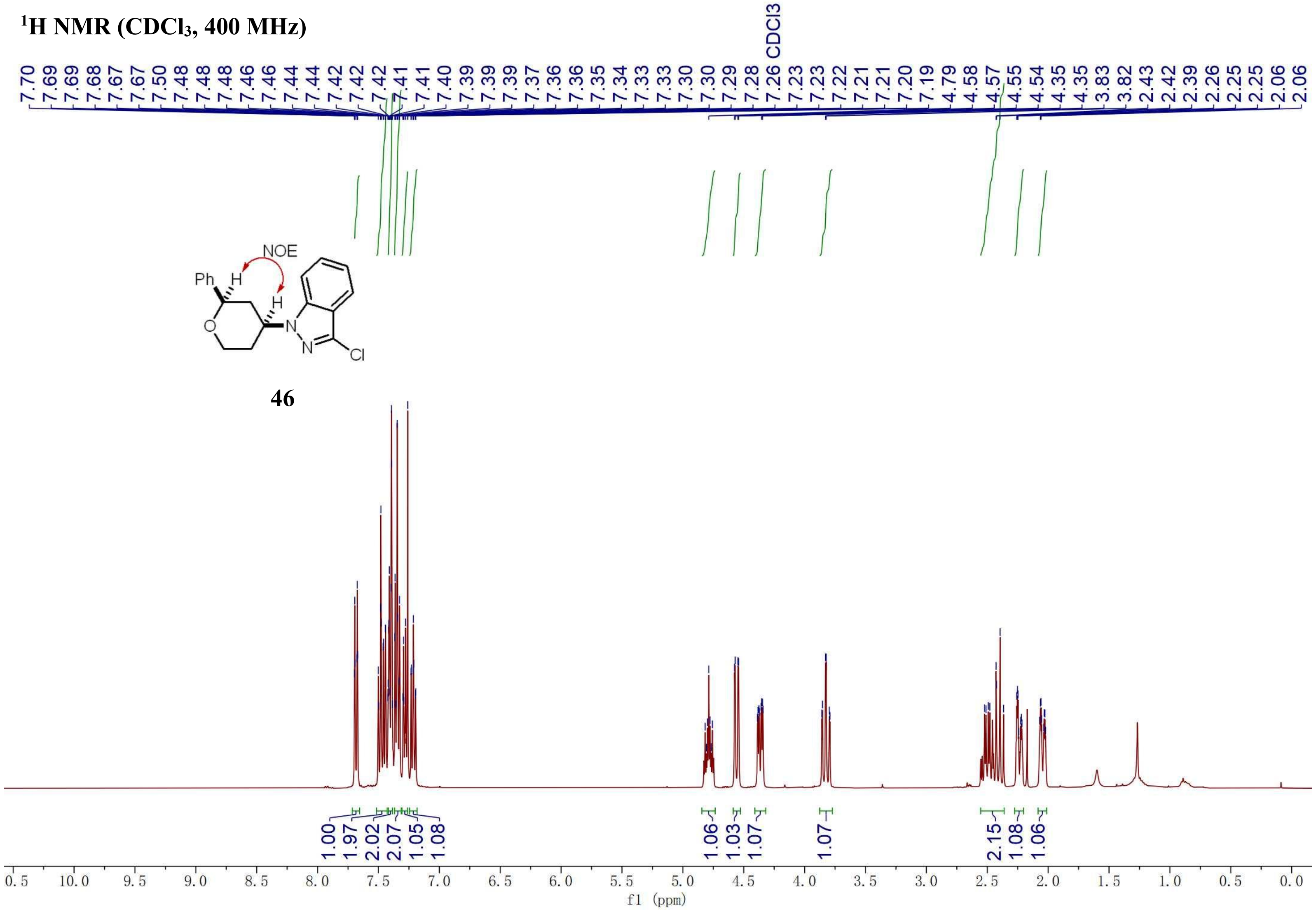

^{13}C NMR ($CDCl_3$, 101 MHz)

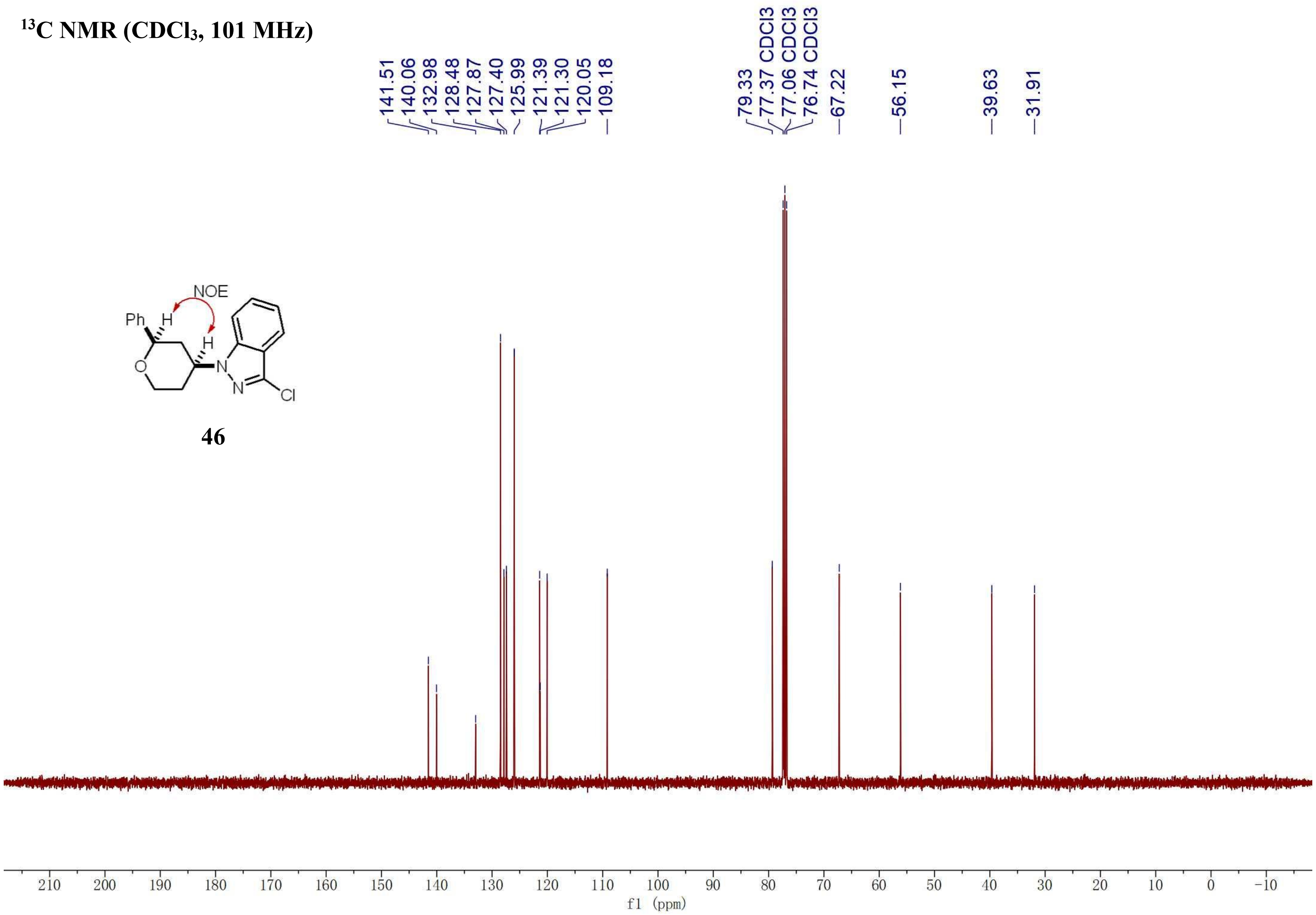

46

^{1}H-^{1}H NOESY

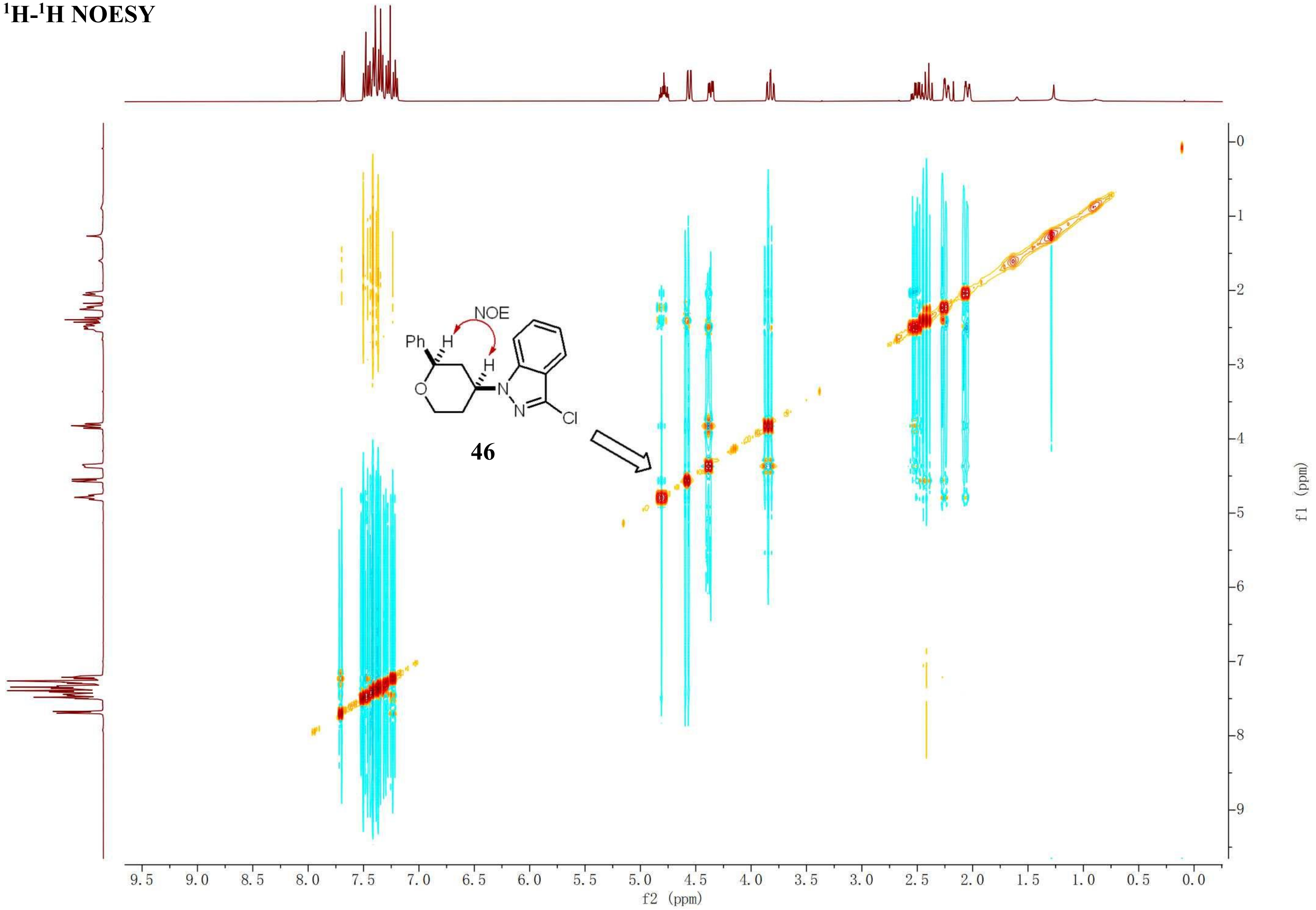
NOE
Ph
H
H
O
N
N
Cl
46
f2 (ppm)
f1 (ppm)
9.5 9.0 8.5 8.0 7.5 7.0 6.5 6.0 5.5 5.0 4.5 4.0 3.5 3.0 2.5 2.0 1.5 1.0 0.5 0.0
0 1 2 3 4 5 6 7 8 9

^{1}H NMR ($CDCl_3$, 400 MHz)

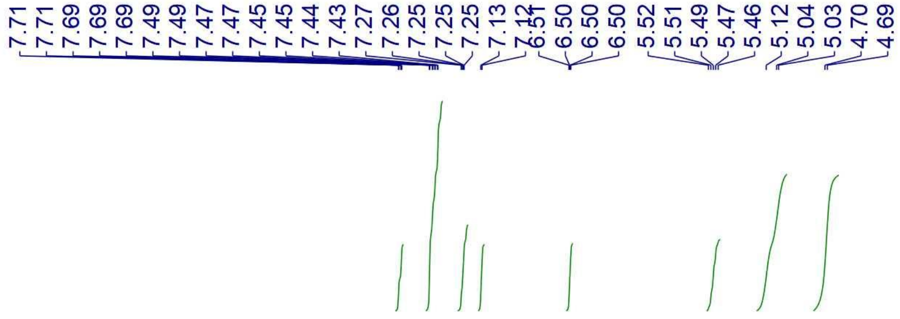

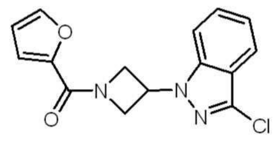

47

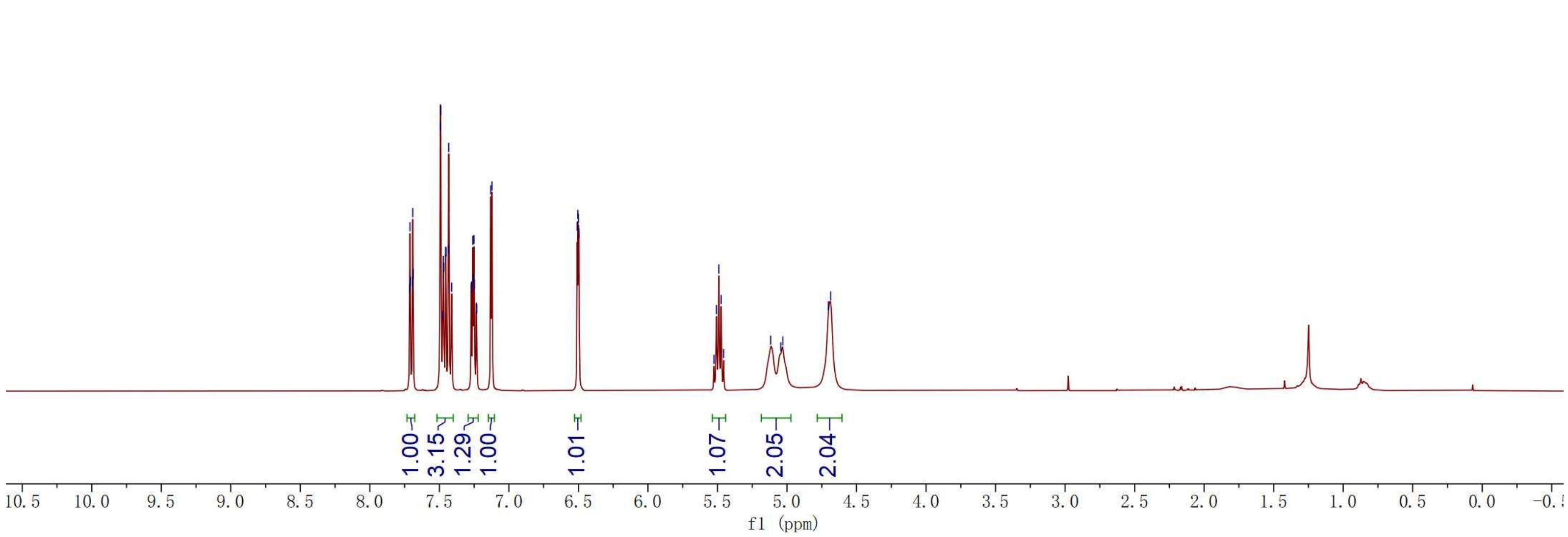

^{13}C NMR ($CDCl_3$, 101 MHz)

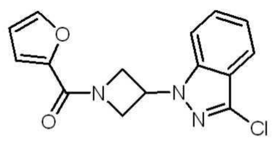

47

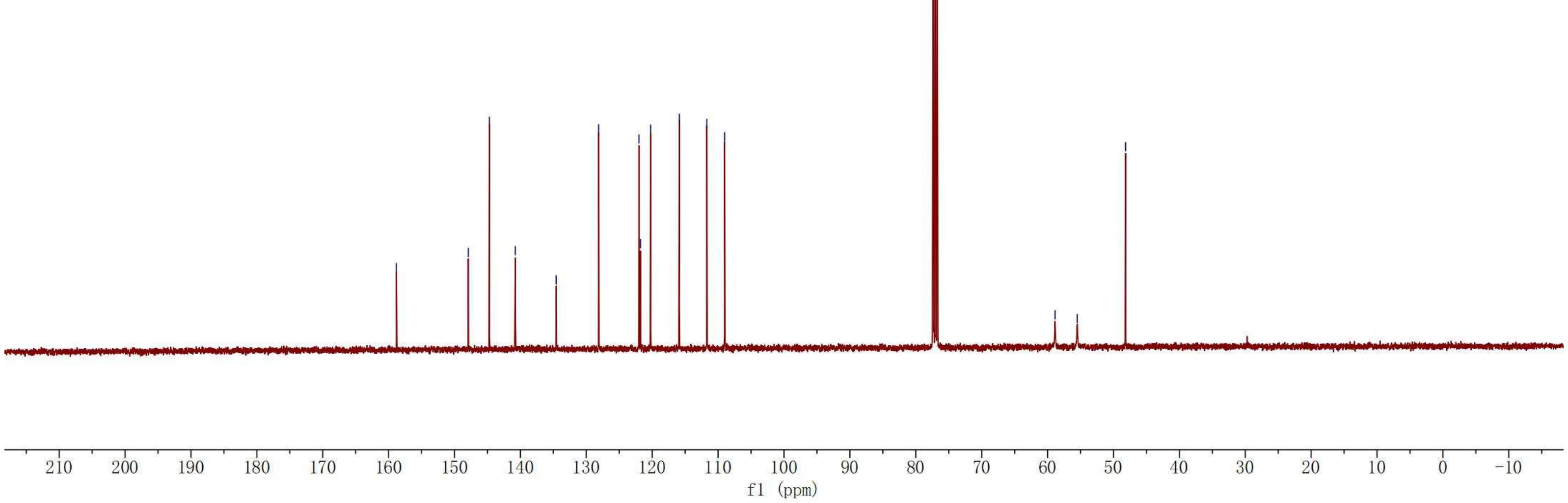

^{1}H NMR ($CDCl_3$, 400 MHz)

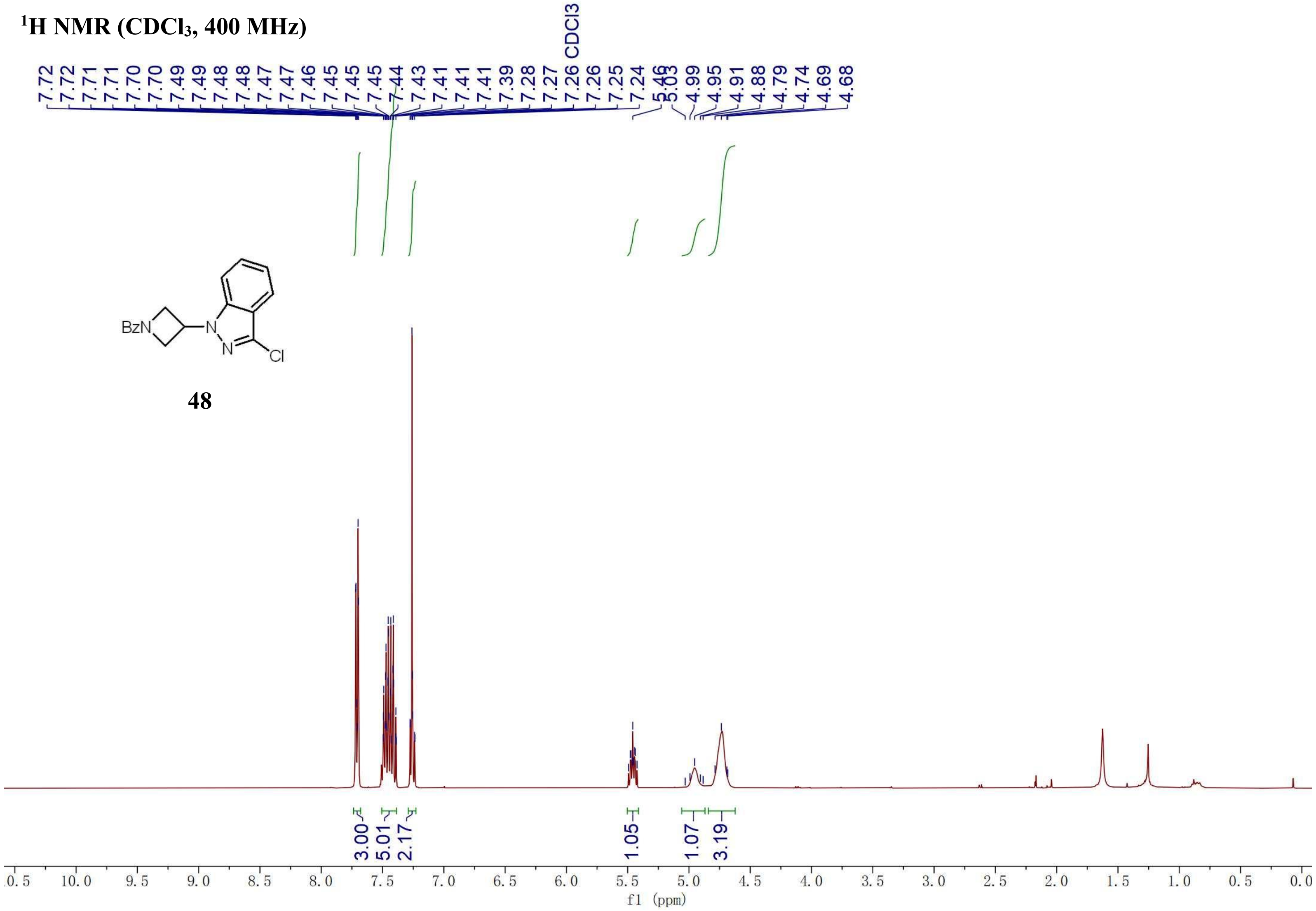

48

^{13}C NMR ($CDCl_3$, 101 MHz)

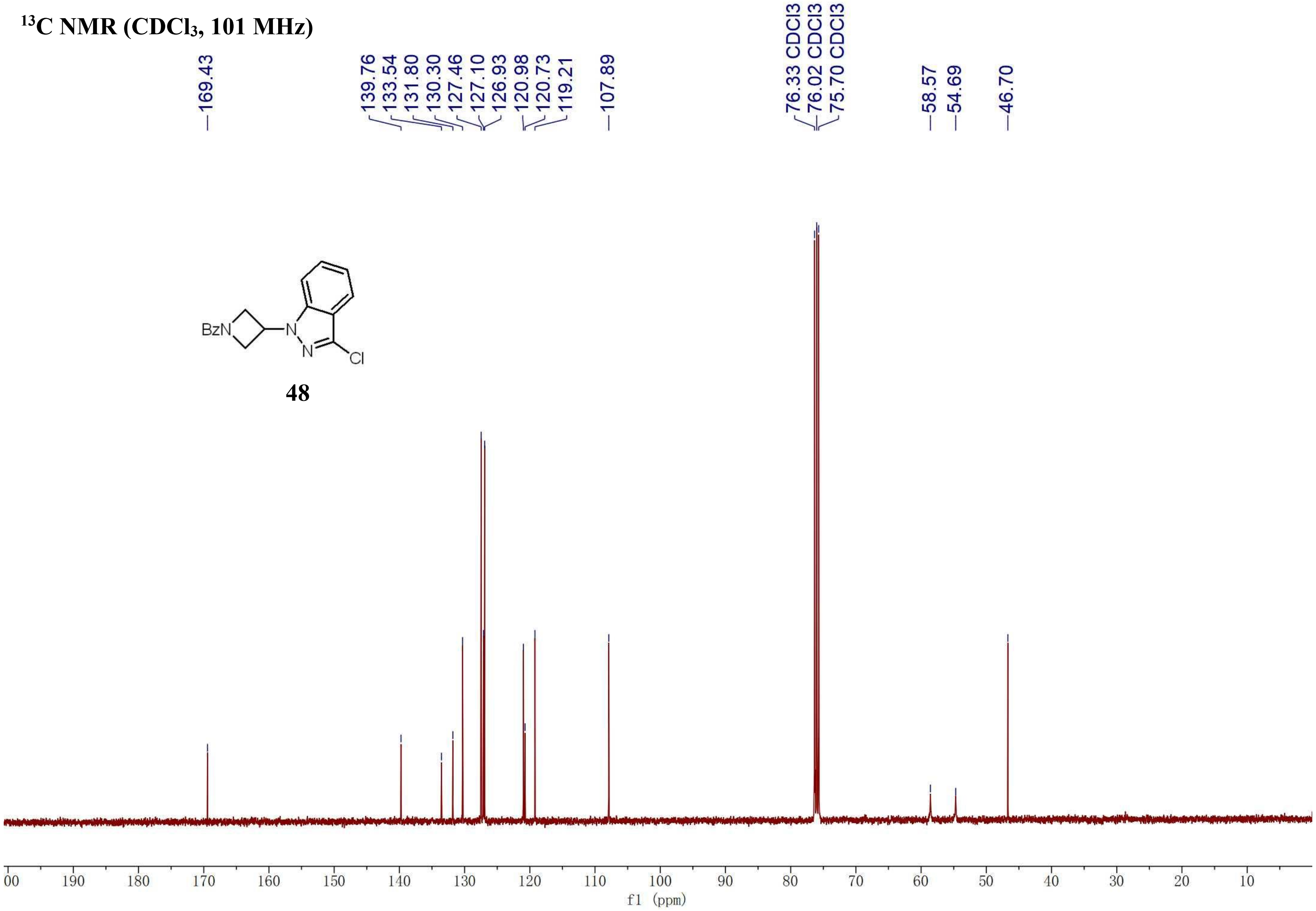

^{1}H NMR ($CDCl_3$, 400 MHz)

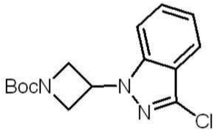

49

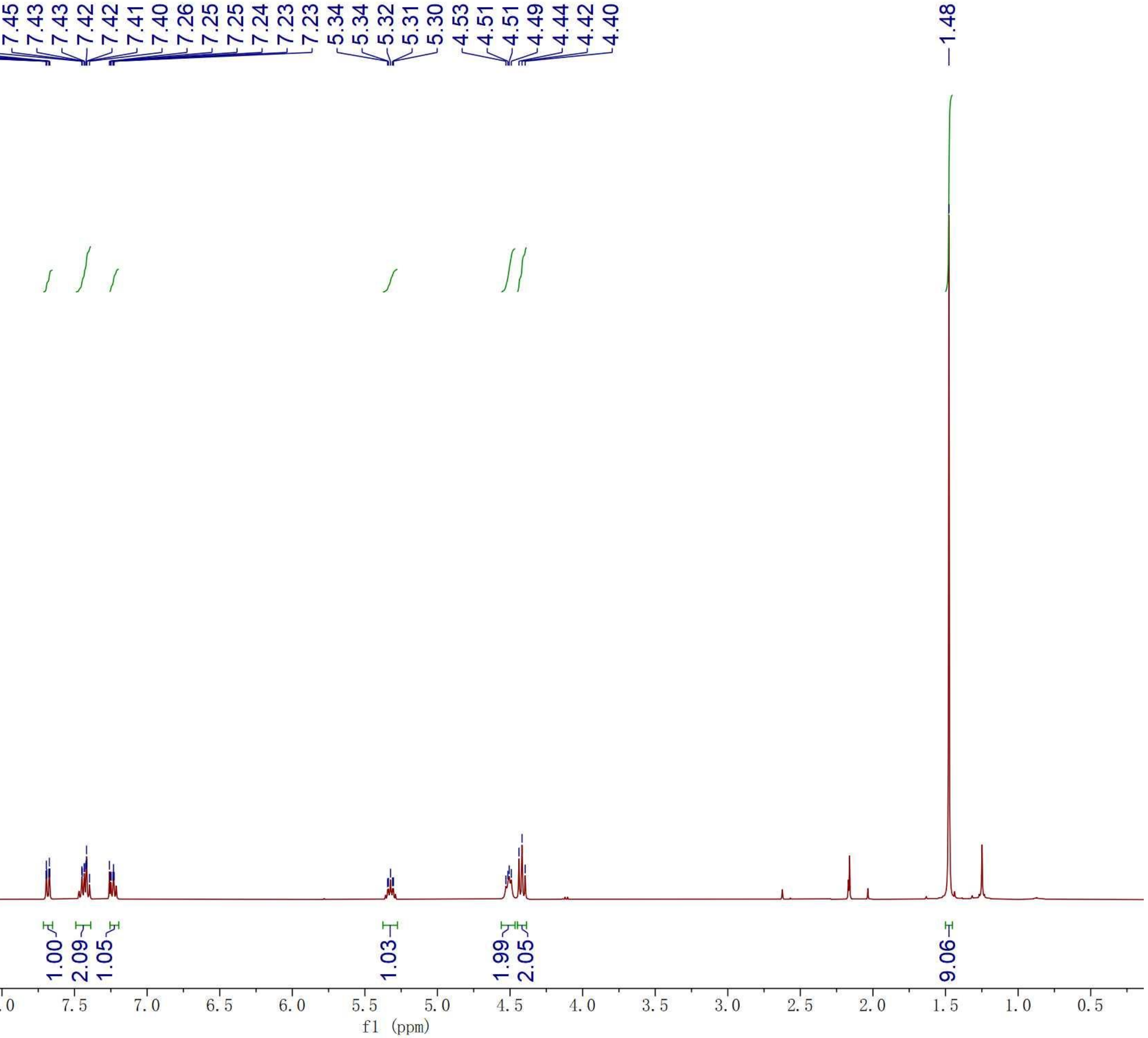

^{13}C NMR ($CDCl_3$, 101 MHz)

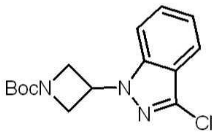

49

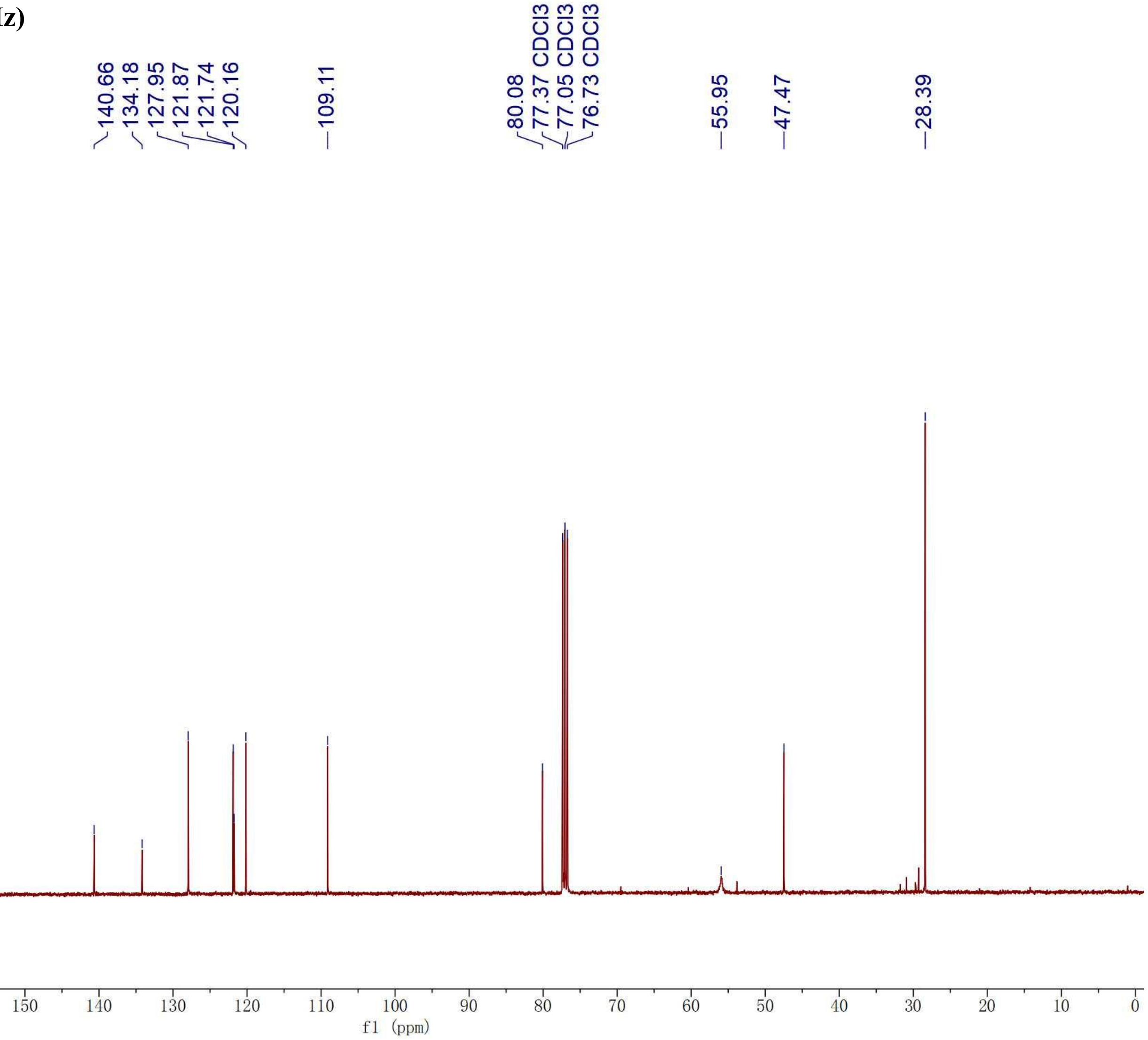

^{1}H NMR ($CDCl_3$, 400 MHz)

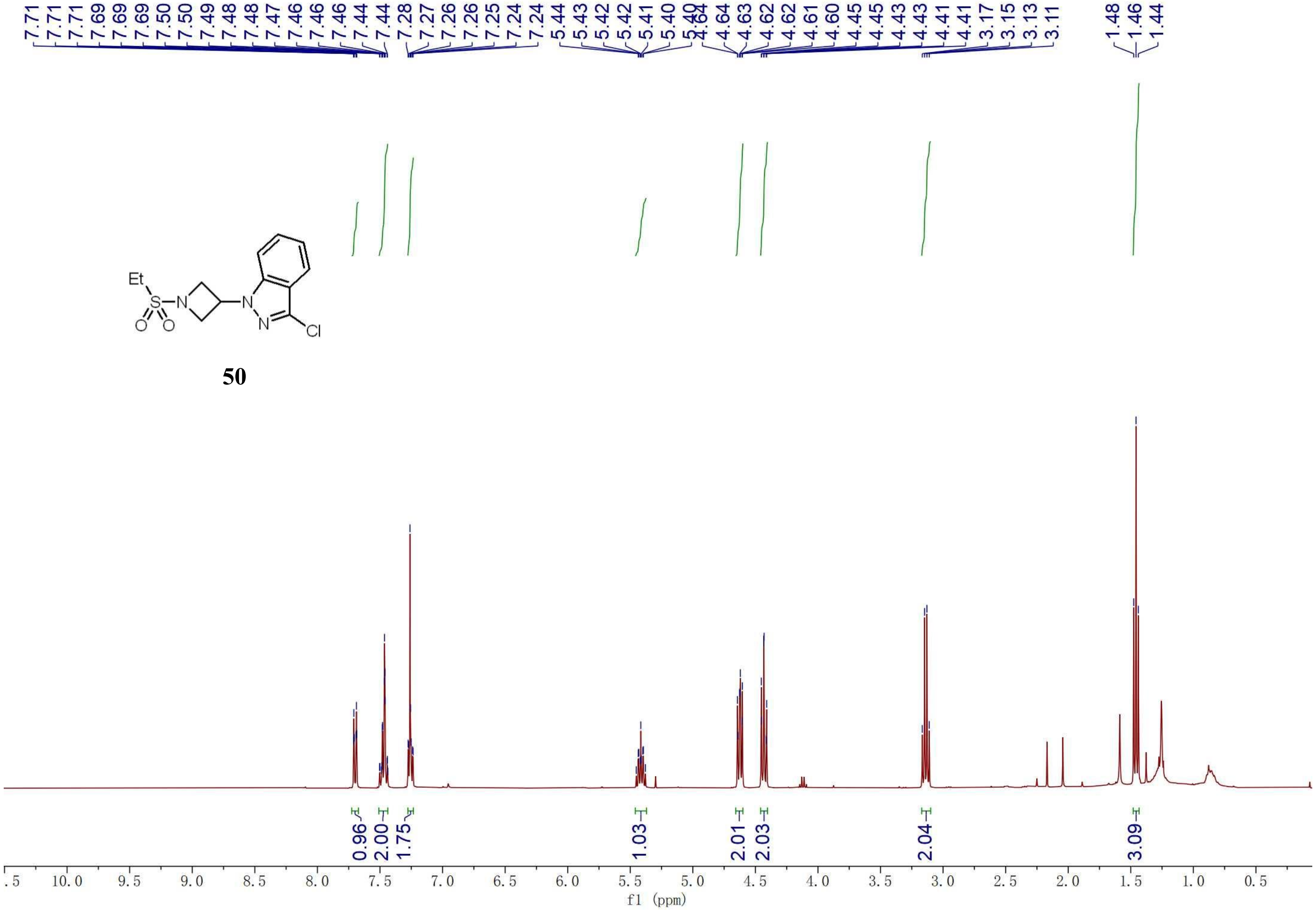

^{13}C NMR (CDCl$_3$, 101 MHz)

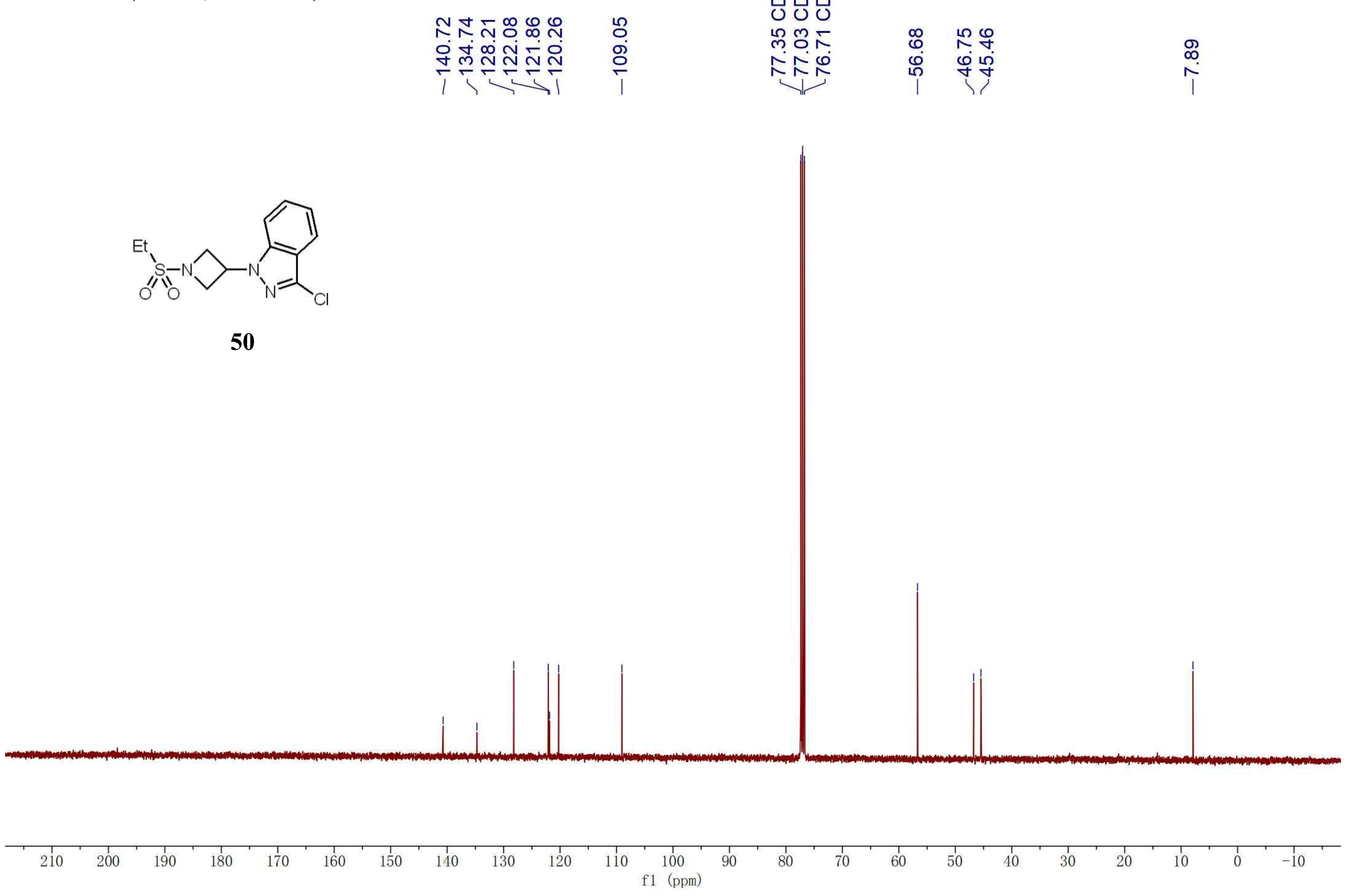

^{1}H NMR ($CDCl_3$, 400 MHz)

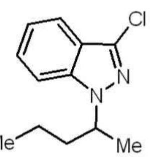

51

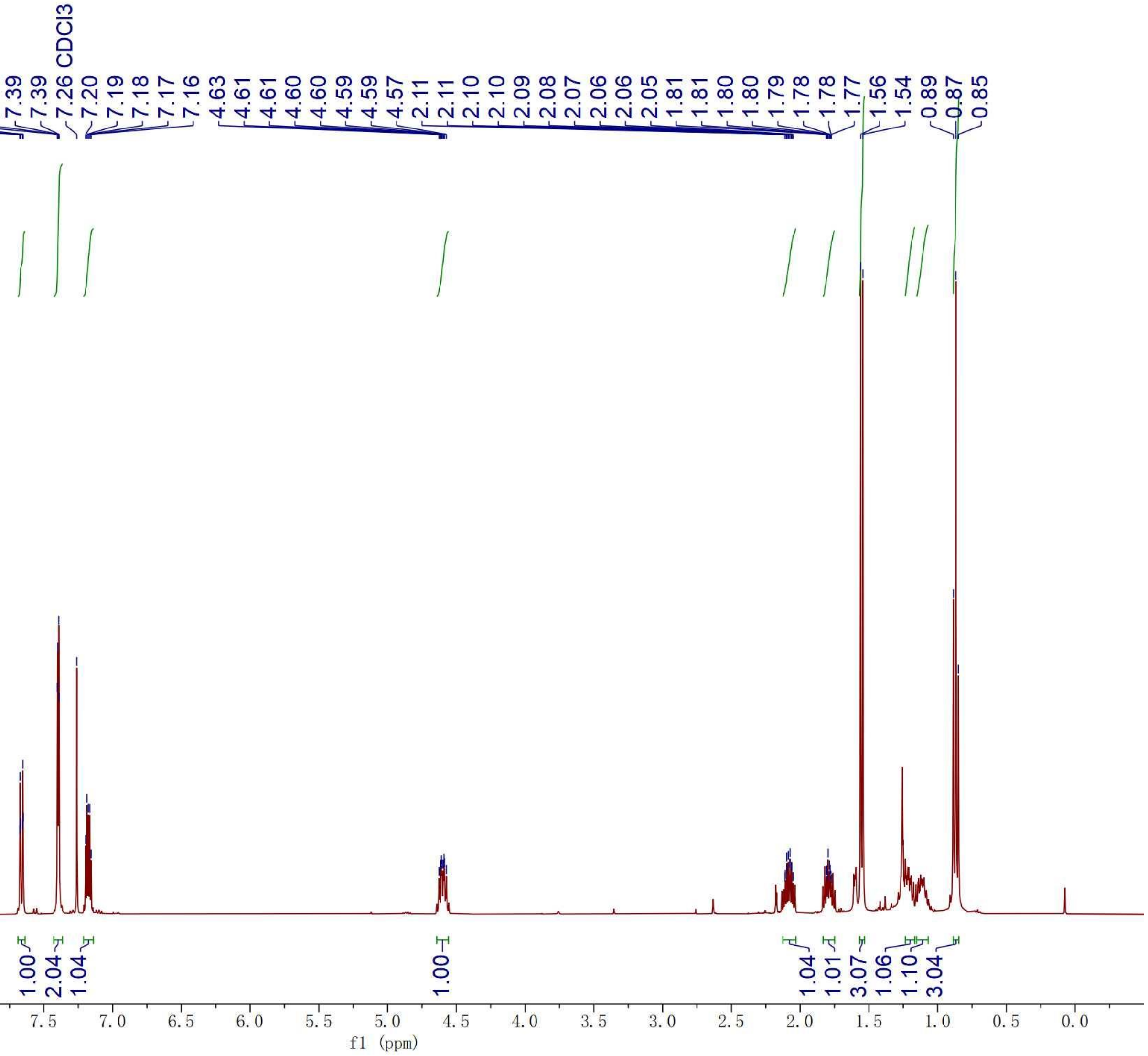

^{13}C NMR ($CDCl_3$, 101 MHz)

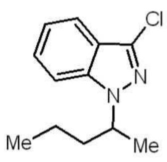

51

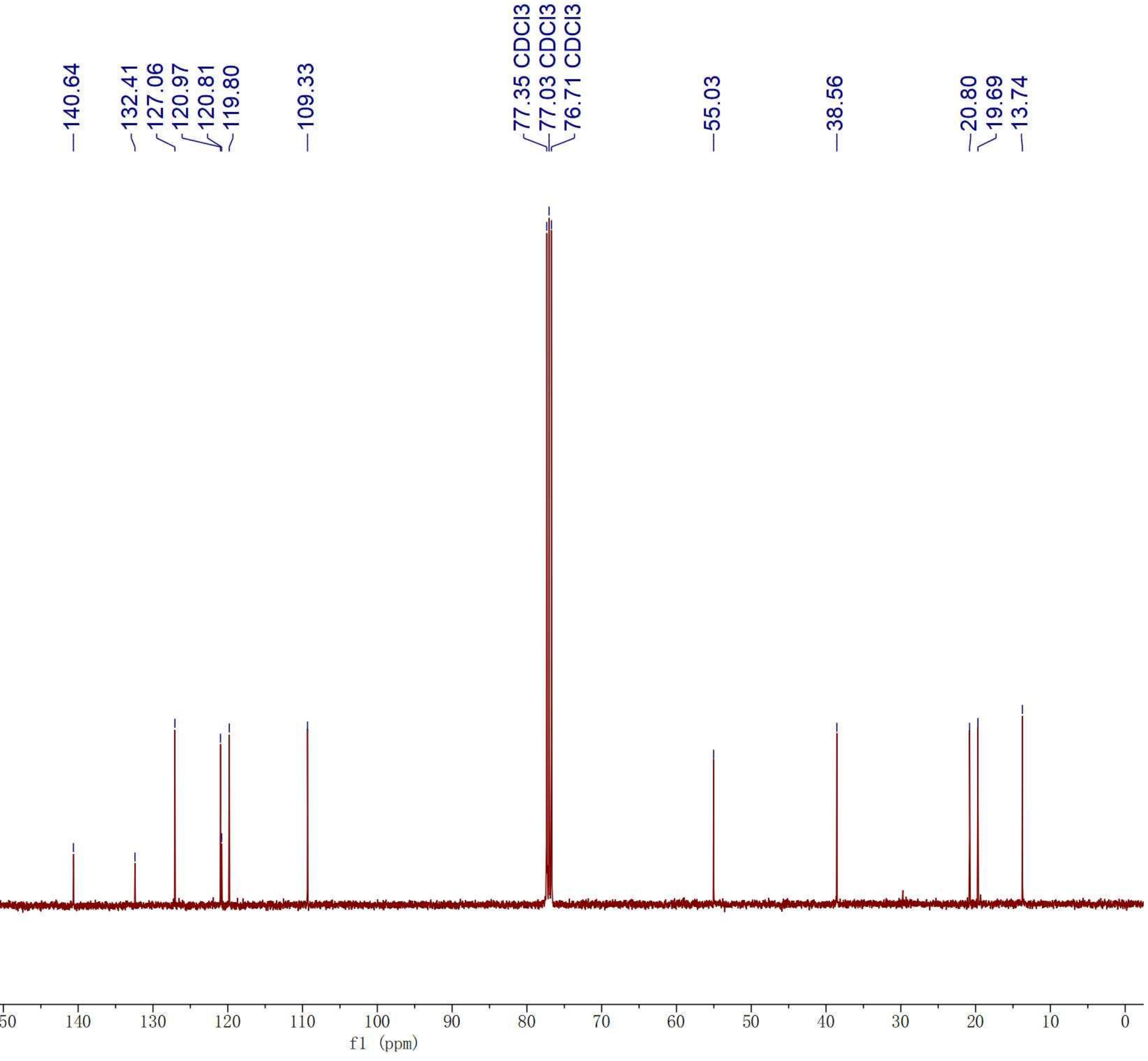

^{1}H NMR ($CDCl_3$, 400 MHz)

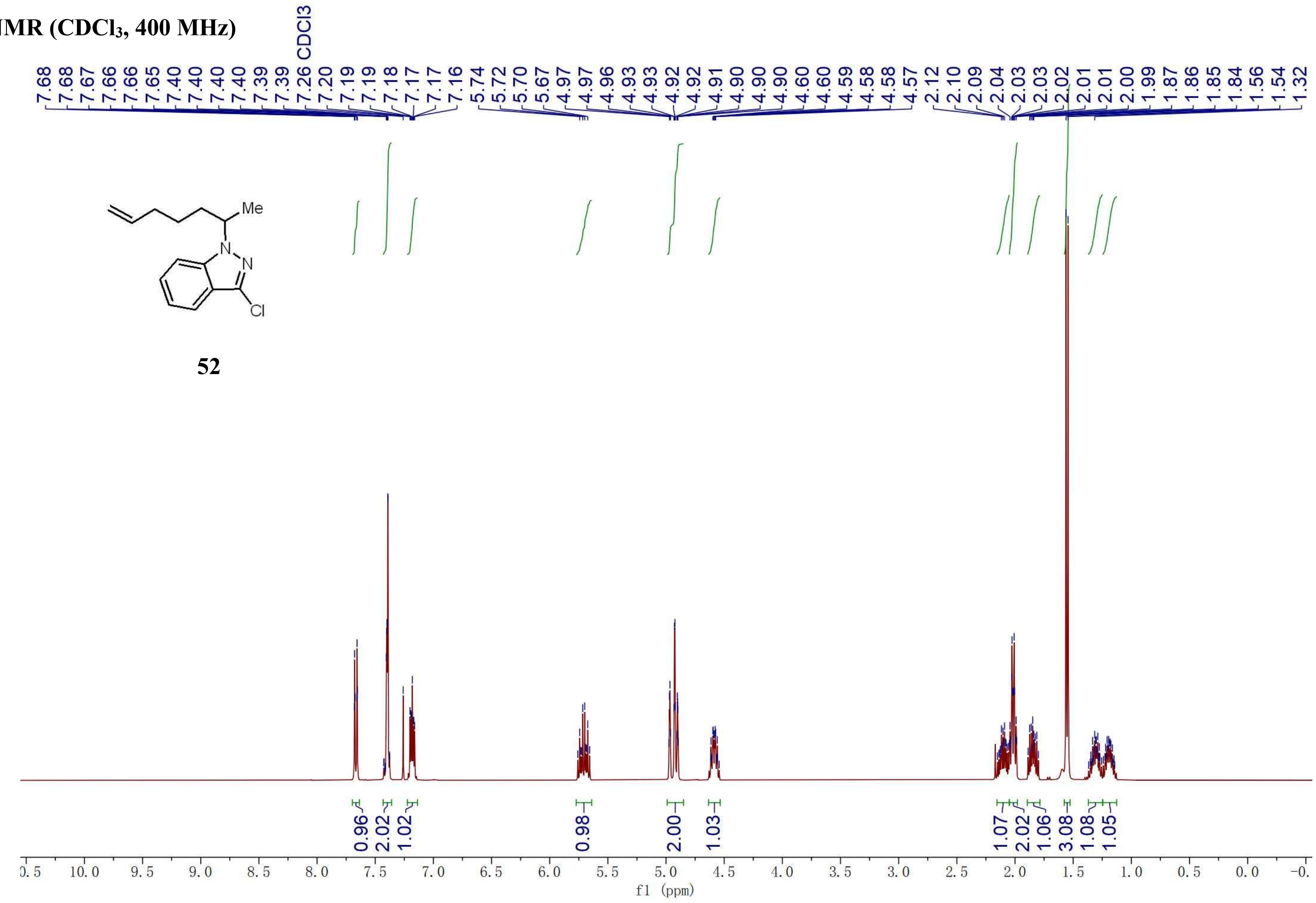

^{13}C NMR ($CDCl_3$, 101 MHz)

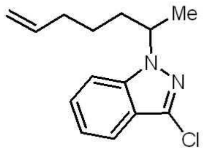

52

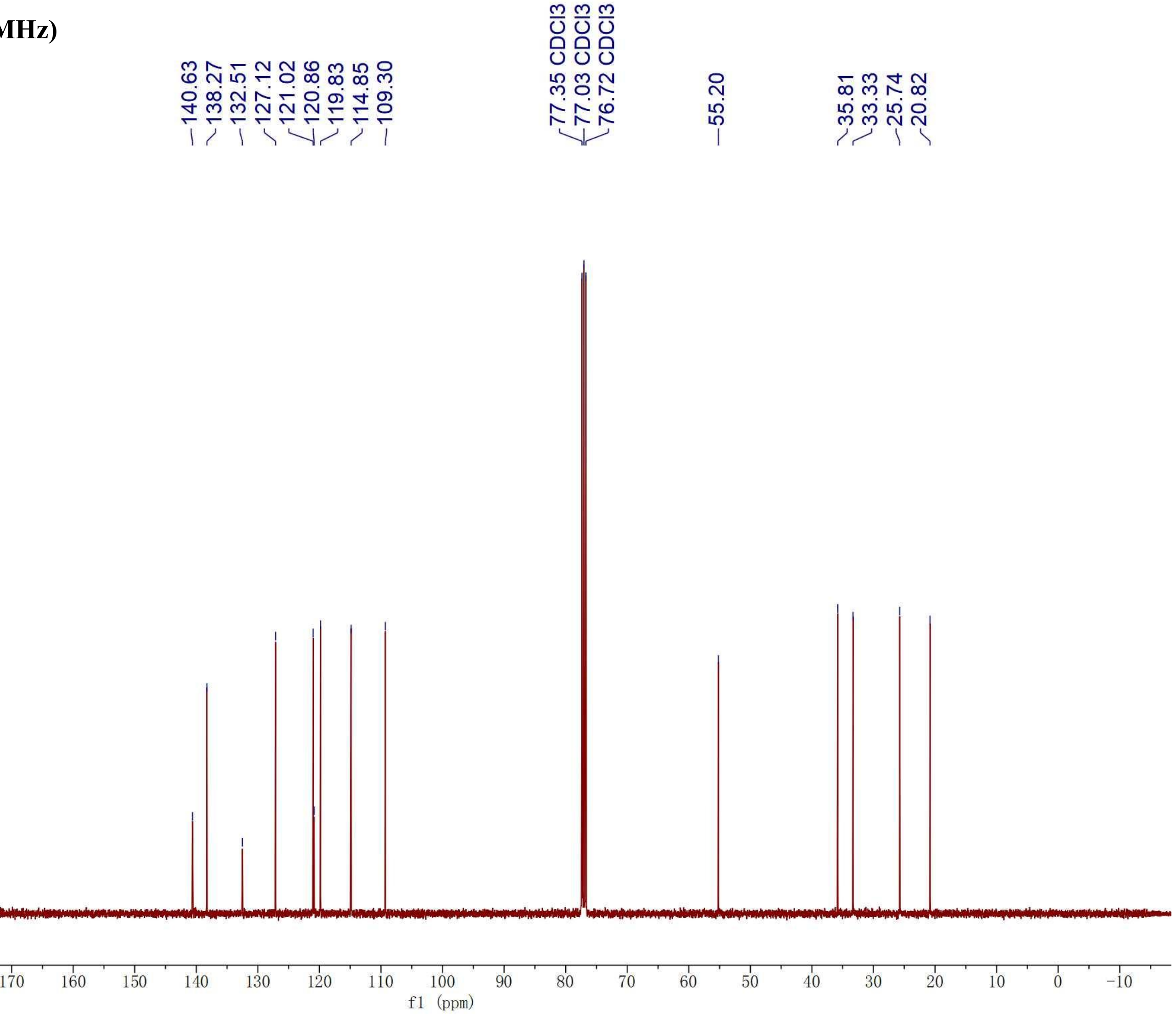

^{1}H NMR ($CDCl_3$, 400 MHz)

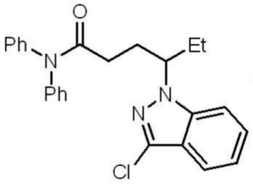

53

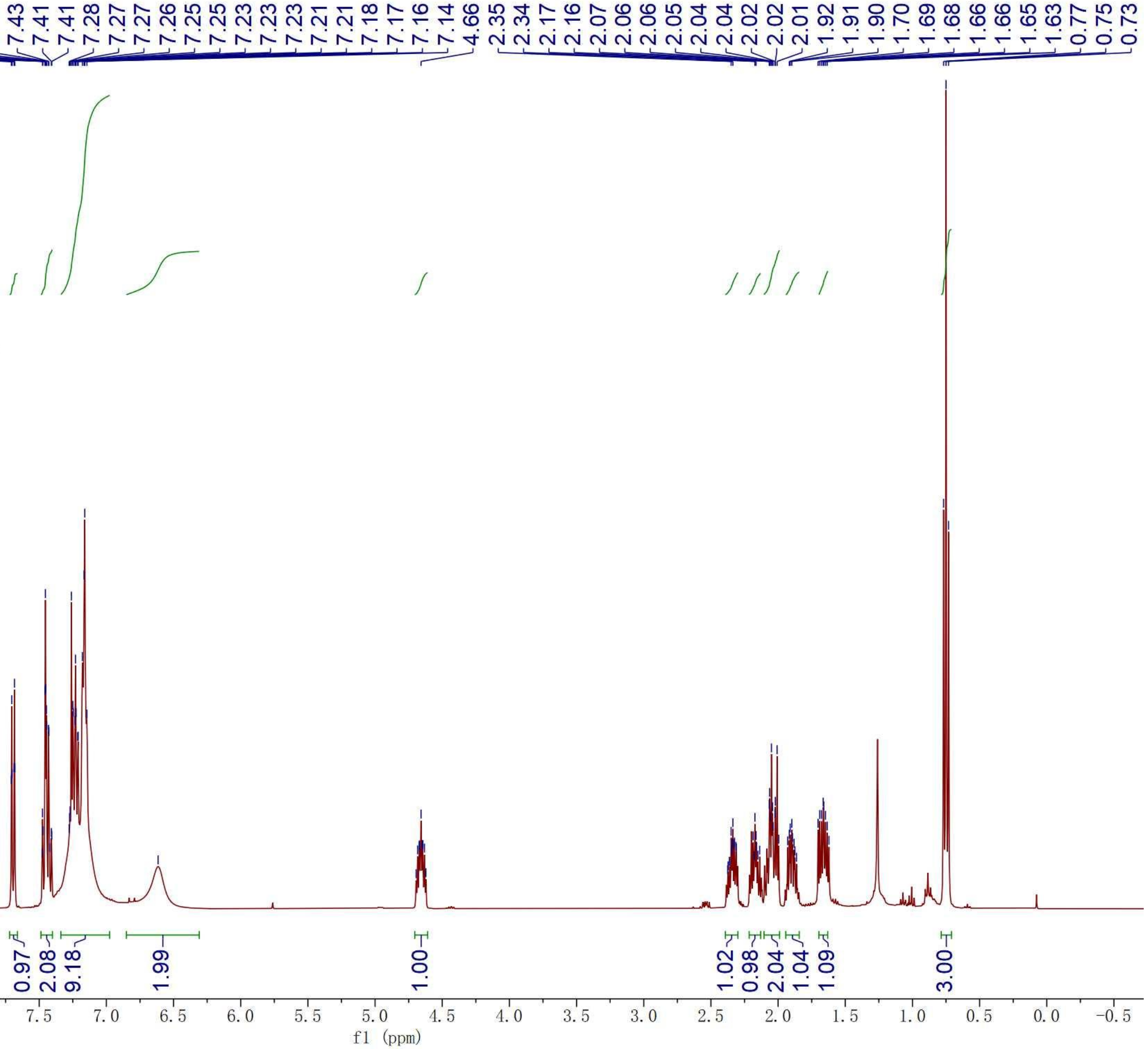

^{13}C NMR ($CDCl_3$, 101 MHz)

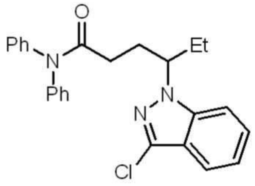

53

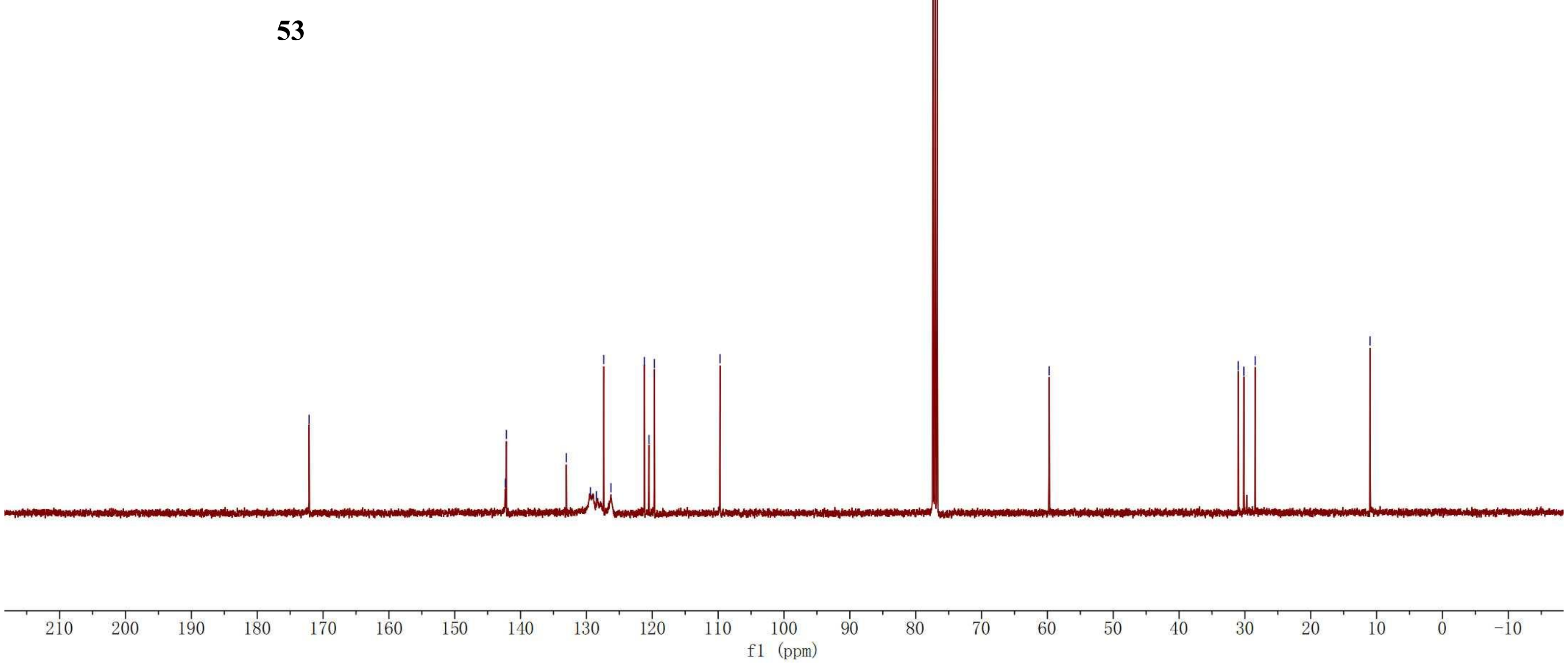

^{1}H NMR ($CDCl_3$, 400 MHz)

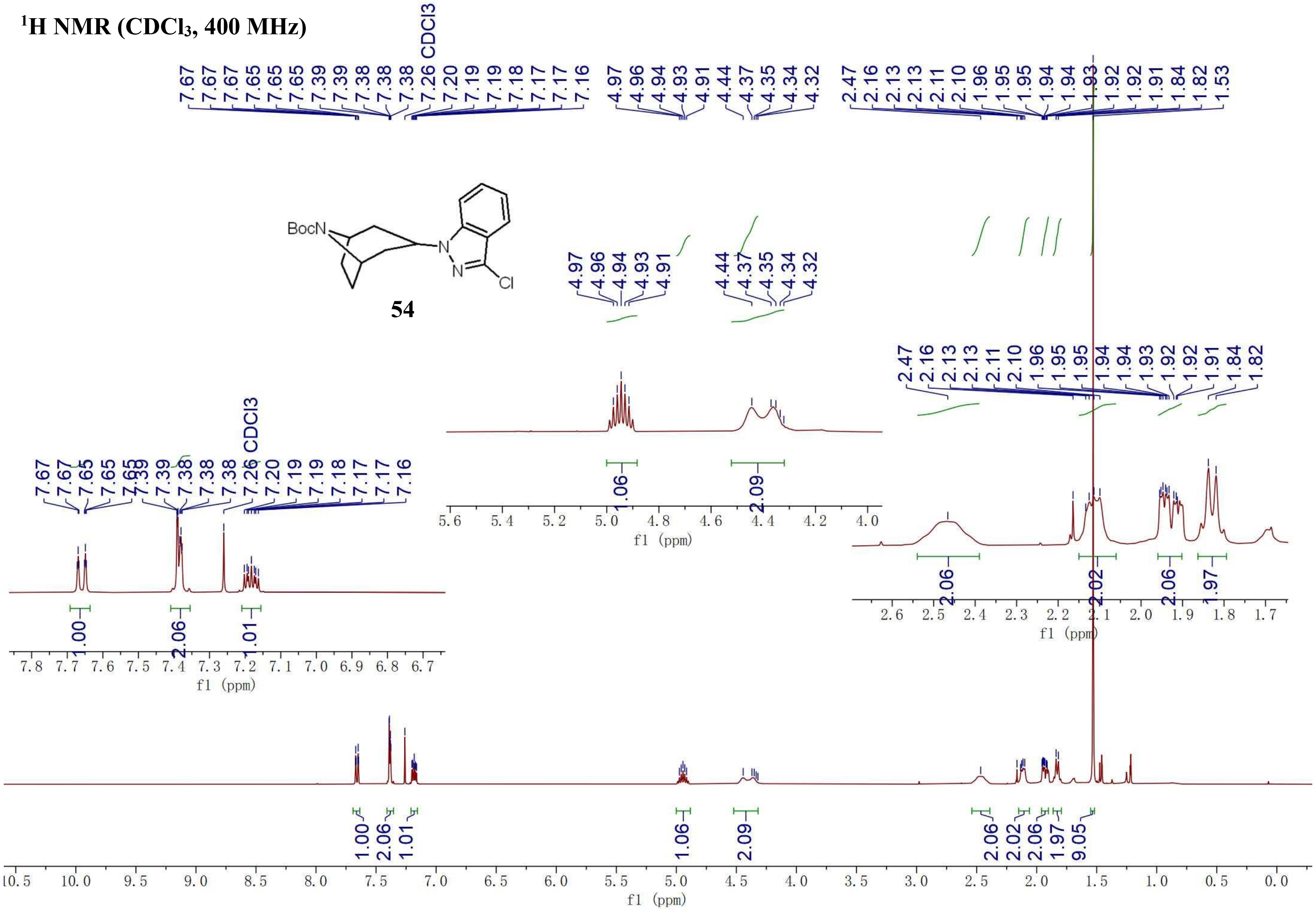

^{13}C NMR (CDCl3, 101 MHz)

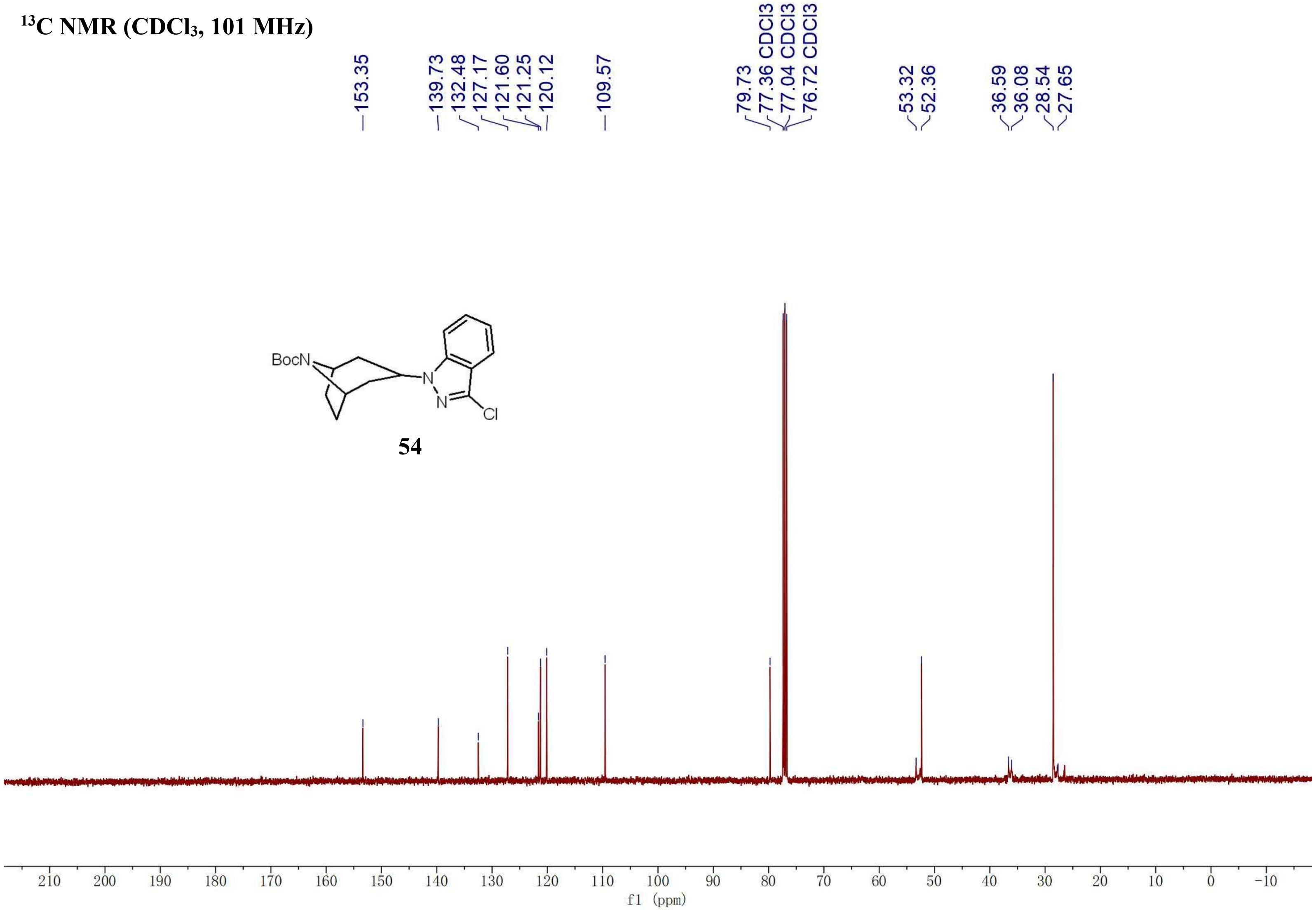

54

^{1}H NMR ($CDCl_3$, 400 MHz)

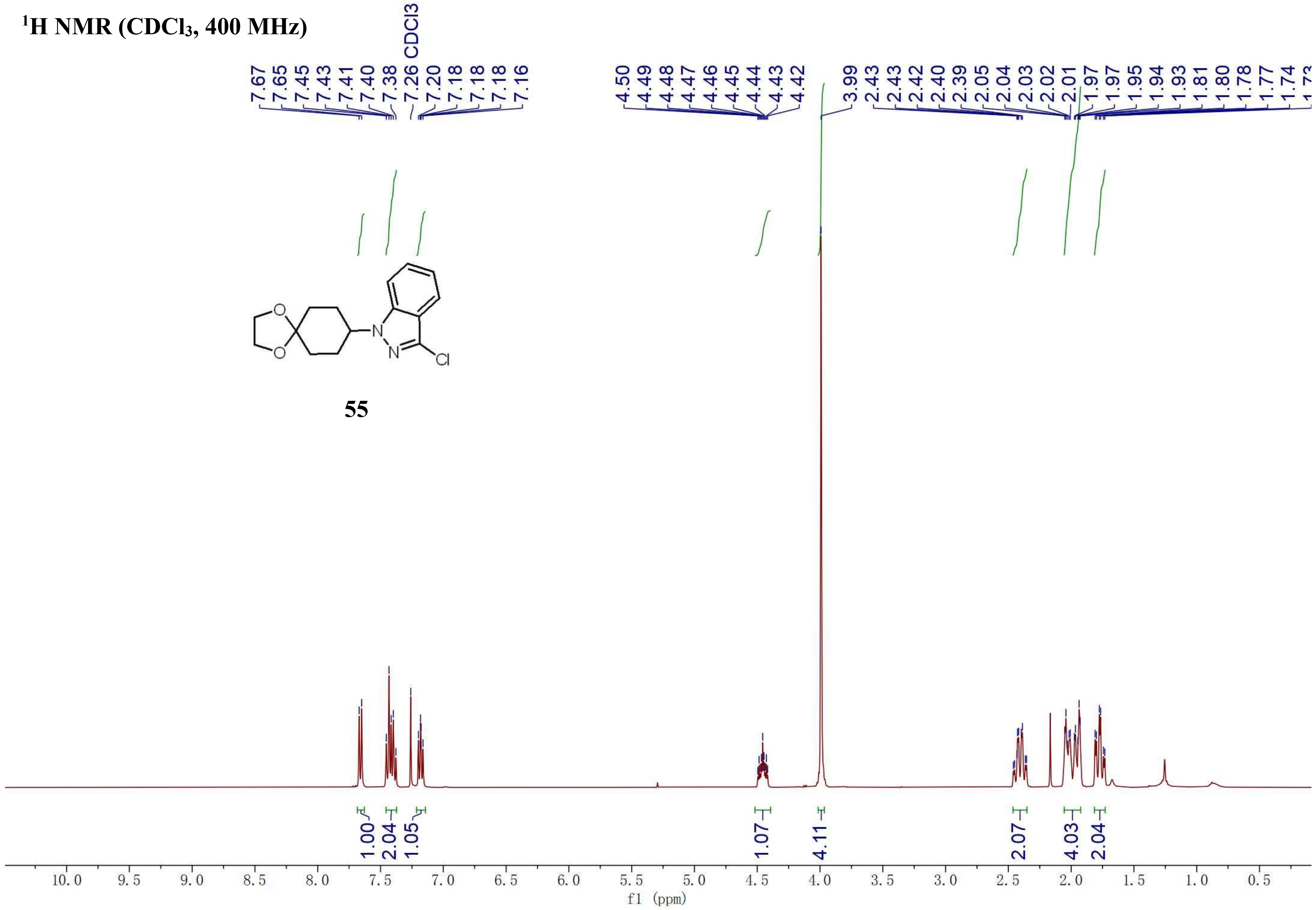

55

^{13}C NMR ($CDCl_3$, 101 MHz)

55

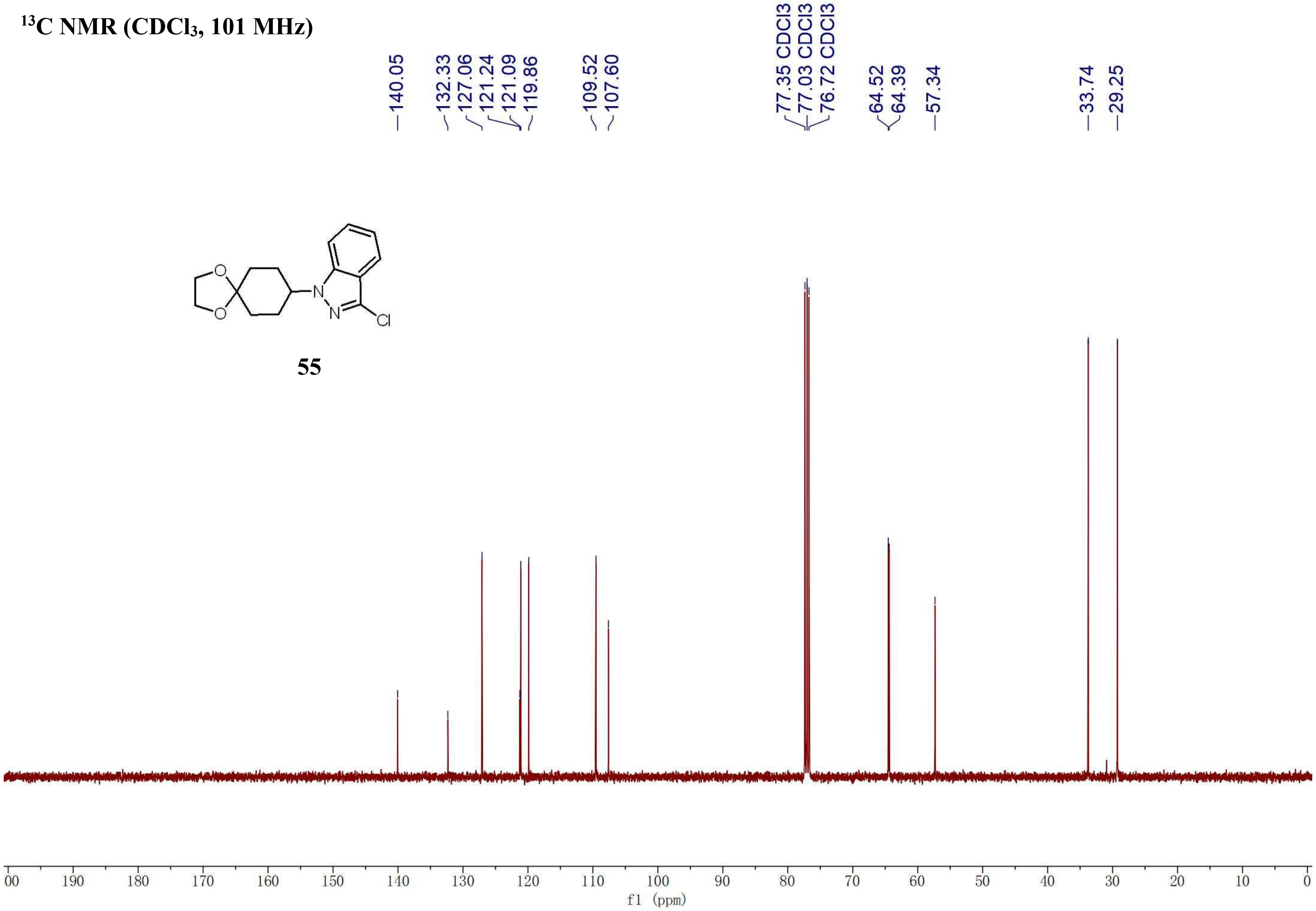

^{1}H NMR ($CDCl_3$, 400 MHz)

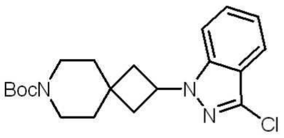

56

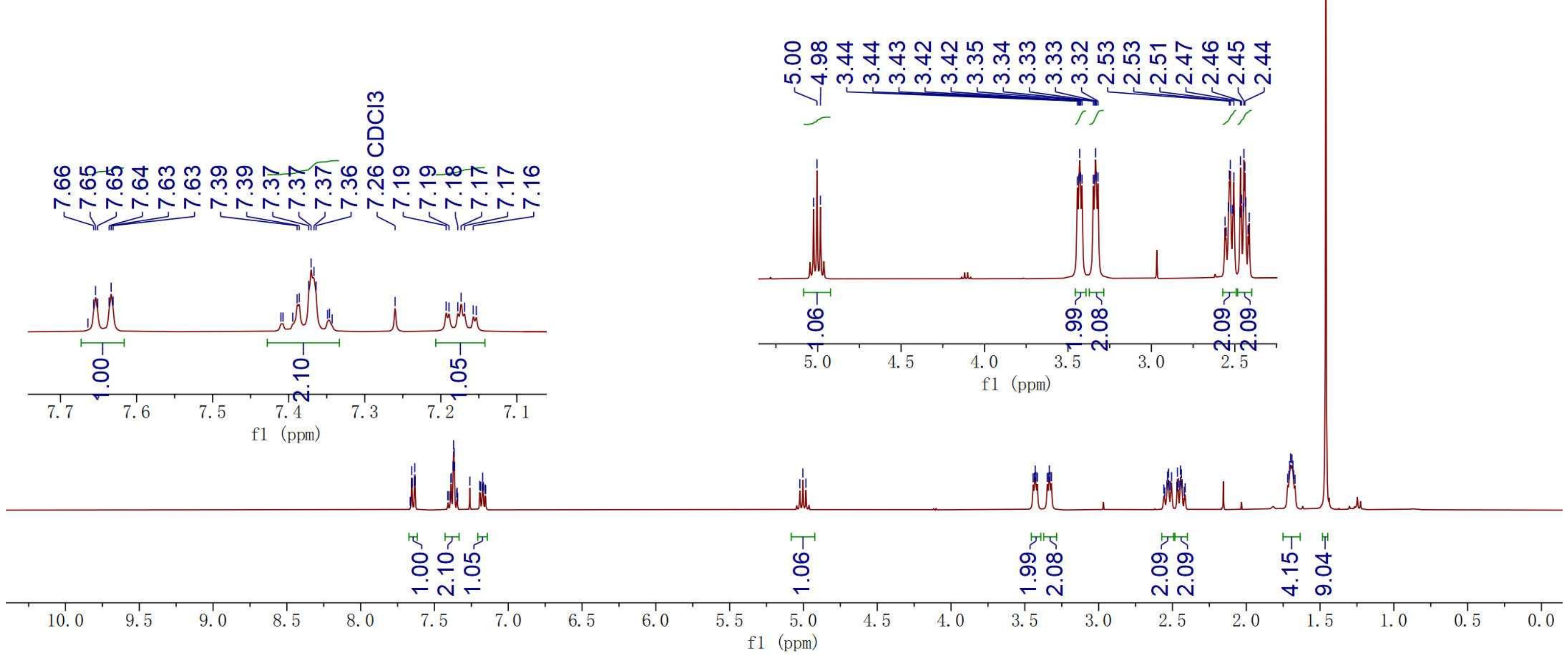

^{13}C NMR ($CDCl_3$, 101 MHz)

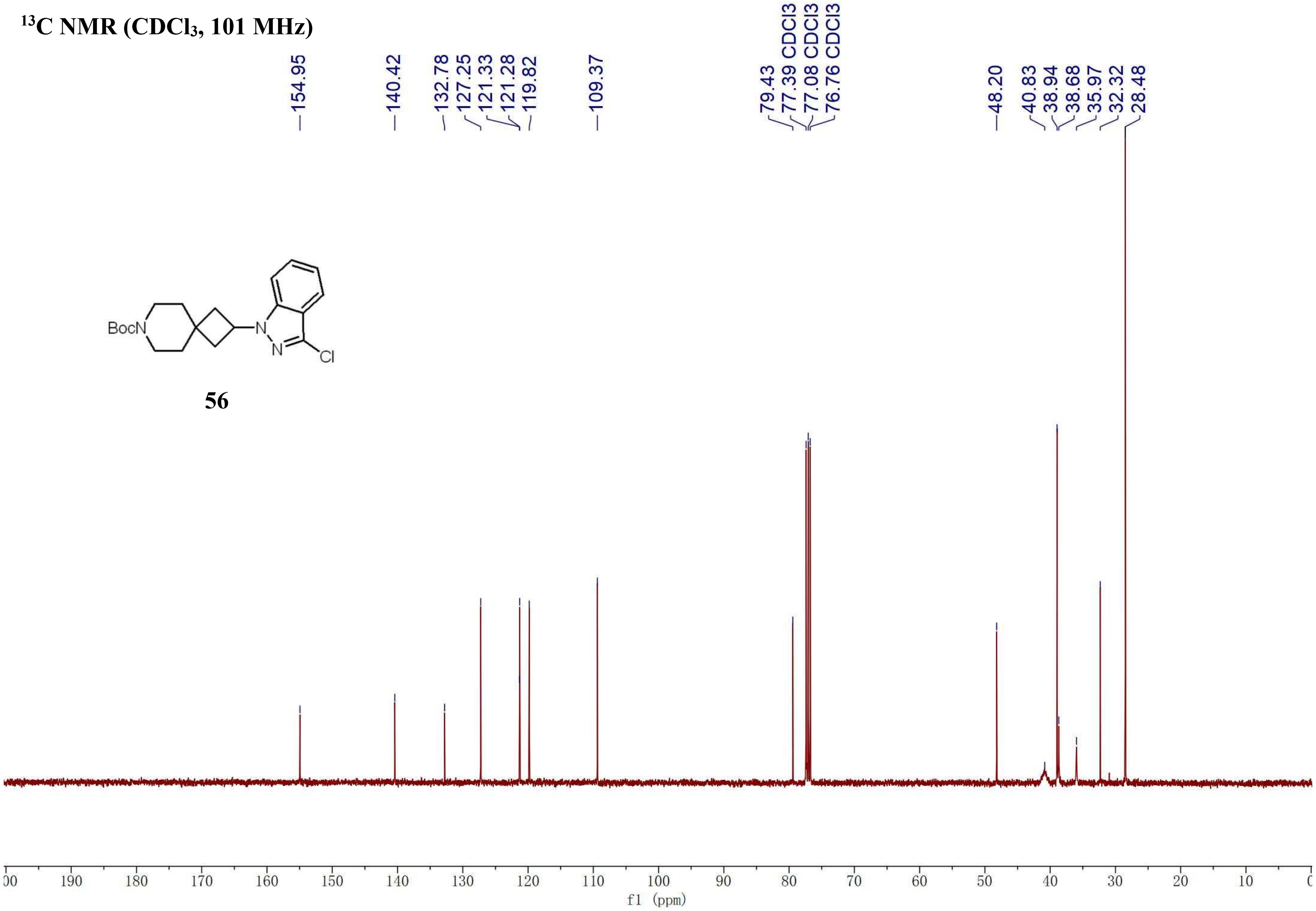

56

^{1}H NMR (CDCl$_3$, 400 MHz)

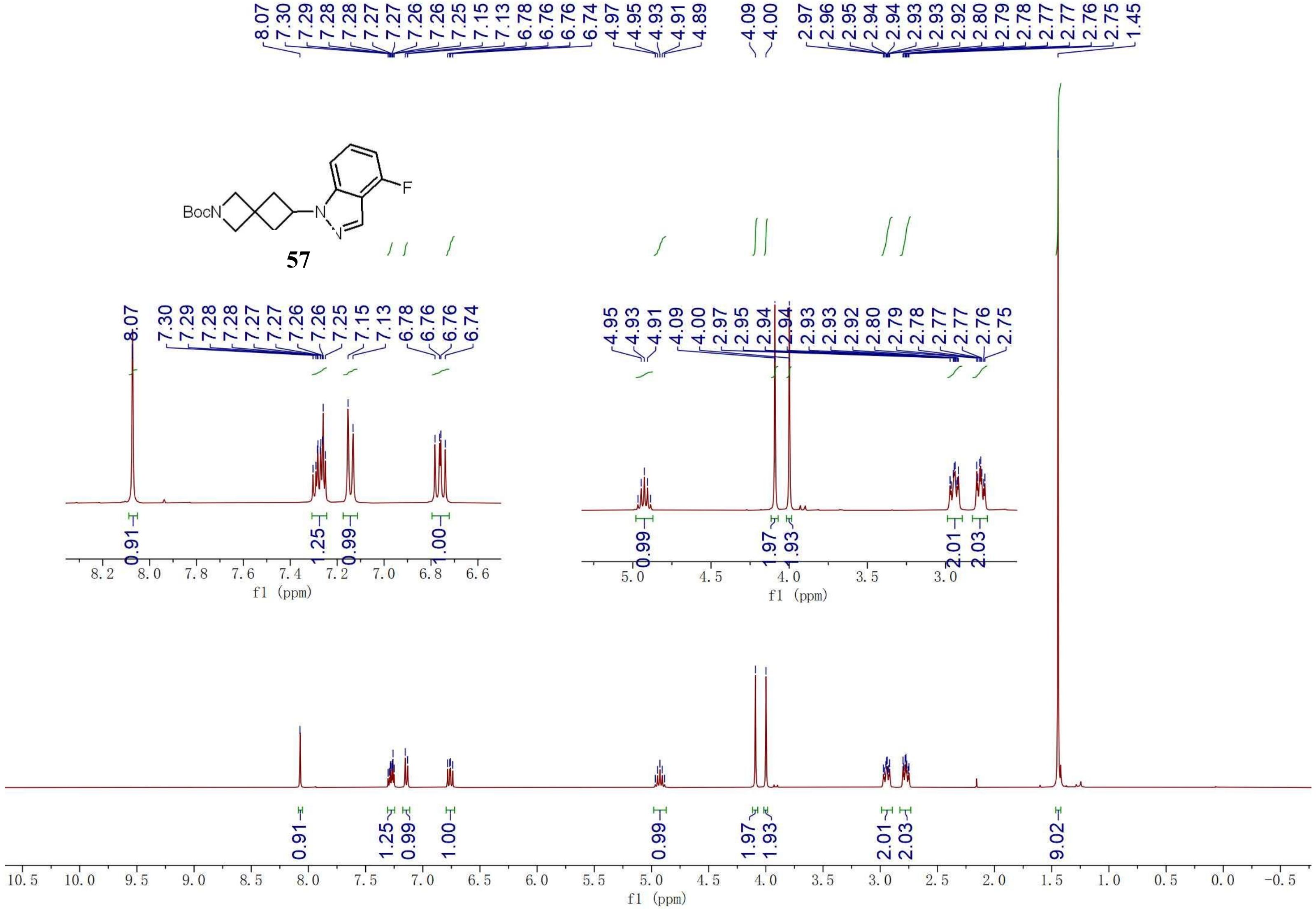

^{19}F NMR ($CDCl_3$, 376 MHz)

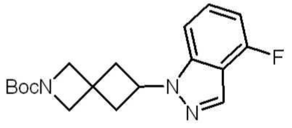

57

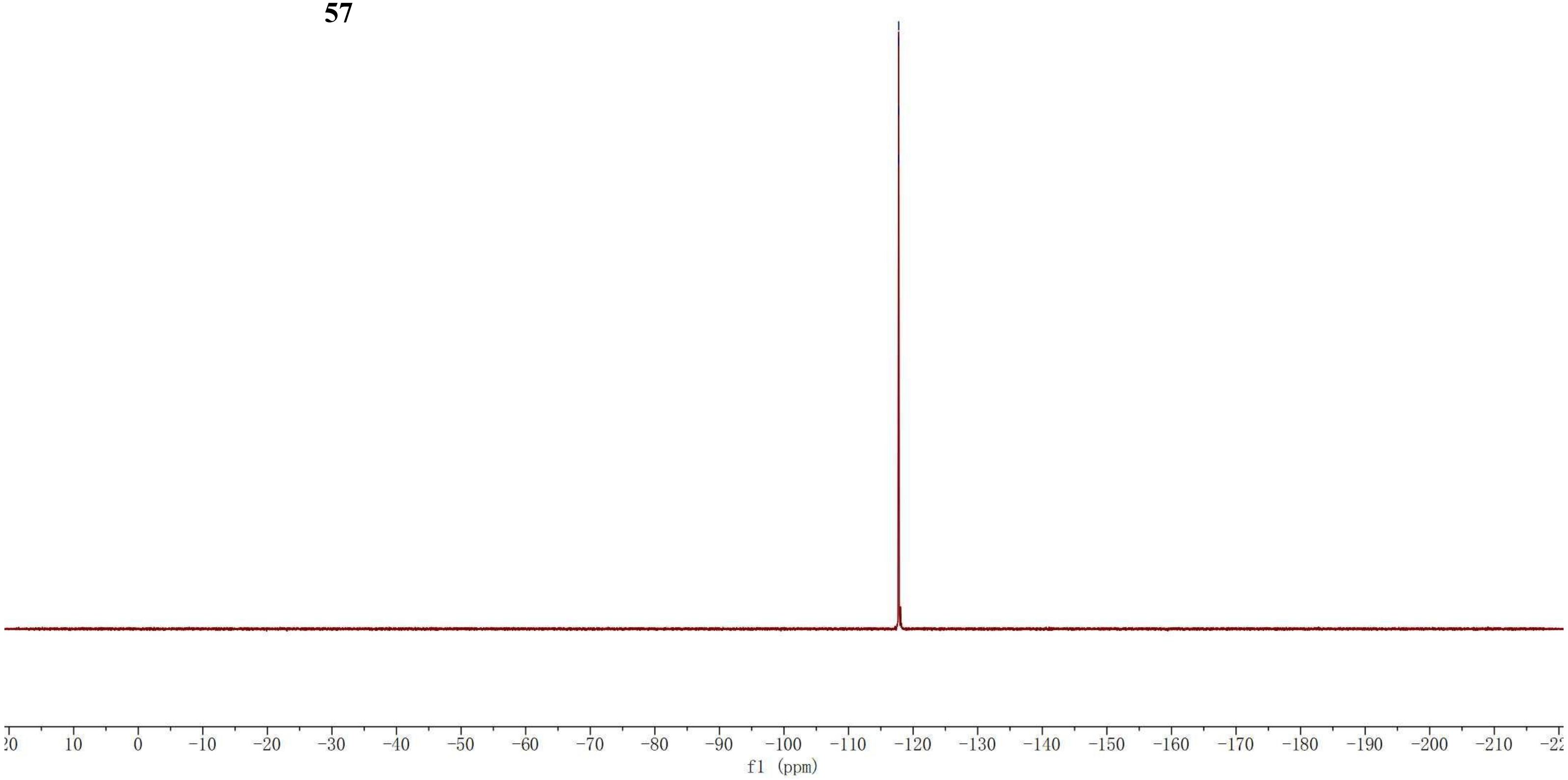

^{13}C NMR (CDCl₃, 101 MHz)

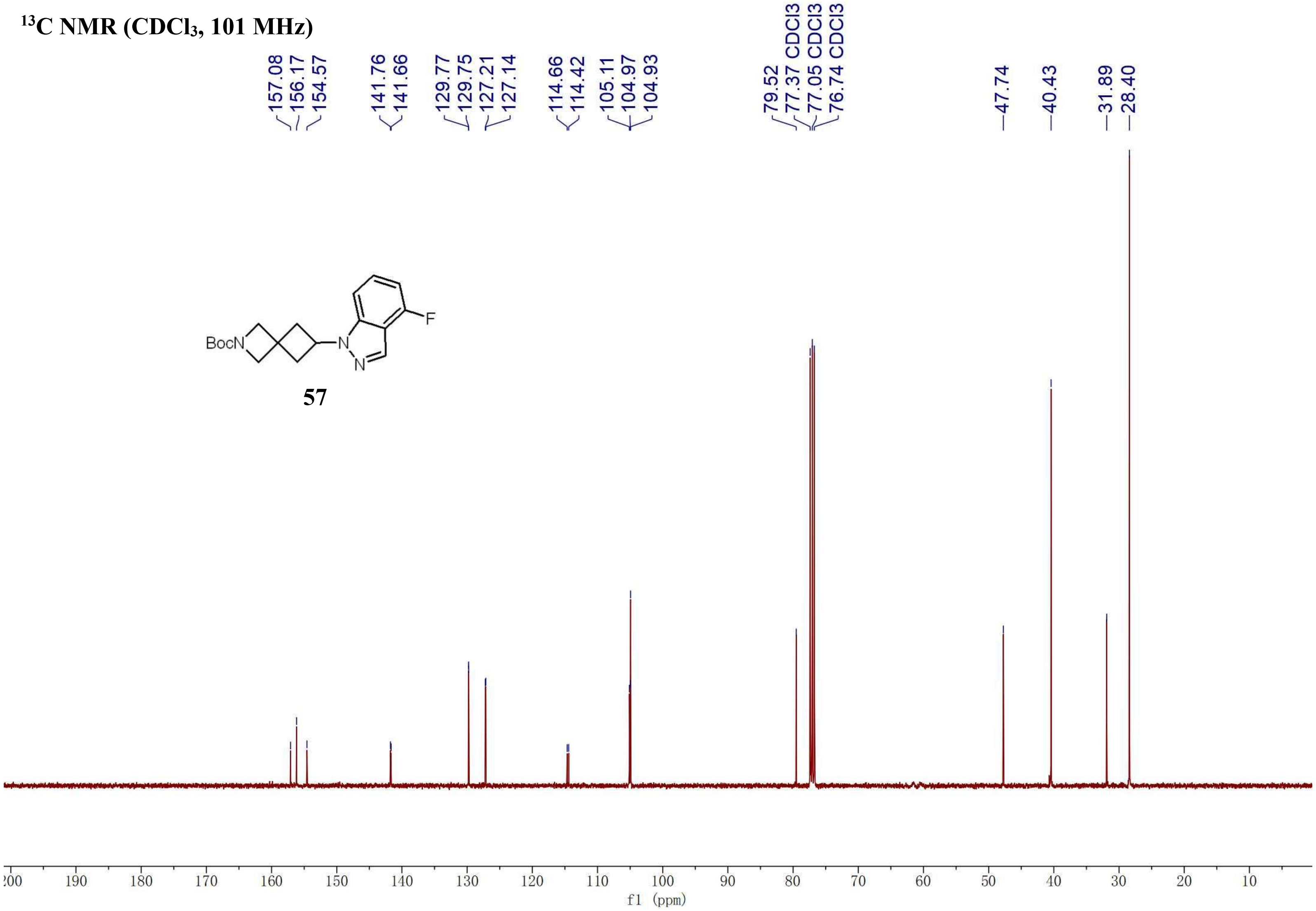

57

^{1}H NMR ($CDCl_3$, 400 MHz)

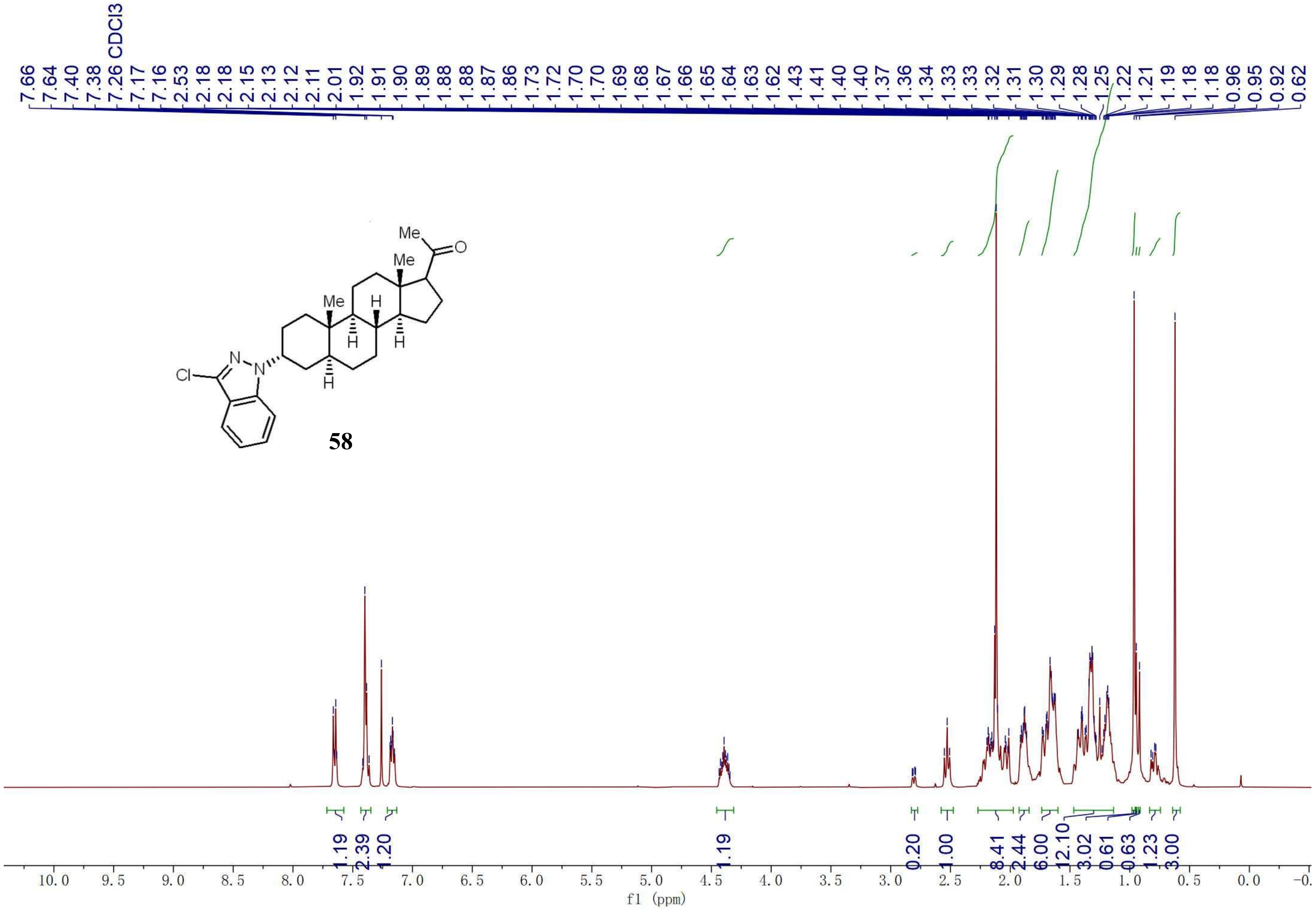

58

^{13}C NMR ($CDCl_3$, 101 MHz)

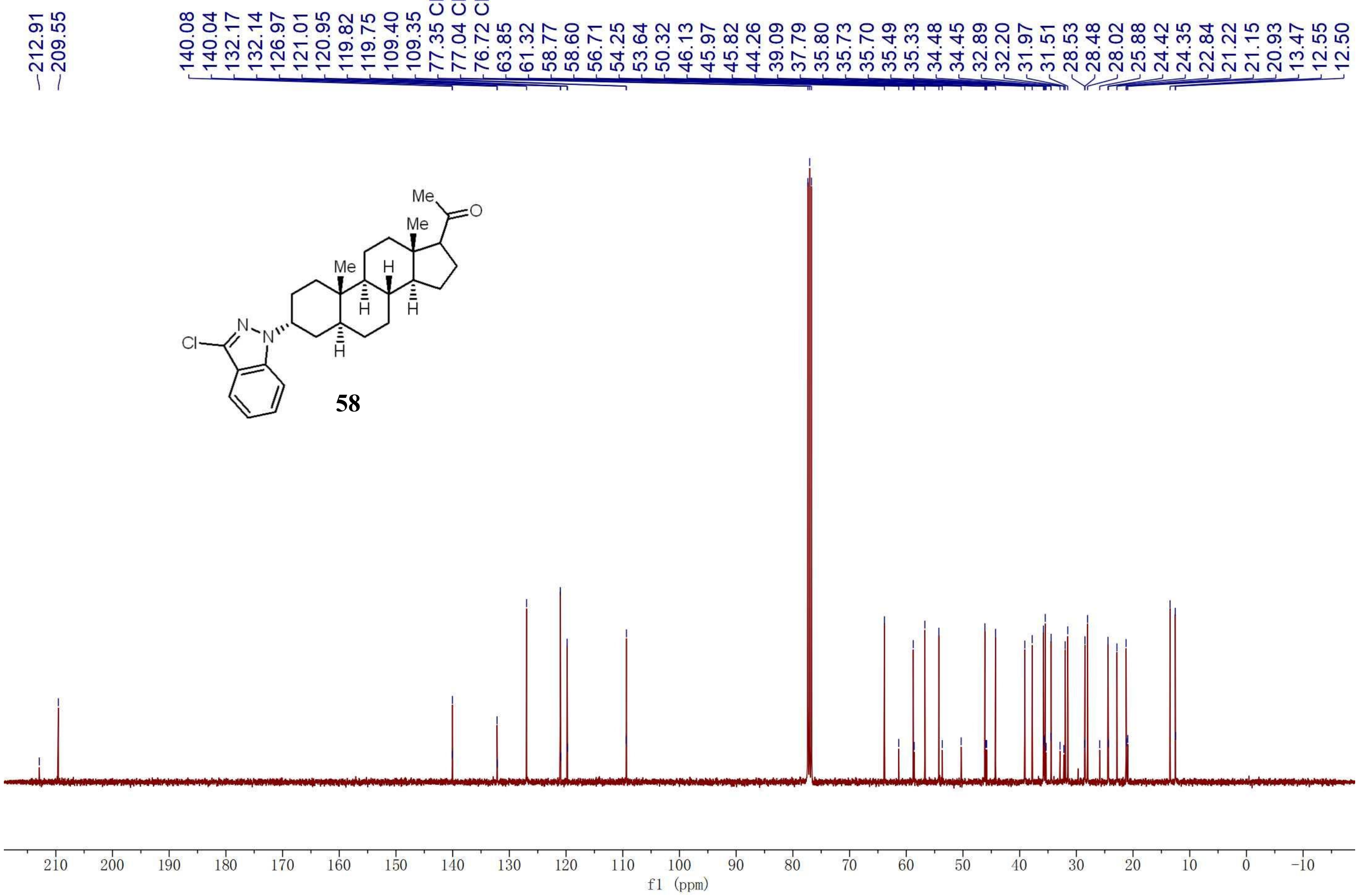

58

^{1}H NMR ($CDCl_3$, 400 MHz)

59

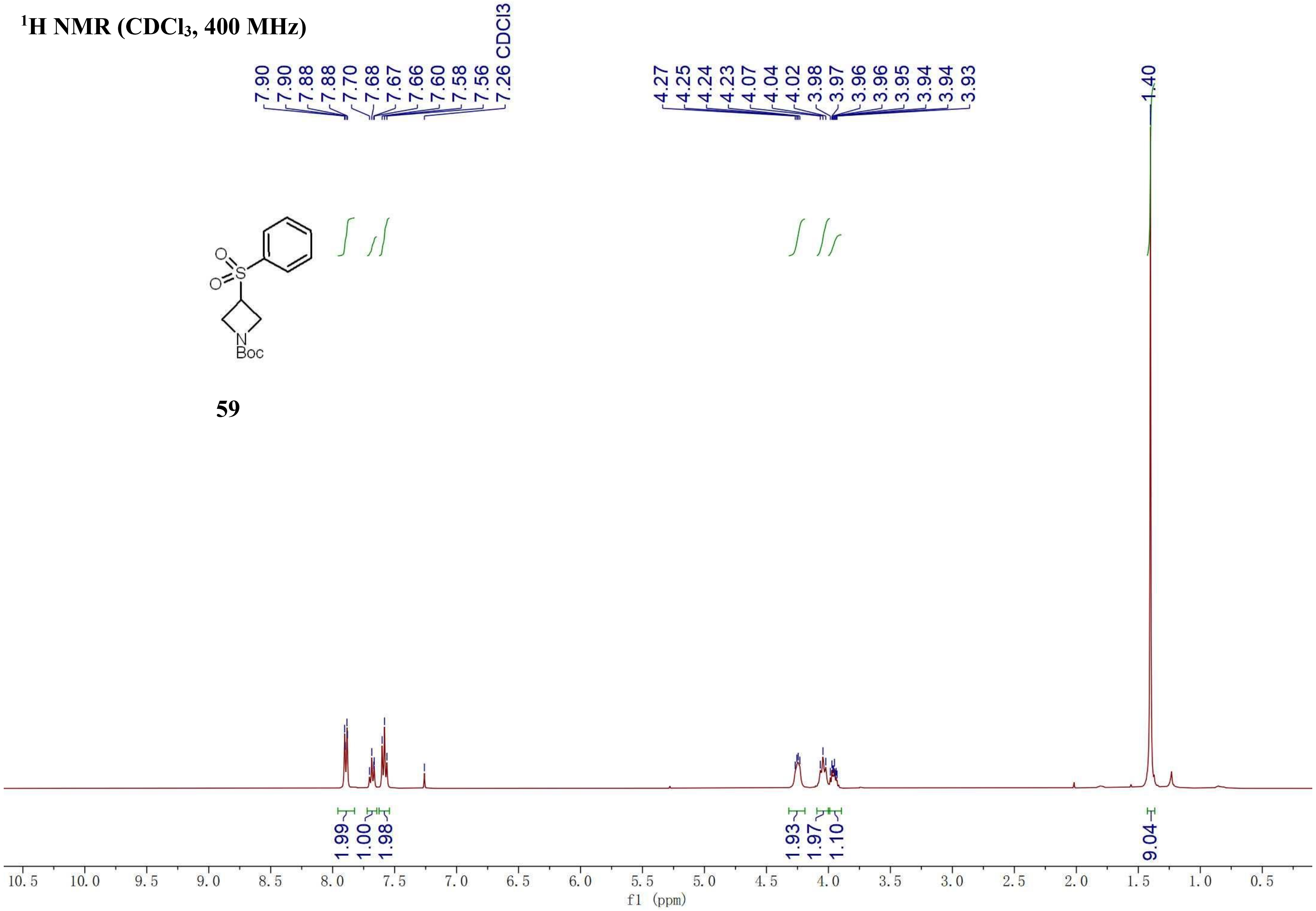

^{13}C NMR ($CDCl_3$, 101 MHz)

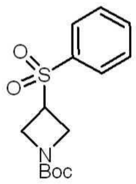

59

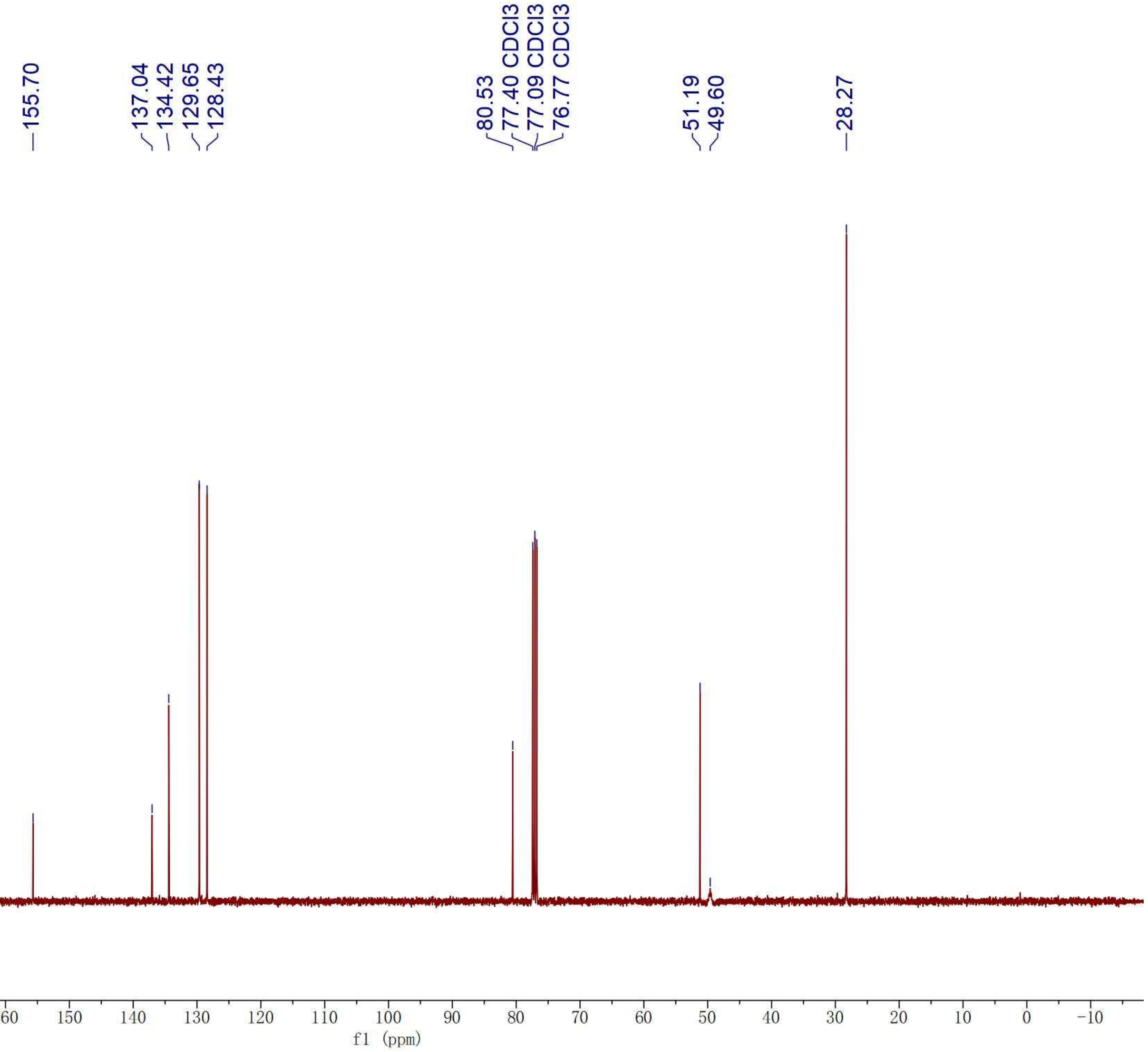

^{1}H NMR (CDCl$_3$, 400 MHz)

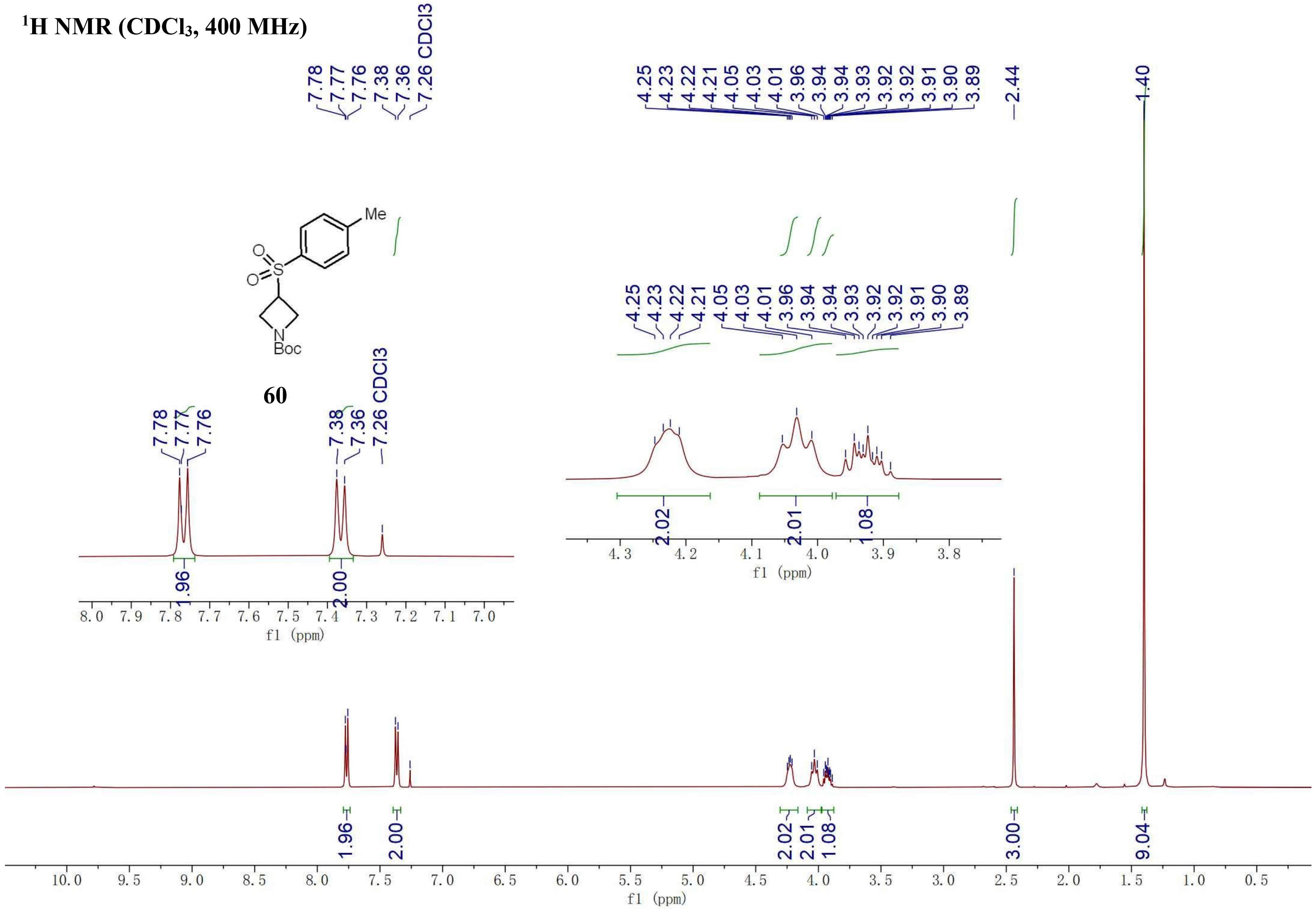

^{13}C NMR ($CDCl_3$, 101 MHz)

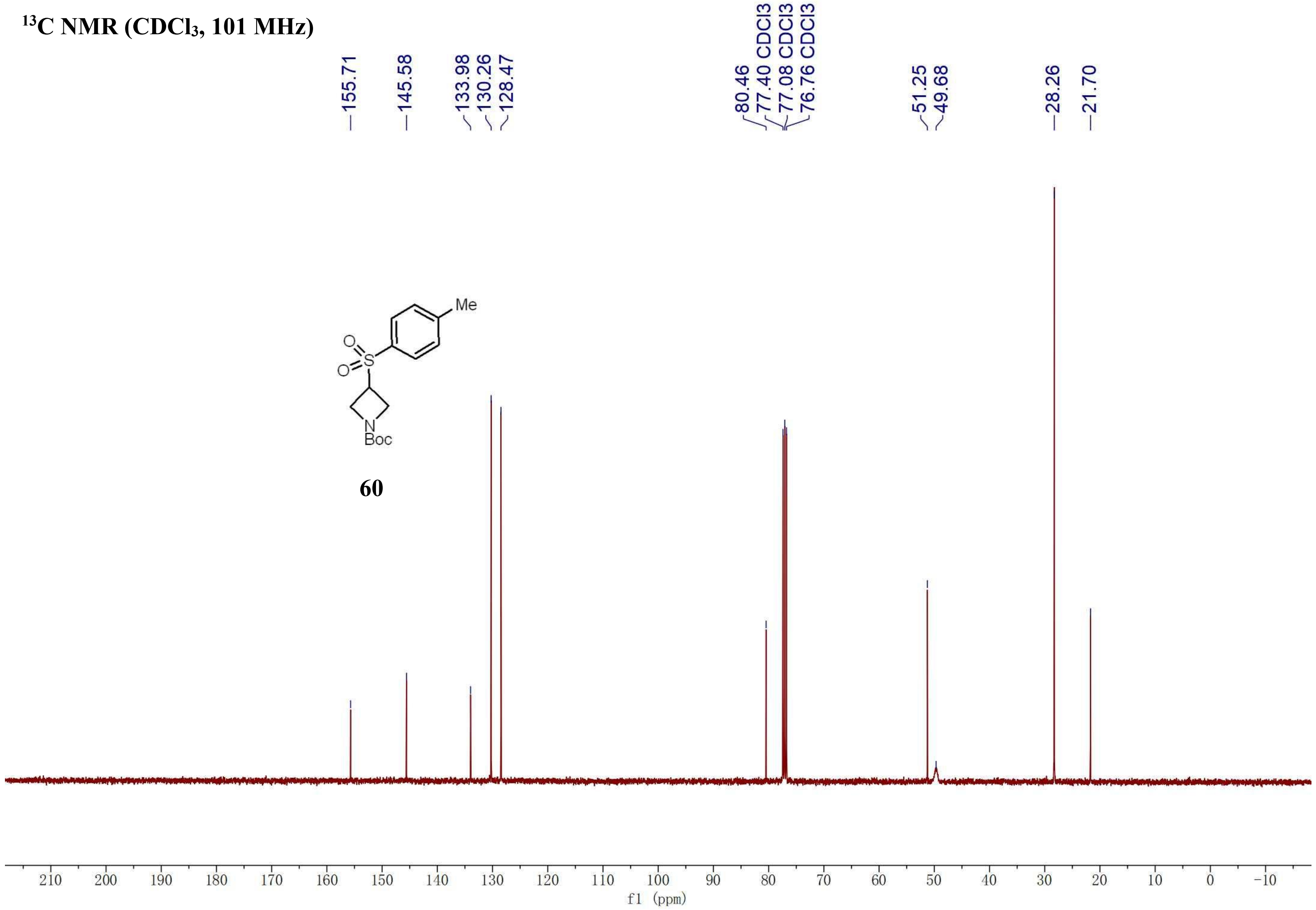

^{1}H NMR (CDCl$_3$, 400 MHz)

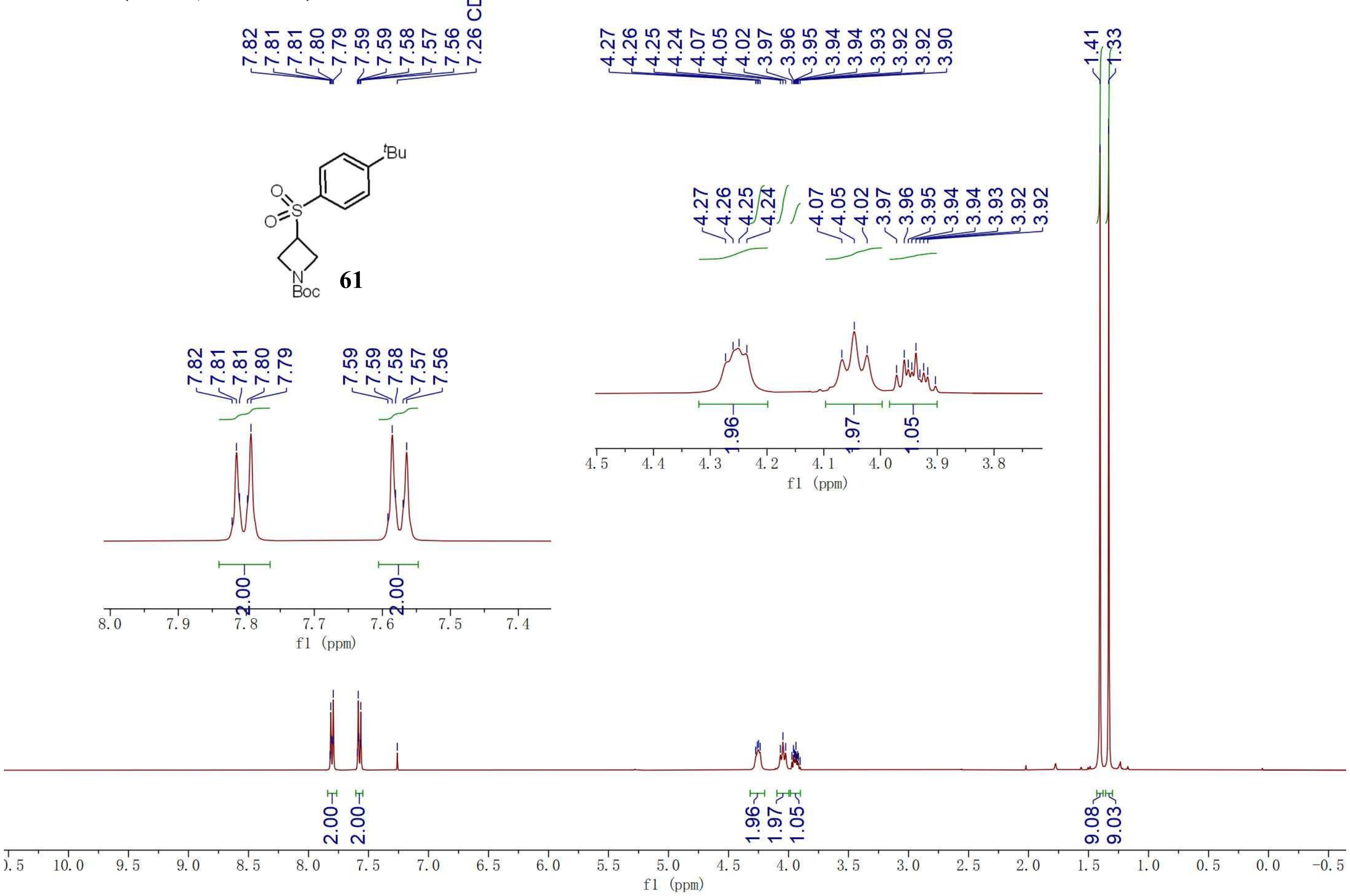

^{13}C NMR ($CDCl_3$, 101 MHz)

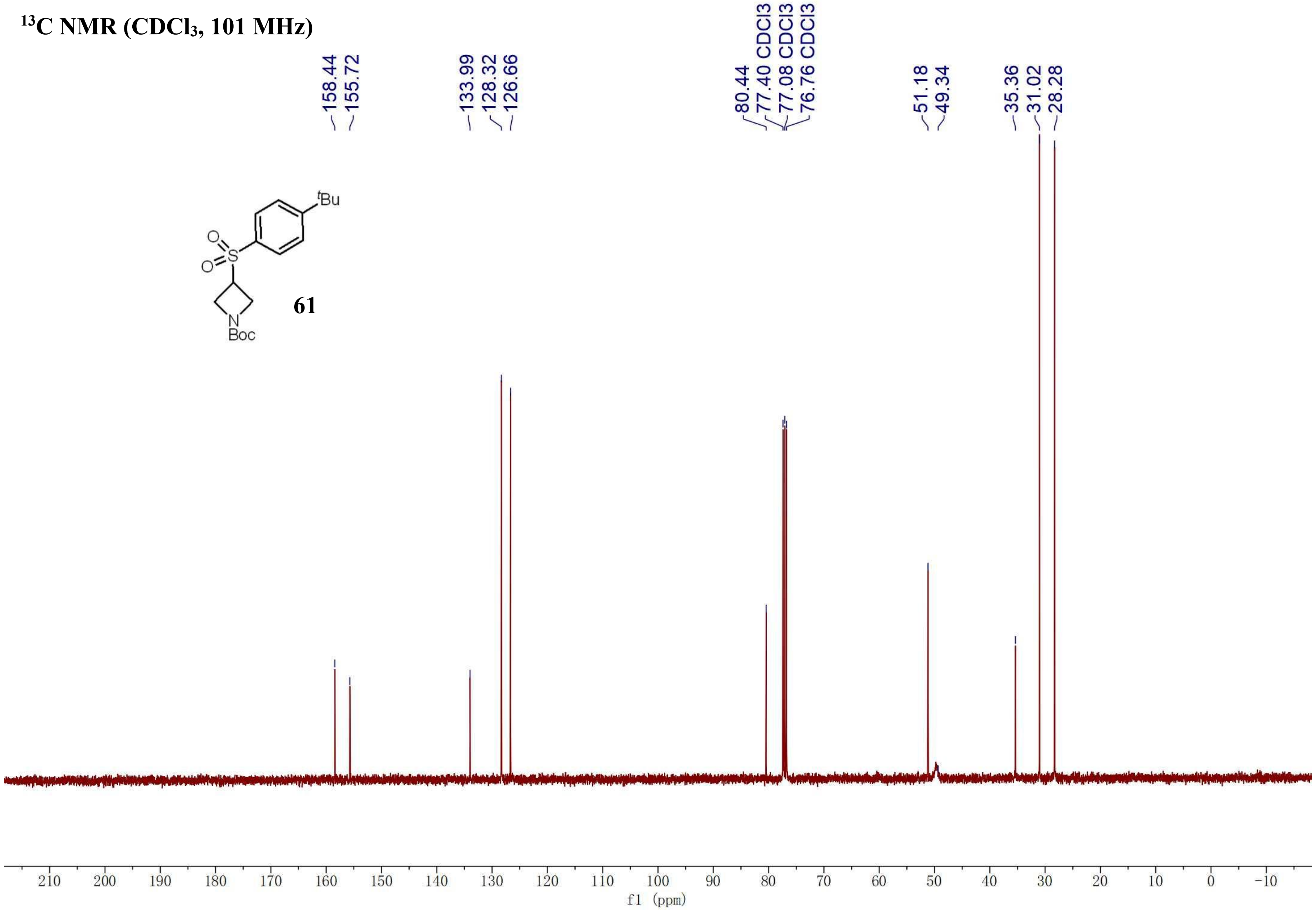

^{1}H NMR ($CDCl_3$, 400 MHz)

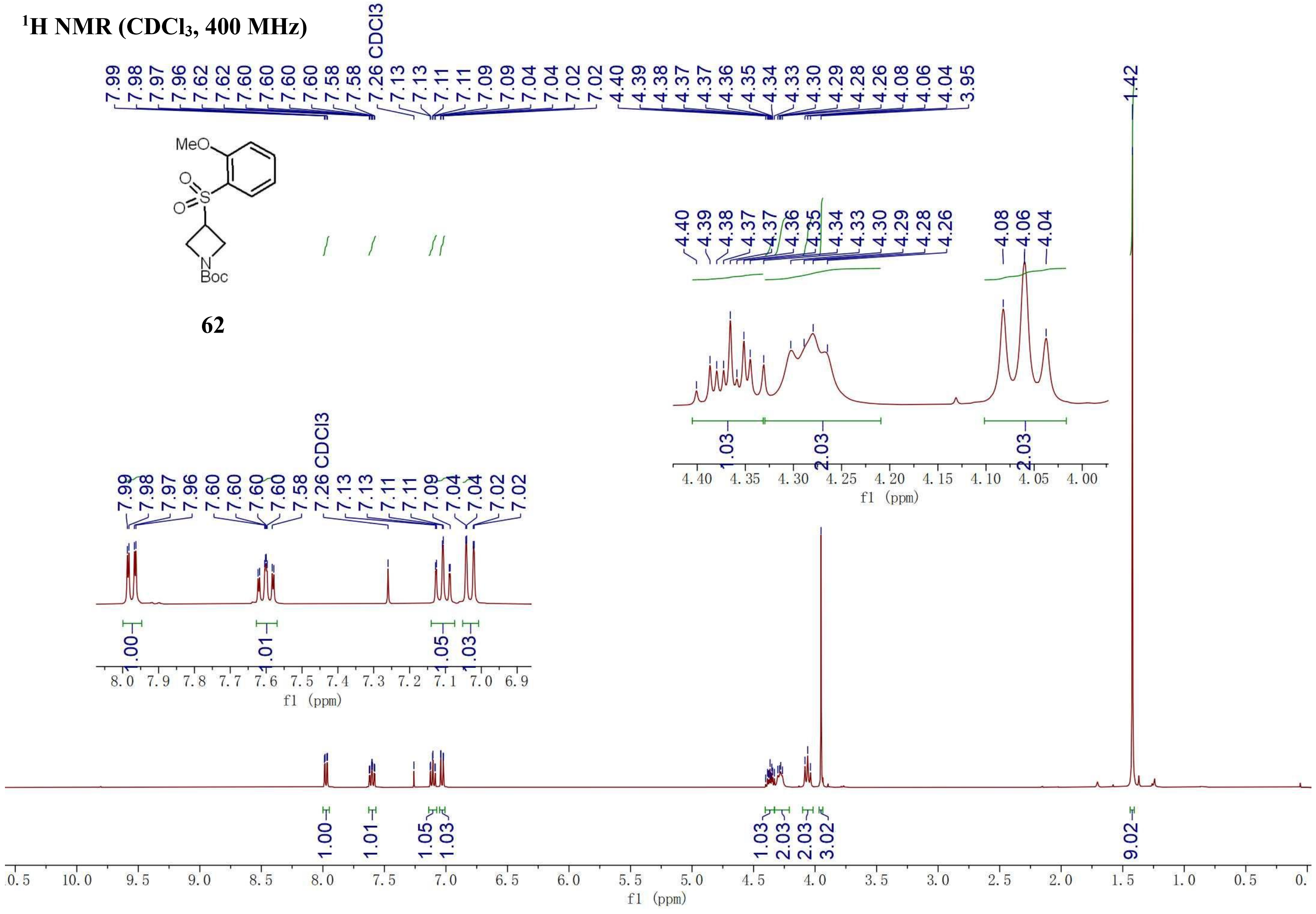

62

^{13}C NMR ($CDCl_3$, 101 MHz)

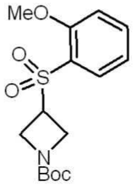

62

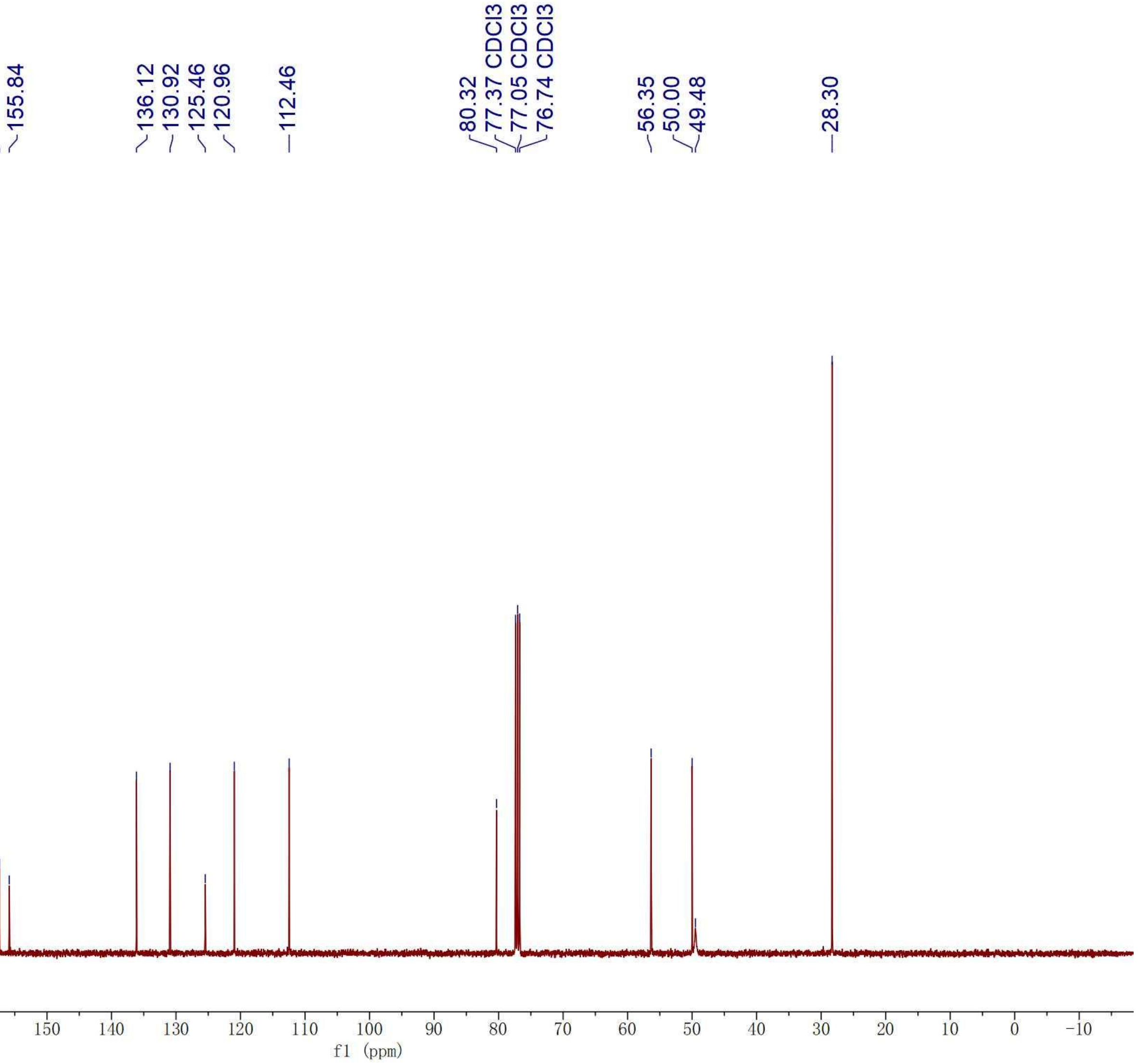

^{1}H NMR ($CDCl_3$, 400 MHz)

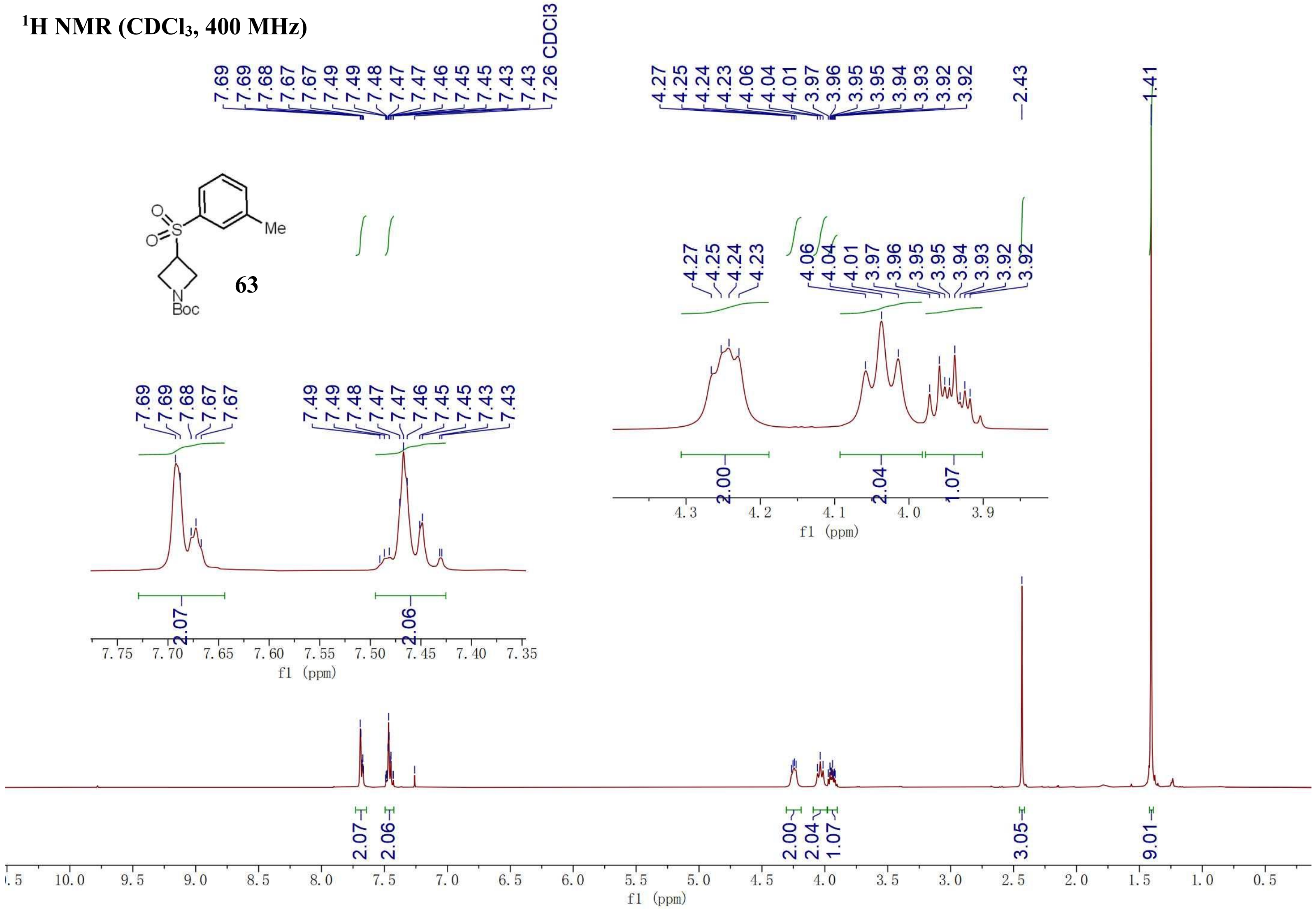

^{13}C NMR ($CDCl_3$, 101 MHz)

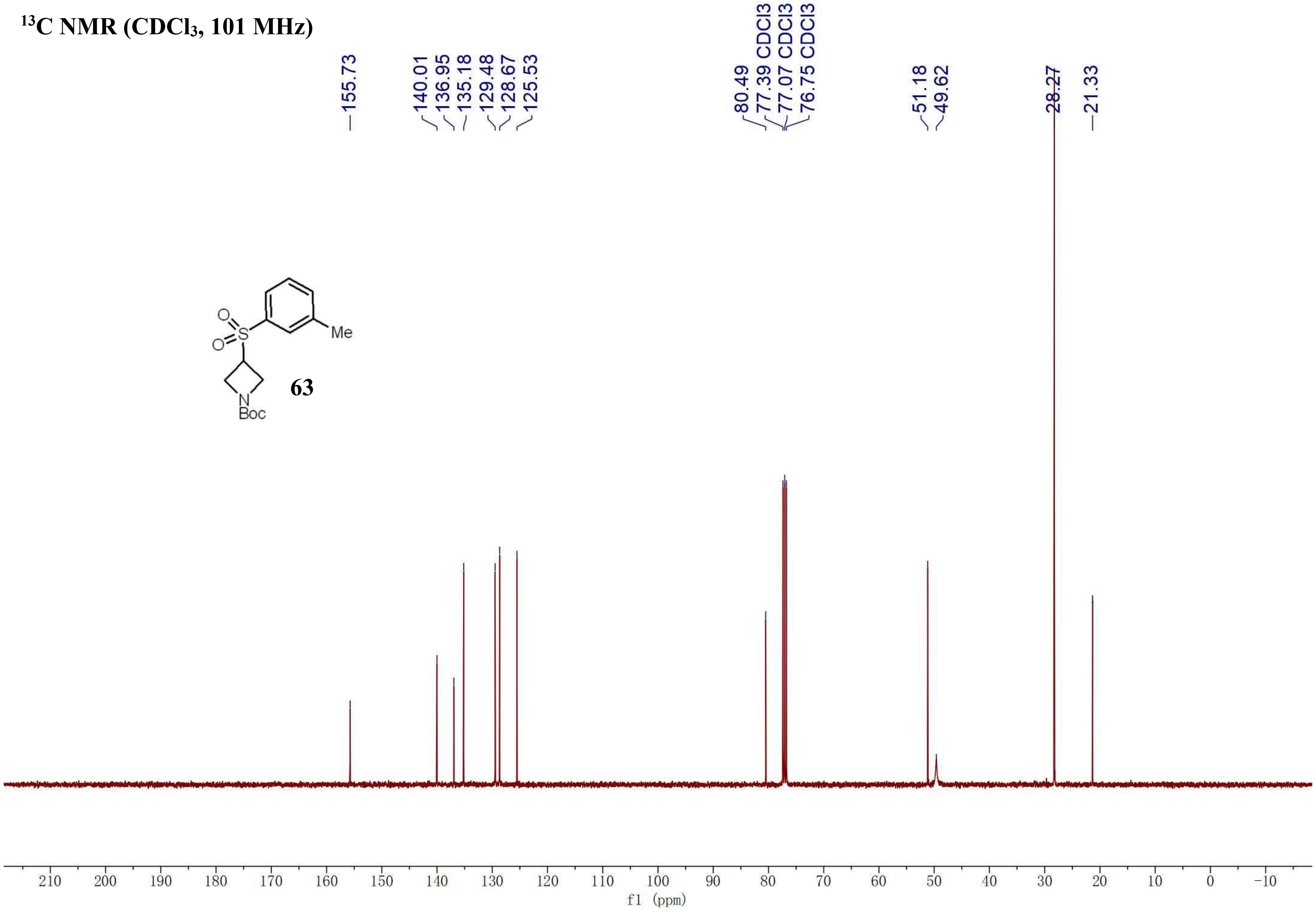

^{1}H NMR ($CDCl_3$, 400 MHz)

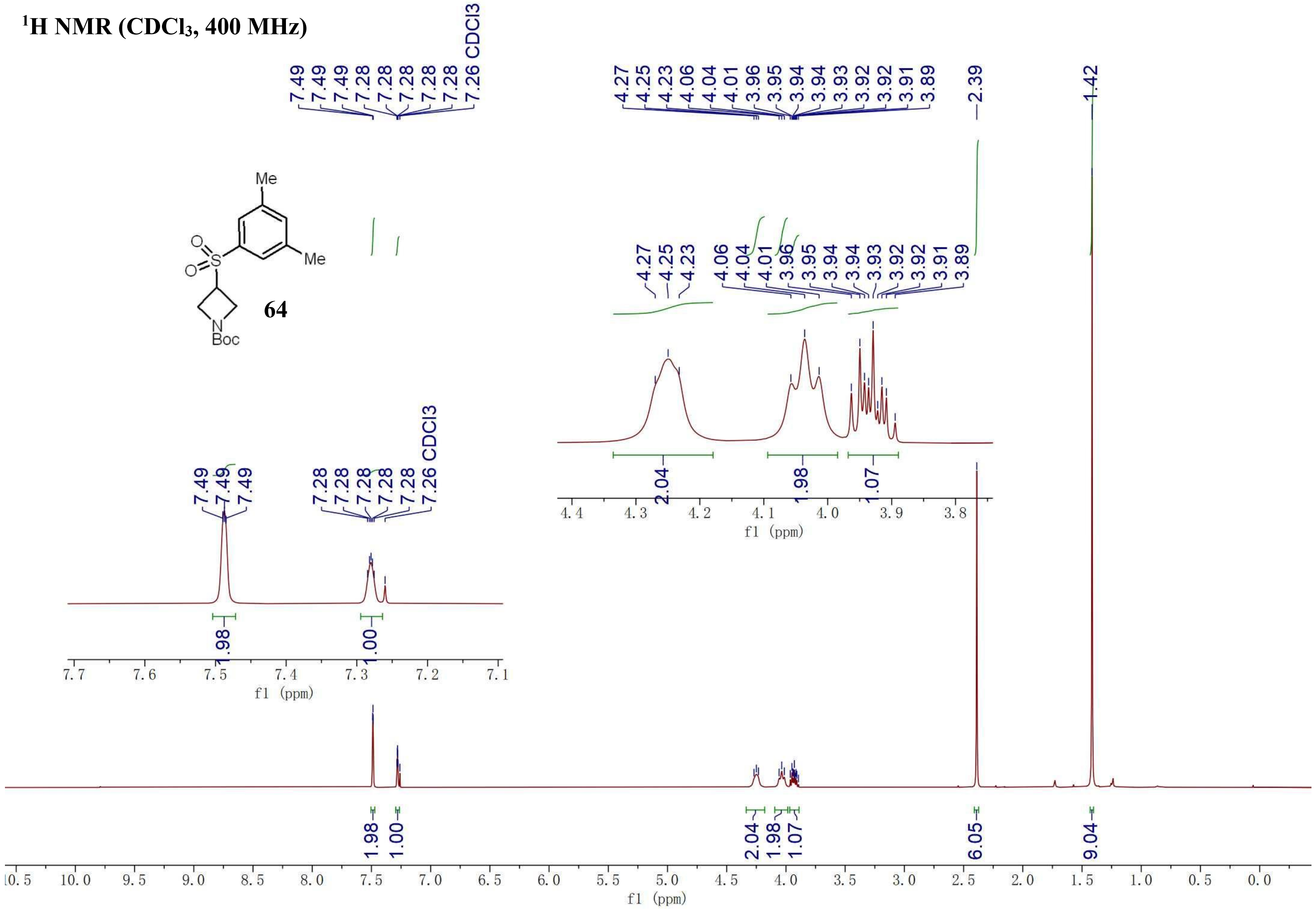

^{13}C NMR ($CDCl_3$, 101 MHz)

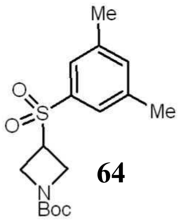

64

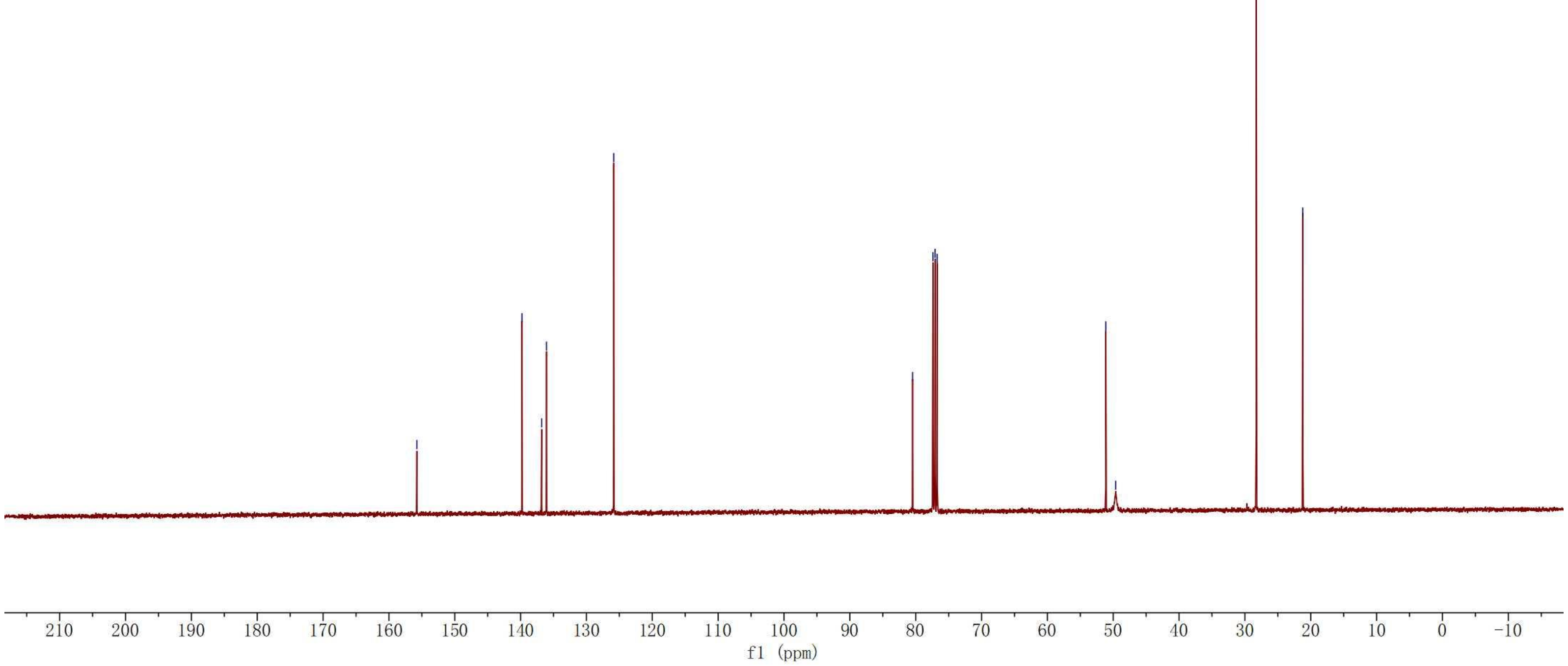

^{1}H NMR ($CDCl_3$, 400 MHz)

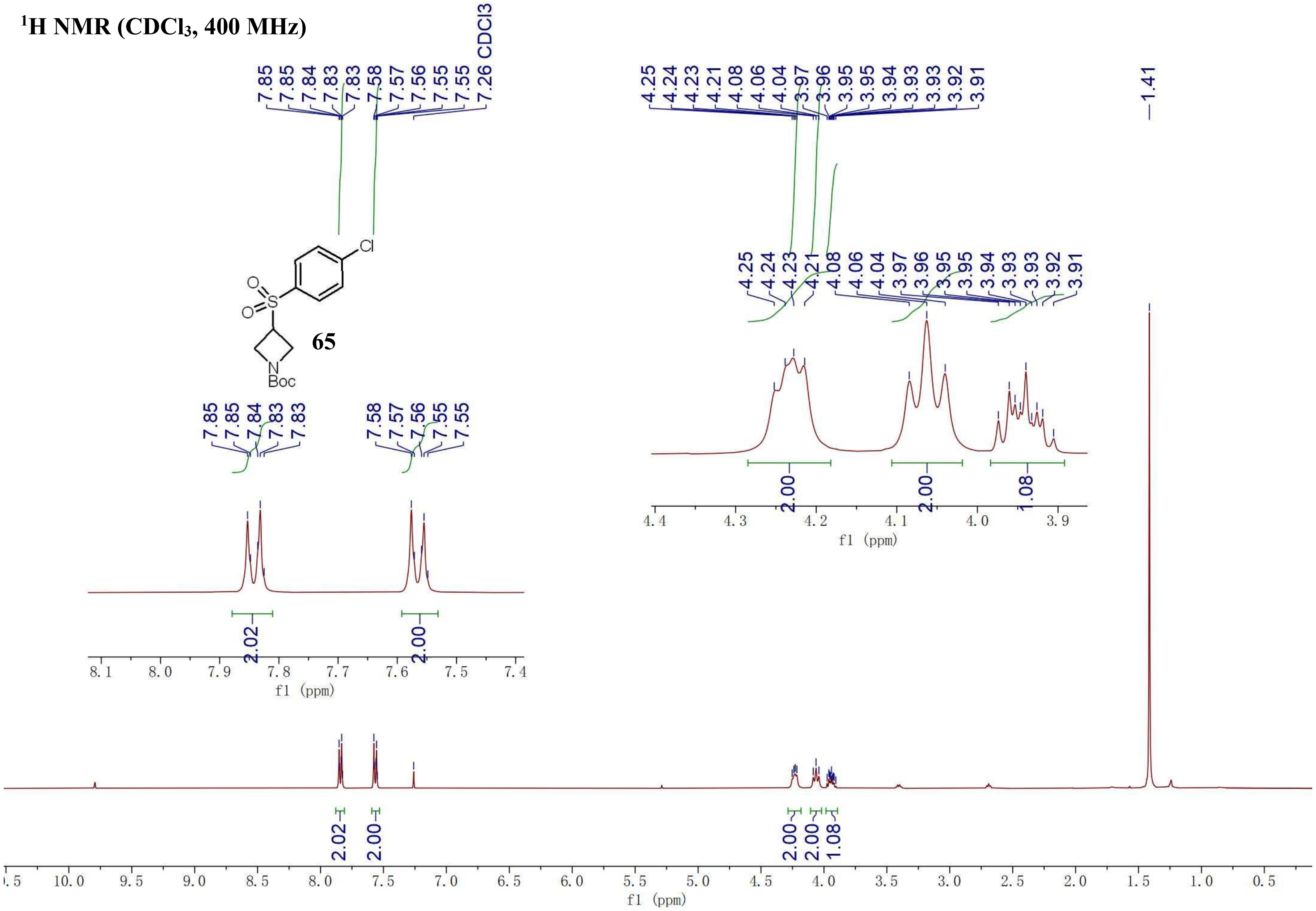

^{13}C NMR ($CDCl_3$, 101 MHz)

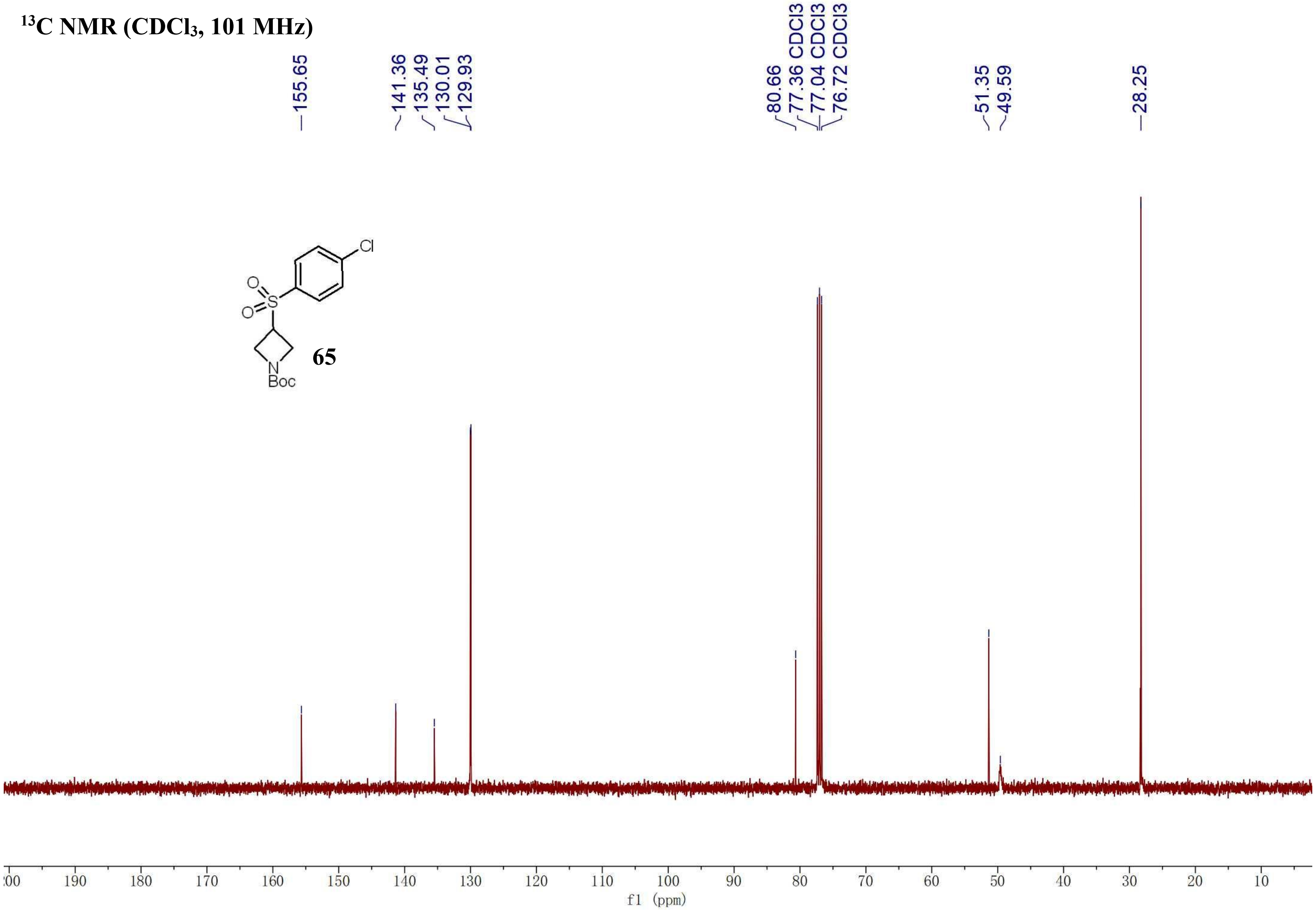

^{1}H NMR ($CDCl_3$, 400 MHz)

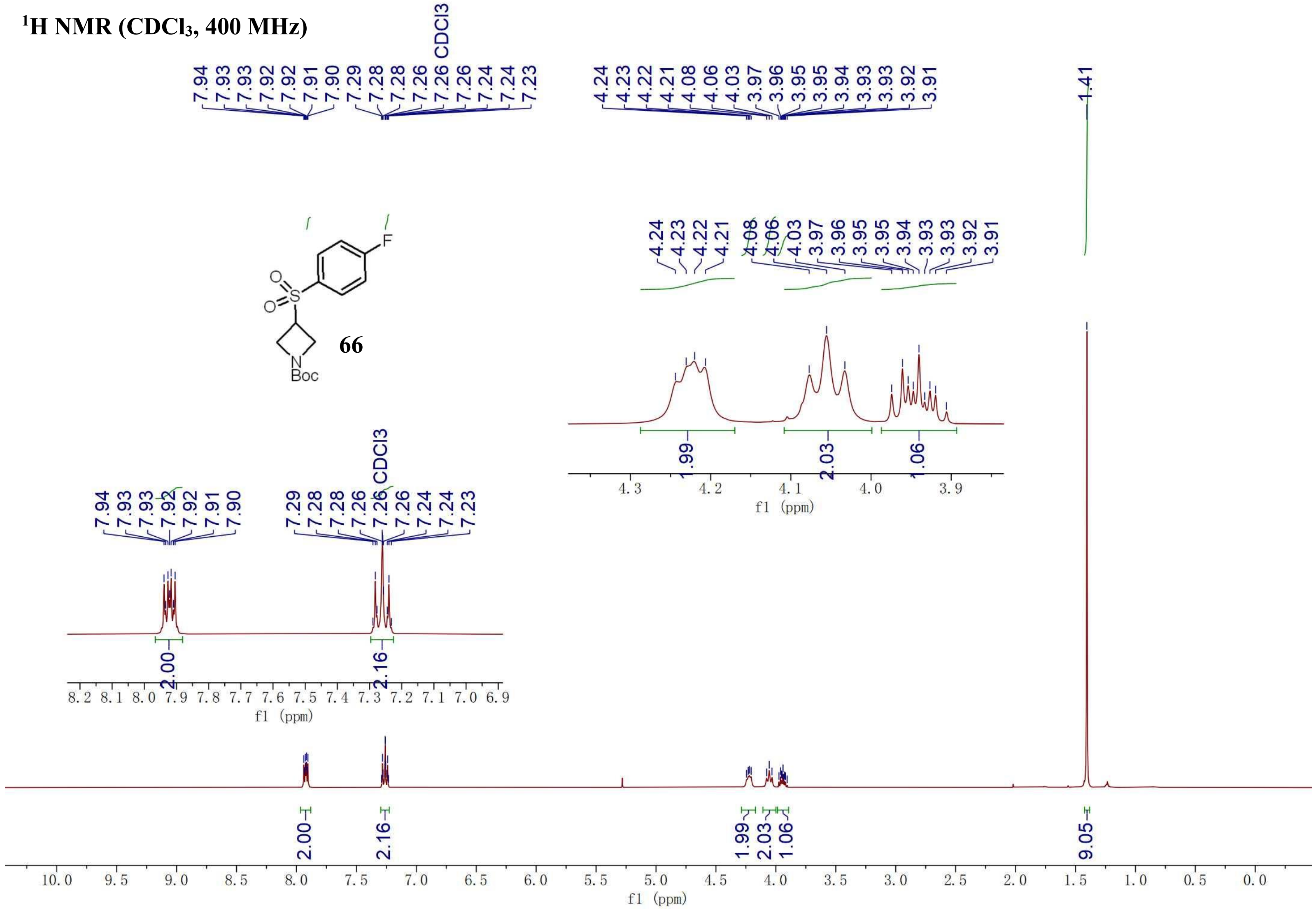

^{19}F NMR ($CDCl_3$, 376 MHz)

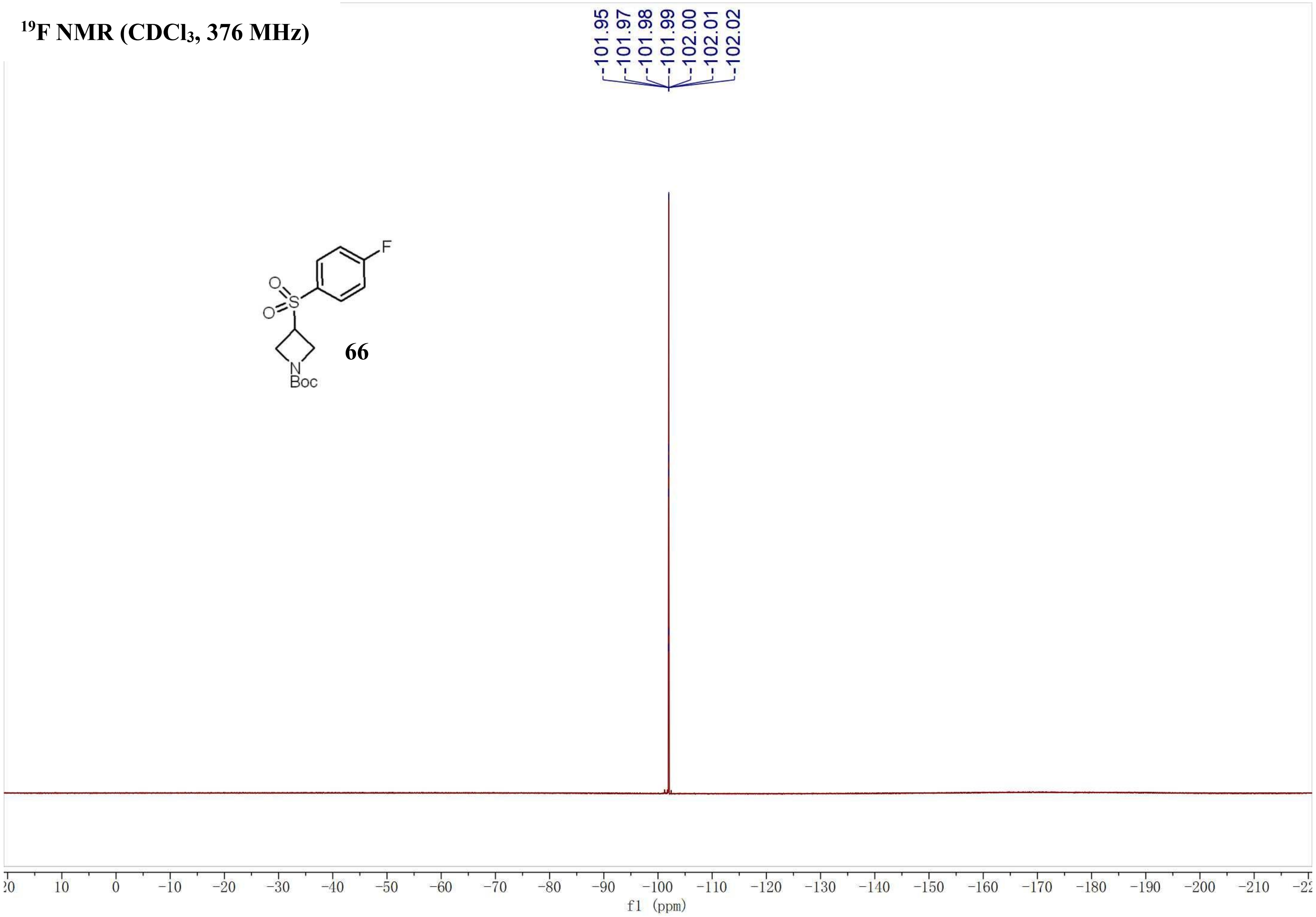

66

^{13}C NMR ($CDCl_3$, 101 MHz)

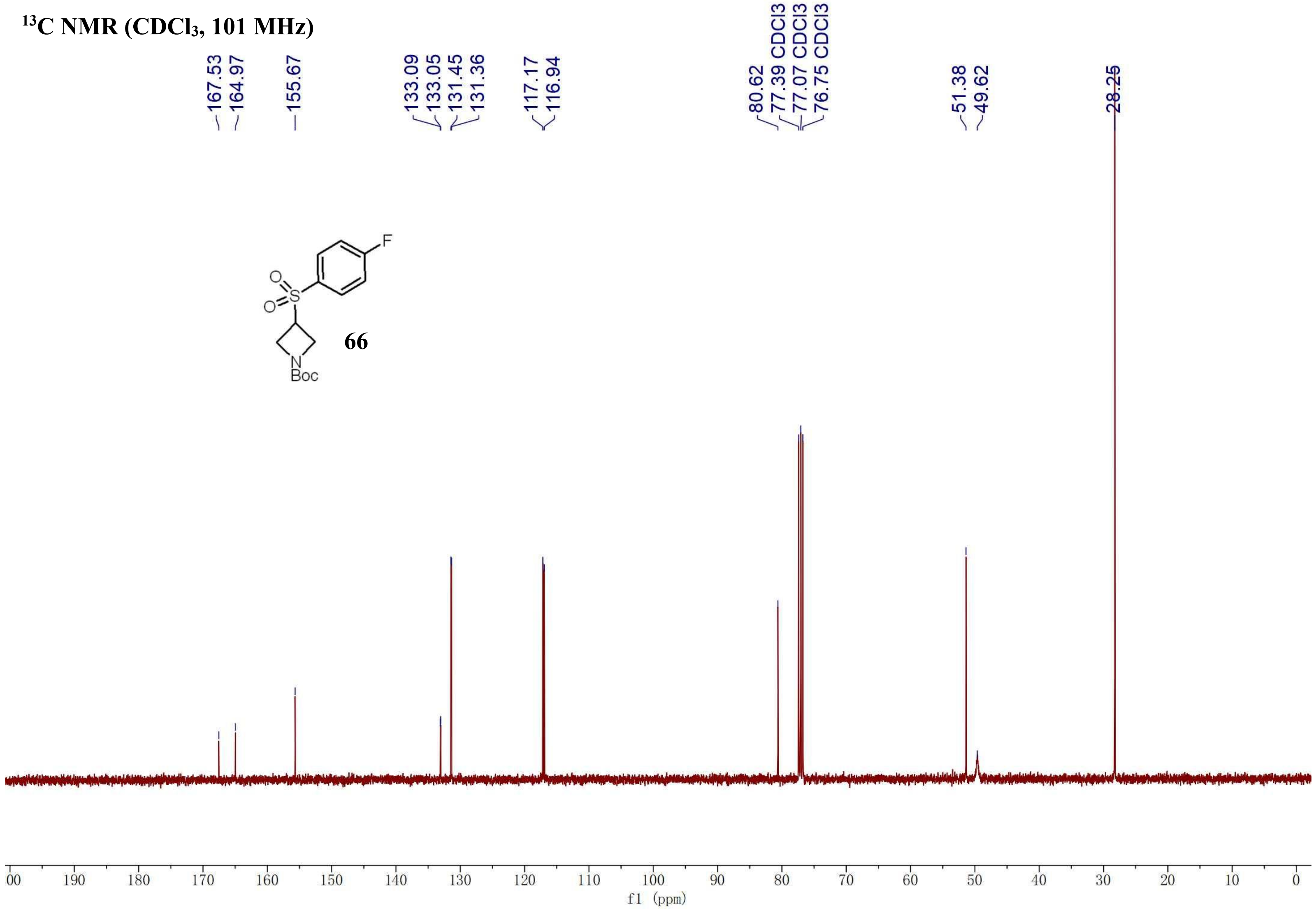

66

^{1}H NMR (CDCl$_3$, 400 MHz)

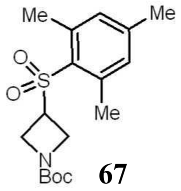

67

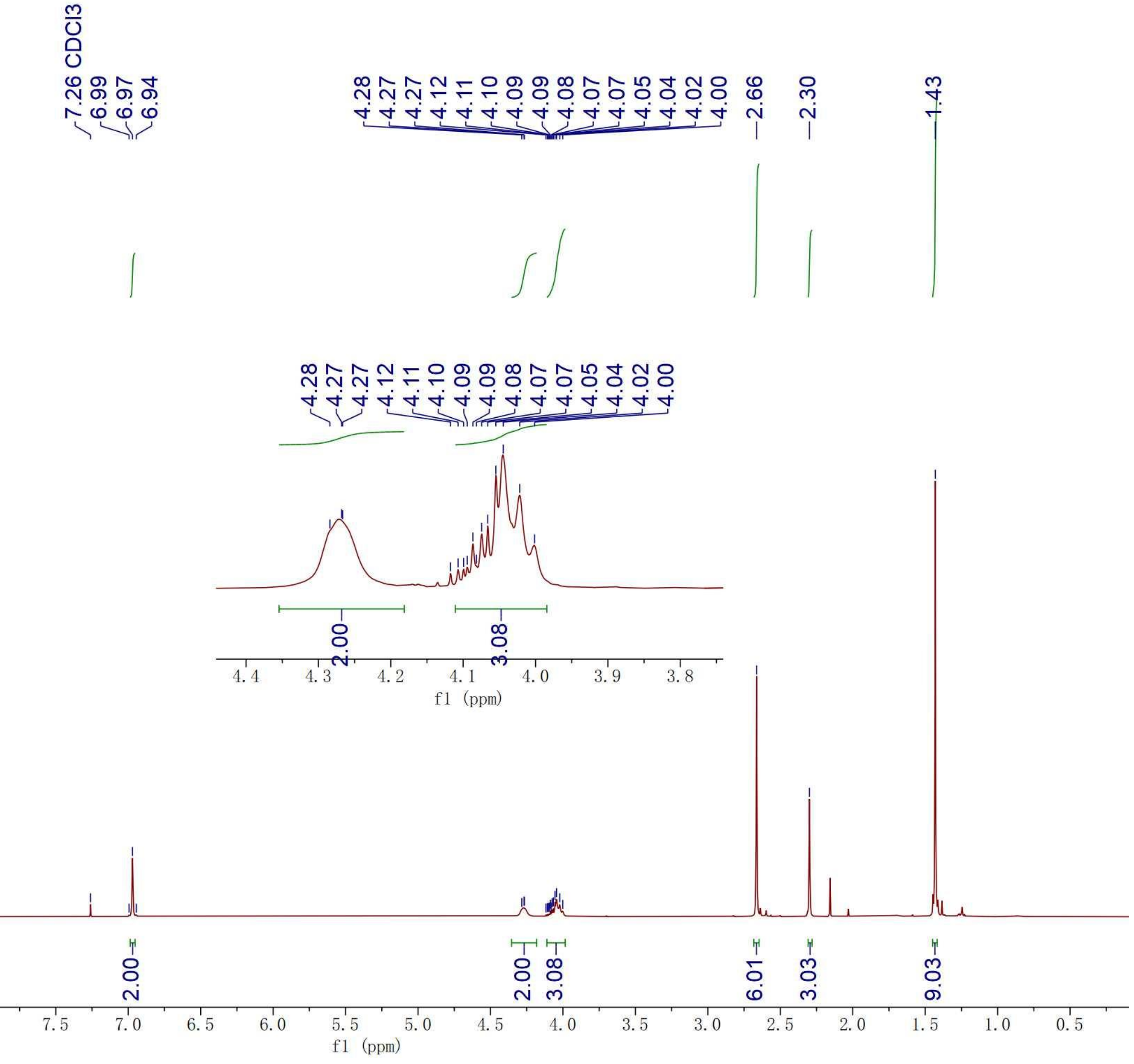

^{13}C NMR ($CDCl_3$, 101 MHz)

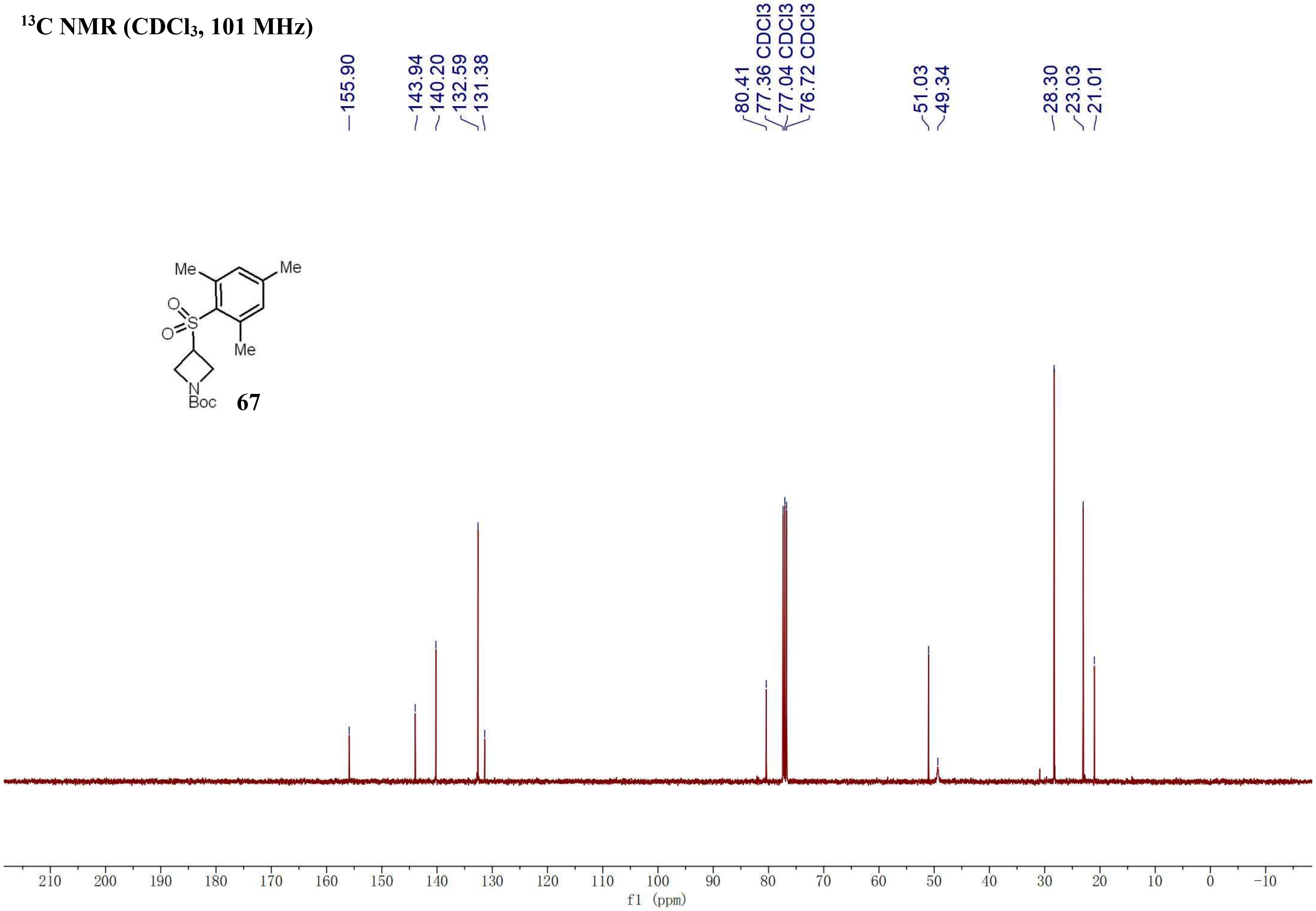

^{1}H NMR ($CDCl_3$, 400 MHz)

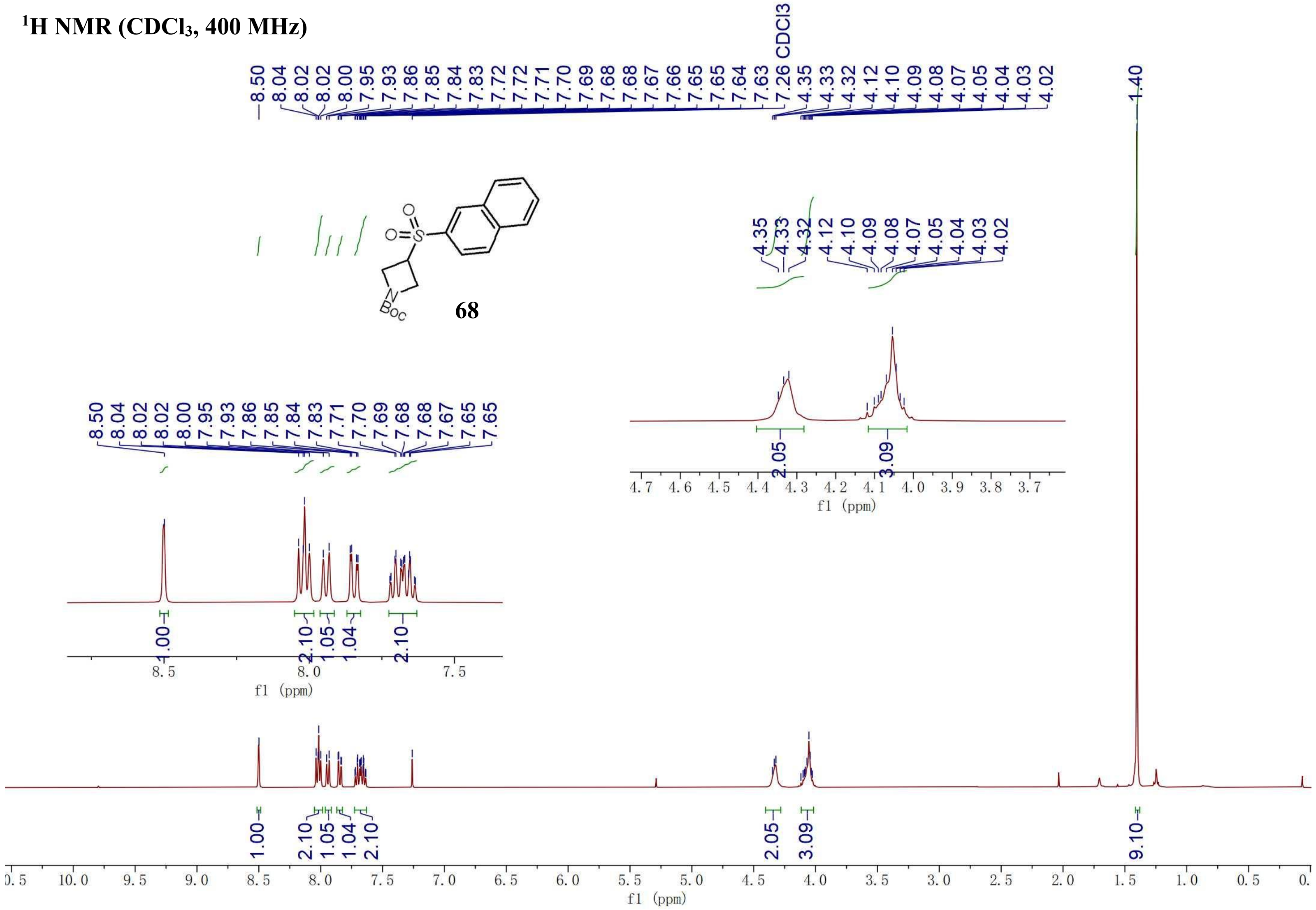

^{13}C NMR ($CDCl_3$, 101 MHz)

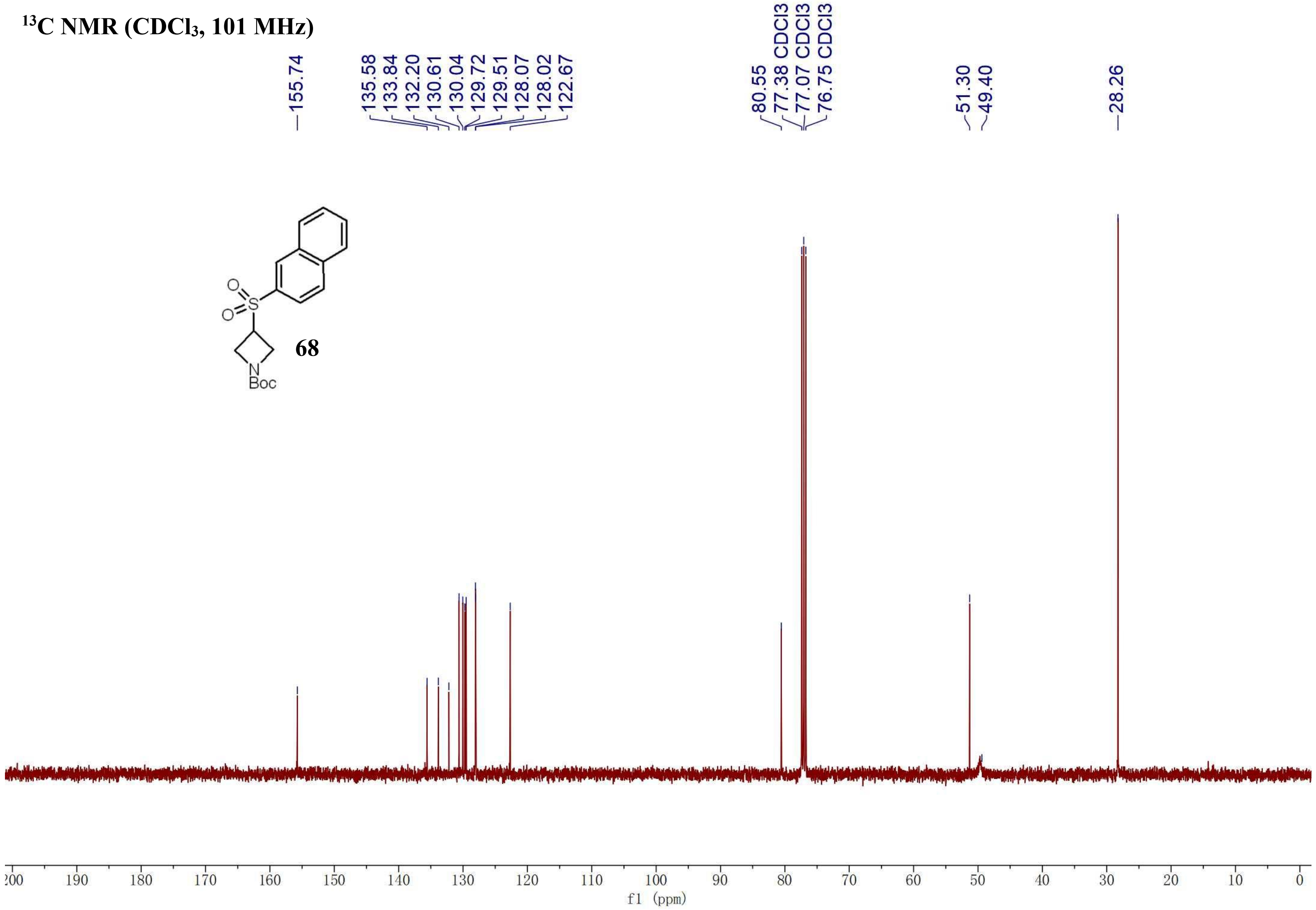

68

^{1}H NMR ($CDCl_3$, 400 MHz)

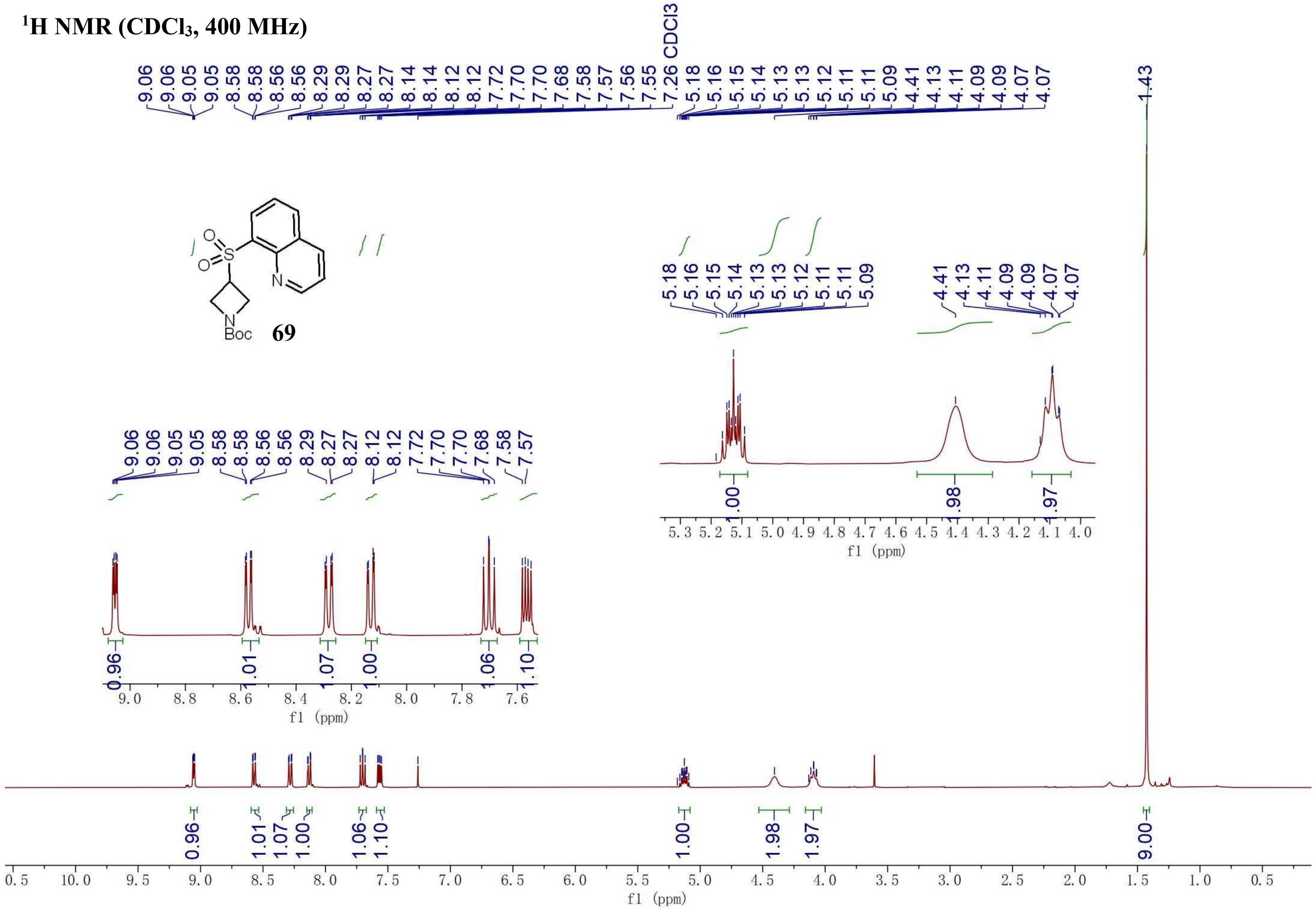

^{13}C NMR ($CDCl_3$, 101 MHz)

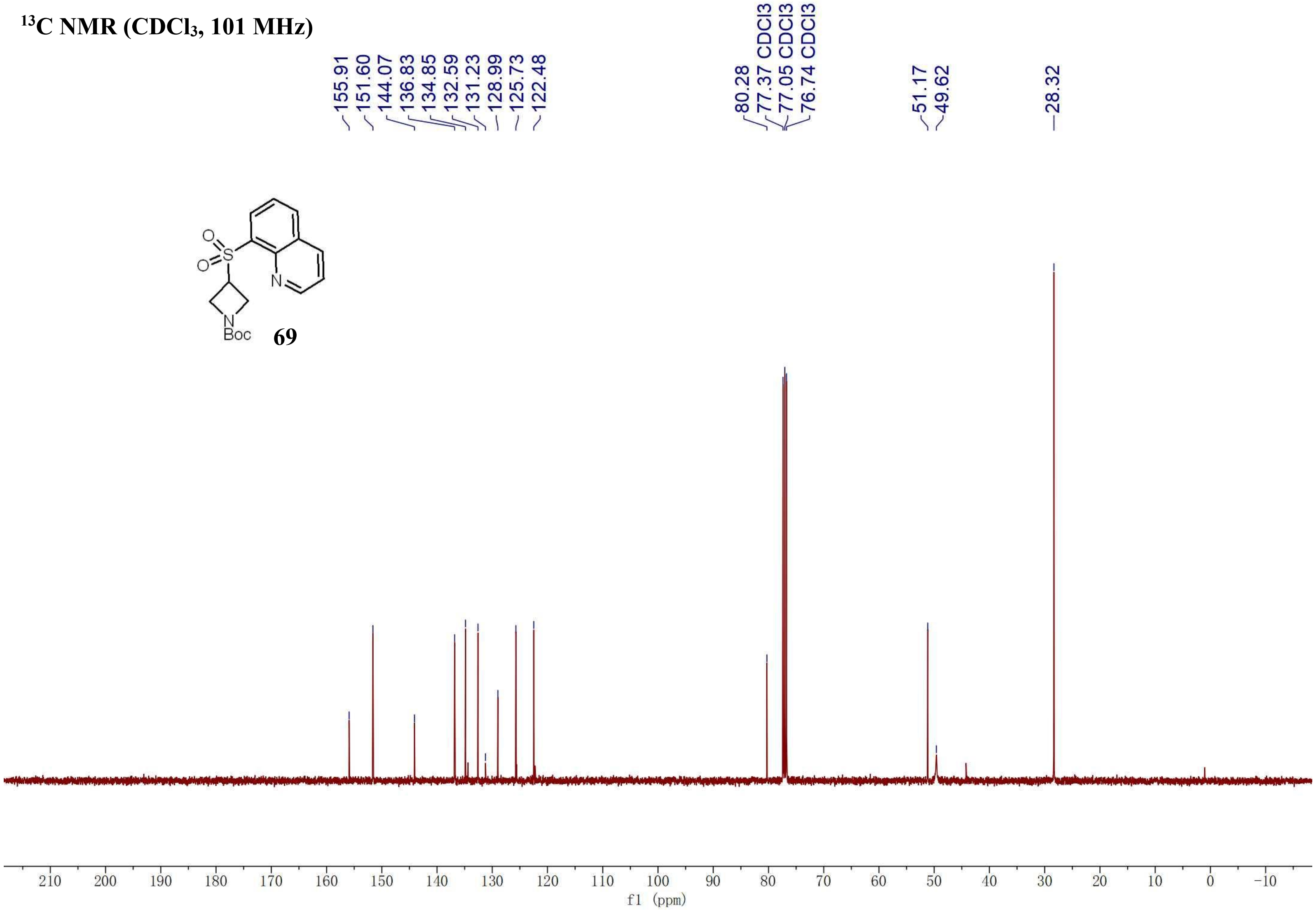

^{1}H NMR ($CDCl_3$, 400 MHz)

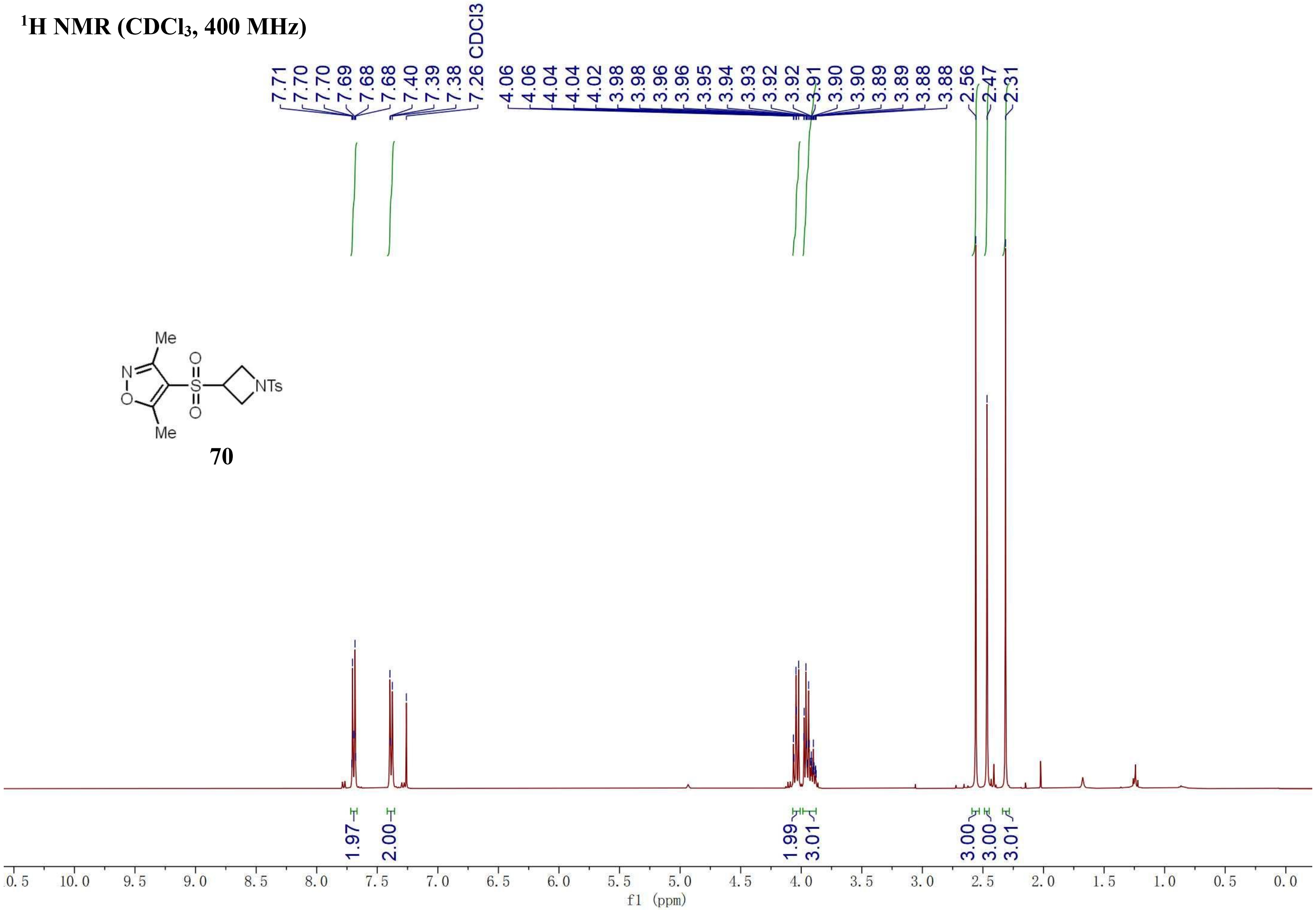

70

^{13}C NMR ($CDCl_3$, 101 MHz)

70

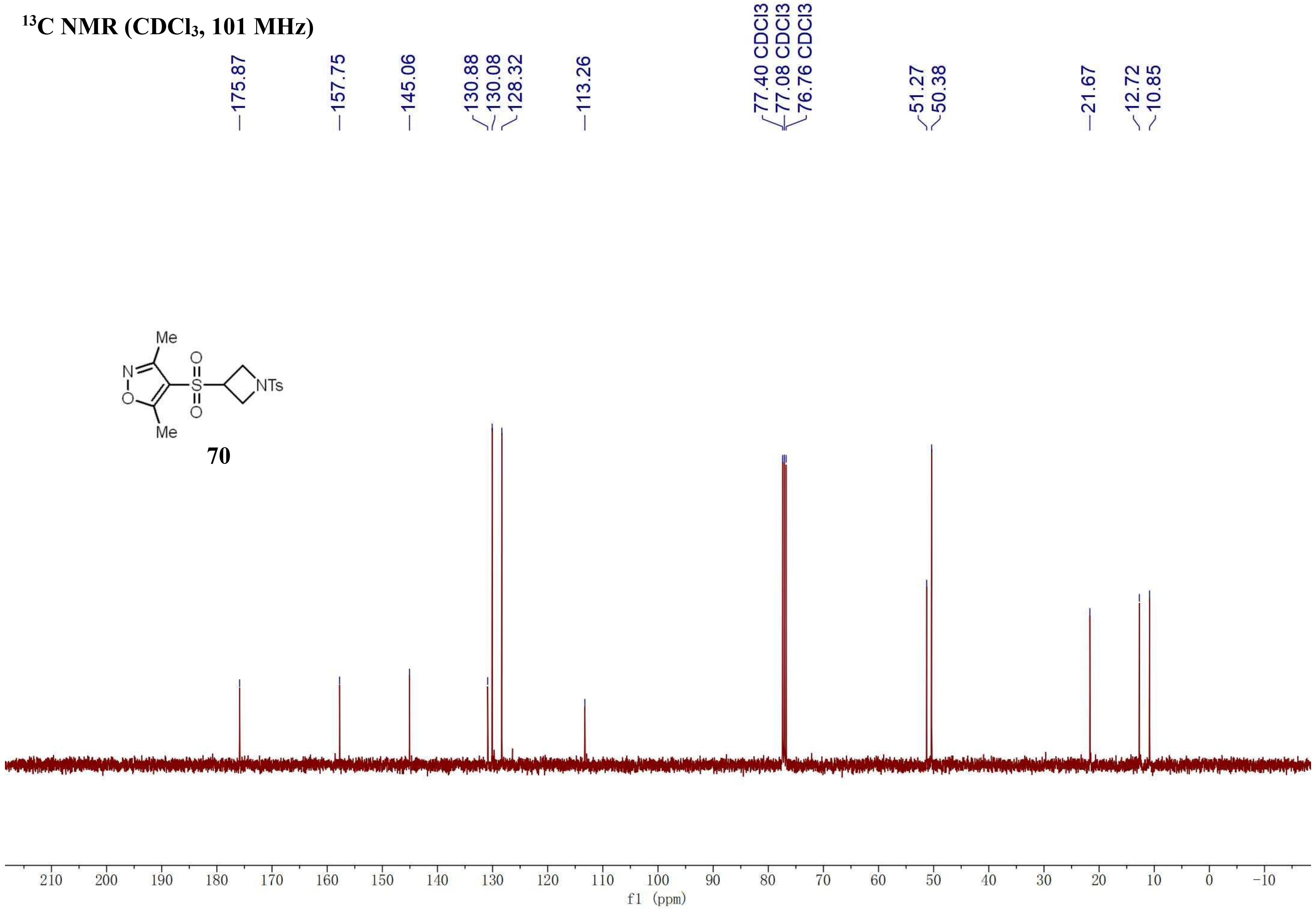

^{1}H NMR ($CDCl_3$, 400 MHz)

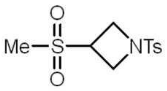

71

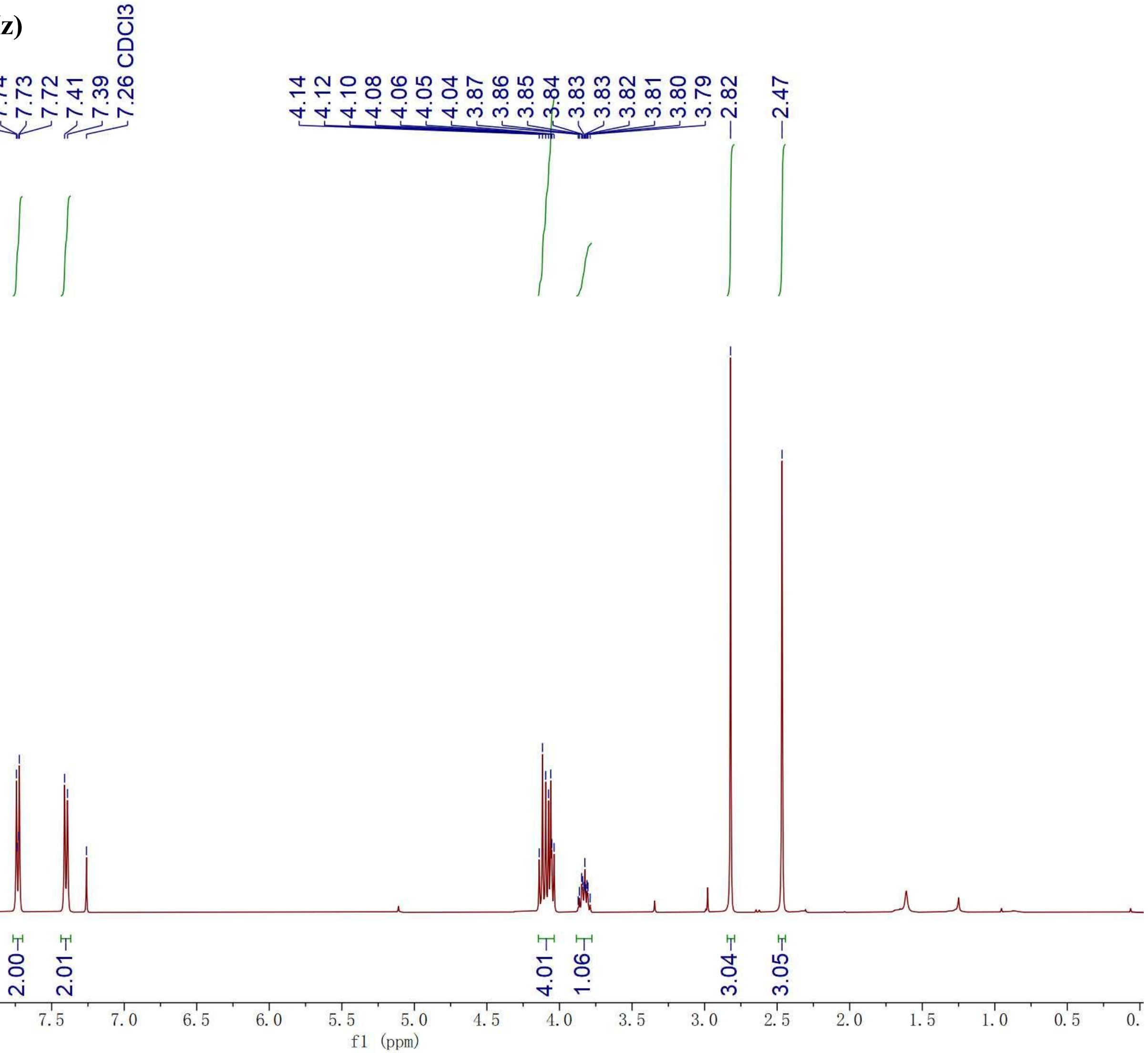

^{13}C NMR ($CDCl_3$, 101 MHz)

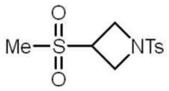

71

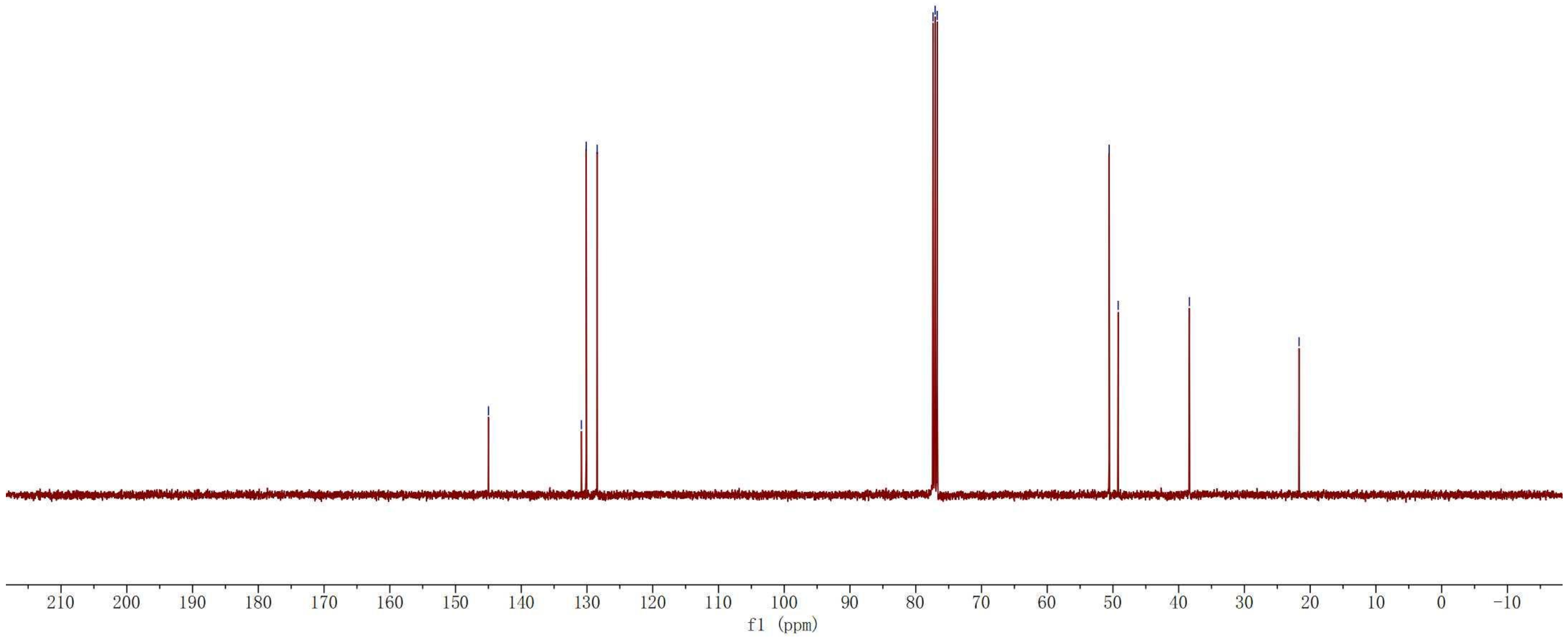

^{1}H NMR ($CDCl_3$, 400 MHz)

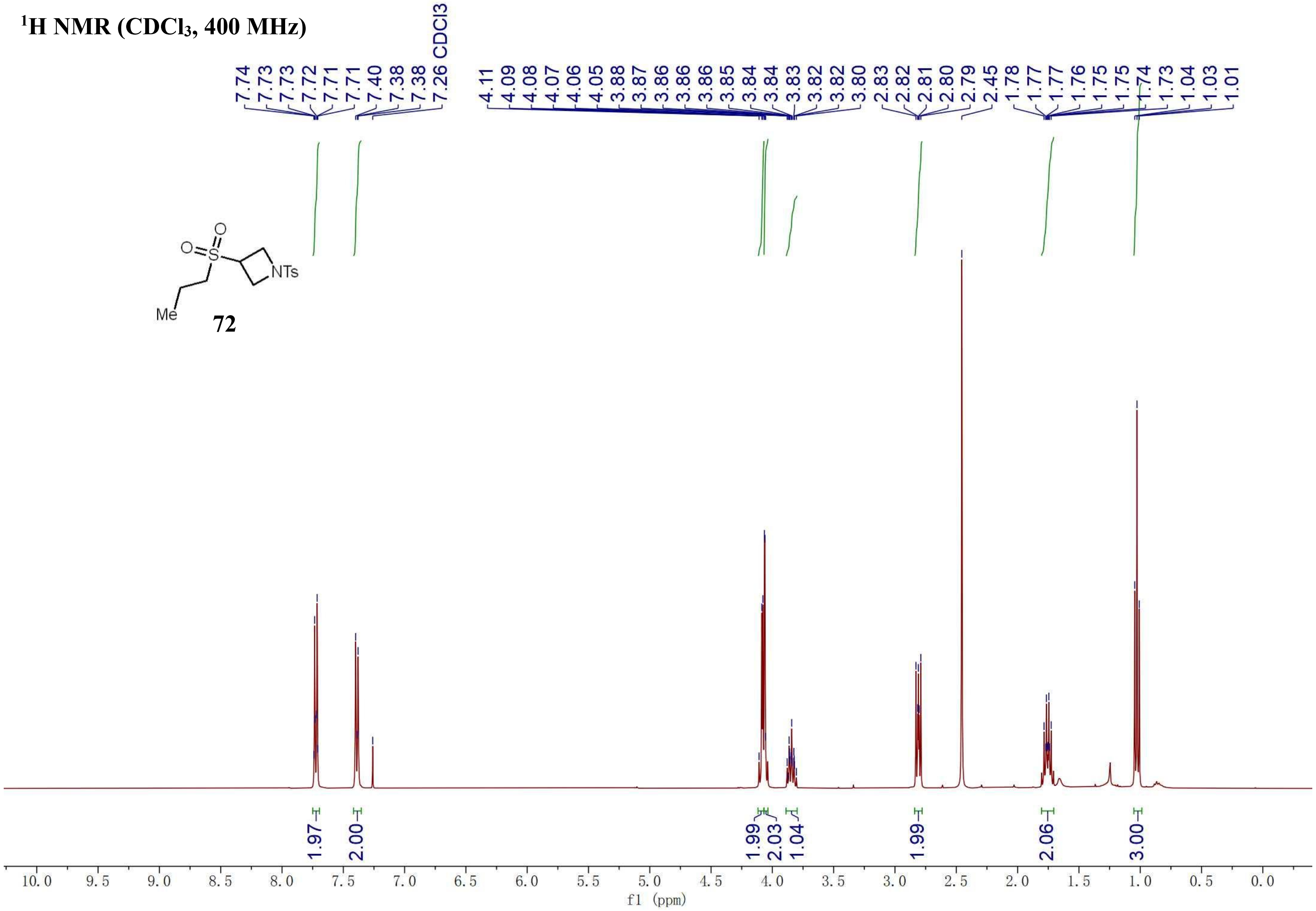

^{13}C NMR ($CDCl_3$, 101 MHz)

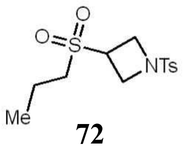

72

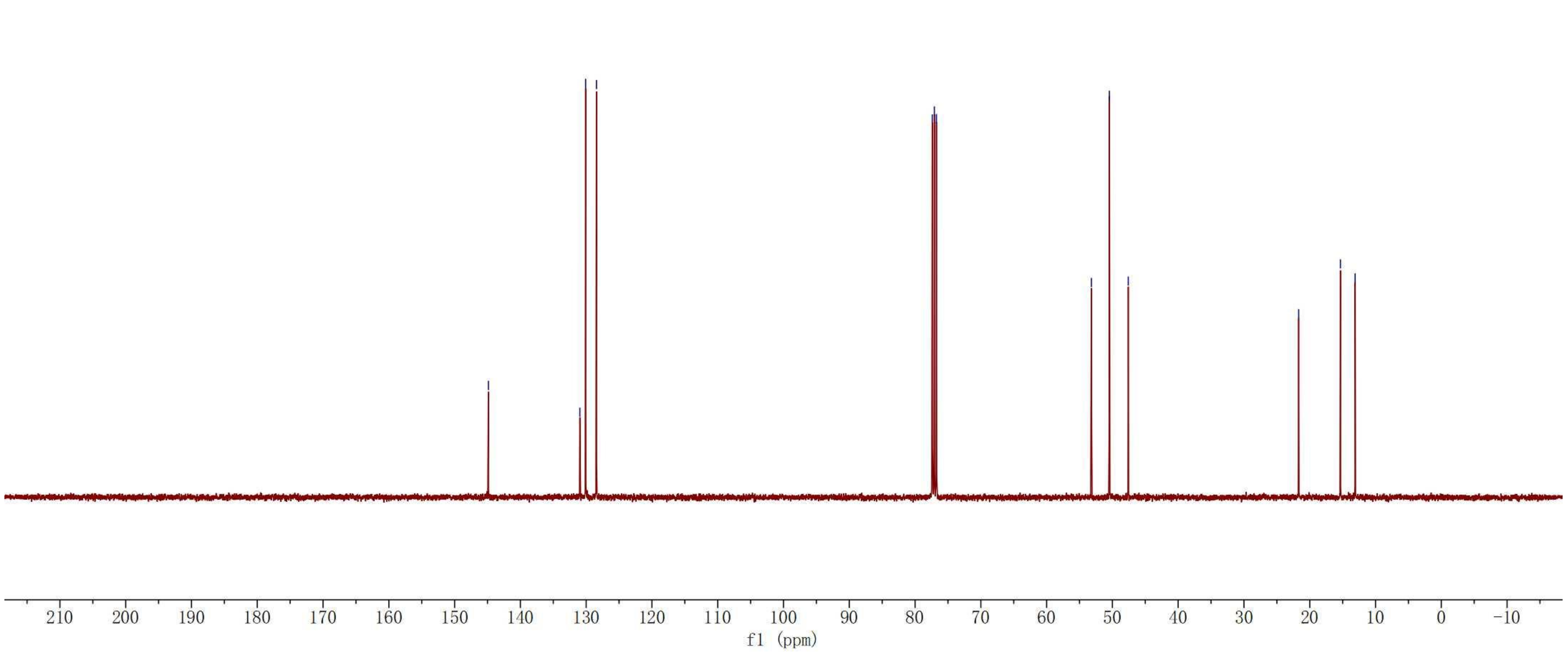

^{1}H NMR (CDCl$_3$, 400 MHz)

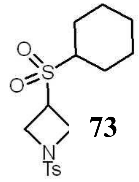

73

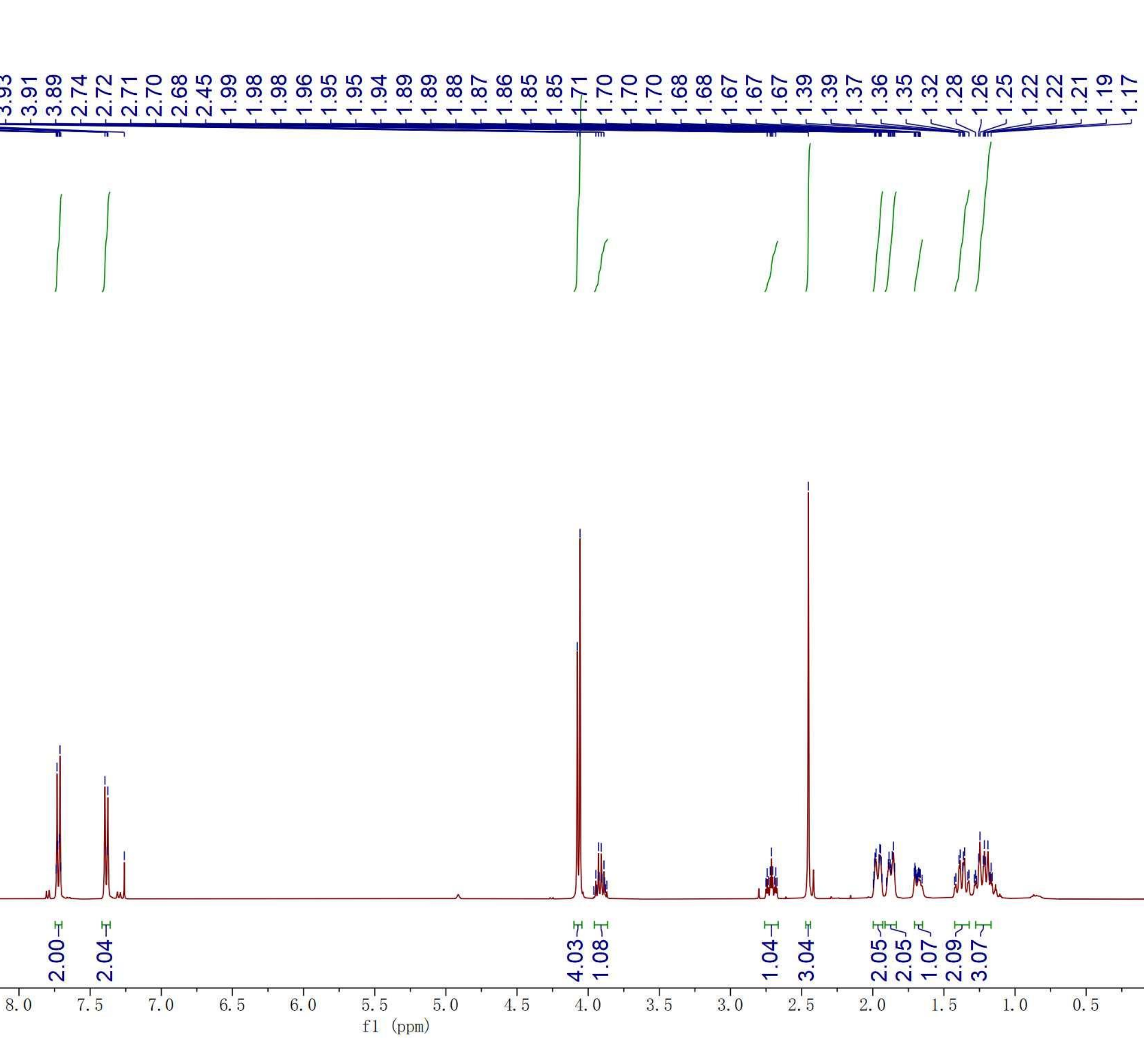

^{13}C NMR ($CDCl_3$, 101 MHz)

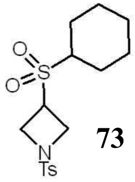

73

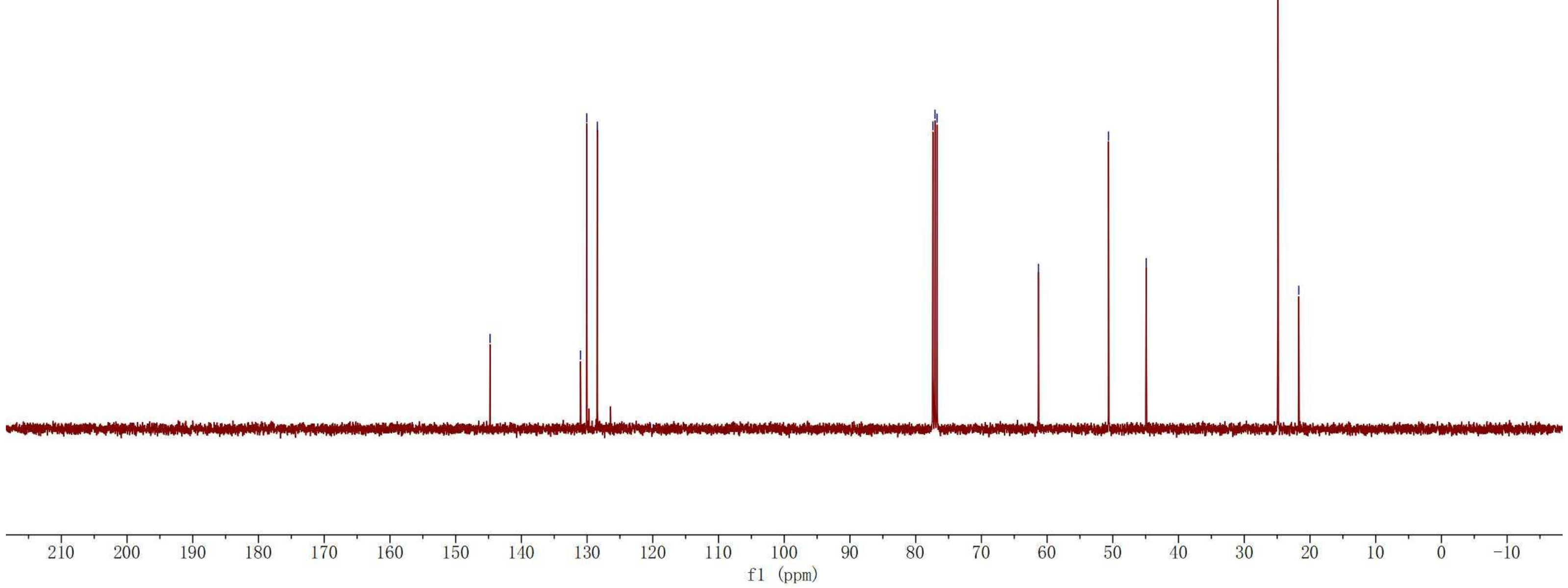

^{1}H NMR ($CDCl_3$, 400 MHz)

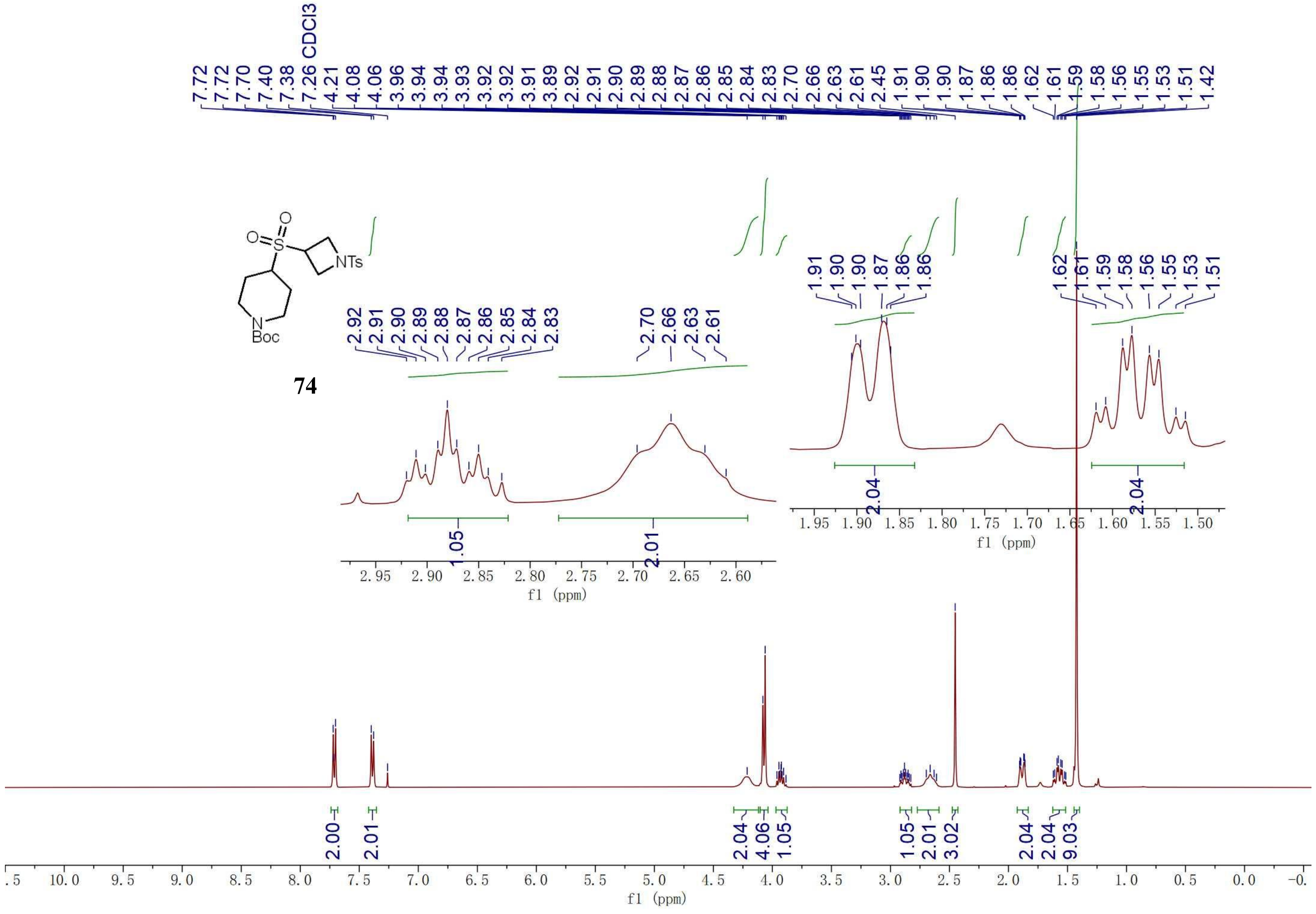

74

^{13}C NMR (CDCl$_3$, 101 MHz)

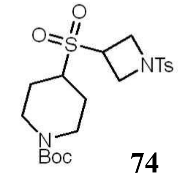

74

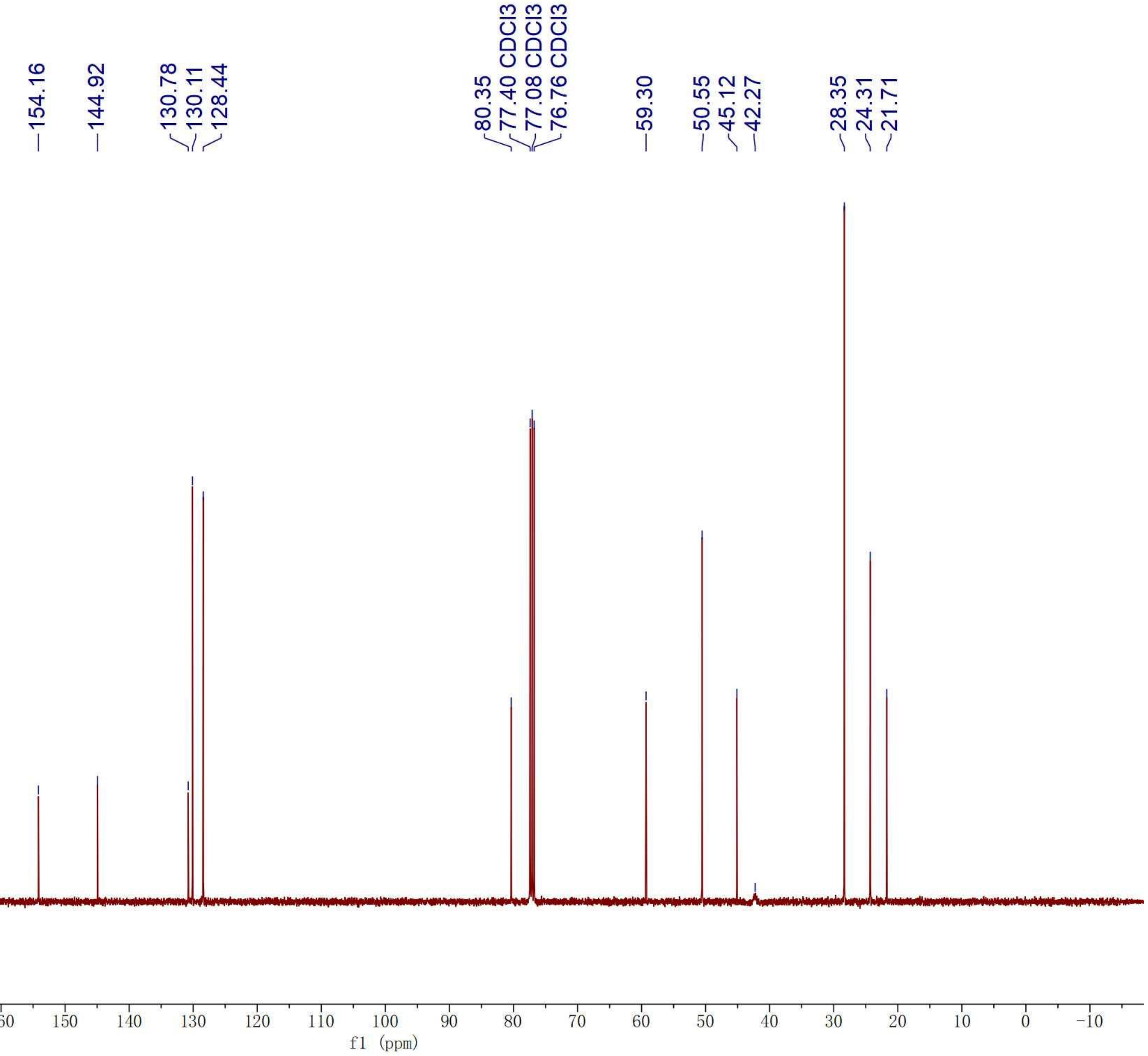

^{1}H NMR ($CDCl_3$, 400 MHz)

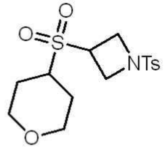

75

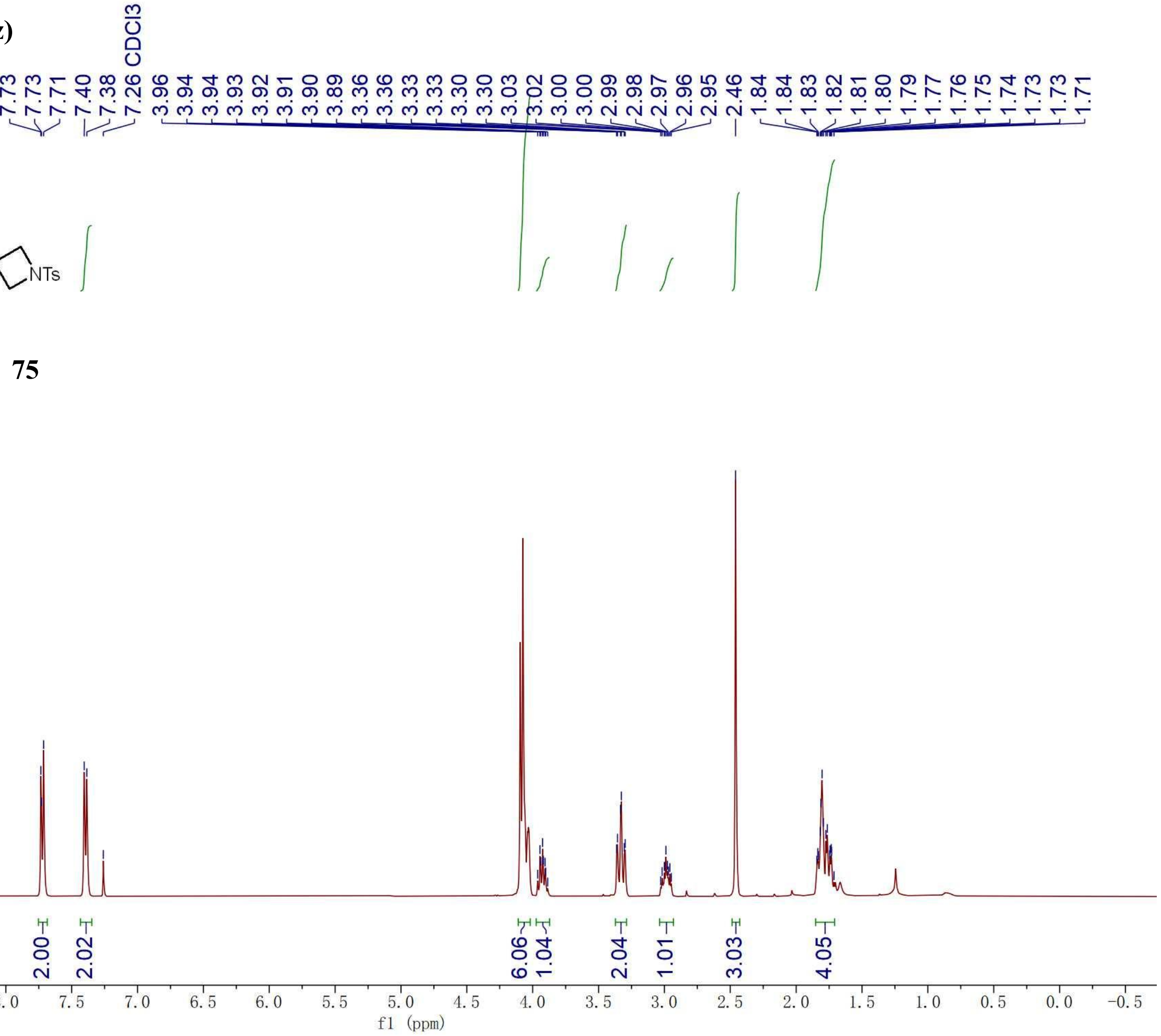

^{13}C NMR (CDCl$_3$, 101 MHz)

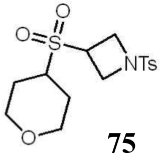

75

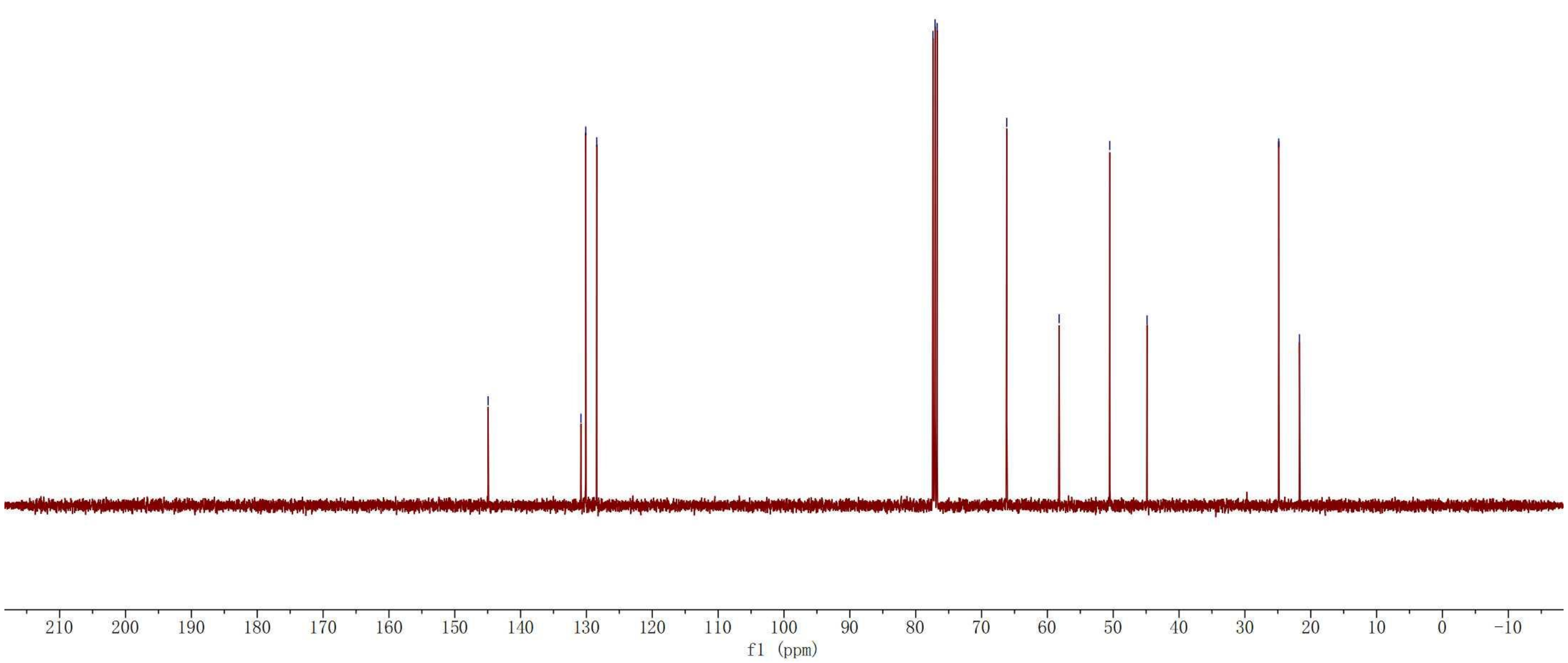

^{1}H NMR ($CDCl_3$, 400 MHz)

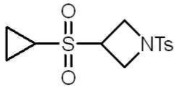

76

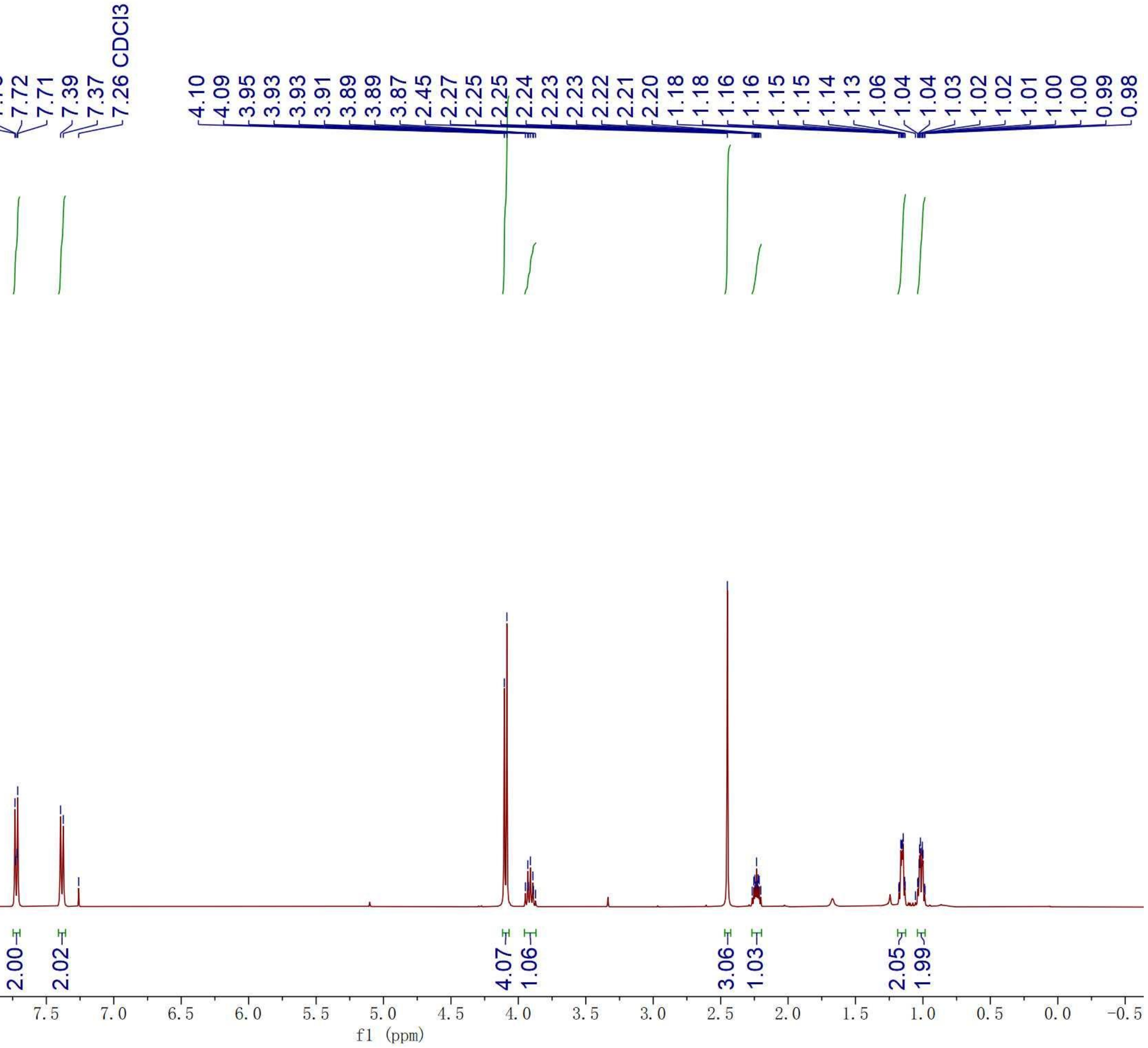

^{13}C NMR ($CDCl_3$, 101 MHz)

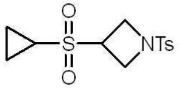

76

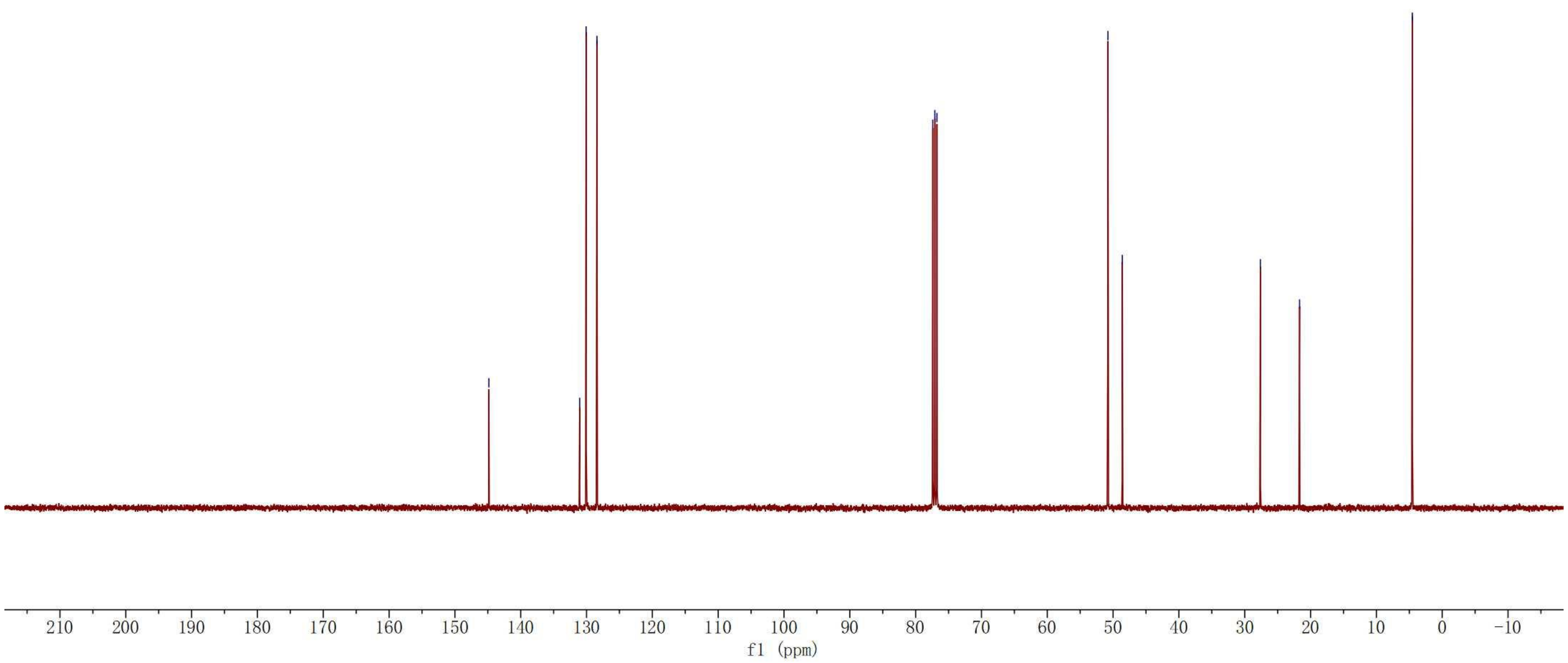

^{1}H NMR ($CDCl_3$, 400 MHz)

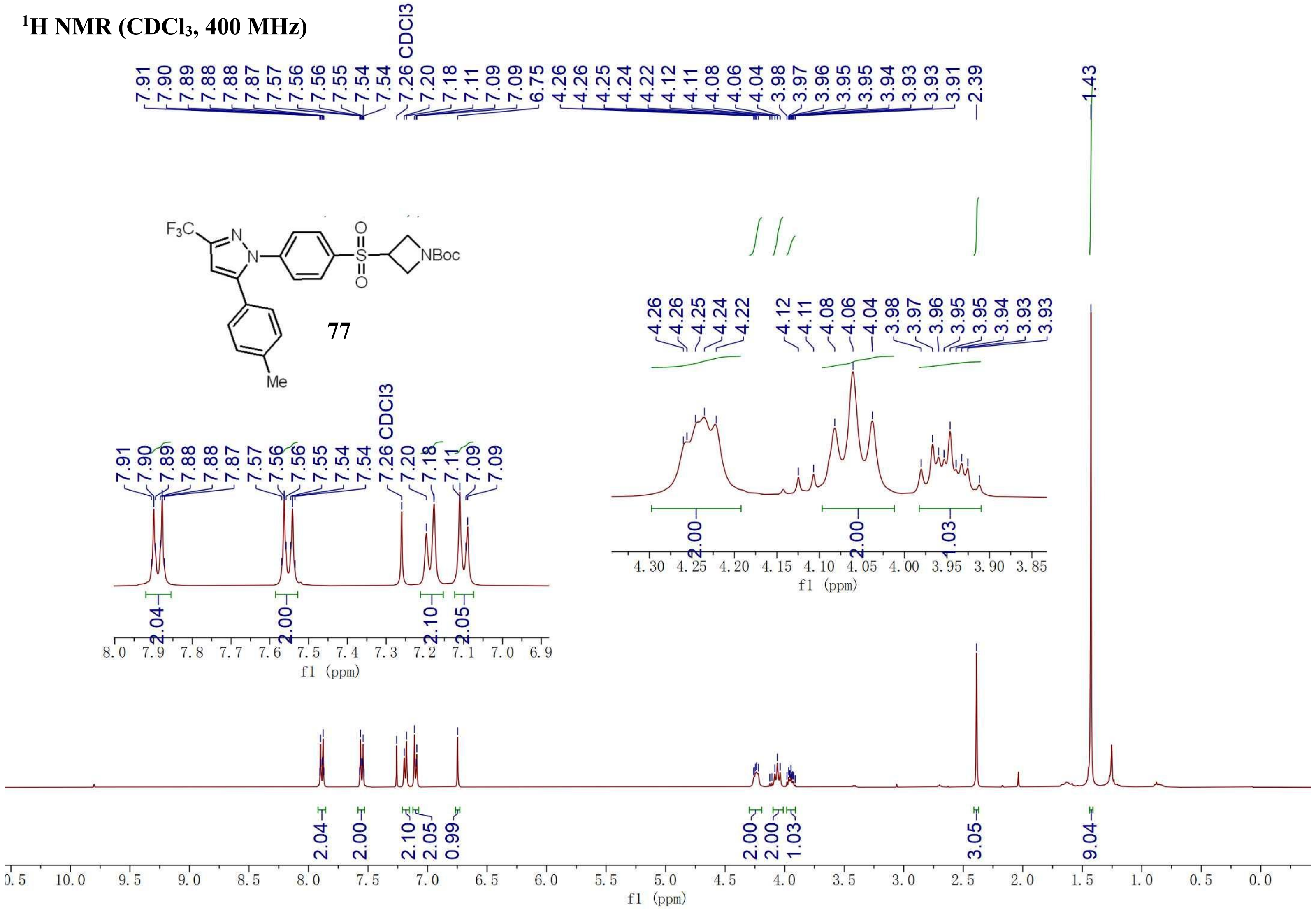

^{19}F NMR ($CDCl_3$, 376 MHz)

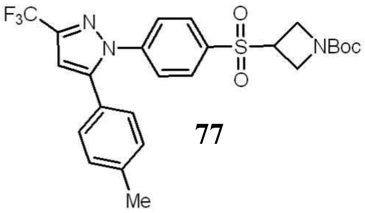

77

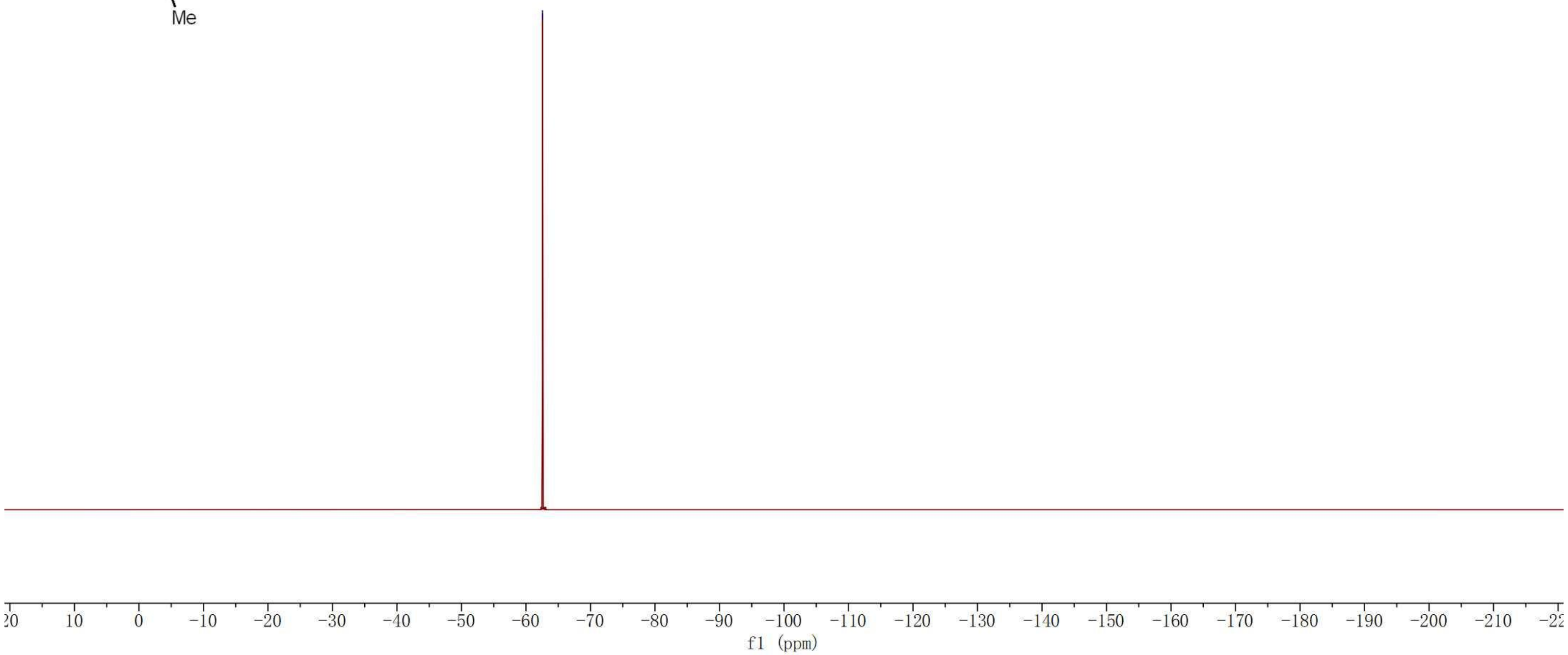

^{13}C NMR ($CDCl_3$, 101 MHz)

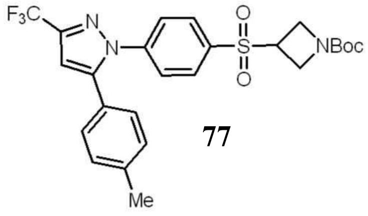

77

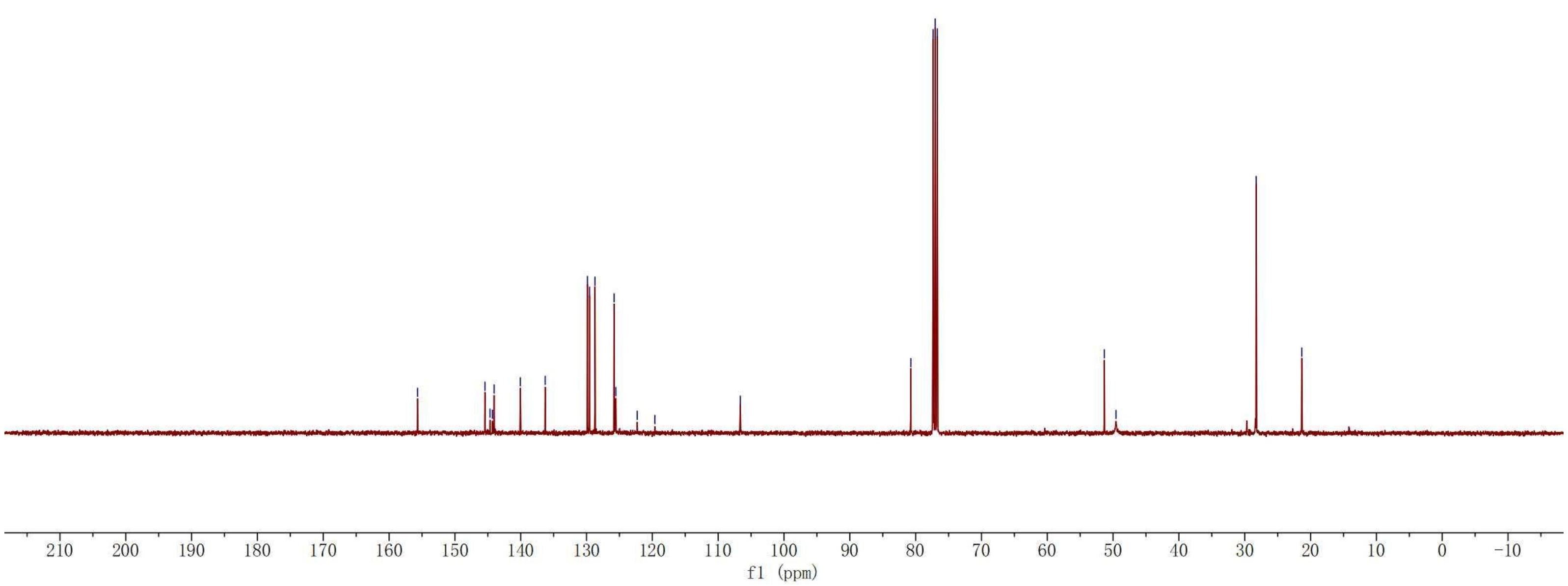

^{1}H NMR ($CDCl_3$, 400 MHz)

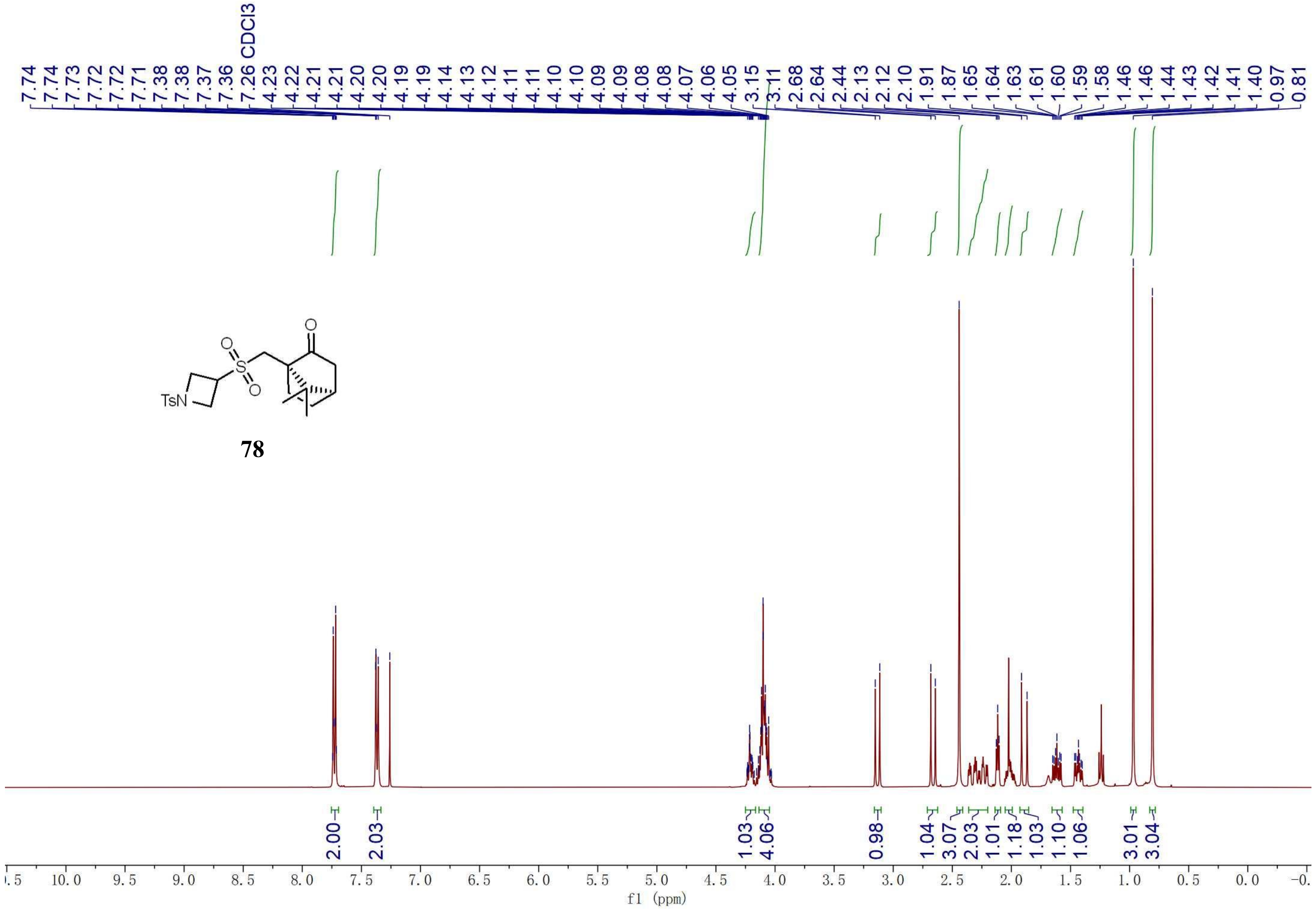

78

^{13}C NMR ($CDCl_3$, 101 MHz)

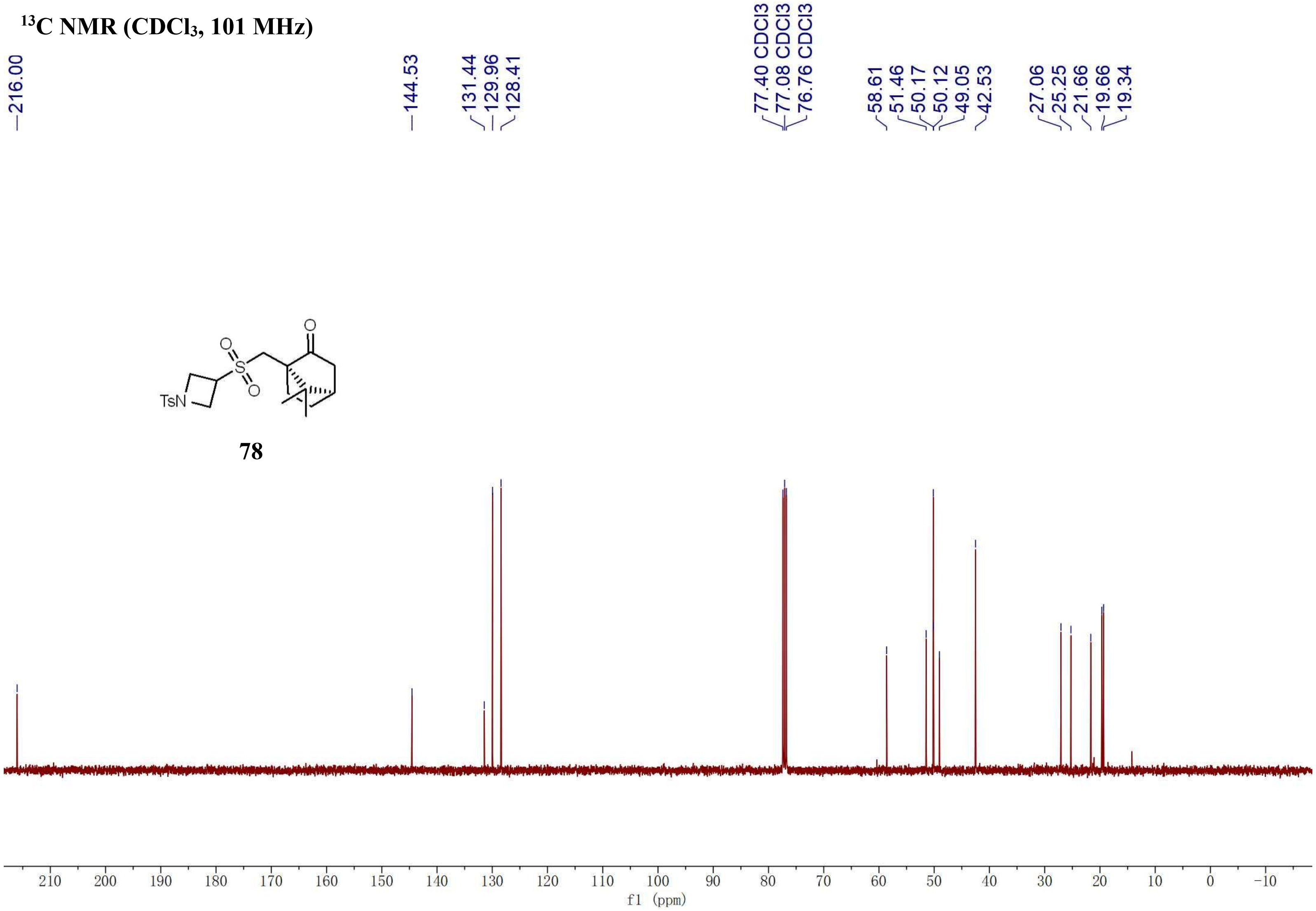

78

^{1}H NMR ($CDCl_3$, 400 MHz)

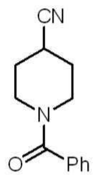

79

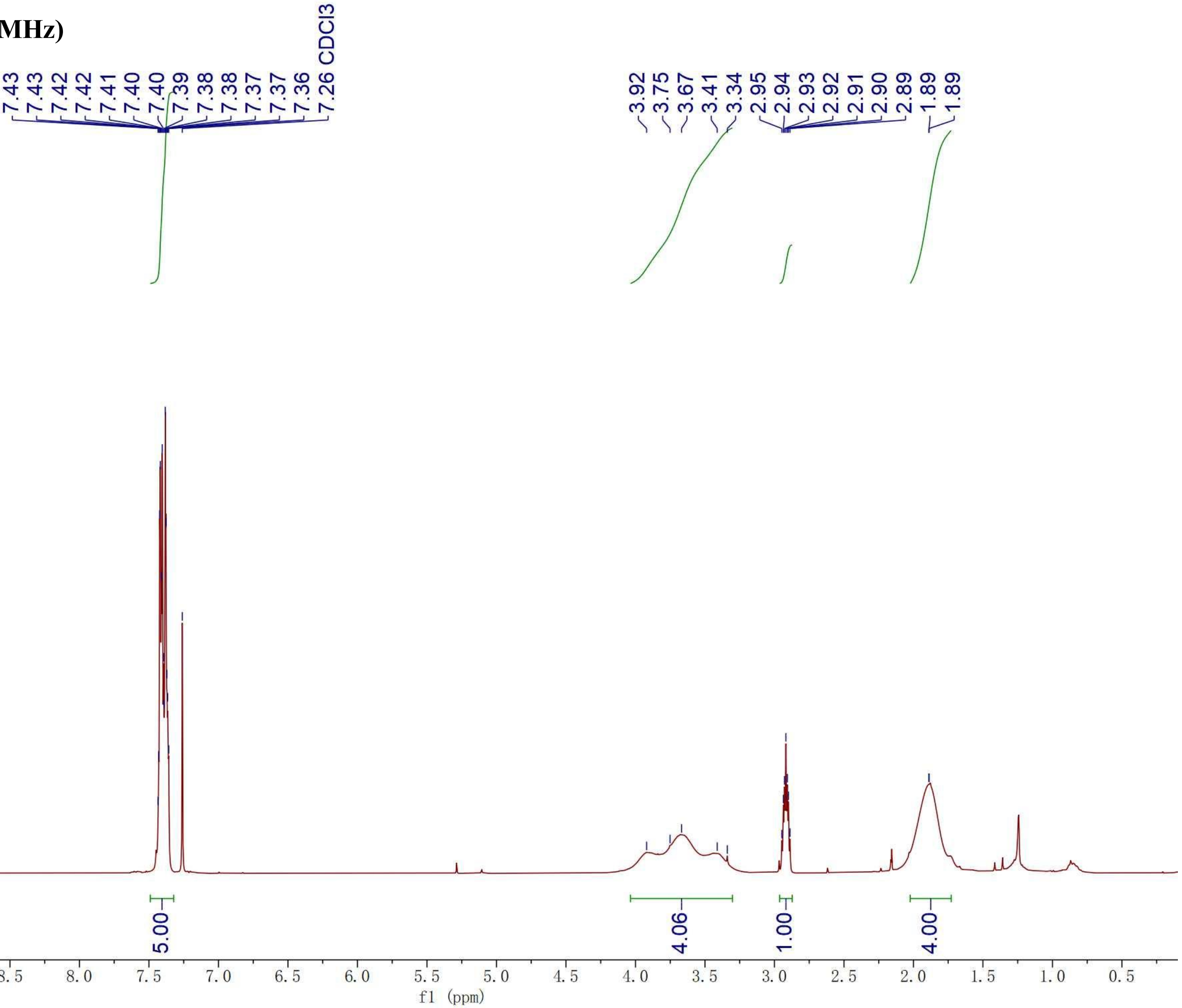

^{13}C NMR ($CDCl_3$, 101 MHz)

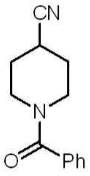

79

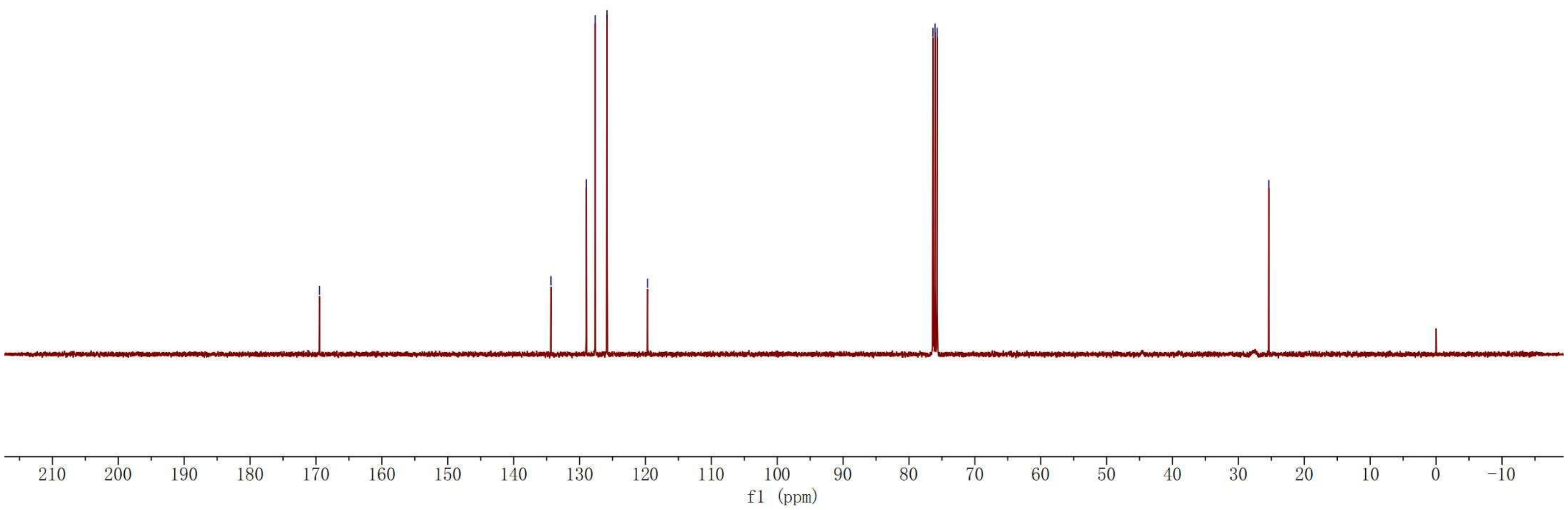

^{1}H NMR (CDCl$_3$, 400 MHz)

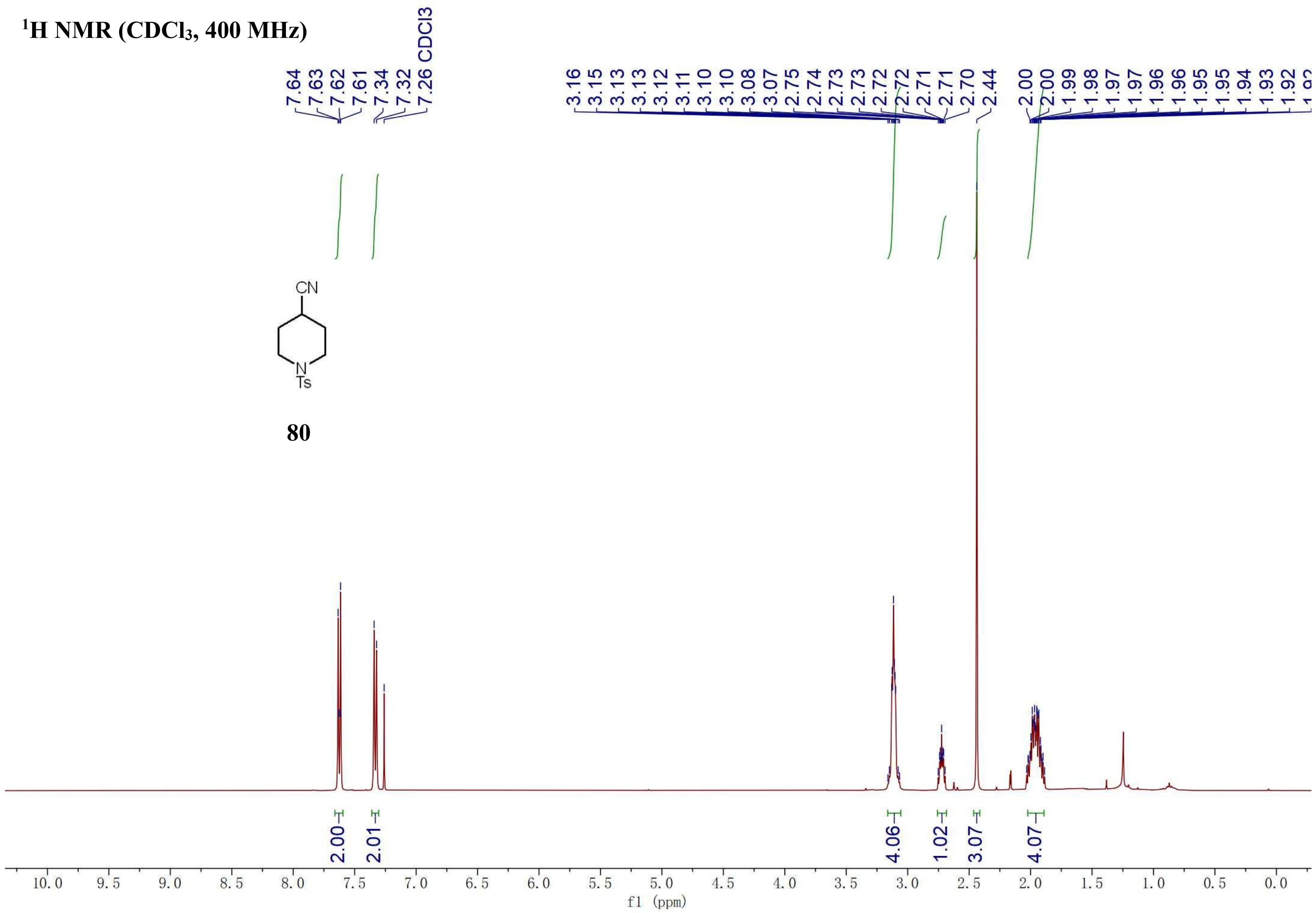

80

^{13}C NMR ($CDCl_3$, 101 MHz)

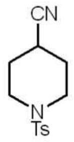

80

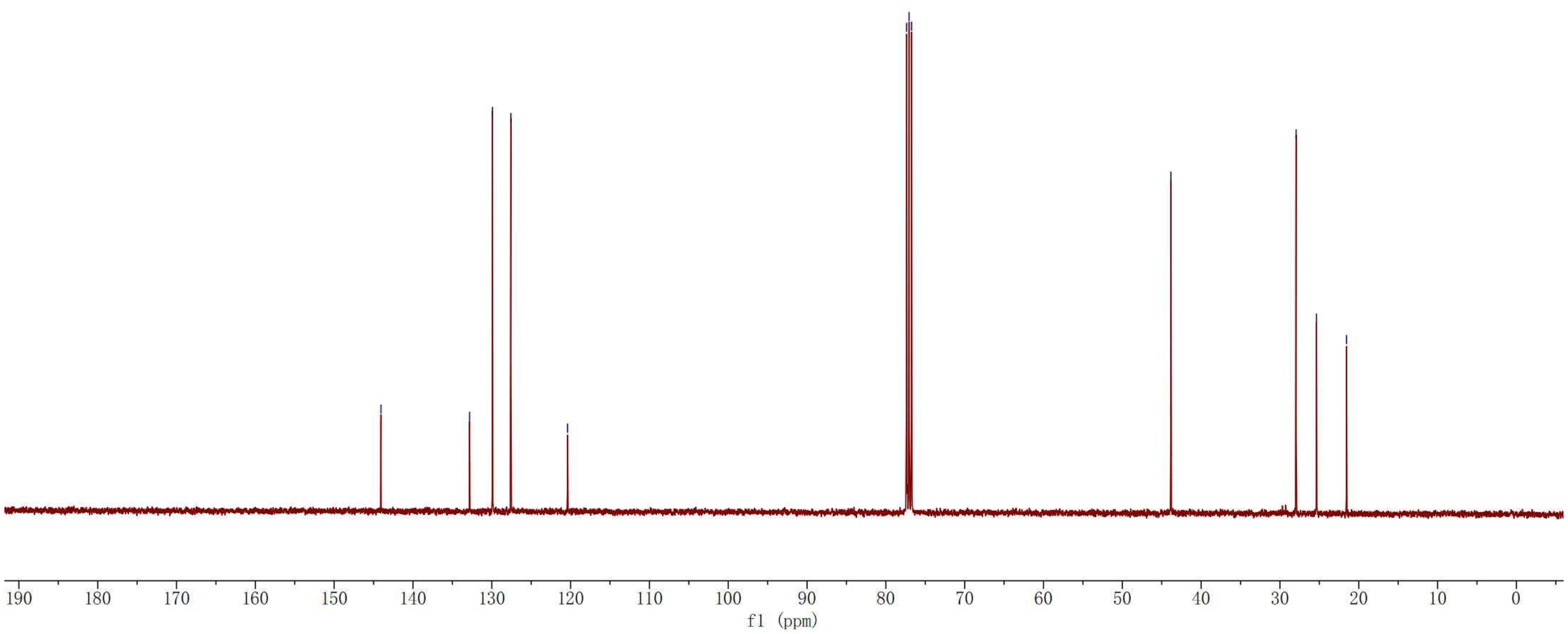

^{1}H NMR ($CDCl_3$, 400 MHz)

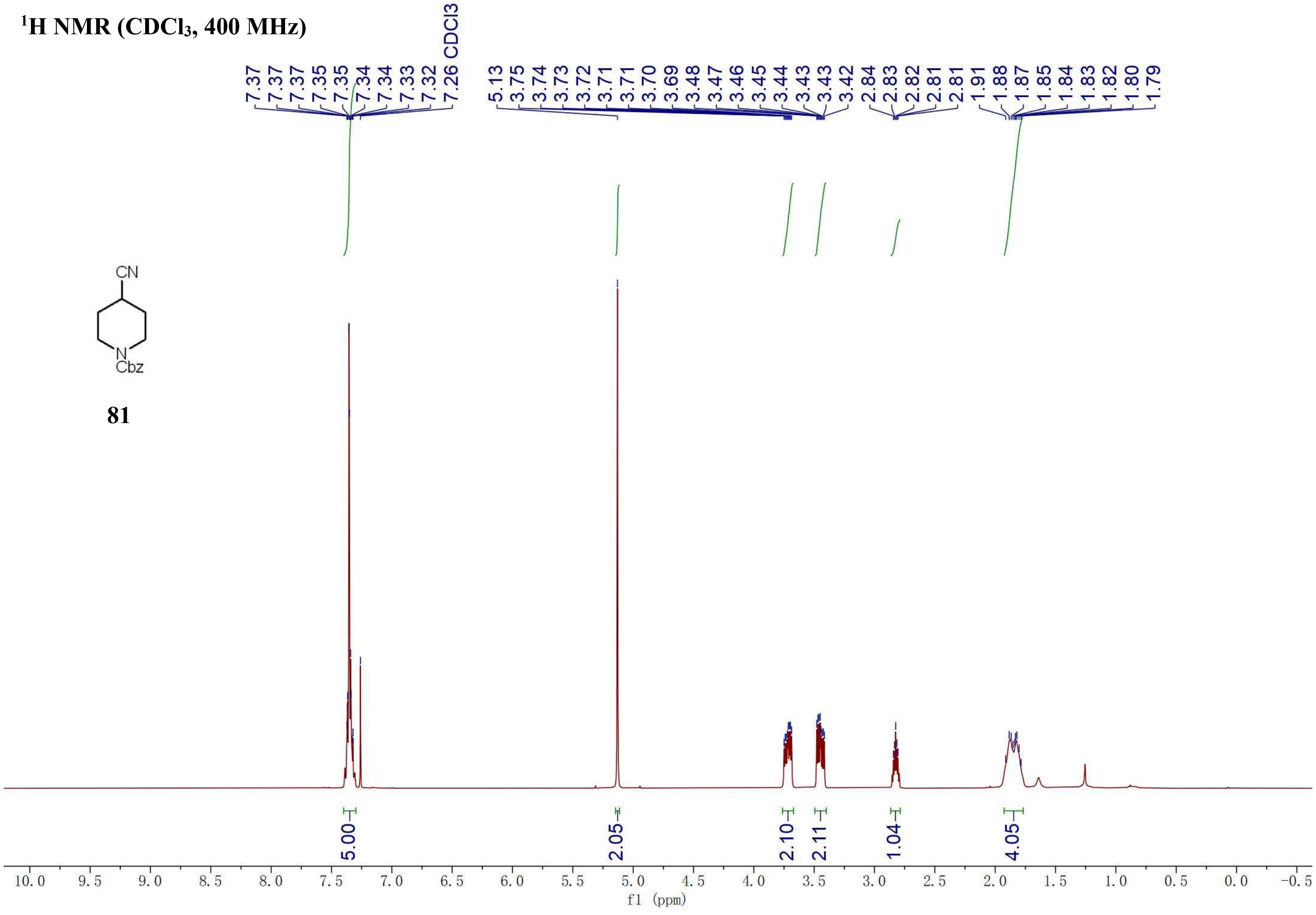

81

^{13}C NMR ($CDCl_3$, 101 MHz)

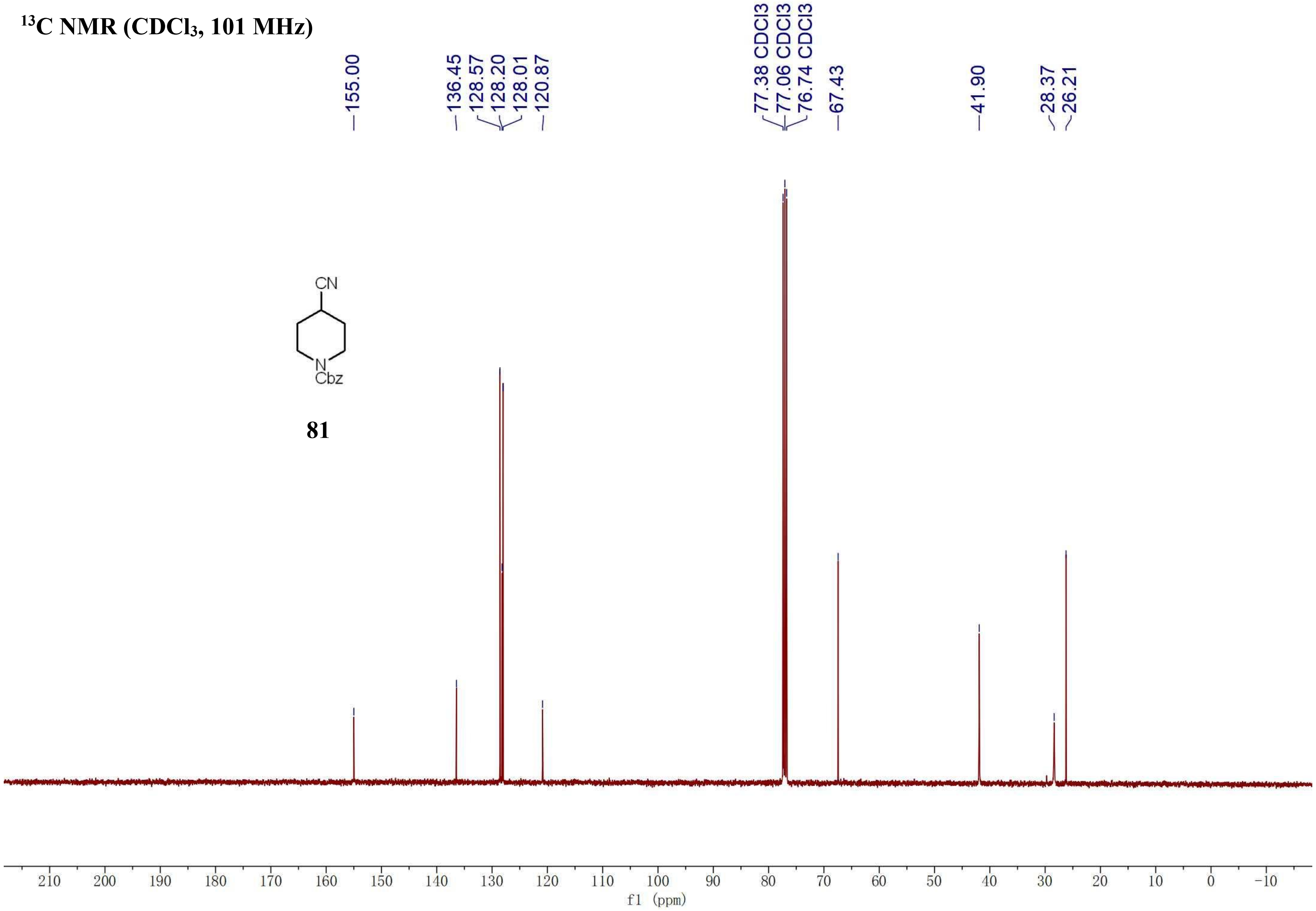

81

^{1}H NMR ($CDCl_3$, 400 MHz)

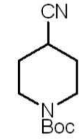

82

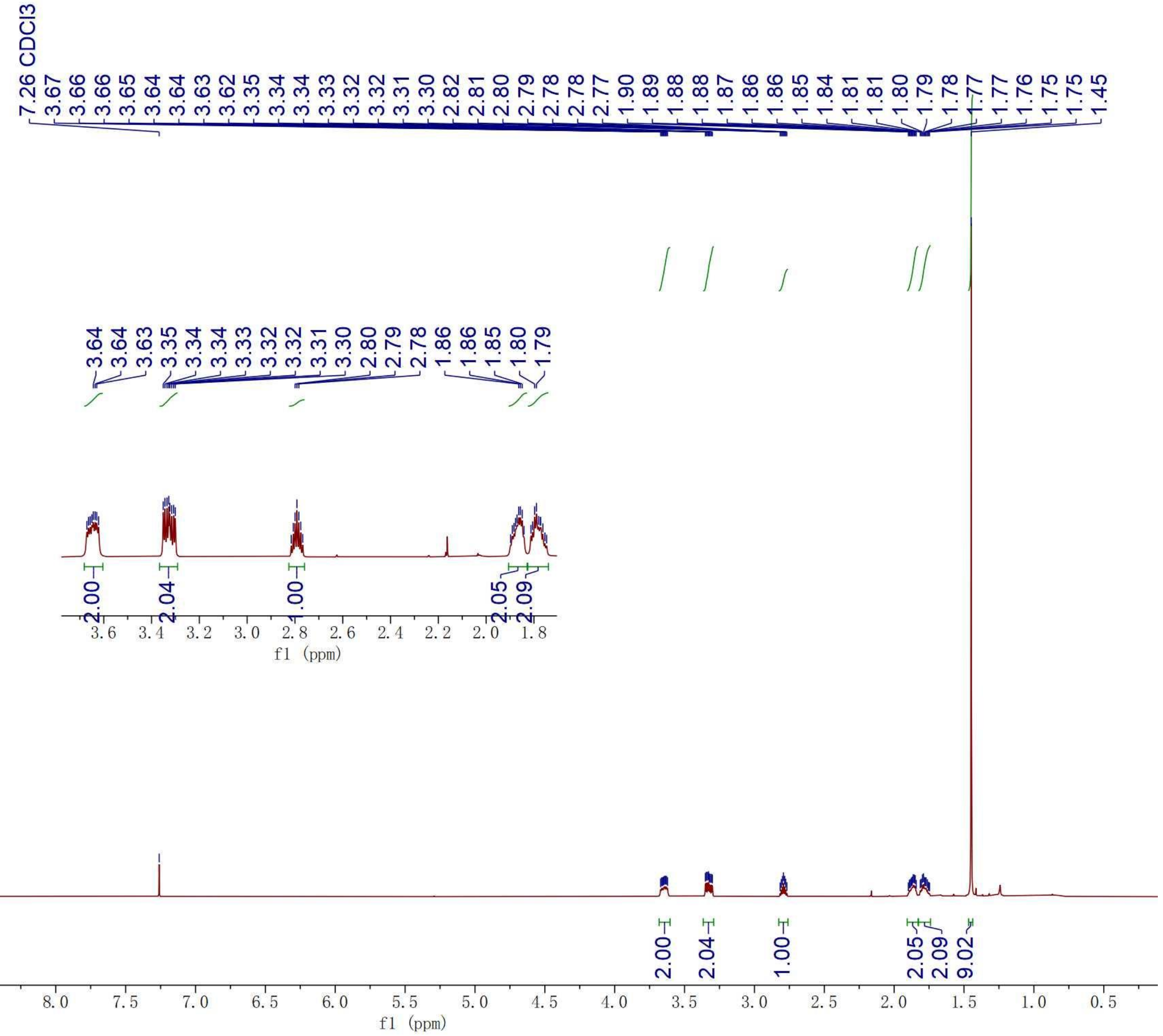

^{13}C NMR ($CDCl_3$, 101 MHz)

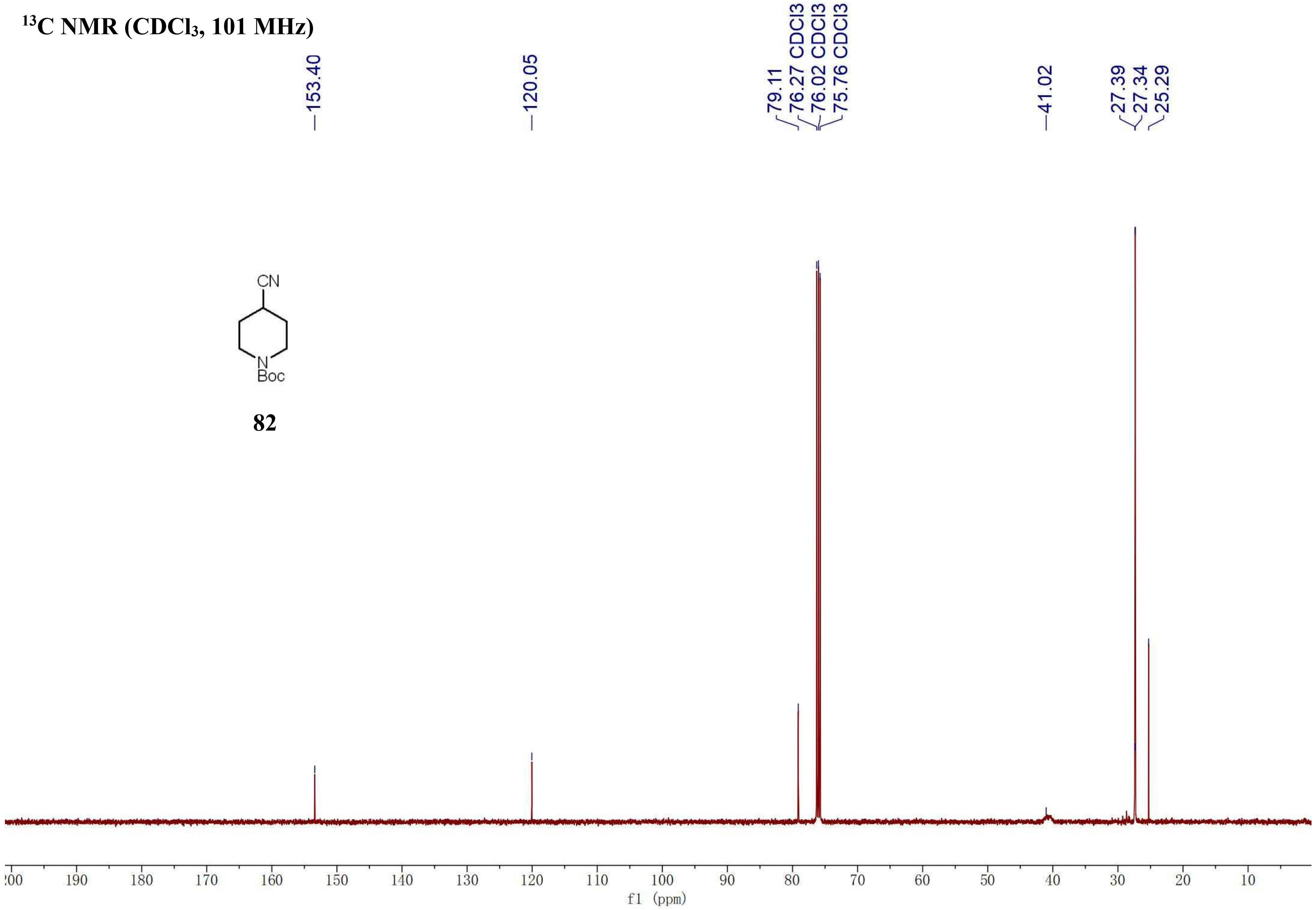

^{1}H NMR ($CDCl_3$, 400 MHz)

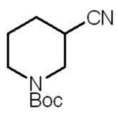

83

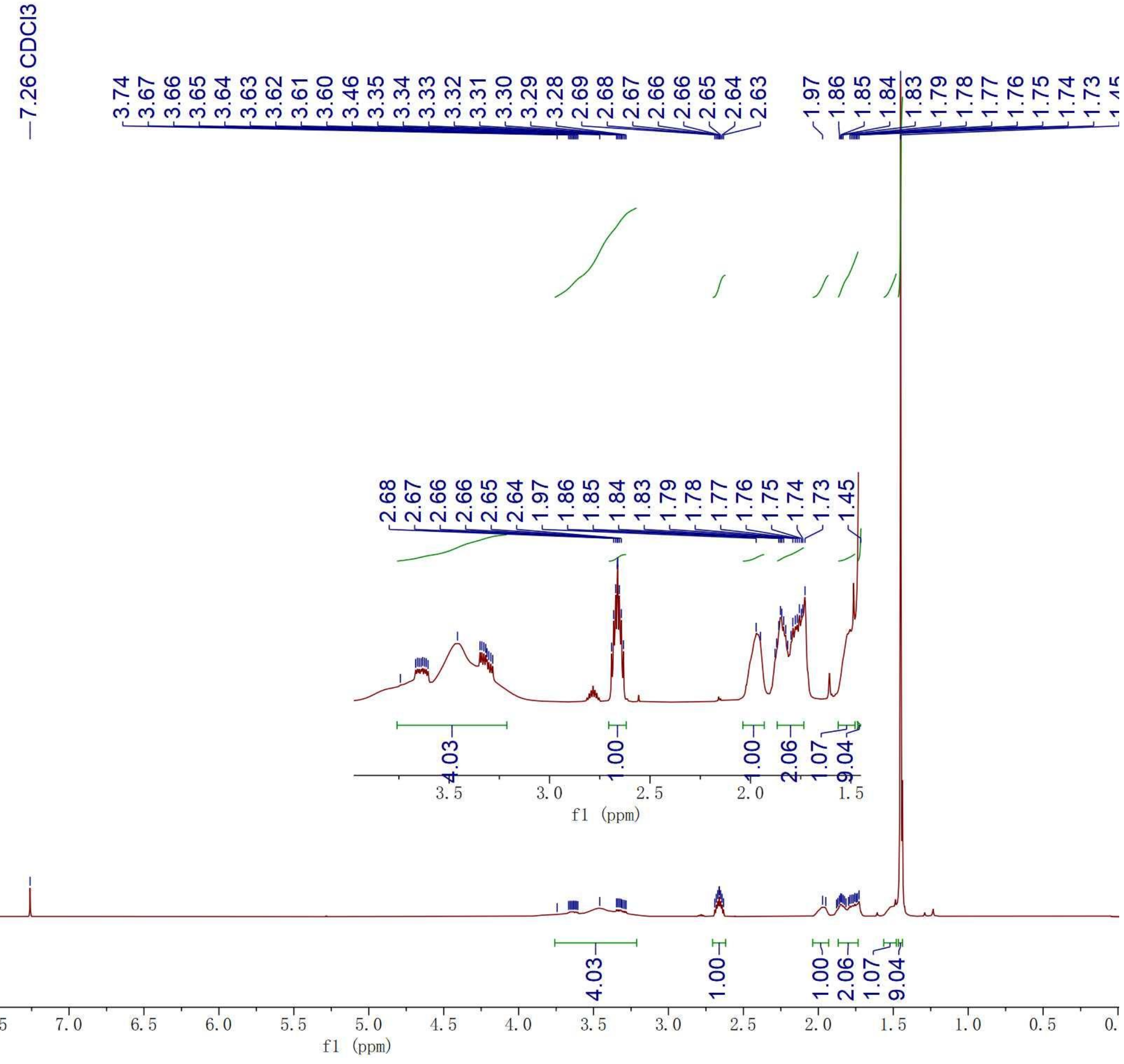

^{13}C NMR (CDCl$_3$, 101 MHz)

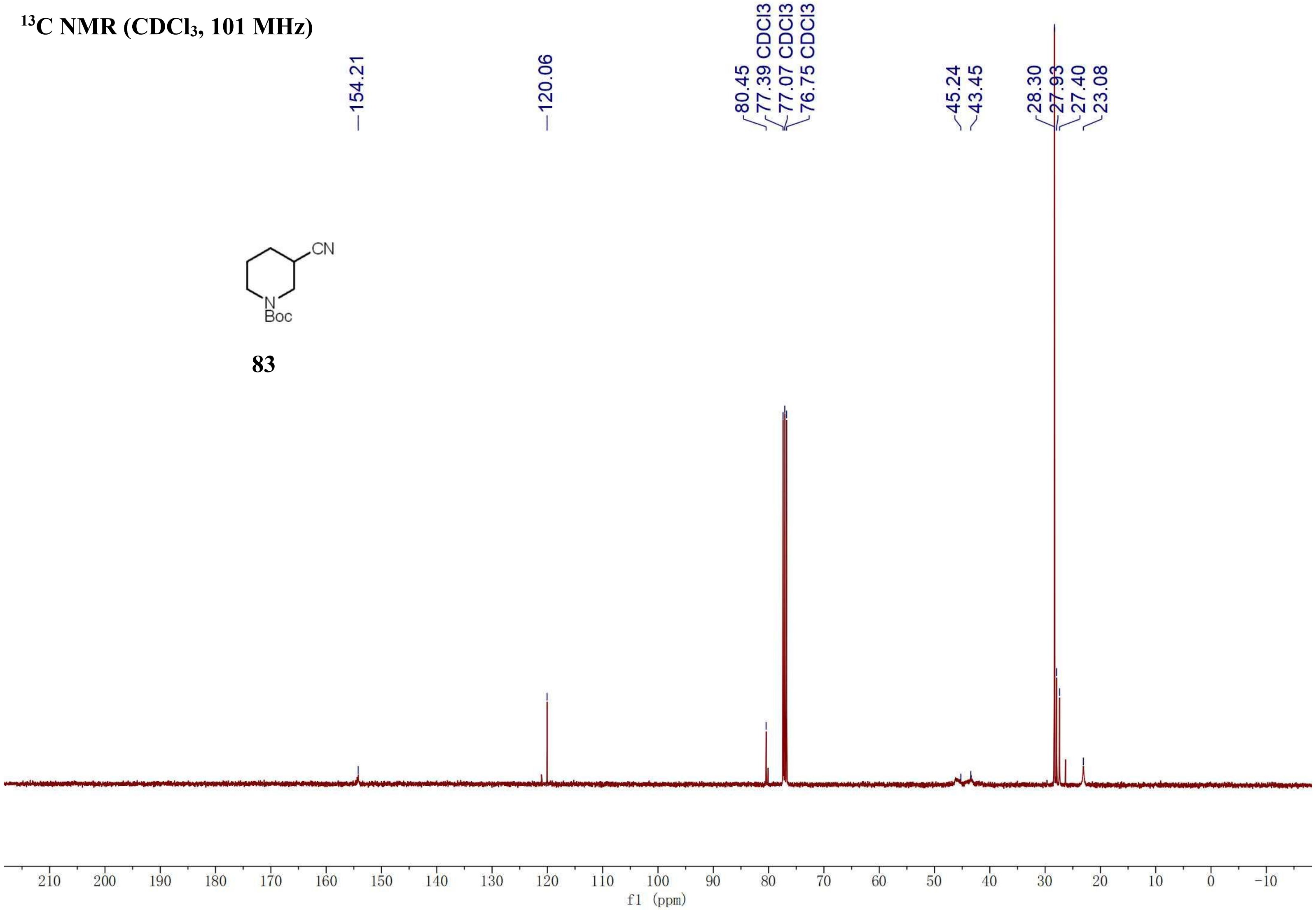

^1H NMR ($CDCl_3$, 400 MHz)

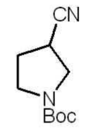

84

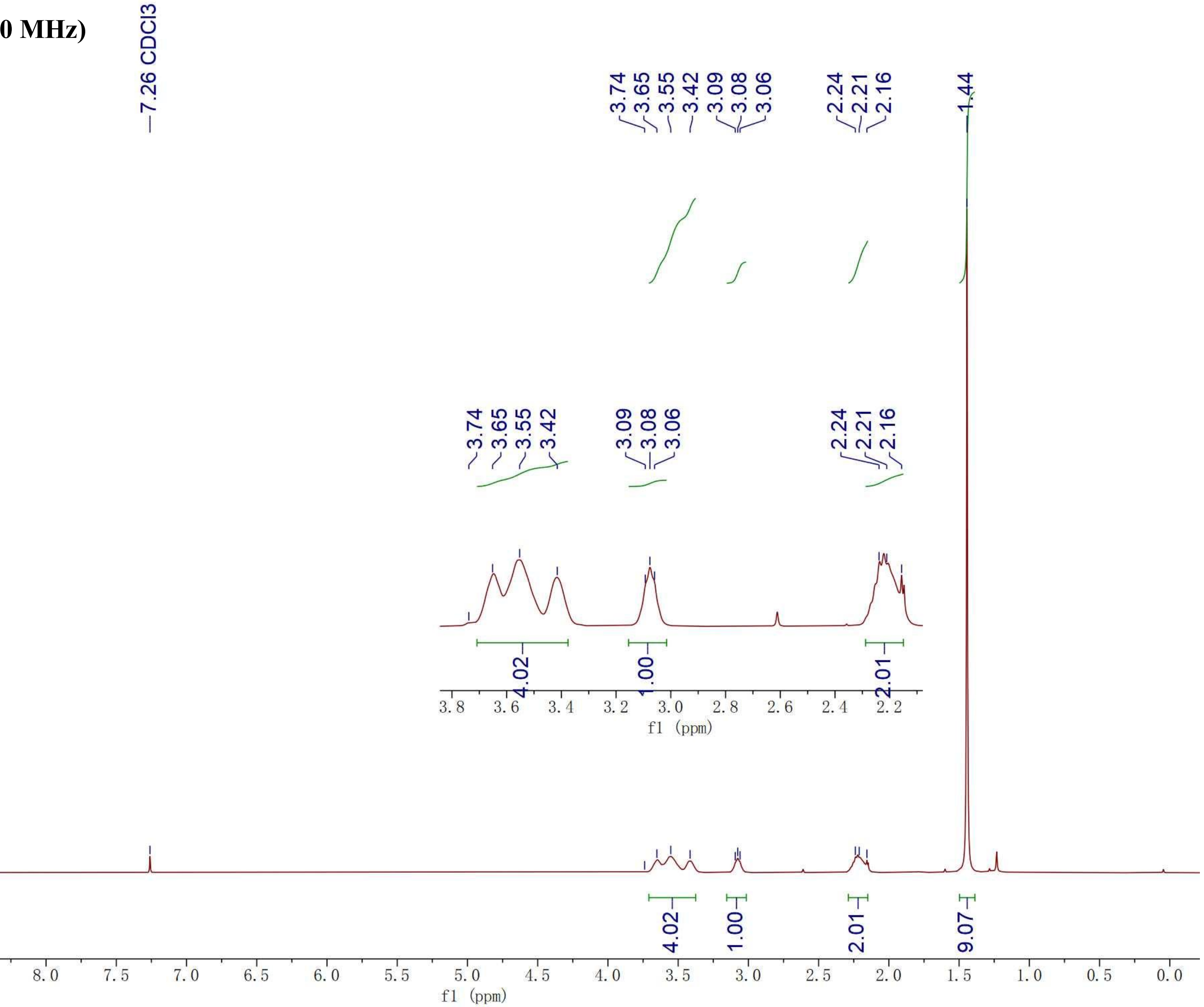

^{13}C NMR ($CDCl_3$, 101 MHz)

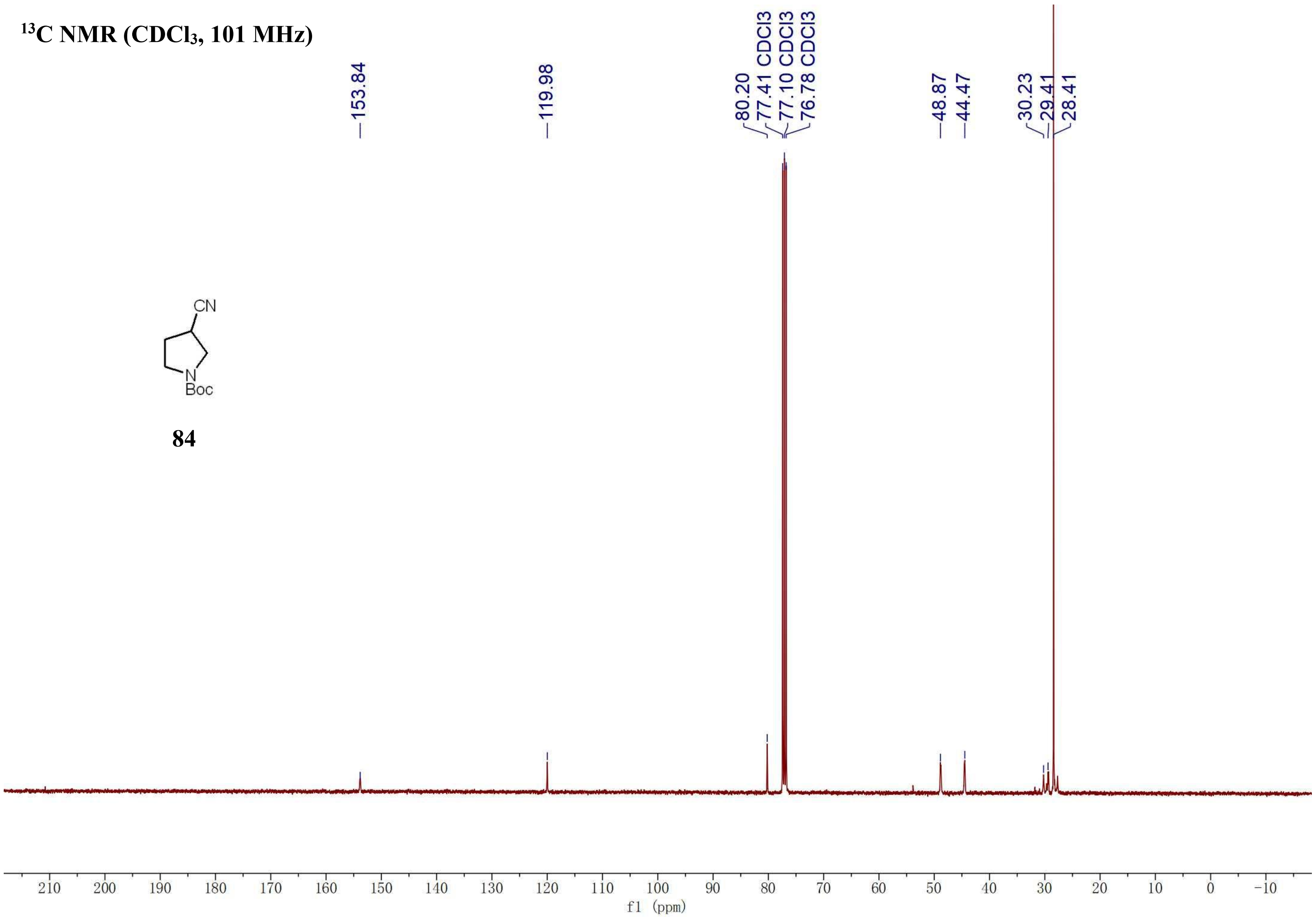

^{1}H NMR (CDCl$_3$, 400 MHz)

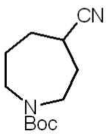

85

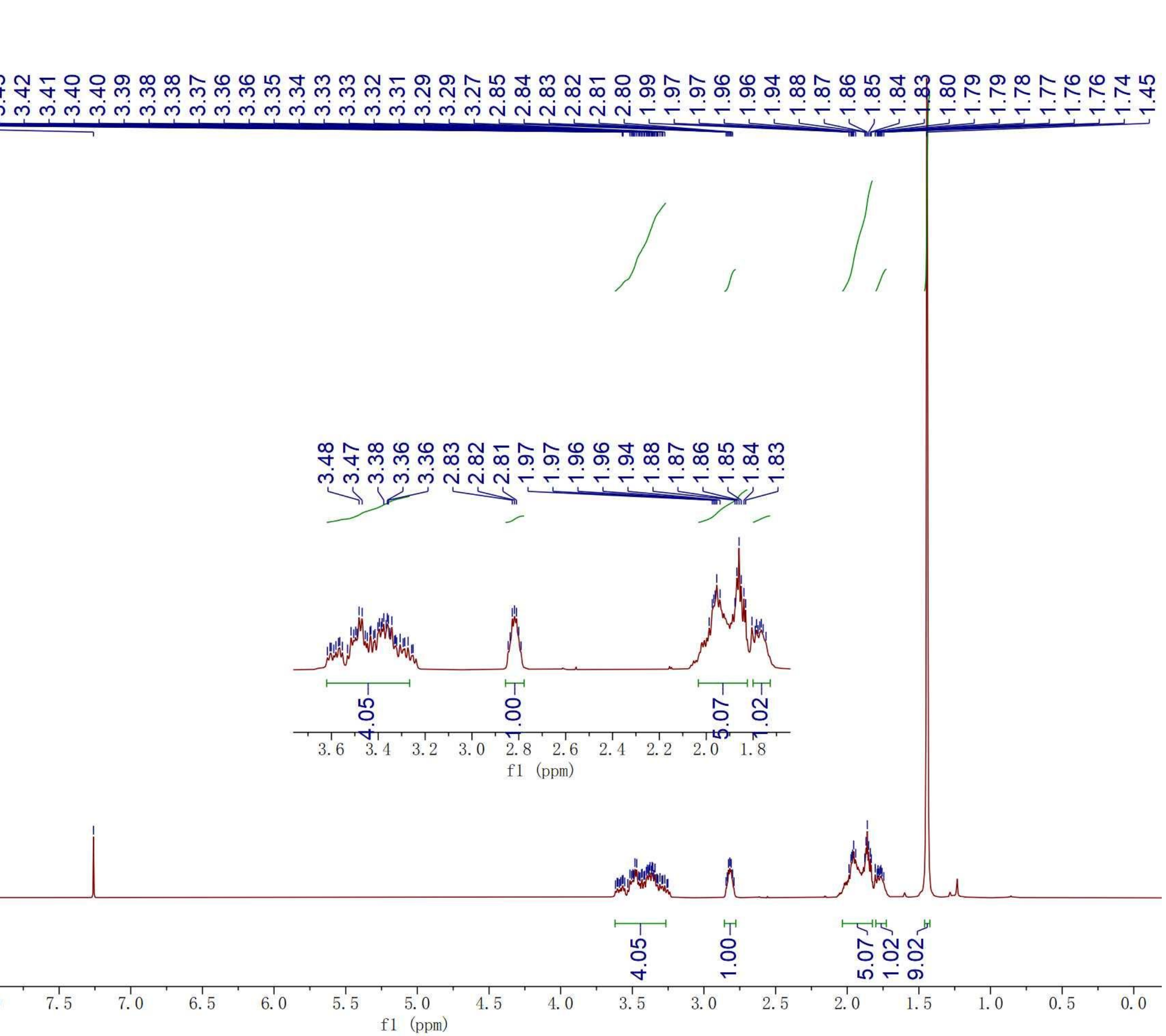

^{13}C NMR ($CDCl_3$, 101 MHz)

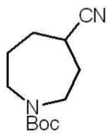

85

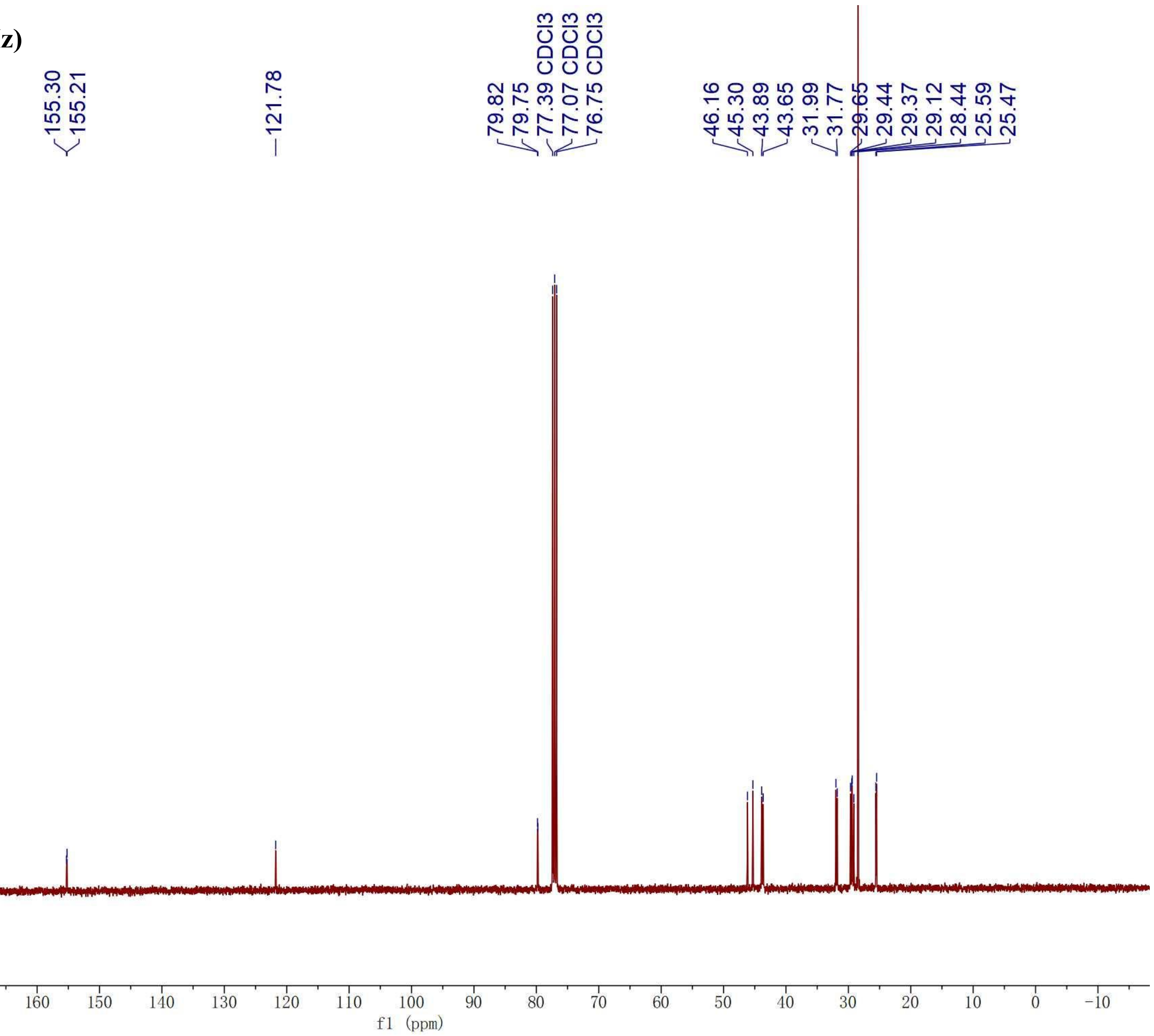

^{1}H NMR ($CDCl_3$, 400 MHz)

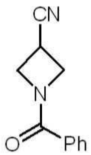

86

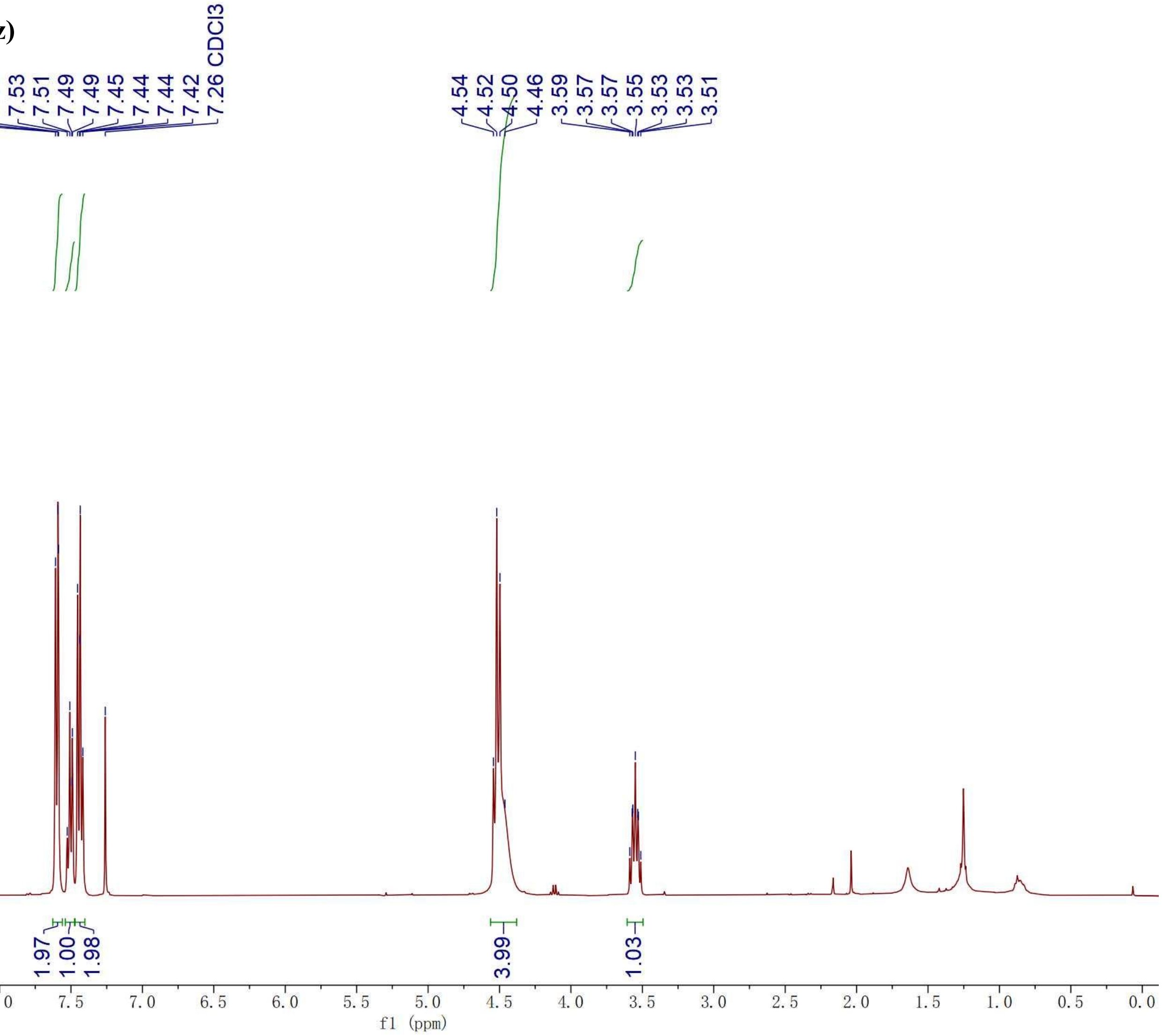

^{13}C NMR ($CDCl_3$, 101 MHz)

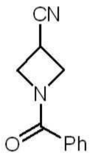

86

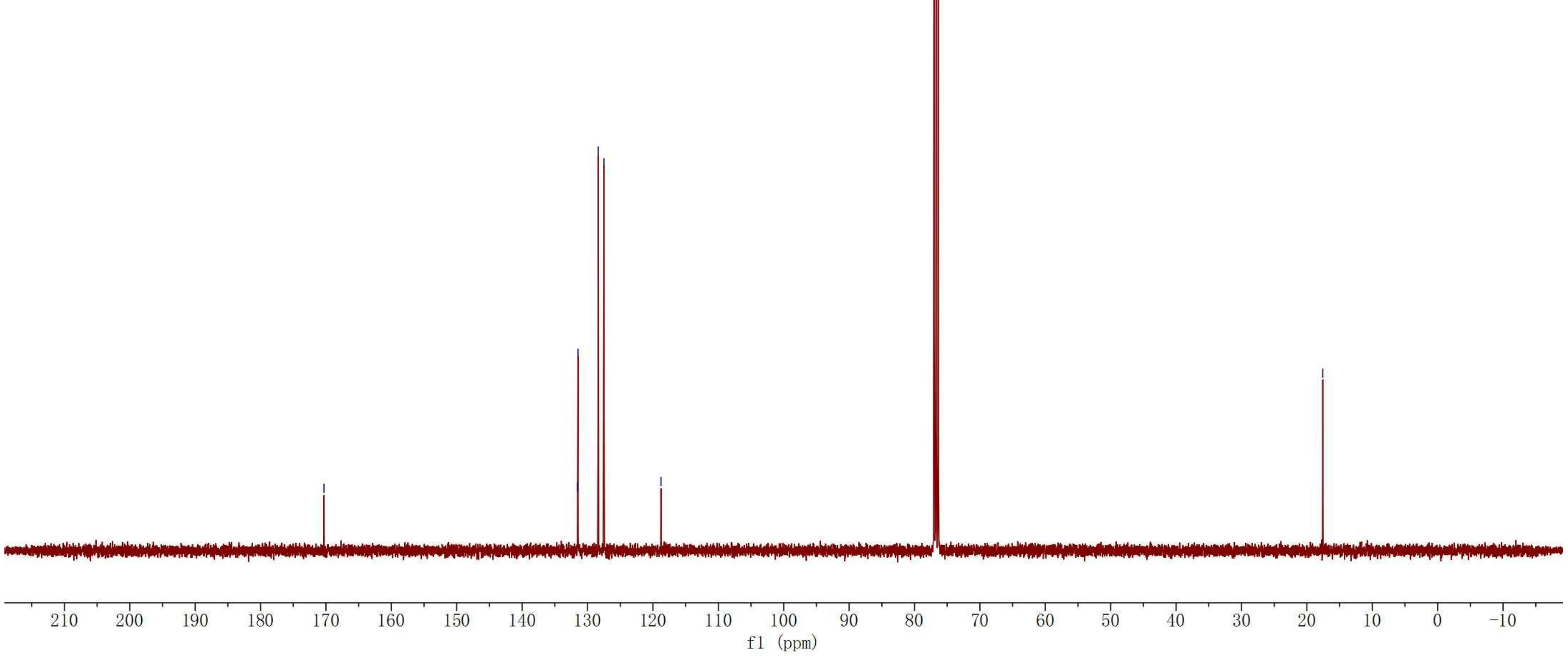

^{1}H NMR ($CDCl_3$, 400 MHz)

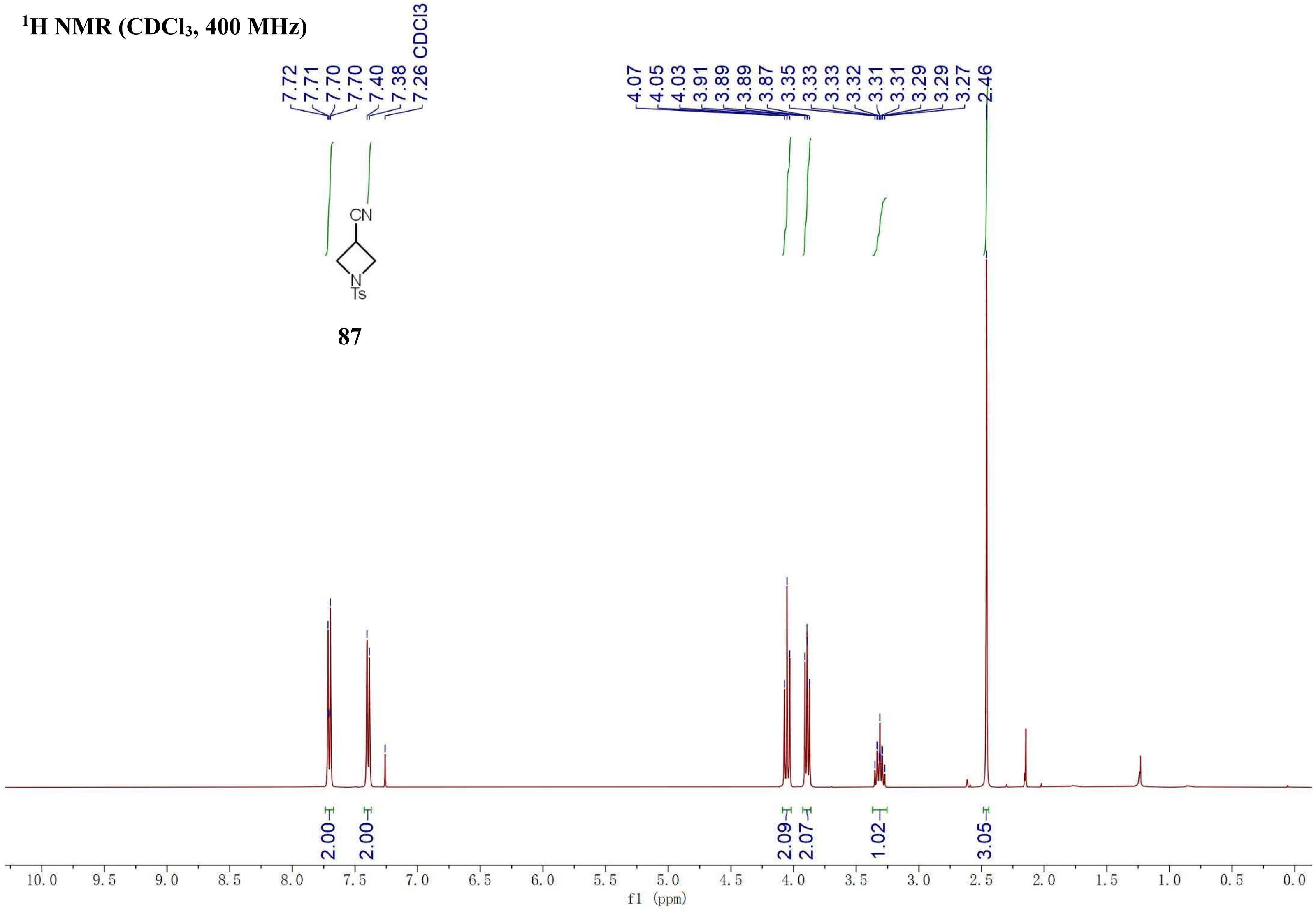

87

^{13}C NMR ($CDCl_3$, 101 MHz)

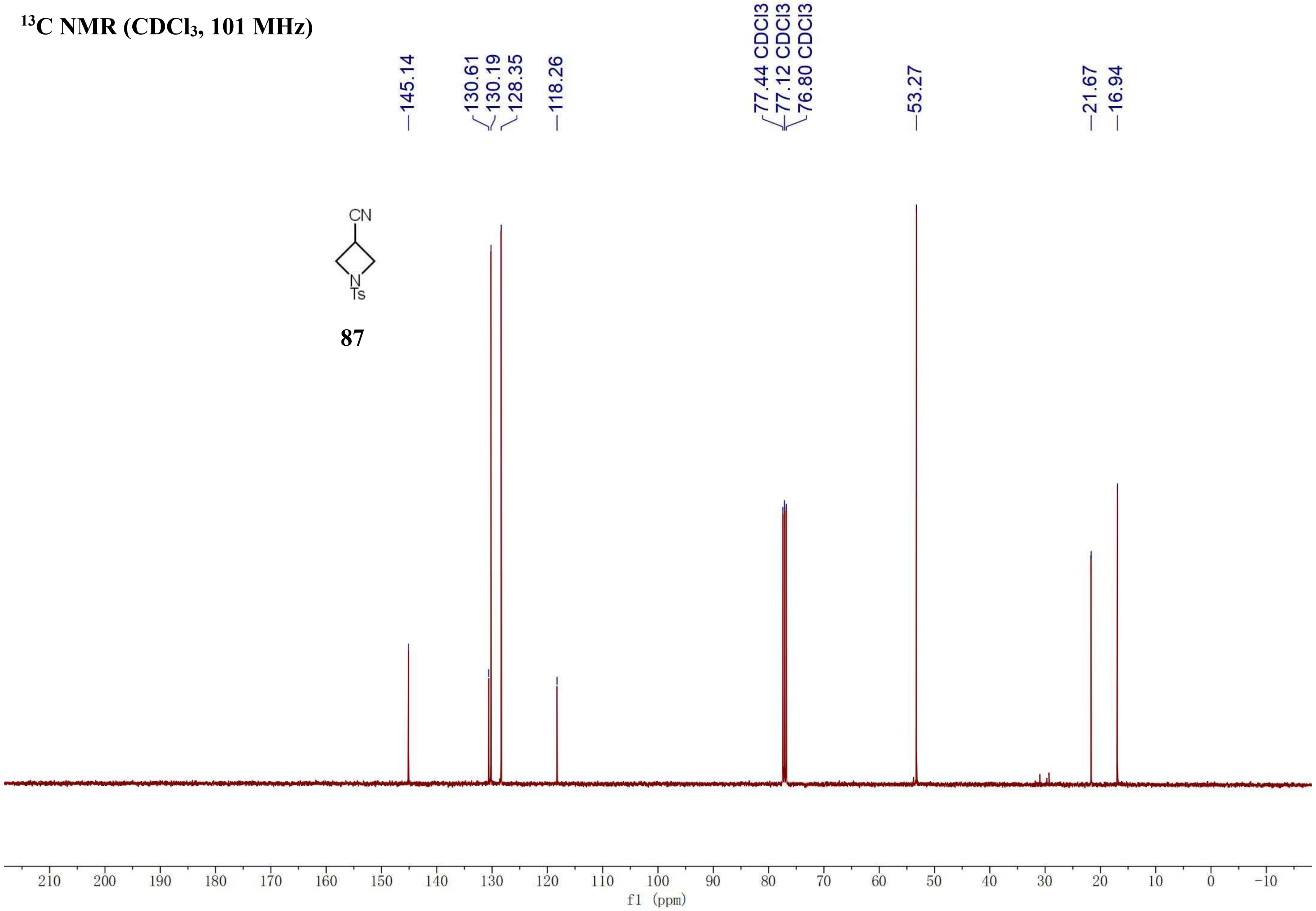

^{1}H NMR ($CDCl_3$, 400 MHz)

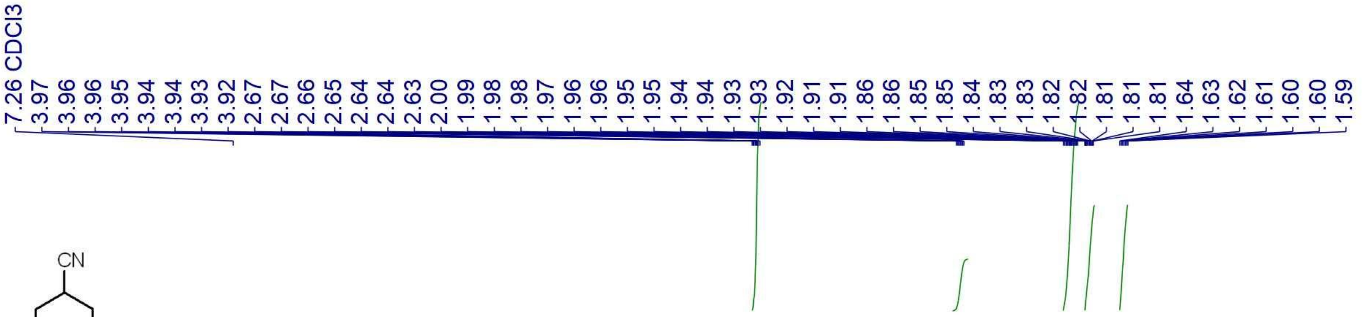

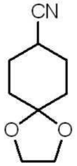

88

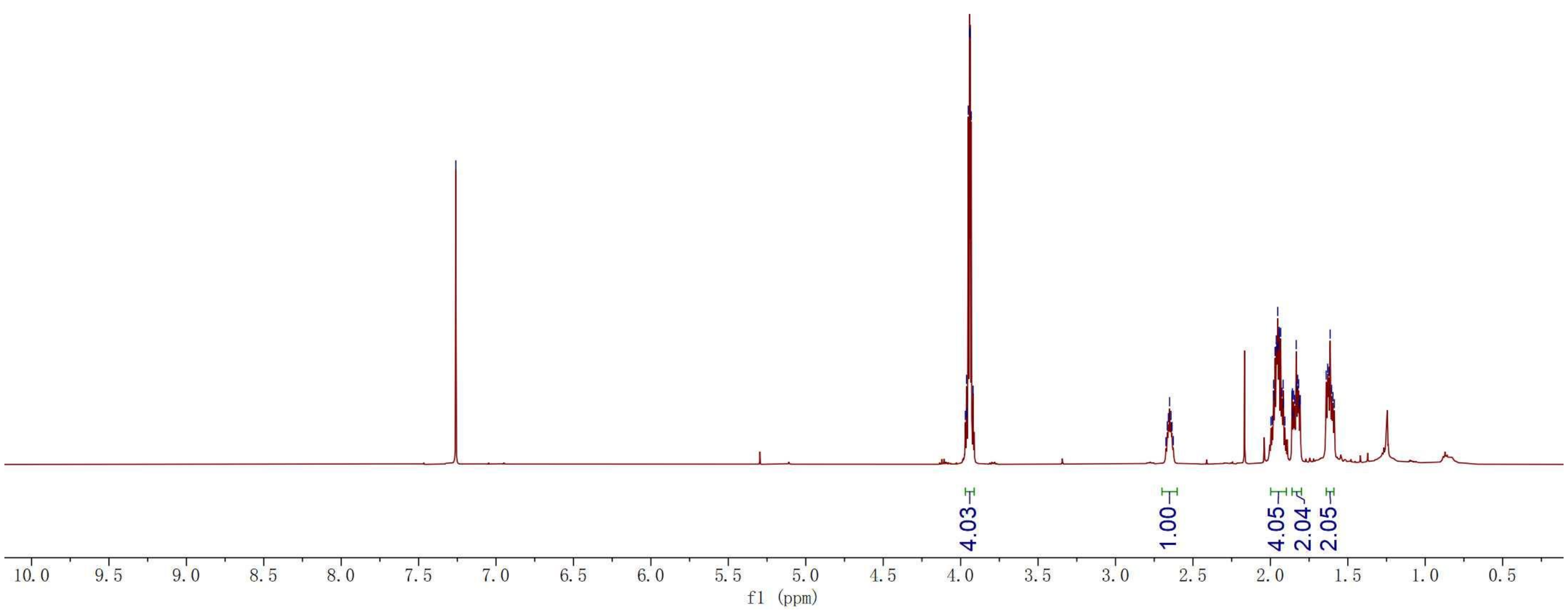

^{13}C NMR ($CDCl_3$, 101 MHz)

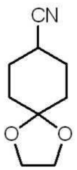

88

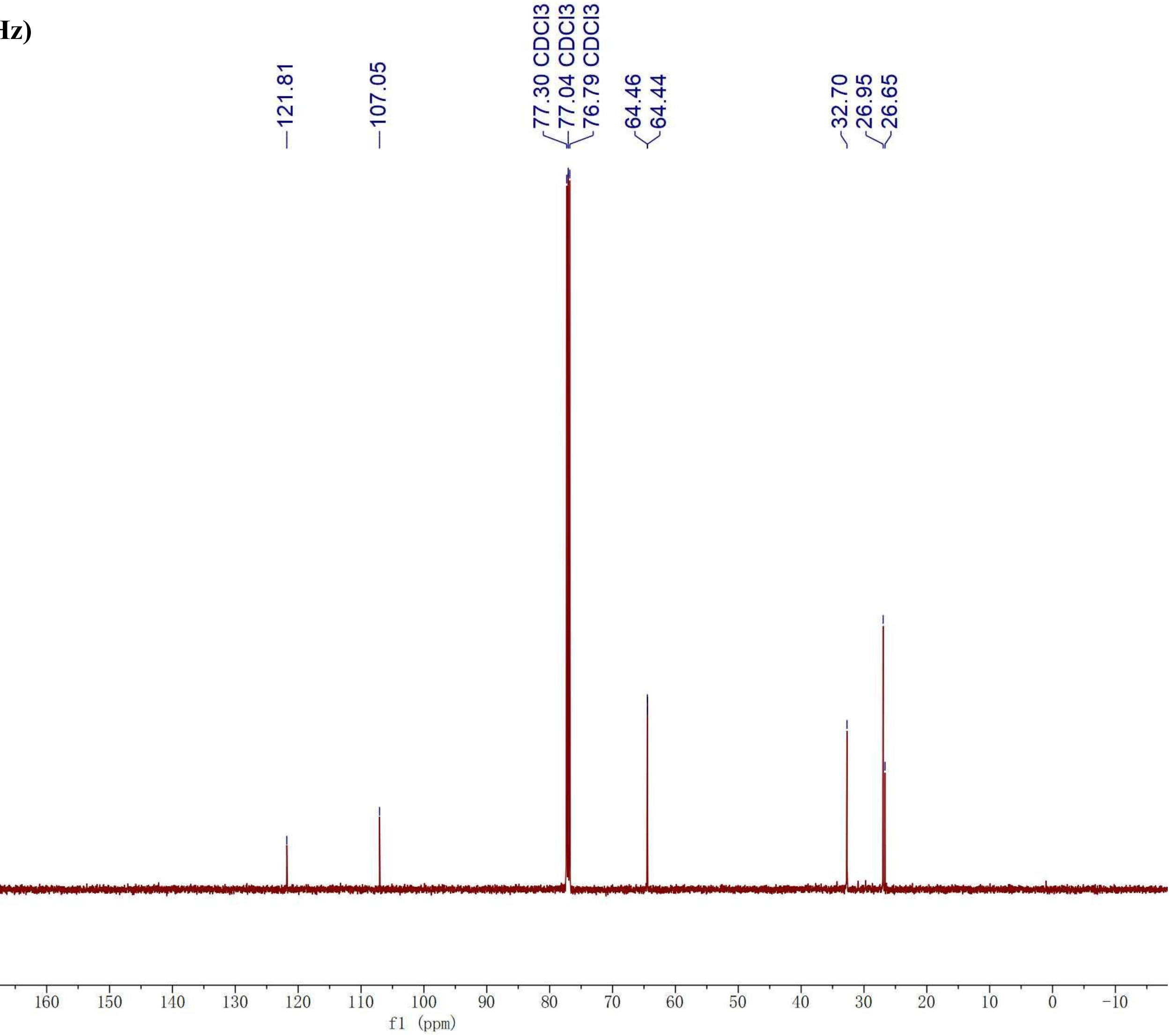

^{1}H NMR ($CDCl_3$, 400 MHz)

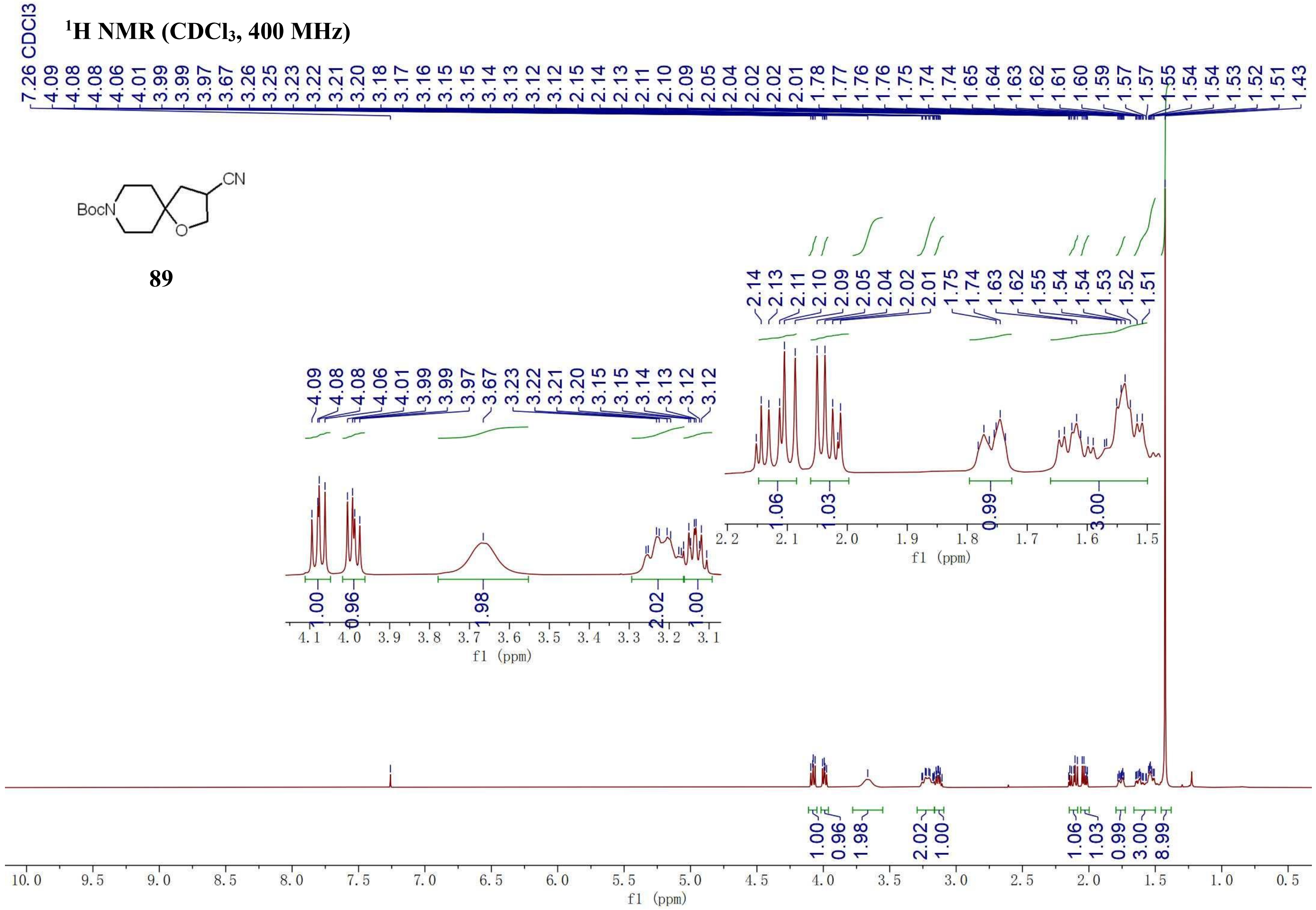

^{13}C NMR ($CDCl_3$, 101 MHz)

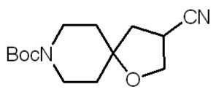

89

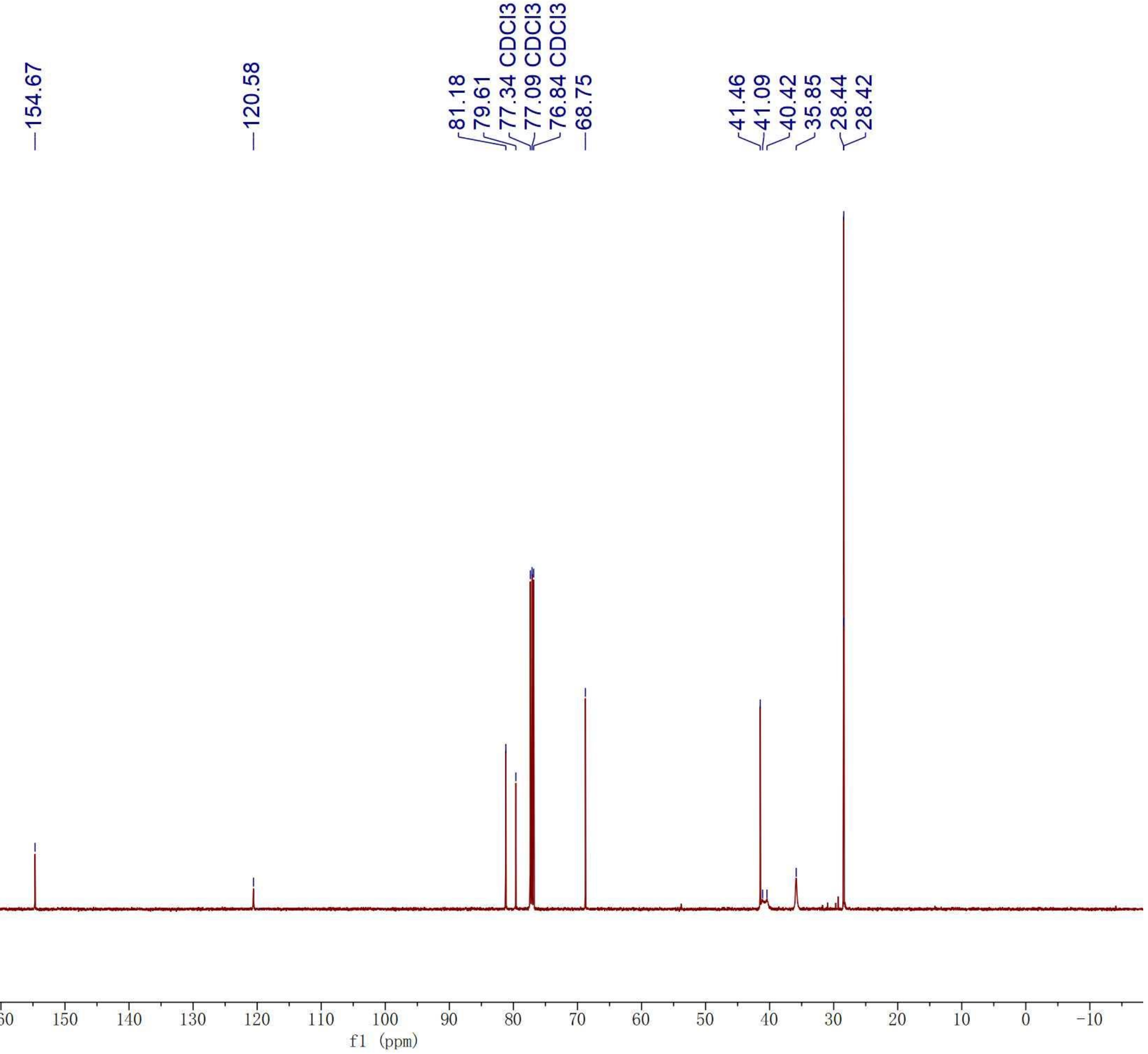

^{1}H NMR ($CDCl_3$, 400 MHz)

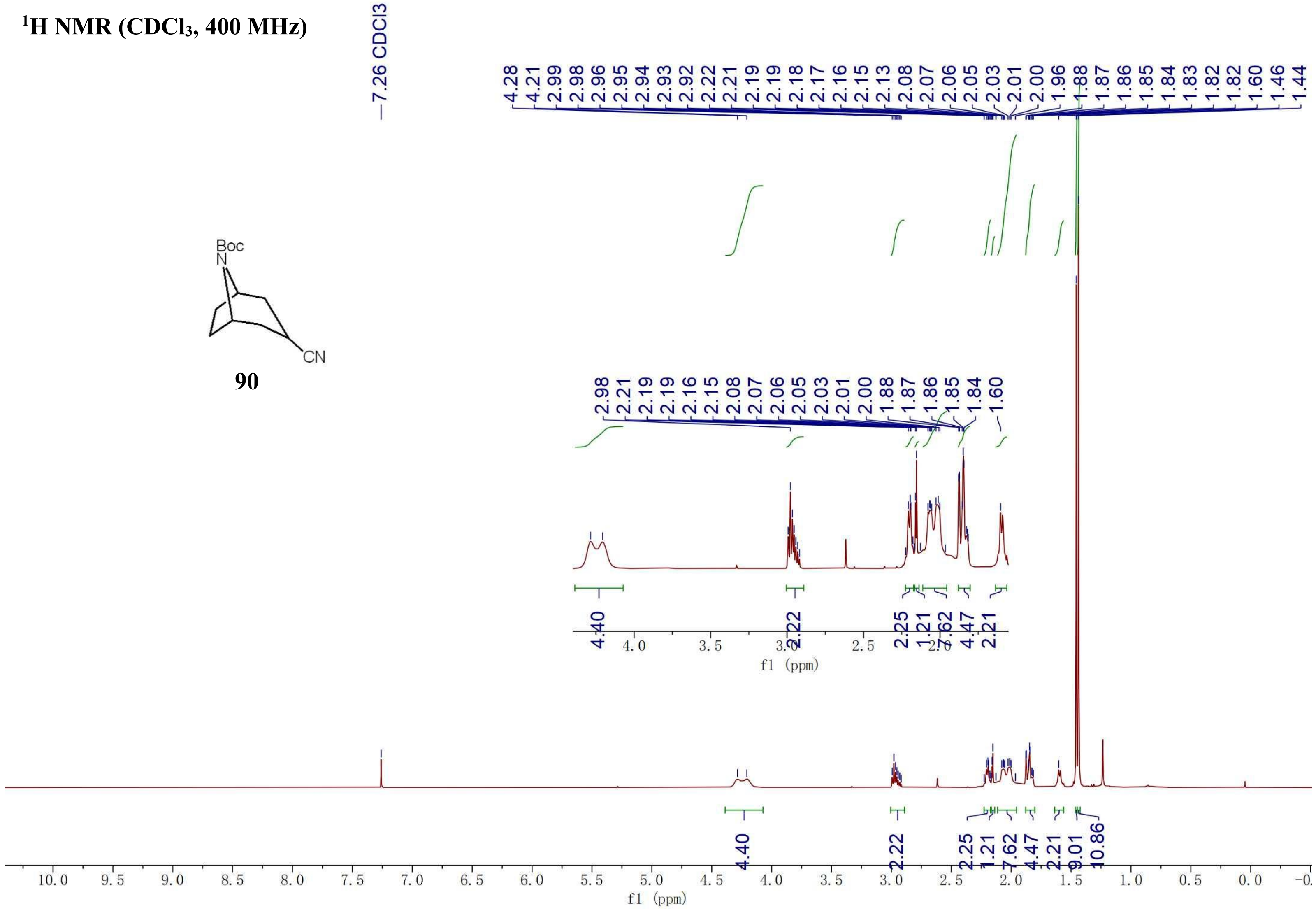

^{13}C NMR (CDCl$_3$, 101 MHz)

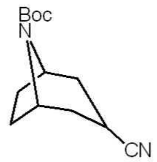

90

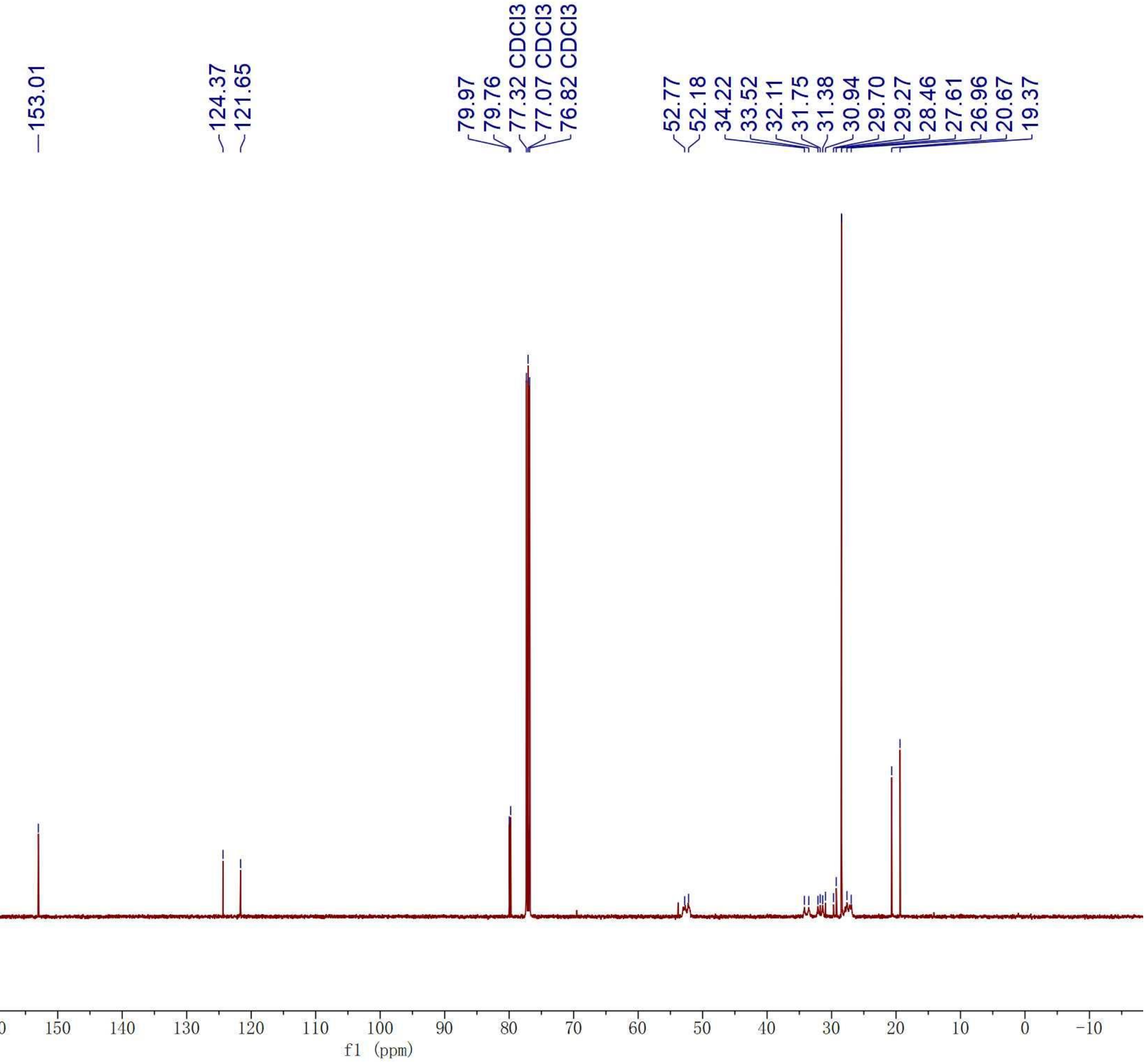

^{1}H NMR (CDCl$_3$, 400 MHz)

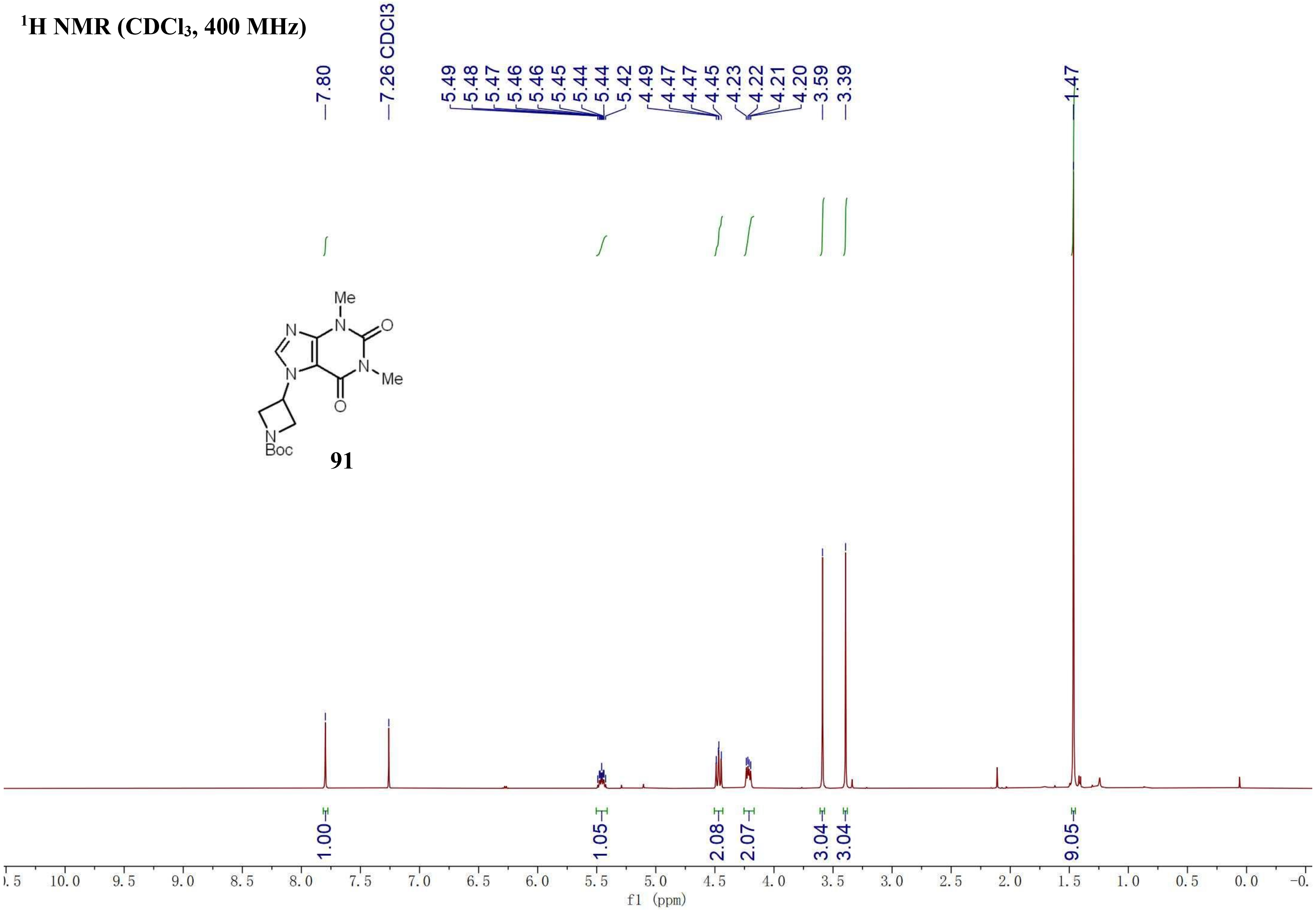

91

^{13}C NMR ($CDCl_3$, 101 MHz)

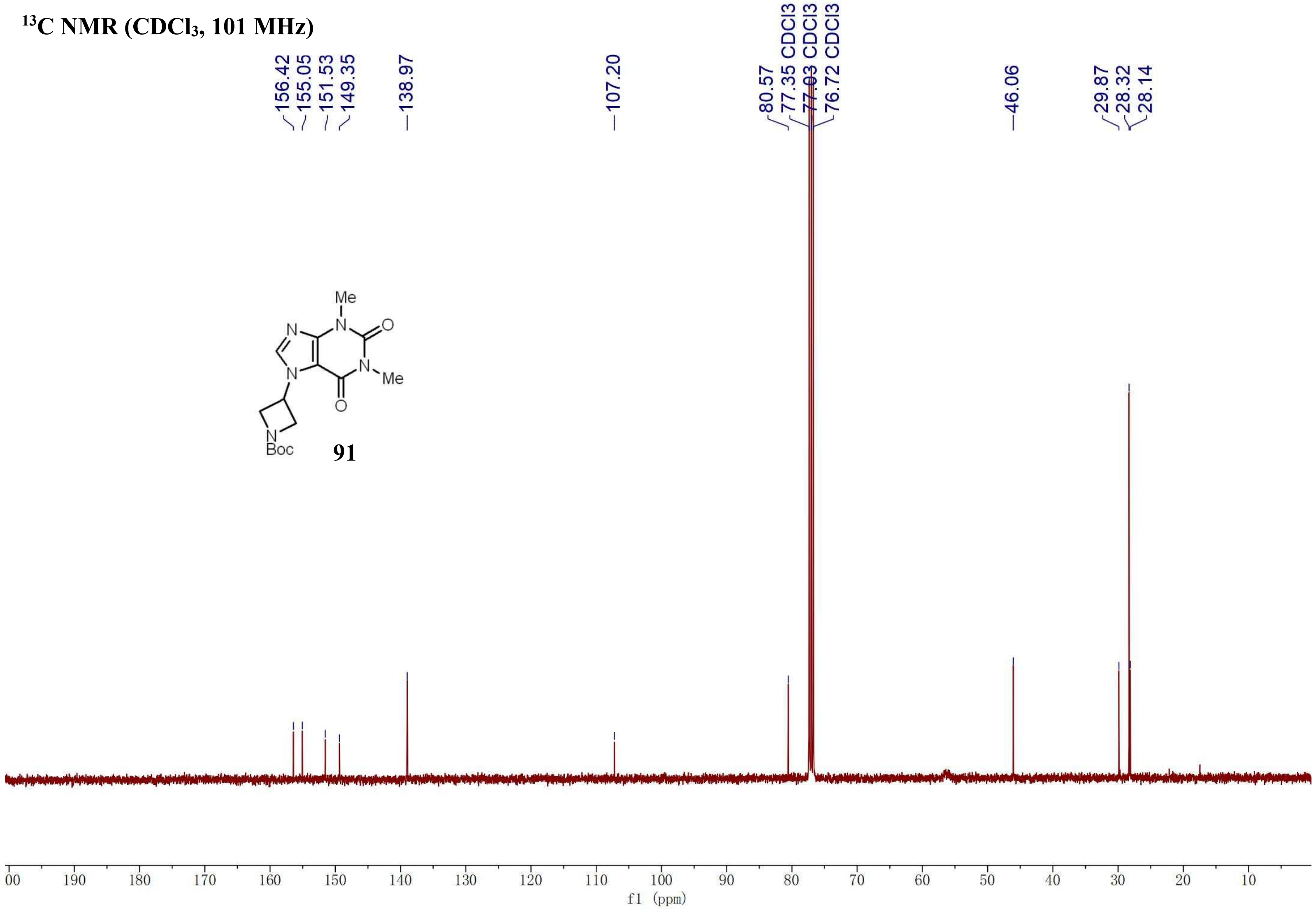

91

^{1}H NMR (CDCl$_3$, 400 MHz)

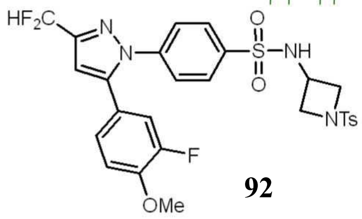

92

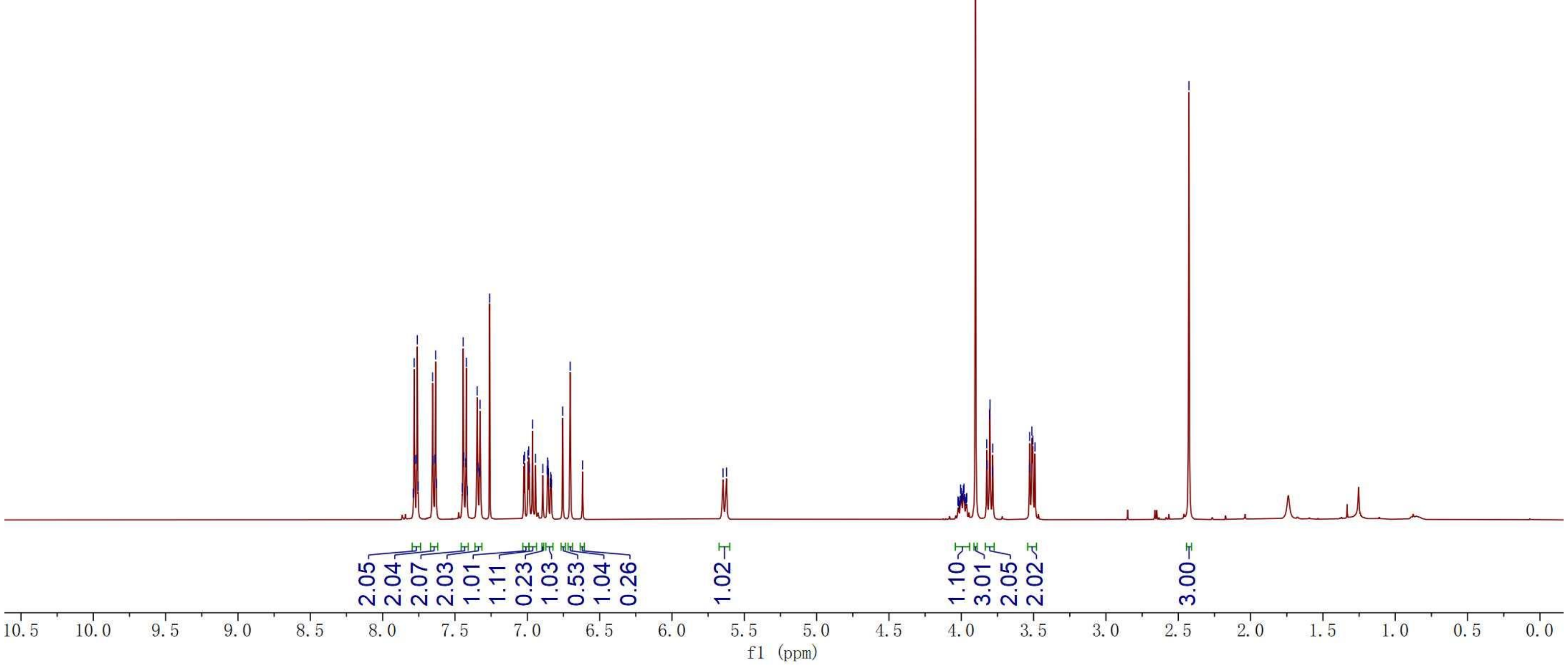

^{19}F NMR ($CDCl_3$, 376 MHz)

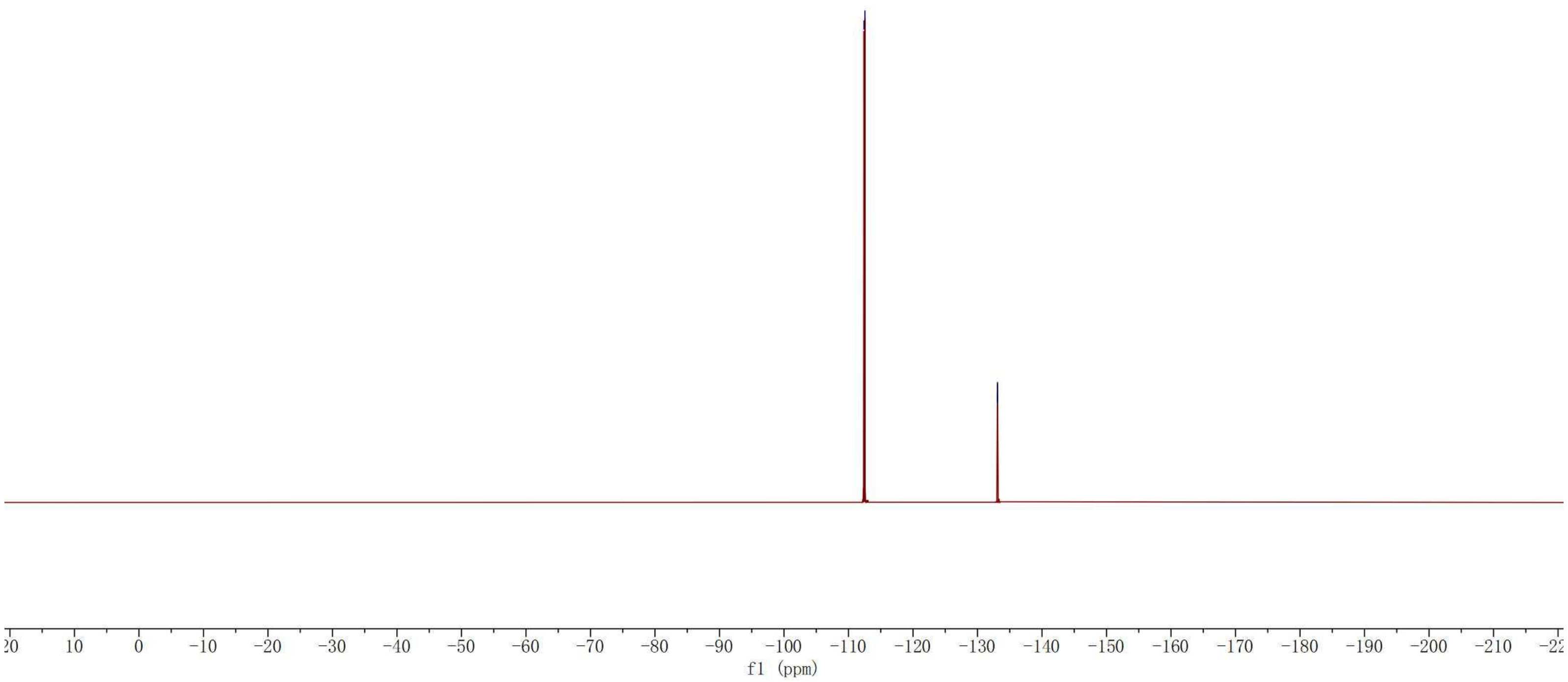

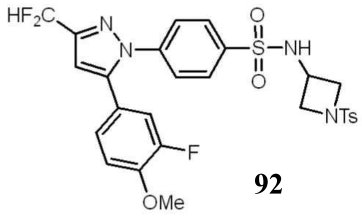

92

^{13}C NMR ($CDCl_3$, 101 MHz)

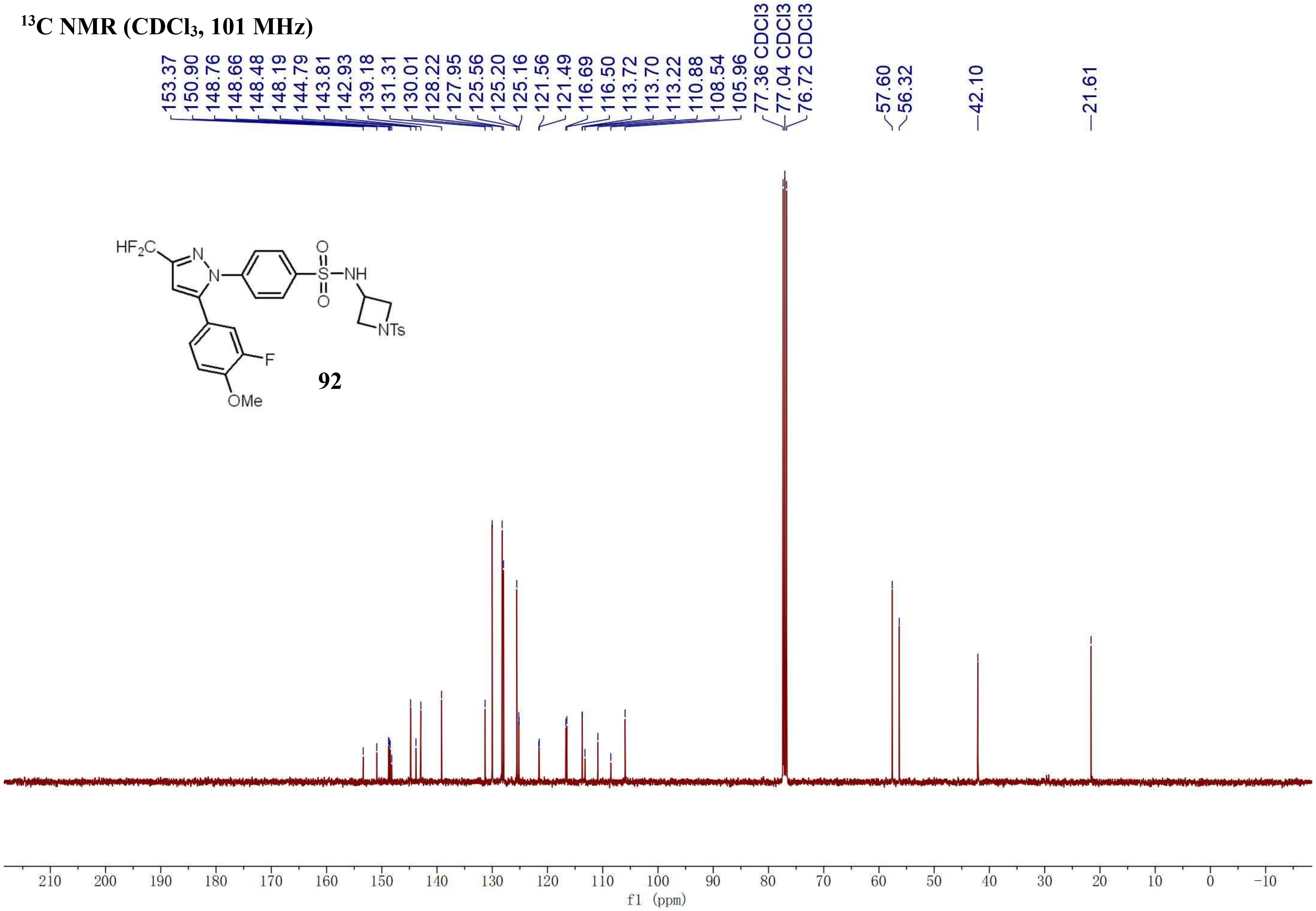

^{1}H NMR ($CDCl_3$, 400 MHz)

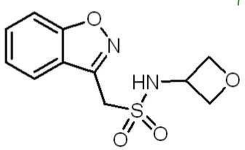

93

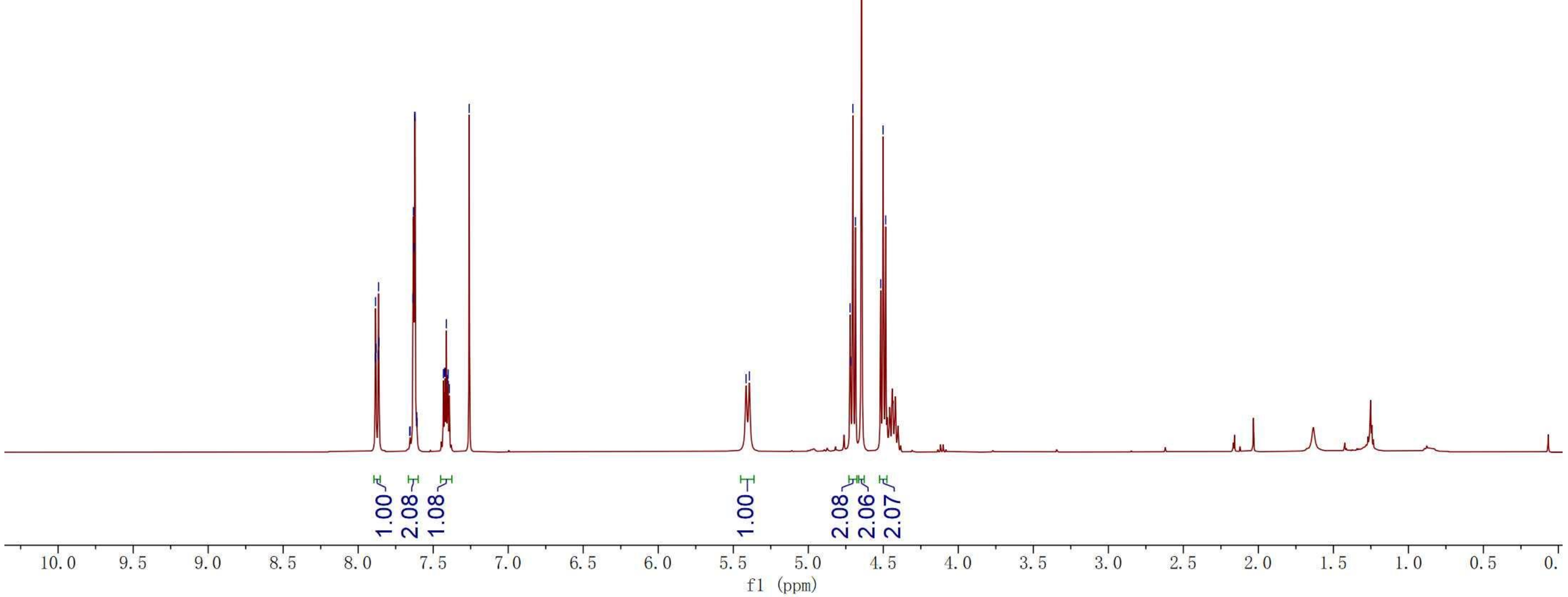

^{13}C NMR (CDCl$_3$, 101 MHz)

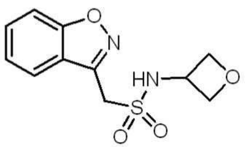

93

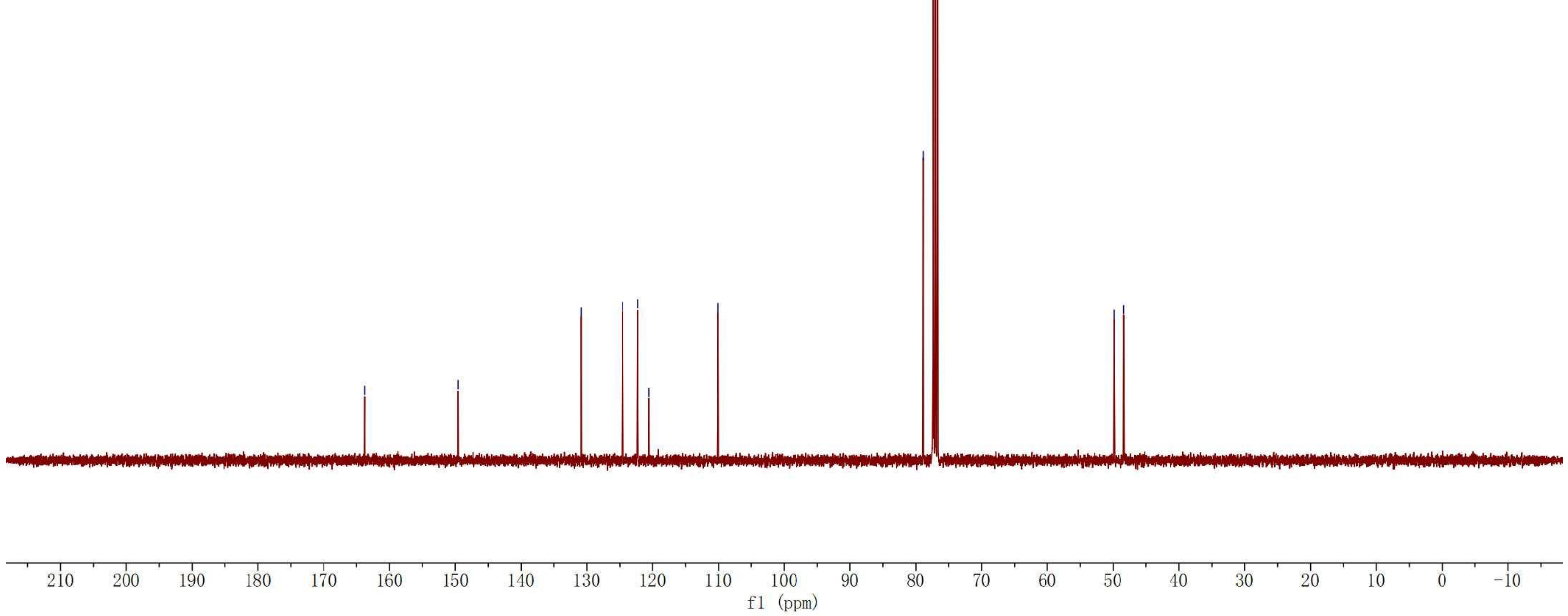

^{1}H NMR ($CDCl_3$, 400 MHz)

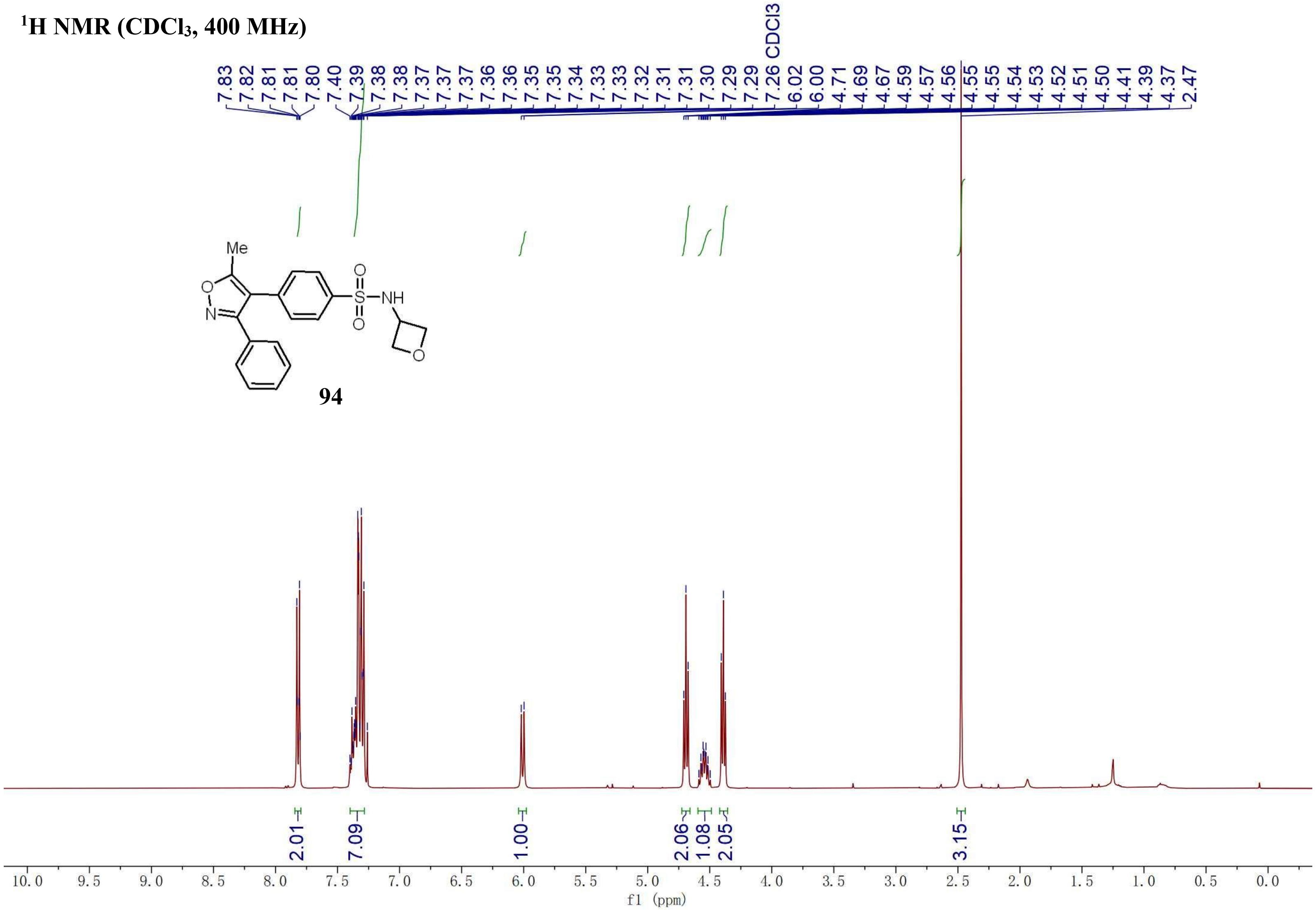

^{13}C NMR ($CDCl_3$, 101 MHz)

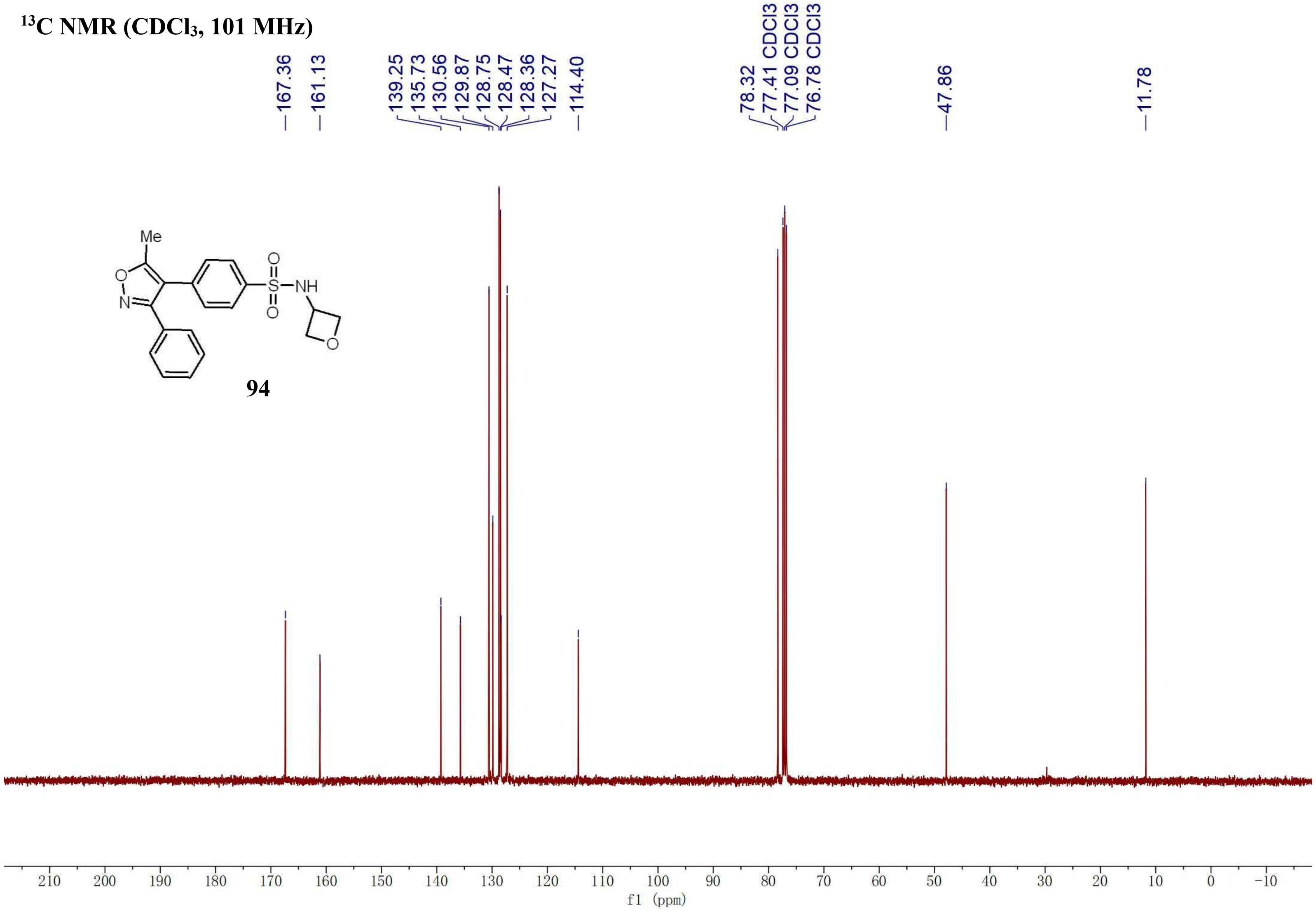

94

^{1}H NMR ($CDCl_3$, 400 MHz)

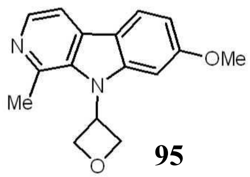

95

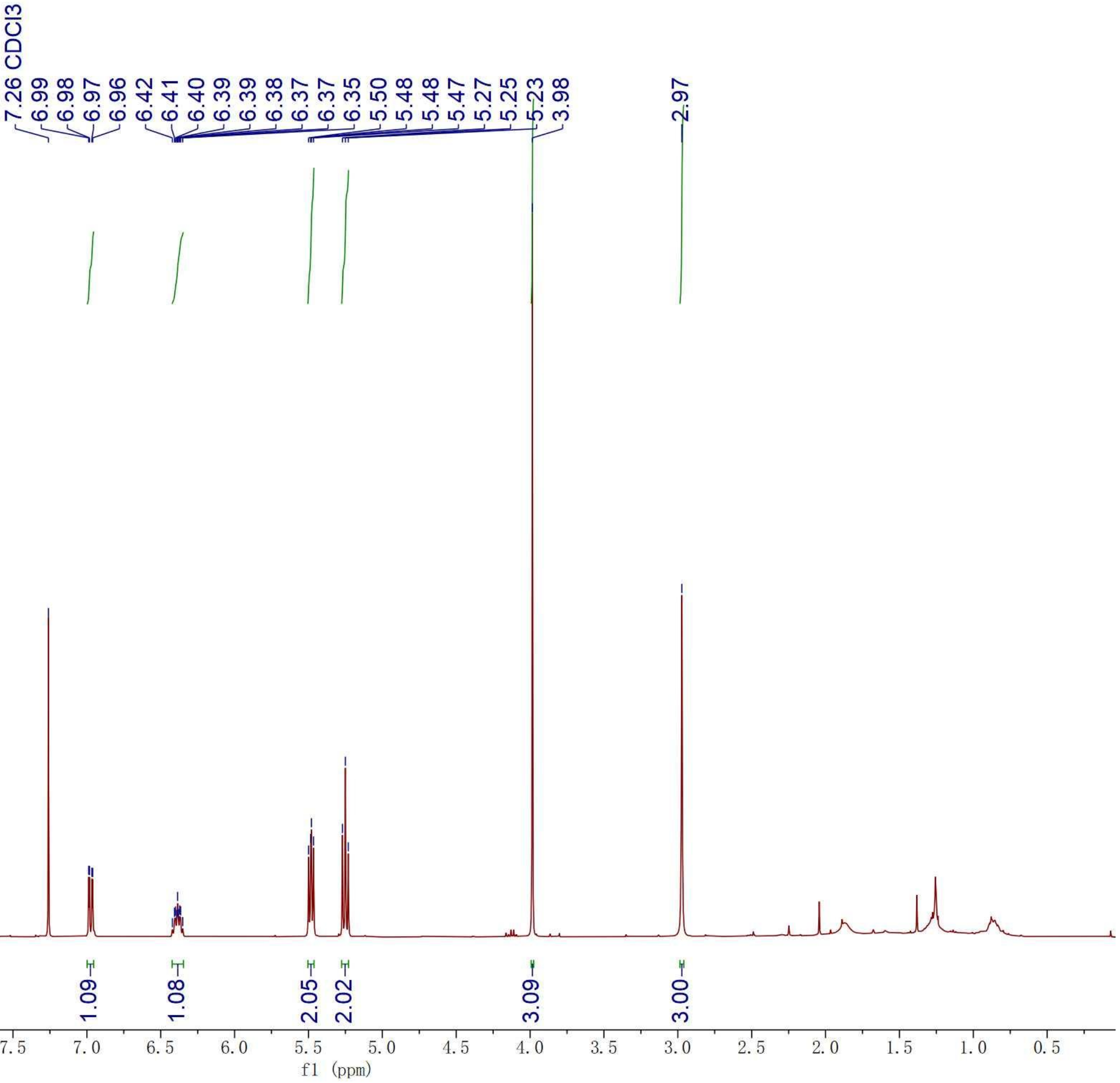

^{13}C NMR ($CDCl_3$, 101 MHz)

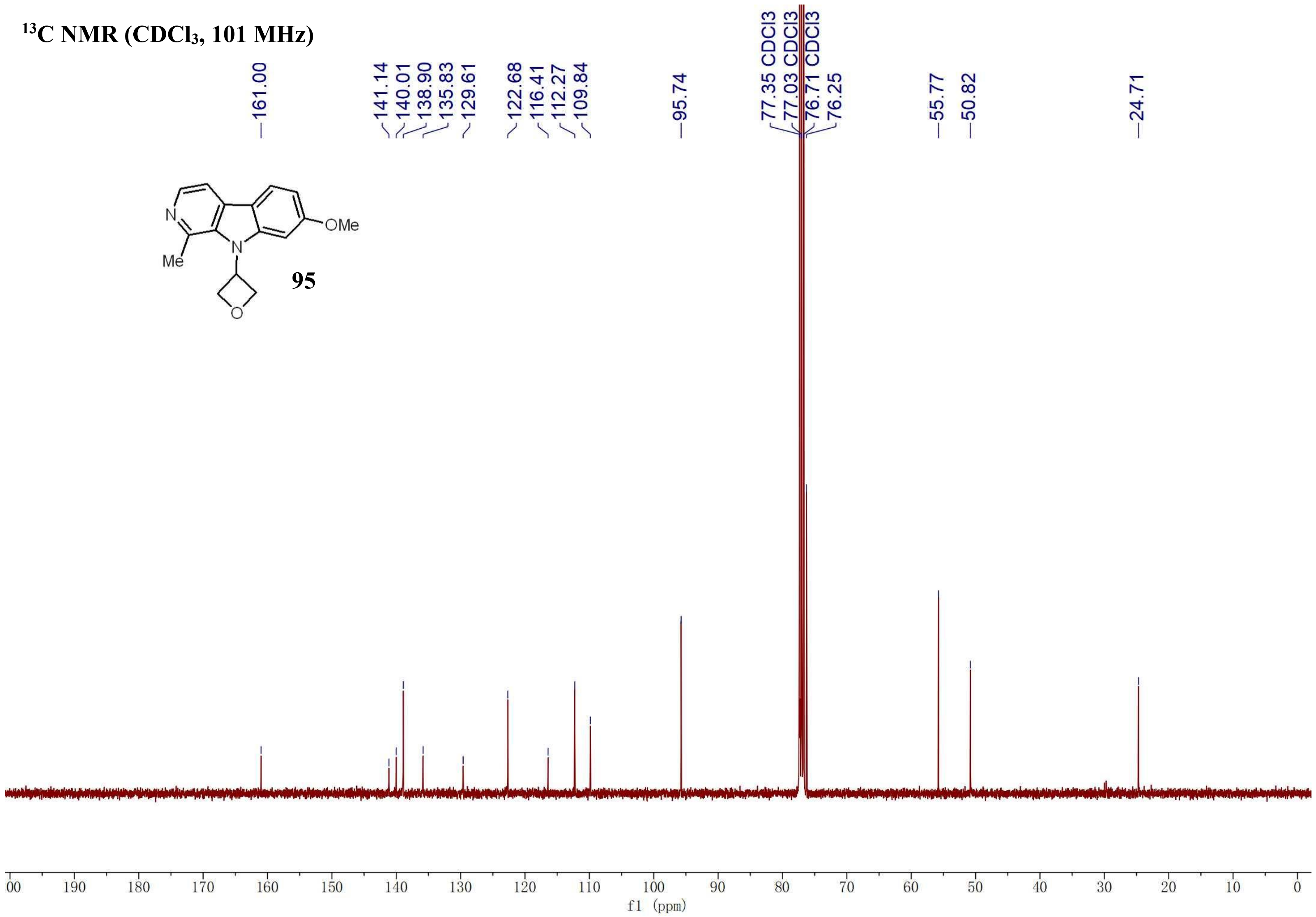

^{1}H NMR ($CDCl_3$, 400 MHz)

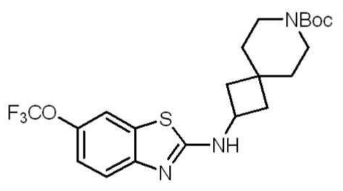

96

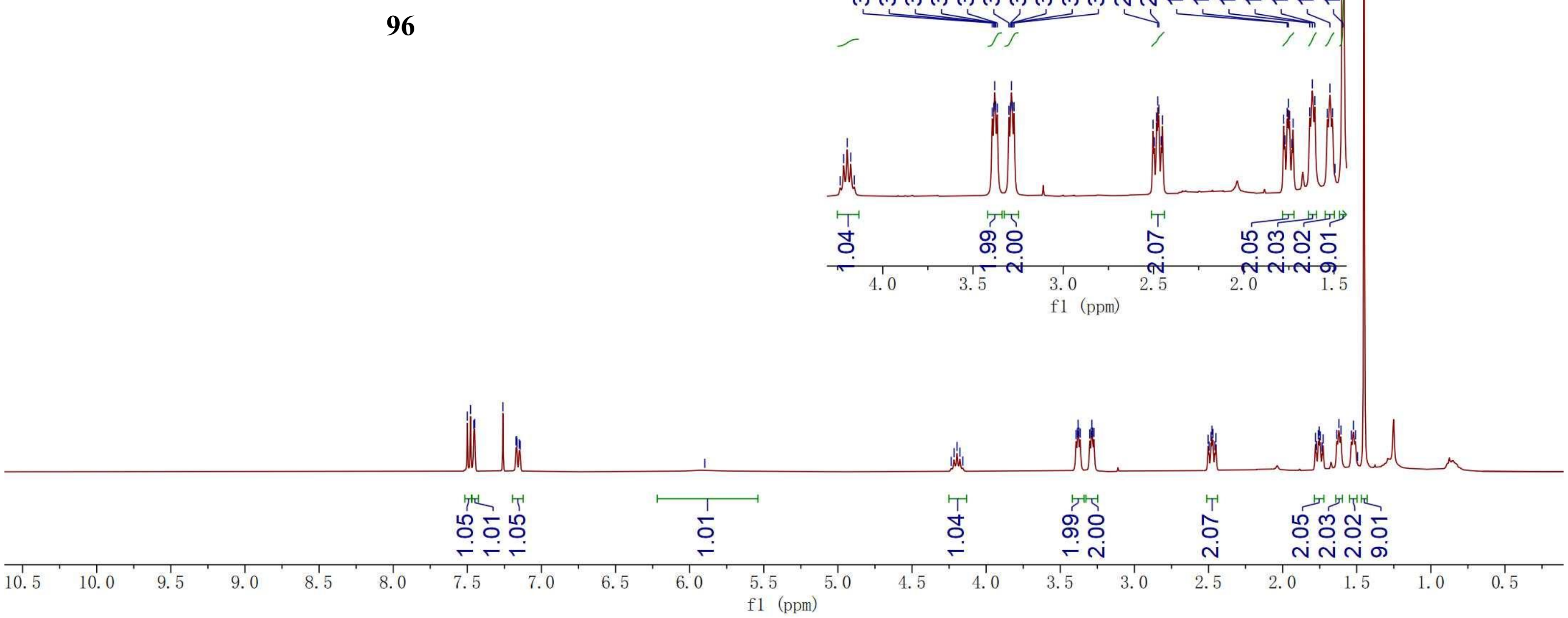

^{19}F NMR ($CDCl_3$, 376 MHz)

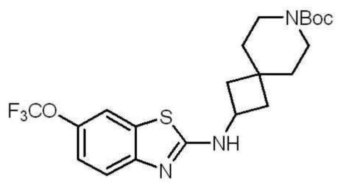

96

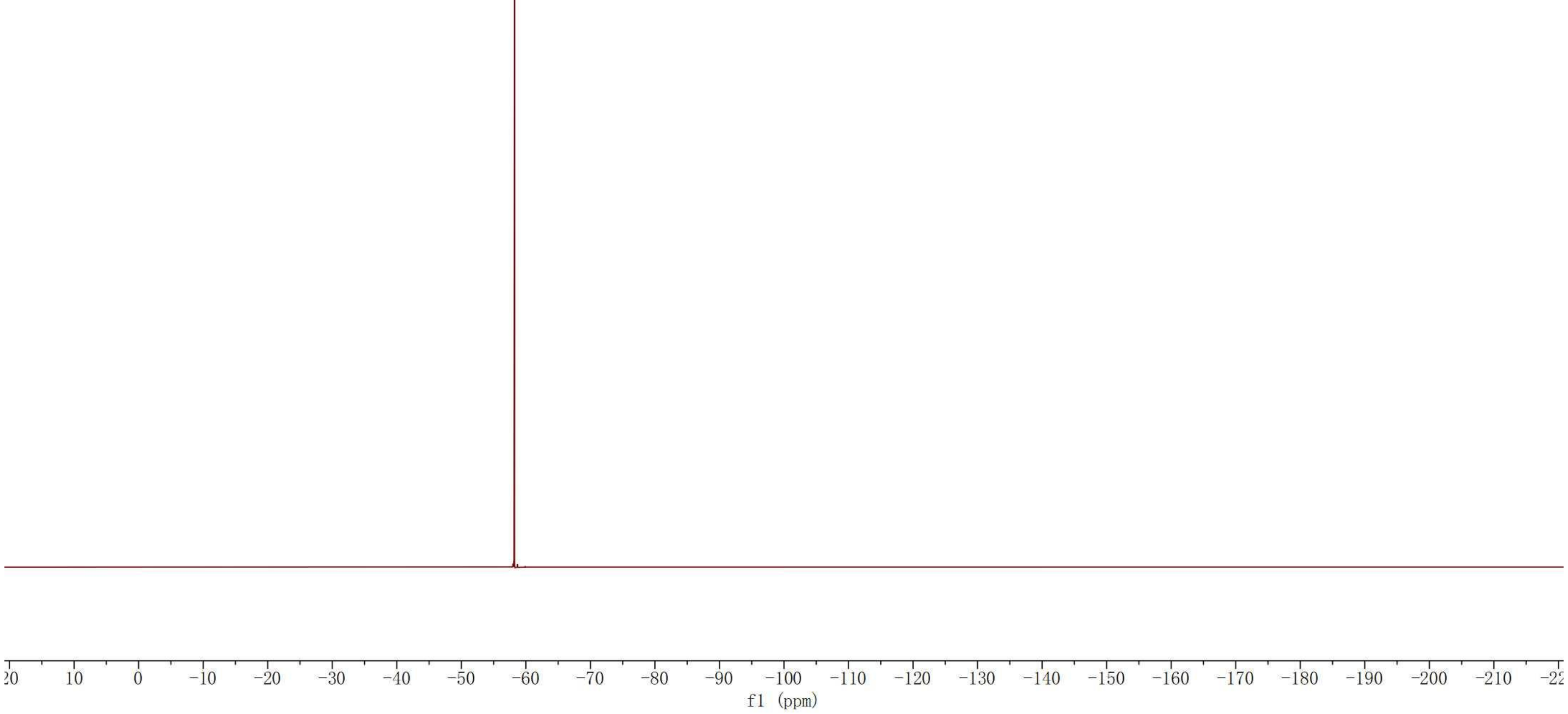

^{13}C NMR ($CDCl_3$, 101 MHz)

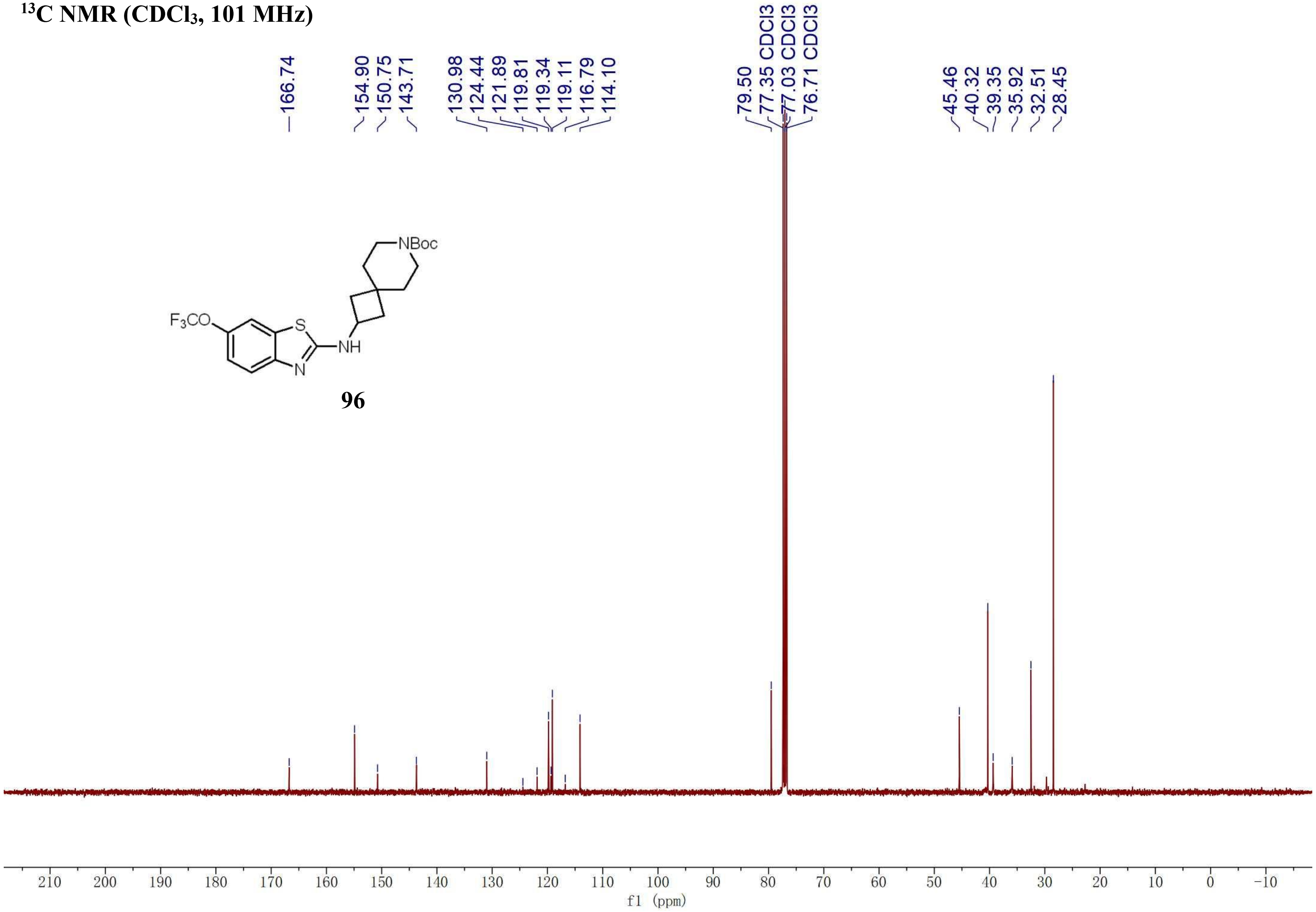

96

^{1}H NMR ($CDCl_3$, 400 MHz)

97

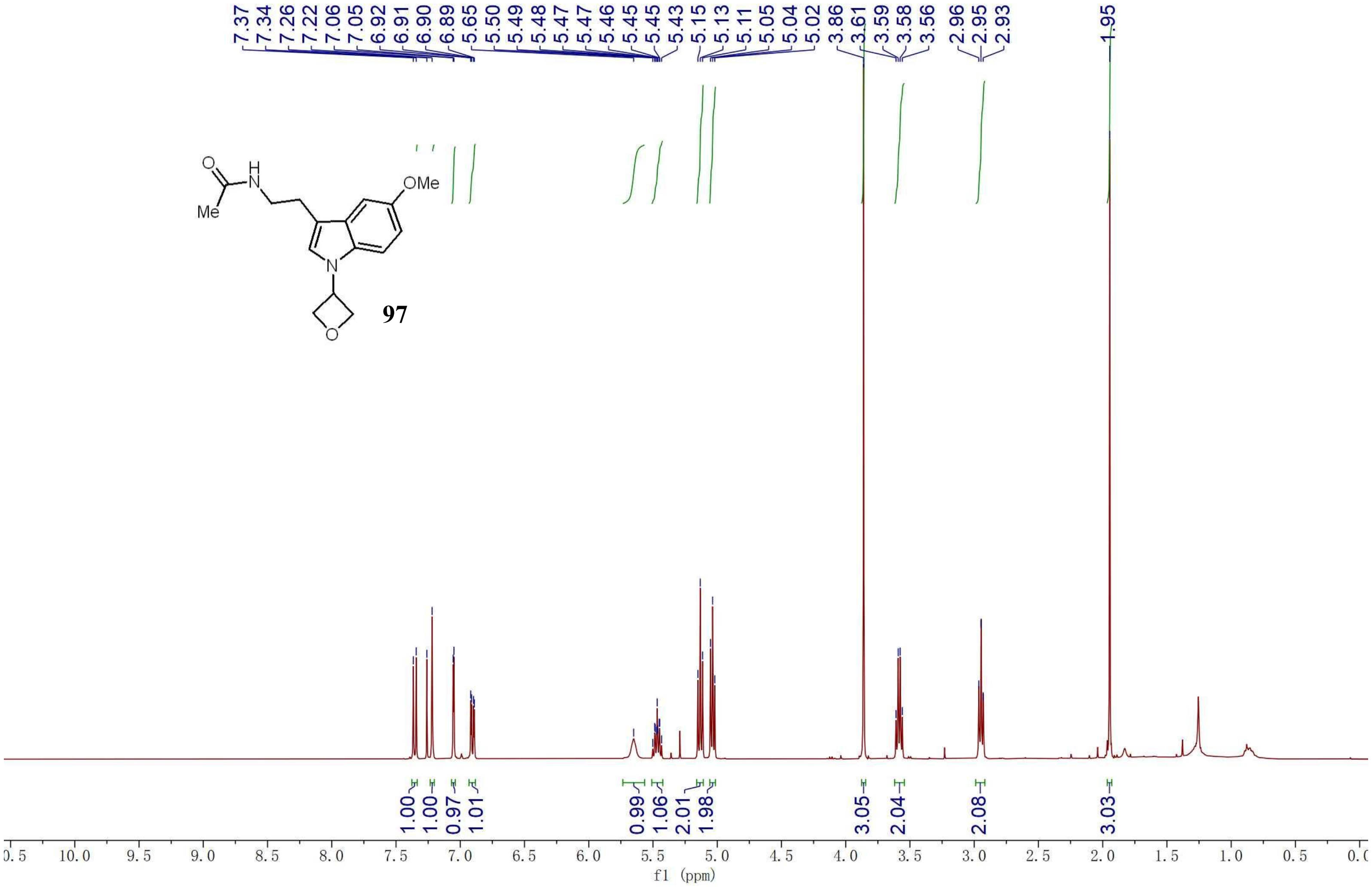

^{13}C NMR ($CDCl_3$, 101 MHz)

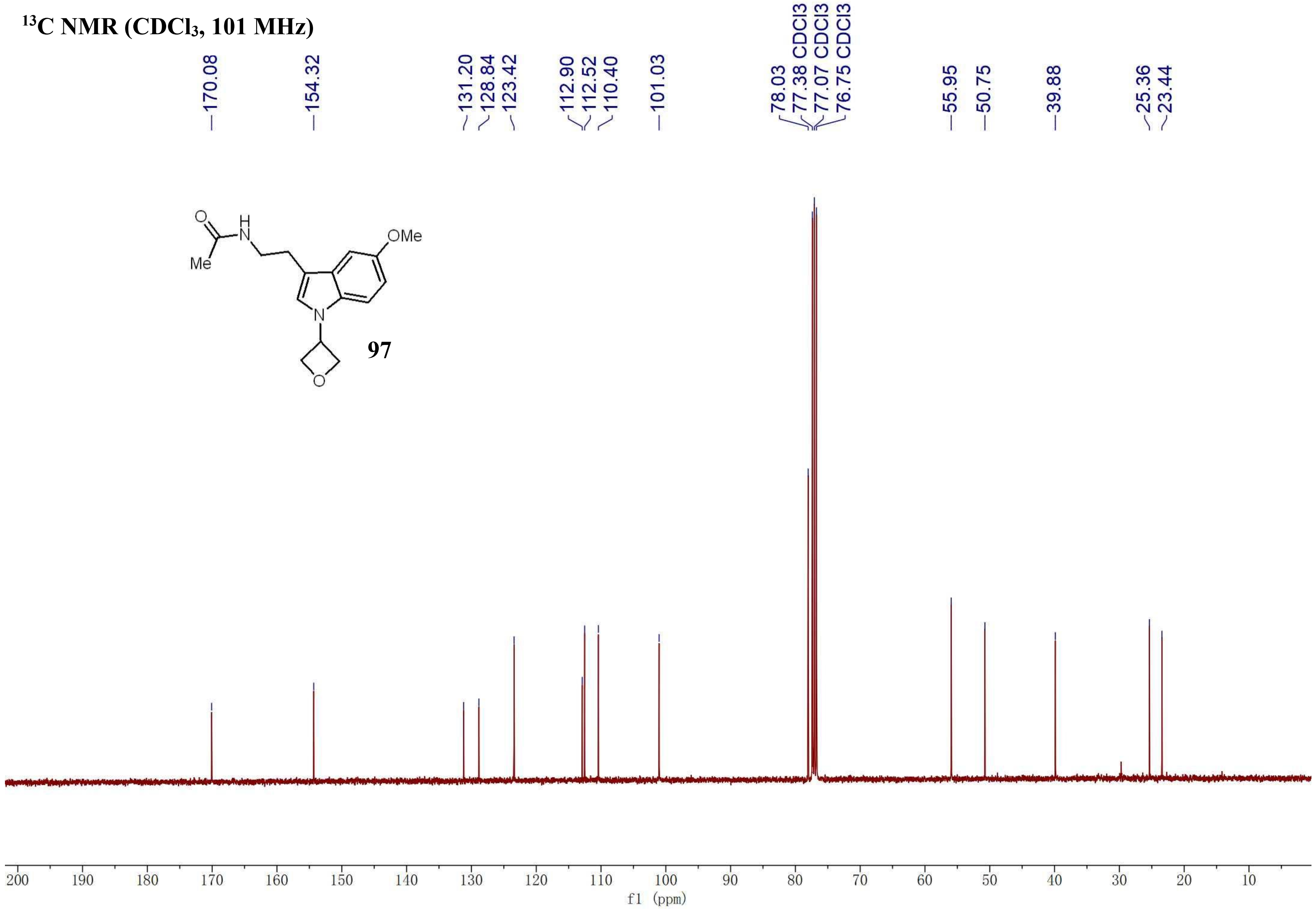

^{1}H NMR ($CDCl_3$, 400 MHz)

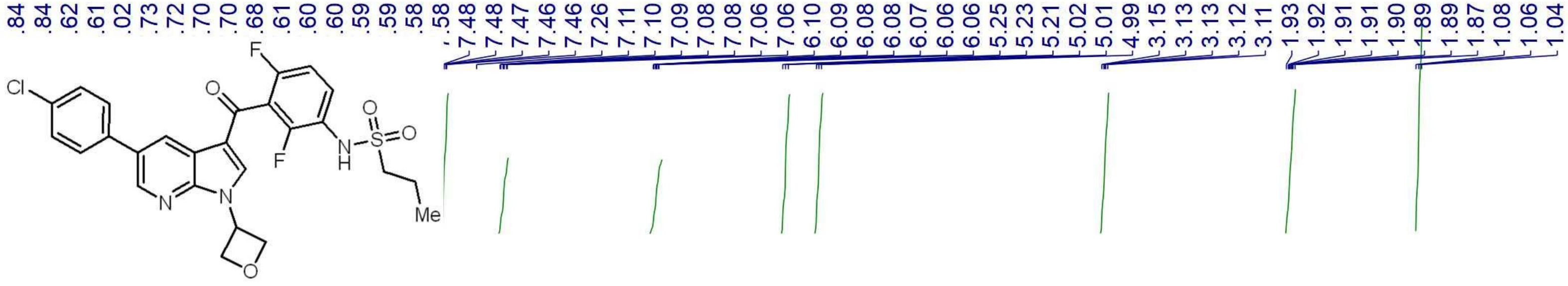

98

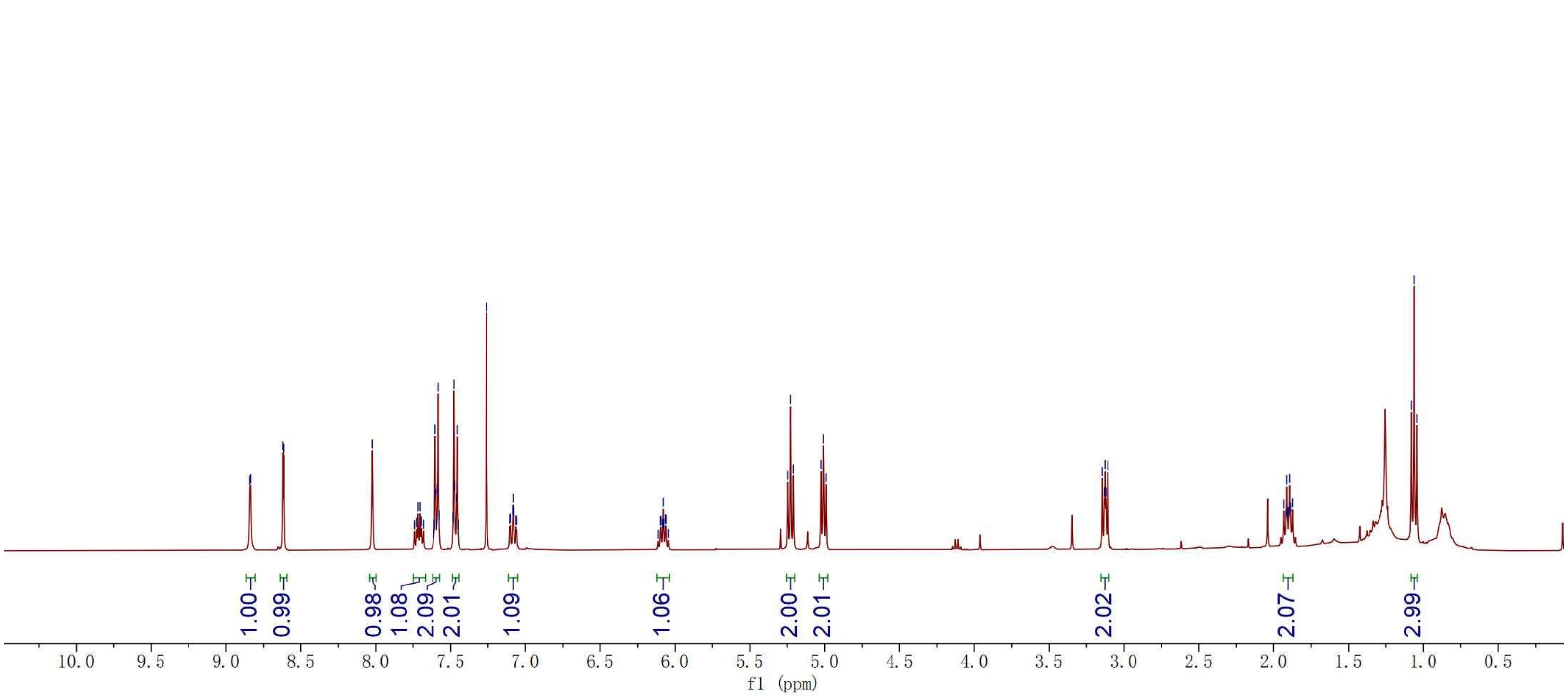

^{19}F NMR ($CDCl_3$, 376 MHz)

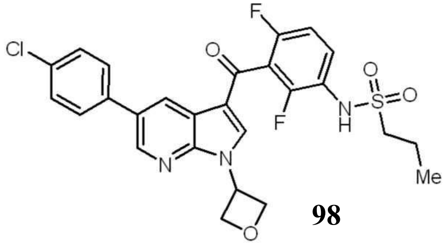

98

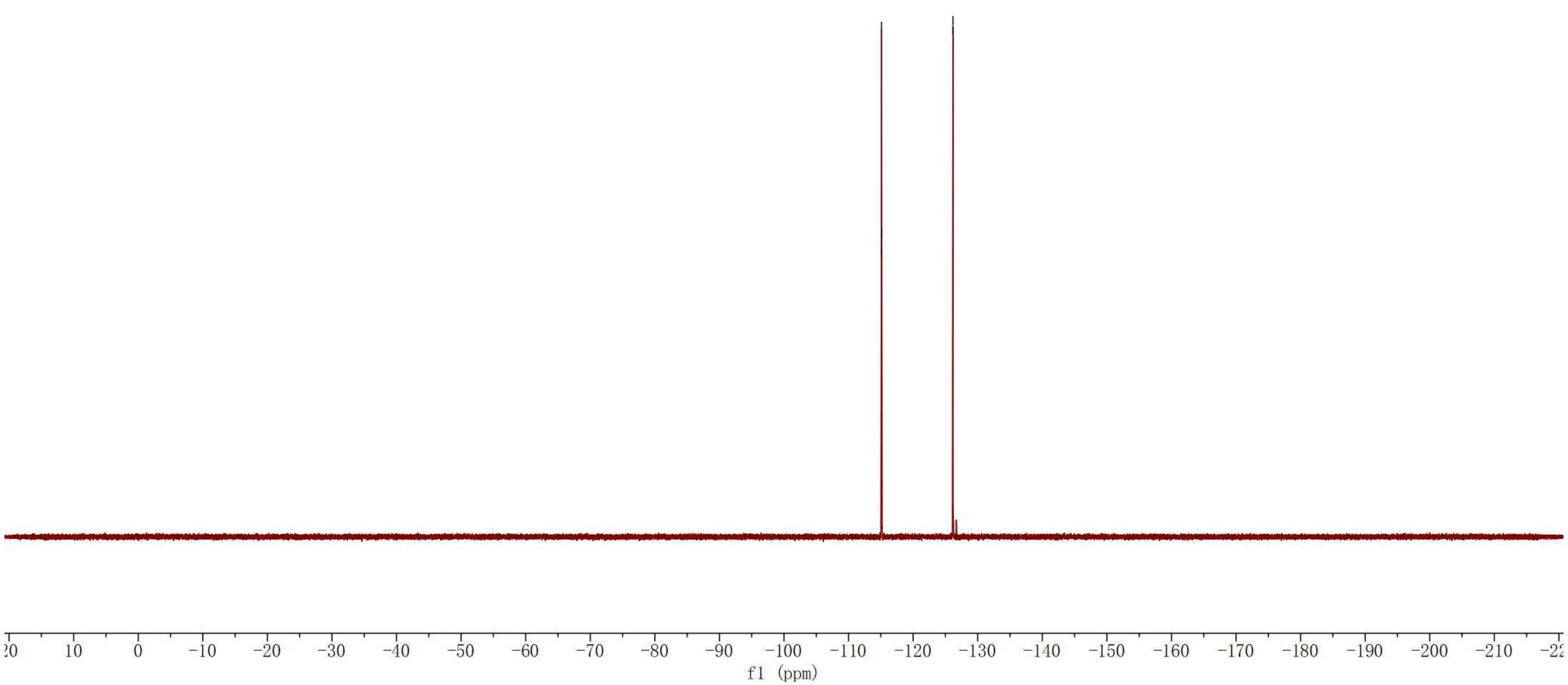

^{13}C NMR ($CDCl_3$, 101 MHz)

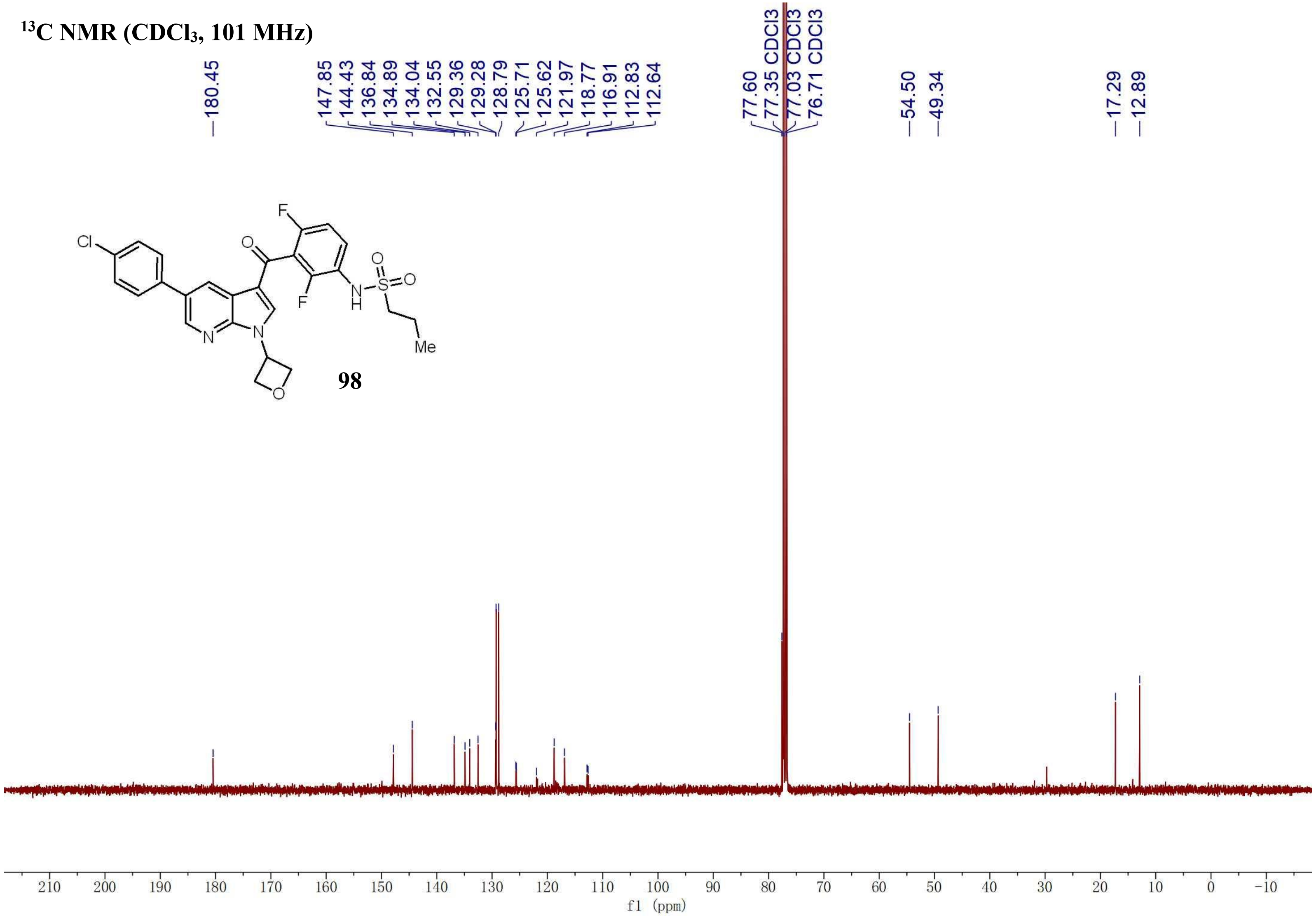

98

^{1}H NMR ($CDCl_3$, 400 MHz)

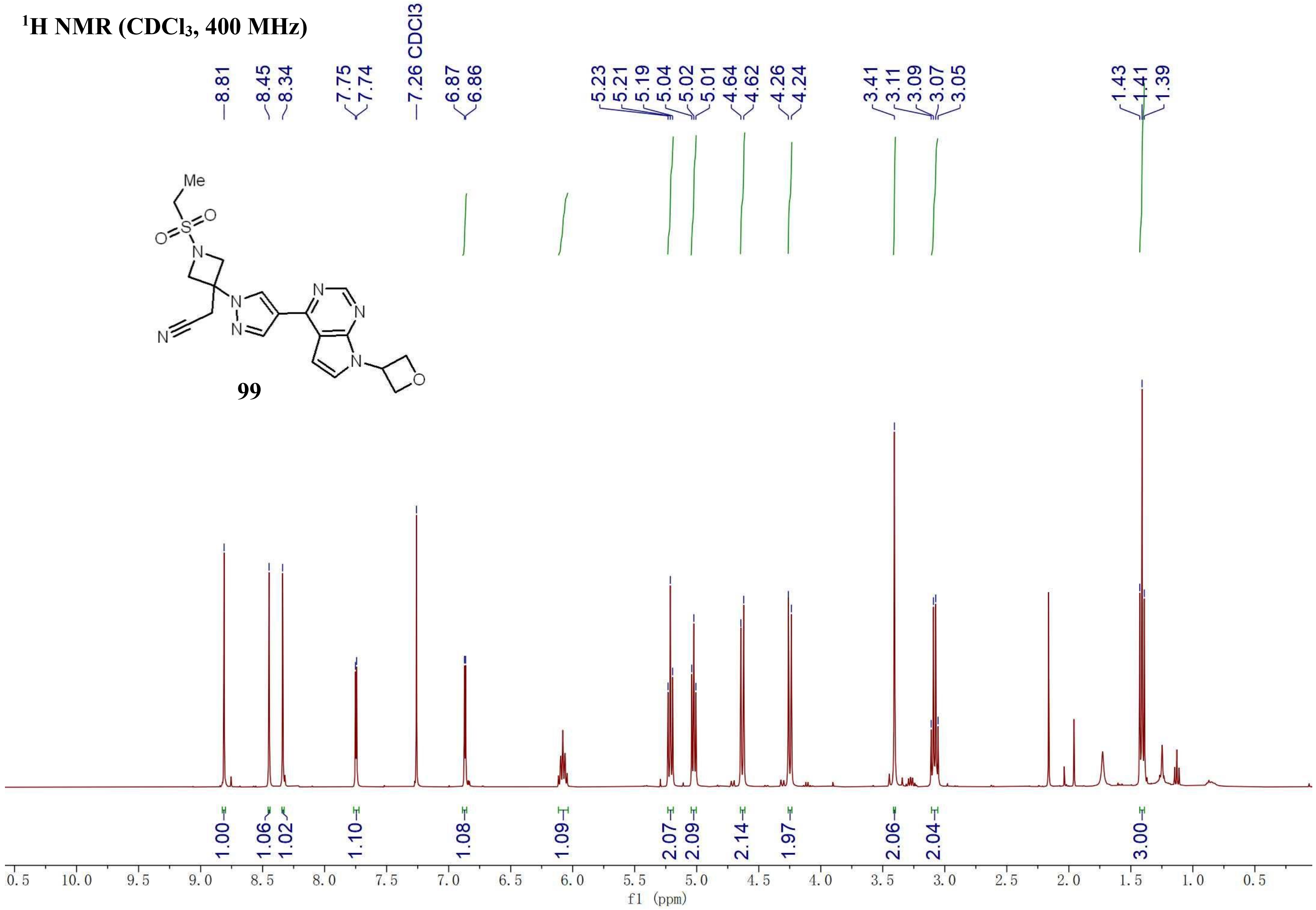

^{13}C NMR (CDCl3, 101 MHz)

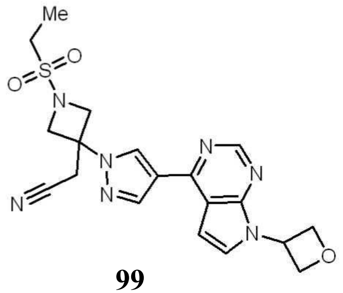

99

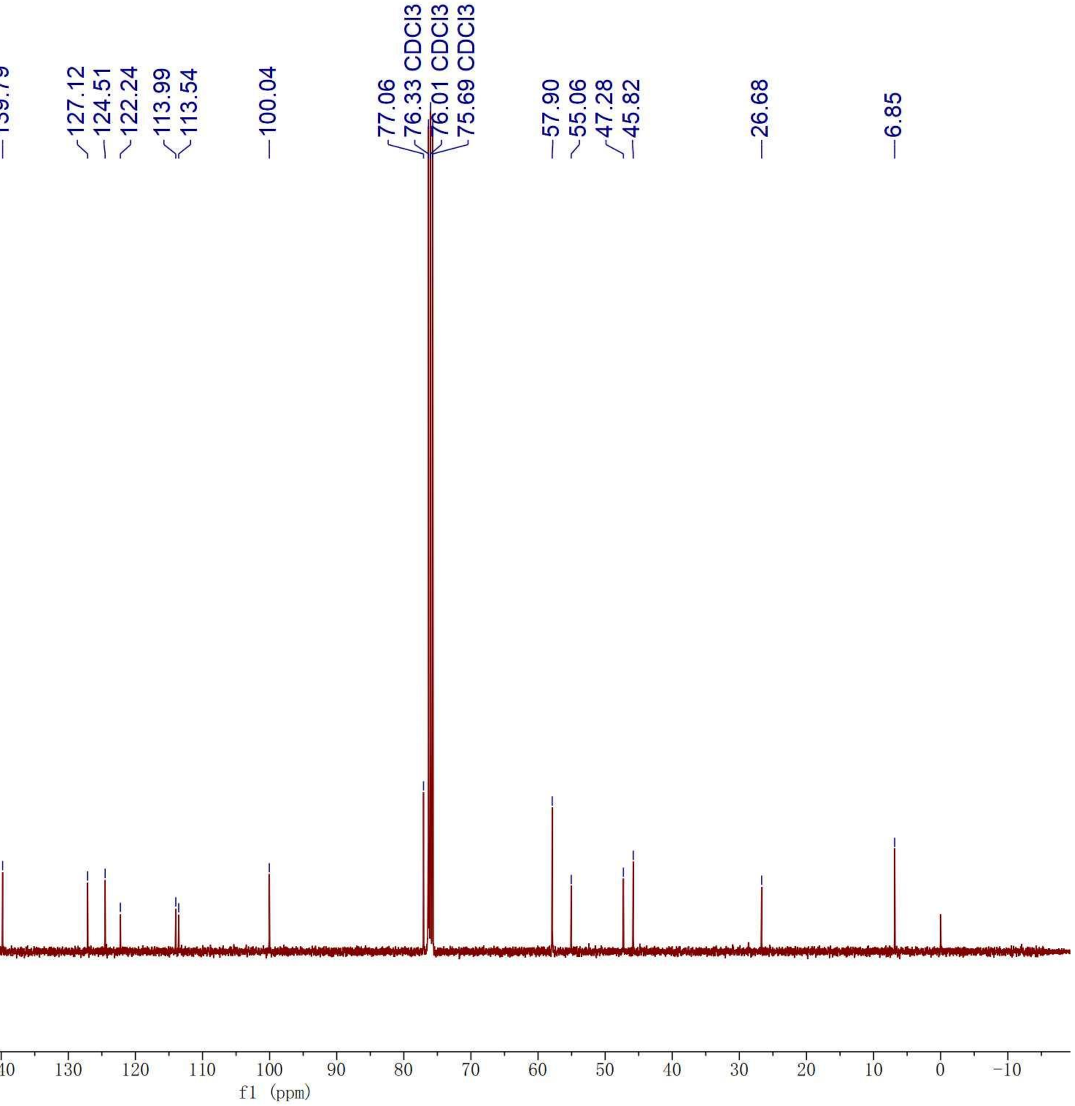

^{1}H NMR ($CDCl_3$, 400 MHz)

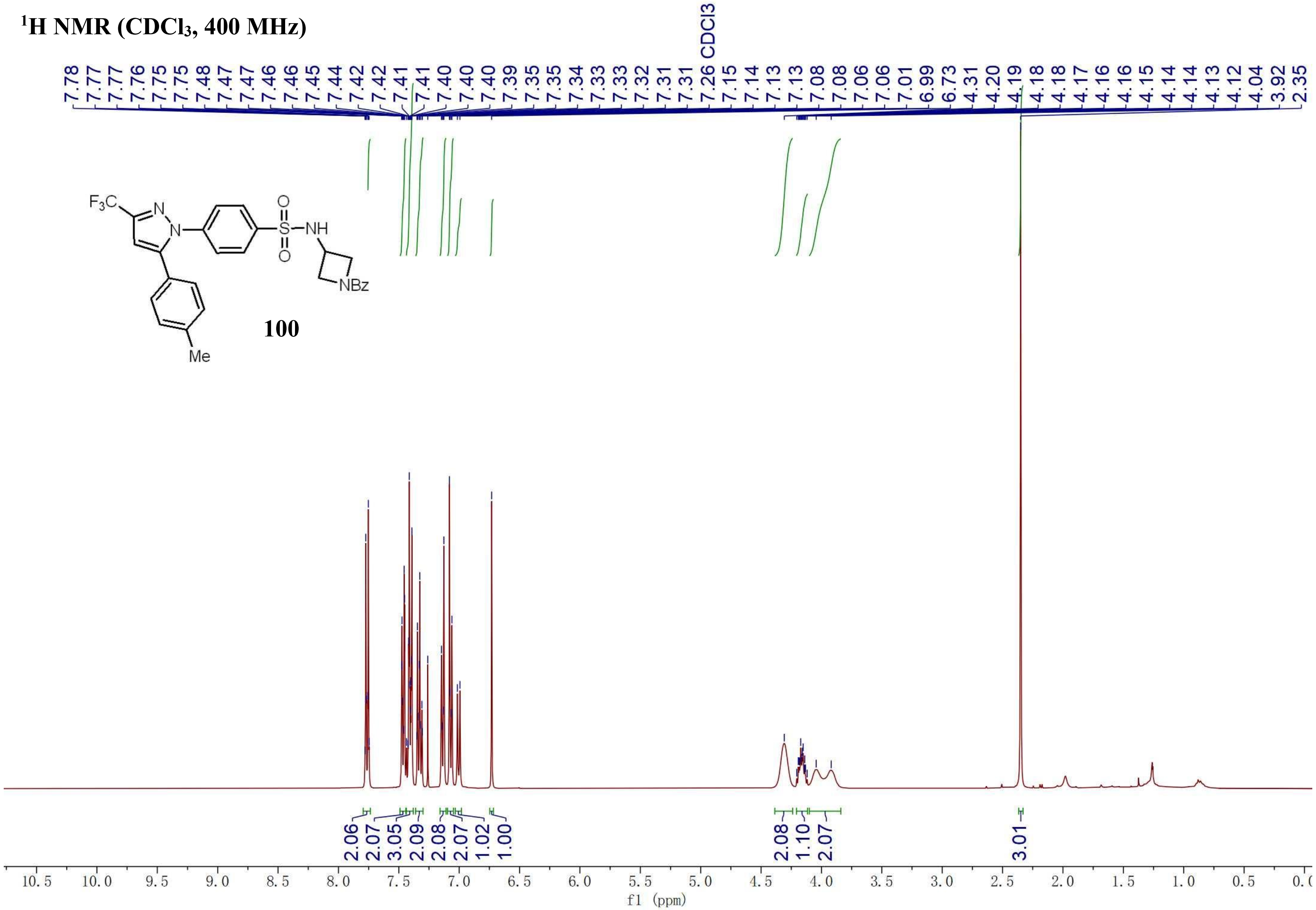

^{19}F NMR ($CDCl_3$, 376 MHz)

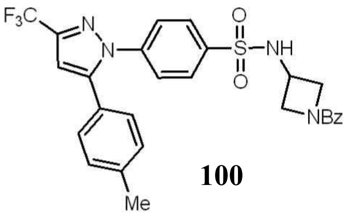

100

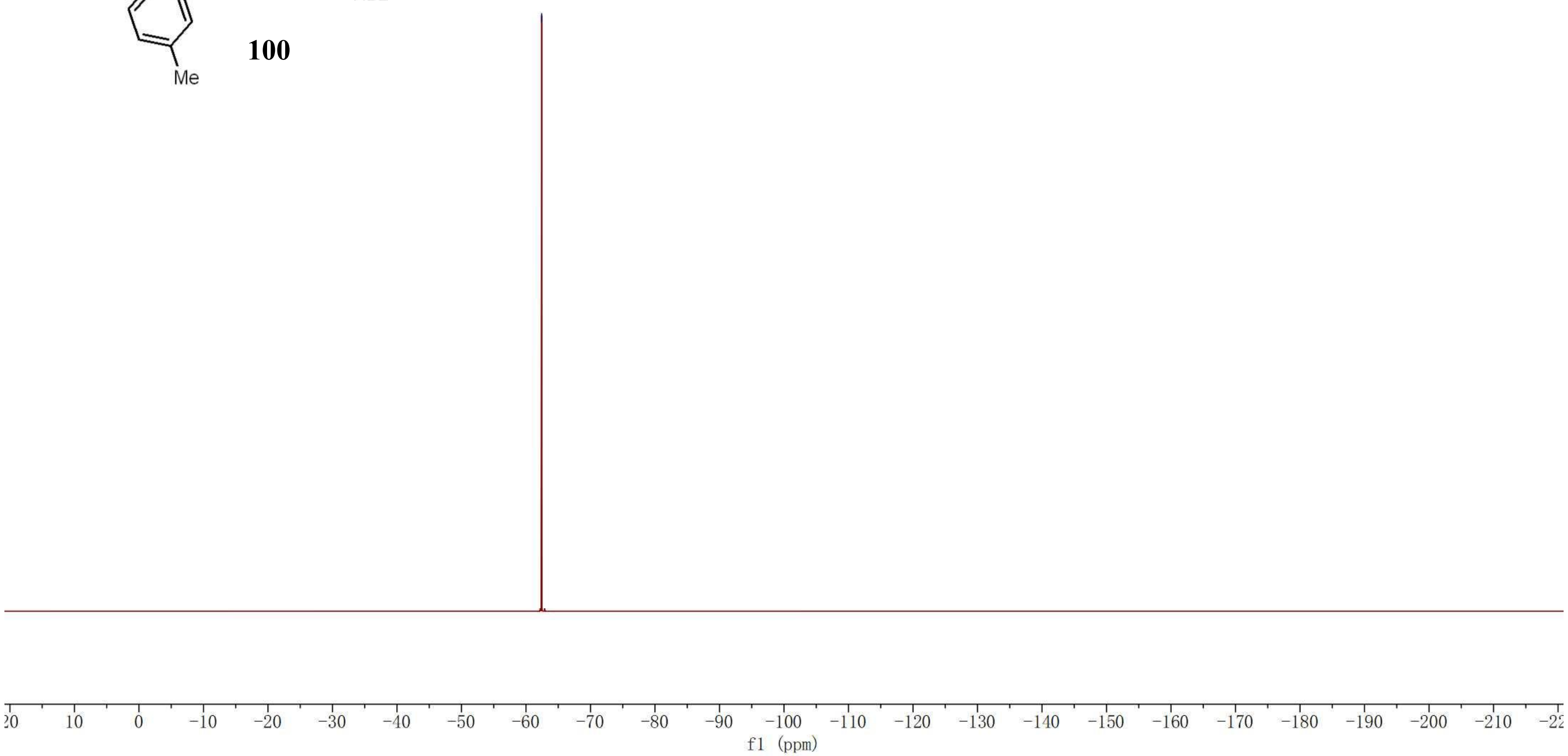

^{13}C NMR ($CDCl_3$, 101 MHz)

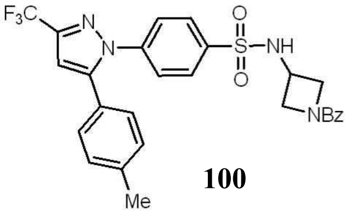

100

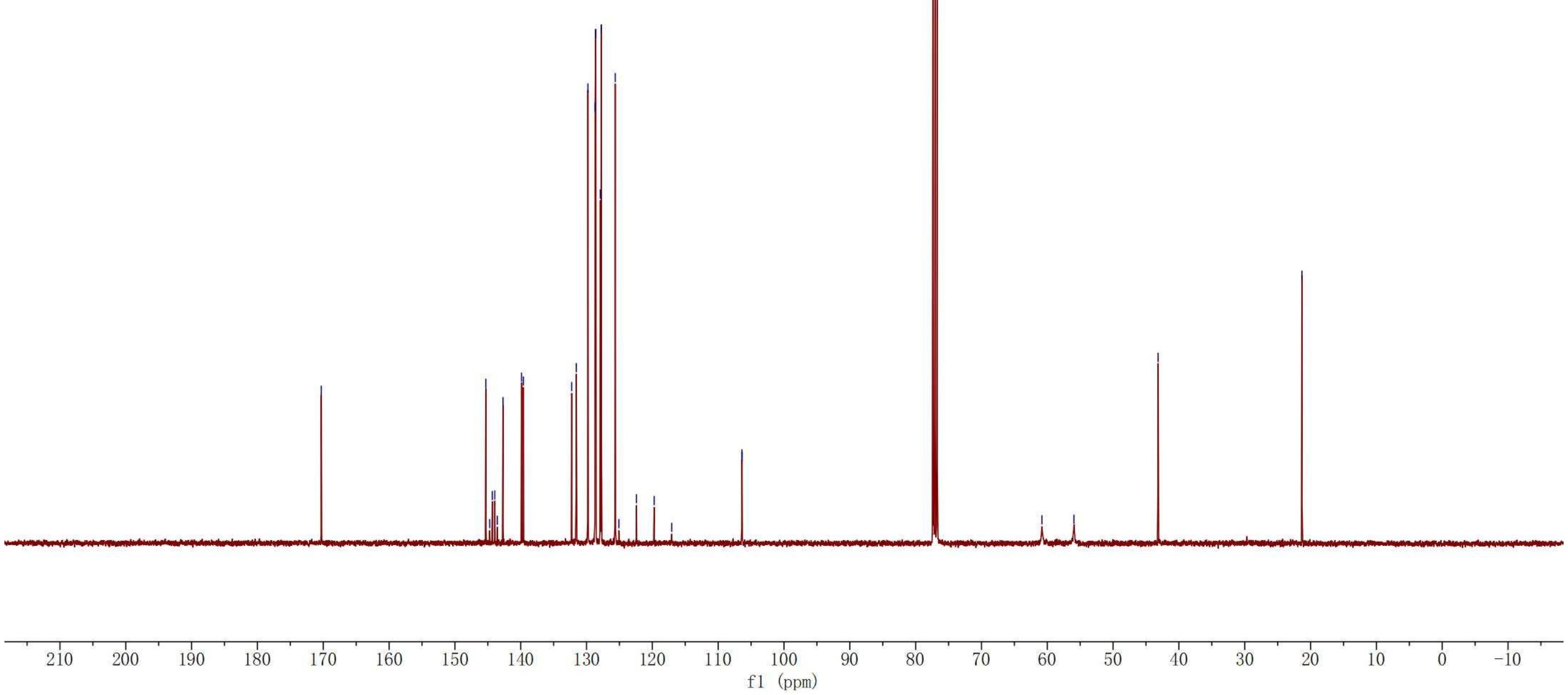

^{1}H NMR (CDCl$_3$, 400 MHz)

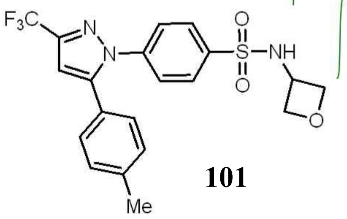

101

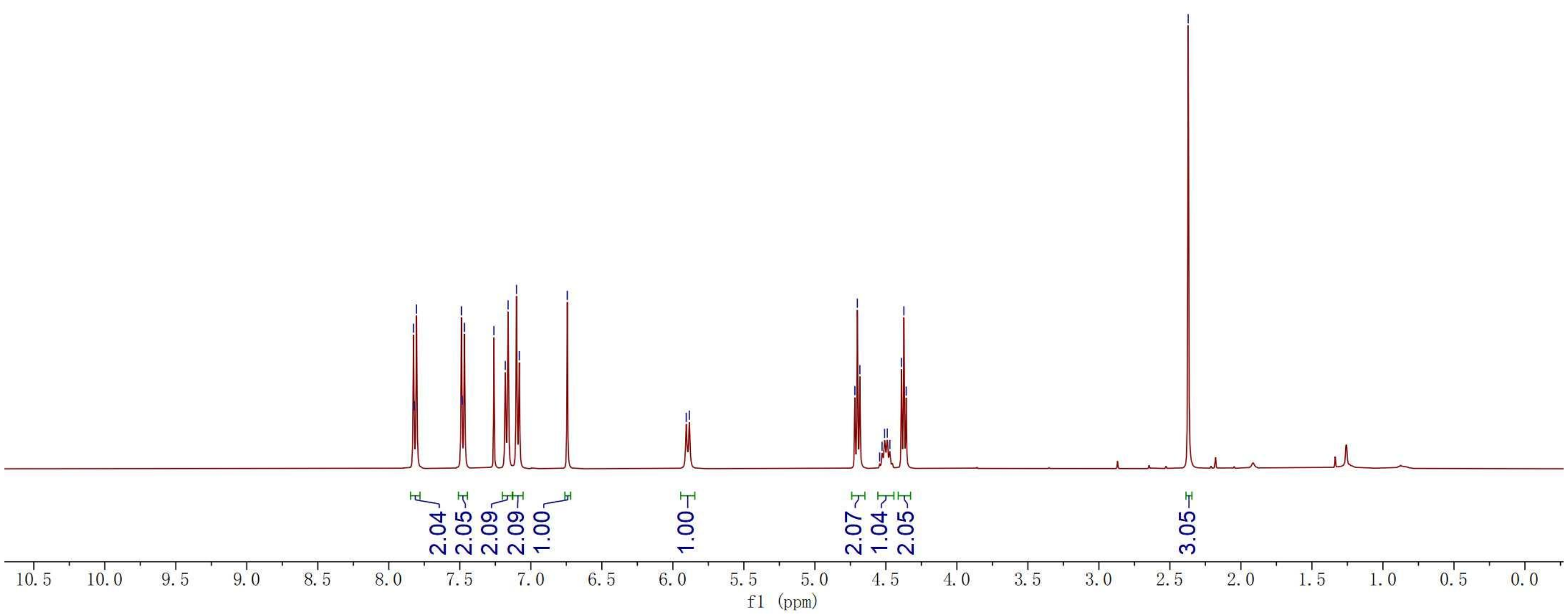

^{19}F NMR ($CDCl_3$, 376 MHz)

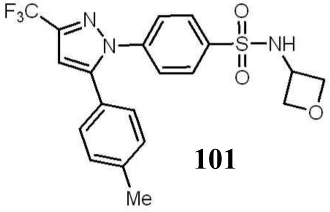

101

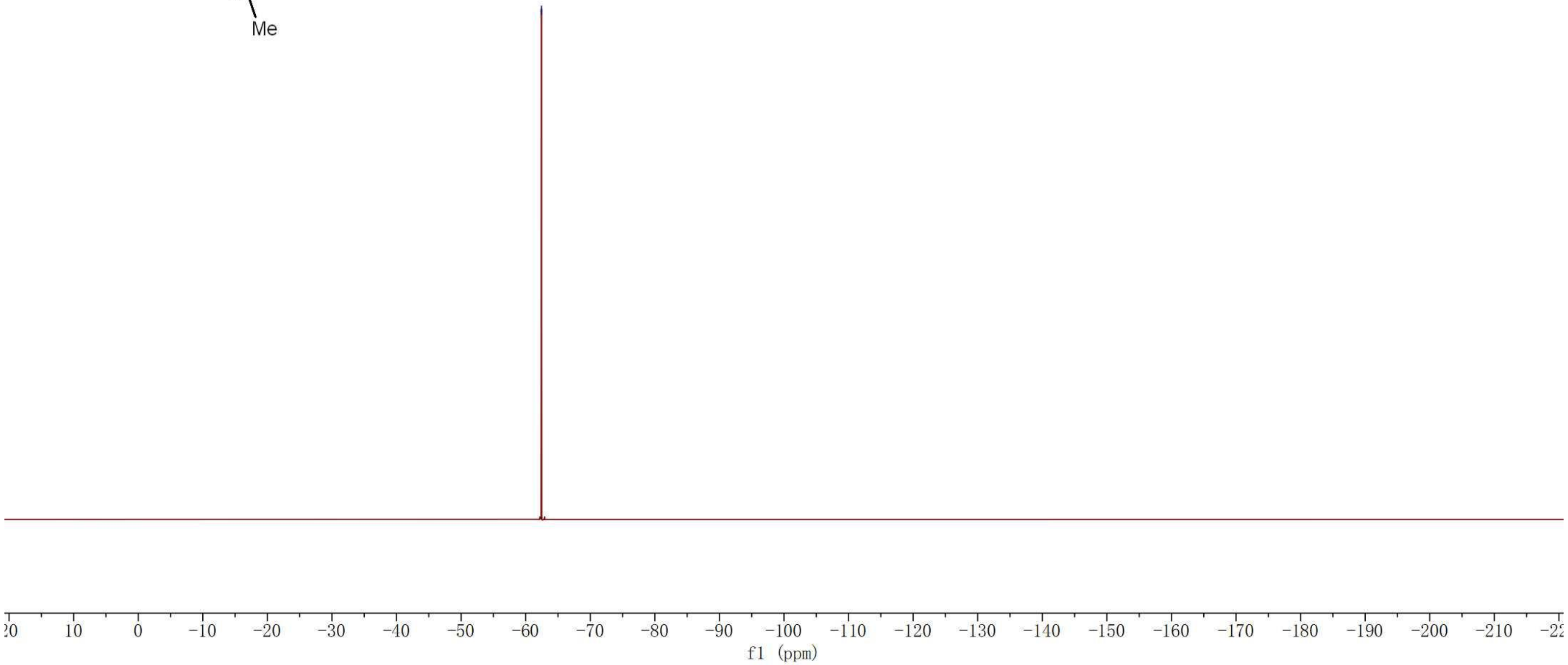

^{13}C NMR (CDCl$_3$, 101 MHz)

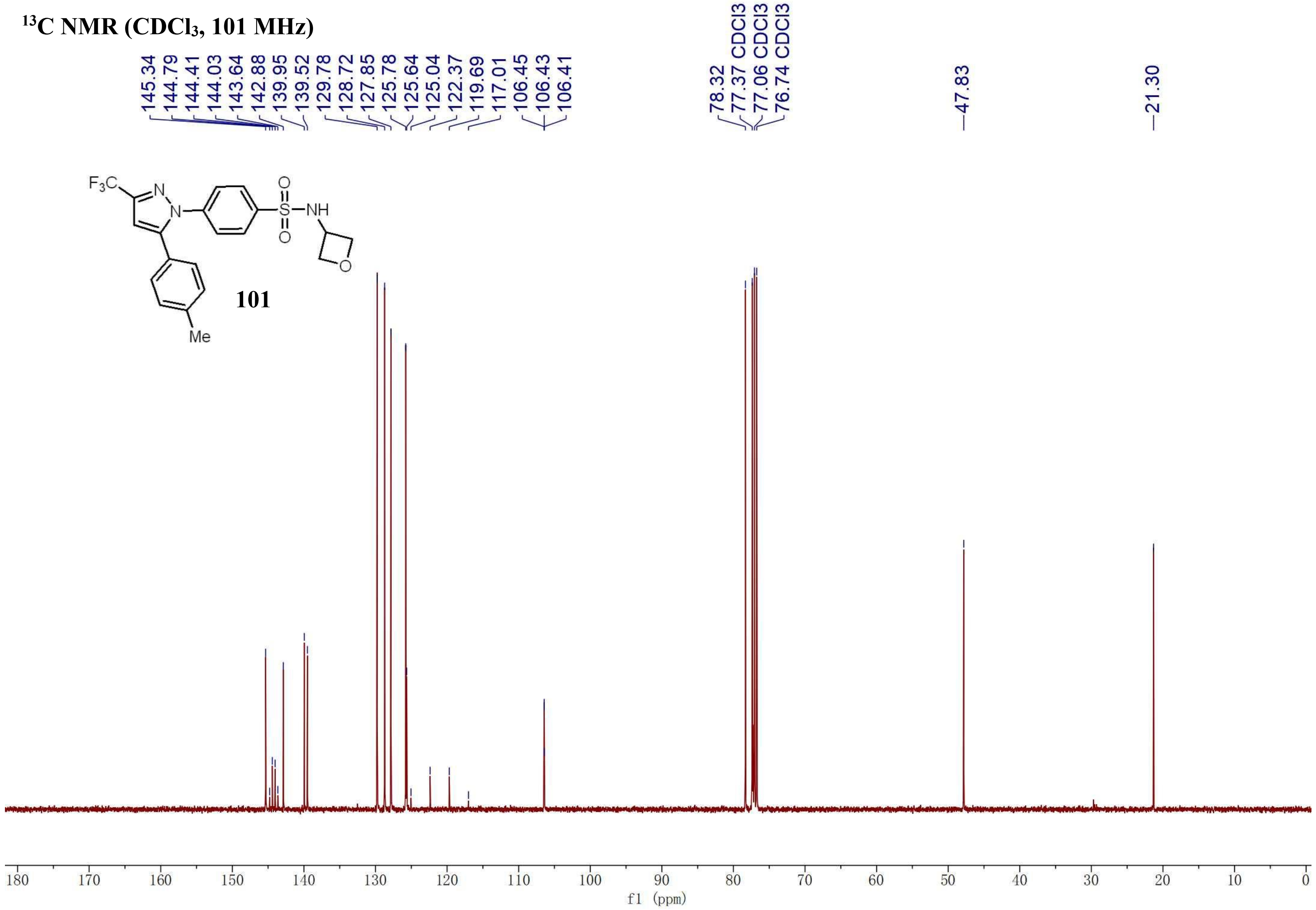

^{1}H NMR ($CDCl_3$, 400 MHz)

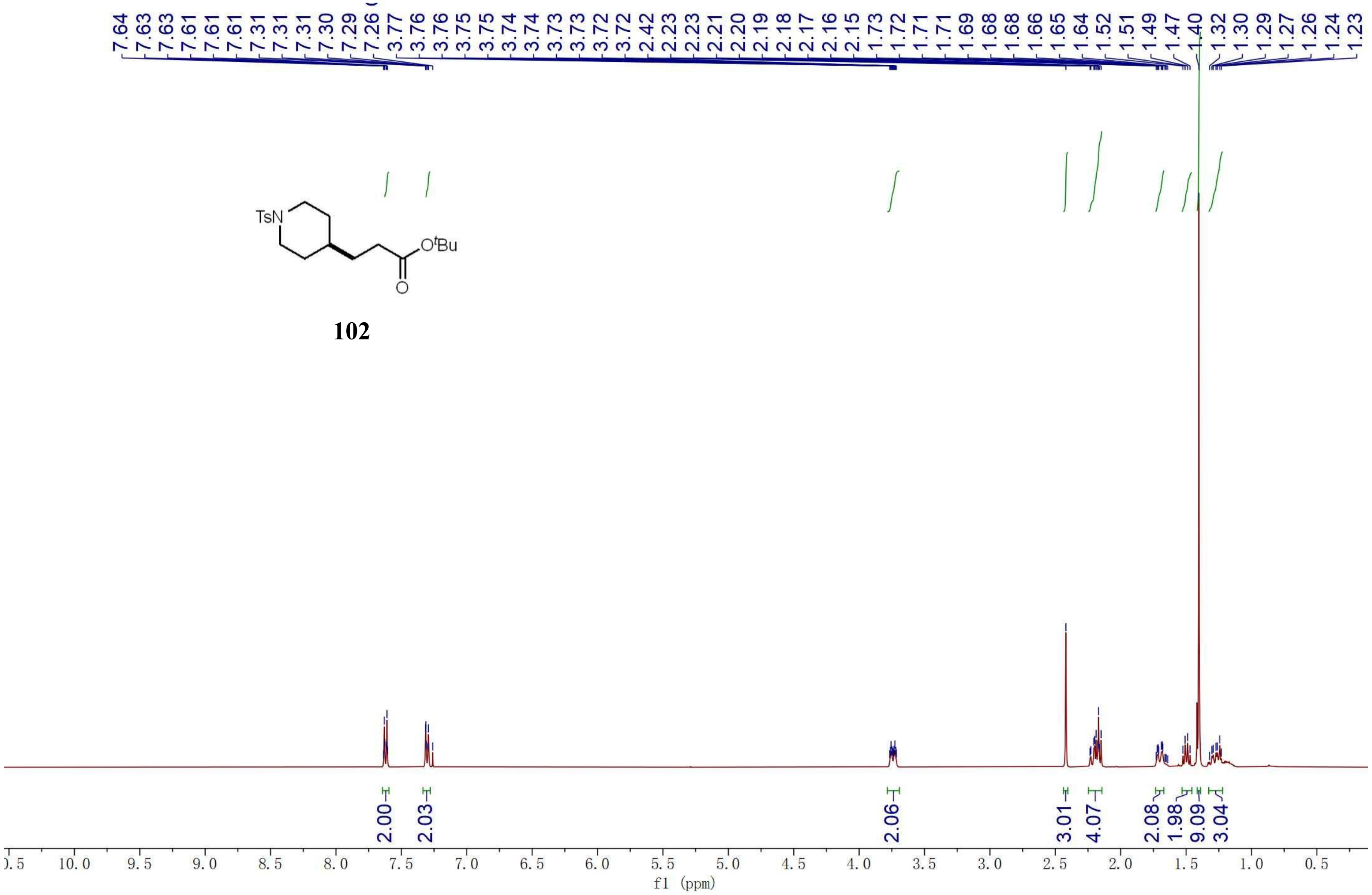

^{13}C NMR ($CDCl_3$, 101 MHz)

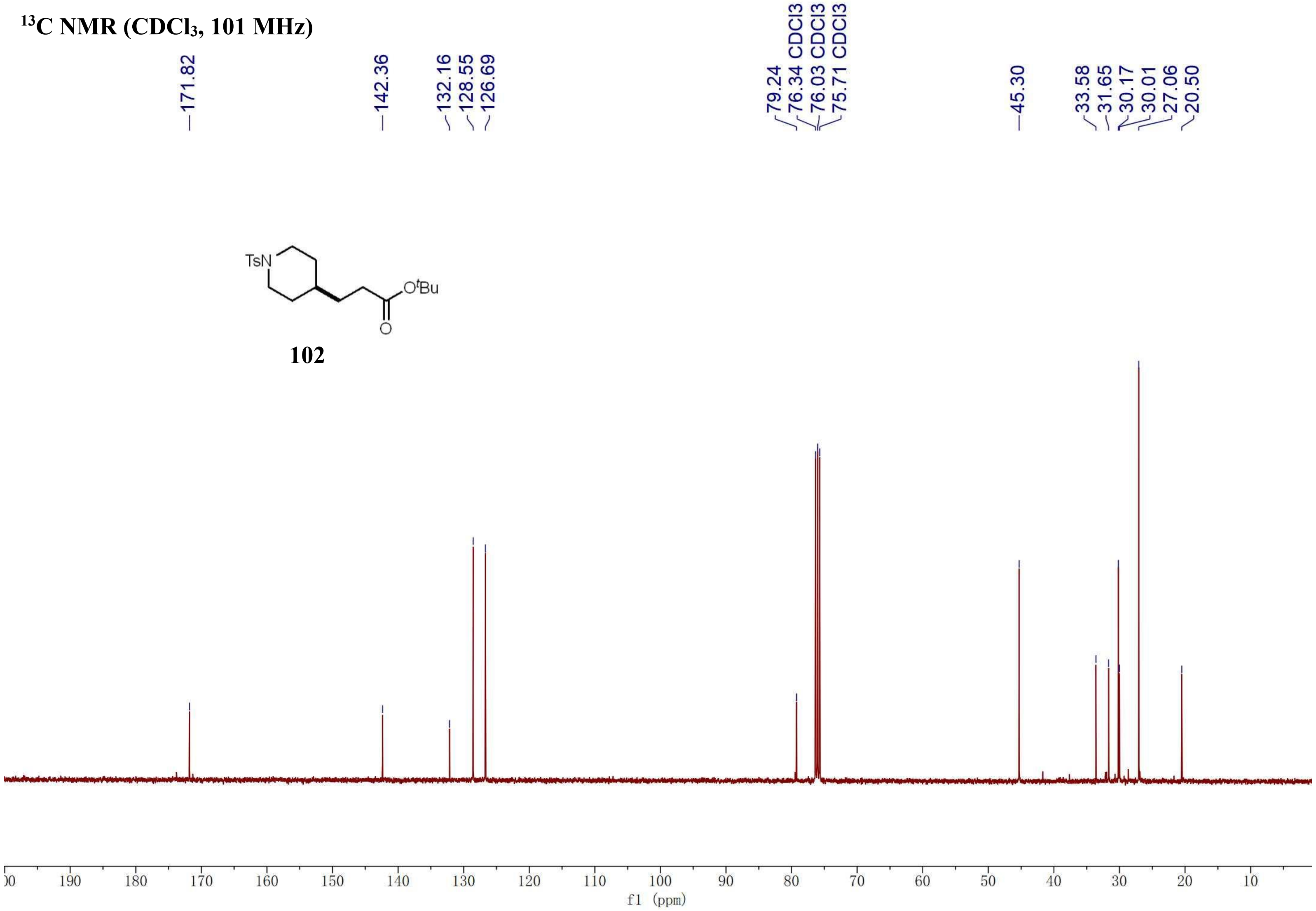

^{1}H NMR ($CDCl_3$, 400 MHz)

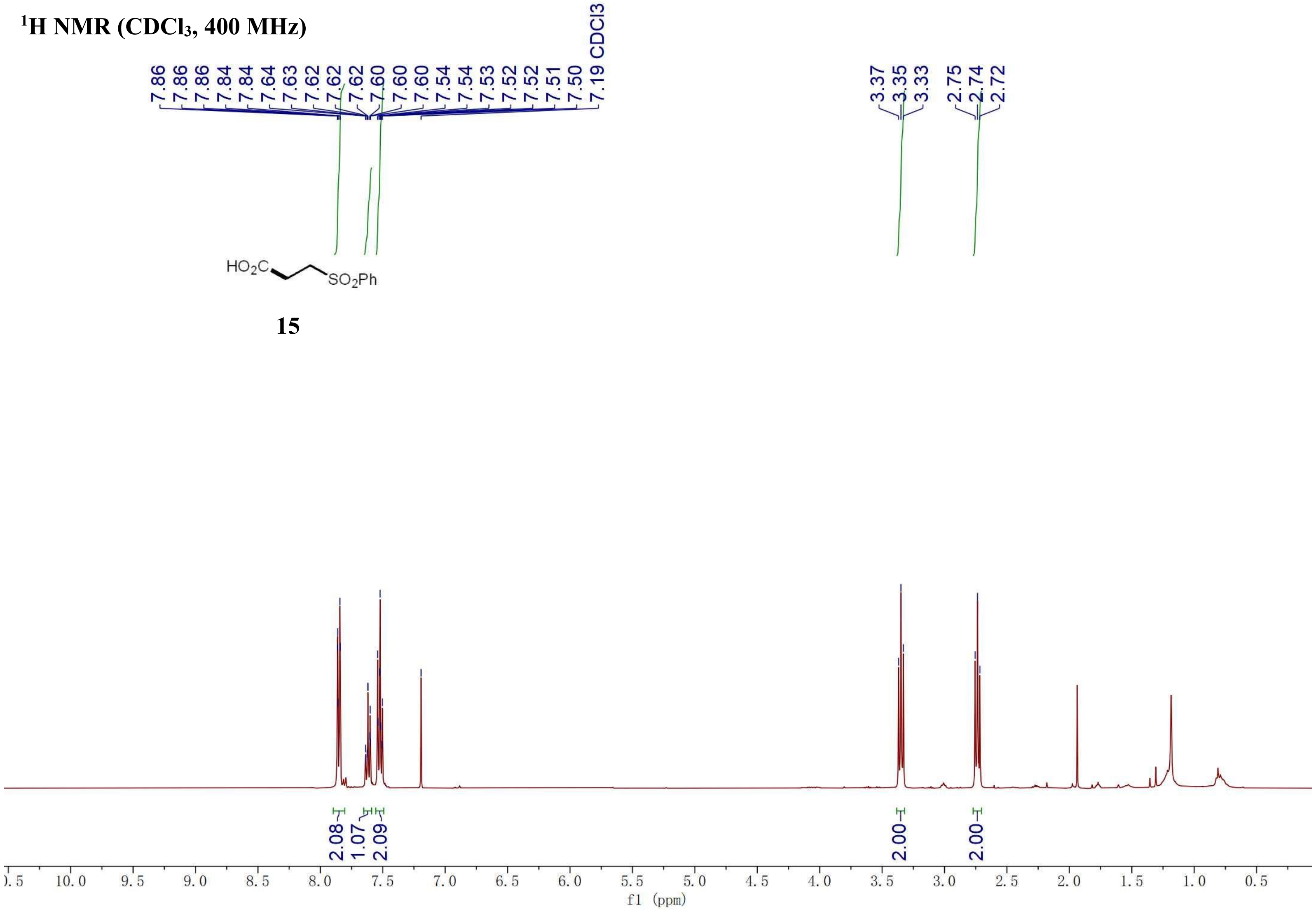

15

^{13}C NMR (CDCl₃, 101 MHz)

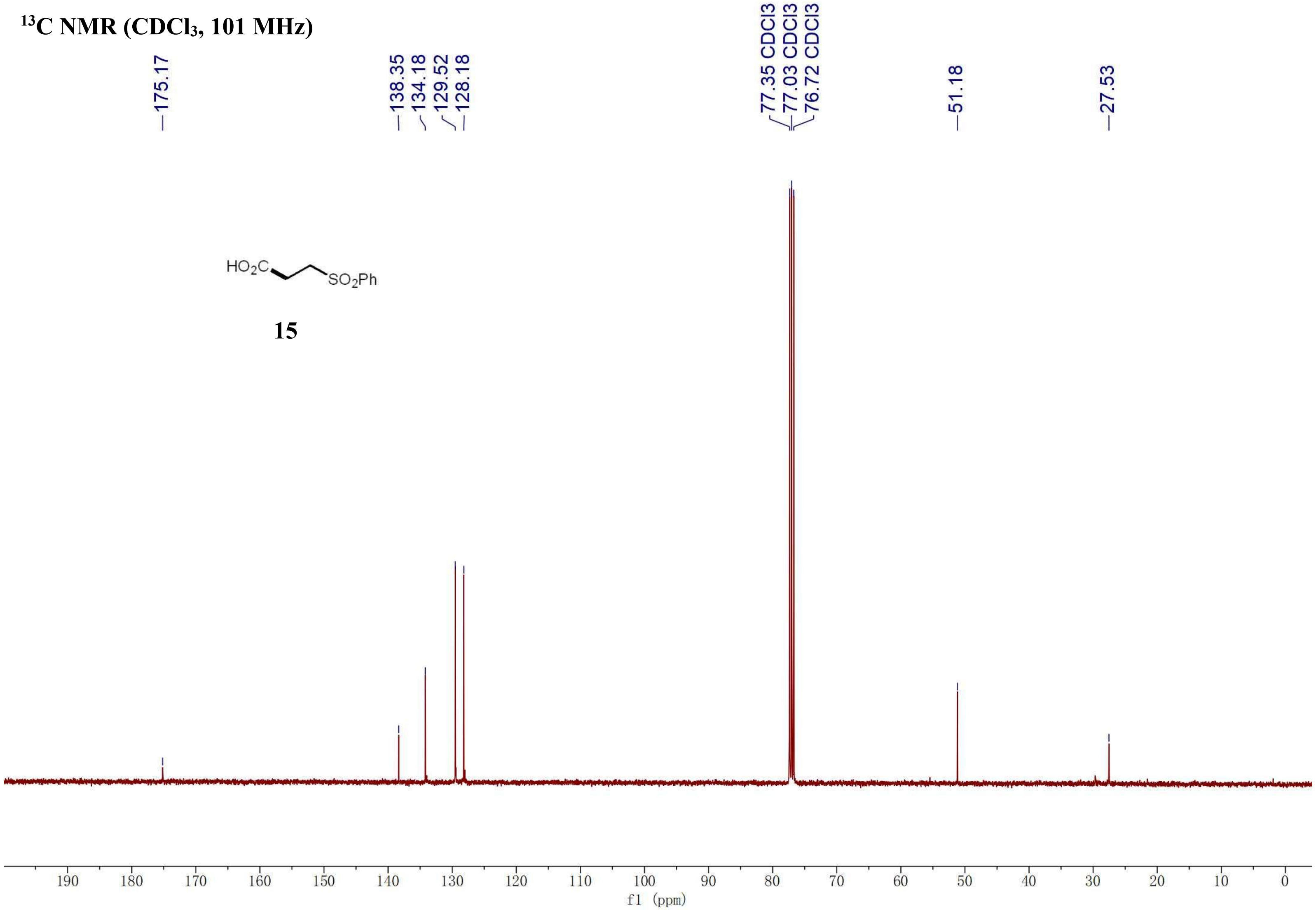

15
